# Supplementary material for: Para-selective nitrobenzene amination lead by C(sp2)-H/N-H oxidative cross-coupling through aminyl radical
Source: Nat Commun. 2024 May 17;15:4186. doi: 10.1038/s41467-024-48540-6 (PMC11101647; doi:10.1038/s41467-024-48540-6)
Supplement: Supplementary file 1 — Supplementary Information [file 41467_2024_48540_MOESM1_ESM.pdf]

Supplementary Information For

Para-Selective Nitrobenzene Amination Lead by C(sp^2)-H/N-H Oxidative Cross-Coupling through Aminyl Radical

Zhen Zhang,[1,‡,*] Shusheng Yue,[1,‡] Bo Jin,[1] Ruchun Yang,[1,2] Shengchun Wang,[3] Tianqi Zhang,[1] Li Sun,[1] Aiwen Lei,[1,3,*] and Hu Cai [1,*]

[1] School of Chemistry and Chemical Engineering, Nanchang University, Jiangxi Province, Nanchang 330031, People's Republic of China; E-mail: zhangzhen@ncu.edu.cn; caihu@ncu.edu.cn

[2] Institute of Organic Chemistry, Jiangxi Science and Technology Normal University

[3] College of Chemistry and Molecular Sciences, The Institute for Advanced Studies (IAS), Wuhan University, Wuhan, Hubei 430072, People's Republic of China; E-mail: aiwenlei@whu.edu.cn

Table of Contents

1. General information S2

2. Synthetic Procedures S2-S15

3. General procedure for transition metal-free nitrobenzene *para*-selectivity C-H amination........ S14-S28

4. Gram-scale synthesis of **3c-10** and denitrative transformations of the NO_2 group S28-S32

5. EPR experiments S33-S33

6. Radical clock experiments S34

7. Theoretical Calculations........ S34-S45

8. Characterization Data of the Compounds........ S46-S103

9. X-Ray Ellipsoid Plots of **3a-6**, **3b-1**, **4a-7**, **4b-8**, **4b-9** and **4c-10**. S104-S109

10. NMR spectra of substrates and products S281-S280

11. Supplementary References........ S281-S286

1. General information

All reactions under standard conditions were carried out under nitrogen and monitored by thin-layer chromatography (TLC) on gel F254 plates. The neutral Al_2O_3 (200-300 meshes) was used for column chromatography with light petroleum ether (b.p. 60~90 °C), dichloromethane and ethyl acetate used as eluents. All solvents were purified and dried by standard techniques and distilled prior to use. ^{1}H NMR, ^{13}C NMR and ^{19}F NMR spectra were recorded in $CDCl_3$ or DMSO-d_6 solution on Agilent ProPulse AM-400 MHz instruments and the spectral data were reported in ppm relative to tetramethylsilane (0.00 ppm) or residual undeuterated solvent $CHCl_3$ (7.26 ppm) and DMSO (2.50 pm) as internal standard for ^{1}H NMR and deuterated solvent $CDCl_3$ (77.0 ppm) and DMSO-d_6 (39.5 ppm) as internal standard for ^{13}C NMR. High-resolution mass spectral analysis (HRMS) data were measured on an Agilent 7890-5975C spectrometer by means of the ESI technique. Electron paramagnetic resonance spectrometer (EPR) data were measured on a EMXplus-9.5/12 and Bruker A200 spectrometer.

Commercially available compounds: **1a-1**, **1a-2**, **1a-4-1a-9**, **1a-12**, **1a-15-1a-17**, **1a-19-1a-25**, **1b-1-1b-11**, **1b-14-1b-18**, **1b-20**, **1b-21**, **1b-24**, **1c-1-1c-14**, **1c-16-1c-24**, **1d-1-1d-14**, **2a-2a-20**, **2b-1-2b-3**.

Known compounds: 7-ethoxyquinoline, **1a-3**, **1a-10**, **1a-11**, **1a-13**, **1a-14**, **1a-18**, **1b-12**, **1b-13**, **1b-19**, **1b-22**, **1c-15**, **3a-1**, **3a-6**, **3b-1-3b-3**, **3b-10**, **3b-15**, **3b-17**, **3b-18**, **3b-20**, **3b-21**, **3c-1-3c-13**, **3d-1-3d-13**, **4a-3**, **4a-10**, **4b-2**, **4b-3**, **4b-6**, **4b-8**, **4b-9**, **4b-12-4b-14**, **4c-1**, **4c-8**, **4c-9**, **4c-10**, **4c-12-4c-14**, **5a**, **5b**, **5c**, **5d**, **5g**, **5h**, **6d**, **6e**, **6g**, **6i**.

2. Synthetic Procedures

2.1 General procedure 1: for the synthesis of 1,2,3,4-tetrahydroquinoline derivatives **1a-10**, **1a-11**, **1a-14**:

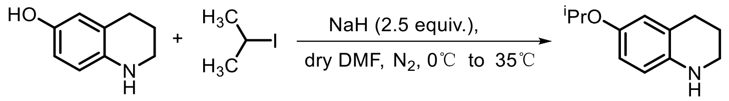

To a three-necked flask of NaH (0.67 g, 16.8 mmol, 2.5 equiv.) in dry DMF (15 mL) at 0°C was added 5-hydroxyquinoline (1.0 g, 6.7 mmol) in dry DMF (15 mL) under nitrogen atmosphere. The mixture was stirred for 1 h and then allowed to warm to room temperature. Iodoisopropane (2.28 g, 13.4 mmol, 2.0 equiv.) was added and the mixture was stirred for 12 h while progress of the reaction was monitored by TLC. The reaction mixture was then poured into ice water. The resulting mixture was extracted with ethyl acetate (50 mL × 3), washed with brine (50 mL), dried over Na_2SO_4 and concentrated in vacuo. Purification of the residue by neutral silica gel column chromatography afforded the desired **1a-10**. **1a-11** and **1a-14** were prepared by this method.[1]

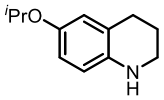

6-isopropoxy-1,2,3,4-tetrahydroquinoline (1a-10)

Prepared according to general procedure 1 (PE: EtOAc = 20:1, v/v) to afford **1a-10** (0.58 g, 45% yield) as a yellow oil. ^{1}H NMR (400 MHz, $CDCl_3$), δ 6.52-6.47 (m, 2H), 6.34 (d, J = 8.0 Hz, 1H), 4.30-4.20 (m, 1H), 3.41 (s, 1H), 3.16 (t, J = 5.4 Hz, 2H), 2.65 (t, J = 6.4 Hz, 2H), 1.87-1.80 (m, 2H), 1.20 (d, J = 6.0 Hz, 6H). ^{13}C NMR (101 MHz, $CDCl_3$) δ 149.7, 138.9, 122.8, 118.0, 115.6, 115.4, 71.0, 42.2, 27.0, 22.3, 22.2. HRMS (m/z): calcd for $C_{12}H_{17}NO$ $[M+H]^+$: 192.1383, Found: 192.1393.

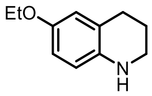

6-ethoxy-1,2,3,4-tetrahydroquinoline (1a-11)

Prepared according to general procedure 1 (PE: EtOAc = 20:1, v/v) to afford **1a-11** (0.53 g, 45% yield) as a yellow oil. ^{1}H NMR (400 MHz, $CDCl_3$), δ 6.53-6.47 (m, 2H), 6.36 (d, J = 8.4 Hz, 1H), 3.86 (q, J = 7.2 Hz, 2H), 3.35 (s, 1H), 3.17 (t, J = 5.2 Hz, 2H), 2.66 (t, J = 6.4 Hz, 2H), 1.88-1.81 (m, 2H), 1.28 (t, J = 7.0 Hz, 2H). ^{13}C NMR (101

MHz, $CDCl_3$) δ 151.1, 138.7, 122.9, 115.8, 115.6, 113.7, 64.0, 42.3, 27.1, 22.4, 15.0. HRMS (ESI-Orbitrap MS) m/z: Calcd. for $C_{11}H_{15}NO$ $[M+H]^+$: 178.12264; Found: 178.12231.

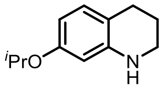

7-isopropoxy-1,2,3,4-tetrahydroquinoline (1a-14)

Prepared according to general procedure 1 (PE: EtOAc = 20:1, v/v) to afford **1a-14** (1.09 g, 85% yield) as a yellow oil. ^{1}H NMR (400 MHz, $CDCl_3$), δ 6.73 (d, J = 8.4 Hz, 1H), 6.10 (dd, J = 8.2, 2.2 Hz, 1H), 5.94 (d, J = 2.4 Hz, 1H), 4.39-4.31 (m, 1H), 3.64 (s, 1H), 3.16 (t, J = 5.4 Hz, 2H), 2.60 (t, J = 6.4 Hz, 2H), 1.85-1.78 (m, 2H), 1.21 (d, J = 6.0 Hz, 6H). ^{13}C NMR (101 MHz, $CDCl_3$) δ 156.8, 145.4, 129.9, 113.9, 104.7, 101.5, 69.5, 41.8, 26.1, 22.3, 22.1. HRMS (m/z): calcd for $C_{12}H_{17}NO$ $[M+H]^+$: 192.1383, Found: 192.1393.

2.2 General procedure 2: for the synthesis of 7-ethoxyquinoline

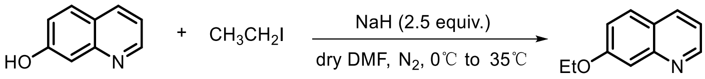

To a three-necked flask of NaH (6.9 g, 172.5 mmol, 2.5 equiv.) in dry DMF (150 mL) at 0°C was added 7-hydroxyquinoline (10.0 g, 69.0 mmol) in dry DMF (150 mL) under nitrogen atmosphere. The mixture was stirred for 1 h and then allowed to warm to room temperature. Iodoethane (21.6 g, 138.0 mmol, 2.0 equiv.) was added and the mixture was stirred for 12 h while progress of the reaction was monitored by TLC. The reaction mixture was then poured into ice water. The resulting mixture was extracted with ethyl acetate (150 mL × 3), washed with brine (150 mL), dried over Na_2SO_4 and concentrated in vacuo. Purification of the residue by neutral silica gel column chromatography afforded the desired 7-ethoxyquinoline.[1]

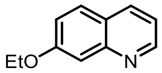

7-ethoxyquinoline [2]

Prepared according to general procedure 2 (PE: EtOAc = 15:1, v/v) to afford 7-ethoxyquinoline (10.9 g, 92% yield) as a yellow oil. ^{1}H NMR (400 MHz, $CDCl_3$), δ 8.69 (dd, J = 4.4, 1.6 Hz, 1H), 7.88 (d, J = 8.0 Hz, 1H), 7.52 (d, J = 8.8 Hz, 1H), 7.28 (d, J = 2.4 Hz, 1H), 7.10-7.03 (m, 2H), 4.04 (q, J = 6.8 Hz, 2H), 1.35 (t, J = 7.0 Hz, 3H). ^{13}C NMR (101 MHz, $CDCl_3$) δ 159.7, 150.2, 149.7, 135.4, 128.5, 123.2, 119.8, 118.6, 107.6, 63.4, 14.4.

2.3 General procedure 3: for the synthesis of 1,2,3,4-tetrahydroquinoline derivatives **1a-13**, **1a-3:**

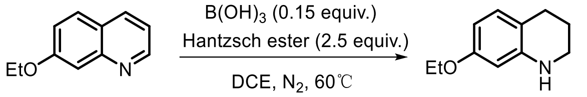

In a three-neck flask (500 mL), 7-ethoxyquinoline (10.9 g, 63.2 mmol), Hantzsch ester (40.0 g, 158.0 mmol, 2.5 equiv.), $B(OH)_3$ (586 mg, 15 mol%) and DCE (200 mL) were added under nitrogen atmosphere. The reaction was stirred at 60 ℃ and then monitored by TLC. After the reaction, the reaction tube was cooled to room temperature, dried over Na_2SO_4 and concentrated in vacuo. Purification of the residue by neutral silica gel column chromatography afforded the desired **1a-13**. Substrate **1a-3** was prepared by this method.[3]

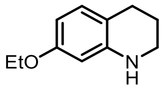

7-ethoxy-1,2,3,4-tetrahydroquinoline (1a-13)

Prepared according to general procedure 3 (PE: EtOAc = 20:1, v/v) to afford **1a-13** (7.87 g, 71% yield) as a yellow oil. ^{1}H NMR (400 MHz, $CDCl_3$), δ 6.72 (d, J = 8.4 Hz, 1H), 6.09 (dd, J = 8.2, 2.6 Hz, 1H), 5.91 (d, J = 2.4 Hz, 1H), 3.84 (q J = 7.2 Hz, 2H), 3.71 (s, 1H), 3.14 (t, J = 5.4 Hz, 2H), 2.58 (t, J = 6.4 Hz, 2H), 1.83-1.76 (m, 2H), 1.26 (t, J = 7.0 Hz, 3H). ^{13}C NMR (101 MHz, $CDCl_3$) δ 157.9, 145.3, 129.9, 113.7, 103.2, 99.9, 63.0, 41.7, 26.1, 22.3, 14.8. HRMS (m/z): calcd for $C_{11}H_{15}NO$ $[M+H]^+$: 178.1226, Found: 178.1229.

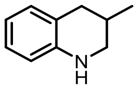

3-methyl-1,2,3,4-tetrahydroquinoline (1a-3) [4]

Prepared according to general procedure 3 (PE: EtOAc = 20:1, v/v) to afford **1a-3** (6.04 g, 96% yield) as a yellow oil. ^{1}H NMR (400 MHz, $CDCl_3$), δ 6.88 (dd, J = 13.2, 7.2 Hz, 2H), 6.53 (t, J = 7.2 Hz, 1H), 6.40 (d, J = 8.0 Hz, 1H), 3.73 (s, 1H), 3.20-3.15 (m, 1H), 2.80 (t, J = 10.4 Hz, 1H), 2.69 (dd, J = 16.0, 3.6 Hz, 1H), 2.35 (dd, J = 16.0, 10.4 Hz, 1H), 2.01-1.93 (m, 1H), 0.96 (d, J = 6.8 Hz, 3H). ^{13}C NMR (101 MHz, $CDCl_3$) δ 144.1, 129.5, 126.6, 121.1, 116.9, 113.9, 48.8, 35.4, 27.1, 19.0.

2.4 General procedure 4: for the synthesis of 1,2,3,4-tetrahydroquinoline derivative **1a-18:**

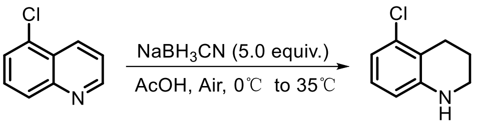

To a round bottom flask was added 5-Chloroquinoline (2.0 g, 12.3 mmol.) and glacial acetic acid (0.15 M). To the resulting solution was added $NaBH_3CN$ (3.86 g, 61.5 mmol, 5.0 equiv.). The flask was capped with a balloon and the reaction mixture was stirred overnight at room temperature. To the flask was then slowly added a saturated aqueous solution of sodium carbonate, along with DCM and the mixture was stirred for further 30 min. The organic layer was separated and the aqueous layer was extracted with DCM (50 mL × 3). dried over Na_2SO_4 and concentrated in vacuo. Purification of the residue by neutral silica gel column chromatography afforded the desired **1a-18**. [5]

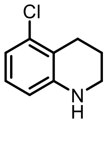

5-chloro-1,2,3,4-tetrahydroquinoline (1a-18) [6]

Prepared according to general procedure 4 (PE: EtOAc = 20:1, v/v) to afford **1a-18** (1.50 g, 73% yield) as a yellow oil. ^{1}H NMR (400 MHz, $CDCl_3$), δ 6.80 (t, J = 8.0 Hz, 1H), 6.60 (d, J = 8.0 Hz, 1H), 6.29 (d, J = 8.0 Hz, 1H), 3.75 (s, 1H), 3.17 (t, J = 5.4 Hz,

2H), 2.70 (t, J = 6.4 Hz, 2H), 1.91-1.84 (m, 2H). ^{13}C NMR (101 MHz, $CDCl_3$) δ 146.2, 134.8, 127.0, 119.2, 117.4, 112.5, 41.3, 24.6, 21.8.

2.5 General procedure 5: for the synthesis of indoline derivatives **1b-12**, **1b-13**, **1b-19** and **1b-22**.

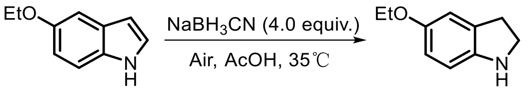

To a solution of 5-ethoxyindole (5.30 g, 33.0 mmol) in AcOH (50 mL) was added $NaBH_3CN$ (8.30 g, 132.0 mmol, 4.0 equiv.) in batches over 30 min at room temperature. The reaction mixture was stirred at room temperature and monitored by TLC. After the complete consumption of 5-ethoxyindole, the reaction was quenched with H_2O (10 mL) and the reaction mixture was basified with solid NaOH. The resulting mixture was extracted with ethyl acetate (100 mL × 3), washed with brine (100 mL), dried over Na_2SO_4 and concentrated in vacuum. Purification of the residue by neutral silica gel column chromatography afforded the desired **1b-12**. Substrates **1b-13**, **1b-19** and **1b-22** were prepared by this method.[7]

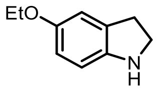

5-ethoxyindoline (1b-12)

Prepared according to general procedure 5 (PE: EtOAc = 30:1, v/v) to afford **1b-12** (3.56 g, 67% yield) as a yellow oil. ^{1}H NMR (400 MHz, $CDCl_3$), δ 6.64 (s, 1H), 6.49-6.41 (m, 2H), 3.83 (q, J = 7.2 Hz, 2H), 3.45 (s, 1H), 3.37 (t, J = 8.4 Hz, 2H), 2.86 (t, J = 8.2 Hz, 2H), 1.25 (t, J = 7.0 Hz, 3H). ^{13}C NMR (101 MHz, $CDCl_3$) δ 152.2, 144.9, 130.6, 112.6, 111.8, 109.6, 63.7, 47.3, 30.0, 14.6. HRMS (m/z): calcd for $C_{10}H_{13}NO$ $[M+H]^+$: 164.1070, Found: 164.1076.

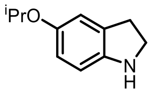

5-isopropoxyindoline (1b-13)

Prepared according to general procedure 5 (PE: EtOAc = 30:1, v/v) to afford **1b-13** (2.56 g, 40% yield) as a yellow oil. ^{1}H NMR (400 MHz, CDCl$_3$), δ 6.67 (s, 1H), 6.53-6.45 (m, 2H), 4.30-4.23 (m, 1H), 3.42 (t, J = 8.4 Hz, 3H), 2.90 (t, J = 8.2 Hz, 2H), 1.20 (d, J = 6.4 Hz, 6H). ^{13}C NMR (101 MHz, CDCl$_3$) δ 151.3, 145.4, 130.9, 115.4, 114.3, 110.0, 71.2, 47.6, 30.3, 22.1. HRMS (m/z): calcd for $C_{11}H_{15}NO$ $[M+H]^+$: 178.1232, Found: 178.1227.

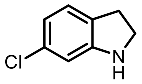

6-chloroindoline(1b-19) [8]

Prepared according to general procedure 5 (PE: EtOAc = 30:1, v/v) to afford **1b-19** (3.06 g, 60% yield) as a yellow oil. ^{1}H NMR (400 MHz, CDCl$_3$), δ 6.88 (d, J = 7.6 Hz, 1H), 6.54 (dd, J = 7.8, 1.8 Hz, 1H), 6.46 (s, 1H), 3.65 (s, 1H), 3.45 (t, J = 8.6 Hz, 2H), 2.87 (t, J = 8.4 Hz, 2H). ^{13}C NMR (101 MHz, CDCl$_3$) δ 152.7, 132.3, 127.6, 125.0, 117.8, 109.0, 47.5, 28.9.

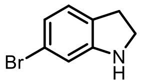

6-bromoindoline(1b-22) [8]

Prepared according to general procedure 5 (PE: EtOAc = 30:1, v/v) to afford **1b-22** (4.40 g, 62% yield) as a yellow oil. ^{1}H NMR (400 MHz, CDCl$_3$), δ 6.85 (d, J = 7.6 Hz, 1H), 6.70 (dd, J = 7.8, 1.4 Hz, 1H), 6.65 (s, 1H), 3.67 (s, 1H), 3.48 (t, J = 8.4 Hz, 2H), 2.88 (t, J = 8.4 Hz, 2H). ^{13}C NMR (101 MHz, CDCl$_3$) δ 153.1, 128.2, 125.6, 120.9, 120.5, 112.0, 47.5, 29.1

2.6 General procedure 6: for the synthesis of 1,2,3,4-tetrahydroquinolines derivatives **5a**, **5b** and **5c**.

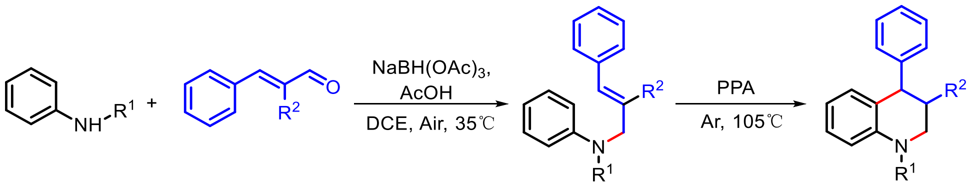

After adding 1,2-dichloroethane (100 mL) to a 250mL dry round-bottomed flask, aniline (4.65 g, 50 mmol, 1.0 equiv.), *trans*-cinnamaldehyde (6.62 g, 50 mmol, 1.0 equiv.) and acetic acid (3.0 g, 50 mmol, 1.0 equiv.) were added in sequence, and sodium triacetoxyborohydride (21.2 g, 100 mmol, 2.0 equiv.), the solution was stirred under atmosphere for 2 h. The reaction was checked by TLC. After all the starting materials were completely converted, the mixture was quenched with saturated aqueous sodium bicarbonate solution. The reaction was diluted by adding DCM. The resulting mixture was extracted with DCM (100 mL × 3), The residue was purified by silica gel column chromatography to give *N*-allylaniline.

N-Allylanilines and polyphosphoric acid (PPA), which is added excess (3 times the mass of *N*-allylanilines), were heated under Ar at 105°C for 6 h. The reaction mixture was cooled to room temperature, and quenched with aqueous ammonia solution. The mixture was diluted by adding EtOAc and water, and stirred for further 30 min. Two layers were separated, and the aqueous layer was further extracted twice more with EtOAc. The combined organic layers were dried over Na_2SO_4, filtered, and concentrated on rotary evaporator. The residue was purified by silica gel column chromatography to give 1,2,3,4-tetrahydroquinolines **5a**, **5b**, **5c** were prepared by this method.[9]

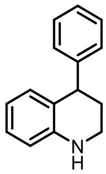

1,2,3,4-tetrahydroquinolines (5a) [10]

Prepared according to general procedure 6 (PE: EtOAc = 20:1, v/v) to afford **5a** (7.11 g, 68% yield) as a yellow liquid. ^{1}H NMR (400 MHz, $CDCl_3$), δ 7.28 (t, J = 7.4 Hz, 2H), 7.19 (t, J = 7.2 Hz, 1H), 7.13 (d, J = 6.8 Hz, 2H), 7.00 (t, J = 7.2 Hz, 1H), 6.74 (d, J = 7.2 Hz, 1H), 6.57-6.51 (m, 2H), 4.13 (t, J = 6.0 Hz, 1H), 3.87 (s, 1H), 3.30-3.17 (m, 2H), 2.24-2.15 (m, 1H), 2.08-1.99 (m, 1H). ^{13}C NMR (101 MHz, $CDCl_3$) δ 146.1, 144.9, 130.4, 128.6, 128.2, 127.3, 126.1, 123.4, 117.0, 114.2, 42.8, 39.2, 31.1.

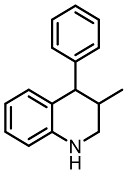

3-methyl-4-phenyl-1,2,3,4-tetrahydroquinoline (5b)

Prepared according to general procedure 6 (PE: EtOAc = 20:1, v/v) to afford **5b** (7.11 g, 68% yield) as a yellow liquid. ^{1}H NMR (400 MHz, $CDCl_3$), δ 7.28-7.11 (m, 5H), 6.97 (s, 1H), 6.62-5.51 (m, 3H), 3.78 (s, 1H), 3.63 (d, J = 8.4 Hz, 1H), 3.24 (d, J = 11.2 Hz, 1H), 2.98 (t, J = 10.0 Hz, 1H), 2.15-2.12 (m, 1H), 0.91 (d, J = 6.8 Hz, 3H). ^{13}C NMR (101 MHz, $CDCl_3$) δ 145.7, 144.6, 130.7, 129.2, 128.2, 127.0, 126.2, 124.0, 117.2, 113.9, 51.2, 47.1, 34.9, 18.0 HRMS (m/z): calcd for $C_{16}H_{17}N$ $[M+H]^+$: 280.1081, Found: 280.1071. HRMS(m/z): calcd for $C_{16}H_{17}N$ $[M+H]^+$:224.1434, Found:224.1443.

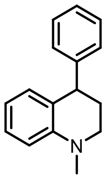

1-methyl-4-phenyl-1,2,3,4-tetrahydroquinoline (5c) [10]

Prepared according to general procedure 6 (PE: EtOAc = 20:1, v/v) to afford **5c** (7.11 g, 68% yield) as a yellow liquid. ^{1}H NMR (400 MHz, $CDCl_3$), δ 7.25 (t, J = 7.6 Hz, 2H), 7.17 (t, J = 7.2 Hz, 1H), 7.12-7.07 (m, 3H), 6.72 (d, J = 7.6 Hz, 1H), 6.65 (d, J = 8.4 Hz, 1H), 6.54 (t, J = 7.2 Hz, 1H), 4.10 (t, J = 6.2 Hz, 1H), 3.21-3.08 (m, 2H), 2.90 (s, 3H), 2.26-2.18 (m, 1H), 2.11-2.02 (M, 1H). ^{13}C NMR (101 MHz, $CDCl_3$) δ 146.8, 146.5, 129.8, 128.6, 128.2, 127.5, 126.0, 124.7, 116.2, 110.9, 48.4, 43.3, 39.2, 31.0.

2.7 General procedure 7: for the synthesis of compounds **6a** and **6b**.

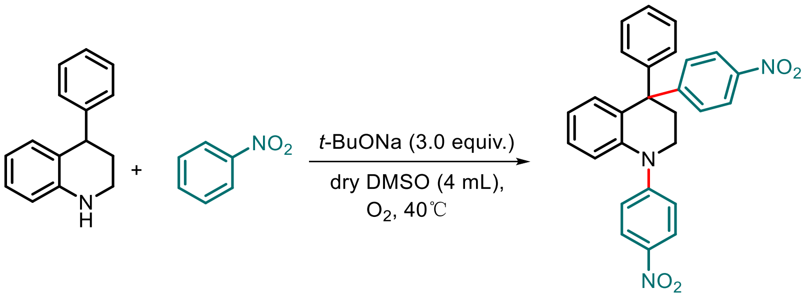

1,2,3,4-tetrahydroquinolines **5a** (104.5 mg, 0.5 mmol, 1.0 equiv.), nitrobenzene (123.2 mg, 1.0 mmol, 2.0 equiv.) and *t*-BuONa (144.2 mg, 1.5 mmol, 3.0 equiv.) were added in dry DMSO (4 mL) at room temperature under O_2 (1.0 atm) atmosphere. The reaction mixture was stirred at 40℃ and monitored by TLC. After complete consumption of **5a**, the reaction mixture was returned to room temperature, then quenched with water (5 mL), diluted with ethyl acetate, and extracted with ethyl acetate (25 mL x 3) The combined organic phases were washed with brine (5 mL), dried over Na_2SO_4 and concentrated in vacuo. The residue was purified by neutral Al_2O_3 column chromatography to give compound **6a** as yellow solid. Substrates **6b** and **6c** were prepared by this method.

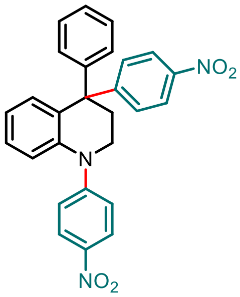

1,4-bis(4-nitrophenyl)-4-phenyl-1,2,34-terahroquinoline (6a)

Prepared according to general procedure 7 (PE: EtOAc = 10:1, v/v) from **5a** (104.5 mg) to afford **6b** (92.5 mg, 41% yield) as a yellow solid (m.p. 94-96 °C). ^{1}H NMR (400 MHz, $CDCl_3$), δ 8.10 (d, *J* = 7.2 Hz, 2H), 8.01 (d, *J* = 7.6 Hz, 2H), 7.34 (d, *J* = 8.0 Hz, 2H), 7.31-7.21 (m, 6H), 7.05-6.99 (m, 4H), 6.95 (d, *J* = 7.6 Hz, 1H), 6.58 (d, *J* = 7.6 Hz, 1H), 3.55-3.42 (m, 2H), 2.93 (t, *J* = 6.4 Hz, 2H). ^{13}C NMR (101 MHz, $CDCl_3$) δ 152.7, 152.2, 146.7 143.2, 140.3, 139.8, 137.1, 129.6, 128.9, 128.9, 128.4, 128.0, 127.4, 125.3, 123.3, 123.0, 122.2, 115.7, 51.8, 45.0, 36.8. HRMS(m/z): calcd for $C_{27}H_{21}N_3O_4$ $[M+H]^+$: 451.1532, Found:451.1534.

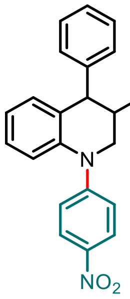

3-methyl-1-(4-nitrophenyl)-4-phenyl-1,2,3,4-tetrahydroquinoline (6b)

Prepared according to general procedure 7 (PE: EtOAc = 10:1, v/v) from **5b** (111.6 mg) to afford **6b** (129.1 mg, 75% yield) as yellow solid (m.p. 114-116 °C). ^{1}H NMR (400 MHz, $CDCl_3$), δ 8.08 (d, J = 9.2 Hz, 2H), 7.25 (t, J = 7.6 Hz, 2H), 7.21-7.14 (m, 4H), 7.06 (d, J = 7.6 Hz, 2H), 7.01 (t, J = 7.4 Hz, 1H), 6.82-6.74 (m, 2H), 3.84 (dd, J = 12.0, 4.0 Hz, 1H), 3.61 (d, J = 8.8 Hz, 1H), 3.32 (dd, J = 11.8, 9.4 Hz, 1H), 3.21-3.11 (m, 1H), 0.88 (d, J = 6.4 Hz, 3H). ^{13}C NMR (101 MHz, $CDCl_3$) δ 153.5, 143.9, 140.6, 140.3, 131.8, 129.0, 128.5, 126.6(4), 126.6(7), 125.5, 122.7, 119.7, 118.5, 54.9, 51.5, 36.7, 17.9. HRMS(m/z): calcd for $C_{22}H_{20}N_2O_2$ $[M+H]^+$: 345.1598, Found:345.1591

2.8 General procedure 8: for the synthesis of compound **6d**.

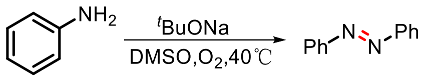

Aniline **5d** (18.6 mg, 0.2 mmol) and ^{t}BuONa (19.2 mg, 0.2 mmol, 1.0 equiv.) were added to dry DMSO (2 mL) at room temperature under O_2 The reaction mixture was stirred at 40°C for 1h and monitored by TLC. After the complete consumption of **5d**, the reaction mixture was cooled to room temperature and then quenched with water (2 mL × 1), and extracted with ethyl acetate (25 mL × 3). The combined organic phases were washed with brine (5 mL × 1), dried over Na_2SO_4 and concentrated in vacuo. The residue was purified by neutral silica gel column chromatography. Product **6d** (15.3 mg, 84%) was obtained as a yellow solid.

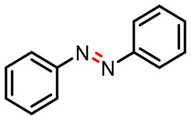

1,2-diphenyldiazene (6d) [11]

Prepared according to general procedure 8 from **5d** (18.6 mg) (PE: EtOAc = 30:1, v/v) to afford **6d** (15.3 mg, 84% yield) as a yellow solid (m.p. 65-67 °C). **^{1}H NMR** (400 MHz, $CDCl_3$): δ 7.99-7.94 (m, 4H), 7.58-7.49 (m, 6H); **^{13}C NMR** (101 MHz, $CDCl_3$): δ 152.7, 131.0, 129.1, 122.8.

2.9 General procedure 10: for the synthesis of *N*-Cyclopropylaniline **5g**. [12]

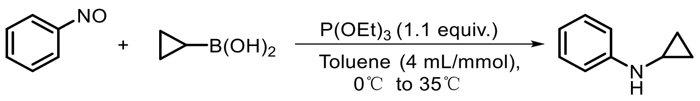

$P(OEt)_3$ (3.66 g, 22 mmol, 1.1 equiv.) was added to a solution of nitrosobenzene (2.15 g, 20 mmol, 1.0 equiv.) and cyclopropylboronic acid (2.58 g, 30 mmol, 1.5 equiv.) in toluene (80 mL), and the mixture was stirred at room temperature under air for 2.0 h. After the complete consumption of nitrosobenzene, the reaction mixture was concentrated in vacuum. Purification of the residue by neutral silica gel column chromatography with hexane/AcOEt = 9:1 afforded the desired **5g** as a colorless liquid.

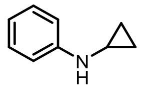

***N*-Cyclopropylaniline (5g)** [12]

Prepared according to general procedure 10 (PE: EtOAc = 50:1, v/v) to afford **5g** (1.86 g, 70% yield) as a colorless liquid. ^{1}H NMR (400 MHz, $CDCl_3$), δ 7.34 (d, J = 7.4 Hz, 2H), 6.93-6.86 (m, 3H), 4.18 (s, 1H), 2.56-2.50 (m, 1H), 0.86-0.81 (m, 2H), 0.65-0.61 (m, 2H). ^{13}C NMR (101 MHz, $CDCl_3$) δ 129.0, 122.2, 117.6, 113.0, 25.1, 7.3.

2.11 General procedure 11: for the synthesis of *N*-Cyclopropylaniline **6h**.

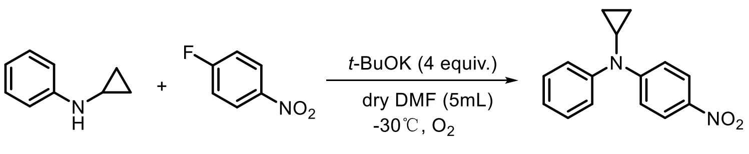

N-Cyclopropylaniline **5g** (66.6 mg, 0.5 mmol, 1.0 equiv.), 4-Fluoronitrobenzene (211.5 mg, 1.5 mmol, 3.0 equiv.) and ^{t}BuOK (224.5 mg, 2.0 mmol, 4.0 equiv.) were added in dry DMF (5 mL) at room temperature under O_2 (1.0 atm) atmosphere. The

reaction mixture was stirred at -30°C and monitored by TLC. After the complete consumption of **5g**, the reaction mixture was warmed to room temperature and then quenched with water (5 mL), diluted with ethyl acetate, and extracted with ethyl acetate (25 mL × 3). The combined organic phases were washed with brine (5 mL), dried over Na_2SO_4 and concentrated in vacuo. The residue was purified by neutral Al_2O_3 column chromatography to give compound **6h** as a yellow solid.

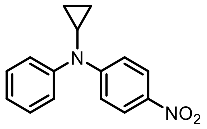

***N*-cyclopropyl-4-nitro-*N*-phenylaniline (6h)**

Prepared according to general procedure 11 (PE: EtOAc = 30:1, v/v) from **5g** (66.6 mg) to afford **6h** (89.0 mg, 70% yield) as a yellow solid (m.p. 114-116 °C). ^{1}H NMR (400 MHz, $CDCl_3$), δ 8.04 (d, J = 9.2 Hz, 2H), 7.45 (t, J = 7.8 Hz, 2H), 7.32 (t, J = 9.0 Hz, 2H), 7.14 (d, J = 8.0 Hz, 2H), 6.90 (t, J = 9.2 Hz, 2H), 2.94-2.87 (m, 1H), 0.96 (q, J = 6.0 Hz, 2H), 0.68-0.63 (m, 2H). ^{13}C NMR (101 MHz, $CDCl_3$) δ 154.6, 144.2, 138.8, 129.9, 127.9, 126.9, 125.4, 113.2, 32.8, 9.6.

2.12 General procedure 12: for the synthesis of *N*-phenylpropan-1-imine **5h**. [13a]

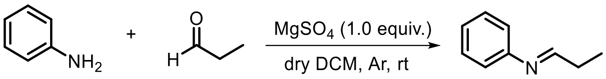

A mixture of a propionaldehyde (1.16 g, 20 mmol, 2.0 equiv.), phenylamine (0.93 g, 10 mmol, 1.0 equiv.) and $MgSO_4$ (1.20 g, 10 mmol, 1.0 equiv.) in dry DCM (20 mL) was stirred for 2.0 h under Ar. Upon completion of the reaction, the reaction mixture was filtered and concentrated in vacuo, and proceed directly to the next reaction.

2.13 General procedure 13: for the synthesis of *N*-methyl-4-(1*H*-pyrrol-1-yl)aniline **1c-15**. [13b]

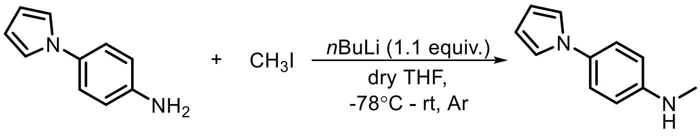

A solution of 4-(1*H*-pyrrol-1-yl)aniline (3.16 g, 20.0 mmol, 1.0 equiv.) in dry THF (50 mL) was prepared and cooled to -78 °C. nBuLi (8.8 mL, 2.5 M in hexane, 22.0 mmol, 1.1 equiv.) was then added dropwise to the reaction mixture at -78 °C and stirred for 30 min under Ar. Then CH_3I (1.38 mL, 22.0 mmol, 1.1 equiv.) was added dropwise over 5 min. The reaction mixture was warmed to rt and stirred for 2 h. Water (60 mL) was added to quench the reaction. The reaction mixture was extracted with AcOEt (3 x 60 mL). The combined organic solution was dried over anhydrous Na_2SO_4, filtered and evaporated under reduced pressure. The residue was purified by flash column chromatography on silica gel (PE: EtOAc = 10:1, v/v) to afford **1c-15** as yellow solid (1.0 g, 31% yield).

3. General procedure for transition metal-free nitrobenzene *para*-selectivity C-H amination

3.1. General procedure A: for synthesis of nitrobenzene C-H amination product **3a-1**

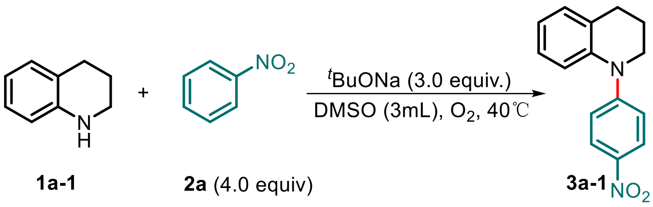

1a-1 **2a** (4.0 equiv) **3a-1**

1,2,3,4-tetrahydroquinoline **1a-1** (66.6 mg, 0.5 mmol), nitrobenzene (246.3 mg, 2.0 mmol, 4.0 equiv.) and ^{t}BuONa (144.2 mg, 1.5 mmol, 3.0 equiv.) were added in dry DMSO (3 mL) solvent at room temperature under O_2 (1.0 atm) atmosphere. The reaction mixture was stirred at 40°C and monitored by TLC. After the complete consumption of **1a-1**, the reaction mixture was cooled to room temperature and then quenched with water (5 mL), diluted with ethyl acetate, and extracted with ethyl acetate (25 mL × 3). The combined organic phases were washed with brine (5 mL), dried over Na_2SO_4 and concentrated in vacuo. The residue was purified by neutral Al_2O_3 column chromatography (PE: EtOAc = 50:1, v/v). Compound **3a-1** (90.3 mg, 71%) was obtained as a yellow solid. Products **3a-1-3a-21**, **3b-23, 3c-1-3c-13**, **4a-1-4a-14**, **4c-1**, **4c-2**, **4c-4-4c-14**, **6g** were prepared by this method.

3.2 Optimization of the Reaction Conditions

Supplementary Table 1, Optimization of the reaction of 1,2,3,4-tetrahydroquinoline **1a-1** with nitrobenzene **2a**.[a]

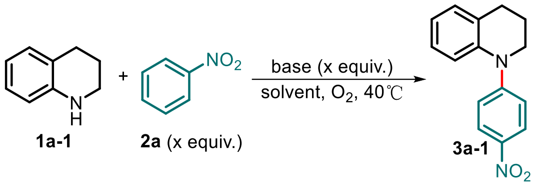

entry	**2a** (equiv.)	Solvent (mL)	Base (equiv.)	Yield (%) [b]
1	4.0	CH_3CN (5.0)	^{t}BuONa (2.0)	N.R.
2	4.0	Toluene (5.0)	^{t}BuONa (2.0)	N.R.
3	4.0	THF (5.0)	^{t}BuONa (2.0)	N.R.
4	4.0	DMF (5.0)	^{t}BuONa (2.0)	36
5	4.0	DMSO (5.0)	^{t}BuONa (2.0)	50
6	3.0	DMSO (5.0)	^{t}BuONa (2.0)	43
7	5.0	DMSO (5.0)	^{t}BuONa (2.0)	49
8	4.0	DMSO (5.0)	^{t}BuONa (3.0)	57
9	4.0	DMSO (5.0)	^{t}BuONa (4.0)	56
10	4.0	DMSO (5.0)	none	N.R.
11	4.0	DMSO (5.0)	Na_2CO_3 (3.0)	N.R.
12	4.0	DMSO (5.0)	NaOH (3.0)	N.R.
13	4.0	DMSO (5.0)	CH_3ONa (3.0)	25
14	4.0	DMSO (5.0)	^{t}BuOLi (3.0)	N.R.
15	4.0	DMSO (5.0)	^{t}BuOK (3.0)	36
16	4.0	DMSO (4.0)	^{t}BuONa (3.0)	60
17	4.0	DMSO (3.0)	^{t}BuONa (3.0)	71
18	4.0	DMSO (2.0)	^{t}BuONa (3.0)	69
19 [c]	4.0	DMSO (3.0)	^{t}BuONa (3.0)	40
20 [d]	4.0	DMSO (3.0)	^{t}BuONa (3.0)	Trace

[a] Reaction conditions: **1a-1** (0.5 mmol), **2a** (3.0-5.0 equiv.), base (2.0-4.0 equiv.), solvent (2.0-5.0 mL), O_2 (1.0 atm) and the reaction was conducted at 40 °C; [b] Isolated yield; [c] Air; [d] Deoxygenation by lyophilization for five times, N_2.

Supplementary Table 2, Screening of reaction solvent. [a]

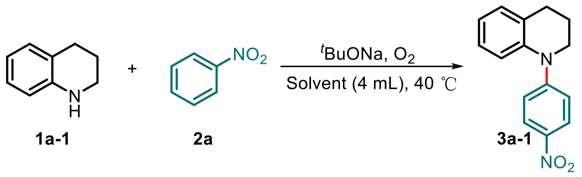

Entry	Solvent	Yield (%) [b]
1	CH_3CN	N.R.
2	Toluene	N.R.
3	THF	N.R.
4	DMF	36
5	DMSO	50

[a] Reaction conditions: **1a-1** (66.6 mg, 0.5 mmol), **2a** (246.3 mg, 2.0 mmol, 4.0 equiv.), ^{t}BuONa (96.1 mg, 1.0 mmol, 2.0 equiv.) in solvent (5 mL) and stirred for 1.5 h, 40 °C, O_2 (1.0 atm); [b] Isolated yield.

Supplementary Table 3, Screening of nitrobenzene equivalent. [a]

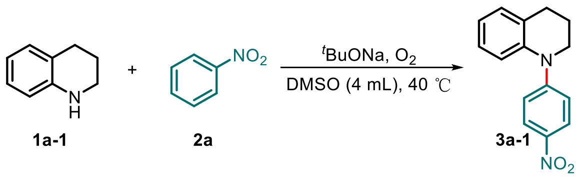

Entry	**2a** (X mmol)	Yield (%) [b]
1	0.50	23
2	0.75	27
3	1.00	33
4	1.25	44
5	1.50	43
6	1.75	44
7	2.00	50
8	2.50	49

[a] Reaction conditions: **1a-1** (66.6 mg, 0.5 mmol), ^{t}BuONa (96.1 mg, 1.0 mmol, 2.0 equiv.) was stirred for 1.5 h in DMSO (5 mL), 40 °C, O_2 (1.0 atm); [b] Isolated yield.

Supplementary Table 4, Screening of base equivalent. [a]

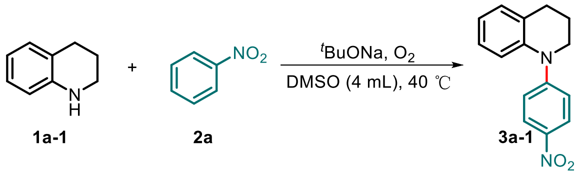

Entry	*t*-BuONa (X mmol)	Yield (%) [b]
1	0.50	26
2	0.75	43
3	1.00	50
4	1.25	55
5	1.50	57
6	1.75	56
7	2.00	57

[a] Reaction conditions: **1a-1** (66.6 mg, 0.5 mmol), **2a** (246.3 mg, 2.0 mmol, 4.0 equiv.), stirring for 1.5 h in DMSO (5 mL) as solvent, 40 °C, O_2 (1.0 atm); [b] Isolated yield.

Supplementary Table 5, Screening of Base. [a]

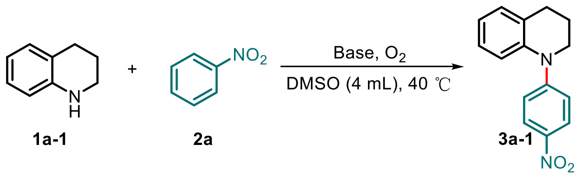

Entry	Base (3.0 equiv.)	Yield (%) [b]
1	K_2CO_3	NR
2	Na_2CO_3	NR
3	KOH	NR
4	NaOH	NR
5	^{t}BuOLi	NR
6	^{t}BuOK	36
7	^{t}BuONa	57
8	CH_3ONa	25
9	C_2H_5ONa	Trace

[a] Reaction conditions: **1a-1** (66.6 mg, 0.5 mmol), **2a** (246.3 mg, 2.0 mmol, 4.0 equiv.), Base (1.5 mmol, 3.0 equiv.) in DMSO (5 mL) and stirred for 1.5 h, 40 °C, O_2 (1.0 atm); [b] Isolated yield.

Supplementary Table 6, Screening of reaction temperature. [a]

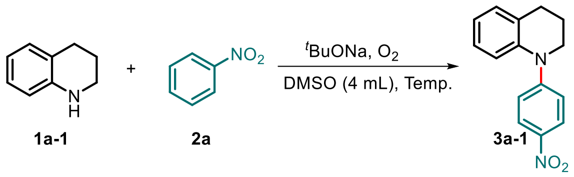

Entry	T/°C	Yield (%) [b]
1	30	41
2	40	57
3	60	33
4	80	41
5	100	19
6	120	42
7	140	33

[a] Reaction conditions: **1a-1** (66.6 mg, 0.5 mmol), **2a** (246.3 mg, 2.0 mmol, 4.0 equiv.), ^{t}BuONa (144.2 mg, 1.5 mmol, 3.0 equiv.) in DMSO (5 mL) and stirring for 1.5 h, O_2; [b] Isolated yield.

Supplementary Table 7, Screening of reaction concentration. [a]

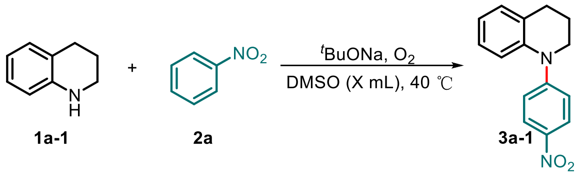

Entry	DMSO (X mL)	Yield (%) [b]
1	2.0	69
2	3.0	71
3	4.0	57
4	5.0	57
5	6.0	55

[a] Reaction conditions: **1a-1** (66.6 mg, 0.5 mmol), **2a** (246.3 mg, 2.0 mmol, 4.0 equiv.), ^{t}BuONa (144.2 mg, 1.5 mmol, 3.0 equiv.), stirred for 1.5 h in DMSO (X mL), 40 °C, O_2 (1.0 atm); [b] Isolated yield.

Supplementary Table 8, Screening of reaction atmosphere. [a]

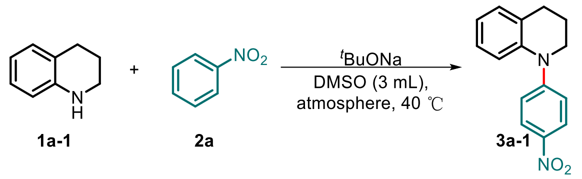

Entry	Atmosphere	Yield (%) [b]
1	Air	40
2	N_2[c]	Trace
3	O_2	71

[a] Reaction conditions: **1a-1** (66.6 mg, 0.5 mmol), **2a** (246.3 mg, 2.0 mmol, 4.0 equiv.), *t*BuONa (144.2 mg, 1.5 mmol, 3.0 equiv.) in DMSO (3 mL) and stirred for 1.5 h, 40 °C, O_2 (1.0 atm); [b] Isolated yield. [c] Deoxygenation by lyophilization five times, N_2.

Supplementary Table 9, Screening of reaction time. [a]

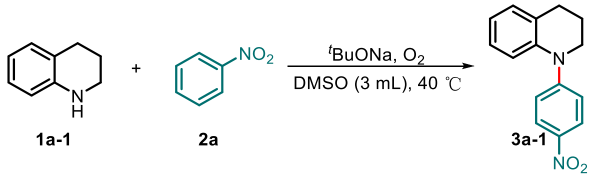

Entry	Reaction time (h)	Yield (%) [b]
1	0.5	62
2	1.0	63
3	1.5	71
4	2.0	68
5	2.5	70
6	3.0	57
7	3.5	58
8	4.0	58

[a] Reaction conditions: **1a-1** (66.6 mg, 0.5 mmol), **2a** (246.3 mg, 2.0 mmol, 4.0 equiv.), *t*BuONa (144.2 mg, 1.5 mmol, 3.0 equiv.) in DMSO (3 mL), stirring, 40 °C, O_2 (1.0 atm); [b] Isolated yield.

3.2. General procedure B: for synthesis of nitrobenzene C-H amination product **3b-1**

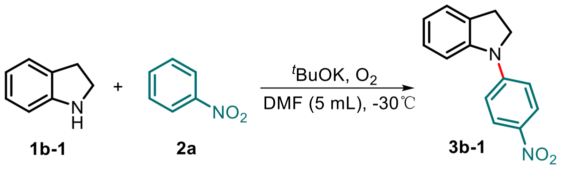

Nitrobenzene (184.7 mg, 1.5 mmol, 3.0 equiv.) was added to dry DMF (5 mL) of ^{t}BuOK (224.5 mg, 2.0 mmol, 4.0 equiv.) at room temperature in argon atmosphere. After the reaction mixture was stirred at -30 °C for 10 min, argon was changed to O_2 (1.0 atm) atmosphere. After indoline **1b-1** (59.6 mg, 0.5 mmol) was added, it was stirred at -30 °C and monitored by TLC. After complete conversion of **1b-1**, the reaction mixture was restored to room temperature and then quenched with water. diluted with ethyl acetate, and extracted with ethyl acetate (25 mL × 3). The combined organic phases were washed with saturated NaCl aqueous solution. The combined organic phases were washed with brine (5 mL), dried over Na_2SO_4 and concentrated in vacuo. The residue was purified by neutral Al_2O_3 column chromatography (PE: EtOAc = 40:1, v/v). Compound **3b-1** (102.1 mg, 85%) was obtained as a yellow solid. Products **3b-1-3b-22**, **4b-1-4b-14**, **4c-3** were prepared by this method.

Supplementary Table 10, Optimization of the reaction of indoline **1b-1** and nitrobenzene **2a**. [a]

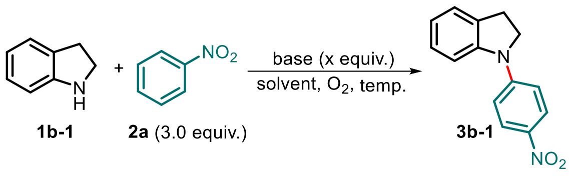

entry	Temp. (°C)	Solvent (mL)	Base (equiv.)	Yield (%) [b]
1	40	CH_3CN (5.0)	^{t}BuONa (4.0)	N.R.
2	40	Toluene (5.0)	^{t}BuONa (4.0)	N.R.
3	40	THF (5.0)	^{t}BuONa (4.0)	N.R.
4	40	DMF (5.0)	^{t}BuONa (4.0)	25
5	40	DMSO (5.0)	^{t}BuONa (4.0)	48
6	40	DMSO (5.0)	none	N.R.
7	40	DMSO (5.0)	^{t}BuOLi (4.0)	N.R.
8	40	DMSO (5.0)	^{t}BuOK (4.0)	52
9	0	DMF (5.0)	^{t}BuOK (4.0)	34
10	-20	DMF (5.0)	^{t}BuOK (4.0)	65
11	-30	DMF (5.0)	^{t}BuOK (4.0)	85
12	-40	DMF (5.0)	^{t}BuOK (4.0)	60
13	-30	DMF (5.0)	^{t}BuOK (2.0)	81
14	-30	DMF (5.0)	^{t}BuOK (3.0)	77
15	-30	DMF (5.0)	^{t}BuOK (5.0)	75
16	-30	DMF (4.0)	^{t}BuOK (4.0)	70
17	-30	DMF (6.0)	^{t}BuOK (4.0)	63
18 [c]	-30	DMF (5.0)	^{t}BuOK (4.0)	31
19 [d]	-30	DMF (5.0)	^{t}BuOK (4.0)	Trace

[a] Reaction conditions: **1b-1** (0.5 mmol), **2a** (3.0 equiv.), base (2.0-5.0 equiv.), solvent (4.0-6.0 mL), O_2 (1.0 atm) and the reaction was conducted at different temperature; [b] Isolated yield; [c] Air; [d] Deoxygenation by lyophilization five times, N_2.

Supplementary Table 11, Screening of reaction solvent. [a]

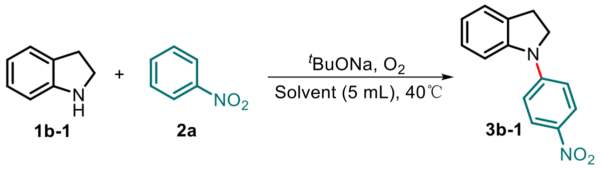

Entry	Solvent	Yield (%) [b]
1	DMSO	48
2	Toluene	N.R.
3	CH_3CN	N.R.
4	THF	N.R.
5	DMF	25
6	1,4-dioxane	N.R.

[a] Reaction conditions: **1b-1** (59.6 mg, 0.5 mmol), **2a** (184.7 mg, 1.5 mmol, 3.0 equiv.), *t*BuONa (192.2 mg, 2.0 mmol, 4.0 equiv.) in solvent (5 mL) and stirring for 5 h, 40 °C, O_2 (1.0 atm); [b] Isolated yield.

Supplementary Table 12, Screening of base. [a]

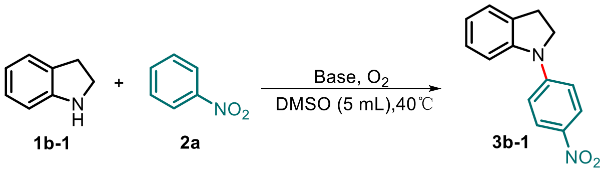

Entry	Base (2.0 mmol)	Yield (%) [b]
1	CH_3ONa	Trace
2	C_2H_5ONa	Trace
3	NaOH	N.R.
4	KOH	N.R.
5	K_2CO_3	N.R.
6	Na_2CO_3	N.R.
7	Et_3N	N.R.
8	*t*BuOLi	N.R.
9	*t*BuOK	52
10	*t*BuONa	48

[a] Reaction conditions: **1b-1** (59.6 mg, 0.5 mmol), **2a** (184.7 mg, 1.5 mmol, 3.0 equiv.), base (2.0 mmol, 4.0 equiv.) in DMSO (5 mL), stirring for 5 h, 40 °C, O_2 (1.0 atm); [b] Isolated yield.

Supplementary Table 13, Screening of reaction temperature. [a]

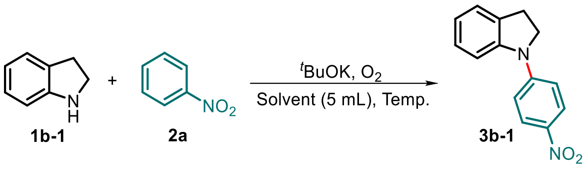

Entry	Solvent	T/°C	Yield (%) [b]
1	DMSO	80	21
2	DMSO	60	25
3	DMSO	40	52
4	DMSO	25	50
5	DMF	0	34
6	DMF	-10	43
7	DMF	-20	65
8	DMF	-30	85
9	DMF	-40	60
10	DMF	-50	55

[a] Reaction conditions: **1b-1** (59.6 mg, 0.5 mmol), **2a** (184.7 mg, 1.5 mmol, 3.0 equiv.), ^{t}BuOK (224.5 mg, 2.0 mmol, 4.0 equiv.) in solvent (5 mL) and stirred for 5 h, O_2 (1.0 atm); [b] Isolated yield.

Supplementary Table 14, Screening of base equivalent. [a]

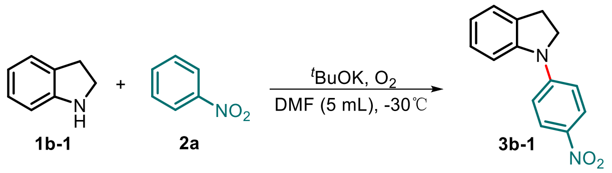

Entry	^{t}BuOK (X mmol)	Yield (%) [b]
1	0.5	22
2	1.0	81
3	1.5	77
4	2.0	85
5	2.5	75
6	3.0	74

[a] Reaction conditions: **1b-1** (59.6 mg, 0.5 mmol), **2a** (184.7 mg, 1.5 mmol, 3.0 equiv.), stirring for 17 h in DMF (5 mL), -30 °C, O_2 (1.0 atm); [b] Isolated yield.

Supplementary Table 15, Screening of nitrobenzene equivalent. [a]

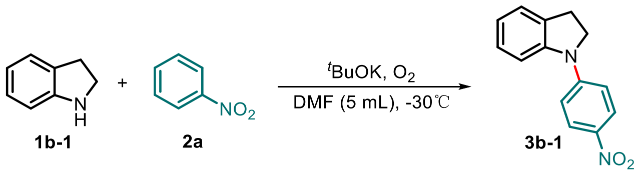

Entry	**2a** (X mmol)	Yield (%) [b]
1	0.5	21
2	1.0	46
3	1.5	85
4	2.0	84
5	2.5	84
6	3.0	82

[a] Reaction conditions: **1b-1** (59.6 mg, 0.5 mmol), ^{t}BuOK (224.5 mg, 2.0 mmol, 4.0 equiv.) in DMF (5 mL), stirring for 17 h, -30 °C, O_2 (1.0 atm); [b] Isolated yield.

Supplementary Table 16, Screening of reaction concentration. [a]

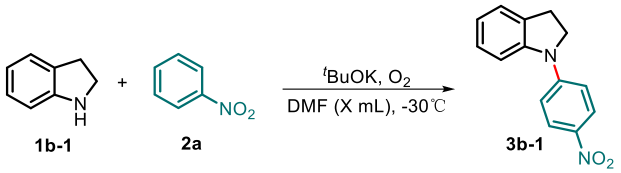

Entry	DMF (X mL)	Yield(%) [b]
1	1.0	65
2	2.0	71
3	3.0	72
4	4.0	70
5	5.0	85
6	6.0	63

[a] Reaction conditions: **1b-1** (59.6 mg, 0.5 mmol), **2a** (184.7 mg, 1.5 mmol, 3.0 equiv.), ^{t}BuOK (224.5 mg, 2.0 mmol, 4.0 equiv.) in DMF (X mL) as the solvent 17 h, -30 °C, O_2 (1.0 atm); [b] Isolated yield.

Supplementary Table 17, Screening of reaction atmosphere. [a]

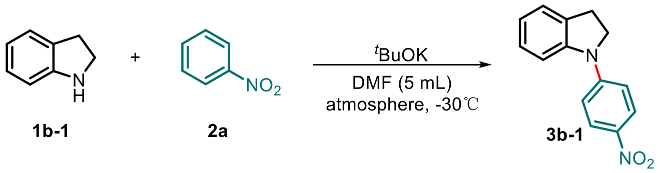

Entry	Atmosphere	Yield (%) [b]
1	Air	31
2	N_2 [c]	Trace
3	O_2	85

[a] Reaction conditions: **1b-1** (59.6 mg, 0.5 mmol), **2a** (184.7 mg, 1.5 mmol, 3.0 equiv.), ^{t}BuOK (224.5 mg, 2.0 mmol, 4.0 equiv.) in DMF (5 mL), stirred for 17 h, -30 °C, O_2 (1.0 atm); [b] Isolated yield. [c] Deoxygenation by lyophilization five times, N_2.

3.3. General procedure C: for synthesis of nitrobenzene C-H amination product **3d-1**

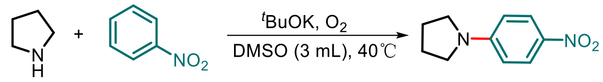

1d-1 **2a** **3d-1**

Pyrrolidine **1d-1** (27.3 mg, 0.3 mmol), ^{t}BuOK (109.8 mg, 0.90 mmol), nitrobenzene (147.6 mg, 1.20 mmol) were added in dry DMSO (3 mL) solvent at room temperature under O_2 (1.0 atm) atmosphere. The reaction mixture was stirred for 6h at 40 ℃. The reaction mixture was cooled to room temperature and then quenched with water (5 mL), diluted with ethyl acetate, and extracted with ethyl acetate (100 mL x 3). The phases were washed with brine (5 mL), dried over Na_2SO_4 and concentrated in vacuo. The residue was purified by silica gel column chromatography (PE: EtOAc = 80:1, v/v). Compound **3d-1** (41.5 mg, 72% yield) was obtained as a white solid and **6g** (7.7 mg, 12% yield) was also obtained. Products **3d-1**, **3d-2** were prepared by this method.

4. Gram-scale synthesis of 3c-10 and denitrative transformations of the NO_2 group

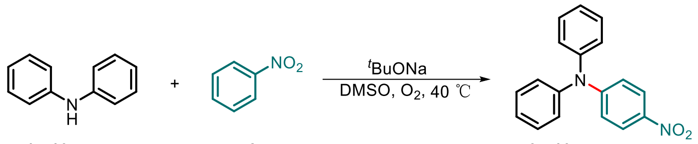

1c-10, 1.20g **2a** **3c-10**, yield: 70%

1,2,3,4-tetrahydroquinoline **1c-10** (1.20 g, 7.1 mmol), nitrobenzene (3.50 g, 4.0 equiv.) and ^{t}BuONa (2.05 g, 3.0 equiv.) were added in dry DMSO (35 mL) solvent at room temperature under O_2 (1.0 atm) atmosphere. The reaction mixture was stirred at 40°C and monitored by TLC. After the complete consumption of **1a-1**, the reaction mixture was cooled to room temperature and then quenched with water (5 mL), diluted with ethyl acetate, and extracted with ethyl acetate (100 mL × 3). The combined organic phases were washed with saturated NaCl aqueous solution. The combined organic phases were washed with brine (5 mL), dried over Na_2SO_4 and concentrated in vacuo. The residue was purified by neutral Al_2O_3 column chromatography (PE: EtOAc = 45:1, v/v). Compound **3c-10** (1.43 g, 70%) was obtained as a yellow solid.

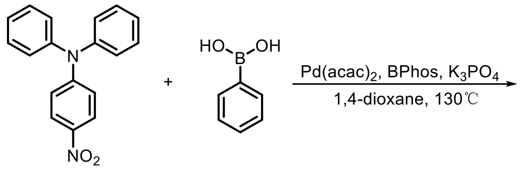

4-nitro-*N*,*N*-diphenylaniline (116.0 mg, 0.40 mmol), BrettPhos (43.0 mg, 0.08 mmol), phenylboronic acid (73.2mg, 0.60 mmol), K_3PO_4 (254.8 mg, 1.2 mmol) were added in dry 1,4-dioxane (1.5 mL) solvent at room temperature under N_2 (1.0 atm) atmosphere. The reaction mixture was stirred for 24 h at 130°C. The reaction mixture was cooled to room temperature and then quenched with water (5 mL), diluted with ethyl acetate, and extracted with ethyl acetate (100 mL x 3). The phases were washed with brine (5 mL), dried over Na_2SO_4 and concentrated in vacuo. The residue was purified by silica gel column chromatography. Compound **3c-10a** (64.8 mg, 55% yield) was obtained as a white solid. [14]

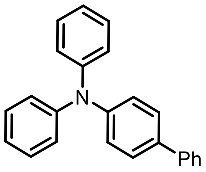

***N,N*-diphenyl-[1,1'-biphenyl]-4-amine (3c-10a)** [14]

Prepared according to above procedure (PE: EtOAc = 50:1, v/v) to give **3c-10a** (64.8 mg, 55% yield) as a white solid. ^{1}H NMR (400 MHz, $CDCl_3$) δ 7.63 (d, J = 8.0 Hz, 2H), 7.53 (d, J = 8.4 Hz, 2H), 7.47 (t, J = 7.6 Hz, 4H), 7.38-7.30 (m, 5H), 7.20 (d, J = 8.4 Hz, 6H), 7.09 (t, J = 7.4 Hz, 2H). ^{13}C NMR (101 MHz, $CDCl_3$) δ 147.6, 147.1, 140.6, 135.1, 129.2, 128.7, 127.7, 126.8, 126.6, 124.4, 123.9, 122.9.

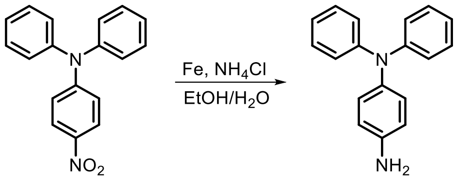

4-nitro-*N,N*-diphenylaniline (116.0 mg, 0.40 mmol), Fe (560.0 mg, 10 mmol), NH_4Cl (530.0 mg, 10 mmol), K_3PO_4 (254.8 mg, 1.2 mmol) were added in EtOH/H_2O = (4/5 mL) solvent at room temperature under N_2 (1.0 atm) atmosphere. The reaction mixture was stirred for 5h at 85 ℃. The reaction mixture was cooled to room temperature and then quenched with water (5 mL), diluted with ethyl acetate, and extracted with ethyl acetate (100 mL x 3). The phases were washed with brine (5 mL), dried over Na_2SO_4 and concentrated in vacuo. The residue was purified by silica gel column chromatography. Compound **3c-10b** (126.6 mg, 97% yield) was obtained as purple oil.[15]

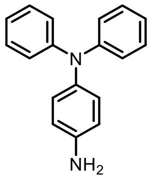

***N^1,N^1*-diphenylbenzene-1,4-diamine (3c-10b)** [15]

Prepared according to above procedure (PE: EtOAc = 1:1, v/v) to give **3c-10b** (126.6 mg, 97% yield) as purple oil. ^{1}H NMR (400 MHz, $CDCl_3$) δ 7.21 (t, J = 7.0 Hz, 4H),

7.06 (d, J = 8.0 Hz, 4H), 7.00-6.91 (m, 4H), 6.65 (d, J = 7.6 Hz, 2H), 3.51(s, 2H). ^{13}C NMR (101 MHz, $CDCl_3$) δ 148.2, 142.9, 138.9, 128.9, 127.8, 122.5, 121.4, 116.1.

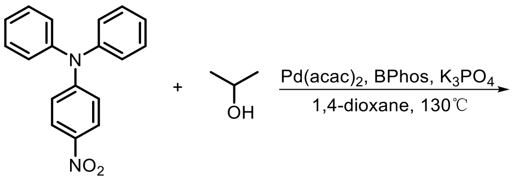

4-nitro-*N,N*-diphenylaniline (116.0 mg, 0.40 mmol), BrettPhos (43.0 mg, 0.08 mmol), isopropanol (36.1 mg, 0.60 mmol), K_3PO_4 (254.8 mg, 1.2 mmol) were added in dry 1,4-dioxane (1.5 mL) solvent at room temperature under N_2 (1.0 atm) atmosphere. The reaction mixture was stirred for 24h at 130 ℃. The reaction mixture was cooled to room temperature and then quenched with water (5 mL), diluted with ethyl acetate, and extracted with ethyl acetate (100 mL x 3). The phases were washed with brine (5 mL), dried over Na_2SO_4 and concentrated in vacuo. The residue was purified by silica gel column chromatography. Compound **3c-10c** (64.8 mg, 55% yield) was obtained as a white solid. [16a]

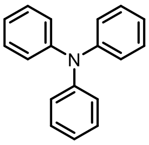

Triphenylamine (3c-10c) [16a]

Prepared according to above procedure (PE: EtOAc = 100:1, v/v) to give **3c-10c** (63.8 mg, 57% yield) as a white solid. ^{1}H NMR (400 MHz, $CDCl_3$) δ 7.34-7.28 (m, 6H), 7.20-7.15 (m, 6H), 7.10-7.05 (m, 3H). ^{13}C NMR (101 MHz, $CDCl_3$) δ 147.8, 129.2, 124.1, 122.6.

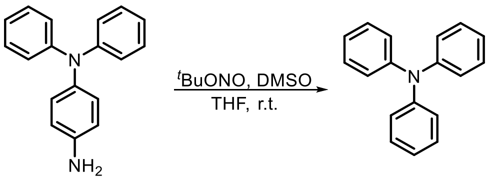

N^1,*N*^{1}-diphenylbenzene-1,4-diamine **3c-10b** (130.0 mg, 0.50 mmol), ^{t}BuONO (73.3 mg, 0.75 mmol), DMSO (3.9mg, 0.05 mmol) were added in THF (3mL) solvent at room temperature under N_2 (1.0 atm) atmosphere. The reaction mixture was stirred for 5h at

35 ℃. The reaction mixture was cooled to room temperature and then quenched with water (5 mL), diluted with ethyl acetate, and extracted with ethyl acetate (100 mL x 3). The phases were washed with brine (5 mL), dried over Na_2SO_4 and concentrated in vacuo. The residue was purified by silica gel column chromatography (PE: EtOAc = 100:1, v/v). Compound **3c-10c** (77.0 mg, 62% yield) was obtained as a white solid.

5. EPR experiments

In order to verify the reaction mechanism of this reaction, we conducted EPR experiments under standard conditions. In the dried reaction tube, 1,2,3,4-tetrahydroquinoline **1a-1** (66.6 mg, 0.5 mmol) and *ᵗ*BuONa (144.2 mg, 1.5 mmol, 3.0 equiv.) were added in a solution of dry DMSO (3.0 mL), stirred at 40 °C under O_2 atmosphere, then the reaction mixture was stirred for 10 min. Remove the solution sample and place it in a small test tube for EPR testing.

When there wasn't **2a**, another competing reaction is that nitrogen radical tended to be easily transformed into aminoxyl radical (g = 2.0054, A_N = 11.5 G) in the DMSO/^{t}BuONa/O_2 system through Korcek's radical-trapping antioxidant (RTA), and was also obvious after reacting for 10 min (Supplementary Fig. 1). Furthermore, the absence of ^{t}BuONa or O_2 resulted in no observation of any nitrogen radical, indicating that both the base and O_2 are essential for the formation of nitrogen radical.

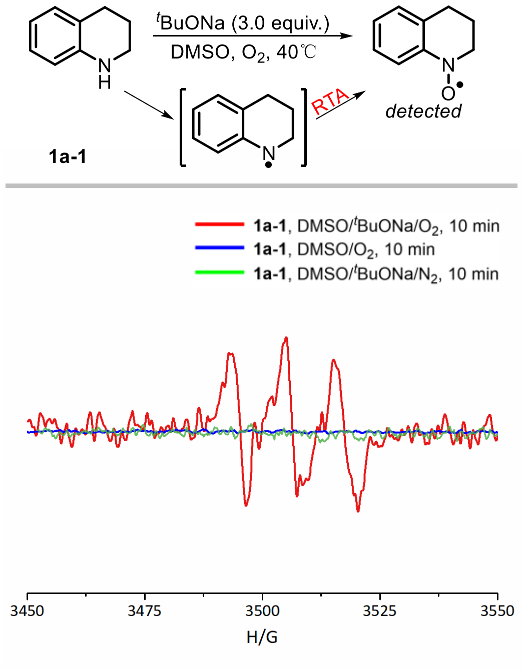

Supplementary Fig. 1 | EPR studies of our reaction design.

6. Radical clock experiments

To further verify the reaction mechanism, a radical clock experiment with *N*-cyclopropylaniline **5g** and **2a** was carried out to determine whether this was a possible radical pathway (Supplementary Fig. 2). In the system, the expected product **6g-1** was not observed, but we could get **6g** in 28% yield (Supplementary Fig. 2a). Then, we considered the possible process may be that **5g** could be transformed into radical **5g-I** by HAT, and radical **5g-I** decomposed into aniline **5d**, which then reacted with **2a** to give the product **6g**. To realize the process, control experiments were carried out. We found that the base and O_2 are all necessary for the formation of **6g** from **5g** and **2a**, which could all remain in the system without base or O_2. As expected, **5d** could be obtained from **5g** in 24% yield, and **5h** was detected by *GC-MS*, demonstrating the occurrence of nitrogen radical. This transformation didn't happen when there's no base or O_2 (Supplementary Fig. 2b). What's more, **6g** couldn't be obtained from *N*-cyclopropyl-4-nitro-*N*-phenylaniline **6h** (Supplementary Fig. 2c). So it wasn't that **5g** firstly reacted with **2a** to give **6h**, which then translated into **5g**, and it was just that **6g** could be directly obtained from **5g** in reaction with **2a**. Meanwhile, we successfully got **6g** from *N*-phenylpropan-1-imine **5h** and **2a** in 32% yield, and also the base and O_2 are all necessary for the formation of **6g** (Supplementary Fig. 2d). Similarly, we could get **5d** from **5h** in 41% yield, and interestingly enough, this transformation also needed for base and O_2 (Supplementary Fig. 2e). To further validate the reaction mechanism, a radical clock experiment was conducted using *N*-cyclopropyl-3-methoxyaniline **5i** and **2a** to confirm the plausibility of a radical-mediated pathway (Supplementary Fig. 2f). The expected product **6i-1** was determined by GC-MS in the system, resulting in the formation of **6i** with a yield of 28%. According to these experiment results, we further came to the conclusion that the reaction may proceed through a radical pathway.

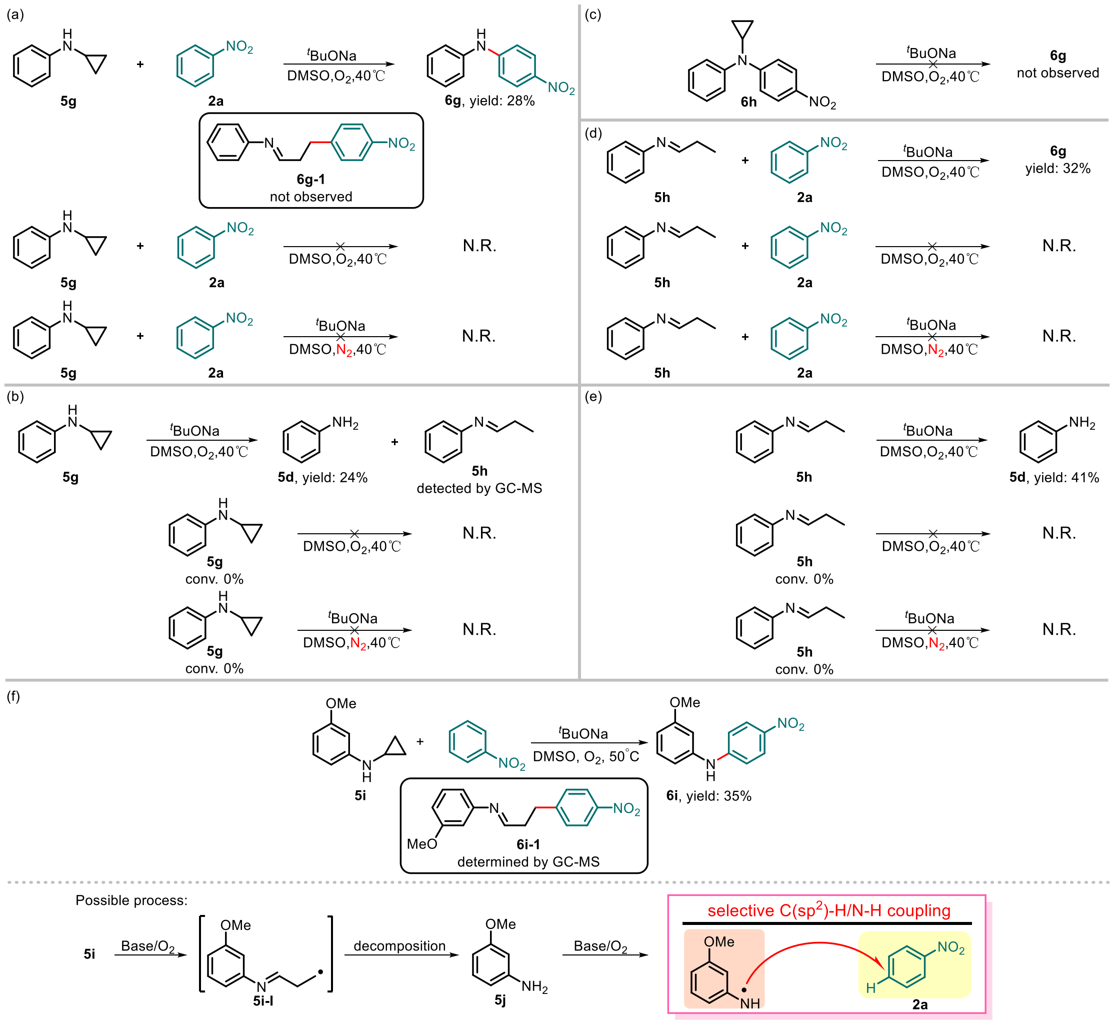

Supplementary Fig. 2 | Radical clock experiments. (**a**) Control experiments of **5g** with **2a**. (**b**) Control experiments of **5g**. (**c**) Control experiments of **6h**. (**d**) Control experiments of **5h** with **2a**. (**e**) Control experiments of **5h**. (**f**) Control experiments of **5i** with **2a**.

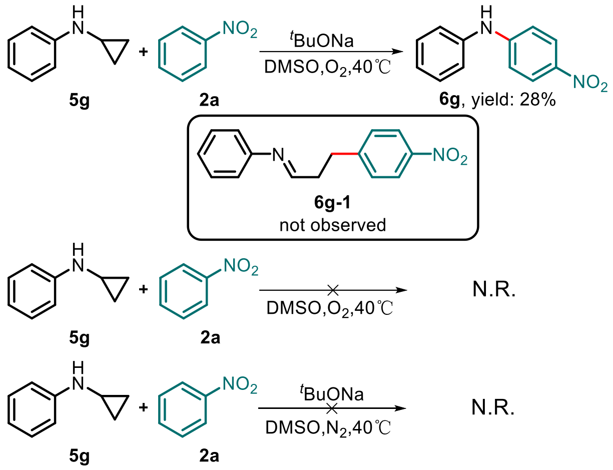

Supplementary Fig. 3 | Control experiments of **5g** with **2a**.

N-cyclopropylaniline **5g** (66.6 mg, 0.5 mmol), nitrobenzene **2a** (246.3 mg, 2.0 mmol, 4.0 equiv.) and ^{t}BuONa (144.2 mg, 1.5 mmol, 3.0 equiv.) were added in dry DMSO (3 mL) at room temperature under O_2 (1.0 atm) atmosphere. The reaction mixture was stirred at 40℃ and monitored by TLC. After complete consumption of **5g**, the reaction mixture was cooled to room temperature and then quenched with water (5 mL), diluted with ethyl acetate, and extracted with ethyl acetate (100 mL × 3). The combined organic phases were washed with saturated NaCl aqueous solution. The combined organic phases were washed with brine (5 mL), dried over Na_2SO_4 and concentrated in vacuo. The residue was purified by neutral Al_2O_3 column chromatography (PE: EtOAc = 40:1, v/v). Compound **6g** (30 mg, 28%) was obtained as a yellow solid. When there is no base in the system or under nitrogen atmosphere, the conversion of **5g** is 0%, and product **6g** is not obtained.

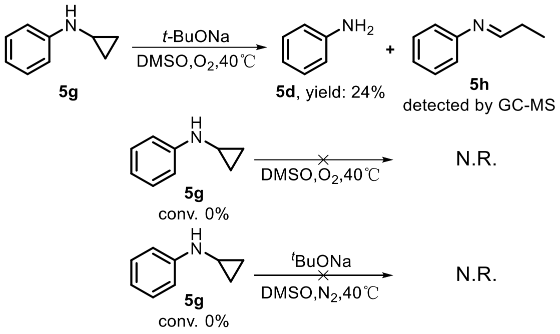

Supplementary Fig. 4 | Control experiments of **5g**.

With above-mentioned operation, in the system, *N*-cyclopropylaniline **5g** (66.6 mg, 0.5 mmol) and ^{t}BuONa (144.2 mg, 1.5 mmol, 3.0 equiv.) were added in dry DMSO (3 mL) solvent at room temperature under O_2 (1.0 atm) atmosphere. The reaction mixture was stirred at 40℃, The residue was purified by neutral Al_2O_3 column chromatography (PE: EtOAc = 40:1, v/v). Compound **5d** (11 mg, 24%) was obtained as a yellow liquid. When there is no base in the system or under nitrogen atmosphere, the conversion of **5g** is 0%, and product **5d** is not obtained.

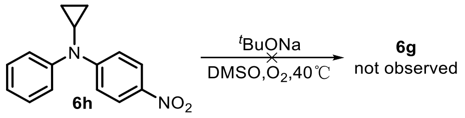

Supplementary Fig. 5 | Control experiments of **6h**.

With above-mentioned operation, in the system, *N*-cyclopropyl-4-nitro-*N*-phenylaniline **8g** (127.1 mg, 0.5 mmol) and *t*-BuONa (144.2 mg, 1.5 mmol, 3.0 equiv.) were added in dry DMSO (3 mL) solvent at room temperature under O_2 (1.0 atm) atmosphere. The reaction mixture was stirred at 40℃, the conversion of **8g** is 0%, and product **9d** was not obtained.

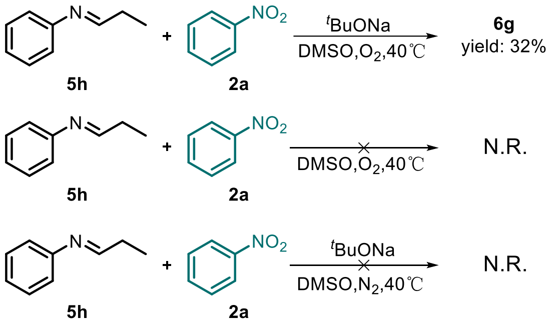

Supplementary Fig. 6 | Control experiments of **5h** with **2a**.

With above-mentioned operation, in the system, *N*-phenylpropan-1-imine **5h** (66.6 mg, 0.5 mmol) nitrobenzene **2a** (246.3 mg, 2.0 mmol, 4.0 equiv.) and *t*-BuONa (144.2 mg, 1.5 mmol, 3.0 equiv.) were added in dry DMSO (3 mL) solvent at room temperature under O_2 (1.0 atm) atmosphere. The reaction mixture was stirred at 40℃, The residue was purified by neutral Al_2O_3 column chromatography (PE: EtOAc = 40:1, v/v). Compound **6g** (34 mg, 32%) was obtained as a yellow liquid. When there is no base in

the system or under nitrogen atmosphere, the conversion of **5h** is 0%, and product **6g** is not obtained.

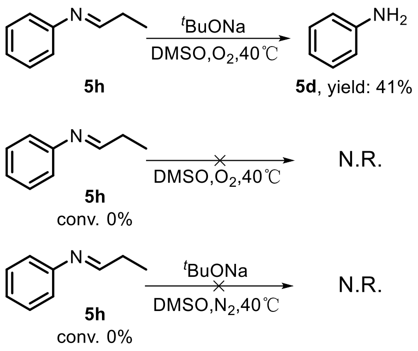

Supplementary Fig. 7 | Control experiments of **5h**.

With above-mentioned operation, in the system, *N*-phenylpropan-1-imine **5h** (66.6 mg, 0.5 mmol) and ᵗBuONa (144.2 mg, 1.5 mmol, 3.0 equiv.) were added in dry DMSO (3 mL) solvent at room temperature under O_2 (1.0 atm) atmosphere. The reaction mixture was stirred at 40 ℃, The residue was purified by neutral Al_2O_3 column chromatography (PE: EtOAc = 40:1, v/v). Compound **5d** (19 mg, 41%) was obtained as a yellow liquid. When there is no base in the system or under nitrogen atmosphere, the conversion of **5h** is 0%, and product **5d** is not obtained.

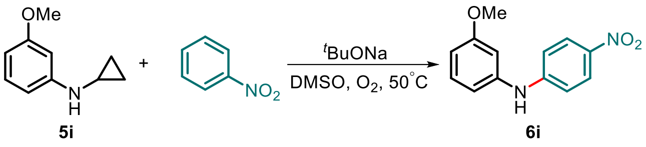

N-cyclopropyl-3-methoxyaniline **5i** (82.0 mg, 0.5 mmol), nitrobenzene **2a** (246.3 mg, 2.0 mmol, 4.0 equiv.) and ᵗBuONa (144.2 mg, 1.5 mmol, 3.0 equiv.) were added in dry DMSO (3 mL) at room temperature under O_2 (1.0 atm) atmosphere. The reaction mixture was stirred at 50℃ and monitored by TLC. After complete consumption of **5i**, the reaction mixture was cooled to room temperature and then quenched with water (5 mL), diluted with ethyl acetate, and extracted with ethyl acetate (100 mL × 3). The combined organic phases were washed with saturated NaCl aqueous solution. The combined organic phases were washed with brine (5 mL), dried over Na_2SO_4 and concentrated in vacuo. The residue was purified by neutral Al_2O_3 column

chromatography (PE: EtOAc = 20:1, v/v). Compound **6i** (42.7 mg, 35% yield) was obtained as a yellow solid.

We did the mass detection of the opened radical clock by GC-MS. After reacting 4.0h, **6i-1**, and **5j** were determined by GC-MS. As the reaction progressed, we found that **6i** increased significantly. **6i** MS (EI) *m/z* (%) 244 (100), 214 (46), 197 (13), 167 (24), 154 (54), 128 (13), 98 (6), 85 (11), 77 (23), 69 (18), 57 (20); **6i-1** MS (EI) *m/z* (%) 284 (93), 267 (72), 253 (42), 237 (30), 222 (21), 207 (73), 191 (24), 179 (17), 167 (25), 147 (100), 134 (42), 121 (60), 107 (57), 91 (60), 77 (91), 57 (51); **5j** MS (EI) *m/z* (%) 123 (50), 93 (15), 77 (100), 65 (16), 51 (64).

(a) 4.0h:

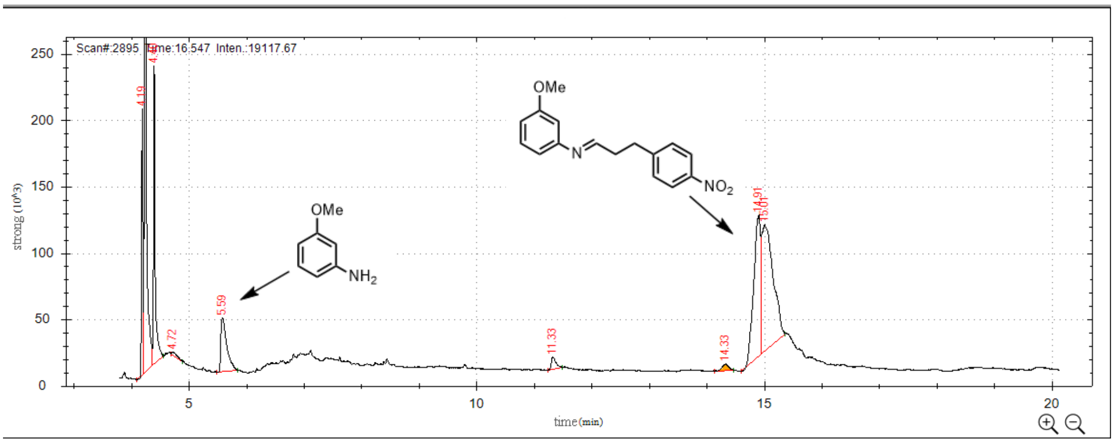

(b) 18.0h:

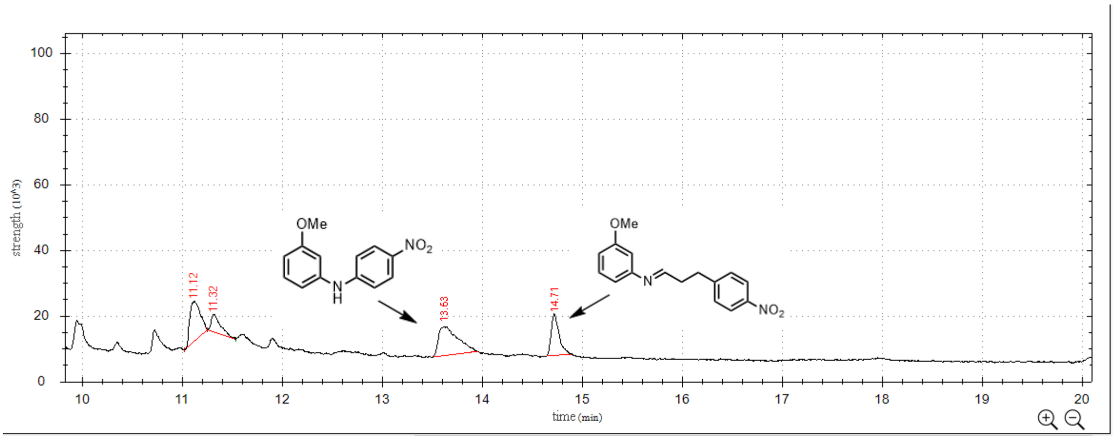

Supplementary Fig. 8 | Mass detection of the opened radical clock by GC-MS. (**a**) 4.0h. (**b**) 18.0h.

7. Theoretical Calculations

1. Computational details

Geometry optimizations and frequency calculations of all structures were performed at PBE0[17]-D3[18] (BJ)[19-21]/6-31G(d) level of theory, the temperature was set to 313.15 K, and Gibbs free energy correction *GFEC* values were obtained. Each transition state (**TS**) structure has only one imaginary frequency (v_i) and the other structures have no imaginary frequency. We also carried out intrinsic reaction coordination (IRC) [22, 23] calculations to ensure that each **TS** structure connected the corresponding reactant and product. Single-point energy *E* values were calculated at PBE0-D3(BJ)/ Def2-TZVP[24]/SMD[25] (DMSO) level of theory to obtain more accurate solvation energies. The Gibbs free energies *G* values were calculated as follows: $G=GFEC+E$.

Geometry optimizations, frequency calculations and IRC calculations were performed using Gaussian 16 C.01 software package [26a], the spin density analyses, Hirshfeld atomic charge calculations[26b], Mayer Bond Order analyses[26c] and bond electron density ρ calculations (by means of QTAIM[26d-26f]) were performed using Multiwfn 3.8 (dev) software [27], and optimized structures were drawn using CYL view software [28].

According to the Arrhenius formula, we determined that the relative rates of **TS1** and **TS1c** are in a ratio of 1:0.38, indicating that the rate of **TS1c** is comparatively slower than that of **TS1**. Consequently, the corresponding yield of **5f** is also lower compared to **2a**. It should be noted that due to limitations in computational precision and cost considerations, our results provide qualitative accuracy rather than quantitative precision.

2. Reaction mechanism

2.1 Reaction mechanism and Gibbs free energy diagram

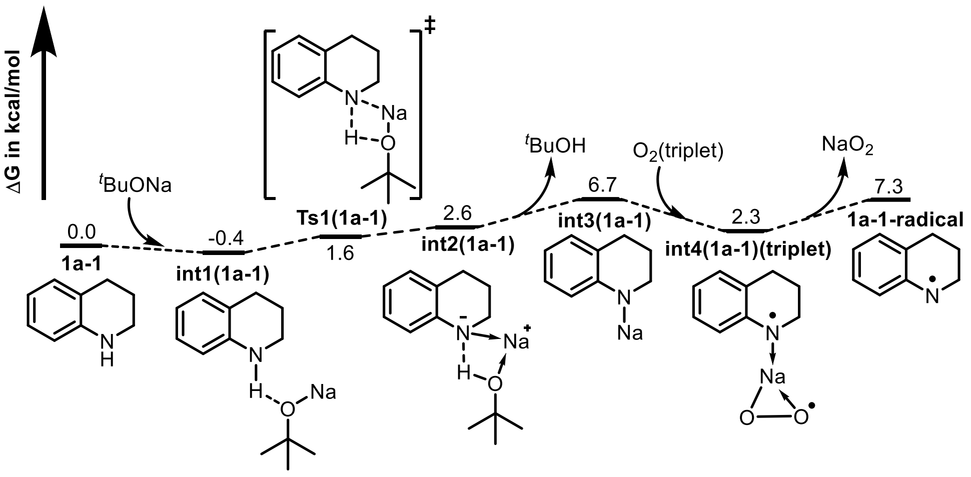

Supplementary Fig. 9 | Gibbs free energy diagram of the formation of N-radicals. The Gibbs free energies of **1a-1**, **PhNH$_2$**, **ᵗBuONa** and **O$_2$(triplet)** were set to 0.0 kcal/mol as references.

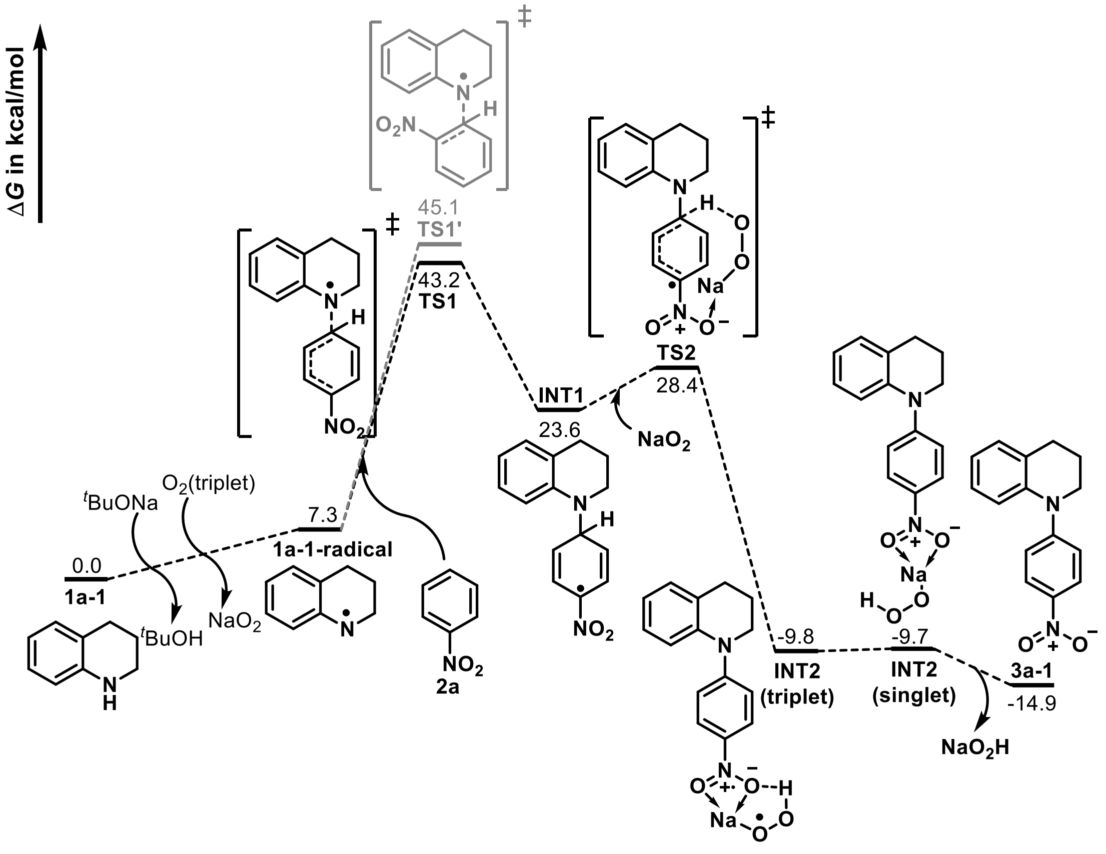

Supplementary Fig. 10 | Gibbs free energy diagram of the reaction between **1a-1** and **2a**. The Gibbs free energies of **1a-1**, **2a**, **ᵗBuONa** and **O$_2$(triplet)** were set to 0.0 kcal/mol as references.

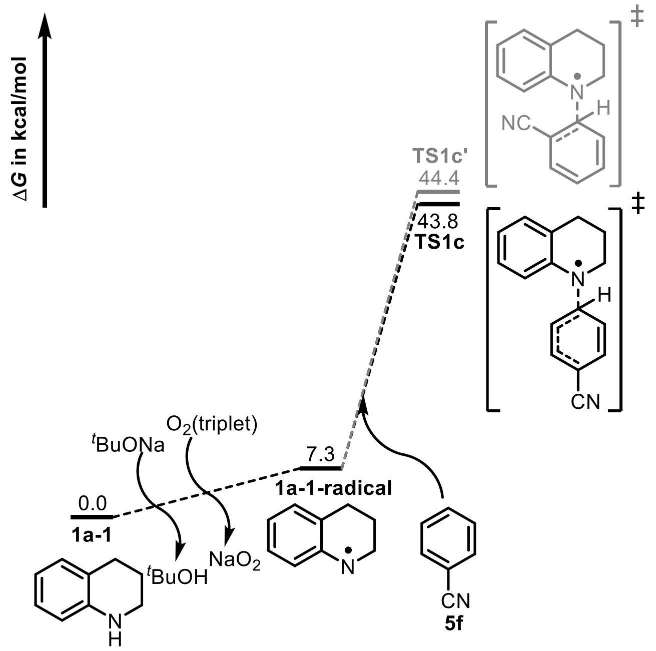

Supplementary Fig. 11 | Gibbs free energy diagram of the reaction between **1a-1** and **PhCN**. The Gibbs free energies of **1a-1**, **PhCN**, **ᵗBuONa** and **O_2(triplet)** were set to 0.0 kcal/mol as references.

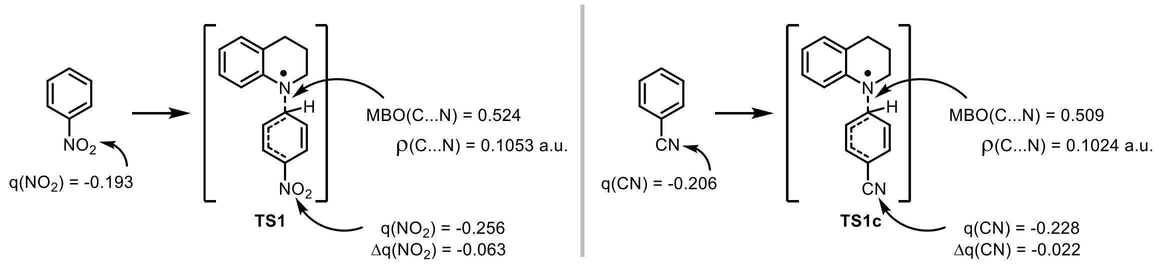

Supplementary Fig. 12 | The results of Hirshfeld charge calculations, Mayer Bond Order (MBO) analyses and QTAIM analyses of **$PhNO_2$**, **TS1**, **PhCN** and **TS1c**.

2.2 Optimized structures and spin density distributions

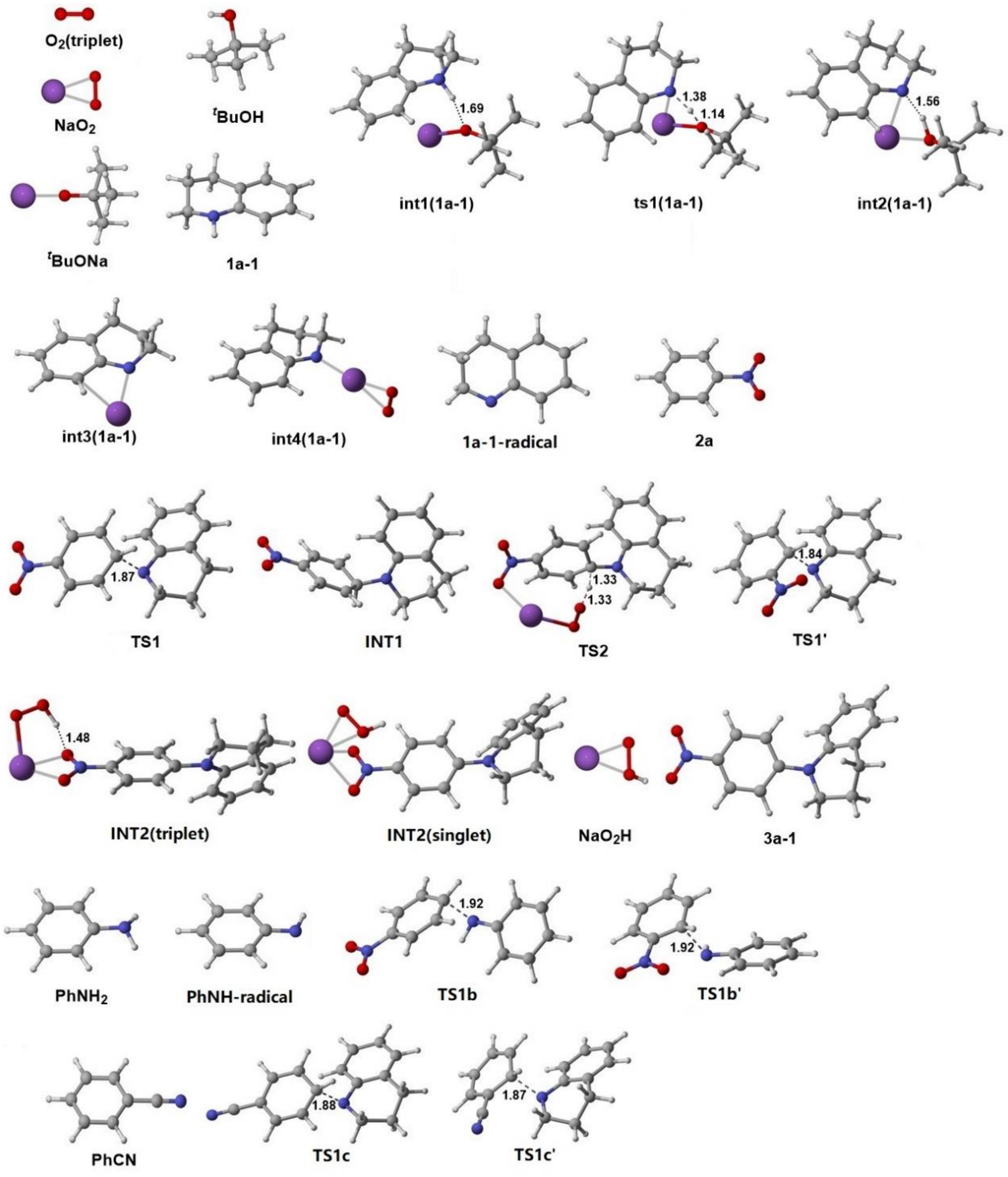

Supplementary Fig. 13 | Optimized structures. Some intermolecular interactions were omitted for clarity. Distances are in Å.

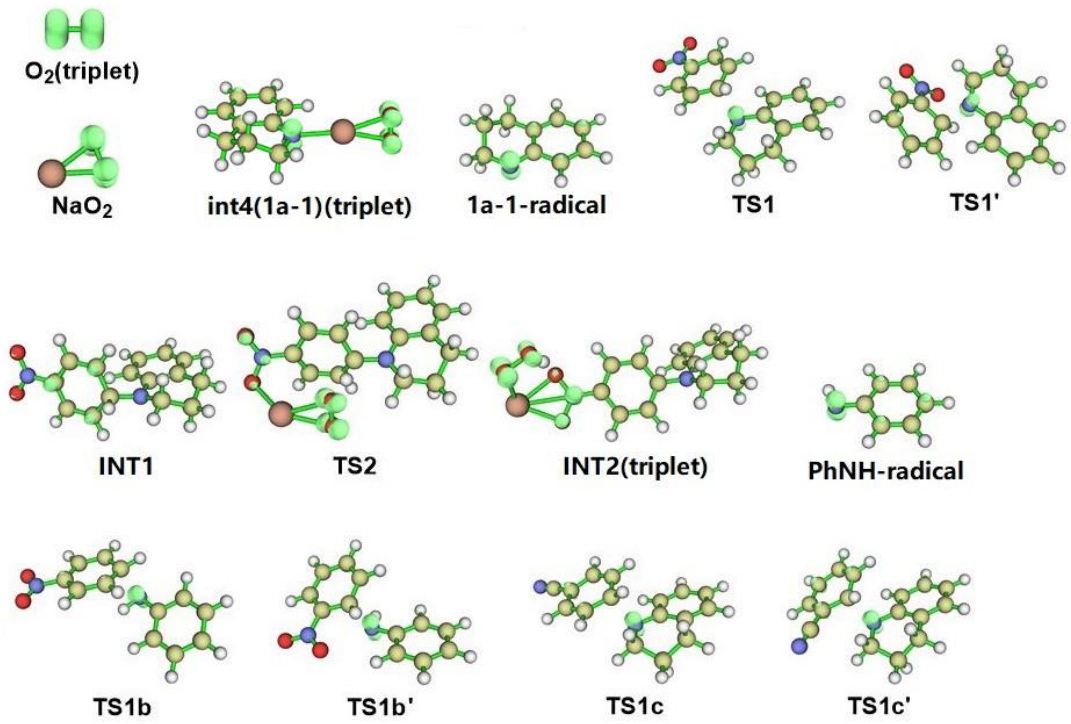

Supplementary Fig. 14 | Spin density distributions of all structures containing unpaired electrons. Green and blue cloud represents for alpha and beta electron distributions, respectively. Isovalues were set as 0.03 a.u..

3. Calculated original data

Supplementary Table 18. Calculated Gibbs free energy correction *GFEC* values (in a.u.), single-point energy *E* values (in a.u.), Gibbs free energy *G* values (in a.u.) of all optimized structures, and imaginary frequencies (denoted as v_is, in cm^{-1}) of all optimized transition state structures.

	GFEC	*E*	*G*	v_i
**O_2(triplet)**	-0.011778	-150.247555	-150.259333	
**1a-1**	0.149958	-404.033867	-403.883909	
**^{t}BuONa**	0.090171	-395.117732	-395.027561	
**^{t}BuOH**	0.106531	-233.501868	-233.395337	
**int1(1a-1)**	0.259223	-799.171367	-798.912144	
**ts1(1a-1)**	0.257030	-799.165885	-798.908855	-372.5
**int2(1a-1)**	0.259406	-799.166795	-798.907389	
**int3(1a-1)**	0.134387	-565.639842	-565.505455	
**int4(1a-1)**	0.126618	-715.897916	-715.771298	
**NaO_2**	-0.022647	-312.489755	-312.512402	
**1a-1-radical**	0.135469	-403.386913	-403.251444	
**2a**	0.070943	-436.452701	-436.381758	
**TS1**	0.228646	-839.804659	-839.576013	-662.6
**TS1'**	0.229300	-839.802174	-839.572874	-600.7
**INT1**	0.230314	-839.837541	-839.607227	
**TS2**	0.224137	-1152.336162	-1152.112025	-1008.9
**INT2(triplet)**	0.228313	-1152.401179	-1152.172866	
**INT2(singlet)**	0.231416	-1152.404134	-1152.172718	
**NaO_2H**	-0.011020	-313.096972	-313.107992	
**3a-1**	0.223804	-839.296765	-839.072961	
**$PhNH_2$**	0.087498	-287.390039	-287.302541	
**PhNH-radical**	0.073483	-286.734862	-286.661379	
**TS1b**	0.165506	-723.167330	-723.001824	-582.4
**TS1b'**	0.166357	-723.168751	-723.002394	-564.9
**PhCN**	0.068120	-324.238480	-324.170360	
**TS1c**	0.225643	-727.589222	-727.363579	-671.9
**TS1c'**	0.225239	-727.587955	-727.362716	-680.1

8. Characterization Data of the Compounds

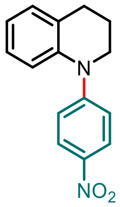

1-(4-nitrophenyl)-1,2,3,4-tetrahydroquinoline (3a-1)

Prepared according to general procedure A (PE: EtOAc = 50:1, v/v) from **1a-1** (66.6 mg) to afford **3a-1** (90.3 mg, 71% yield) as a yellow solid (m.p. 115-117 °C). ^{1}H NMR (400 MHz, $CDCl_3$), δ 8.02 (d, J = 8.4 Hz, 2H), 7.14 (d, J = 8.4 Hz, 1H), 7.09 (d, J = 9.2 Hz, 3H), 7.02 (t, J = 7.4 Hz, 1H), 6.89 (t, J = 7.2 Hz, 1H), 3.62 (t, J = 6.2 Hz, 2H), 2.67 (t, J = 6.0 Hz, 2H), 1.99-1.92 (m, 2H). ^{13}C NMR (101 MHz, $CDCl_3$) δ 153.2, 140.5, 139.8, 131.0, 129.2, 126.4, 125.4, 122.9, 120.5, 117.2, 48.7, 27.1, 24.1. HRMS (m/z): calcd for $C_{15}H_{14}N_2O_2$ $[M+H]^+$: 255.1128, Found: 255.1121.

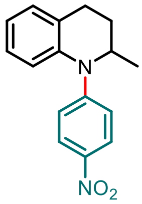

2-methyl-1-(4-nitrophenyl)-1,2,3,4-tetrahydroquinoline (3a-2)

Prepared according to general procedure A (PE: EtOAc = 50:1, v/v) from **1a-2** (73.6 mg) to afford **3a-2** (55.0 mg, 41% yield) as a yellow solid (m.p. 70-72 °C). ^{1}H NMR (400 MHz, $CDCl_3$), δ 8.01 (d, J = 9.2 Hz, 2H), 7.11-6.98 (m, 5H), 6.90 (t, J = 7.2 Hz, 1H), 4.18-4.10 (m, 1H), 2.78-2.60 (m, 2H), 2.20-2.09 (m, 1H), 1.68-1.58 (m, 1H), 1.22 (d, J = 6.8 Hz, 3H). ^{13}C NMR (101 MHz, $CDCl_3$) δ 153.5, 139.9, 139.2, 129.9, 129.3, 126.5, 125.5, 122.9, 121.7, 117.6, 53.6, 29.9, 23.8, 18.6. HRMS (m/z): calcd for $C_{16}H_{16}N_2O_2$ $[M+H]^+$: 269.1285, Found: 269.1274.

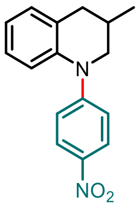

3-methyl-1-(4-nitrophenyl)-1,2,3,4-tetrahydroquinoline (3a-3)

Prepared according to general procedure A (PE: EtOAc = 50:1, v/v) from **1a-3** (73.6 mg) to afford **3a-3** (104.6 mg, 78% yield) as a yellow solid (m.p. 138-140 °C). ^{1}H NMR (400 MHz, $CDCl_3$), δ 8.04 (d, J = 9.2 Hz, 2H), 7.13 (t, J = 7.4 Hz, 3H), 7.07 (d, J = 7.2 Hz, 1H), 7.01 (t, J = 7.6 Hz, 1H), 6.88 (t, J = 7.2 Hz, 1H), 3.78 (dd, J = 11.4, 4.6 Hz, 1H), 3.11 (t, J = 10.6 Hz, 1H), 2.78 (dd, J = 15.6, 4.8 Hz, 1H), 2.38 (dd, J = 15.6, 10.4 Hz, 1H), 2.11-2.01 (m, 1H), 1.02 (d, J = 6.4 Hz, 3H). ^{13}C NMR (101 MHz, $CDCl_3$) δ 153.3, 140.2, 139.9, 129.5(0), 129.4(6), 126.3, 125.3, 122.6, 119.8, 117.8, 55.8, 35.4, 29.7, 19.0. HRMS (m/z): calcd for $C_{16}H_{16}N_2O_2$ $[M+H]^+$: 269.1285, Found: 269.1274.

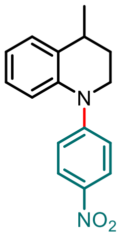

4-methyl-1-(4-nitrophenyl)-1,2,3,4-tetrahydroquinoline (3a-4)

Prepared according to general procedure A (PE: EtOAc = 50:1, v/v) from **1a-4** (73.6 mg) to afford **3a-4** (122.1 mg, 81% yield) as a yellow solid (m.p. 70-72 °C). ^{1}H NMR (400 MHz, $CDCl_3$) δ 7.98 (d, J = 8.8 Hz, 2H), 7.13 (t, J = 6.6 Hz, 2H), 7.06-6.98 (m, 3H), 6.92 (t, J = 7.2 Hz, 1H), 3.70-3.53 (m, 1H), 2.79-2.73 (m, 1H), 2.08-2.01 (m, 1H), 1.65-1.55 (m, 1H), 1.22 (d, J = 6.8 Hz, 3H). ^{13}C NMR (101 MHz, $CDCl_3$) δ 153.2, 139.8, 139.7, 136.0, 127.1, 126.4, 125.4, 123.1, 120.8, 116.8, 46.9, 31.9, 30.8, 19.6. HRMS (m/z): calcd for $C_{16}H_{16}N_2O_2$ $[M+H]^+$: 269.1285, Found: 269.1278.

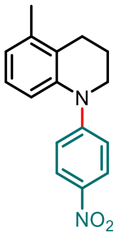

5-methyl-1-(4-nitrophenyl)-1,2,3,4-tetrahydroquinoline (3a-5)

Prepared according to general procedure A (PE: EtOAc = 50:1, v/v) from **1a-5** (73.6 mg) to afford **3a-5** (90.0 mg, 67% yield) as a yellow solid (m.p. 113-115 °C). ^{1}H NMR (400 MHz, $CDCl_3$). δ 7.95 (d, J = 8.8 Hz, 2H), 7.02 (d, J = 8.8 Hz, 2H), 6.93 (d, J = 8.0Hz, 1H), 6.88 (t, J = 7.6 Hz, 1H), 6.76 (d, J = 7.2 Hz, 1H), 3.59 (t, J = 5.8 Hz, 2H), 2.58 (t, J = 6.4 Hz, 2H), 2.16 (s, 3H), 1.94-1.87 (m, 2H). ^{13}C NMR (101 MHz, $CDCl_3$) δ 153.6, 140.8, 139.7, 137.1, 128.6, 125.6, 125.2, 124.5, 118.6, 117.8, 48.5, 24.0, 23.4, 19.4. HRMS (m/z): calcd for $C_{16}H_{16}N_2O_2$ $[M+H]^+$: 269.1285, Found: 269.1275.

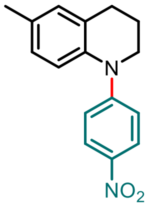

6-methyl-1-(4-nitrophenyl)-1,2,3,4-tetrahydroquinoline (3a-6)

Prepared according to general procedure A (PE: EtOAc = 50:1, v/v) from **1a-6** (73.6 mg) to afford **3a-6** (88.5 mg, 66% yield) as a yellow solid (m.p. 86-88 °C). ^{1}H NMR (400 MHz, $CDCl_3$) δ 8.02 (d, J = 9.2 Hz, 2H), 7.07-7.04 (m, 3H), 6.91 (s, 1H), 6.86 (d, J = 8.4 Hz, 1H), 3.61(t, J = 6.2 Hz, 2H), 2.63 (t, J = 6.2 Hz, 2H), 2.23 (s, 3H), 1.98-1.90 (m, 2H). ^{13}C NMR (101 MHz, $CDCl_3$) δ 153.3, 139.4, 137.8, 132.7, 131.2, 129.6, 127.1, 125.4, 120.8, 116.5, 48.6, 27.1, 24.2, 20.7. HRMS(m/z): calcd for $C_{16}H_{16}N_2O_2$ $[M+H]^+$: 269.1285, Found: 269.1274.

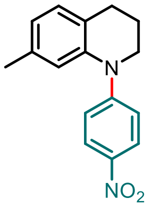

7-methyl-1-(4-nitrophenyl)-1,2,3,4-tetrahydroquinoline (3a-7)

Prepared according to general procedure A (PE: EtOAc = 50:1, v/v) from **1a-7** (73.6 mg) to afford **3a-7** (108.7 mg, 81% yield) as a yellow solid (m.p. 95-97 °C). ^{1}H NMR (400 MHz, $CDCl_3$) δ 7.97 (d, J = 9.2 Hz, 2H), 7.05 (d, J = 9.2 Hz, 2H), 6.93 (d, J = 4.8 Hz, 2H), 6.68 (d, J = 7.6 Hz, 1H), 3.57 (t, J = 6.0 Hz, 2H), 2.60 (t, J = 5.8 Hz, 2H), 2.14 (s, 1H), 1.93-1.86 (m, 2H). ^{13}C NMR (101 MHz, $CDCl_3$) δ 153.2, 140.2, 139.6, 136.0, 128.9, 127.8, 125.3, 123.7, 120.8, 117.1, 48.6, 26.6, 24.0, 21.1. HRMS (m/z): calcd for $C_{16}H_{16}N_2O_2$ $[M+H]^+$: 269.1285, Found: 269.1273.

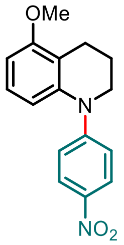

5-methoxy-1-(4-nitrophenyl)-1,2,3,4-tetrahydroquinoline (3a-8)

Prepared according to general procedure A (PE: EtOAc = 50:1, v/v) from **1a-8** (81.6 mg) to afford **3a-8** (126.5 mg, 89% yield) as a yellow solid (m.p. 121-123 °C). ^{1}H NMR (400 MHz, $CDCl_3$) δ 8.01 (d, J = 8.8 Hz, 2H), 7.08 (d, J = 8.8 Hz, 2H), 6.95 (t, J = 8.2 Hz, 1H), 6.70 (d, J = 8.0 Hz, 1H), 6.45 (d, J = 8.4 Hz, 1H), 3.76 (s, 3H), 3.61 (t, J = 5.4 Hz, 2H), 2.64 (t, J = 6.6 Hz, 2H), 1.92-1.85 (m, 2H). ^{13}C NMR (101 MHz, $CDCl_3$) δ 157.7, 153.5, 141.7, 140.0, 126.1, 125.1, 118.6, 118.0, 112.8, 104.0, 55.3, 48.9, 22.7, 20.6. HRMS (m/z): calcd for $C_{16}H_{16}N_2O_3$ $[M+H]^+$: 285.1234, Found: 285.1226.

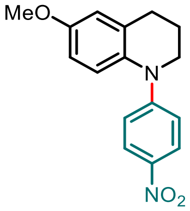

6-methoxy-1-(4-nitrophenyl)-1,2,3,4-tetrahydroquinoline (3a-9)

Prepared according to general procedure A (PE: EtOAc = 50:1, v/v) from **1a-9** (81.6 mg) to afford **3a-9** (89.6 mg, 63% yield) as a yellow solid (m.p. 154-156 °C). ^{1}H NMR

(400 MHz, $CDCl_3$) δ 8.01 (d, J = 9.2 Hz, 2H), 7.10 (d, J = 8.4 Hz, 1H), 7.00 (d, J = 9.2 Hz, 2H), 6.66-6.60 (m, 2H), 3.73 (s, 3H), 3.61 (t, J = 6.4 Hz, 2H), 2.64 (t, J = 6.2 Hz, 2H), 1.97-1.90 (m, 2H). ^{13}C NMR (101 MHz, $CDCl_3$) δ 155.6, 153.4, 139.0, 133.4, 129.2, 125.5, 122.6, 115.7, 113.8, 112.1, 55.4, 48.3, 27.3, 24.2. HRMS (m/z): calcd for $C_{16}H_{16}N_2O_3$ $[M+H]^+$: 285.1234, Found: 285.1233.

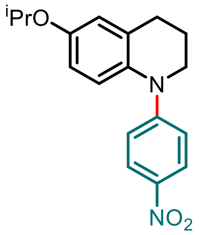

6-isopropoxy-1-(4-nitrophenyl)-1,2,3,4-tetrahydroquinoline (3a-10)

Prepared according to general procedure A (PE: EtOAc = 50:1, v/v) from **1a-10** (95.6 mg) to afford **3a-10** (107.7 mg, 69% yield) as a yellow solid (m.p. 97-99 °C). ^{1}H NMR (400 MHz, $CDCl_3$) δ 8.09 (d, J = 9.2 Hz, 2H), 7.15 (d, J = 8.8 Hz, 1H), 7.08 (d, J = 9.2 Hz, 2H), 6.73-6.67 (m, 2H), 4.54-4.47 (m, 2H), 3.68 (t, J = 6.2 Hz, 2H), 2.70 (t, J = 6.2 Hz, 2H), 2.04-1.97 (m, 2H), 1.35 (d, J = 6.0 Hz, 6H). ^{13}C NMR (101 MHz, $CDCl_3$) δ 154.0, 153.5, 139.2, 133.4, 133.3, 125.6, 122.6, 116.1, 115.9, 114.1, 70.3, 48.4, 27.4, 24.2, 22.1. HRMS (ESI-Orbitrap MS) m/z: Calcd. for $C_{18}H_{20}N_2O_3$ $[M+H]^+$: 313.15467; Found: 313.15372.

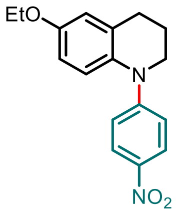

6-ethoxy-1-(4-nitrophenyl)-1,2,3,4-tetrahydroquinoline (3a-11)

Prepared according to general procedure A (PE: EtOAc = 50:1, v/v) from **1a-11** (88.6 mg) to afford **3a-11** (96.9 mg, 65% yield) as a yellow solid (m.p. 71-73 °C). ^{1}H NMR (400 MHz, $CDCl_3$) δ 8.02 (d, J = 9.6 Hz, 2H), 7.09 (d, J = 8.8 Hz, 1H), 7.01 (d, J = 9.6 Hz, 2H), 6.65-6.61 (m, 2H), 3.95 (q, J = 7.2 Hz, 2H), 3.61 (t, J = 6.4 Hz, 2H), 2.63 (t, J = 6.4 Hz, 2H), 1.97-1.90 (m, 2H), 1.35 (t, J = 7.0 Hz, 3H). ^{13}C NMR (101 MHz,

$CDCl_3$) δ 155.1, 153.6, 139.2, 133.4, 133.3, 125.6, 122.6, 115.8, 114.5, 112.8, 63.7, 48.4, 27.4, 24.2, 14.9. HRMS (ESI-Orbitrap MS) m/z: Calcd. for $C_{17}H_{18}N_2O_3$ $[M+H]^+$: 299.13902; Found: 299.13834.

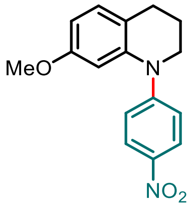

7-methoxy-1-(4-nitrophenyl)-1,2,3,4-tetrahydroquinoline (3a-12)

Prepared according to general procedure A (PE: EtOAc = 50:1, v/v) from **1a-12** (81.6 mg) to afford **3a-12** (133.6 mg, 94% yield) as a yellow solid (m.p. 75-77 °C) ^1H NMR (400 MHz, $CDCl_3$) δ 8.03 (d, J = 9.2 Hz, 2H), 7.13 (d, J = 9.2 Hz, 2H), 6.98 (d, J = 8.4 Hz, 1H), 6.66 (d, J = 2.0 Hz, 1H), 6.47 (dd, J = 8.2, 2.2 Hz, 1H), 3.63-3.58 (m, 5H), 2.63 (t, J = 6.2 Hz, 2H), 1.97-1.89 (m, 2H). ^{13}C NMR (101 MHz, $CDCl_3$) δ 158.1, 153.2, 141.3, 140.1, 129.8, 125.3, 122.6, 117.9, 108.5, 105.7, 55.2, 48.9, 26.3, 23.9. HRMS (m/z): calcd for $C_{16}H_{16}N_2O_3$ $[M+H]^+$: 285.1234, Found: 285.1221.

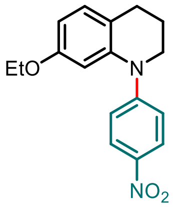

7-ethoxy-1-(4-nitrophenyl)-1,2,3,4-tetrahydroquinoline (3a-13)

Prepared according to general procedure A (PE: EtOAc = 50:1, v/v) from **1a-13** (88.6 mg) to afford **3a-13** (126.8 mg, 85% yield) as a yellow solid (m.p. 51-53 °C). ^1H NMR (400 MHz, $CDCl_3$) δ 7.94 (d, J = 9.2 Hz, 2H), 7.07 (d, J = 8.8 Hz, 2H), 6.90 (d, J = 8.4 Hz, 1H), 6.61 (s, 1H), 6.41 (dd, J = 8.4, 2.4Hz, 1H), 3.78 (q, J = 6.8 Hz, 2H), 3.54 (t, J = 5.8 Hz, 2H), 2.56 (t, J = 6.2 Hz, 2H), 1.90-1.83 (m, 2H), 1.22 (t, J = 7.0 Hz, 3H). ^{13}C NMR (101 MHz, $CDCl_3$) δ 157.3, 153.0, 141.1, 139.8, 129.6, 125.1, 122.4, 117.7, 109.1, 106.1, 63.2, 48.7, 26.2, 23.8, 14.6. HRMS (m/z): calcd for $C_{17}H_{18}N_2O_3$ $[M+H]^+$: 299.1390, Found: 299.1378.

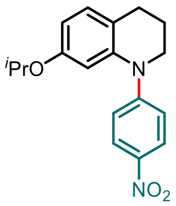

7-isopropoxy-1-(4-nitrophenyl)-1,2,3,4-tetrahydroquinoline (3a-14)

Prepared according to general procedure A (PE: EtOAc = 50:1, v/v) from **1a-14** (95.6 mg) to afford **3a-14** (132.6 mg, 85% yield) as a yellow solid (m.p. 71-73 °C). ^{1}H NMR (400 MHz, $CDCl_3$) δ 8.05 (d, J = 9.2 Hz, 2H), 7.14 (d, J = 9.6 Hz, 2H), 6.97 (d, J = 8.4 Hz, 1H), 6.65 (d, J = 1.6 Hz, 1H), 6.47 (dd, J = 8.2, 2.2 Hz, 1H), 4.36-4.29 (m, 1H), 3.62 (t, J = 6.2 Hz, 3H), 2.63 (t, J = 6.4 Hz, 2H), 1.98-1.90 (m, 2H), 1.21 (d, J = 6.0 Hz, 6H). ^{13}C NMR (101 MHz, $CDCl_3$) δ 156.4, 153.3, 141.4, 140.1, 129.9, 125.4, 122.5, 118.0, 110.9, 107.8, 70.0, 49.0, 26.4, 24.0, 22.0. HRMS (m/z): calcd for $C_{18}H_{20}N_2O_3$ $[M+H]^+$: 313.1547, Found: 313.1546.

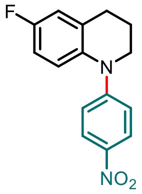

6-fluoro-1-(4-nitrophenyl)-1,2,3,4-tetrahydroquinoline (3a-15)

Prepared according to general procedure A (PE: EtOAc = 50:1, v/v) from **1a-15** (75.6 mg) to afford **3a-15** (96.6 mg, 71% yield) as a yellow solid (m.p. 128-130 °C). ^{1}H NMR (400 MHz, $CDCl_3$) δ 8.00 (d, J = 4.8 Hz, 2H), 7.09 (dd, J = 8.6, 5.0 Hz, 1H), 7.03 (d, J = 8.8 Hz, 2H), 6.79 (d, J = 8.4 Hz, 1H), 6.73 (t, J = 8.2 Hz, 1H), 3.61 (t, J = 6.2 Hz, 2H), 2.65 (t, J = 6.0 Hz, 2H), 1.98-1.90 (m, 2H). ^{13}C NMR (101 MHz, $CDCl_3$) δ 158.4 (d, J = 968.0 Hz), 153.3, 139.7, 136.5 (d, J = 8.0 Hz), 133.3 (d, J = 32.0 Hz), 125.4, 122.3 (d, J = 32.0 Hz), 116.7, 115.4 (d, J = 88.0 Hz), 113.3 (d, J = 92.0 Hz), 48.5, 27.2, 23.8. ^{19}F NMR (376 MHz, $CDCl_3$) δ -119.6 (s, 1F). HRMS (m/z): calcd for $C_{15}H_{13}FN_2O_2$ $[M+H]^+$: 273.1034, Found: 273.1033.

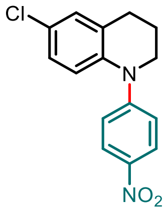

6-chloro-1-(4-nitrophenyl)-1,2,3,4-tetrahydroquinoline (3a-16)

Prepared according to general procedure A (PE: EtOAc = 50:1, v/v) from **1a-16** (81.6 mg) to afford **3a-16** (101.0 mg, 70% yield) as a yellow solid (m.p. 106-108 °C). ^{1}H NMR (400 MHz, $CDCl_3$) δ 8.01 (d, J = 8.8 Hz, 2H), 7.10-7.02 (m, 4H), 6.96 (d, J = 8.8 Hz, 1H), 3.61 (t, J = 6.2 Hz, 2H), 2.66 (t, J = 6.4 Hz, 2H), 1.98-1.91 (m, 2H). ^{13}C NMR (101 MHz, $CDCl_3$) δ 153.0, 140.3, 139.3, 132.2, 128.9, 127.2, 126.4, 125.4, 121.4, 117.7, 48.8, 27.0, 23.6. HRMS (m/z): calcd for $C_{15}H_{13}ClN_2O_2$ $[M+H]^+$: 289.0738, Found: 289.0731.

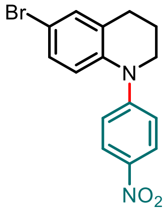

6-bromo-1-(4-nitrophenyl)-1,2,3,4-tetrahydroquinoline (3a-17)

Prepared according to general procedure A (PE: EtOAc = 50:1, v/v) from **1a-17** (105.5 mg) to afford **3a-17** (101.6 mg, 61% yield) as a yellow solid (m.p. 110-112 °C). ^{1}H NMR (400 MHz, $CDCl_3$) δ 8.02 (d, J = 9.6 Hz, 2H), 7.20-7.17 (m, 1H), 7.12-7.07 (m, 3H), 6.99 (d, J = 8.8 Hz, 1H), 3.61 (t, J = 6.2 Hz, 2H), 2.66 (t, J = 6.2 Hz, 2H), 2.00-1.90 (m, 2H). ^{13}C NMR (101 MHz, $CDCl_3$) δ 152.9, 140.4, 139.9, 132.5, 131.8, 129.3, 125.4, 121.7, 117.9, 114.8, 48.8, 27.0, 23.6. HRMS (m/z): calcd for $C_{15}H_{13}BrN_2O_2$ $[M+H]^+$: 333.0233, Found: 333.0228.

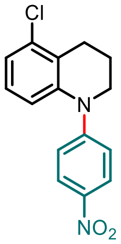

5-chloro-1-(4-nitrophenyl)-1,2,3,4-tetrahydroquinoline (3a-18)

Prepared according to general procedure A (PE: EtOAc = 50:1, v/v) from **1a-18** (81.6 mg) to afford **3a-17** (82.2 mg, 57% yield) as a yellow solid (m.p. 153-155 °C). ^{1}H NMR (400 MHz, $CDCl_3$) δ 8.06 (t, J = 9.2 Hz, 2H), 7.12 (d, J = 9.2 Hz, 2H), 7.01-6.91 (m, 3H), 3.64 (t, J = 5.8 Hz, 2H), 2.80 (t, J = 6.6 Hz, 2H), 2.00-1.93 (m, 2H). ^{13}C NMR (101 MHz, $CDCl_3$) δ 153.3, 142.8, 140.8, 134.8, 127.2, 126.7, 125.4, 123.0, 119.3, 118.5, 49.0, 24.9, 22.9. HRMS (m/z): calcd for $C_{15}H_{13}ClN_2O_2$ [M+H]$^+$: 289.0738, Found: 289.0746.

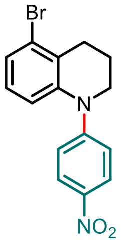

5-bromo-1-(4-nitrophenyl)-1,2,3,4-tetrahydroquinoline (3a-19)

Prepared according to general procedure A (PE: EtOAc = 50:1, v/v) from **1a-19** (105.5 mg) to afford **3a-19** (91.6 mg, 55% yield) as a yellow solid (m.p. 151-153 °C). ^{1}H NMR (400 MHz, $CDCl_3$) δ 8.06 (d, J = 9.2 Hz, 2H), 7.16 (d, J = 8.0 Hz, 1H), 7.11 (d, J = 9.2 Hz, 2H), 7.04 (d, J = 8.0 Hz, 1H), 6.87 (t, J = 8.0 Hz, 1H), 3.64 (t, J = 6.0 Hz, 2H), 2.78 (t, J = 6.8 Hz, 2H), 2.00-1.92 (m, 2H). ^{13}C NMR (101 MHz, $CDCl_3$) δ 153.3, 142.9, 140.8, 128.9, 127.2, 126.4, 125.6, 125.4, 119.3, 119.2, 49.1, 27.9, 23.3. HRMS (m/z): calcd for $C_{15}H_{13}BrN_2O_2$ [M+H]$^+$: 333.0233, Found: 333.0230.

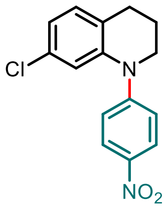

7-chloro-1-(4-nitrophenyl)-1,2,3,4-tetrahydroquinoline (3a-20)

Prepared according to general procedure A (PE: EtOAc = 50:1, v/v) from **1a-20** (81.6 mg) to afford **3a-20** (105.3 mg, 73% yield) as a yellow solid (m.p. 123-125 °C). ^{1}H NMR (400 MHz, $CDCl_3$) δ 8.06 (d, J = 9.2 Hz, 2H), 7.14 (d, J = 8.8 Hz, 2H), 7.07 (s,

1H), 6.99 (d, J = 8.0 Hz, 1H), 6.82 (d, J = 7.2 Hz, 1H), 3.61 (t, J = 6.2 Hz, 2H), 2.67 (t, J = 6.4 Hz, 2H), 1.99-1.92 (m, 2H). ^{13}C NMR (101 MHz, $CDCl_3$) δ 152.8, 142.0, 140.9, 131.8, 130.3, 128.1, 125.5, 122.2, 119.3, 118.7, 48.9, 26.7, 23.5. HRMS (m/z): calcd for $C_{15}H_{13}ClN_2O_2$ $[M+H]^+$: 289.0738, Found: 289.0733.

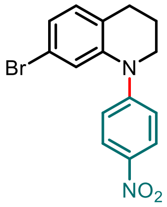

7-bromo-1-(4-nitrophenyl)-1,2,3,4-tetrahydroquinoline (3a-21)

Prepared according to general procedure A (PE: EtOAc = 50:1, v/v) from **1a-21** (105.5 mg) to afford **3a-21** (108.2 mg, 65% yield) as a yellow solid (m.p. 139-141 °C). ^{1}H NMR (400 MHz, $CDCl_3$) δ 8.06 (d, J = 9.2 Hz, 2H), 7.22 (s, 1H), 7.14 (d, J = 9.2 Hz, 2H), 6.99-6.91 (m, 2H), 3.61 (t, J = 6.2 Hz, 1H), 2.65 (t, J = 6.4 Hz, 2H), 1.99-1.92 (m, 2H). ^{13}C NMR (101 MHz, $CDCl_3$) δ 152.7, 142.2, 140.9, 130.6, 128.7, 125.5, 125.1, 122.2, 119.5, 118.6, 48.9, 26.8, 23.5. HRMS (m/z): calcd for $C_{15}H_{13}BrN_2O_2$ $[M+H]^+$: 333.0233, Found: 333.0228.

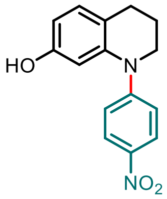

1-(4-nitrophenyl)-1,2,3,4-tetrahydroquinolin-7-ol (3a-22)

Prepared according to general procedure A (PE: EtOAc = 2:1, v/v) from **1a-22** (74.6 mg) to afford **3a-22** (66.0 mg, 49% yield) as a yellow oily liquid. ^{1}H NMR (400 MHz, $CDCl_3$) δ 8.12 (d, J = 8.4 Hz, 2H), 7.22 (d, J = 8.8 Hz, 2H), 7.01 (d, J = 8.0 Hz, 1H), 6.69 (d, J = 2.4 Hz, 1H), 6.47 (dd, J = 8.4, 2.4 Hz, 1H), 4.80 (s, 1H), 3.68 (t, J = 6.2 Hz, 2H), 2.71 (t, J = 6.2 Hz, 2H), 2.05 - 1.98 (m, 2H). ^{13}C NMR (101 MHz, $CDCl_3$) δ 154.2, 153.3, 141.6, 140.3, 130.1, 125.4, 122.4, 118.4, 110.1, 106.7, 49.0, 26.4, 23.9. HRMS (ESI-Orbitrap MS) m/z: Calcd. for $C_{15}H_{14}N_2O_3$ $[M+H]^+$: 271.1083; Found: 271.1079.

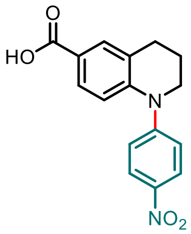

1-(4-nitrophenyl)-1,2,3,4-tetrahydroquinoline-6-carboxylic acid (3a-23)

Prepared according to general procedure A (PE: EtOAc = 2:1, v/v) from **1a-23** (88.6 mg) to afford **3a-23** (111.0 mg, 75% yield) as a yellow solid (m.p. 241-243℃). ^{1}H NMR (400 MHz, DMSO-d^6) δ 12.6 (s, 1H), 8.17 (d, J = 9.2 Hz, 2H), 7.75 (s, 1H), 7.64 (d, J = 8.8 Hz, 1H), 7.42 (d, J = 9.2 Hz, 2H), 7.15 (d, J = 8.8 Hz, 1H), 3.75 (t, J = 6.0 Hz, 2H), 2.81 (t, J = 6.4 Hz, 2H), 2.02 - 1.94 (m, 2H). ^{13}C NMR (101 MHz, DMSO-d^6) δ 167.5, 152.9, 145.4, 141.4, 132.2, 128.9, 128.4, 125.7, 123.6, 120.9, 118.4, 49.6, 27.1, 23.3. HRMS (ESI-Orbitrap MS) m/z: Calcd. for $C_{16}H_{14}N_2O_4$ [M+H]$^+$: 299.1032; Found: 299.1025.

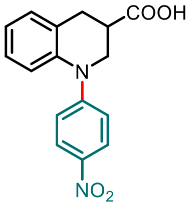

1-(4-nitrophenyl)-1,2,3,4-tetrahydroquinoline-3-carboxylic acid (3a-24)

Prepared according to general procedure A (PE: EtOAc = 20:1, v/v) from **1a-24** (88.6 mg) to afford **3a-24** (104.0 mg, 70% yield) as a yellow solid (m.p. 149-151℃). ^{1}H NMR (400 MHz, $CDCl_3$) δ 8.13 (d, J = 9.2 Hz, 2H), 7.22 - 7.18 (m, 3H), 7.17 - 7.09 (m, 2H), 7.00 (t, J = 7.2 Hz, 1H), 4.04 - 3.98 (m, 1H), 3.90 - 3.84 (m, 1H), 2.12 - 3.06 (m, 3H). ^{13}C NMR (101 MHz, $CDCl_3$) δ 178.3, 153.0, 140.9, 140.5, 129.8, 127.0, 126.8, 125.4, 123.1, 120.0, 118.9, 49.9, 39.7, 29.2. HRMS (ESI-Orbitrap MS) m/z: Calcd. for $C_{16}H_{14}N_2O_4$ [M+H]$^+$: 299.1032; Found: 299.1025.

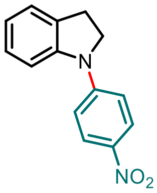

1-(4-nitrophenyl) indoline (3b-1)

Prepared according to general procedure B (PE: EtOAc = 40:1, v/v) from **1b-1** (59.6 mg) to afford **3b-1** (102.1 mg, 85% yield) as a yellow solid (m.p. 103-105 °C). ^{1}H NMR (400 MHz, $CDCl_3$) δ 8.15 (d, J = 9.2 Hz, 2H), 7.28 (d, J = 8.0 Hz, 1H), 7.19-7.09 (m, 4H), 6.86 (t, J = 7.4 Hz, 1H), 4.00 (t, J = 8.4 Hz, 2H), 3.14 (t, J = 8.2 Hz, 2H). ^{13}C NMR (101 MHz, $CDCl_3$) δ 149.1, 144.2, 139.5, 132.4, 127.2, 125.8, 125.6, 121.6, 114.7, 110.5, 52.0, 28.0. HRMS (m/z): calcd for $C_{14}H_{12}N_2O_2$ $[M+H]^+$: 241.0972, Found: 241.0960.

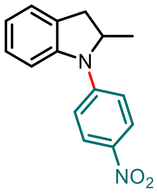

2-methyl-1-(4-nitrophenyl) indoline (3b-2)

Prepared according to general procedure B (PE: EtOAc = 40:1, v/v) from **1b-2** (66.6 mg) to afford **3b-2** (95.3 mg, 75% yield) as a yellow solid (m.p. 94-96 °C). ^{1}H NMR (400 MHz, $CDCl_3$) δ 8.11 (d, J = 9.2 Hz, 2H), 7.22-7.16 (m, 4H), 7.12 (t, J = 7.8 Hz, 1H), 6.87 (t, J = 7.4 Hz, 1H), 4.48-4.38 (m, 1H), 3.38 (dd, J = 15.4, 8.6 Hz, 1H), 2.62 (dd, J = 15.6, 2.4 Hz, 1H), 1.31 (d, J = 6.4 Hz, 3H). ^{13}C NMR (101 MHz, $CDCl_3$) δ 148.9, 143.3, 139.6, 131.4, 127.2, 125.9(2), 125.8(6), 122.0, 114.7, 112.1, 59.7, 36.7, 19.9. HRMS (m/z): calcd for $C_{15}H_{14}N_2O_2$ $[M+H]^+$: 255.1288, Found: 255.1134.

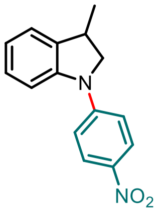

3-methyl-1-(4-nitrophenyl) indoline (3b-3)

Prepared according to general procedure B (PE: EtOAc = 40:1, v/v) from **1b-3** (66.6 mg) to afford **3b-3** (57.2 mg, 45% yield) as a yellow solid (m.p. 116-118 °C). ^{1}H NMR (400 MHz, $CDCl_3$) δ 8.12 (d, J = 9.2 Hz, 2H), 7.25 (d, J = 8.0 Hz, 1H), 7.16-7.08 (m,

4H), 6.88 (t, J = 7.8 Hz, 1H), 4.08 (t, J = 9.2 Hz, 1H), 3.55 (q, J = 2.4 Hz, 1H), 3.49-3.39 (m, 1H), 1.31 (d, J = 6.8 Hz, 3H). ^{13}C NMR (101 MHz, $CDCl_3$) δ 149.0, 143.7, 139.4, 137.6, 127.4, 125.8, 124.3, 121.8, 114.7, 110.4, 59.9, 34.6, 19.3. HRMS (m/z): calcd for $C_{15}H_{14}N_2O_2$ $[M+H]^+$: 255.1128, Found: 255.1132.

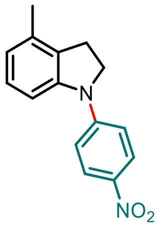

4-methyl-1-(4-nitrophenyl) indoline (3b-4)

Prepared according to general procedure B (PE: EtOAc = 40:1, v/v) from **1b-4** (66.6 mg) to afford **3b-4** (104.2 mg, 82% yield) as a yellow solid (m.p. 152-154 °C). ^{1}H NMR (400 MHz, $CDCl_3$) δ 8.08 (d, J = 9.2 Hz, 2H), 7.08 (d, J = 9.6 Hz, 3H), 7.00 (t, J = 7.8 Hz, 1H), 6.67 (d, J = 7.6 Hz, 1H), 3.96 (t, J = 8.2 Hz, 2H), 3.00 (t, J = 8.4 Hz, 2H), 2.18 (s, 3H). ^{13}C NMR (101 MHz, $CDCl_3$) δ 149.1, 143.7, 139.2, 135.0, 131.1, 127.2, 125.7, 122.8, 114.5, 108.1, 51.8, 26.7, 18.6. HRMS (m/z): calcd for $C_{15}H_{14}N_2O_2$ $[M+H]^+$: 255.1128, Found: 225.1117.

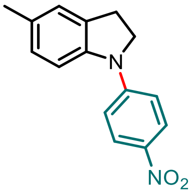

5-methyl-1-(4-nitrophenyl) indoline (3b-5)

Prepared according to general procedure B (PE: EtOAc = 40:1, v/v) from **1b-5** (66.6 mg) to afford **3b-5** (73.7 mg, 58% yield) as a yellow solid (m.p. 107-109 °C). ^{1}H NMR (400 MHz, $CDCl_3$) δ 8.12 (d, J = 8.4 Hz, 2H), 7.16 (d, J = 8.4 Hz, 1H), 7.08 (d, J = 8.8 Hz, 2H), 7.00 (s, 1H), 6.90 (d, J = 8.0 Hz, 1H), 3.97 (t, J = 8.2 Hz, 2H), 3.09 (t, J = 8.0 Hz, 2H), 2.24 (s, 3H). ^{13}C NMR (101 MHz, $CDCl_3$) δ 149.2, 141.7, 139.0, 132.7, 131.4, 127.5, 126.4, 125.8, 114.2, 110.5, 52.1, 27.9, 20.8. HRMS (m/z): calcd for $C_{15}H_{14}N_2O_2$ $[M+H]^+$: 255.1128, Found: 255.1116.

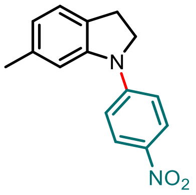

6-methyl-1-(4-nitrophenyl) indoline (3b-6)

Prepared according to general procedure B (PE: EtOAc = 40:1, v/v) from **1b-6** (66.6 mg) to afford **3b-6** (87.7 mg, 69% yield) as a yellow solid (m.p. 149-151 °C). ^{1}H NMR (400 MHz, $CDCl_3$) δ 8.14 (d, J = 9.2 Hz, 2H), 7.12 (t, J = 9.0 Hz, 3H), 7.06 (d, J = 7.6 Hz, 1H), 6.68 (d, J = 7.6 Hz, 1H), 3.98 (t, J = 8.2 Hz, 2H), 3.08 (t, J = 8.2 Hz, 2H), 2.28 (s, 3H). ^{13}C NMR (101 MHz, $CDCl_3$) δ 149.1, 144.4, 137.2, 130.0, 129.5, 125.8, 125.2, 122.3, 114.8, 111.4, 52.4, 27.7, 21.8. HRMS (m/z): calcd for $C_{15}H_{14}N_2O_2$ $[M+H]^+$: 255.1128, Found: 255.1130.

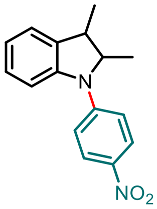

2,3-dimethyl-1-(4-nitrophenyl) indoline (3b-7)

Prepared according to general procedure B (PE: EtOAc = 40:1, v/v) from **1b-7** (73.6 mg) to afford **3b-7** (103.2 mg, 77% yield) as a yellow solid (m.p. 107-109 °C). ^{1}H NMR (400 MHz, $CDCl_3$) δ 8.11 (d, J = 9.2 Hz, 2H), 7.16 (t, J = 8.4 Hz, 4H), 7.10 (t, J = 7.6 Hz, 1H), 6.87 (t, J = 7.2 Hz, 1H), 3.96-3.89 (m, 1H), 2.89-2.82 (m, 1H), 1.29 (d, J = 6.4 Hz, 3H), 1.21 (d, J = 6.8 Hz, 3H). ^{13}C NMR (101 MHz, $CDCl_3$) δ 149.1, 142.9, 139.8, 136.6, 127.4, 125.8, 124.9, 121.9, 115.6, 111.4, 67.2, 43.7, 20.4, 19.1. HRMS (m/z): calcd for $C_{16}H_{16}N_2O_2$ $[M+H]^+$: 269.1285, Found: 269.1282.

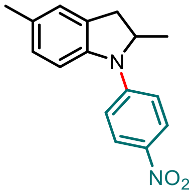

2,5-dimethyl-1-(4-nitrophenyl) indoline (3b-8)

Prepared according to general procedure B (PE: EtOAc = 40:1, v/v) from **1b-8** (73.6 mg) to afford **3b-8** (81.8 mg, 61% yield) as a yellow solid (m.p. 89-91 °C). ^{1}H NMR (400 MHz, $CDCl_3$) δ 8.07 (d, J = 9.2 Hz, 2H), 7.12-7.08 (m, 3H), 6.99 (s, 1H), 6.91 (d, J = 8.0 Hz, 1H), 4.44-4.34 (m, 1H), 3.33 (dd, J = 15.2, 8.4 Hz, 1H), 2.54 (d, J = 15.2 Hz, 1H), 2.24 (s, 3H), 1.27 (d, J = 6.4 Hz, 3H). ^{13}C NMR (101 MHz, $CDCl_3$) δ 158.8, 148.9, 140.4, 138.9, 131.8, 127.5, 126.7, 125.9, 113.7, 112.3, 59.7, 36.6, 20.9, 19.8. HRMS (m/z): calcd for $C_{16}H_{16}N_2O_2$ $[M+H]^+$: 269.1285, Found: 269.1285.

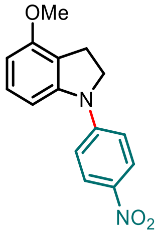

4-methoxy-1-(4-nitrophenyl) indoline (3b-9)

Prepared according to general procedure B (PE: EtOAc = 40:1, v/v) from **1b-9** (74.6 mg) to afford **3b-9** (56.7 mg, 42% yield) as a yellow solid (m.p. 177-179 °C). ^{1}H NMR (400 MHz, $CDCl_3$) δ 8.13 (d, J = 9.2 Hz, 2H), 7.15-7.07 (m, 3H), 6.91 (d, J = 8.0 Hz, 1H), 6.45 (d, J = 8.0 Hz, 1H), 4.01 (t, J = 8.4 Hz, 2H), 3.80 (s, 3H), 3.06 (t, J = 7.8 Hz, 2H). ^{13}C NMR (101 MHz, $CDCl_3$) δ 158.2, 156.6, 149.2, 145.7, 128.7, 125.8, 119.2, 114.8, 104.5, 104.1, 55.4, 52.5, 24.9. HRMS (m/z): calcd for $C_{15}H_{14}N_2O_3$ $[M+H]^+$: 271.1077, Found: 271.1083.

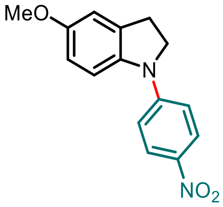

5-methoxy-1-(4-nitrophenyl) indoline (3b-10)

Prepared according to general procedure B (PE: EtOAc = 40:1, v/v) from **1b-10** (74.6 mg) to afford **3b-10** (97.8 mg, 68% yield) as a yellow solid (m.p. 157-159 °C) ^{1}H NMR (400 MHz, $CDCl_3$) δ 8.12 (d, J = 9.2 Hz, 2H), 7.19 (s, 1H), 7.06 (d, J = 9.2 Hz, 2H), 6.79 (s, 1H), 6.64 (d, J = 8.8 Hz, 1H), 3.99 (t, J = 8.2 Hz, 2H), 3.73 (s, 3H), 3.11 (t, J =

8.2 Hz, 2H). ^{13}C NMR (101 MHz, $CDCl_3$) δ 155.0, 149.1, 134.3, 125.9, 124.8, 113.7, 112.2, 111.5, 111.3, 102.2, 55.7, 52.1, 28.2. HRMS (m/z): calcd for $C_{15}H_{14}N_2O_3$ $[M+H]^+$: 271.1077, Found: 271.1077.

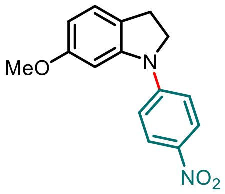

6-methoxy-1-(4-nitrophenyl) indoline (3b-11)

Prepared according to general procedure B (PE: EtOAc = 40:1, v/v) from **1b-11** (74.6 mg) to afford **3b-11** (63.5 mg, 47% yield) as a yellow solid (m.p. 140-142 °C). ^{1}H NMR (400 MHz, $CDCl_3$) δ 8.21 (d, J = 8.8 Hz, 2H), 7.21 (d, J = 8.8 Hz, 2H), 7.13 (d, J = 8.0 Hz, 1H), 6.91 (s, J = 2.0 Hz, 1H), 6.46 (d, J = 8.0 Hz, 1H), 4.07 (t, J = 8.2 Hz, 2H), 3.81 (s, 3H), 3.13 (t, J = 8.0 Hz, 2H). ^{13}C NMR (101 MHz, $CDCl_3$) δ 159.5, 149.0, 145.4, 125.8, 125.6, 125.5, 124.6, 115.0, 105.6, 98.5, 55.6, 52.9, 27.2. HRMS (m/z): calcd for $C_{15}H_{14}N_2O_3$ $[M+H]^+$: 271.1077, Found: 271.1063.

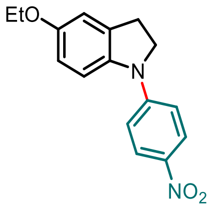

5-ethoxy-1-(4-nitrophenyl) indoline (3b-12)

Prepared according to general procedure B (PE: EtOAc = 40:1, v/v) from **1b-12** (81.6 mg) to afford **3b-12** (57.8 mg, 41% yield) as a yellow solid (m.p. 104-106 °C) ^{1}H NMR (400 MHz, $CDCl_3$) δ 8.18 (d, J = 8.8 Hz, 2H), 7.25 (d, J = 7.2 Hz, 1H), 7.12 (d, J = 9.2 Hz, 2H), 6.84 (s, 1H), 6.70 (d, J = 8.4 Hz, 1H), 4.05-3.98 (m, 4H), 3.17 (s, 2H), 1.41 (t, J = 7.0 Hz, 3H). ^{13}C NMR (101 MHz, $CDCl_3$) δ 154.4, 149.2, 138.7, 137.6, 134.2, 125.9, 113.7, 112.9, 112.4, 111.3, 64.0, 52.1, 28.2, 14.9. HRMS (m/z): calcd for $C_{16}H_{16}N_2O_2$ $[M+H]^+$: 285.1234, Found: 285.1237.

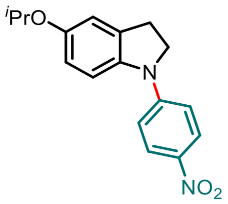

5-isopropoxy-1-(4-nitrophenyl) indoline (3b-13)

Prepared according to general procedure B (PE: EtOAc = 40:1, v/v) from **1b-13** (88.6 mg) to afford **3b-13** (67.1 mg, 45% yield) as a yellow solid (m.p. 87-89 °C). ^{1}H NMR (400 MHz, $CDCl_3$) δ 8.10 (d, J = 8.4 Hz, 2H), 7.17 (d, J = 8.8 Hz, 1H), 7.04 (d, J = 8.4 Hz, 2H), 6.77 (s, 1H), 6.63 (d, J = 8.4 Hz, 1H), 4.44-4.34 (m, 1H), 3.97 (t, J = 8.0 Hz, 2H), 3.09 (t, J = 8.2 Hz, 2H), 1.25 (d, J = 6.0 Hz, 6H). ^{13}C NMR (101 MHz, $CDCl_3$) δ 153.2, 149.1, 137.7, 134.2, 130.1, 125.9, 114.5, 114.3, 113.7, 111.4, 70.8, 52.1, 28.2, 22.1. HRMS (m/z): calcd for $C_{17}H_{18}N_2O_3$ [M+H]$^+$: 299.1390, Found: 299.1400.

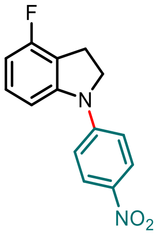

4-fluoro-1-(4-nitrophenyl) indoline (3b-14)

Prepared according to general procedure B (PE: EtOAc = 40:1, v/v) from **1b-14** (68.6 mg) to afford **3b-14** (73.6 mg, 57% yield) as a yellow solid (m.p. 131-133 °C). ^{1}H NMR (400 MHz, $CDCl_3$) δ 8.09 (d, J = 8.4 Hz, 2H), 7.09 (d, J = 8.4 Hz, 2H), 7.03 (t, J = 6.8 Hz, 1H), 6.98 (d, J = 7.6 Hz, 1H), 6.53 (t, J = 8.0 Hz, 1H), 4.01 (t, J = 8.2 Hz, 2H), 3.11 (t, J = 8.0 Hz, 2H). ^{13}C NMR (101 MHz, $CDCl_3$) δ 158.4, 154.7 (d, J = 4876.0 Hz), 146.7 (d, J = 36.0 Hz), 139.9, 129.1 (d, J = 32.0 Hz,), 125.6, 118.1 (d, J = 88.0 Hz), 115.1, 108.6 (d, J = 80.0 Hz), 106.2 (d, J = 12.0 Hz), 52.4, 24.0. ^{19}F NMR (376 MHz, $CDCl_3$) δ -117.8 (s, 1F). HRMS (m/z): calcd for $C_{14}H_{11}FN_2O_2$ [M+H]$^+$: 259.0877, Found: 259.0885.

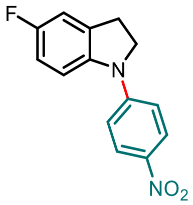

5-fluoro-1-(4-nitrophenyl) indoline (3b-15)

Prepared according to general procedure B (PE: EtOAc = 40:1, v/v) from **1b-15** (68.6 mg) to afford **3b-15** (77.4 mg, 60% yield) as a yellow solid (m.p. 119-121 °C). ^{1}H NMR (400 MHz, $CDCl_3$) δ 8.20 (d, J = 9.2 Hz, 2H), 7.24 (t, J = 4.4 Hz, 1H), 7.16 (d, J = 9.2 Hz, 2H), 6.97(dd, J = 7.8, 1.0 Hz, 1H), 6.89-6.83 (m, 1H), 4.09 (t, J = 8.2 Hz, 2H), 3.20 (t, J = 8.4 Hz, 2H). ^{13}C NMR (101 MHz, $CDCl_3$) δ 156.7, 154.0 (d, J = 4076.0 Hz), 134.5 (d, J = 32.0 Hz), 125.8, 125.5, 123.3, 114.3, 113.2 (d, J = 92.0 Hz), 113.1 (d, J = 96.0 Hz), 110.8 (d, J = 32.0 Hz), 52.4, 28.0. ^{19}F NMR (376 MHz, $CDCl_3$) δ -121.6 (s, 1F). HRMS (m/z): calcd for $C_{14}H_{11}FN_2O_2$ $[M+H]^+$: 259.0877, Found: 259.0868.

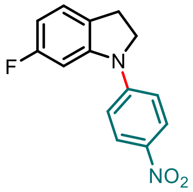

6-fluoro-1-(4-nitrophenyl) indoline (3b-16)

Prepared according to general procedure B (PE: EtOAc = 40:1, v/v) from **1b-16** (68.6 mg) to afford **3b-16** (96.1 mg, 71% yield) as a yellow solid (m.p. 133-135 °C). ^{1}H NMR (400 MHz, $CDCl_3$) δ 8.22 (d, J = 8.8 Hz, 2H), 7.20 (d, J = 8.8 Hz, 2H), 7.14 (t, J = 7.0 Hz, 1H), 7.01 (d, J = 10.4 Hz, 1H), 6.59 (t, J = 8.6 Hz, 1H), 4.11 (t, J = 8.4 Hz, 2H), 3.16 (t, J = 8.4 Hz, 2H). ^{13}C NMR (101 MHz, $CDCl_3$) δ 162.5 (d, J = 142.0 Hz), 148.6, 145.7 (d, J = 11.0 Hz), 140.2, 127.6 (d, J = 2.0 Hz), 125.9, 125.7(8), 125.7(6), 115.2, 107.5 (d, J = 23.0 Hz), 98.6 (d, J = 28.0 Hz), 52.9, 27.3. ^{19}F NMR (376 MHz, $CDCl_3$) δ -114.1 (s, 1F). HRMS (m/z): calcd for $C_{14}H_{11}FN_2O_2$ $[M+H]^+$: 259.0877, Found: 259.0884.

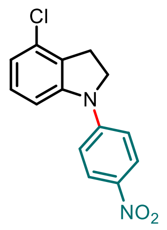

4-chloro-1-(4-nitrophenyl) indoline (3b-17)

Prepared according to general procedure B (PE: EtOAc = 40:1, v/v) from **1b-17** (76.6 mg) to afford **3b-17** (57.5 mg, 42% yield) as a yellow solid (m.p. 147-149 °C). ^{1}H NMR (400 MHz, $CDCl_3$) δ 8.22 (d, J = 9.2 Hz, 2H), 7.20 (t, J = 8.2 Hz, 3H), 7.11 (t, J = 7.8 Hz, 1H), 6.90 (d, J = 8.0 Hz, 1H), 4.11 (t, J = 8.8 Hz, 2H), 3.23 (t, J = 8.2 Hz, 2H). ^{13}C NMR (101 MHz, $CDCl_3$) δ 148.7, 145.6, 140.0, 131.4, 130.6, 128.7, 125.7, 121.4, 115.1, 108.4, 51.7, 27.3. HRMS (m/z): calcd for $C_{14}H_{11}ClN_2O_2$ $[M+H]^+$: 275.0582, Found: 275.0575.

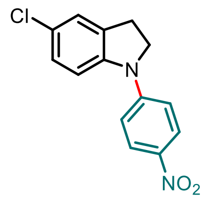

5-chloro-1-(4-nitrophenyl) indoline (3b-18)

Prepared according to general procedure B (PE: EtOAc = 40:1, v/v) from **1b-18** (76.6 mg) to afford **3b-18** (61.6 mg, 45% yield) as a yellow solid (m.p. 161-163 °C). ^{1}H NMR (400 MHz, $CDCl_3$) δ 8.12 (d, J = 9.2 Hz, 2H), 7.16-7.07 (m, 4H), 7.04 (d, J = 8.4 Hz, 1H), 4.00 (t, J = 8.2 Hz, 2H), 3.11 (t, J = 8.2 Hz, 2H). ^{13}C NMR (101 MHz, $CDCl_3$) δ 148.7, 143.0, 139.9, 134.4, 127.0, 126.1, 125.8, 125.7, 114.8, 111.0, 52.2, 27.8. HRMS (m/z): calcd for $C_{14}H_{11}ClN_2O_2$ $[M+H]^+$: 275.0582, Found: 275.0583.

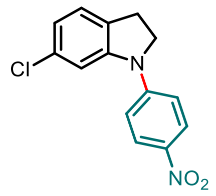

6-chloro-1-(4-nitrophenyl) indoline (3b-19)

Prepared according to general procedure B (PE: EtOAc = 40:1, v/v) from **1b-19** (76.6 mg) to afford **3b-19** (89.1 mg, 65% yield) as a yellow solid (m.p. 176-178 °C). ^{1}H NMR (400 MHz, $CDCl_3$) δ 8.14 (d, J = 9.2 Hz, 2H), 7.18 (s, 1H), 7.12 (d, J = 9.2 Hz, 2H), 7.06 (d, J = 8.0 Hz, 1H), 6.79 (d, J = 7.2 Hz, 1H), 4.01 (t, J = 8.4 Hz, 2H), 3.09 (t, J = 8.2 Hz, 2H). ^{13}C NMR (101 MHz, $CDCl_3$) δ 148.5, 145.5, 140.2, 132.9, 130.8, 126.1, 125.7, 121.2, 115.2, 110.5, 52.6, 27.4. HRMS (m/z): calcd for $C_{14}H_{11}ClN_2O_2$ $[M+H]^+$: 275.0582, Found: 275.0582.

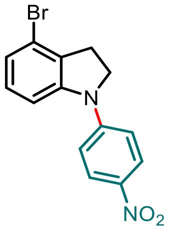

4-bromo-1-(4-nitrophenyl) indoline (3b-20)

Prepared according to general procedure B (PE: EtOAc = 40:1, v/v) from **1b-20** (98.5 mg) to afford **3b-20** (84.5 mg, 53% yield) as a yellow solid (m.p. 164-166 °C). ^{1}H NMR (400 MHz, $CDCl_3$) δ 8.14 (d, J = 9.2 Hz, 2H), 7.17-7.10 (m, 3H), 7.00-6.90 (m, 2H), 4.02 (t, J = 8.4 Hz, 2H), 3.12 (t, J = 8.4 Hz, 2H). ^{13}C NMR (101 MHz, CDCl3) δ 148.8, 145.4, 140.1, 132.8, 128.9, 125.7, 124.3, 120.4, 115.1, 108.9, 51.4, 29.4. HRMS (m/z): calcd for $C_{14}H_{11}BrN_2O_2$ $[M+H]^+$: 319.0077, Found: 319.0086.

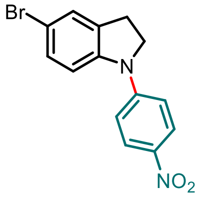

5-bromo-1-(4-nitrophenyl) indoline (3b-21)

Prepared according to general procedure B (PE: EtOAc = 40:1, v/v) from **1b-21** (98.5 mg) to afford **3b-21** (110.1 mg, 69% yield) as a yellow solid (m.p. 170-172 °C). ^{1}H NMR (400 MHz, $CDCl_3$) δ 8.15 (d, J = 9.2 Hz, 2H), 7.27 (s, 1H), 7.20-7.17 (m, 1H), 7.11 (dd, J = 9.0, 2.2 Hz, 3H), 4.01 (t, J = 8.4 Hz, 2H), 3.13 (t, J = 8.4 Hz, 2H). ^{13}C NMR (101 MHz, $CDCl_3$) δ 148.6, 143.4, 139.9, 134.8, 130.0, 128.6, 125.8, 114.9, 113.3,

111.5, 52.2, 27.7. HRMS (ESI-Orbitrap MS) m/z: Calcd. for $C_{14}H_{11}BrN_2O_2$ $[M+H]^+$: 319.00708; Found: 319.00767.

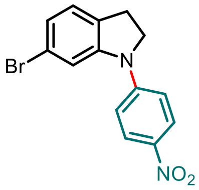

6-bromo-1-(4-nitrophenyl) indoline (3b-22)

Prepared according to general procedure B (PE: EtOAc = 40:1, v/v) from **1b-22** (98.5 mg) to afford **3b-22** (87.8 mg, 55% yield) as a yellow solid (m.p. 179-181 °C). ^1H NMR (400 MHz, $CDCl_3$) δ 8.16 (d, J = 9.2 Hz, 2H), 7.34 (s, 1H), 7.12 (d, J = 9.2 Hz, 2H), 7.01 (d, J = 8.0 Hz, 1H), 6.95 (dd, J = 7.6, 1.2 Hz, 1H), 4.01 (t, J = 8.2 Hz, 2H), 3.07 (t, J = 8.4 Hz, 1H). ^{13}C NMR (101 MHz, $CDCl_3$) δ 148.4, 145.8, 140.2, 131.4, 126.5, 125.8, 124.1, 120.8, 115.3, 113.2, 52.5, 27.5. HRMS (m/z): calcd for $C_{14}H_{11}BrN_2O_2$ $[M+H]^+$: 319.0077, Found:319.0087.

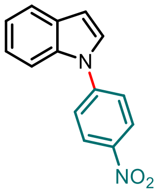

1-(4-nitrophenyl)-*1H*-indole (3b-23) [29]

Prepared according to general procedure A (PE: EtOAc = 40:1, v/v) from **1b-23** (58.5 mg) to afford **3b-23** (65.3 mg, 55% yield) as a yellow solid (m.p. 133-135 °C). ^1H NMR (400 MHz, $CDCl_3$) δ 8.42-8.36 (m, 2H), 7.72-7.62 (m, 4H), 7.38 (d, J = 3.2 Hz, 1H), 7.33-7.28 (m, 1H), 7.26-7.21 (m, 1H), 6.79-6.77 (m, 1H). ^{13}C NMR (101 MHz, $CDCl_3$) δ 145.2, 144.9, 135.1, 130.0, 127.1, 125.5, 123.4, 123.3, 121.6, 121.5, 110.4, 106.1.

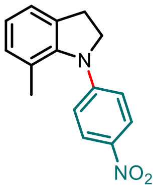

7-methyl-1-(4-nitrophenyl)indoline (3b-24)

Prepared according to general procedure B (PE: EtOAc = 40:1, v/v) from **1b-24** (73.6 mg) to afford **3b-24** (38.1 mg, 30% yield) as a yellow solid (m.p. 184-186℃). ^{1}H NMR (400 MHz, $CDCl_3$) δ 8.05 (d, J = 8.8 Hz, 2H), 7.05 (d, J = 6.8 Hz, 1H), 6.97 - 6.87 (m, 2H), 6.72 (d, J = 8.8 Hz, 2H), 4.09 (t, J = 7.6 Hz, 2H), 3.04 (t, J = 7.6 Hz, 1H), 1.93 (s, 3H). ^{13}C NMR (101MHz, $CDCl_3$) δ 158.6, 152.4, 143.4, 140.0, 134.2, 129.9, 125.0, 123.8, 122.6, 117.1, 56.8, 29.8, 19.6. HRMS(m/z): calcd for $C_{15}H_{14}N_2O_2$ $[M+H]^+$:255.1128, Found:255.1120.

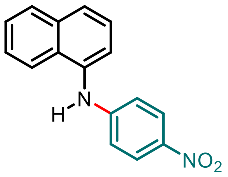

4-(naphthalen-1-ylamino) phenyl nitrate (3c-1)

Prepared according to general procedure A (PE: EtOAc = 10:1, v/v) from **1c-1** (71.6 mg) to afford give **3c-1** (105.7 mg, 80% yield) as a yellow solid (m.p. 174-176 °C). ^{1}H NMR (400 MHz, DMSO-d_6) δ 9.42 (s, 1H), 8.06 (d, J = 9.2 Hz, 2H), 8.03-8.00 (m, 1H), 7.99-9.75 (m, 1H), 7.81 (d, J = 7.6 Hz, 1H), 7.58-7.48 (m, 4H), 6.90 (d, J = 9.2 Hz, 2H). ^{13}C NMR (101 MHz, DMSO-d_6) δ 152.9, 137.6, 135.7, 134.4, 128.8, 128.3, 126.4, 126.1, 126.0(4), 125.9(8), 125.6, 122.7, 121.2, 113.0. HRMS (m/z): calcd for $C_{16}H_{12}N_2O_2$ $[M+H]^+$: 265.0972, Found: 265.0974.

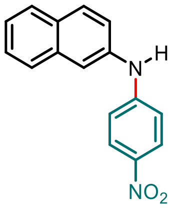

***N*-(4-nitrophenyl) naphthalen-2-amine (3c-2)**

Prepared according to general procedure A (PE: EtOAc = 10:1, v/v) from **1c-2** (71.6 mg) to afford give **3c-2** (104.3 mg, 79% yield) as a yellow solid (m.p. 171-173 °C). ^{1}H NMR (400 MHz, DMSO-d_6) δ 9.53 (s, 1H), 8.12 (d, J = 9.2 Hz, 2H), 7.90 (d, J = 8.8 Hz, 1H), 7.84 (t, J = 6.6 Hz, 2H), 7.74 (d, J = 1.2 Hz, 1H), 7.47 (t, J = 7.4 Hz, 1H), 7.42-7.37 (m, 2H), 7.19 (d, J = 9.2 Hz, 2H). ^{13}C NMR (101 MHz, DMSO-d_6) δ 150.5,

138.2, 137.9, 133.8, 129.8, 129.2, 127.5, 127.0, 126.6, 126.1, 124.6, 121.5, 115.7, 113.8. HRMS (m/z): calcd for $C_{16}H_{12}N_2O_2$ $[M+H]^+$: 265.0972, Found: 265.0978.

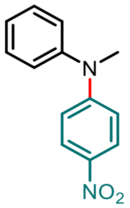

N-methyl-4-nitro-N-phenylaniline (3c-3)

Prepared according to general procedure A (PE: EtOAc = 40:1, v/v) from **1c-3** (53.6 mg) to afford give **3c-3** (106.1 mg, 93% yield) as a yellow solid (m.p. 65-67 °C). ^1H NMR (400 MHz, $CDCl_3$) δ 7.88 (d, J = 8.8 Hz, 2H), 7.32 (t, J = 7.8 Hz, 2H), 7.18 (t, J = 7.4 Hz, 1H), 7.10 (d, J = 8.0 Hz, 2H), 6.52 (d, J = 9.2 Hz, 2H), 3.27 (s, 3H). ^{13}C NMR (101 MHz, $CDCl_3$) δ 153.5, 146.0, 137.7, 130.0, 126.6, 126.5, 125.5, 112.1, 40.3. HRMS (m/z): calcd for $C_{13}H_{12}N_2O_2$ $[M+H]^+$: 229.0972, Found: 229.0965.

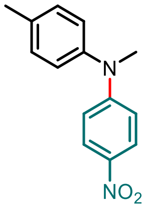

N,4-dimethyl-N-(4-nitrophenyl) aniline (3c-4)

Prepared according to general procedure A (PE: EtOAc = 40:1, v/v) from **1c-4** (60.6 mg) to afford give **3c-4** (105.3 mg, 87% yield) as a yellow solid (m.p. 75-77 °C). ^1H NMR (400 MHz, $CDCl_3$) δ 7.95 (d, J = 9.6 Hz, 2H), 7.14 (d, J = 8.0 Hz, 2H), 7.02 (d, J = 8.4 Hz, 2H), 6.54 (d, J = 9.2 Hz, 2H), 3.29 (s, 3H), 2.31 (s, 3H). ^{13}C NMR (101 MHz, $CDCl_3$) δ 153.9, 143.6, 137.8, 136.8, 130.8, 126.6, 125.7, 112.0, 40.5, 21.0. HRMS (m/z): calcd for $C_{14}H_{14}N_2O_2$ $[M+H]^+$: 243.1128, Found: 243.1125.

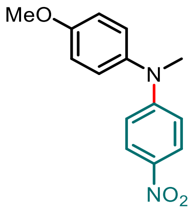

4-methoxy-*N*-methyl-*N*-(4-nitrophenyl) aniline (3c-5)

Prepared according to general procedure A (PE: EtOAc = 40:1, v/v) from **1c-5** (68.6 mg) to afford give **3c-5** (102.0 mg, 79% yield) as a yellow solid (m.p. 116-118 °C). ^{1}H NMR (400 MHz, $CDCl_3$) δ 7.96 (d, J = 9.2 Hz, 2H), 7.06 (d, J = 8.4 Hz, 2H), 6.90 (d, J = 8.4 Hz, 2H), 6.51 (d, J = 9.2 Hz, 2H), 3.77 (s, 3H), 3.29 (s, 3H). ^{13}C NMR (101 MHz, $CDCl_3$) δ 158.2, 154.2, 139.0, 137.6, 128.2, 125.8, 115.3, 111.7, 55.5, 40.7. HRMS (ESI-Orbitrap MS) m/z: Calcd. for $C_{14}H_{14}N_2O_3$ $[M+K]^+$: 297.06360; Found: 297.06296.

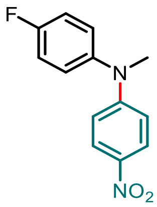

4-fluoro-*N*-methyl-*N*-(4-nitrophenyl) aniline (3c-6)

Prepared according to general procedure A (PE: EtOAc = 40:1, v/v) from **1c-6** (62.6 mg) to afford give **3c-6** (112.0 mg, 91% yield) as a yellow solid (m.p. 67-69 °C). ^{1}H NMR (400 MHz, $CDCl_3$) δ 7.98-7.93 (m, 2H), 7.16-7.11 (m, 2H), 7.07 (d, J = 8.4 Hz, 2H), 6.53 (d, J = 9.2 Hz, 2H), 3.30 (s, 3H). ^{13}C NMR (101 MHz, $CDCl_3$) δ 159.7, 158.0 (d, J =3376.0 Hz), 142.2, 138.0, 128.7 (d, J = 36.0 Hz), 125.7, 117.1 (d, J = 92.0 Hz), 112.1, 40.6. ^{19}F NMR (376 MHz, $CDCl_3$) δ -114.2 (s, 1F). HRMS (m/z): calcd for $C_{13}H_{11}FN_2O_2$ $[M+H]^+$: 247.0877, Found: 247.0872.

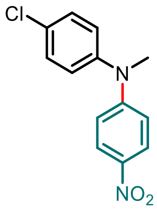

4-chloro-*N*-methyl-*N*- (4-nitrophenyl) aniline (3c-7)

Prepared according to general procedure A (PE: EtOAc = 40:1, v/v) from **1c-7** (70.6 mg) to afford give **3c-7** (117.9 mg, 90% yield) as a yellow solid (m.p. 82-84 °C). ^{1}H NMR (400 MHz, $CDCl_3$) δ 7.99 (d, J = 9.2 Hz, 2H), 7.35 (d, J = 8.4 Hz, 2H), 7.11 (d,

J = 8.4 Hz, 2H), 6.61 (d, *J* = 9.2 Hz, 2H), 3.32 (s, 3H). ^{13}C NMR (101 MHz, $CDCl_3$) δ 153.4, 144.9, 138.5, 132.1, 130.3, 127.9, 125.7, 112.7, 40.5. HRMS (m/z): calcd for $C_{13}H_{11}ClN_2O_2$ $[M+H]^+$: 263.0582, Found: 263.0575.

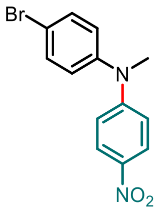

4-bromo-*N*-methyl-*N*-(4-nitrophenyl) aniline (3c-8)

Prepared according to general procedure A (PE: EtOAc = 40:1, v/v) from **1c-8** (92.5 mg) to afford give **3c-8** (132.7 mg, 87% yield) as a yellow solid (m.p. 111-113 °C). ^{1}H NMR (400 MHz, $CDCl_3$) δ 7.96 (d, *J* = 9.2 Hz, 2H), 7.48 (d, *J* = 8.4 Hz, 2H), 7.04 (d, *J* = 8.4 Hz, 2H), 6.61 (d, *J* = 9.2 Hz, 2H), 3.31 (s, 3H). ^{13}C NMR (101 MHz, $CDCl_3$) δ 153.3, 145.4, 138.5, 133.3, 128.2, 125.7, 119.8, 112.8, 40.4. HRMS (m/z): calcd for $C_{13}H_{11}BrN_2O_2$ $[M+H]^+$: 307.0077, Found: 307.0074.

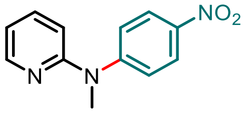

***N*-methyl-*N*-(4-nitrophenyl)pyridin-3-amine (3c-9)**

Prepared according to general procedure **A** (PE: EtOAc = 20:1, v /v) from **1c-9** (54.0 mg) to afford **3c-9** (58.5 mg, 85% yield) as yellow solid (m.p. 96-98 °C). ^{1}H NMR (400 MHz, $CDCl_3$) δ 8.35 (d, *J* = 4.8 Hz, 1H), 8.09 (d, *J* = 8.4 Hz, 1H), 7.14 (d, *J* = 9.2 Hz, 2H), 7.06 (d, *J* = 8.4 Hz, 1H), 6.93 (t, *J* = 6.2 Hz, 1H), 3.53 (s, 3H). ^{13}C NMR (101 MHz, $CDCl_3$) δ 157.3, 152.2, 148.6, 140.9, 137.8, 125.0, 118.6, 118.1, 114.8, 38.0. HRMS (m/z): calcd for $C_{12}H_{11}N_3O_2$ $[M+H]^+$: 230.0924, Found: 230.0923.

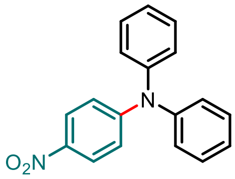

4-nitro-*N*,*N*-diphenylaniline (3c-10) [30a]

Prepared according to general procedure A (PE: EtOAc = 45:1, v/v) from **1c-10** (84.6 mg) to afford **3c-10** (63.5 mg, 73% yield) as yellow solid. ^{1}H NMR (400 MHz, $CDCl_3$) δ 7.21 (d, J = 8.8 Hz, 2H), 7.40-7.35 (m, 4H), 7.24-7.20 (m, 6H), 7.19-6.91 (m, 2H). ^{13}C NMR (101 MHz, $CDCl_3$) δ 153.4, 145.6, 140.1, 129.9, 126.5, 125.7, 125.4, 118.1.

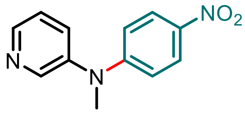

***N*-methyl-*N*-(4-nitrophenyl)pyridin-2-amine (3c-11)**

Prepared according to general procedure **A** (PE: EtOAc = 2:1, v/v) from **1c-11** (54.0 mg) to afford **3c-11** (60.5 mg, 88% yield) as yellow solid (m.p. 95-97 °C). ^{1}H NMR (400 MHz, $CDCl_3$) δ 8.43 (d, J = 2.0 Hz, 1H), 8.41 (d, J = 4.4 Hz, 1H), 7.89 (d, J = 9.2 Hz, 2H), 7.52 (d, J = 8.0 Hz, 1H), 7.32-7.28 (m, 1H), 6.60 (d, J = 9.6 Hz, 2H), 3.33 (s, 3H). ^{13}C NMR (101 MHz, $CDCl_3$) δ 152.8, 147.8, 146.9, 142.5, 138.5, 133.4, 125.3, 124.2, 112.7, 40.2. HRMS (m/z): calcd for $C_{12}H_{11}N_3O_2$ $[M+H]^+$: 230.0924, Found: 230.0934.

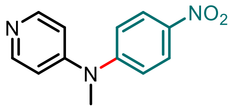

***N*-methyl-*N*-(4-nitrophenyl)pyridin-4-amine (3c-12)**

Prepared according to general procedure **A** (PE: EtOAc = 2:1, v/v) from **1c-12** (54.0 mg) to afford **3c-12** (48.1 mg, 70% yield) as yellow solid (m.p. 95-97 °C). ^{1}H NMR (400 MHz, $CDCl_3$) δ 8.40 (d, J = 0.4 Hz, 2H), 8.20 (d, J = 8.4 Hz, 2H), 7.23 (d, J = 8.4 Hz, 2H), 6.92 (d, J = 4.4 Hz, 2H), 3.45 (s, 3H). ^{13}C NMR (101 MHz, $CDCl_3$) δ 153.0, 151.7, 150.2, 125.5, 121.5, 113.2, 39.5. HRMS (m/z): calcd for $C_{12}H_{11}N_3O_2$ $[M+H]^+$: 230.0924, Found: 230.0934.

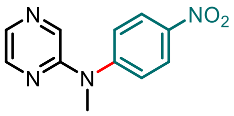

***N*-methyl-*N*-(4-nitrophenyl)pyrazin-2-amine (3c-13)**

Prepared according to general procedure **A** (PE: EtOAc = 8:1, v/v) from **1c-13** (54.5 mg) to afford **3c-13** (69.0 mg, 87% yield) as yellow solid (m.p. 95-97 °C). ^{1}H NMR (400 MHz, $CDCl_3$) δ 8.33 (s, 1H), 8.20-8.15 (m, 3H), 8.04 (d, *J* = 1.2 Hz, 1H), 7.31 (d, *J* = 8.8 Hz, 2H), 3.54 (s, 3H). ^{13}C NMR (101 MHz, $CDCl_3$) δ 153.6, 151.0, 142.9, 142.0, 136.2, 135.2, 125.2, 121.6, 37.7. HRMS (m/z): calcd for $C_{11}H_{10}N_4O_2$ $[M+H]^+$: 231.0877, Found: 231.0879.

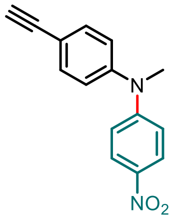

4-ethynyl-*N*-methyl-*N*-(4-nitrophenyl)aniline (3c-14)

Prepared according to general procedure A (PE: EtOAc = 20:1, v/v) from **1c-14** (65.6 mg) to afford **3c-14** (60.0 mg, 48% yield) as a yellow solid (m.p. 122 – 124 °C). ^{1}H NMR (400 MHz, $CDCl_3$) δ 8.08 (d, *J* = 9.2 Hz, 2H), 7.55 (d, *J* = 8.8 Hz, 2H), 7.19 (d, *J* = 8.4 Hz, 2H), 7.76 (d, *J* = 9.2 Hz, 2H), 3.41 (s, 3H), 3.12 (s, 1H). ^{13}C NMR (101 MHz, $CDCl_3$) δ 153.2, 146.7, 139.0, 133.9, 125.7(3), 125.6(9), 119.8, 113.6, 82.8, 77.9, 40.4. HRMS (ESI-Orbitrap MS) m/z: Calcd. for $C_{15}H_{12}N_2O_2$ $[M+H]^+$: 253.0977; Found: 253.0972.

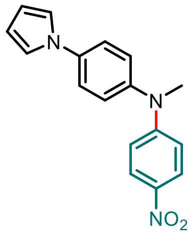

***N*-(4-(1H-pyrrol-1-yl)phenyl)-*N*-methyl-4-nitroaniline (3c-15)**

Prepared according to general procedure A (PE: EtOAc = 15:1, v/v) from **1c-15** (86.1 mg) to afford **3c-15** (74.0 mg, 51% yield) as a yellow solid (m.p. 164 – 166 °C). ^{1}H NMR (400 MHz, $CDCl_3$) δ 8.08 (d, *J* = 9.2 Hz, 2H), 7.47 (d, *J* = 8.8 Hz, 2H), 7.29 (d, *J* = 8.8 Hz, 2H), 7.10 (s, 2H), 6.71 (d, *J* = 9.2 Hz, 2H), 6.38 (s, 2H), 3.42 (s, 3H). ^{13}C NMR (101 MHz, $CDCl_3$) δ 153.6, 143.7, 139.0, 138.4, 127.8, 125.8, 121.9, 119.3, 112.6,

110.8, 40.6. HRMS (ESI-Orbitrap MS) m/z: Calcd. for $C_{17}H_{15}N_3O_2$ [M+H]$^+$: 294.1243; Found: 294.1241.

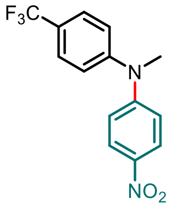

_N_-methyl-4-nitro-_N_-(4-(trifluoromethyl)phenyl)aniline (3c-16)

Prepared according to general procedure A (PE: EtOAc = 40:1, v/v) from **1c-16** (87.6 mg) to afford **3c-16** (111.0 mg, 83% yield) as a yellow solid (m.p. 93 – 95 °C). ^{1}H NMR (400 MHz, $CDCl_3$) δ 8.11 (d, J = 8.8 Hz, 2H), 7.68 (d, J = 8.4 Hz, 2H), 7.33 (d, J = 8.0 Hz, 2H), 6.85 (d, J = 8.8 Hz, 2H), 3.46 (s, 3H). ^{13}C NMR (101 MHz, $CDCl_3$) δ 153.0, 149.6, 139.6, 127.8 (d, J = 33.0 Hz), 127.2 (q, J = 3.0 Hz), 125.6, 125.2, 122.5, 114.6, 40.3. ^{19}F NMR (376 MHz, $CDCl_3$) δ -62.34 (s, 3F). HRMS (ESI-Orbitrap MS) m/z: Calcd. for $C_{14}H_{11}F_3N_2O_2$ [M+H]$^+$: 297.0851; Found: 297.0847.

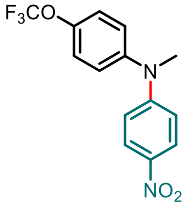

_N_-methyl-4-nitro-_N_-(4-(trifluoromethoxy)phenyl)aniline (3c-17)

Prepared according to general procedure A (PE: EtOAc = 40:1, v/v) from **1c-17** (95.6 mg) to afford **3c-17** (134.0 mg, 86% yield) as a yellow solid (m.p. 65 – 67 °C). ^{1}H NMR (400 MHz, $CDCl_3$) δ 8.08 (d, J = 8.8 Hz, 2H), 7.29 (q, J = 7.2 Hz, 4H), 6.70 (d, J = 8.8 Hz, 2H), 3.41 (s, 3H). ^{13}C NMR (101 MHz, $CDCl_3$) δ 153.4, 147.1, 145.0, 138.6, 127.9, 125.7, 122.7, 120.4 (q, J = 256.0 Hz), 112.8, 40.6. ^{19}F NMR (376 MHz, $CDCl_3$) δ -58.02 (s, 3F). HRMS (ESI-Orbitrap MS) m/z: Calcd. for $C_{14}H_{11}F_3N_2O_3$ [M+H]$^+$: 313.0800; Found: 313.0797.

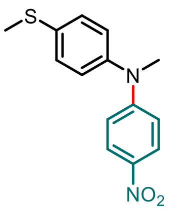

N-methyl-4-(methylthio)-N-(4-nitrophenyl)aniline (3c-18)

Prepared according to general procedure A (PE: EtOAc = 40:1, v/v) from **1c-18** (76.6 mg) to afford **3c-18** (100.0 mg, 74% yield) as a yellow solid (m.p. 87 – 89 °C). ^{1}H NMR (400 MHz, $CDCl_3$) δ 8.05 (d, J = 9.2 Hz, 2H), 7.32 (d, J = 8.4 Hz, 2H), 7.15 (d, J = 8.4 Hz, 2H), 6.66 (d, J = 9.2 Hz, 2H), 3.38 (s, 3H), 2.52 (s, 3H). ^{13}C NMR (101 MHz, $CDCl_3$) δ 153.7, 143.3, 138.1, 137.1, 128.0, 127.1, 125.7, 112.3, 40.4, 15.9. HRMS (ESI-Orbitrap MS) m/z: Calcd. for $C_{14}H_{14}N_2O_2S$ $[M+H]^+$: 275.0854; Found: 275.0849.

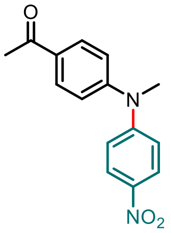

1-(4-(methyl(4-nitrophenyl)amino)phenyl)ethan-1-one (3c-19)

Prepared according to general procedure A (PE: EtOAc = 2:1, v/v) from **1c-19** (74.6 mg) to afford **3c-19** (58.0 mg, 43% yield) as a yellow oily liquid. ^{1}H NMR (400 MHz, DMSO-d^6) δ 8.10 (d, J = 9.2 Hz, 2H), 8.00 (d, J = 8.0 Hz, 2H), 7.40 (d, J = 8.4 Hz, 2H), 7.01 (d, J = 8.8 Hz, 2H), 3.45 (s, 3H), 3.00 (s, 3H). ^{13}C NMR (101 MHz, DMSO-d^6) δ 167.2, 153.5, 150.5, 139.1, 131.6, 127.6, 126.0, 124.8, 120.2, 115.8, 42.6. HRMS (ESI-Orbitrap MS) m/z: Calcd. for $C_{15}H_{14}N_2O_3$ $[M+H]^+$: 271.1083; Found: 271.1078.

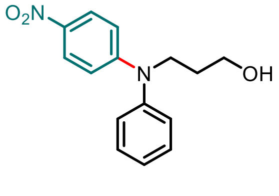

3-((4-nitrophenyl)(phenyl)amino)propan-1-ol (3c-20) [30b]

Prepared according to general procedure A (PE: EtOAc = 2:1, v/v) from **1c-20** (30.2 mg) to afford give **3c-20** (33.8 mg, 62% yield) as a red liquid. ^{1}H NMR (400 MHz,

CDCl$_3$) 8.00 (d, *J* = 9.2 Hz, 2H), 7.45 (t, *J* = 7.6 Hz, 2H), 7.32 (t, *J* = 7.6 Hz, 1H), 7.21 (d, *J* = 7.6 Hz, 2H), 6.66 (d, *J* = 8.0 Hz, 2H), 3.91 (t, *J* = 7.4 Hz, 2H), 3.74 (t, *J* = 6.0 Hz, 2H), 1.97 - 1.88 (m, 2H). ^{13}C NMR (100 MHz, CDCl$_3$) δ 153.6, 144.9, 137.8, 130.3, 127.5, 127.0, 126.1, 125.8, 115.6, 112.5, 59.8, 49.4, 29.9.

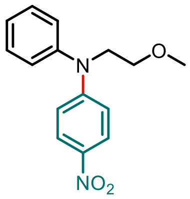

_N_-(2-methoxyethyl)-4-nitro-_N_-phenylaniline (3c-21)

Prepared according to general procedure A (PE: EtOAc = 40:1, v/v) from **1c-21** (75.6 mg) to afford **3c-21** (110.0 mg, 81% yield) as a yellow liquid. ^{1}H NMR (400 MHz, CDCl$_3$) δ 8.00 (d, *J* = 9.2 Hz, 2H), 7.46 (t, *J* = 7.6 Hz, 2H), 7.32 (t, *J* = 7.4 Hz, 1H), 7.24 (d, *J* = 7.6 Hz, 2H), 6.67 (d, *J* = 9.2 Hz, 2H), 3.94 (t, *J* = 5.8 Hz, 2H), 3.60 (t, *J* = 5.8 Hz, 2H), 3.34 (s, 3H). ^{13}C NMR (101 MHz, CDCl$_3$) δ 153.6, 144.9, 138.0, 130.2, 127.6, 126.9, 125.6, 112.8, 69.2, 58.9, 52.0. HRMS (ESI-Orbitrap MS) m/z: Calcd. for $C_{15}H_{16}N_2O_3$ [M+H]$^+$: 273.1239; Found: 273.1229.

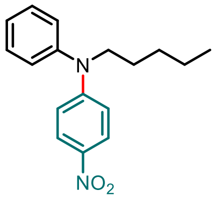

4-nitro-N-pentyl-N-phenylaniline (3c-22)

Prepared according to general procedure A (PE: EtOAc = 40:1, v/v) from **1c-22** (81.6 mg) to afford **3c-22** (106.5 mg, 75% yield) as a yellow liquid. ^{1}H NMR (400 MHz, CDCl$_3$) δ 8.01 (d, *J* = 9.2 Hz, 2H), 7.46 (t, *J* = 7.6 Hz, 2H), 7.32 (t, *J* = 7.8 Hz, 1H), 7.20 (d, *J* = 8.0 Hz, 2H), 6.58 (d, *J* = 9.2 Hz, 2H), 3.72 (t, *J* = 8.0 Hz, 2H), 1.74 - 1.66 (m, 2H), 1.39 - 1.25 (m, 4H), 0.91 - 0.87 (m, 3H). ^{13}C NMR (101 MHz, CDCl$_3$) δ 153.3, 145.1, 137.6, 130.2, 127.6, 126.9, 125.8, 112.2, 52.8, 28.9, 26.7, 22.3, 13.9. HRMS (ESI-Orbitrap MS) m/z: Calcd. for $C_{17}H_{20}N_2O_2$ [M+H]$^+$: 285.1603; Found: 285.1597.

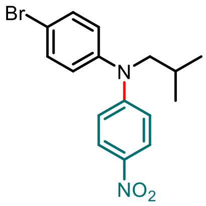

4-bromo-*N*-isobutyl-*N*-(4-nitrophenyl)aniline (3c-23)

Prepared according to general procedure A (PE: EtOAc = 40:1, v/v) from **1c-23** (113.6 mg) to afford **3c-23** (135.7 mg, 78% yield) as a yellow solid (m.p. 60-62 °C). ^{1}H NMR (400 MHz, $CDCl_3$) δ 8.03 (d, J = 8.8 Hz, 2H), 7.57 (d, J = 8.8 Hz, 2H), 7.11 (d, J = 8.8 Hz, 2H), 6.66 (d, J = 9.2 Hz, 2H), 3.57 (d, J = 7.6 Hz, 2H), 2.10 - 2.00 (m, 1H), 0.97 (d, J = 6.4 Hz, 6H). ^{13}C NMR (101 MHz, $CDCl_3$) δ 153.8, 144.8, 138.5, 133.4, 129.0, 125.7, 119.8, 113.4, 60.3, 27.3, 20.3. HRMS (ESI-Orbitrap MS) m/z: Calcd. for $C_{16}H_{17}BrN_2O_2$ [M+H]+: 349.0552; Found: 349.0544.

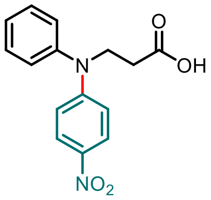

3-((4-nitrophenyl)(phenyl)amino)propanoic acid (3c-24)

Prepared according to general procedure A (PE: EtOAc = 2:1, v/v) from **1c-24** (82.6 mg) to afford **3c-24** (88.0 mg, 61% yield) as a yellow oily liquid. ^{1}H NMR (400 MHz, DMSO-d_6) δ 7.98 (d, J = 9.2 Hz, 2H), 7.47 (t, J = 7.6 Hz, 2H), 7.33 (t, J = 7.4 Hz, 2H), 7.26 (d, J = 8.0 Hz, 2H), 6.67 (d, J = 9.2 Hz, 2H), 3.98 (t, J = 7.2 Hz, 2H), 2.53 (t, J = 7.2 Hz, 2H). ^{13}C NMR (101 MHz, DMSO-d_6) δ 177.0, 157.6, 148.7, 141.6, 134.9, 132.2, 131.7, 130.3, 117.2, 52.6, 36.4. HRMS (ESI-Orbitrap MS) m/z: Calcd. for $C_{15}H_{14}N_2O_4$ [M+H]+: 287.1032; Found: 287.1029.

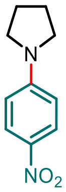

1-(4-nitrophenyl)pyrrolidine (3d-1) [31]

Prepared according to general procedure C (PE: EtOAc = 80:1, v/v) from **1d-1** (14.2 mg) to afford **3d-1** (41.5 mg, 72% yield) as a yellow solid. ^{1}H NMR (400 MHz, $CDCl_3$) δ 8.07 (d, J = 8.8 Hz, 2H), 7.37 (d, J = 8.8 Hz, 2H), 3.38 (t, J = 6.4 Hz, 4H), 2.08-2.04 (m, 4H). ^{13}C NMR (101 MHz, $CDCl_3$) δ 151.8, 136.4, 126.2, 110.3, 47.6, 25.3.

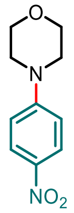

4-(4-nitrophenyl)morpholine (3d-2) [32]

Prepared according to general procedure C (PE: EtOAc = 40:1, v/v) from **1d-2** (17.4 mg) to afford **3d-2** (15.6 mg, 25% yield) as a yellow solid. ^{1}H NMR (400 MHz, $CDCl_3$) δ 8.12 (d, J = 8.8 Hz, 2H), 6.62 (d, J = 8.8 Hz, 2H), 3.85 (t, J = 4.8 Hz, 4H), 3.36 (t, J = 4.8 Hz, 4H). ^{13}C NMR (101 MHz, $CDCl_3$) δ 154.9, 138.9, 125.8, 112.6, 66.3, 47.1.

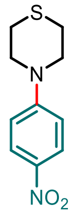

4-(4-nitrophenyl)thiomorpholine (3d-3) [33]

Prepared according to general procedure C (PE: EtOAc = 40:1, v/v) from **1d-3** (20.6 mg) to afford **3d-3** (14.8 mg, 33% yield) as a yellow solid. ^{1}H NMR (400 MHz, $CDCl_3$) δ 8.11 (d, J = 9.2 Hz, 2H), 6.77 (d, J = 9.2 Hz, 2H), 3.85 (t, J = 4.8 Hz, 4H), 2.71 (t, J = 4.8 Hz, 4H). ^{13}C NMR (101 MHz, $CDCl_3$) δ 153.6, 126.2, 112.8, 50.3, 25.8.

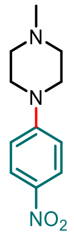

1-methyl-4-(4-nitrophenyl)piperazine (3d-4) [34]

Prepared according to general procedure C (PE: EtOAc = 10:1, v/v) from **1d-4** (20.0 mg) to afford **3d-4** (24.3 mg, 55% yield) as a yellow solid. ^{1}H NMR (400 MHz, DMSO-d_6) δ 8.01 (d, J = 9.2 Hz, 2H), 6.96 (d, J = 9.6 Hz, 2H), 3.40 (t, J = 5.2 Hz, 4H), 2.40 (t, J = 5.0 Hz, 4H), 2.20 (s, 3H). ^{13}C NMR (101 MHz, DMSO-d_6) δ 155.0, 137.2, 126.0, 112.8, 54.4, 46.5, 45.9.

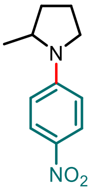

2-methyl-1-(4-nitrophenyl)pyrrolidine (3d-5) [35]

Prepared according to general procedure C (PE: EtOAc = 100:1, v/v) from **1d-5** (17.0 mg) to afford **3d-5** (23.9 mg, 58% yield) as a yellow solid. ^{1}H NMR (400 MHz, $CDCl_3$) δ 8.05 (d, J = 9.2 Hz, 2H), 6.44 (d, J = 9.2 Hz, 2H), 3.99 (t, J = 6.0 Hz, 1H), 3.48 (t, J = 8.4 Hz, 1H), 3.26 (q J = 7.8 Hz, 1H), 2.16-2.03 (m, 3H), 1.77 (q, J = 11.6 Hz, 1H), 1.18 (d, J = 6.4 Hz, 3H). ^{13}C NMR (101 MHz, $CDCl_3$) δ 151.1, 136.1, 126.2, 110.5, 54.1, 48.0, 32.6, 22.8, 18.4.

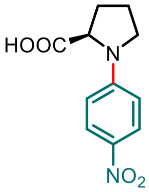

(4-nitrophenyl)proline (3d-6) [35]

Prepared according to general procedure C (PE: EtOAc = 10:1, v/v) from **1d-6** (23.0 mg) to afford **3d-6** (23.6 mg, 50% yield) as a yellow solid. ^{1}H NMR (400 MHz, DMSO-d_6) δ 8.02 (d, J = 8.8 Hz, 2H), 6.53 (d, J = 8.8 Hz, 2H), 4.36 (d, J = 7.6 Hz, 1H), 3.50 (q, J = 8 Hz, 1H), 3.38 (q J = 8.2 Hz, 1H), 2.31-2.20 (m, 1H), 2.12 (d, J = 12.4 Hz, 1H), 2.00 (q, J = 8.8 Hz, 2H). ^{13}C NMR (101 MHz, DMSO-d_6) δ 174.2, 151.9, 136.5, 126.3, 111.7, 61.2, 48.9, 30.8, 23.4.

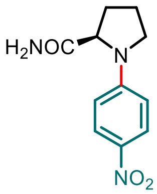

1-(4-nitrophenyl)pyrrolidine-2-carboxamide (3d-7) [36]

Prepared according to general procedure C (PE: EtOAc = 10:1, v/v) from **1d-7** (22.8 mg) to afford **3d-7** (25.4 mg, 54% yield) as a yellow solid. ^{1}H NMR (400 MHz, DMSO-d_6) δ 8.06 (d, J = 9.2 Hz, 2H), 7.62 (s, 1H), 7.17 (s, 1H), 6.55 (d, J = 8.8 Hz, 2H), 4.21 (d J = 8.8 Hz, 1H), 3.66-3.60 (m, 1H), 3.38 (q, J = 8.4 Hz, 1H), 2.29-2.20 (m, 1H), 2.00 (q, J = 5.6 Hz, 3H). ^{13}C NMR (101 MHz, DMSO-d_6) δ 174.0, 152.0, 136.4, 126.2, 111.8, 62.1, 49.1, 31.4, 23.7.

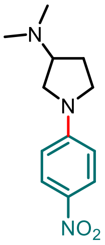

***N*,*N*-dimethyl-1-(4-nitrophenyl)pyrrolidin-3-amine (3d-8)** [37]

Prepared according to general procedure C (PE: EtOAc = 10:1, v/v) from **1d-8** (14.2 mg) to afford **3d-8** (26.3 mg, 56% yield) as a yellow solid. ^{1}H NMR (400 MHz, DMSO-d_6) δ 7.97 (d, J = 8.8 Hz, 2H), 6.51 (d, J = 8.8 Hz, 2H), 3.53-3.41 (m, 2H), 3.26 (q, J = 9.8 Hz, 1H), 3.06 (t J = 9.2 Hz, 1H), 2.76-2.68 (m, 1H), 2.16 (s, 7H), 1.83-1.72 (m, 1H). ^{13}C NMR (101 MHz, DMSO-d_6) δ 151.9, 135.8, 126.1, 110.9, 65.0, 52.4, 47.2, 44.1, 29.7.

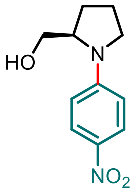

(1-(4-nitrophenyl)pyrrolidin-2-yl)methanol (3d-9) [38]

Prepared according to general procedure C (PE: EtOAc = 2:1, v/v) from **1d-9** (20.2 mg) to afford **3d-9** (32.4 mg, 73% yield) as a yellow solid. ^{1}H NMR (400 MHz, DMSO-d_6) δ 8.02 (d, J = 9.2 Hz, 2H), 6.65 (d, J = 9.2 Hz, 2H), 4.89 (s, 1H), 3.90 (q, J = 7.2 Hz, 1H), 3.46 (q, J = 5.8 Hz, 2H), 3.30 (q, J = 8.0 Hz, 1H), 3.20 (q, J = 8.4 Hz, 1H), 2.05-1.89 (m, 4H). ^{13}C NMR (101 MHz, DMSO-d_6) δ 152.2, 135.8, 126.3, 111.6, 60.9, 60.7, 48.7, 28.1, 22.8.

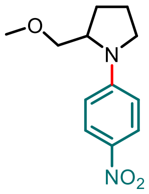

2-(methoxymethyl)-1-(4-nitrophenyl)pyrrolidine (3d-10) [35]

Prepared according to general procedure C (PE: EtOAc = 10:1, v/v) from **1d-10** (30.4 mg) to afford **3d-10** (29.3 mg, 62% yield) as a yellow solid. ^{1}H NMR (400 MHz, DMSO-d_6) δ 7.99 (d, J = 9.2 Hz, 2H), 6.53 (d, J = 9.2 Hz, 2H), 3.49-3.26 (m, 8H), 3.08 (q, J = 7 Hz, 1H), 2.63-2.52 (m, 1H), 2.11-2.03 (m, 1H), 1.78-1.68 (m, 1H). ^{13}C NMR (101 MHz, DMSO-d_6) δ 152.1, 135.8, 126.2, 111.1, 74.0, 58.6, 51.0, 47.5, 38.3, 28.0.

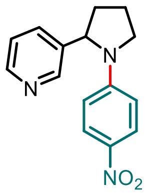

3-(1-(4-nitrophenyl)pyrrolidin-2-yl)pyridine (3d-11) [39]

Prepared according to general procedure C (PE: EtOAc = 10:1, v/v) from **1d-11** (29.6 mg) to afford **3d-11** (21.5 mg, 45% yield) as a yellow solid. ^{1}H NMR (400 MHz, DMSO-d_6) δ 8.46 (d, J = 8.0 Hz, 2H), 7.97 (d, J = 9.2 Hz, 2H), 7.55 (d, J = 8.0 Hz, 1H), 7.30 (t, J = 6.2 Hz, 1H), 6.51 (d, J = 8.4 Hz, 2H), 5.08 (d, J = 7.6 Hz, 1H), 3.82 (t, J = 7.6 Hz, 1H), 2.42 (q, J = 9.2 Hz, 1H), 1.99-1.85 (m, 4H). ^{13}C NMR (101 MHz, DMSO-d_6) δ 151.4, 148.7, 148.0, 138.2, 136.4, 133.8, 126.1, 123.9, 112.0.

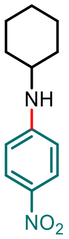

***N*-cyclohexyl-4-nitroaniline (3d-12)** [40]

Prepared according to general procedure C (PE: EtOAc = 15:1, v/v) from **1d-12** (19.8 mg) to afford **3d-12** (12.3 mg, 28% yield) as a yellow solid. ^{1}H NMR (400 MHz, DMSO-*d*$_6$) δ 7.96 (d, *J* = 8.8 Hz, 2H), 7.14 (d, *J* = 7.6 Hz, 2H), 6.62 (d, *J* = 9.2 Hz, 2H), 3.38-3.28 (m, 1H), 1.89 (d, *J* = 10.4 Hz, 2H), 1.70 (d, *J* = 13.2 Hz, 2H), 1.56 (d, *J* = 12.8 Hz, 1H), 1.33 (q, *J* = 12.2 Hz, 2H), 1.25-1.11 (m, 3H). ^{13}C NMR (101 MHz, DMSO-*d*$_6$) δ 154.0, 135.6, 126.6, 111.23, 51.0, 32.5, 25.6, 24.7.

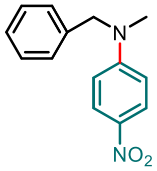

***N*-benzyl-*N*-methyl-4-nitroaniline (3d-13)** [35]

Prepared according to general procedure C (PE: EtOAc = 10:1, v/v) from **1d-13** (21.4 mg) to afford **3d-13** (20.3 mg, 42% yield) as a yellow solid. ^{1}H NMR (400 MHz, DMSO-*d*$_6$) δ 8.02 (d, *J* = 8.8 Hz, 2H), 7.32 (t, *J* = 7.2 Hz, 2H), 7.26-7.17 (m, 3H), 4.74 (s, 2H), 3.16 (s, 3H). ^{13}C NMR (101 MHz, DMSO-*d*$_6$) δ 154.1, 137.7, 136.3, 129.0, 127.4, 126.8, 126.2, 111.2, 55.4, 39.4.

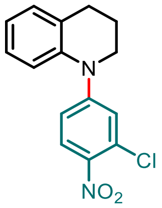

1-(3-chloro-4-nitrophenyl)-1,2,3,4-tetrahydroquinoline (4a-1)

Prepared according to general procedure A (PE: EtOAc = 50:1, v/v) from **1a-1** (66.6 mg) to afford **4a-1** (99.1 mg, 69% yield) as a yellow solid (m.p. 75-77 °C). ^{1}H NMR

(400 MHz, $CDCl_3$) δ 7.90 (d, J = 9.2 Hz, 1H), 7.16-7.04 (m, 4H), 7.00 (dd, J = 9.2, 2.4 Hz, 1H), 6.94 (t, J = 7.4 Hz, 1H), 3.60 (t, J = 6.2 Hz, 2H), 2.68 (t, J = 6.2 Hz, 2H), 2.00-1.93 (m, 2H). ^{13}C NMR (101 MHz, $CDCl_3$) δ 152.1, 139.9, 131.4, 129.7, 129.2, 127.9, 126.5, 125.3, 123.3, 120.7, 119.4, 115.4, 48.6, 27.0, 24.0. HRMS (m/z): calcd for $C_{15}H_{13}ClN_2O_2$ $[M+H]^+$: 289.0738, Found: 289.0740.

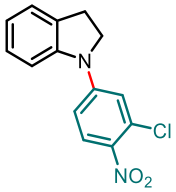

1-(3-chloro-4-nitrophenyl) indoline (4b-1)

Prepared according to general procedure B (PE: EtOAc = 40:1, v/v) from **1b-1** (59.6 mg) to afford **4b-1** (42.4 mg, 31% yield) as a yellow solid (m.p. 78-80 °C). ^{1}H NMR (400 MHz, $CDCl_3$) δ 8.02 (d, J = 9.2 Hz, 1H), 7.25 (d, J = 8.0 Hz, 1H), 7.19-7.10 (m, 3H), 7.05 (dd, J = 9.2, 2.4 Hz, 1H), 6.88 (t, J = 7.4 Hz, 1H), 3.97 (t, J = 8.2 Hz, 2H), 3.14 (t, J = 8.2 Hz, 2H). ^{13}C NMR (101 MHz, $CDCl_3$) δ 148.0, 143.7, 138.0, 132.5, 130.2, 128.4, 127.3, 125.7, 122.1, 117.4, 113.2, 110.6, 52.0, 28.0. HRMS (m/z): calcd for $C_{14}H_{11}ClN_2O_2$ $[M+H]^+$: 275.0582, Found: 275.0591.

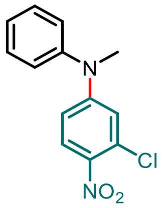

3-chloro-*N*-methyl-4-nitro-*N*-phenylaniline (4c-1)

Prepared according to general procedure A (PE: EtOAc = 40:1, v/v) from **1c-3** (53.6 mg) to afford **4c-1** (94.0 mg, 72% yield) as a yellow solid (m.p. 83-85 °C) ^{1}H NMR (400 MHz, $CDCl_3$) δ 7.88 (d, J = 9.2 Hz, 1H), 7.40 (t, J = 7.6 Hz, 2H), 7.26 (t, J = 7.4 Hz, 1H), 7.14 (d, J = 7.6 Hz, 2H), 6.63 (d, J = 2.8 Hz, 1H), 6.48 (dd, J = 9.2, 2.8 Hz, 1H), 3.31 (s, 3H). ^{13}C NMR (101 MHz, $CDCl_3$) δ 152.7, 145.6, 136.2, 130.3, 130.1, 128.3, 127.1, 126.6, 114.8, 111.1, 40.5. HRMS (m/z): calcd for $C_{13}H_{11}ClN_2O_2$ $[M+H]^+$: 263.0582, Found: 263.0589.

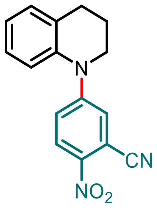

5-(3,4-dihydroquinolin-1(2*H*)-yl)-2-nitrobenzonitrile (4a-2)

Prepared according to general procedure A (PE: EtOAc = 30:1, v/v) from **1a-1** (66.6 mg) to afford **4a-2** (62.8 mg, 45% yield) as a yellow solid (m.p. 155-157 °C). ^{1}H NMR (400 MHz, $CDCl_3$) δ 8.20 (d, J = 9.6 Hz, 1H), 7.51 (d, J = 2.4 Hz, 1H), 7.35 (dd, J = 9.2, 2.8 Hz, 1H), 7.26-7.16 (m, 3H), 7.09 (t, J = 7.4 Hz, 1H), 3.72 (t, J = 6.4 Hz, 2H), 2.76 (t, J = 6.2 Hz, 2H), 2.11-2.04 (m, 2H). ^{13}C NMR (101 MHz, $CDCl_3$) δ 152.3, 139.1, 138.6, 132.6, 129.3, 127.4, 126.9, 124.5, 122.4, 121.0, 119.1, 115.8, 109.7, 48.6, 27.0, 24.3. HRMS (m/z): calcd for $C_{16}H_{13}N_3O_2$ $[M+H]^+$: 280.1081, Found: 280.1071.

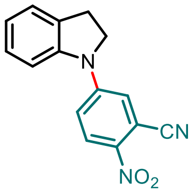

5-(indolin-1-yl)-2-nitrobenzonitrile (4b-2)

Prepared according to general procedure B (PE: EtOAc = 30:1, v/v) from **1b-1** (59.6 mg) to afford **4b-2** (86.2 mg, 65% yield) as a yellow solid (m.p. 170-172 °C). ^{1}H NMR (400 MHz, $CDCl_3$) δ 8.22 (d, J = 9.2 Hz, 1H), 7.47 (d, J = 2.4 Hz, 1H), 7.33 (dd, J = 9.2, 2.4 Hz, 1H), 7.28 (d, J = 8.4 Hz, 1H), 7.23 (t, J = 7.2 Hz, 1H), 7.19-7.14 (m, 1H), 6.95 (t, J =7.4 Hz, 1H), 4.03 (t, J = 8.2 Hz, 2H), 3.19 (t, J = 8.0 Hz, 2H). ^{13}C NMR (101 MHz, $CDCl_3$) δ 148.2, 143.0, 138.4, 132.8, 127.7, 127.5, 126.0, 123.1, 120.7, 117.4, 115.8, 111.0, 110.1, 52.2, 28.0. HRMS (m/z): calcd for $C_{15}H_{11}N_3O_2$ $[M+H]^+$: 266.0924, Found: 266.0929.

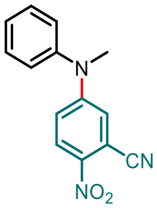

5-(methyl(phenyl)amino)-2-nitrobenzonitrile (4c-2)

Prepared according to general procedure A (PE: EtOAc = 30:1, v/v) from **1c-3** (53.6 mg) to afford **4c-2** (77.2 mg, 61% yield) as a yellow solid (m.p. 89-91 °C). ^{1}H NMR (400 MHz, $CDCl_3$) δ 8.05 (d, J = 9.2 Hz, 1H), 7.44 (t, J = 7.8 Hz, 1H), 7.32 (t, J = 7.4 Hz, 1H), 7.15 (d, J = 8.0 Hz, 1H), 6.87 (d, J = 2.4 Hz, 1H), 6.75 (dd, J = 9.6, 2.8 Hz, 1H), 3.37 (s, 3H). ^{13}C NMR (101 MHz, $CDCl_3$) δ 152.8, 144.9, 137.0, 130.7, 127.9, 127.6, 126.6, 118.5, 116.1, 114.9, 109.7, 40.7. HRMS (m/z): calcd for $C_{14}H_{11}N_3O_2$ $[M+H]^+$: 254.0924, Found: 254.0932.

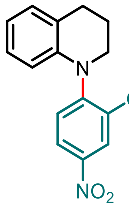

2-(3,4-dihydroquinolin-1(2*H*)-yl)-5-nitrobenzonitrile (4a-3)

Prepared according to general procedure A (PE: EtOAc = 30:1, v/v) from **1a-1** (66.6 mg) to afford **4a-3** (76.8 mg, 55% yield) as a yellow solid (m.p. 90-92 °C). ^{1}H NMR (400 MHz, $CDCl_3$) δ 8.41 (d, J = 2.4 Hz, 1H), 8.13 (dd, J = 9.4, 2.6 Hz, 1H), 7.30 (d, J = 9.2 Hz, 1H), 7.11 (d, J = 7.2 Hz, 1H),7.01 (t, J = 7.0 Hz, 1H), 6.94 (t, J = 7.2 Hz, 1H), 6.84 (d, J = 7.6 Hz, 1H), 3.83 (t, J = 5.6 Hz, 2H), 2.83 (t, J = 6.4 Hz, 2H), 2.02-1.94 (m, 2H). ^{13}C NMR (101 MHz, $CDCl_3$) δ 157.0, 141.0, 140.8, 131.2, 130.1, 128.7, 128.1, 126.7, 124.2, 123.6, 120.0, 116.3, 105.8, 51.7, 26.6, 23.0. HRMS (m/z): calcd for $C_{16}H_{13}N_3O_2$ $[M+H]^+$: 280.1081, Found: 280.1071.

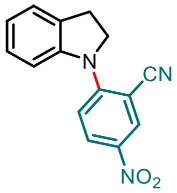

2-(indolin-1-yl)-5-nitrobenzonitrile (4b-3)

Prepared according to general procedure B (PE: EtOAc = 30:1, v/v) from **1b-1** (59.6 mg) to afford **4b-3** (68.9 mg, 52% yield) as a yellow solid (m.p. 169-171 °C). ^{1}H NMR (400 MHz, $CDCl_3$) δ 8.44 (d, J = 2.8 Hz, 1H), 8.16 (dd, J = 9.2, 2.8 Hz, 1H), 7.60 (d, J

= 9.2 Hz, 1H), 7.24 (d, *J* = 7.2 Hz, 1H), 7.16-7.07 (m, 2H), 6.96 (t, *J* = 7.2 Hz, 1H), 4.33 (t, *J* = 8.2 Hz, 2H), 3.18 (t, *J* =8.0 Hz, 2H). ^{13}C NMR (101 MHz, $CDCl_3$) δ 152.7, 143.6, 140.2, 132.9, 131.8, 128.4, 127.1, 125.9, 123.6, 118.4, 116.9, 112.8, 101.2, 55.2, 29.4. HRMS (m/z): calcd for $C_{15}H_{11}N_3O_2$ $[M+H]^+$: 266.0924, Found: 266.0929.

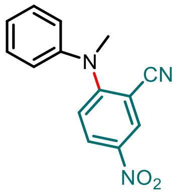

2-(indolin-1-yl)-5-nitrobenzonitrile (4c-3)

Prepared according to general procedure **B** (PE: EtOAc = 30:1, v/v) from **1c-3** (53.6 mg) to afford **4c-3** (22.8 mg, 30% yield) as yellow solid (m.p. 93-95 °C). ^{1}H NMR (400 MHz, $CDCl_3$) δ 8.35 (d, *J* = 2.4 Hz, 1H), 8.20 (dd, *J* = 5.6 Hz, 1H), 7.46 (t, *J* = 7.8 Hz, 2H), 7.34 (t, *J* = 7.4 Hz, 1H), 7.22 (d, *J* = 8.0 Hz, 2H), 6.96 (d, *J* = 9.2 Hz, 1H), 3.58 (s, 3H). ^{13}C NMR (101 MHz, $CDCl_3$) δ 155.1, 146.5, 138.6, 132.2, 130.2, 128.6, 127.3, 125.6, 117.5, 116.0, 99.4, 42.8. HRMS (m/z): calcd for $C_{11}H_{10}N_4O_2$ $[M+H]^+$: 254.0923, Found: 254.0928.

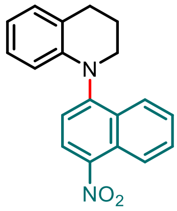

1-(4-nitronaphthalen-1-yl)-4-phenyl-1,2,3,4-tetrahydroquinoline (4a-4)

Prepared according to general procedure A (PE: EtOAc = 50:1, v/v) from **1a-1** (66.6 mg) to afford **4a-4** (110.3 mg, 58% yield) as a yellow liquid. ^{1}H NMR (400 MHz, $CDCl_3$) δ 8.61 (d, *J* = 8.8 Hz, 1H), 8.20 (d, *J* = 8.4 Hz, 1H), 8.05 (d, *J* = 8.8 Hz, 1H), 7.64 (t, *J* = 7.8 Hz, 1H), 7.47 (t, *J* = 7.6 Hz, 1H), 7.31 (d, *J* = 8.0 Hz, 1H), 7.04 (d, *J* = 7.2 Hz, 1H), 6.77 (t, *J* = 7.4 Hz, 1H), 6.68 (t, *J* = 7.2 Hz, 1H), 6.15 (d, *J* = 8.4 Hz, 1H), 3.61 (t, *J* = 5.4 Hz, 2H), 2.92 (t, *J* = 6.6 Hz, 2H), 2.10-2.03 (m, 2H). ^{13}C NMR (101 MHz, $CDCl_3$) δ 151.9, 144.7, 143.2, 131.1, 129.7, 129.6, 127.2, 127.1, 126.6, 125.4,

124.8, 124.2, 123.9, 122.0, 119.3, 116.6, 51.9, 27.5, 22.3. HRMS (m/z): calcd for $C_{19}H_{16}N_2O_2$ $[M+H]^+$: 305.1290, Found: 305.1288.

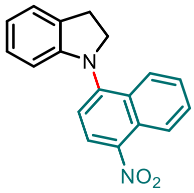

1-(4-nitronaphthalen-1-yl) indoline (4b-4)

Prepared according to general procedure B (PE: EtOAc = 40:1, v/v) from **1b-1** (59.6 mg) to afford **4b-4** (68.2 mg, 47% yield) as a yellow solid (m.p. 148-150 °C). ^{1}H NMR (400 MHz, $CDCl_3$) δ 8.76 (d, J = 8.8 Hz, 1H), 8.32 (d, J = 8.4 Hz, 1H), 8.17 (d, J = 8.4 Hz, 1H), 7.73 (t, J = 7.6 Hz, 1H), 7.53 (t, J = 7.6 Hz, 1H), 7.36 (d, J = 8.4 Hz, 1H), 7.27 (d, J = 7.6 Hz, 1H), 6.98 (t, J = 7.6 Hz, 1H), 6.85 (t, J = 7.4 Hz, 1H), 6.39 (d, J = 8.0 Hz, 1H), 4.08 (t, J = 8.0 Hz, 2H), 3.26 (t, J = 8.2 Hz, 2H). ^{13}C NMR (101 MHz, $CDCl_3$) δ 148.7, 148.6, 142.0, 131.5, 129.8, 128.9, 127.0, 126.9, 126.3, 125.8, 125.4, 125.1, 123.9, 120.4, 114.9, 110.5, 55.4, 29.0. HRMS (ESI-Orbitrap MS) m/z: Calcd. for $C_{18}H_{14}N_2O_2$ $[M+H]^+$: 291.11280; Found: 291.11227.

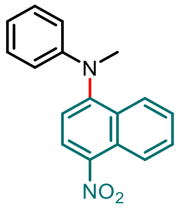

***N*-methyl-4-nitro-*N*-phenylnaphthalen-1-amine (4c-4)**

Prepared according to general procedure A (PE: EtOAc = 40:1, v/v) from **1c-3** (53.6 mg) to afford **4c-4** (80.7 mg, 58% yield) as a yellow liquid. ^{1}H NMR (400 MHz, $CDCl_3$) δ 8.54 (d, J = 8.8 Hz, 1H), 8.14 (d, J = 8.4 Hz, 1H), 7.90 (d, J = 8.8 Hz, 1H), 7.53 (t, J = 8.2 Hz, 1H), 7.34 (t, J = 7.8 Hz, 1H), 7.20 (d, J = 8.4 Hz, 1H), 7.07 (t, J = 8.0 Hz, 2H), 6.75 (t, J = 7.4 Hz, 1H), 6.63 (d, J = 8.0 Hz, 2H), 3.31 (s, 3H). ^{13}C NMR (101 MHz, $CDCl_3$) δ 152.1, 149.5, 143.1, 130.7, 129.4, 129.1, 127.1, 127.0, 125.4, 125.0, 123.7, 120.8, 120.0, 116.7, 41.2. HRMS (m/z): calcd for $C_{17}H_{14}N_2O_2$ $[M+H]^+$: 279.1128, Found: 279.1134.

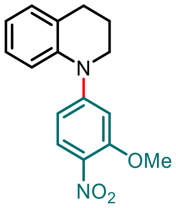

1-(3-methoxy-4-nitrophenyl)-1,2,3,4-tetrahydroquinoline (4a-5)

Prepared according to general procedure A (PE: EtOAc = 50:1, v/v) from **1a-1** (66.6 mg) to afford **4a-5** (92.3 mg, 65% yield) as a yellow liquid. ^{1}H NMR (400 MHz, $CDCl_3$) δ 7.89 (d, J = 9.2 Hz, 1H), 7.17 (t, J = 7.4 Hz, 1H), 7.09 (d, J = 7.6 Hz, 1H), 7.03 (t, J = 7.6 Hz, 1H), 6.90 (t, J = 7.4 Hz, 1H), 6.69-6.64 (m, 2H), 3.80 (s, 3H), 3.63 (t, J = 6.2 Hz, 2H), 2.70 (t, J = 6.2 Hz, 2H), 2.00-1.93 (m, 2H). ^{13}C NMR (101 MHz, $CDCl_3$) δ 155.6, 153.9, 140.8, 131.5, 130.7, 129.3, 128.1, 126.4, 122.7, 120.6, 110.1, 102.3, 56.3, 48.9, 27.2, 24.0. HRMS (m/z): calcd for $C_{16}H_{16}N_2O_3$ $[M+H]^+$: 285.1340, Found: 285.1350.

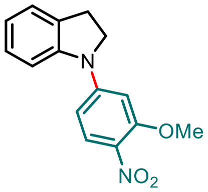

1-(3-methoxy-4-nitrophenyl) indoline (4b-5)

Prepared according to general procedure B (PE: EtOAc = 40:1, v/v) from **1b-1** (59.6 mg) to afford **4b-5** (87.8 mg, 65% yield) as a yellow solid (m.p. 114-116 °C). ^{1}H NMR (400 MHz, $CDCl_3$) δ 7.98 (d, J = 8.8 Hz, 1H), 7.24 (d, J = 8.0 Hz, 1H), 7.17 (d, J = 6.0 Hz, 1H), 7.10 (t, J = 7.8 Hz, 1H), 6.85 (t, J = 7.4 Hz, 1H), 6.71-6.65 (m, 2H), 3.98 (t, J = 8.4 Hz, 2H), 3.90 (s, 3H), 3.12 (t, J = 8.4 Hz, 2H). ^{13}C NMR (101 MHz, $CDCl_3$) δ 156.0, 149.6, 144.3, 132.5, 130.7, 128.6, 127.3, 125.6, 121.6, 110.5, 107.4, 99.1, 56.3, 52.1, 28.0. HRMS (m/z): calcd for $C_{15}H_{14}N_2O_3$ $[M+H]^+$: 271.1077, Found: 271.1084.

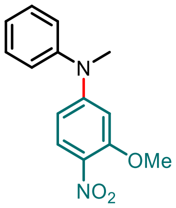

3-methoxy-*N*-methyl-4-nitro-*N*-phenylaniline (4c-5)

Prepared according to general procedure A (PE: EtOAc = 40:1, v/v) from **1c-3** (53.6 mg) to afford **4c-5** (96.8 mg, 75% yield) as a yellow solid (m.p. 107-109 °C) ^{1}H NMR (400 MHz, $CDCl_3$) δ 7.87 (d, J =9.2 Hz, 1H), 7.38 (t, J = 7.8 Hz, 2H), 7.23 (t, J = 7.4 Hz, 1H), 7.16 (d, J = 7.2 Hz, 2H), 6.18 (dd, J = 9.2, 2.4 Hz, 1H), 6.11 (d, J = 2.0 Hz, 1H), 3.75 (s, 3H), 3.33 (s, 3H). ^{13}C NMR (101 MHz, $CDCl_3$) δ 156.2, 154.5, 146.3, 130.1, 129.2, 128.7, 126.8, 126.7, 105.4, 96.7, 56.1, 40.4. HRMS (m/z): calcd for $C_{14}H_{14}N_2O_3$ $[M+H]^+$: 259.1077, Found: 259.1090.

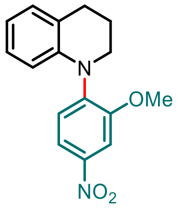

1-(2-methoxy-4-nitrophenyl)-1,2,3,4-tetrahydroquinoline (4a-6)

Prepared according to general procedure A (PE: EtOAc = 50:1, v/v) from **1a-1** (66.6 mg) to afford **4a-6** (106.5 mg, 75% yield) as a yellow solid (m.p. 95-97 °C). ^{1}H NMR (400 MHz, $CDCl_3$) δ 7.85-7.82 (m, 2H), 7.31-7.27 (m, 1H), 7.07 (d, J = 7.2 Hz, 1H), 6.93 (t, J = 7.4 Hz, 1H), 6.77 (t, J = 7.0 Hz, 1H), 6.50 (d, J = 8.4 Hz, 1H), 3.90 (s, 3H), 3.60 (t, J = 5.4 Hz, 2H), 2.89 (t, J = 6.4 Hz, 2H), 2.05-1.97 (m, 2H). ^{13}C NMR (101 MHz, $CDCl_3$) δ 154.3, 144.3, 143.5, 143.0, 129.6, 126.3, 126.2, 124.8, 119.6, 117.1, 116.7, 107.8, 56.0, 50.1, 27.4, 22.1. HRMS (m/z): calcd for $C_{16}H_{16}N_2O_3$ $[M+H]^+$: 285.1234, Found: 285.1234.

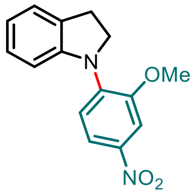

1-(2-methoxy-4-nitrophenyl) indoline (4b-6)

Prepared according to general procedure B (PE: EtOAc = 40:1, v/v) from **1b-1** (59.6 mg) to afford **4b-6** (117.5 mg, 87% yield) as a yellow solid (m.p. 107-109 °C). ^{1}H NMR (400 MHz, $CDCl_3$) δ 7.78 (dd, J = 8.8, 2.4 Hz, 1H), 7.74 (d, J = 2.0 Hz, 1H), 7.33 (d, J

= 8.8 Hz, 1H), 7.12 (d, J = 7.2 Hz, 1H), 7.00 (t, J = 7.6 Hz, 1H), 6.75 (t, J = 7.2 Hz, 1H), 6.63 (d, J = 7.6 Hz, 1H), 3.96 (t, J = 8.4 Hz, 2H), 3.86 (s, 3H), 3.09 (t, J = 8.2 Hz, 2H). ^{13}C NMR (101 MHz, $CDCl_3$) δ 151.8, 146.4, 142.7, 140.6, 131.5, 126.8, 125.0, 120.3, 118.9, 117.4, 111.1, 107.4, 55.8, 53.6, 28.9. HRMS (m/z): calcd for $C_{15}H_{14}N_2O_3$ $[M+H]^+$: 271.1077, Found: 271.1075.

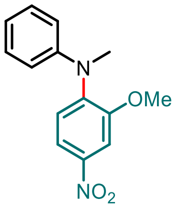

2-methoxy-*N*-methyl-4-nitro-*N*-phenylaniline (4c-6)

Prepared according to general procedure A (PE: EtOAc = 40:1, v/v) from **1c-3** (53.6 mg) to afford **4c-6** (120.0 mg, 93% yield) as a yellow solid (m.p. 80-82 °C). ^{1}H NMR (400 MHz, $CDCl_3$) δ 7.78 (dd, J = 8.8, 2.4 Hz, 1H), 7.71 (d, J = 2.4 Hz, 1H), 7.15 (q, J = 6.4 Hz, 3H), 6.84 (t, J = 7.2 Hz, 1H), 6.77 (d, J = 7.6 Hz, 2H), 3.74 (s, 3H), 3.24 (s, 3H). ^{13}C NMR (101 MHz, $CDCl_3$) δ 153.6, 148.3, 144.1, 143.9, 128.9, 124.5, 120.8, 117.6, 117.3, 107.6, 56.0, 39.8. HRMS (m/z): calcd for $C_{14}H_{14}N_2O_3$ $[M+H]^+$: 259.1077, Found: 259.1079.

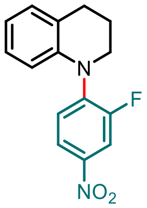

1-(2-fluoro-4-nitrophenyl)-1,2,3,4-tetrahydroquinoline (4a-7)

Prepared according to general procedure A (PE: EtOAc = 40:1, v/v) from **1a-1** (66.6 mg) to afford **4a-7** (98.0 mg, 72% yield) as a yellow solid (m.p. 116-118 °C). ^{1}H NMR (400 MHz, $CDCl_3$) δ 7.95-7.89 (m, 2H), 7.27 (t, J = 8.8 Hz, 1H), 7.04 (d, J = 7.6 Hz, 1H), 6.94 (t, J = 7.4 Hz, 1H), 6.81 (t, J = 7.4 Hz, 1H), 6.65 (d, J = 8.4 Hz, 1H), 3.61 (t, J = 5.4 Hz, 2H), 2.82 (t, J = 6.4 Hz, 2H), 2.01-1.93 (m, 2H). ^{13}C NMR (101 MHz, $CDCl_3$) δ 156.5, 154.0, 142.3 (d, J = 40.0 Hz), 141.6, 129.8, 126.5, 126.4, 124.8 (d, J =12.0 Hz), 121.3, 120.5 (d, J = 12.0 Hz), 117.5, 113.2 (d, J = 100.0 Hz), 50.2, 27.0,

22.6. ^{19}F NMR (376 MHz, $CDCl_3$) δ -114.9 (s, 1F). HRMS (ESI-Orbitrap MS) m/z: Calcd. for $C_{15}H_{13}FN_2O_2$ $[M+H]^+$: 273.10338; Found: 273.10263.

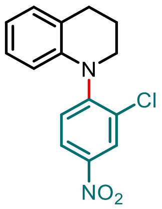

1-(2-chloro-4-nitrophenyl)-1,2,3,4-tetrahydroquinoline (4a-8)

Prepared according to general procedure A (PE: EtOAc = 50:1, v/v) from **1a-1** (66.6 mg) to afford **4a-8** (100.8 mg, 70% yield) as a yellow solid (m.p. 68-70 °C). ^{1}H NMR (400 MHz, $CDCl_3$) δ 8.28 (d, J = 2.8 Hz, 1H), 8.02 (dd, J = 8.8, 2.8 Hz, 1H), 7.33 (d, J = 9.2 Hz, 1H), 7.03 (d, J = 7.6 Hz, 1H), 6.89 (t, J = 7.4 Hz, 1H), 6.75 (t, J = 7.0 Hz, 1H), 6.35 (d, J = 8.0 Hz, 1H), 3.55 (t, J = 5.4 Hz, 2H), 2.84 (t, J = 6.4 Hz, 2H), 2.01-1.94 (m, 2H). ^{13}C NMR (101 MHz, $CDCl_3$) δ 151.7, 144.1, 142.6, 131.7, 129.8, 128.1, 126.9, 126.6, 125.1, 123.1, 120.4, 116.9, 50.6, 27.2, 22.1. HRMS (ESI-Orbitrap MS) m/z: Calcd. for $C_{15}H_{13}ClN_2O_2$ $[M+H]^+$: 289.07383; Found: 289.07036.

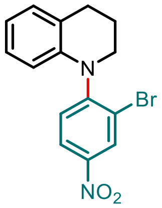

1-(2-bromo-4-nitrophenyl)-1,2,3,4-tetrahydroquinoline (4a-9)

Prepared according to general procedure A (PE: EtOAc = 50:1, v/v) from **1a-1** (66.6 mg) to afford **4a-9** (124.5 mg, 75% yield) as a yellow solid (m.p. 75-77 °C). ^{1}H NMR (400 MHz, $CDCl_3$) δ 8.57 (d, J = 2.4 Hz, 1H), 8.18 (dd, J = 8.8, 2.4 Hz, 1H), 7.42 (d, J = 8.8 Hz, 1H), 7.13 (d, J = 7.6 Hz, 1H), 6.98 (t, J = 7.4 Hz, 1H), 6.84 (t, J = 7.4 Hz, 1H), 6.39 (d, J = 8.4 Hz, 1H), 3.64 (t, J = 5.4 Hz, 2H), 2.94 (t, J = 6.4 Hz, 2H), 2.12-2.06 (m, 2H). ^{13}C NMR (101 MHz, $CDCl_3$) δ 153.4, 144.5, 142.8, 130.0, 129.8, 128.8, 126.6, 124.8, 123.8, 122.0, 120.1, 116.7, 50.8, 27.3, 22.0. HRMS (ESI-Orbitrap MS) m/z: Calcd. for $C_{15}H_{13}BrN_2O_2$ $[M+H]^+$: 333.02332; Found: 333.02249.

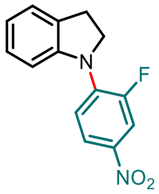

1-(2-fluoro-4-nitrophenyl) indoline (4b-7)

Prepared according to general procedure B (PE: EtOAc = 40:1, v/v) from **1b-1** (59.6 mg) to afford **4b-7** (96.8 mg, 75% yield) as a yellow solid (m.p. 116-118 °C). ^{1}H NMR (400 MHz, $CDCl_3$) δ 7.96-7.91 (m, 2H), 7.40 (t, J = 7.6 Hz, 1H), 7.17 (d, J = 7.2 Hz, 1H), 7.07 (t, J = 7.2 Hz, 1H), 6.84 (t, J = 8.0 Hz, 2H), 4.04 (t, J = 8.2 Hz, 2H), 3.13 (t, J = 7.8 Hz, 2H). ^{13}C NMR (101 MHz, $CDCl_3$) δ 154.0, 151.5, 144.9, 141.1 (d, J = 32.0 Hz), 138.8 (d, J = 44.0 Hz), 131.8, 126.2 (d, J = 712.0 Hz), 121.5, 120.8 (d, J = 8.0 Hz), 118.5 (d, J = 20.0 Hz), 113.2 (d, J = 100.0 Hz), 111.0 (d, J = 16.0 Hz), 53.5, 28.9. ^{19}F NMR (376 MHz, $CDCl_3$) δ -116.8 (s, 1F). HRMS (ESI-Orbitrap MS) m/z: Calcd. for $C_{14}H_{11}FN_2O_2$ $[M+H]^+$: 259.08773; Found: 259.08731.

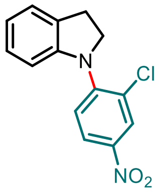

1-(2-chloro-4-nitrophenyl) indoline (4b-8)

Prepared according to general procedure B (PE: EtOAc = 40:1, v/v) from **1b-1** (59.6 mg) to afford **4b-8** (131.6 mg, 96% yield) as a yellow solid (m.p. 115-117 °C). ^{1}H NMR (400 MHz, $CDCl_3$) δ 8.23 (d, J = 2.4 Hz, 1H), 7.97 (dd, J = 9.0, 2.6 Hz, 1H), 7.45 (d, J = 8.8 Hz, 1H), 7.15 (d, J = 7.2 Hz, 1H), 7.02 (t, J = 7.6 Hz, 1H), 6.81 (t, J = 7.2 Hz, 1H), 6.64 (d, J = 8.0 Hz, 1H), 4.0 (t, J = 8.4 Hz, 2H), 3.11 (t, J = 8.2 Hz, 2H). ^{13}C NMR (101 MHz, $CDCl_3$) δ 149.0, 146.0, 142.8, 131.7, 127.5, 127.2, 126.9, 125.3, 123.0, 121.5, 121.3, 111.6, 54.2, 29.1. HRMS (ESI-Orbitrap MS) m/z: Calcd. for $C_{14}H_{11}ClN_2O_2$ $[M+H]^+$: 275.05818; Found: 275.05737.

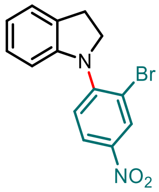

1-(2-bromo-4-nitrophenyl) indoline (4b-9)

Prepared according to general procedure B (PE: EtOAc = 40:1, v/v) from **1b-1** (59.6 mg) to afford **4b-9** (152.7 mg, 96% yield) as a yellow liquid. ^{1}H NMR (400 MHz, $CDCl_3$) δ 8.53 (d, J = 2.4 Hz, 1H), 8.12 (dd, J = 9.0, 2.6 Hz, 1H), 7.52 (d, J = 9.2 Hz, 1H), 7.24 (d, J = 8.0 Hz, 1H), 7.10 (t, J = 7.6 Hz, 1H), 6.89 (t, J = 7.4 Hz, 1H), 6.69 (d, J = 8.0 Hz, 1H), 4.08 (t, J = 8.4 Hz, 2H), 3.20 (t, J = 8.4 Hz, 2H). ^{13}C NMR (101 MHz, $CDCl_3$) δ 150.9, 146.5, 143.5, 131.7, 130.4, 126.9, 125.3, 123.7, 122.6, 121.2, 117.6, 111.6, 54.5, 29.2. HRMS (ESI-Orbitrap MS) m/z: Calcd. for $C_{14}H_{11}BrN_2O_2$ $[M+H]^+$: 319.00767; Found: 319.00699.

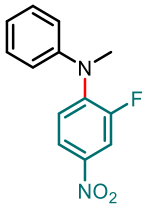

2-fluoro-*N*-methyl-4-nitro-*N*-phenylaniline (4c-7)

Prepared according to general procedure A (PE: EtOAc = 40:1, v/v) from **1c-3** (53.6 mg) to afford **4c-7** (105.8 mg, 86% yield) as a yellow solid (m.p. 57-59 °C). ^{1}H NMR (400 MHz, $CDCl_3$) δ 8.01 (dd, J = 9.0, 1.8 Hz, 1H), 7.95 (dd, J = 12.4, 2.4 Hz, 1H), 7.37 (t, J = 7.8 Hz, 2H), 7.17-7.12 (m, 2H), 7.09 (d, J = 7.6 Hz, 2H), 3.47 (s, 3H). ^{13}C NMR (101 MHz, $CDCl_3$) δ 153.3 (d, J = 1004.0 Hz), 147.5, 143.1 (d, J = 36.0 Hz), 141.0 (d, J = 28.0 Hz), 129.4, 123.9, 121.3, 121.0 (d, J = 12.0 Hz), 120.8 (d, J = 12.0 Hz), 113.0 (d, J = 100.0 Hz), 41.1. ^{19}F NMR (376 MHz, $CDCl_3$) δ -115.9 (s, 1F). HRMS (ESI-Orbitrap MS) m/z: Calcd. for $C_{13}H_{11}FN_2O_2$ $[M+H]^+$: 247.08773; Found: 247.08743.

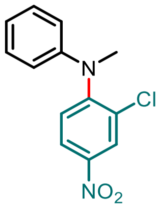

2-chloro-*N*-methyl-4-nitro-*N*-phenylaniline (4c-8)

Prepared according to general procedure A (PE: EtOAc = 40:1, v/v) from **1c-3** (53.6 mg) to afford **4c-8** (98.3 mg, 75% yield) as a yellow solid (m.p. 66-68 °C). ^{1}H NMR (400 MHz, $CDCl_3$) δ 8.23 (d, J = 2.4 Hz, 1H), 8.05 (dd, J = 9.0, 2.6 Hz, 1H), 7.26 (d, J = 8.8 Hz, 1H), 7.22-7.17 (m, 2H), 6.90 (t, J = 7.4 Hz, 1H), 6.76 (d, J = 8.0 Hz, 2H), 3.29 (s, 3H). ^{13}C NMR (101 MHz, $CDCl_3$) δ 152.0, 147.6, 143.7, 130.6, 129.2, 127.0, 126.1, 123.3, 121.6, 118.2, 40.5. HRMS (ESI-Orbitrap MS) m/z: Calcd. for $C_{13}H_{11}ClN_2O_2$ $[M+H]^+$: 236.05818; Found: 236.05746.

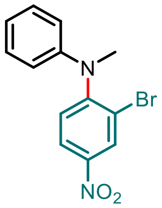

2-bromo-*N*-methyl-4-nitro-*N*-phenylaniline (4c-9)

Prepared according to general procedure A (PE: EtOAc = 40:1, v/v) from **1c-3** (53.6 mg) to afford **4c-9** (119.4 mg, 78% yield) as a yellow liquid. ^{1}H NMR (400 MHz, $CDCl_3$) δ 8.51 (d, J = 2.8 Hz, 1H), 8.18 (dd, J = 8.8, 2.8 Hz, 1H), 7.35 (d, J = 8.8 Hz, 1H), 7.26 (t, J = 8.0 Hz, 2H), 6.95 (t, J = 7.4 Hz, 1H), 6.79 (d, J = 8.0 Hz, 2H), 3.35 (s, 3H). ^{13}C NMR (101 MHz, $CDCl_3$) δ 153.6, 147.6, 144.2, 130.1, 129.2, 127.1, 123.9, 121.2, 121.1, 117.6, 40.4. HRMS (ESI-Orbitrap MS) m/z: Calcd. for $C_{13}H_{11}BrN_2O_2$ $[M+H]^+$: 307.00767; Found: 307.00696.

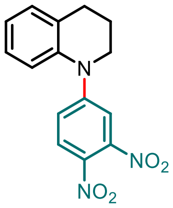

1-(3,4-dinitrophenyl)-1,2,3,4-tetrahydroquinoline (4a-10)

Prepared according to general procedure A (PE: EtOAc = 50:1, v/v) from **1a-1** (66.6 mg) to afford **4a-10** (95.7 mg, 64% yield) as a yellow solid (m.p. 85-87 °C). ^{1}H NMR (400 MHz, $CDCl_3$) δ 7.92 (d, J = 9.2 Hz, 1H), 7.23 (d, J = 2.4 Hz, 1H), 7.21-7.09 (m, 4H), 7.01 (t, J = 7.2 Hz, 1H), 3.64 (t, J = 6.4 Hz, 2H), 2.68 (t, J = 6.0 Hz, 2H), 2.03-1.96 (m, 2H). ^{13}C NMR (101 MHz, $CDCl_3$) δ 152.6, 146.4, 139.1, 132.6, 131.2, 129.4, 127.5, 126.9, 124.7, 121.0, 117.4, 111.5, 48.7, 27.0, 24.3. HRMS (ESI-Orbitrap MS) m/z: Calcd. for $C_{15}H_{13}N_3O_4$ $[M+H]^+$: 300.09788; Found: 300.09702.

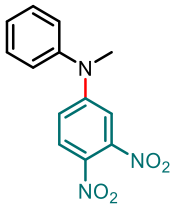

_N_-methyl-3,4-dinitro-_N_-phenylaniline (4c-10)

Prepared according to general procedure A (PE: EtOAc = 40:1, v/v) from **1c-3** (53.6 mg) to afford **4c-10** (71.0 mg, 52% yield) as a yellow solid (m.p. 94-96 °C). ^{1}H NMR (400 MHz, $CDCl_3$) δ 7.88 (d, J = 10.0 Hz, 1H), 7.43 (t, J = 7.8 Hz, 2H), 7.31 (d, J = 7.6 Hz, 1H), 7.17-7.13 (m, 2H), 6.67-6.62 (m, 2H), 3.36 (s, 3H). ^{13}C NMR (101 MHz, $CDCl_3$) δ 153.3, 146.7, 144.9, 130.7, 129.3, 128.0, 127.6, 126.6, 113.2, 107.3, 40.8. HRMS (ESI-Orbitrap MS) m/z: Calcd. for $C_{13}H_{11}N_3O_4$ $[M+Na]^+$: 296.06418; Found: 296.06357.

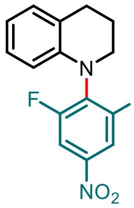

1-(2,6-difluoro-4-nitrophenyl)-1,2,3,4-tetrahydroquinoline (4a-11)

Prepared according to general procedure A (PE: EtOAc = 50:1, v/v) from **1a-1** (66.6 mg) to afford **4a-11** (88.5 mg, 61% yield) as a yellow liquid. ^{1}H NMR (400 MHz, $CDCl_3$) δ 7.81 (d, J = 8.0 Hz, 2H), 7.01 (d, J = 7.2 Hz, 1H), 6.90 (t, J = 7.6 Hz, 1H), 6.74 (t, J = 7.2 Hz, 1H), 6.29 (d, J = 8.0 Hz, 1H), 3.56 (t, J = 5.2 Hz, 2H), 2.85 (t, J = 6.2 Hz, 2H), 2.05-1.98 (m, 2H). ^{13}C NMR (101 MHz, $CDCl_3$) δ 159.7 (d, J = 24.0 Hz),

157.1 (d, J = 24.0 Hz), 141.5, 129.7, 126.7, 123.6, 120.1, 114.4, 108.9(2) (d, J = 112.0 Hz), 108.9(2) (d, J = 48.0 Hz), 50.5, 27.1, 22.5. ^{19}F NMR (376 MHz, $CDCl_3$) δ -111.0 (s, 2F). HRMS (ESI-Orbitrap MS) m/z: Calcd. for $C_{15}H_{12}F_2N_2O_2$ $[M+H]^+$: 291.09396; Found: 291.09332.

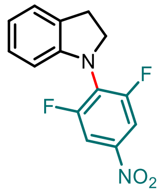

1-(2,6-difluoro-4-nitrophenyl) indoline (4b-11)

Prepared according to general procedure B (PE: EtOAc = 40:1, v/v) from **1b-1** (59.6 mg) to afford **4b-11** (62.1 mg, 45% yield) as a yellow liquid. ^{1}H NMR (400 MHz, $CDCl_3$) δ 7.82 (d, J = 8.8 Hz, 2H), 7.14 (d, J = 7.2 Hz, 1H), 7.04 (d, J = 7.6 Hz, 1H), 6.80 (t, J = 7.2 Hz, 1H), 6.41-6.36 (m, 1H), 4.07 (t, J = 8.4 Hz, 2H), 3.17 (t, J = 8.4 Hz, 2H). ^{13}C NMR (101 MHz, $CDCl_3$) δ 157.4 (d, J = 28.0 Hz,), 154.9, 145.5, 130.1, 127.0, 124.8, 120.7, 109.8, 108.9(2) (d, J = 116.0 Hz), 108.9(2) (d, J = 44.0 Hz), 53.5, 29.2. ^{19}F NMR (376 MHz, $CDCl_3$) δ -110.7 (s, 2F). HRMS (ESI-Orbitrap MS) m/z: Calcd. for $C_{14}H_{10}F_2N_2O_2$ $[M+H]^+$: 277.07831; Found: 277.07785.

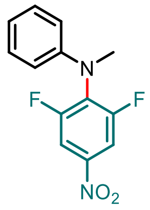

2,6-difluoro-*N*-methyl-4-nitro-*N*-phenylaniline (4c-11)

Prepared according to general procedure A (PE: EtOAc = 40:1, v/v) from **1c-3** (53.6 mg) to afford **4c-11** (76.6 mg, 58% yield) as a yellow liquid. ^{1}H NMR (400 MHz, $CDCl_3$) δ 7.86 (d, J = 8.0 Hz, 2H), 7.26 (t, J = 7.4 Hz, 2H), 6.93 (t, J = 7.2 Hz, 1H), 6.78 (d, J = 8.0 Hz, 2H), 3.37 (s, 3H). ^{13}C NMR (101 MHz, $CDCl_3$) δ 158.2 (dd, J = 254.0, 7.0 Hz), 146.2, 129.1, 120.5, 116.2 (d, J = 616.0, Hz), 114.7, 108.8 (d, J = 29.0 Hz), 108.8 (d, J = 11.0 Hz), 39.1. ^{19}F NMR (376 MHz, CDCl3) δ -111.4 (d, J = 7.5 Hz, 2F). HRMS

(ESI-Orbitrap MS) m/z: Calcd. for $C_{13}H_{10}F_2N_2O_2$ $[M+H]^+$: 265.07831; Found: 265.07779.

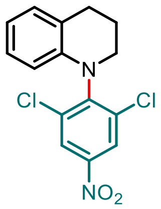

1-(2,6-dichloro-4-nitrophenyl)-1,2,3,4-tetrahydroquinoline (4a-12)

Prepared according to general procedure A (PE: EtOAc = 50:1, v/v) from **1a-1** (66.6 mg) to afford **4a-12** (88.6 mg, 55% yield) as a yellow liquid. ^{1}H NMR (400 MHz, $CDCl_3$) δ 8.22 (s, 2H), 7.00 (d, J = 7.2 Hz, 1H), 6.84 (t, J = 7.6 Hz, 1H), 6.65 (t, J = 7.4 Hz, 1H), 5.86 (d, J = 8.4 Hz, 1H), 3.46 (t, J = 5.4 Hz, 2H), 2.83 (t, J = 6.2 Hz, 2H), 2.09-2.02 (m, 2H). ^{13}C NMR (101 MHz, $CDCl_3$) δ 147.0, 145.6, 141.8, 137.6, 129.6, 127.0, 124.6, 122.3, 118.6, 112.5, 48.7, 27.5, 22.1. HRMS (ESI-Orbitrap MS) m/z: Calcd. for $C_{15}H_{12}Cl_2N_2O_2$ $[M+H]^+$: 323.03486; Found: 323.03436.

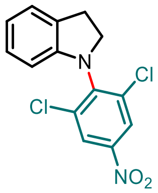

1-(2,6-dichloro-4-nitrophenyl) indoline (4b-12)

Prepared according to general procedure B (PE: EtOAc = 40:1, v/v) from **1b-1** (59.6 mg) to afford **4b-12** (70.9 mg, 46% yield) as a yellow liquid. ^{1}H NMR (400 MHz, $CDCl_3$) δ 8.20 (s, 2H), 7.12 (d, J = 7.2 Hz, 1H), 6.94 (t, J = 7.6 Hz, 1H), 6.70 (t, J = 7.2 Hz, 1H), 6.03 (d, J = 7.6 Hz, 1H), 3.93 (t, J = 8.8 Hz, 2H), 3.22 (t, J = 8.6 Hz, 2H). ^{13}C NMR (101 MHz, $CDCl_3$) δ 147.2, 145.3, 144.0, 136.4, 129.1, 127.1, 125.0, 124.5, 119.3, 108.1, 51.8, 29.2. HRMS (ESI-Orbitrap MS) m/z: Calcd. for $C_{14}H_{10}Cl_2N_2O_2$ $[M+H]^+$: 309.01921; Found: 309.01877.

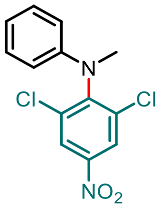

2,6-dichloro-*N*-methyl-4-nitro-*N*-phenylaniline (4c-12)

Prepared according to general procedure A (PE: EtOAc = 40:1, v/v) from **1c-3** (53.6 mg) to afford **4c-12** (80.0 mg, 54% yield) as a yellow liquid. ^{1}H NMR (400 MHz, $CDCl_3$) δ 8.23 (s, 2H), 7.20-7.14 (m, 2H), 6.79 (t, J = 7.4 Hz, 1H), 6.45 (d, J = 8.0 Hz, 2H), 3.21 (s, 3H). ^{13}C NMR (101 MHz, $CDCl_3$) δ 147.7, 146.1, 137.7, 129.3, 124.6, 119.1, 113.1, 112.8, 37.3. HRMS (ESI-Orbitrap MS) m/z: Calcd. for $C_{13}H_{10}Cl_2N_2O_2$ $[M+H]^+$: 297.01921; Found: 297.01871.

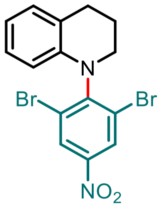

1-(2,6-dibromo-4-nitrophenyl)-1,2,3,4-tetrahydroquinoline (4a-13)

Prepared according to general procedure A (PE: EtOAc = 50:1, v/v) from **1a-1** (66.6 mg) to afford **4a-13** (109.0 mg, 53% yield) as a yellow liquid. ^{1}H NMR (400 MHz, $CDCl_3$) δ 8.45 (s, 2H), 6.99 (d, J = 7.2 Hz, 1H), 6.83 (t, J = 7.6 Hz, 1H), 6.63 (t, J = 7.4 Hz, 1H), 5.81 (d, J = 8.0 Hz, 1H), 3.47 (t, J = 5.6 Hz, 2H), 2.82 (t, J = 6.2 Hz, 2H), 2.12-2.05 (m, 2H). ^{13}C NMR (101MHz, $CDCl_3$) δ 149.5, 146.2, 141.6, 129.5, 128.5, 127.4, 127.0, 122.1, 118.3, 112.2, 48.3, 27.5, 21.9. HRMS (ESI-Orbitrap MS) m/z: Calcd. for $C_{15}H_{12}Br_2N_2O_2$ $[M+H]^+$: 410.93283; Found: 410.93383.

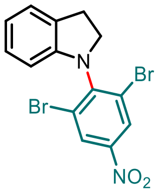

1-(2,6-dibromo-4-nitrophenyl) indoline (4b-13)

Prepared according to general procedure B (PE: EtOAc = 40:1, v/v) from **1b-1** (59.6 mg) to afford **4b-13** (39.7 mg, 20% yield) as a yellow liquid. ^{1}H NMR (400 MHz, $CDCl_3$) δ 8.43 (s, 2H), 7.12 (d, J = 7.2 Hz, 1H), 6.94 (d, J = 7.4 Hz, 1H), 6.69 (t, J = 7.6 Hz, 1H), 5.99 (d, J =7.6 Hz, 1H), 3.92 (t, J = 8.8 Hz, 2H), 3.24 (t, J = 8.6 Hz, 2H). ^{13}C NMR (101 MHz, $CDCl_3$) δ 147.4, 146.7, 146.1, 129.0, 128.3, 127.2, 126.5, 125.0, 119.0, 107.6, 51.3, 29.1. HRMS (ESI-Orbitrap MS) m/z: Calcd. for $C_{14}H_{10}Br_2N_2O_2$ $[M+H]^+$: 396.91742; Found: 396.91818.

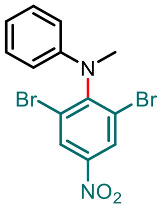

2,6-dibromo-*N*-methyl-4-nitro-*N*-phenylaniline (4c-13)

Prepared according to general procedure A (PE: EtOAc = 40:1, v/v) from **1c-3** (53.6 mg) to afford **4c-13** (100.1 mg, 52% yield) as a yellow liquid. ^{1}H NMR (400 MHz, $CDCl_3$) δ 8.44 (s, 2H), 7.19-7.14 (m, 2H), 6.78 (t, J = 7.4 Hz, 1H), 6.41 (d, J = 8.4 Hz, 2H), 3.18 (s, 3H). ^{13}C NMR (101 MHz, $CDCl_3$) δ 150.3, 145.8, 129.4, 129.2, 128.4, 127.4, 118.9, 112.5, 36.9. HRMS (ESI-Orbitrap MS) m/z: Calcd. for $C_{13}H_{10}Br_2N_2O_2$ $[M+H]^+$: 384.91757 Found: 384.91818.

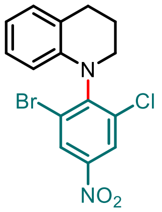

1-(2-bromo-6-chloro-4-nitrophenyl)-1,2,3,4-tetrahydroquinoline(4a-14)

Prepared according to general procedure A (PE: EtOAc = 50:1, v/v) from **1a-1** (66.6 mg) to afford **4a-14** (85.8 mg, 47% yield) as a yellow liquid. ^{1}H NMR (400 MHz, $CDCl_3$) δ 8.39 (d, J = 2.8 Hz, 1H), 8.26 (d, J = 2.4 Hz, 1H), 6.98 (d, J = 7.6 Hz, 1H), 6.82 (t, J = 7.6 Hz, 1H), 6.63 (t, J = 7.2 Hz, 1H), 5.83 (d, J = 8.0 Hz, 1H), 3.45 (t, J = 5.4 Hz, 2H), 2.82 (t, J = 6.2 Hz, 2H), 2.09-2.03 (m, 2H). ^{13}C NMR (101 MHz, $CDCl_3$) δ 148.3, 145.9, 141.7, 137.6, 129.5, 127.7, 127.4, 127.0, 125.3., 122.2, 118.4, 112.3,

48.5, 27.5, 22.0. HRMS (ESI-Orbitrap MS) m/z: Calcd. for $C_{15}H_{12}ClBrN_2O_2$ $[M+H]^+$: 366.98434; Found: 366.98061.

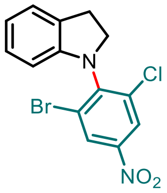

1-(2-bromo-6-chloro-4-nitrophenyl) indoline (4b-14)

Prepared according to general procedure B (PE: EtOAc = 40:1, v/v) from **1b-1** (59.6 mg) to afford **4b-14** (89.6 mg, 51% yield) as a yellow liquid. ^1H NMR (400 MHz, $CDCl_3$) δ 8.36 (d, J = 2.4 Hz, 1H), 8.22 (d, J = 2.4 Hz, 1H), 7.09 (d, J = 7.2 Hz, 1H), 6.91 (t, J = 7.6 Hz, 1H), 6.67 (t, J = 7.0 Hz, 1H), 5.99 (d, J = 8.0 Hz, 1H), 3.97-3.83 (m, 2H), 3.21 (t, J = 8.6 Hz, 2H). ^{13}C NMR (101 MHz, $CDCl_3$) δ 147.3, 145.7, 145.3, 136.7, 129.0, 127.6, 127.1, 126.3, 125.2, 125.0, 119.1, 107.7, 51.5, 29.1. HRMS (ESI-Orbitrap MS) m/z: Calcd. for $C_{14}H_{10}ClBrN_2O_2$ $[M+H]^+$: 352.96869; Found: 352.96814.

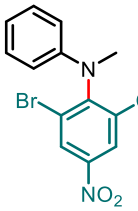

2-bromo-6-chloro-*N*-methyl-4-nitro-*N*-phenylaniline (4c-14)

Prepared according to general procedure A (PE: EtOAc = 40:1, v/v) from **1c-3** (53.6 mg) to afford **4c-14** (84.8 mg, 50% yield) as a yellow liquid. ^1H NMR (400 MHz, $CDCl_3$) δ 8.40 (d, J = 2.4 Hz, 1H), 8.26 (d, J = 2.4 Hz, 1H), 7.18-7.13 (m, 2H), 6.77 (t, J = 7.2 Hz, 1H), 6.42 (d, J = 8.0 Hz, 2H), 3.19 (s, 3H). ^{13}C NMR (101 MHz, $CDCl_3$) δ 149.0, 146.0, 137.7, 129.3, 129.2, 127.7, 127.5, 125.2, 119.0, 112.6, 37.0. HRMS (ESI-Orbitrap MS) m/z: Calcd. for $C_{13}H_{10}ClBrN_2O_2$ $[M+H]^+$: 340.96869; Found: 340.96811.

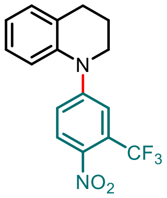

1-(4-nitro-3-(trifluoromethyl)phenyl)-1,2,3,4-tetrahydroquinoline (4a-15)

Prepared according to general procedure A (PE: EtOAc = 40:1, v/v) from **1a-1** (66.6 mg) to afford **4a-15** (98.2 mg, 61% yield) as a yellow solid (m.p. 98-100 °C). ^{1}H NMR (400 MHz, $CDCl_3$) δ 7.98 (d, J = 9.2 Hz, 1H), 7.52 (s, 1H), 7.30 (d, J = 9.2 Hz, 1H), 7.22 - 7.11 (m, 2H), 7.14 (t, J = 7.6 Hz, 1H), 7.03 (t, J = 7.2 Hz, 1H), 3.72 (t, J = 6.2 Hz, 2H), 2.77 (t, J = 6.2 Hz, 2H), 2.10 - 2.03 (m, 2H). ^{13}C NMR (101 MHz, $CDCl_3$) δ 151.8, 139.9, 138.7, 131.4, 129.4, 128.0, 126.7, 125.8 (q, J = 33.0 Hz), 123.6, 122.2 (d, J = 272.0 Hz), 120.3, 119.2, 116.4 (q, J = 6.0 Hz), 48.8, 27.1, 24.1. ^{19}F NMR (376 MHz, CDCl3) δ -60.2 (s, 3F). HRMS (ESI-Orbitrap MS) m/z: Calcd. for $C_{16}H_{13}F_3N_2O_2$ $[M+H]^+$: 323.1007; Found: 323.0996.

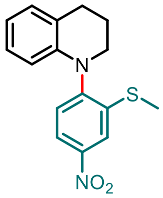

1-(2-(methylthio)-4-nitrophenyl)-1,2,3,4-tetrahydroquinoline (4a-16)

Prepared according to general procedure A (PE: EtOAc = 40:1, v/v) from **1a-1** (66.6 mg) to afford **4a-15** (126.0 mg, 84% yield) as a yellow solid (m.p. 121-123 °C). ^{1}H NMR (400 MHz, $CDCl_3$) δ 8.08 (s, 1H), 7.99 (d, J = 8.4 Hz, 1H), 7.31 (d, J = 8.4 Hz, 1H), 7.07 (d, J = 7.2 Hz, 1H), 6.92 (t, J = 7.6 Hz, 1H), 6.75 (t, J = 7.2 Hz, 1H), 6.22 (t, J = 8.0 Hz, 1H), 3.52 (t, J = 3.8 Hz, 2H), 2.91 (t, J = 6.6 Hz, 2H), 2.12 - 2.05 (m, 2H). ^{13}C NMR (101 MHz, $CDCl_3$) δ 150.8, 145.8, 143.3, 141.0, 129.6, 128.0, 126.6, 124.1, 120.7, 120.2, 119.2, 115.6, 50.1, 27.5, 22.2, 14.6. HRMS (ESI-Orbitrap MS) m/z: Calcd. for $C_{16}H_{16}N_2O_2S$ $[M+H]^+$: 301.1011; Found: 301.1006.

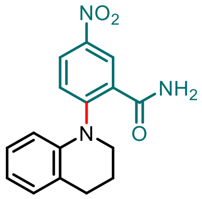

2-(3,4-dihydroquinolin-1(2H)-yl)-5-nitrobenzamide (4a-17)

Prepared according to general procedure A (PE: EtOAc = 5:1, v/v) from **1a-1** (26.6 mg) to afford **4a-17** (27.5 mg, 52% yield) as a red liquid. ^{1}H NMR (400 MHz, $CDCl_3$) δ 8.93 (s, 1H), 8.22 (d, J = 8.8 Hz, 1H), 7.76 (s, J = 8.0 Hz, 1H), 7.37 (d, J = 8.8 Hz, 1H), 7.14 (d, J = 7.2 Hz, 1H), 7.00 (t, J = 7.8 Hz, 1H), 6.91 (t, J = 7.4 Hz, 1H), 6.59 (d, J = 8.4 Hz, 1H), 6.44 (s, 1H), 3.55 (t, J = 5.2 Hz, 2H), 2.93 (t, J = 6.6 Hz, 2H), 2.08 - 2.00 (m, 2H). ^{13}C NMR (100 MHz, $CDCl_3$) δ 166.3, 154.0, 144.3, 142.7, 130.6, 130.2, 127.8, 127.6, 127.0, 127.0, 126.6, 121.9, 118.8, 53.2, 27.0, 21.6. HRMS (ESI-Orbitrap MS) m/z: Calcd. for $C_{16}H_{15}N_3O_3$ $[M+H]^+$: 298.1192; Found: 298.1186.

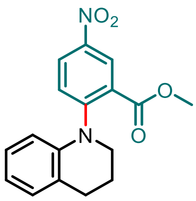

methyl 2-(3,4-dihydroquinolin-1(2*H*)-yl)-5-nitrobenzoate (4a-18)

Prepared according to general procedure A (PE: EtOAc = 30:1, v/v) from **1a-1** (26.6 mg) to afford **4a-18** (40.0 mg, 64% yield) as a yellow solid (m.p. 165-167°C). ^{1}H NMR (400 MHz, $CDCl_3$) 8.57 (d, J = 2.8 Hz, 1H), 8.24 (dd, J = 9.2, 2.8 Hz, 1H), 7.36 (d, J = 9.2 Hz, 1H), 7.12 (d, J = 7.2 Hz, 1H), 6.97 (t, J = 7.6 Hz, 1H), 6.87 (t, J = 7.4 Hz, 1H), 6.74 (d, J = 8.0 Hz, 1H), 3.67 (t, J = 5.8 Hz, 2H), 3.57 (s, 3H), 2.86 (t, J = 6.6 Hz, 2H), 2.20 - 2.00 (m, 2H). ^{13}C NMR (100 MHz, $CDCl_3$) δ 166.0, 153.0, 142.5, 141.2, 129.5, 127.9, 127.7, 127.5, 126.6, 124.9, 124.5, 121.9, 117.2, 52.2, 51.04, 27.0, 23.0. HRMS (ESI-Orbitrap MS) m/z: Calcd. for $C_{17}H_{16}N_2O_4$ $[M+H]^+$: 313.1188; Found: 313.1184.

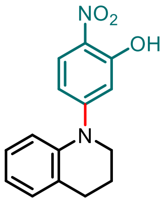

5-(3,4-dihydroquinolin-1(2H)-yl)-2-nitrophenol (4a-20)

Prepared according to general procedure A (PE: EtOAc = 10:1, v/v) from **1a-1** (26.6 mg) to afford **4a-20** (31.7 mg, 51% yield) as a red solid (m.p. 106 - 108°C). ^{1}H NMR (400 MHz, $CDCl_3$) δ 11.18 (s, 1H), 7.85 (d, J = 9.6 Hz, 1H), 7.24 (d, J = 8.0 Hz, 1H),

7.14 (m, 2H), 7.02 (t, *J* = 7.4 Hz, 1H), 6.74 (dd, *J* = 9.6, 2.4 Hz, 1H), 6.61 (d, *J* = 4.0 Hz, 1H), 3.67 (t, *J* = 6.0 Hz, 2H), 2.71 (t, *J* = 8.0 Hz, 2H), 2.05 - 1.98 (m, 2H). ^{13}C NMR (100 MHz, $CDCl_3$) δ 157.5, 155.3, 139.5, 132.3, 128.8, 126.3, 126.1, 125.8, 123.8, 122.0, 110.4, 103.3, 77.3, 77.0, 76.7, 48.1, 26.9, 24.3. HRMS (ESI-Orbitrap MS) m/z: Calcd. for $C_{15}H_{14}N_2O_3$ $[M+H]^+$: 271.1083; Found: 271.1073.

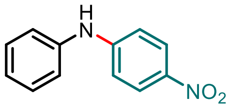

4-nitro-*N*-phenylaniline (6g)

Prepared according to general procedure A from **5g** (66.6 mg) to afford **6g** (30.0 mg, 28 % yield) as a yellow solid (m.p. 115-117 °C). ^{1}H NMR (400 MHz, $CDCl_3$), δ 8.12 (d, *J* = 9.2 Hz, 2H), 7.39 (t, *J* = 7.8 Hz, 2H), 7.23-7.15 (m, 3H), 6.94 (d, *J* = 9.2 Hz, 2H), 6.30 (s, 1H). ^{13}C NMR (101 MHz, $CDCl_3$) δ 150.2, 139.8, 139.5, 129.7, 126.2 124.7, 121.9, 113.7. HRMS (m/z): calcd for $C_{15}H_{14}N_2O_2$ $[M+H]^+$: 255.1128, Found: 255.1121.

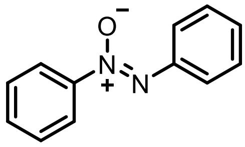

(*Z*)-1,2-diphenyldiazene 1-oxide (6e) [41]

Prepared according to general procedure A to give **6e** (36.6 mg, 37% yield) as a yellow solid. ^{1}H NMR (400 MHz, $CDCl_3$) δ 8.34 (d, *J* = 8.0 Hz, 2H), 8.22 (d, *J* = 8.0 Hz, 2H), 7.58-7.49 (m, 5H), 7.44-7.39 (m, 1H). ^{13}C NMR (101 MHz, $CDCl_3$) δ 148.2, 143.9, 131.5, 129.5, 128.7, 128.6, 125.4, 122.2.

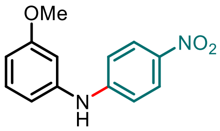

3-methoxy-*N*-(4-nitrophenyl)aniline (6i) [42]

Prepared according to general procedure A (PE: EtOAc = 20:1, v/v) from **5i** (82.0 mg) to afford **6i** (42.7 mg, 35% yield) as a yellow solid (m.p. 140-142°C). ^{1}H NMR (400

MHz, $CDCl_3$) δ 8.12 (d, J = 8.8 Hz, 2H), 7.29 (t, J = 8.2 Hz, 1H), 6.97 (d, J = 9.2 Hz, 2H), 6.80 (d, J = 8.0 Hz, 1H), 6.75 (s, 1H), 6.71 (d, J = 8.4 Hz, 1H), 6.28 (s, 1H), 3.82 (s, 3H). ^{13}C NMR (101 MHz, $CDCl_3$) δ 160.7, 149.9, 140.8, 139.8, 130.5, 126.2, 114.0, 113.9, 109.7, 107.6, 55.4.

9. X-Ray Ellipsoid Plots of 3a-6, 3b-1, 4a-7, 4b-8, 4b-9 and 4c-10.

Single crystal structure of 3a-6:

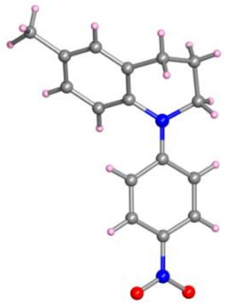

Supplementary Table 19: Crystal data of compound **3a-6** at room temperature

Compounds	6-methyl-1-(4-nitrophenyl)-1,2,3,4-tetrahydroquinoline
CCDC Name	CCDC 2105132
Chemical Formula	$C_{16}H_{16}N_2O_2$
Formula Weight	268.31
Temperature(K)	298
Crystal System	Monoclinic
Space Group	*C2/c*
a (Å)	14.3184(19)
b (Å)	8.1538(11)
c (Å)	24.031(4)
α (°)	90.00
β (°)	102.795(2)
γ (°)	90.00
Volume[Å]^3	2735.9(7)
Z	8
D_{calc} (g/cm^3)	1.303
F(000)	1136.0
GOF, *S*	1.013
R_1, wR_2 (obsd data)	0.0460, 0.1229
R_1, wR_2 (all data)	0.0705, 0.1388

Single crystal structure of 3b-1:

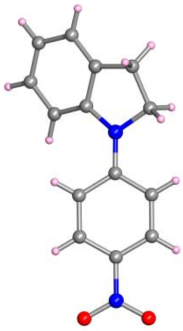

Supplementary Table 20: Crystal data of compound **3b-1** at room temperature

Compounds	1-(4-nitrophenyl)indoline
CCDC Name	CCDC 2105448
Chemical Formula	$C_{14}H_{12}N_2O_2$
Formula Weight	240.26
Temperature(K)	298
Crystal System	Monoclinic
Space Group	*P2(1)*
a (Å)	7.7886(11)
b (Å)	7.0307(10)
c (Å)	10.5845(15)
α (°)	90.00
β (°)	91.678(2)
γ (°)	90.00
Volume[Å]^3	579.35(14)
Z	2
D_{calc} (g/cm^3)	1.377
F(000)	252
GOF, *S*	6.029
R_1, wR_2 (obsd data)	0.2884, 0.6194
R_1, wR_2 (all data)	0.3072, 0.6433

Single crystal structure of 4a-7:

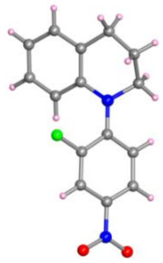

Supplementary Table 21: Crystal data of compound **4a-7** at room temperature

Compounds	1-(2-fluoro-4-nitrophenyl)-1,2,3,4-tetrahydroquinoline
CCDC Name	CCDC 2176176
Chemical Formula	$C_{15}H_{13}FN_2O_2$
Formula Weight	272.27
Temperature(K)	293
Crystal System	Monoclinic
Space Group	*C2/c*
a (Å)	7.7653(6)
b (Å)	12.6284(11)
c (Å)	26.454(2)
α (°)	90.00
β (°)	93.382(8)
γ (°)	90.00
Volume[Å]^3	2589.7(4)
Z	8
D_{calc} (g/cm^3)	1.397
F(000)	1136.0
GOF, *S*	1.158
R_1, wR_2 (obsd data)	0.0813, 0.2409
R_1, wR_2 (all data)	0.0982, 0.2533

Alert level A: This is related to the data set quality since the crystal was poorly diffracting at high angle. Therefore, the completeness of the structure is of 93%.

Single crystal structure of 4b-8:

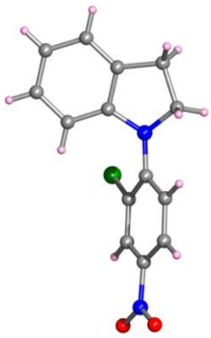

Supplementary Table 22: Crystal data of compound **4b-8** at room temperature

Compounds	1-(2-chloro-4-nitrophenyl)indoline
CCDC Name	CCDC 2175175
Chemical Formula	$C_{14}H_{11}ClN_2O_2$
Formula Weight	274.7
Temperature(K)	293
Crystal System	triclinic
Space Group	P-1
a (Å)	7.31690(10)
b (Å)	7.71830(10)
c (Å)	11.5561(2)
α (°)	99.4160(10)
β (°)	103.1720(10)
γ (°)	97.4940(10)
Volume[Å]^3	617.260(16)
Z	2
D_{calc} (g/cm^3)	1.4779
F(000)	284.0
GOF, *S*	1.052
R_1, wR_2 (obsd data)	0.0395, 0.0185
R_1, wR_2 (all data)	0.0402, 0.1093

Single crystal structure of 4b-9:

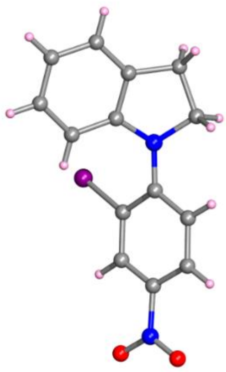

Supplementary Table 23: Crystal data of compound **4b-9** at room temperature

Compounds	1-(2-bromo-4-nitrophenyl)indoline
CCDC Name	CCDC 2175172
Chemical Formula	$C_{14}H_{11}BrN_2O_2$
Formula Weight	219.16
Temperature(K)	298.4
Crystal System	triclinic
Space Group	P-1
a (Å)	7.3715(3)
b (Å)	7.8209(3)
c (Å)	11.5556(4)
α (°)	99.529(3)
β (°)	103.815(3)
γ (°)	97.169(3)
Volume[Å]^3	628.54(4)
Z	2
D_{calc} (g/cm^3)	1.686
F(000)	320.0
GOF, *S*	1.056
R_1, wR_2 (obsd data)	0.0387, 0.1082
R_1, wR_2 (all data)	0.0395, 0.1092

Single crystal structure of 4c-10:

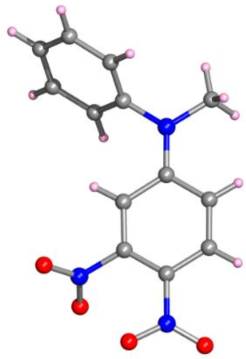

Supplementary Table 24: Crystal data of compound 4c-10 at room temperature

Compounds	1-(2-chloro-4-nitrophenyl)indoline
CCDC Name	CCDC 2181802
Chemical Formula	$C_{13}H_{11}N_3O_4$
Formula Weight	273.25
Temperature(K)	293(2)
Crystal System	monoclinic
Space Group	$P2_1/c$
a (Å)	7.95550(10)
b (Å)	16.4277(4)
c (Å)	19.5416(4)
α (°)	90
β (°)	94.261(2)
γ (°)	90
Volume[Å]^3	2546.84(9)
Z	8
D_{calc} (g/cm^3)	1.425
F(000)	1136.0
GOF, *S*	1.111
R_1, wR_2 (obsd data)	0.0582, 0.1646
R_1, wR_2 (all data)	0.0673, 0.1736

10. NMR spectra of substrates and products

6-ethoxyquinoline

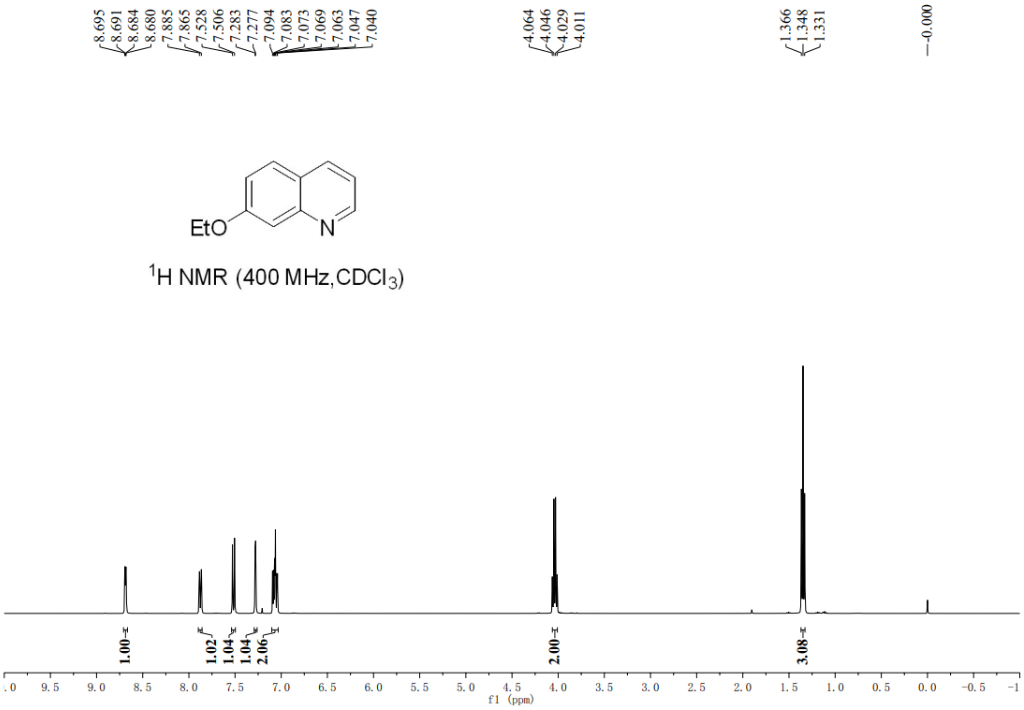

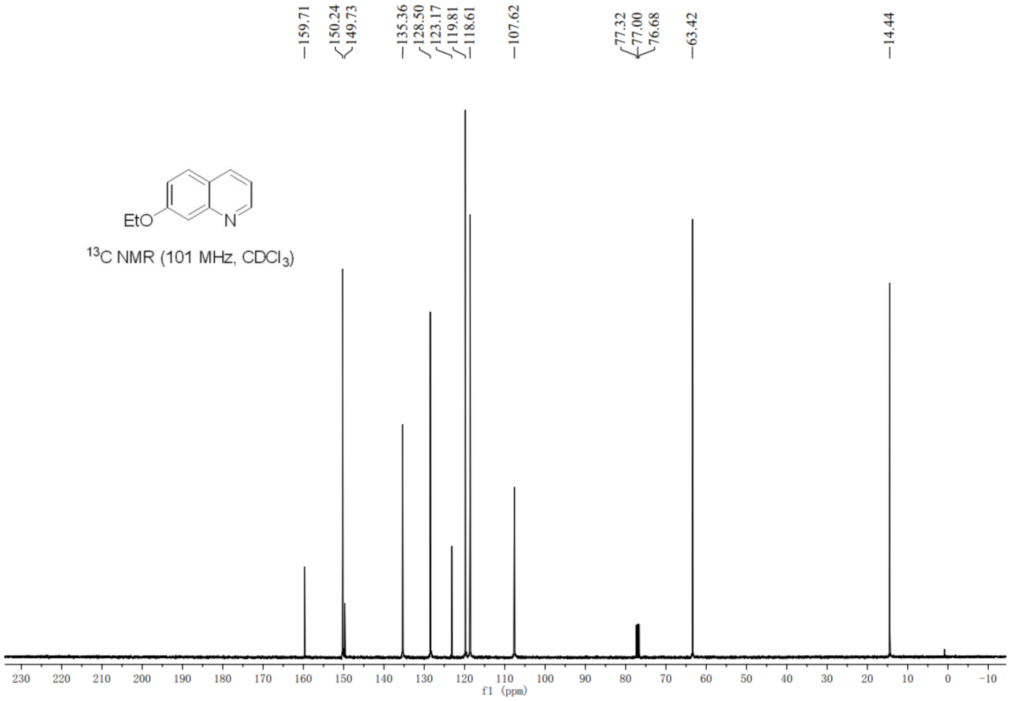

3-methyl-1,2,3,4-tetrahydroquinoline (1a-3)

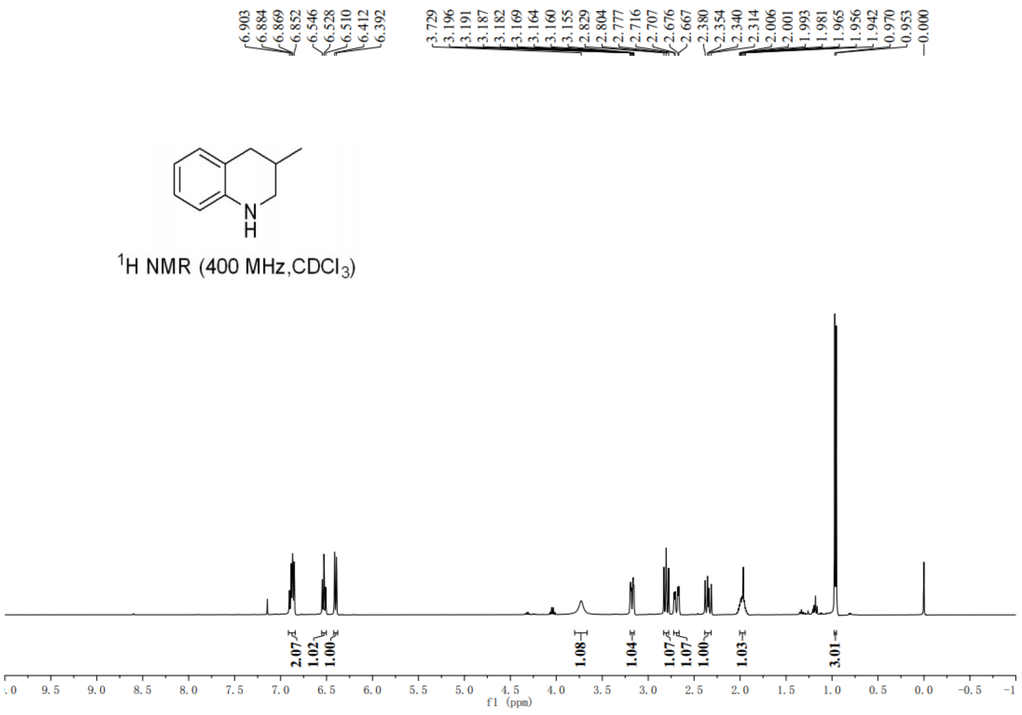

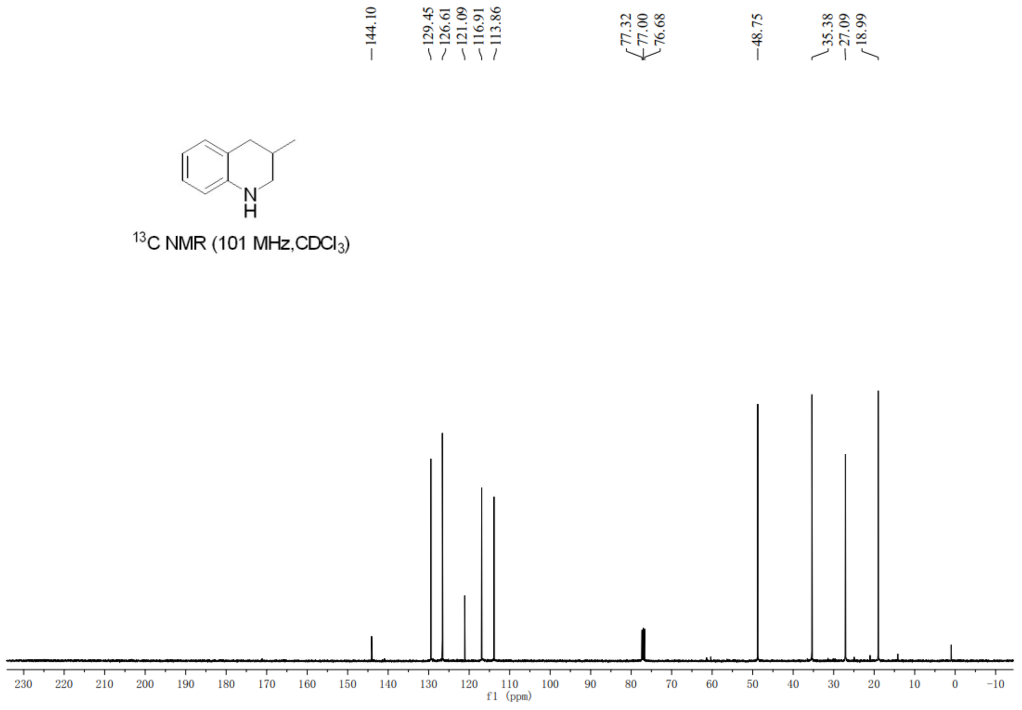

6-isopropoxy-1,2,3,4-tetrahydroquinoline (1a-10)

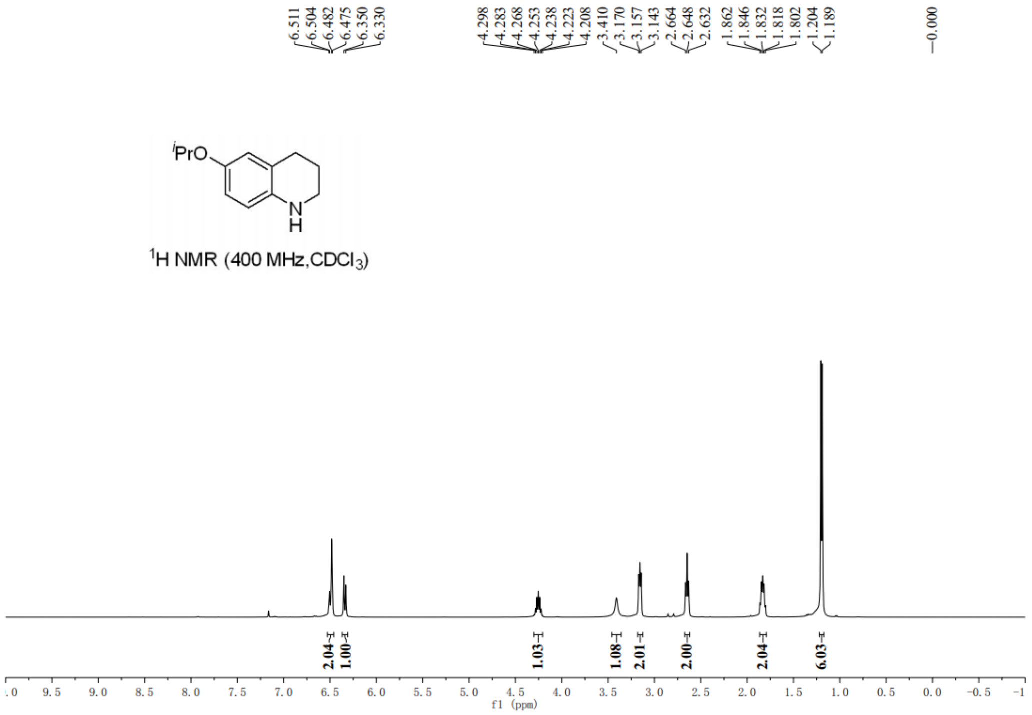

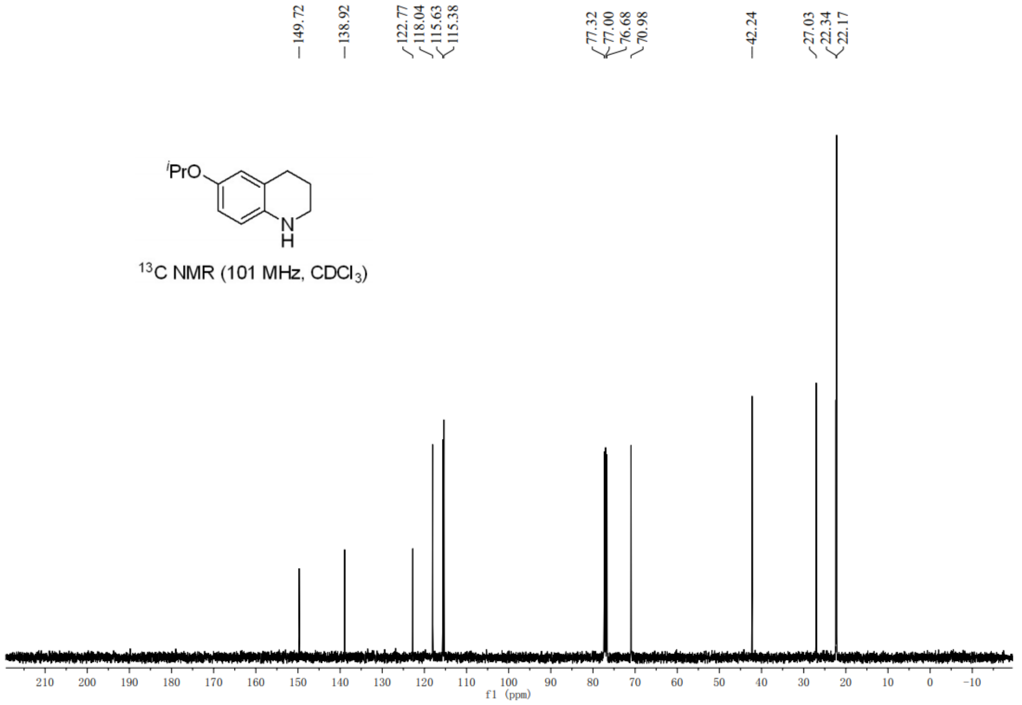

6-ethoxy-1,2,3,4-tetrahydroquinoline (1a-11)

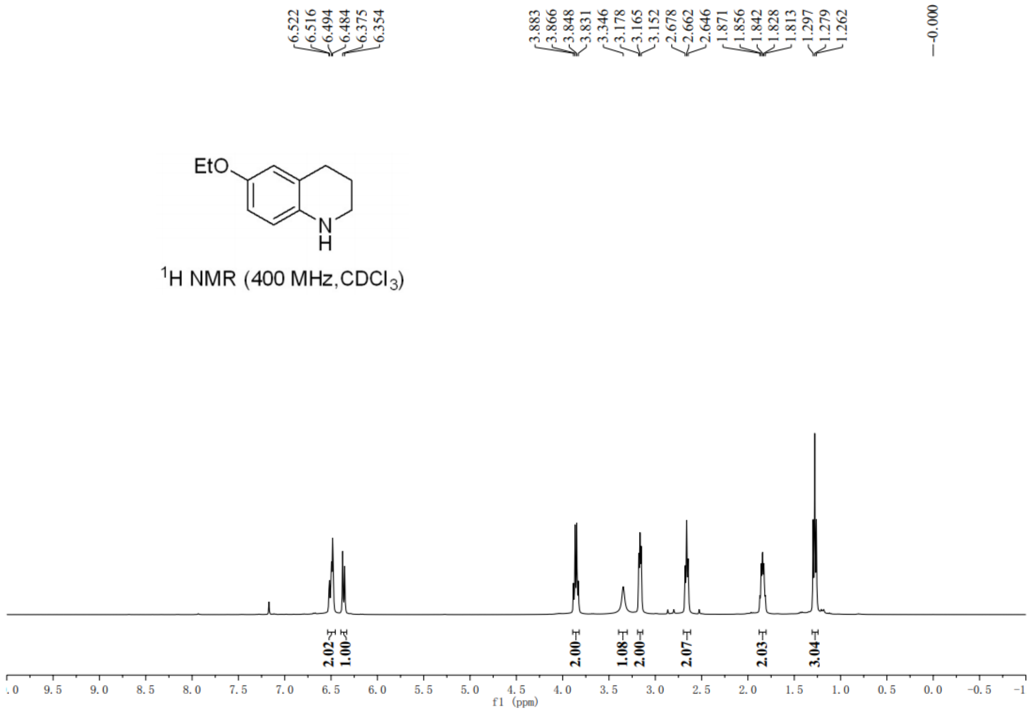

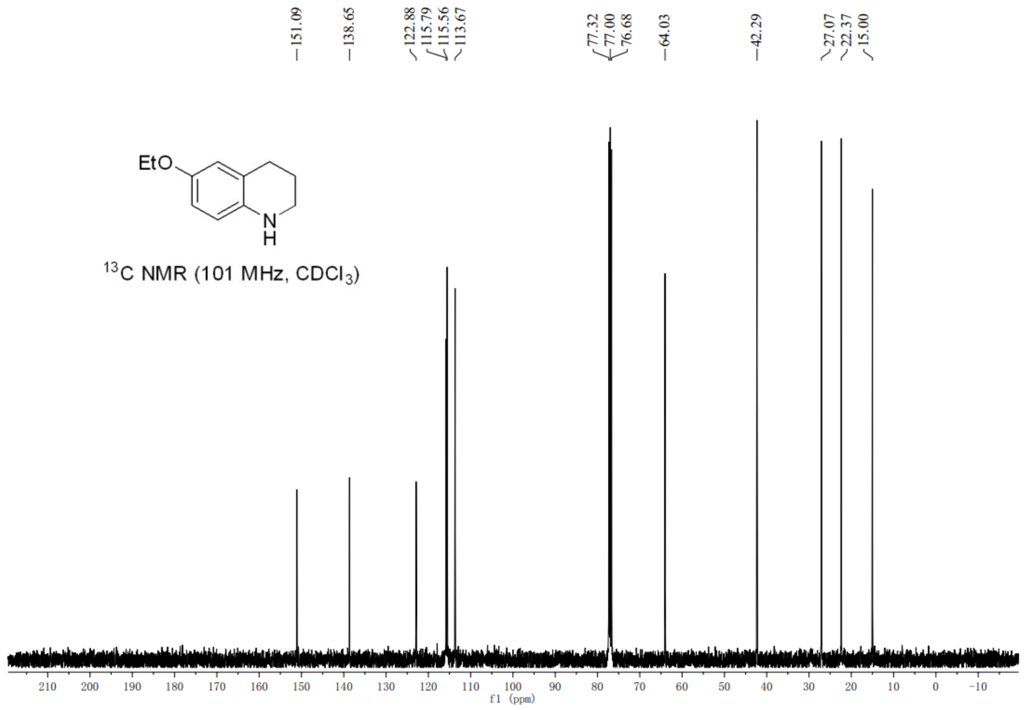

7-ethoxy-1,2,3,4-tetrahydroquinoline (1a-13)

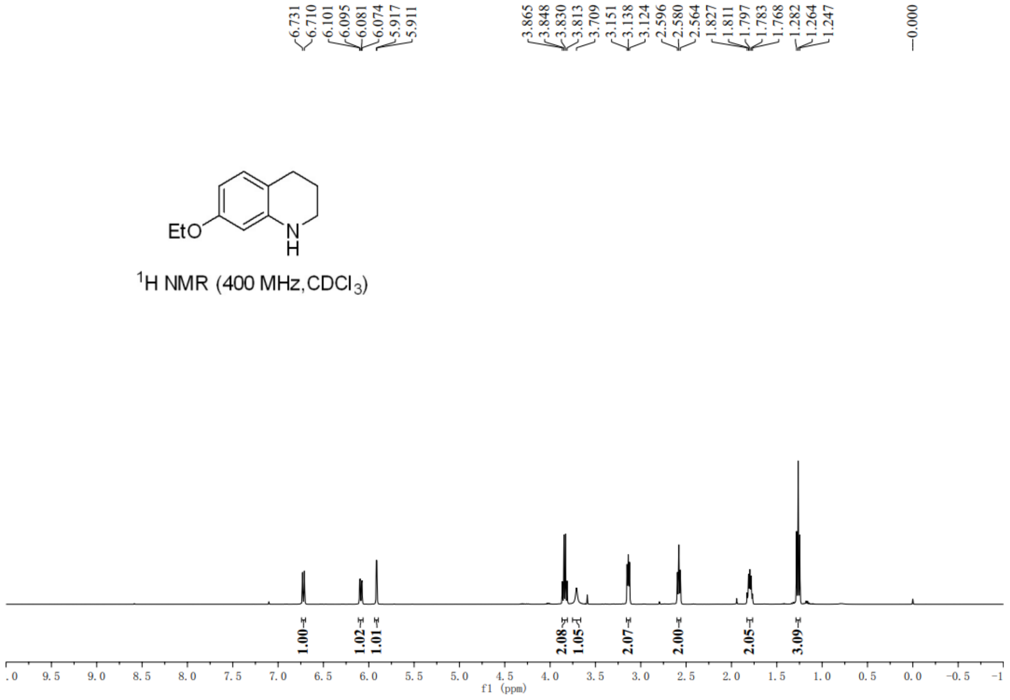

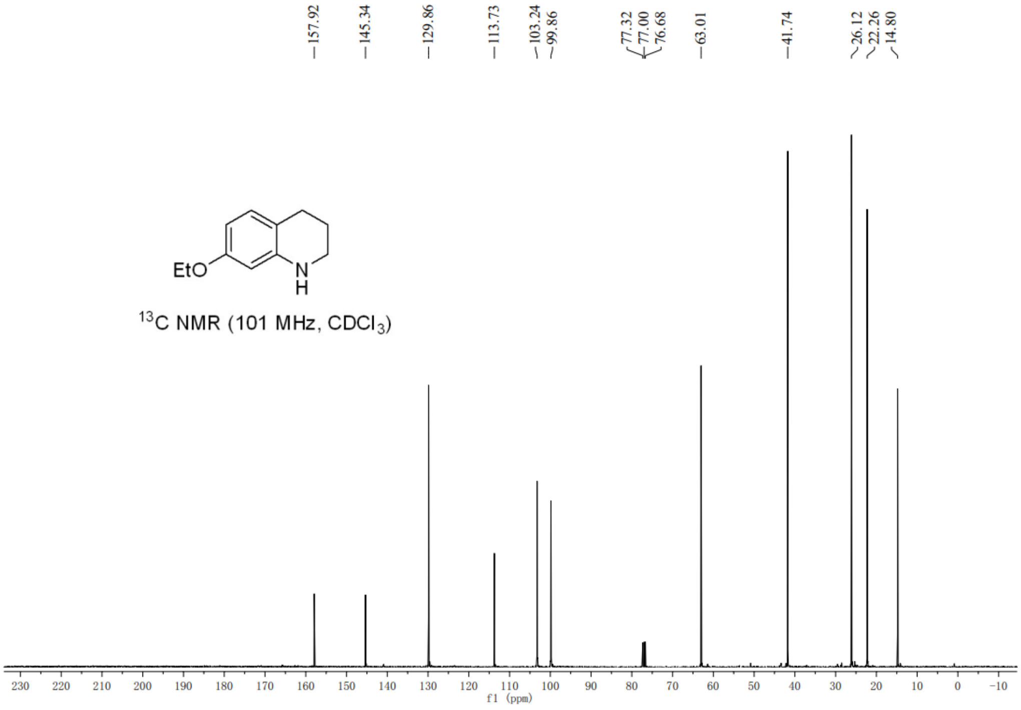

7-isopropoxy-1,2,3,4-tetrahydroquinoline (1a-14)

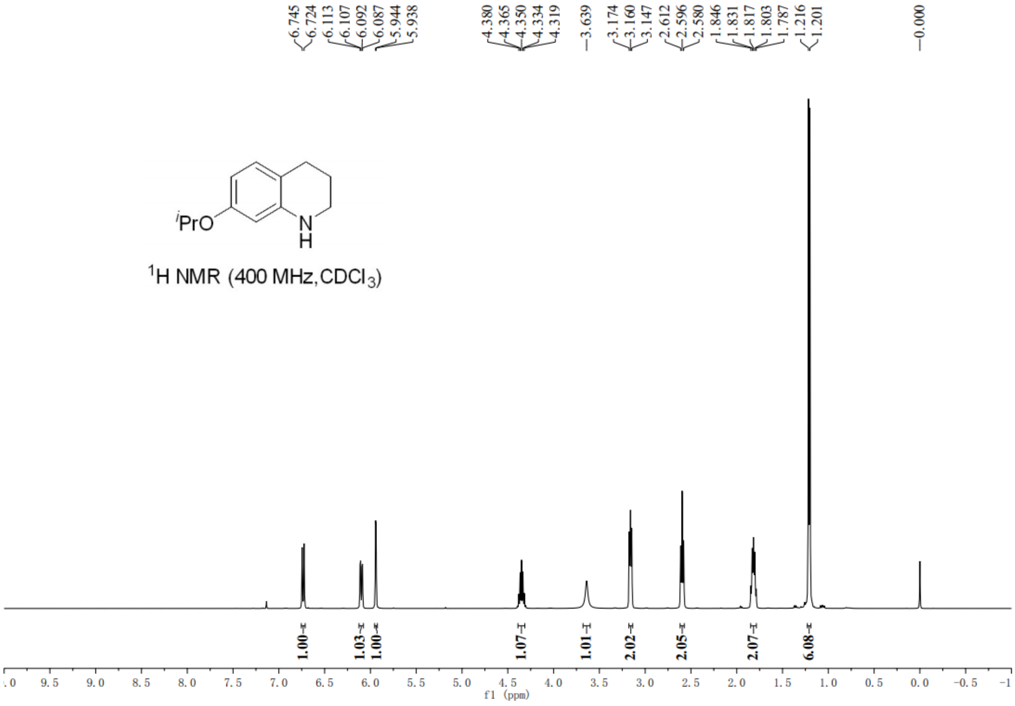

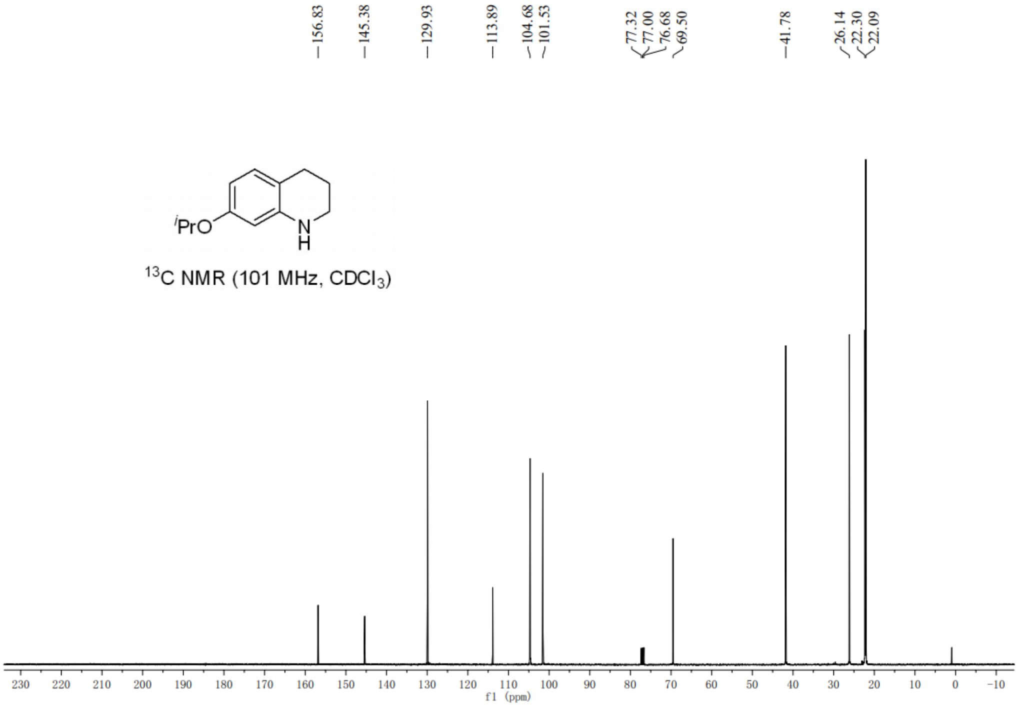

5-chloro-1,2,3,4-tetrahydroquinoline (1a-18)

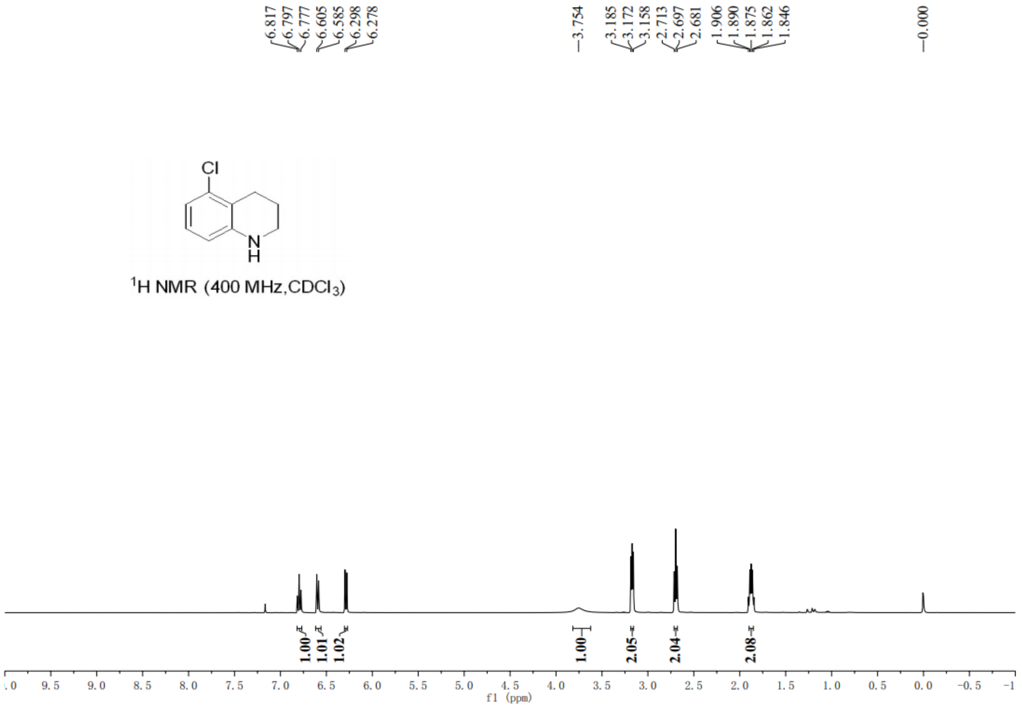

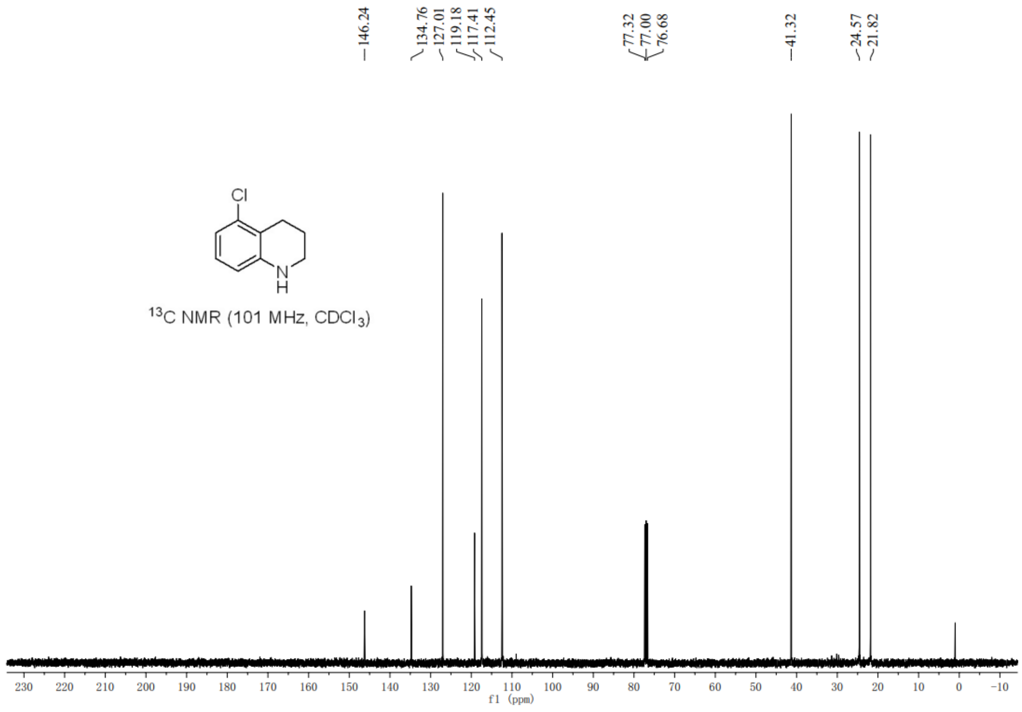

5-ethoxyindoline (1b-12)

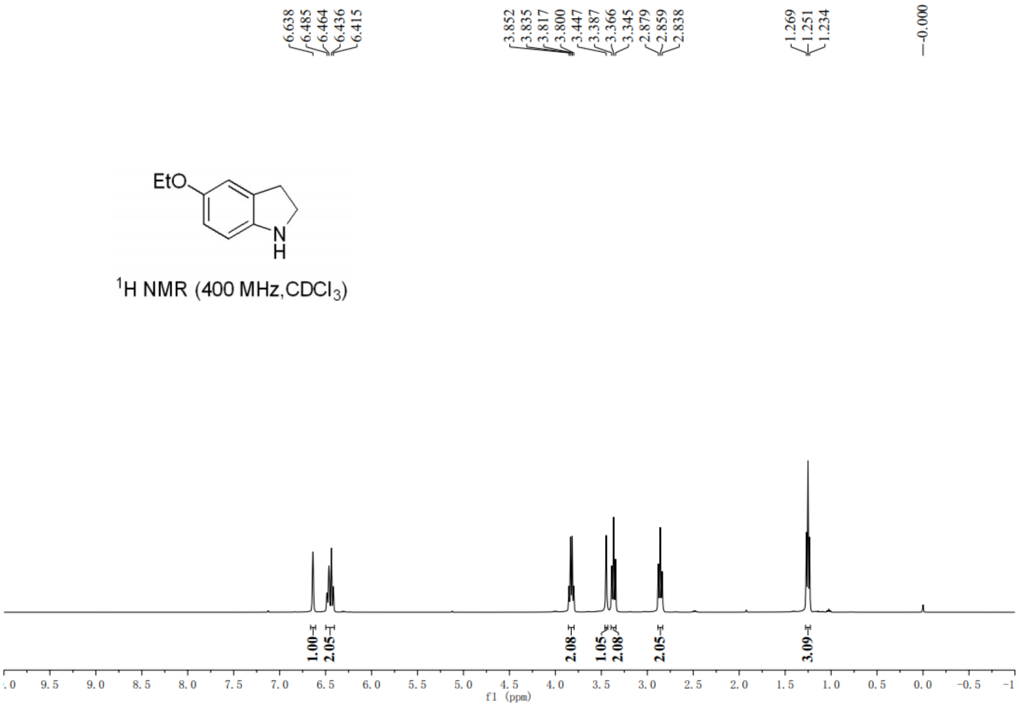

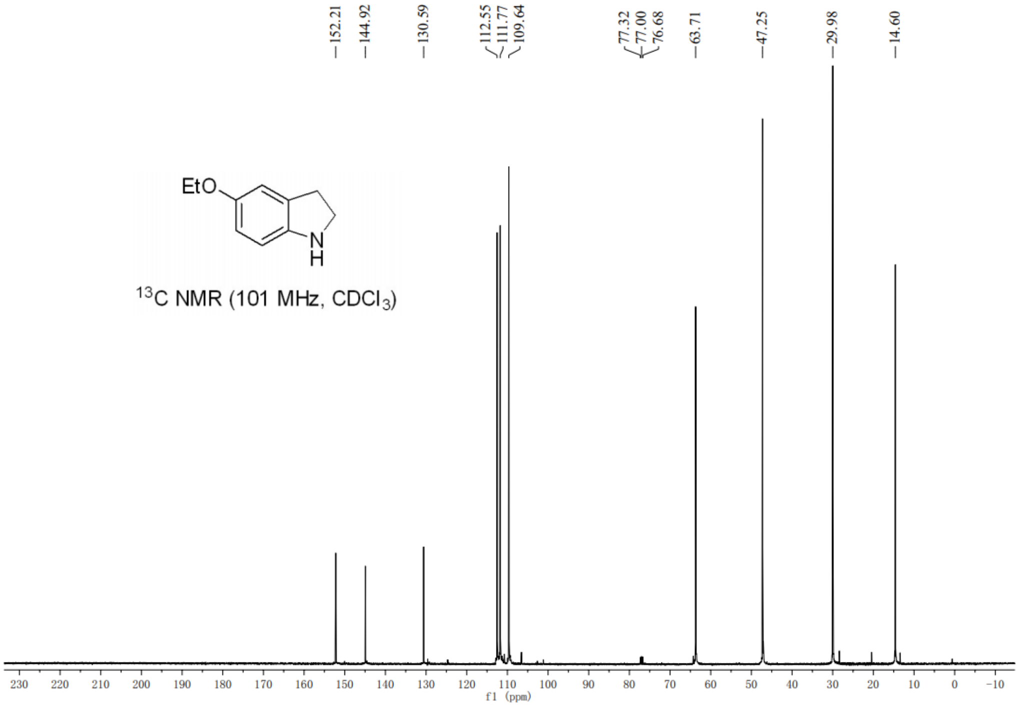

5-Isopropoxyindoline (1b-13)

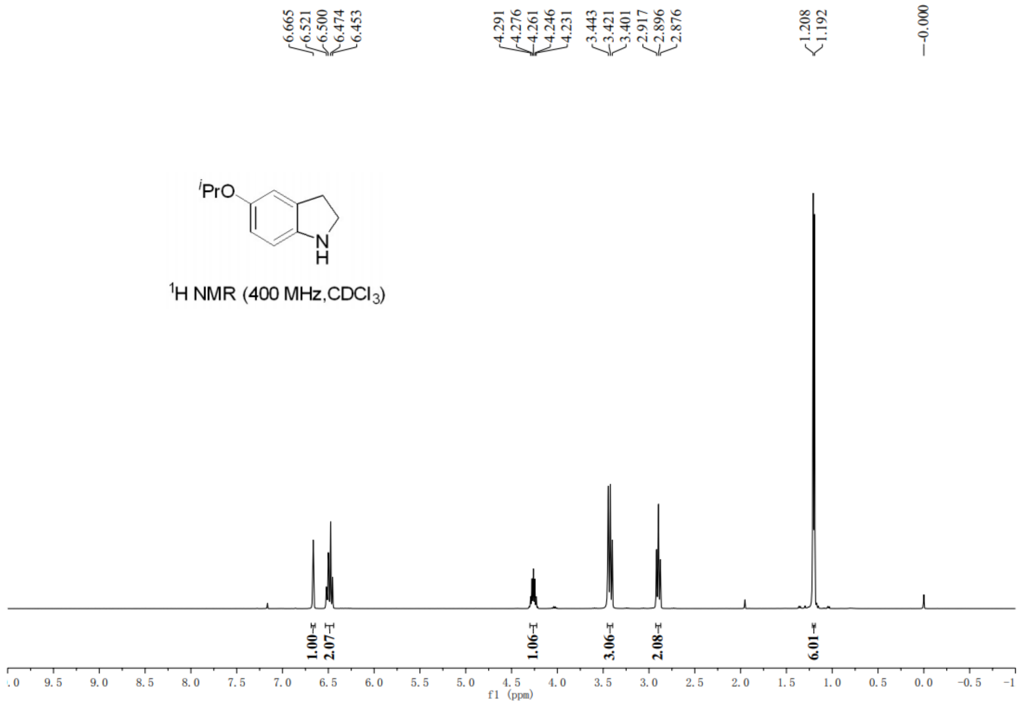

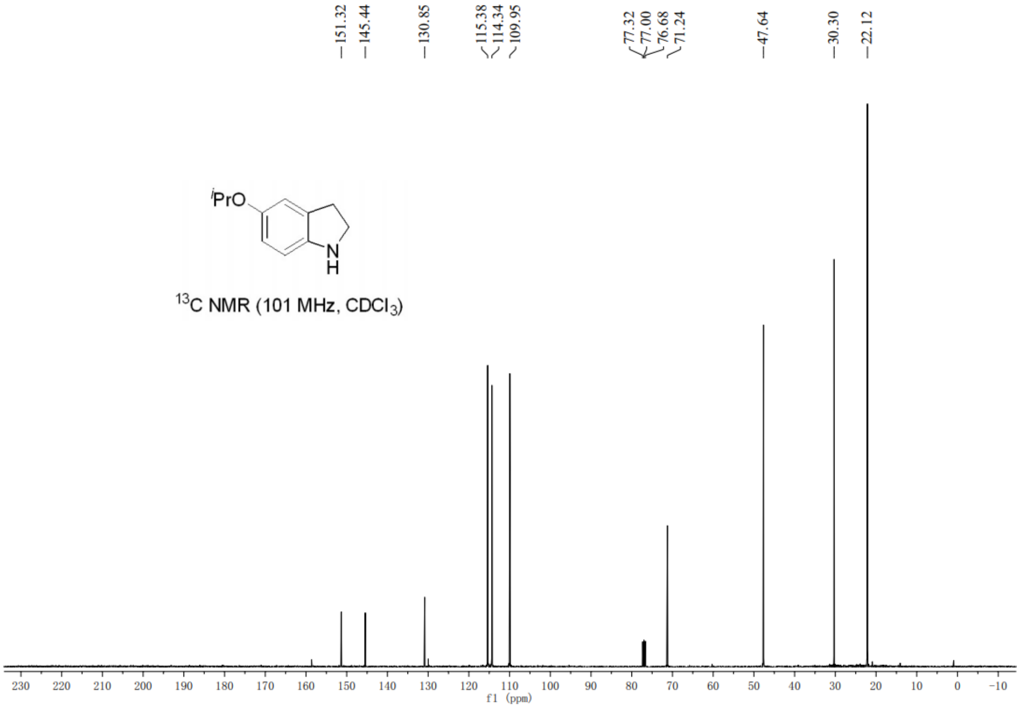

6-chloroindoline (1b-19)

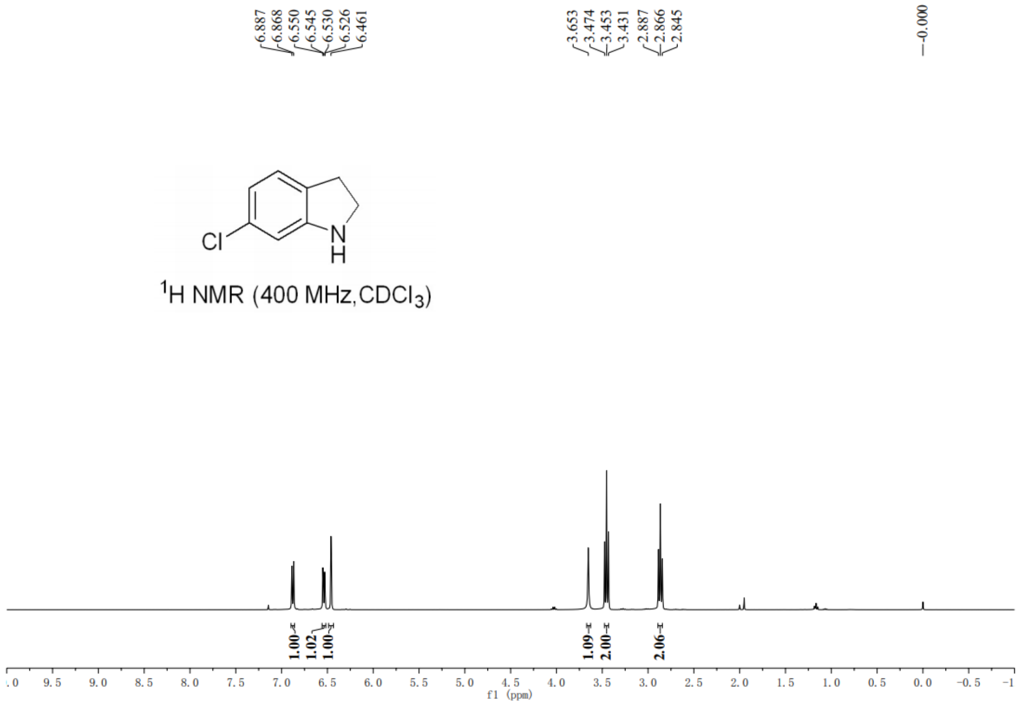

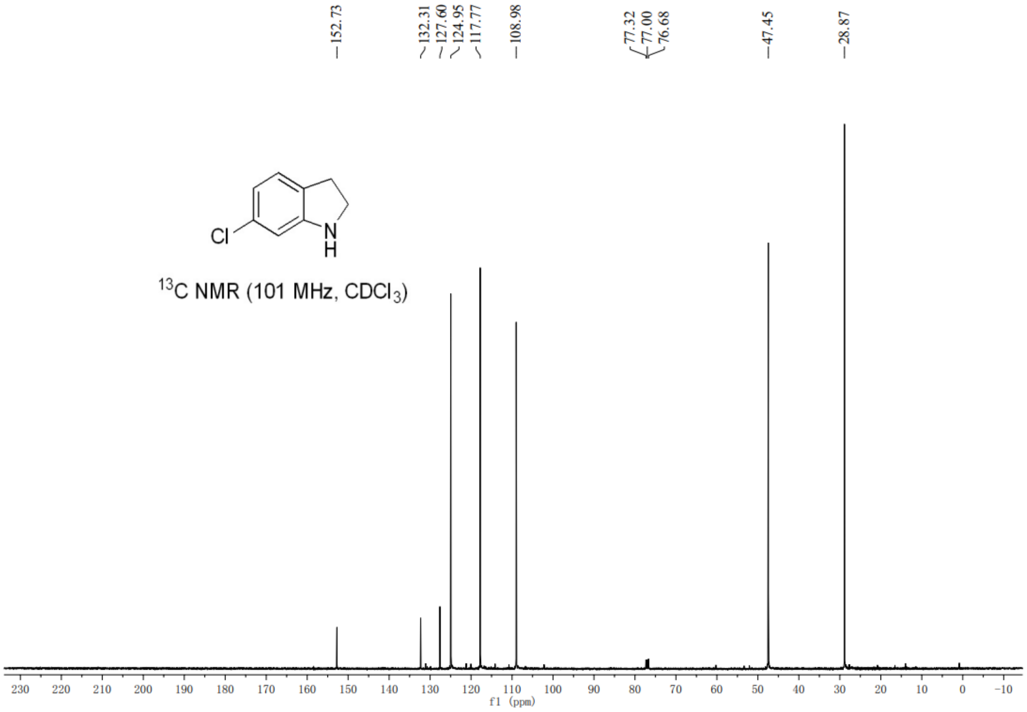

6-bromoindoline (1b-22)

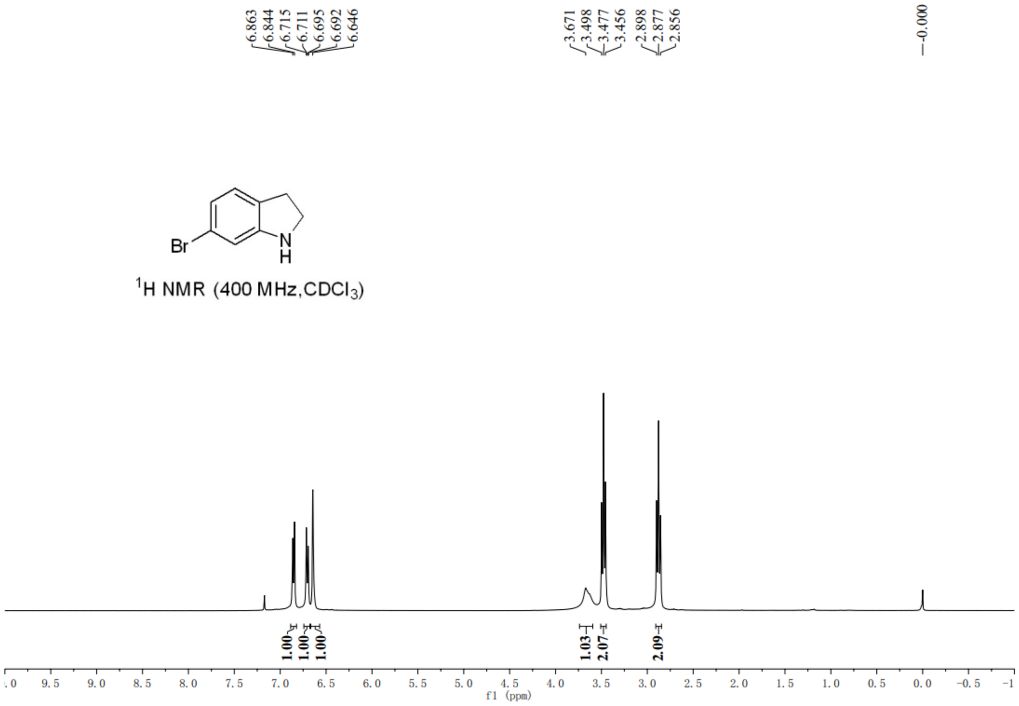

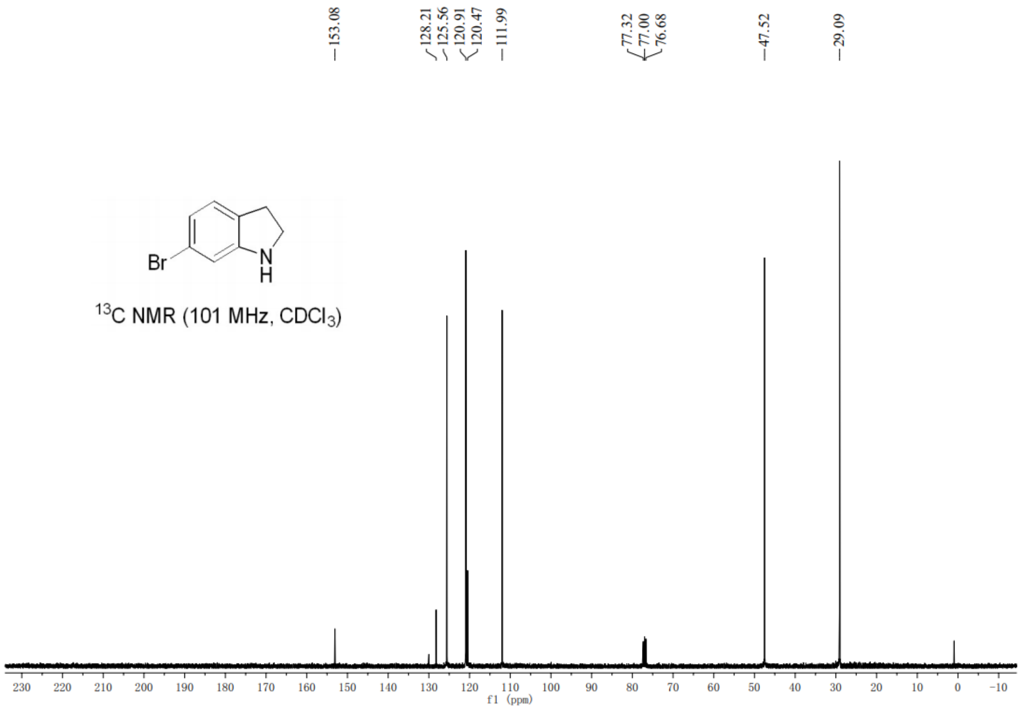

4-phenyl-1,2,3,4-tetrahydroquinoline (5a)

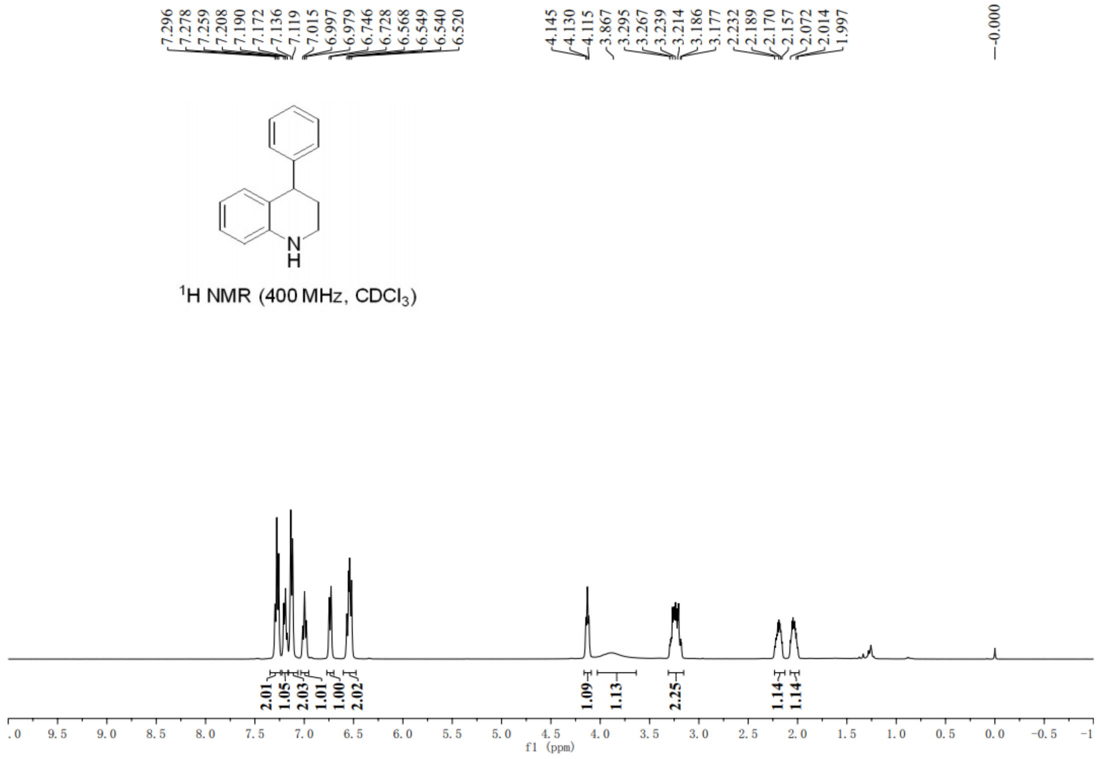

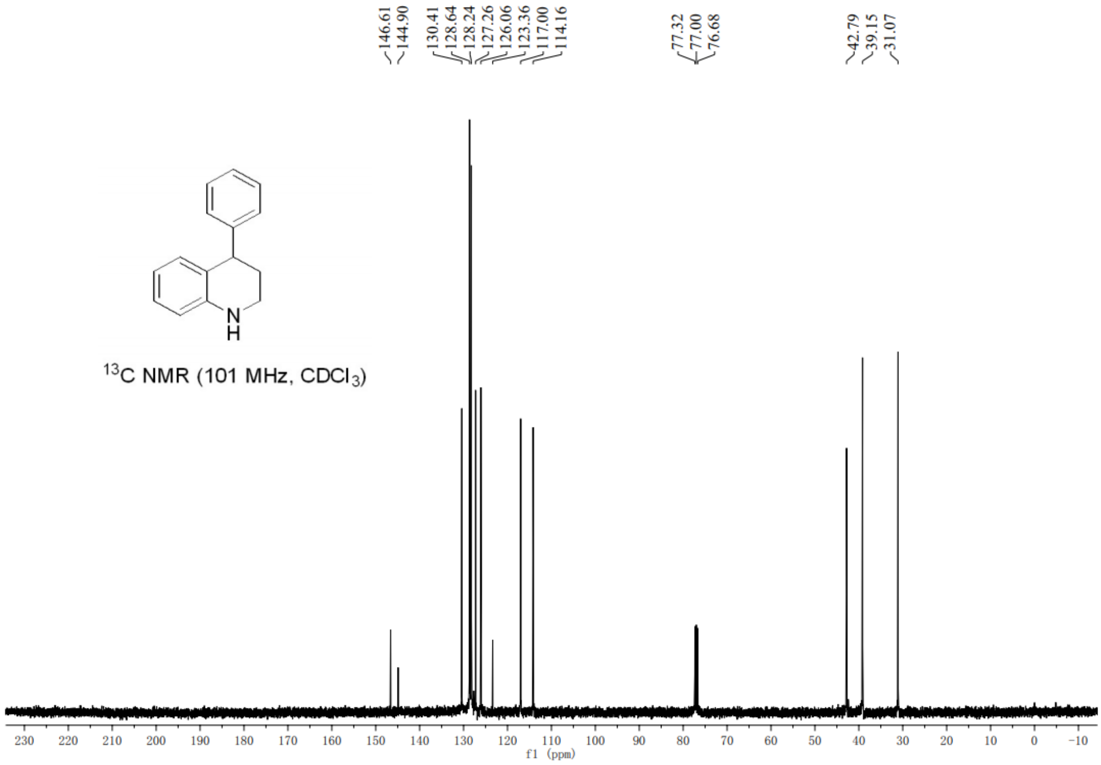

3-methyl-4-phenyl-1,2,3,4-tetrahydroquinoline (5b)

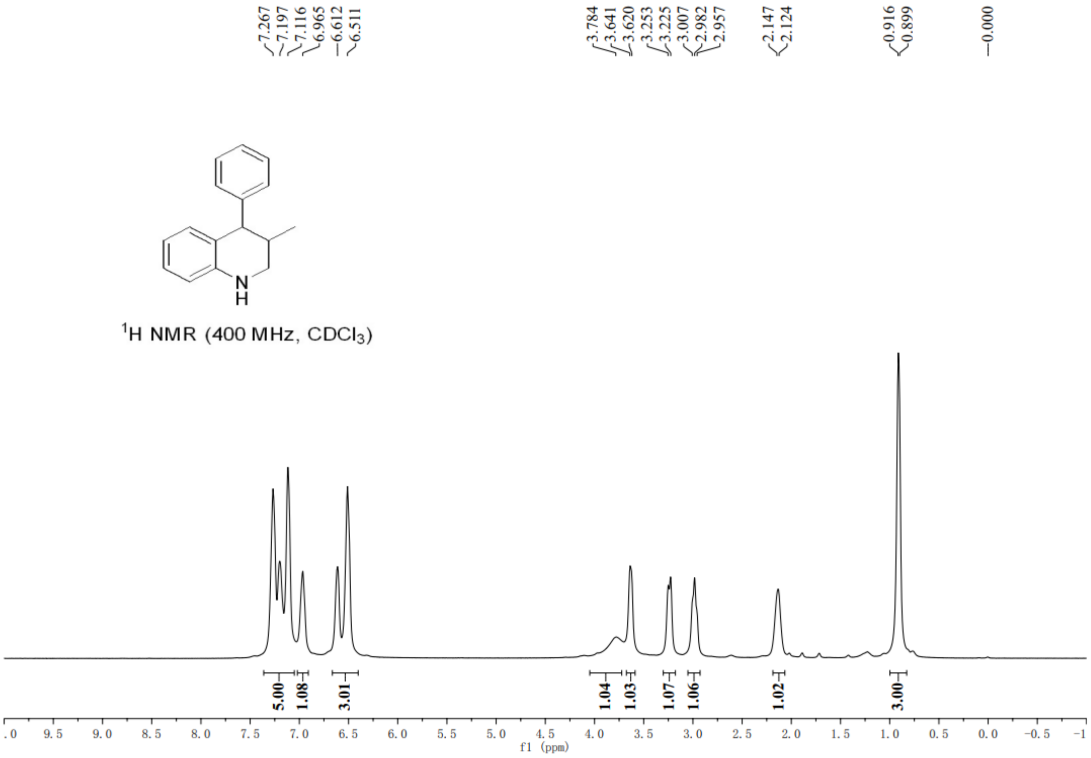

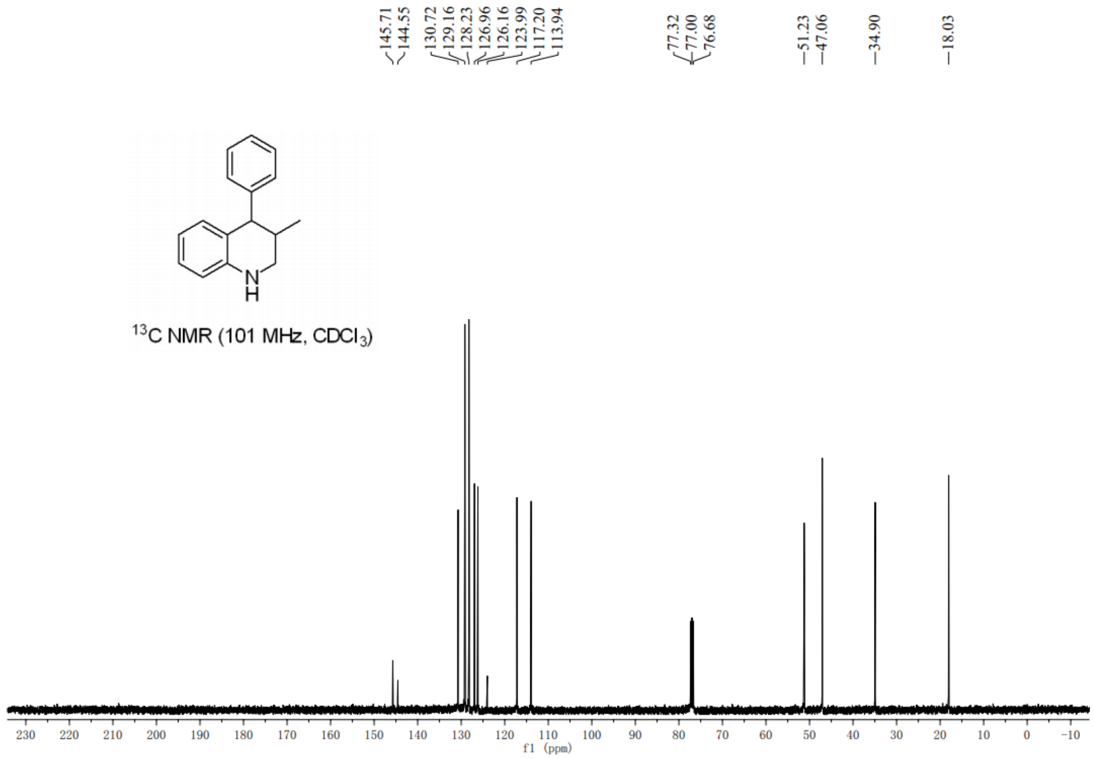

1-methyl-4-phenyl-1,2,3,4-tetrahydroquinoline (5c)

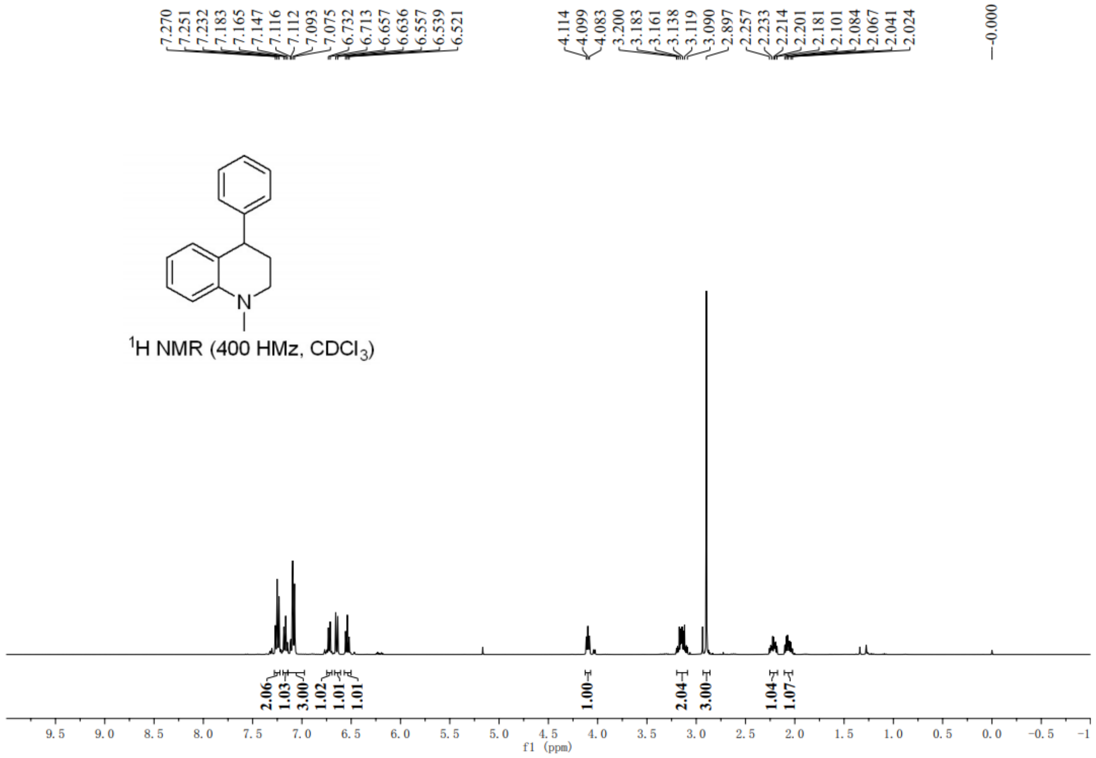

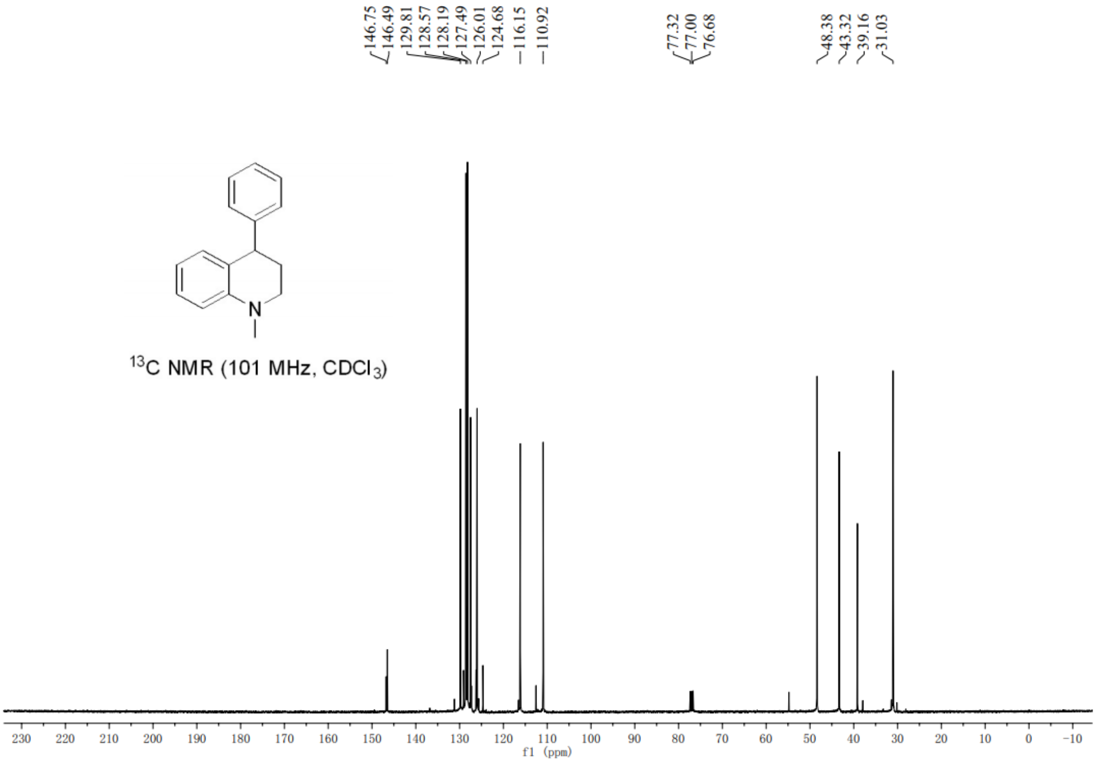

1,4-bis(4-nitrophenyl)-4-phenyl-1,2,3,4-tetrahydroquinoline (6a)

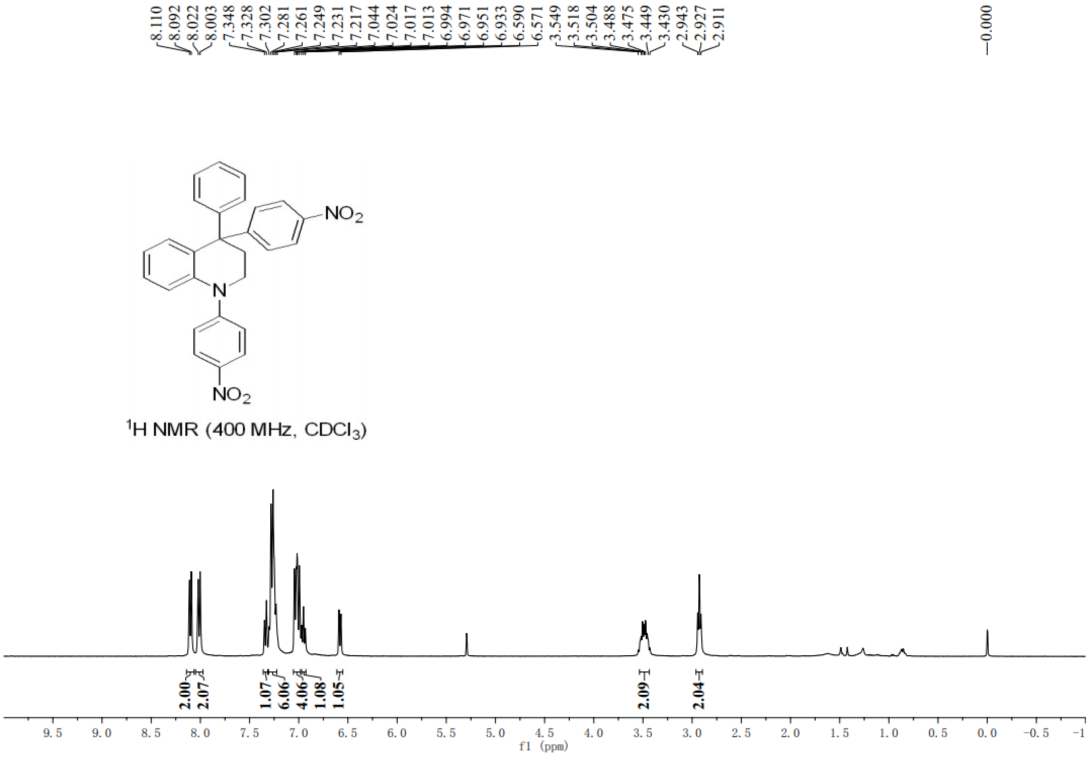

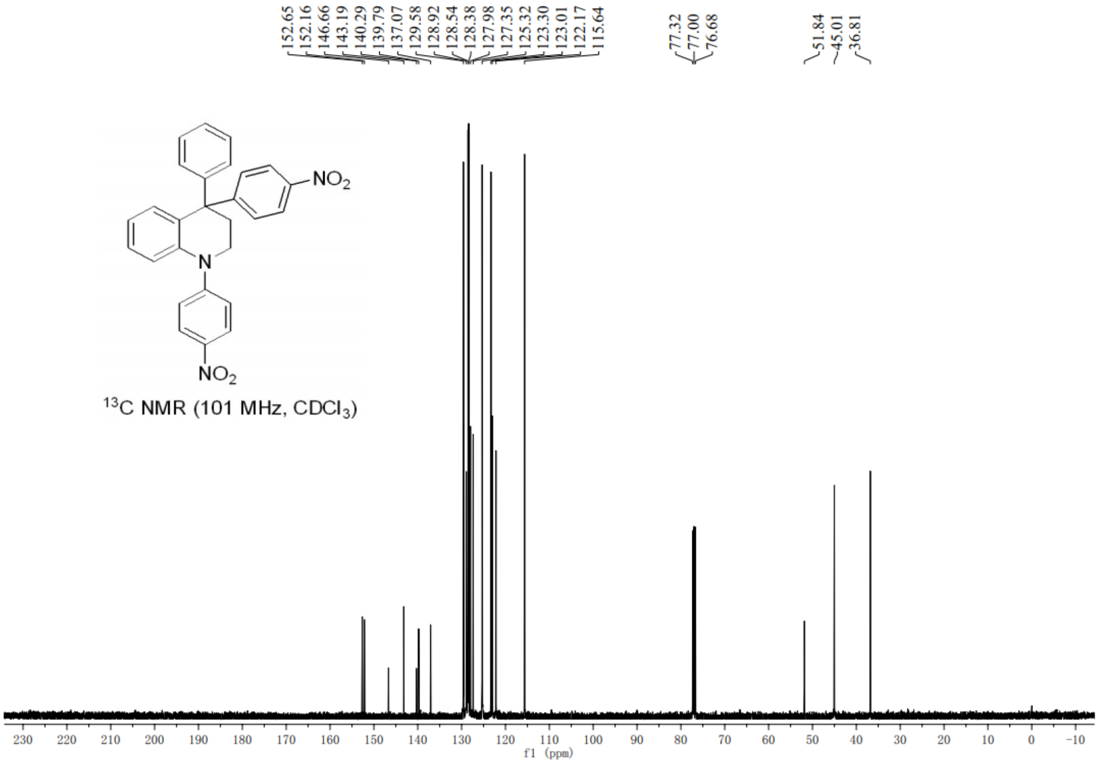

3-methyl-1-(4-nitrophenyl)-4-phenyl-1,2,3,4-tetrahydroquinoline (6b)

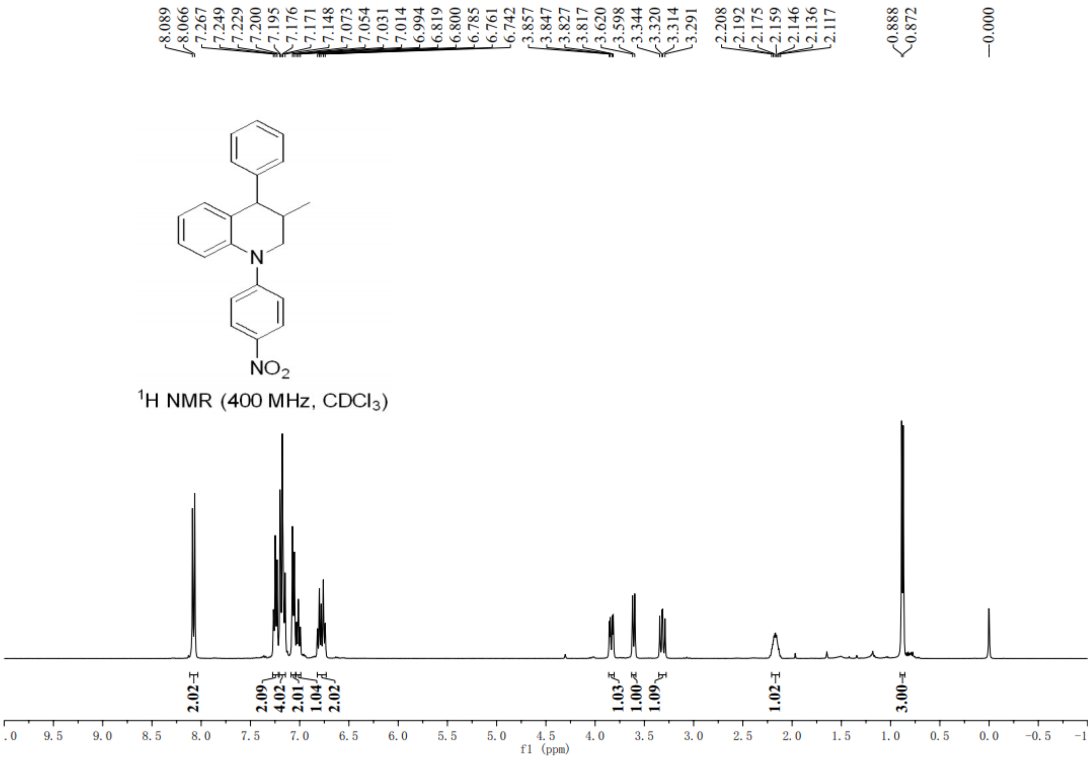

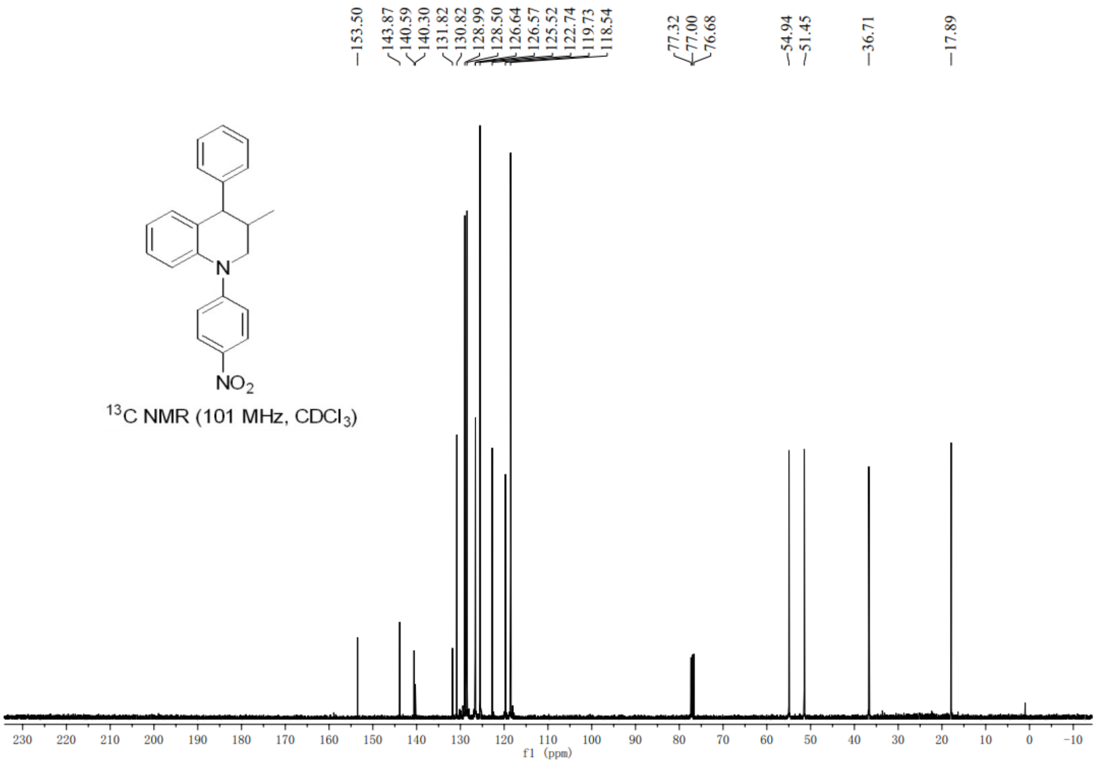

1,2-diphenyldiazene (6d)

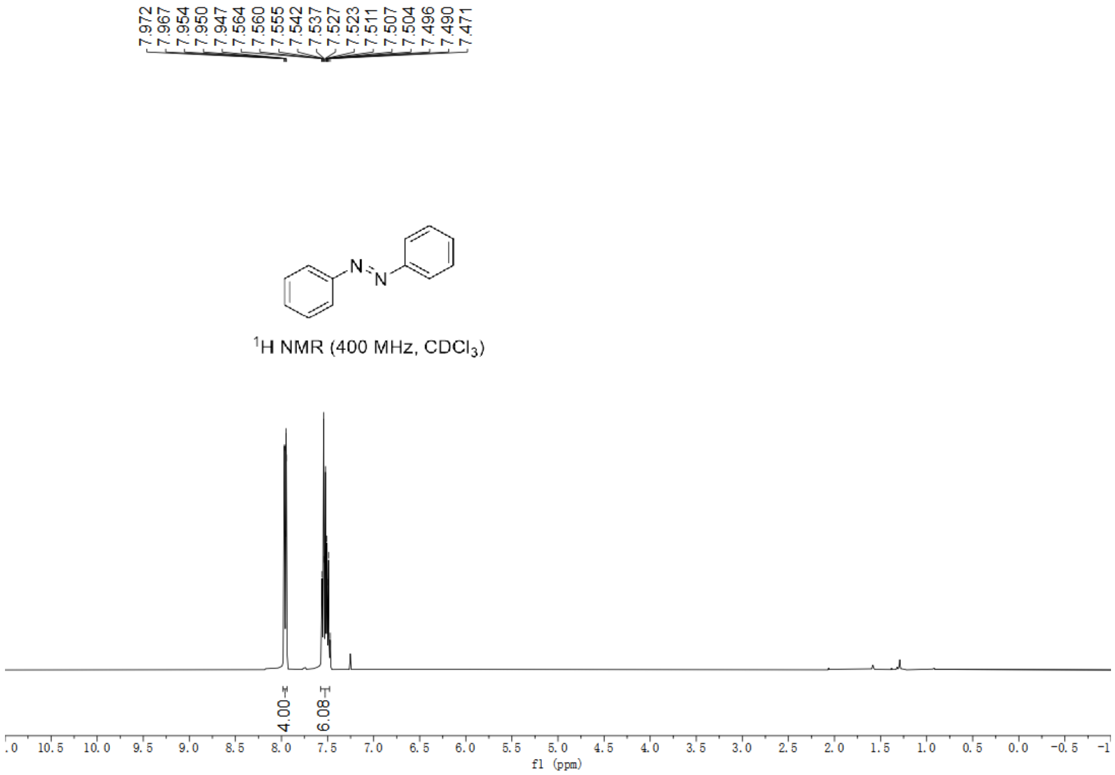

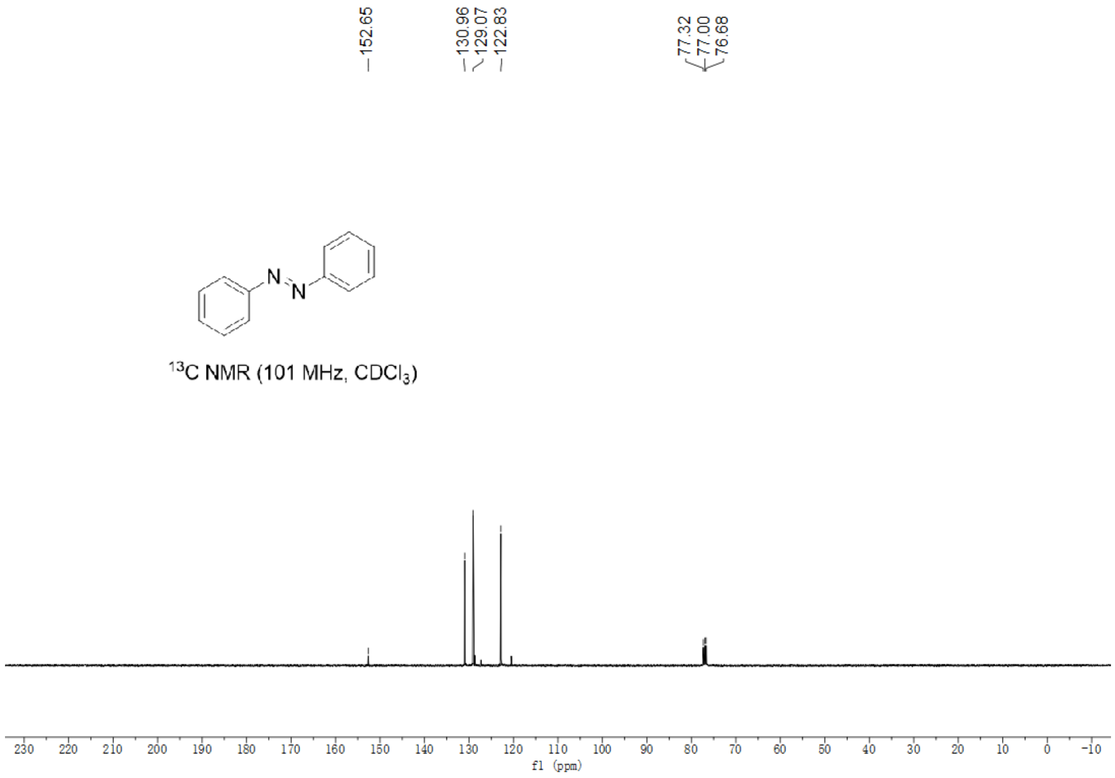

(*Z*)-1,2-diphenyldiazene 1-oxide (6e)

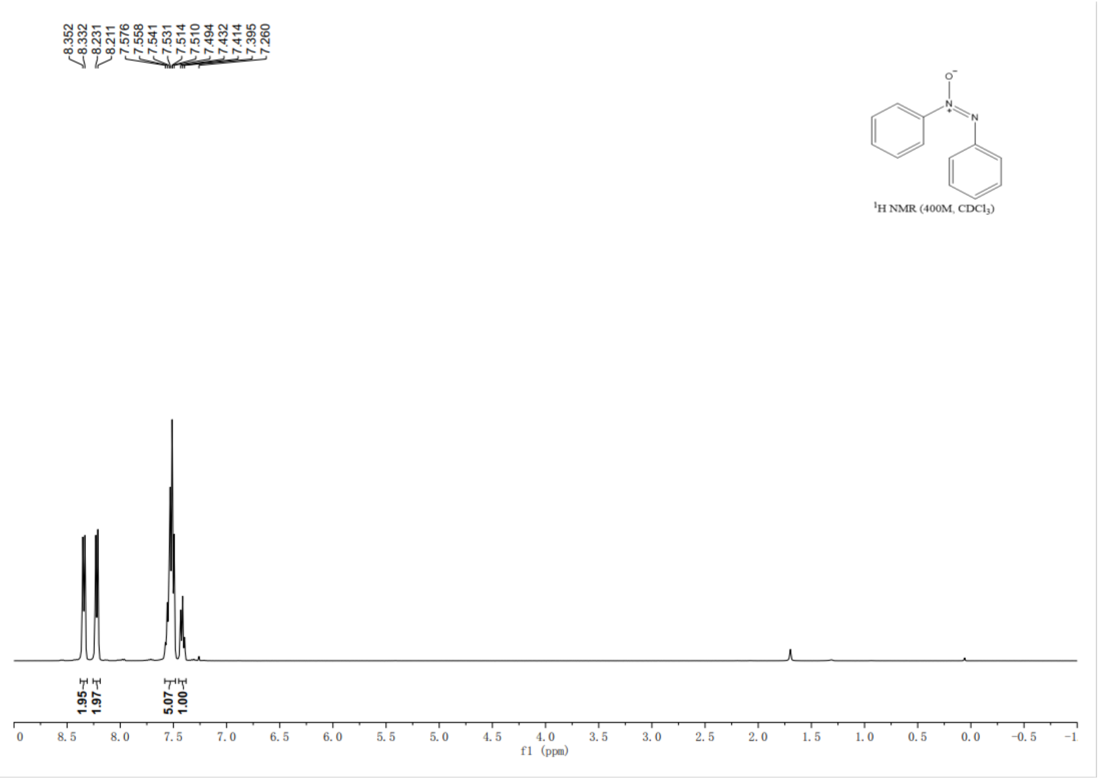

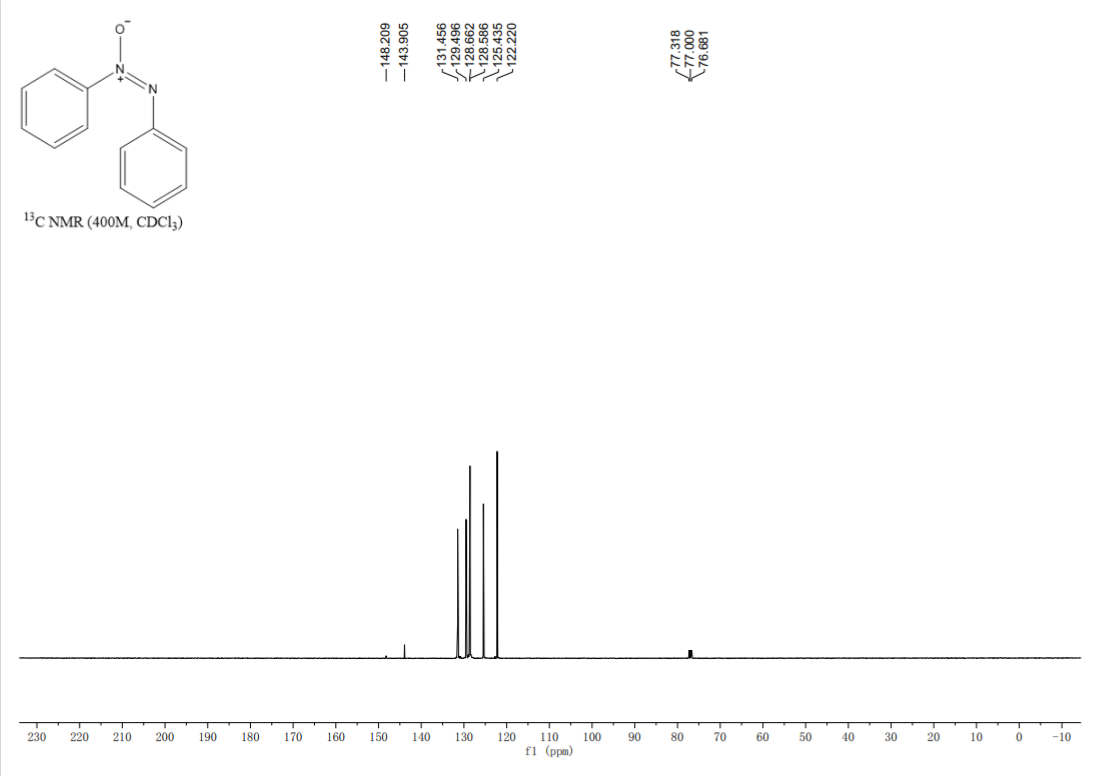

***N*-cyclopropylaniline (5g)**

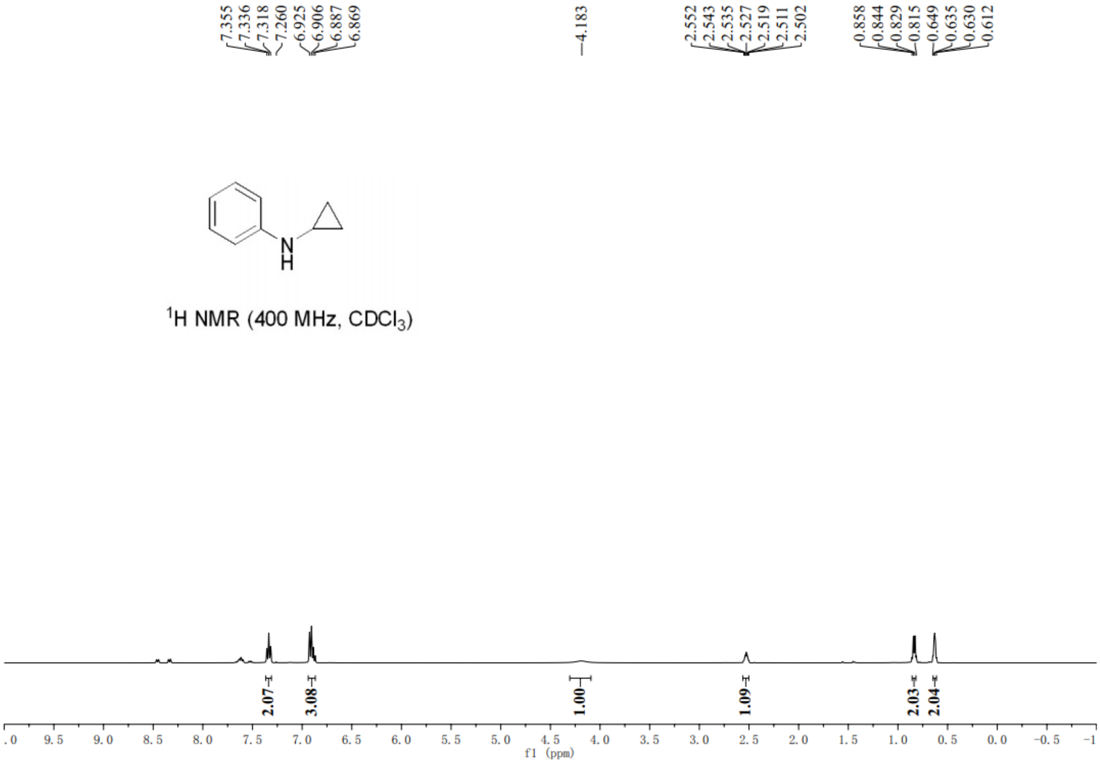

^{1}H NMR (400 MHz, $CDCl_3$)

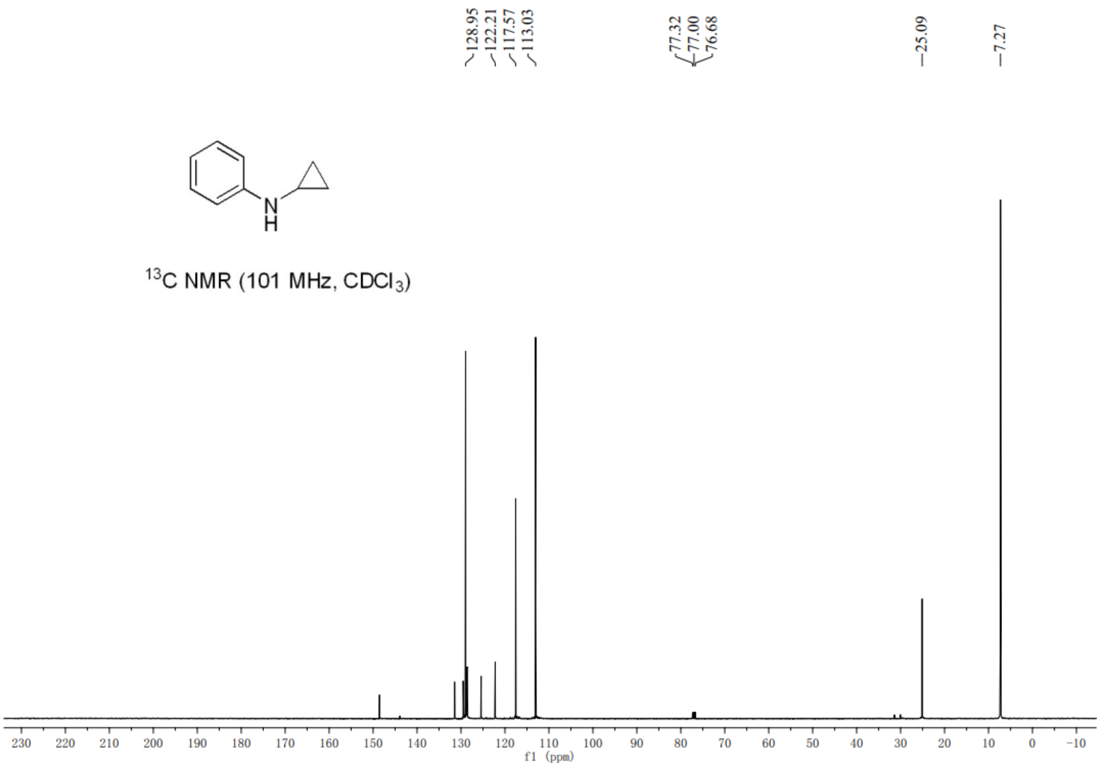

^{13}C NMR (101 MHz, $CDCl_3$)

***N*-cyclopropyl-4-nitro-*N*-phenylaniline (6h)**

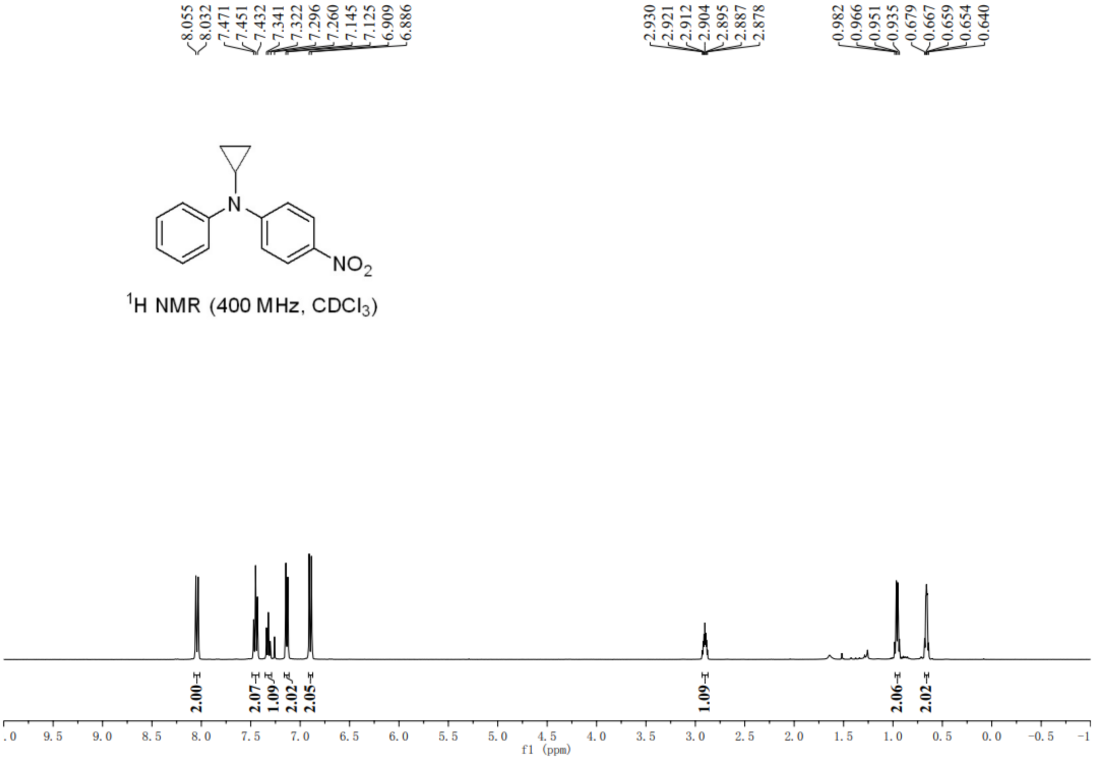

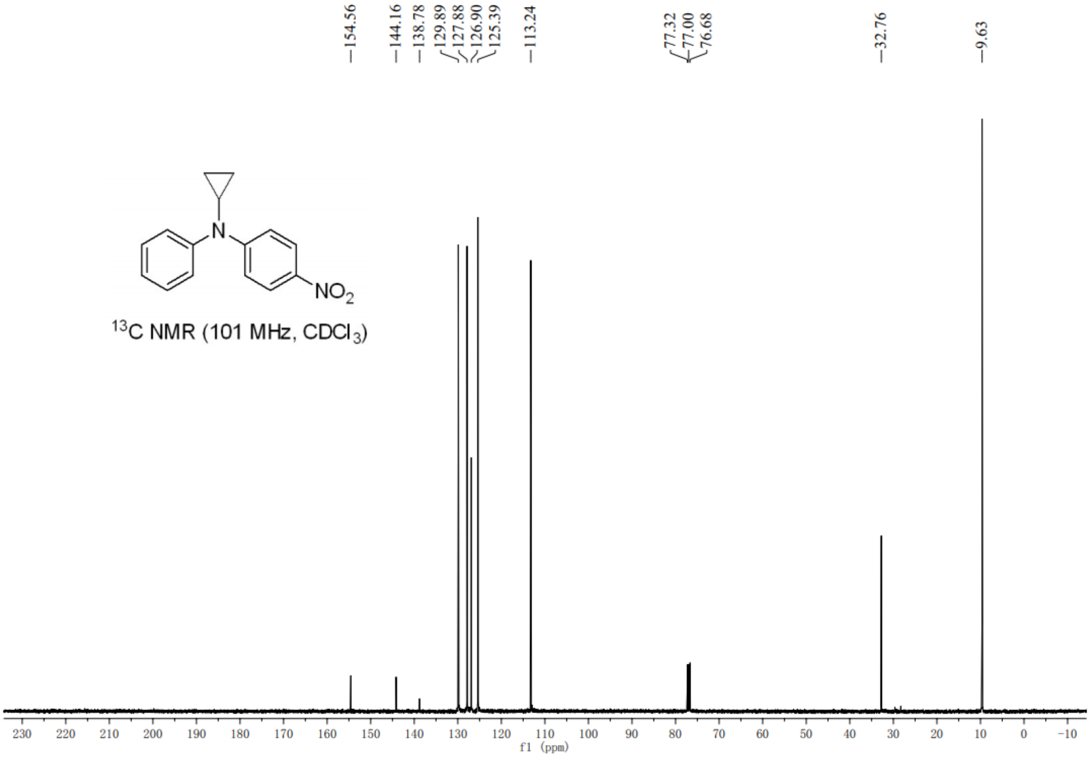

N-methyl-4-(1*H*-pyrrol-1-yl)aniline (1c-15)

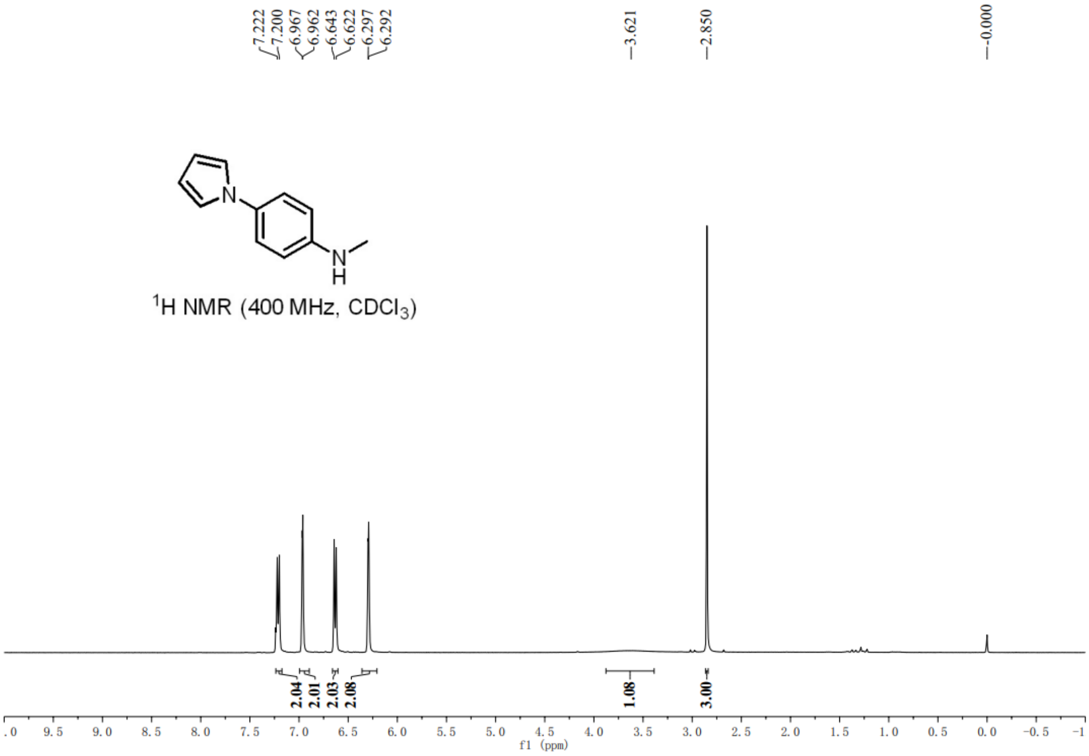

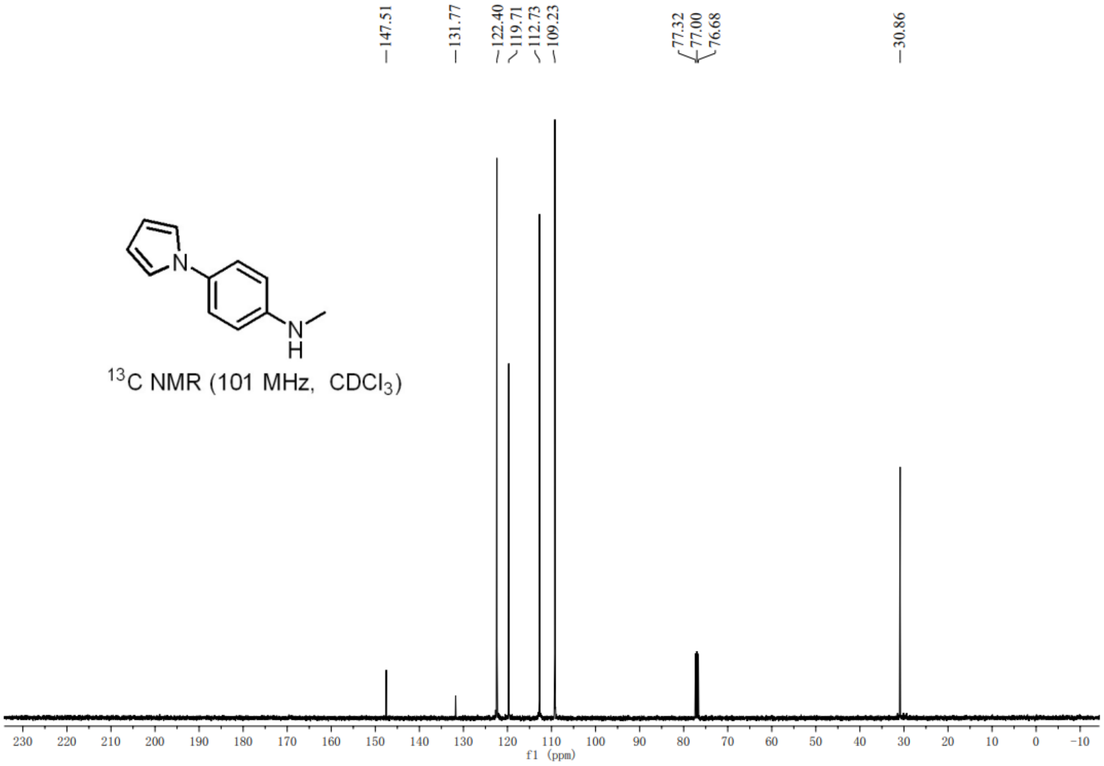

4-nitro-*N*-phenylaniline (6g)

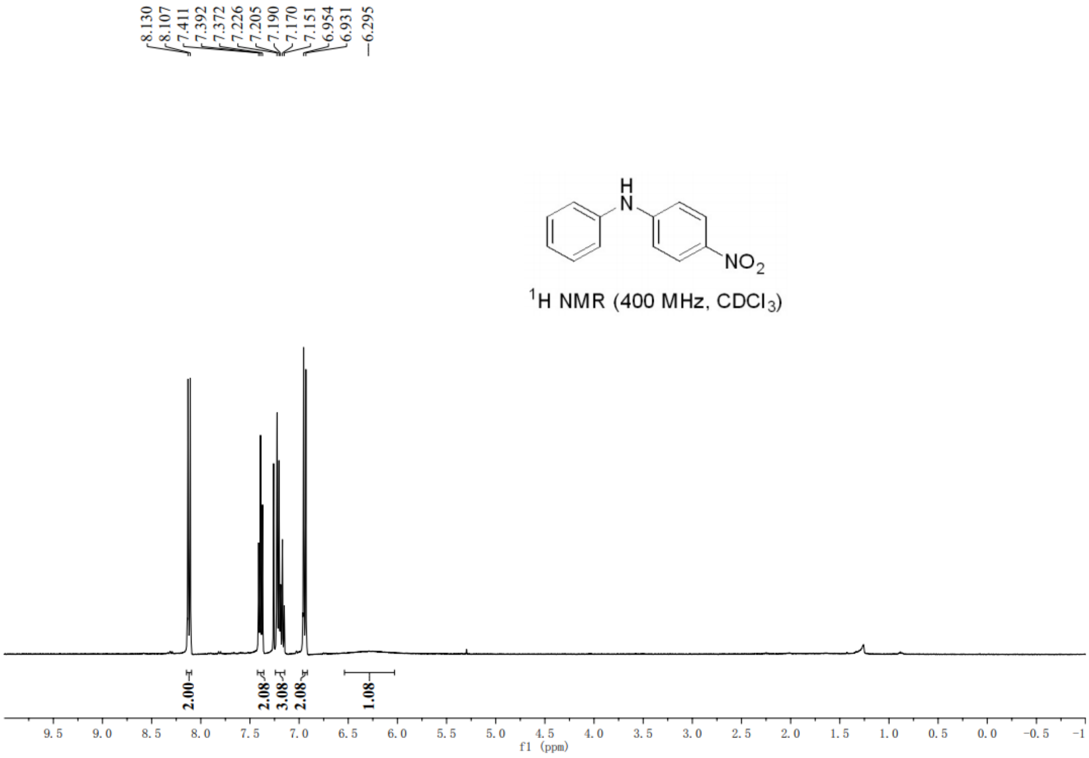

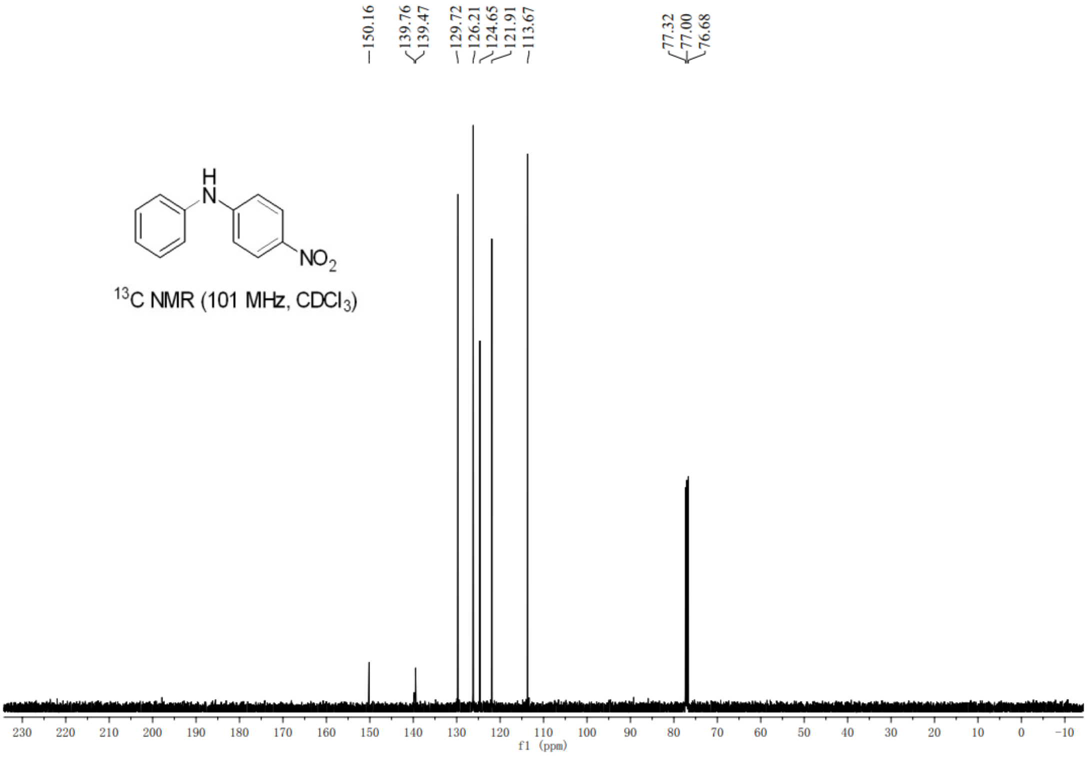

3-methoxy-*N*-(4-nitrophenyl)aniline (6i)

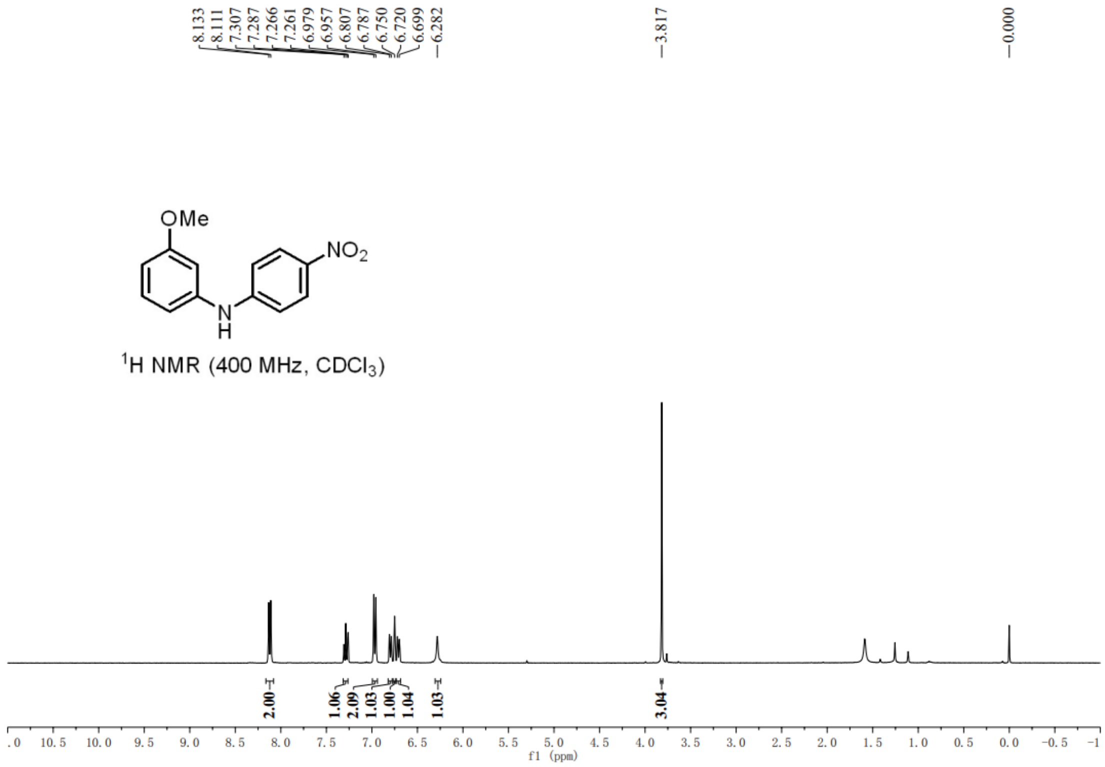

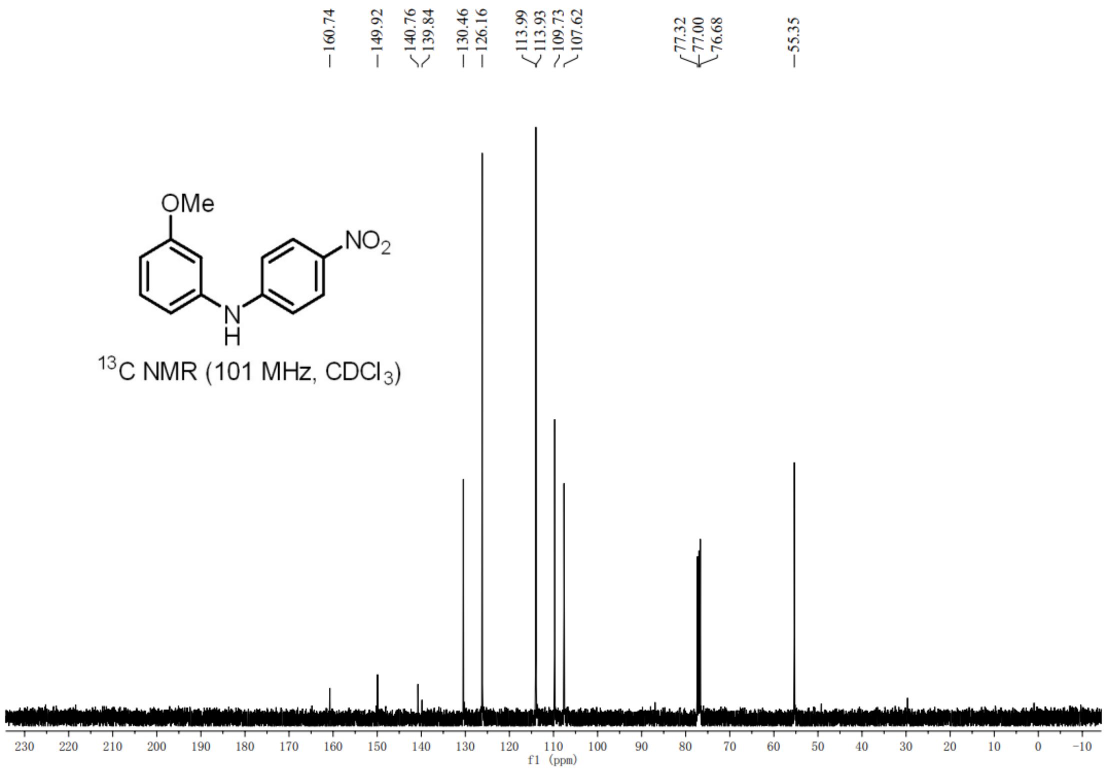

1-(4-nitrophenyl)-1,2,3,4-tetrahydroquinoline (3a-1)

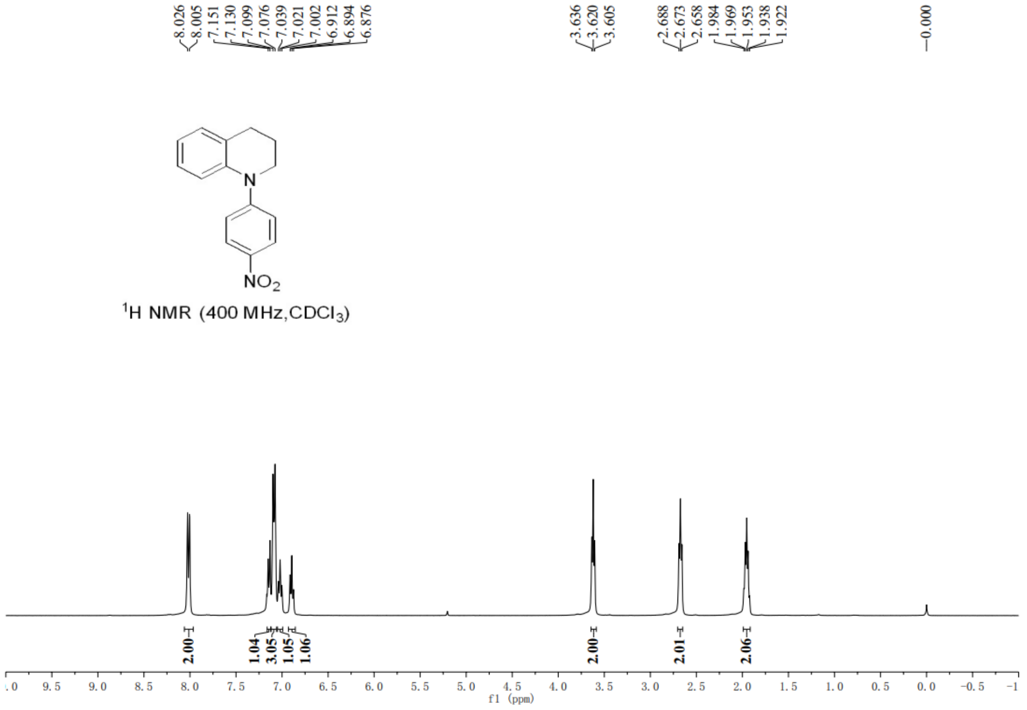

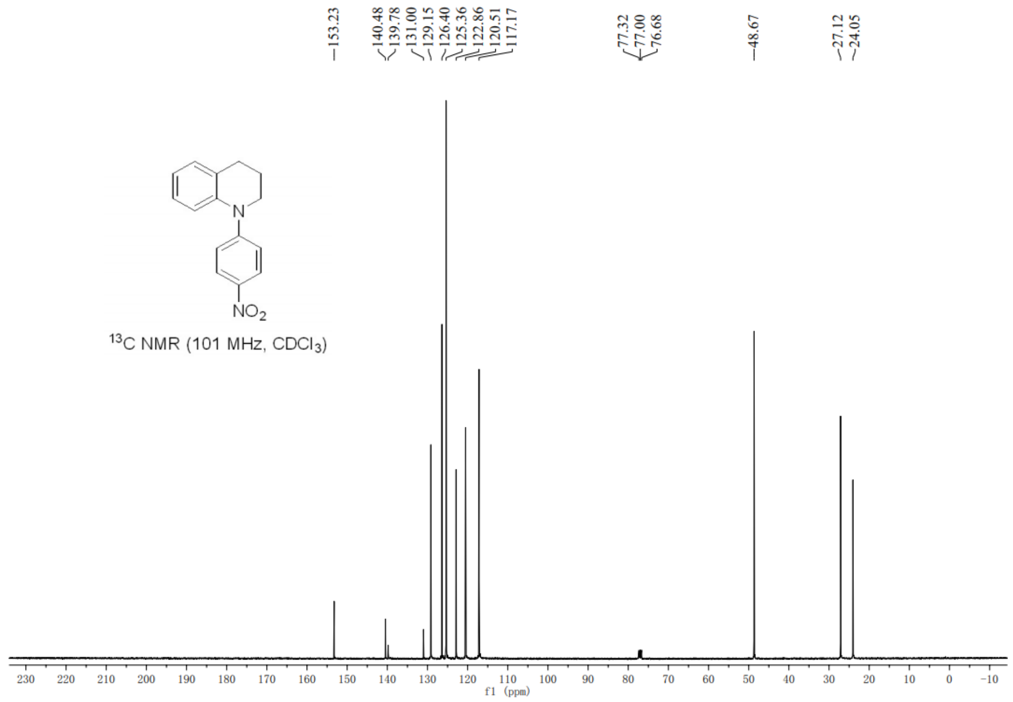

2-methyl-1-(4-nitrophenyl)-1,2,3,4-tetrahydroquinoline (3a-2)

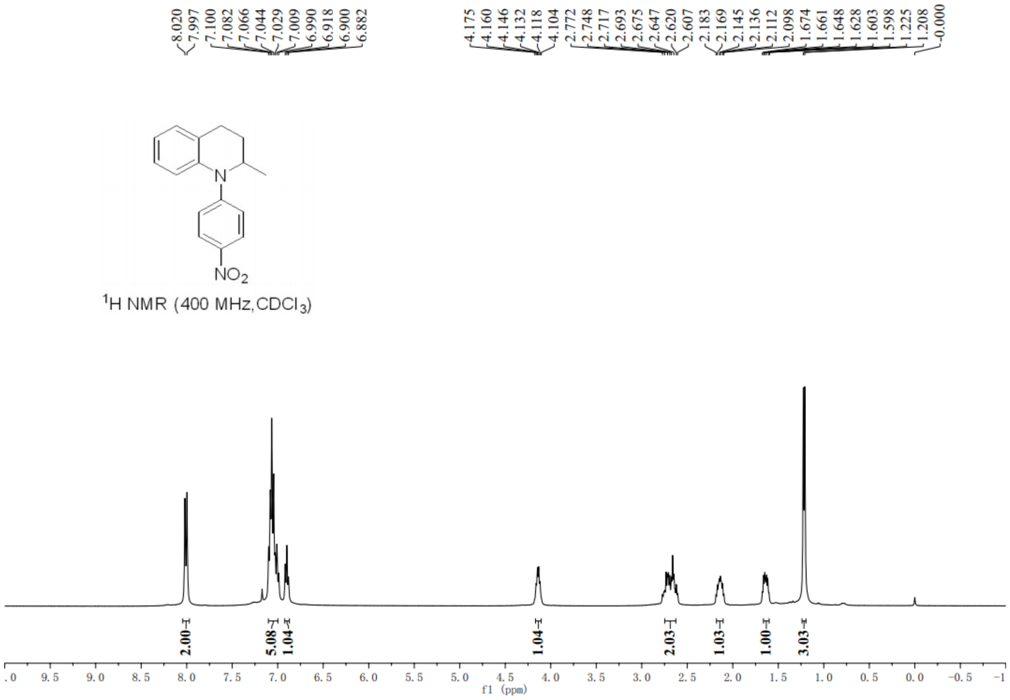

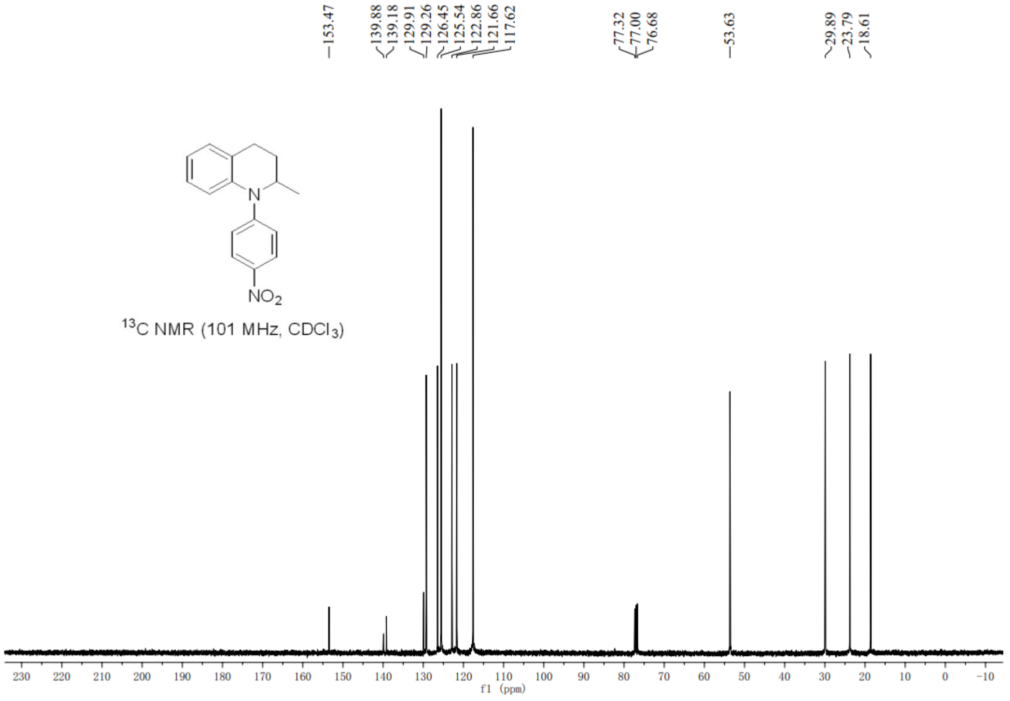

3-methyl-1-(4-nitrophenyl)-1,2,3,4-tetrahydroquinoline (3a-3)

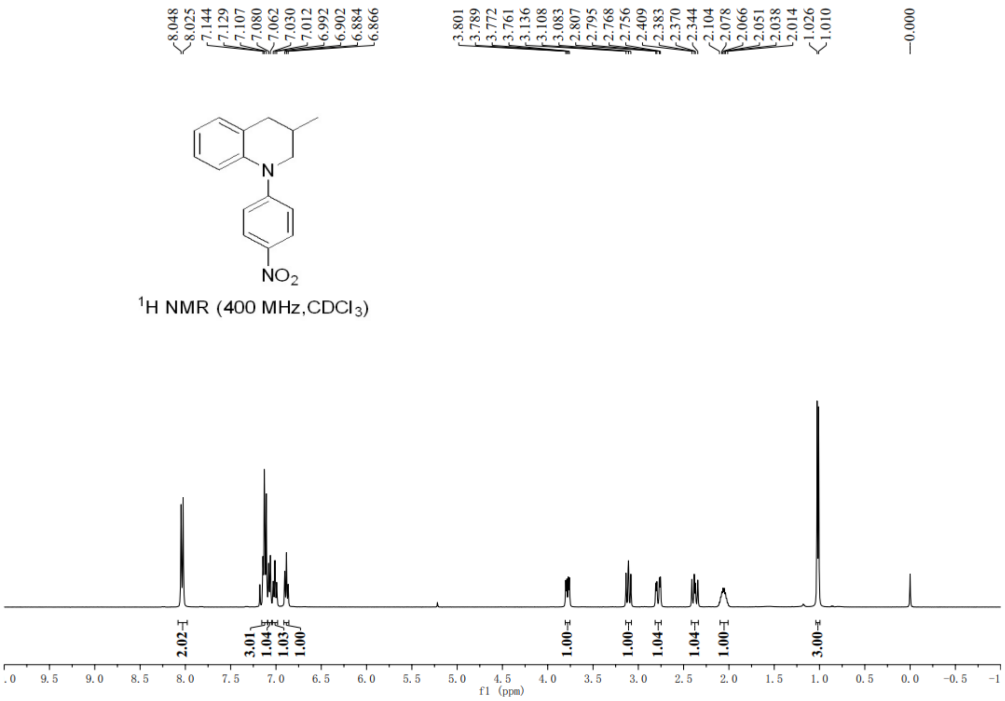

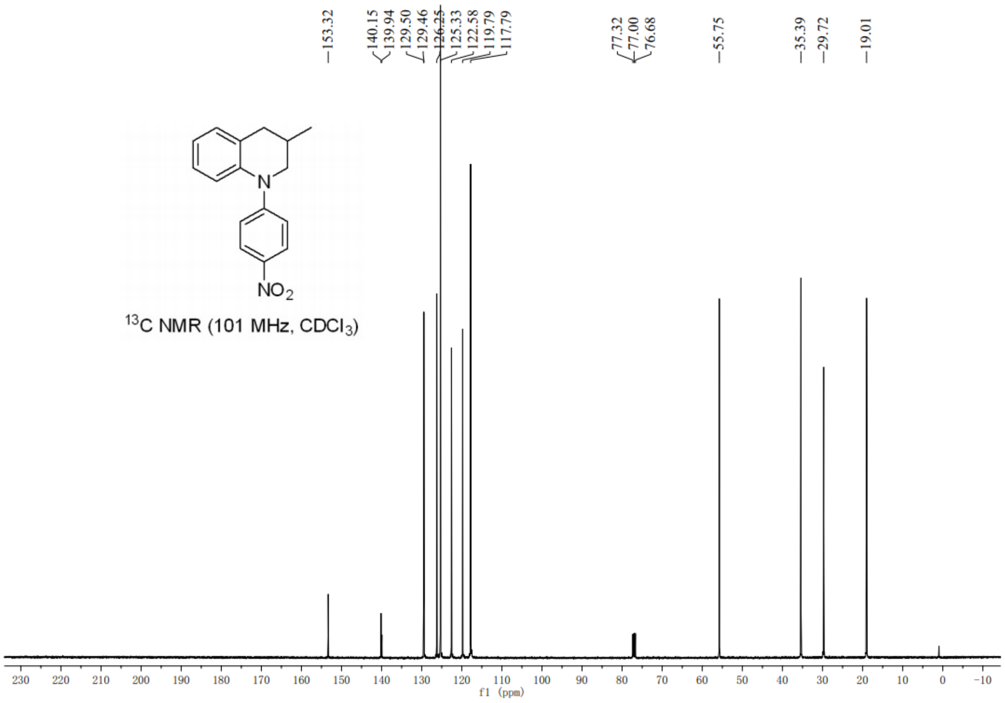

4-methyl-1-(4-nitrophenyl)-1,2,3,4-tetrahydroquinoline (3a-4)

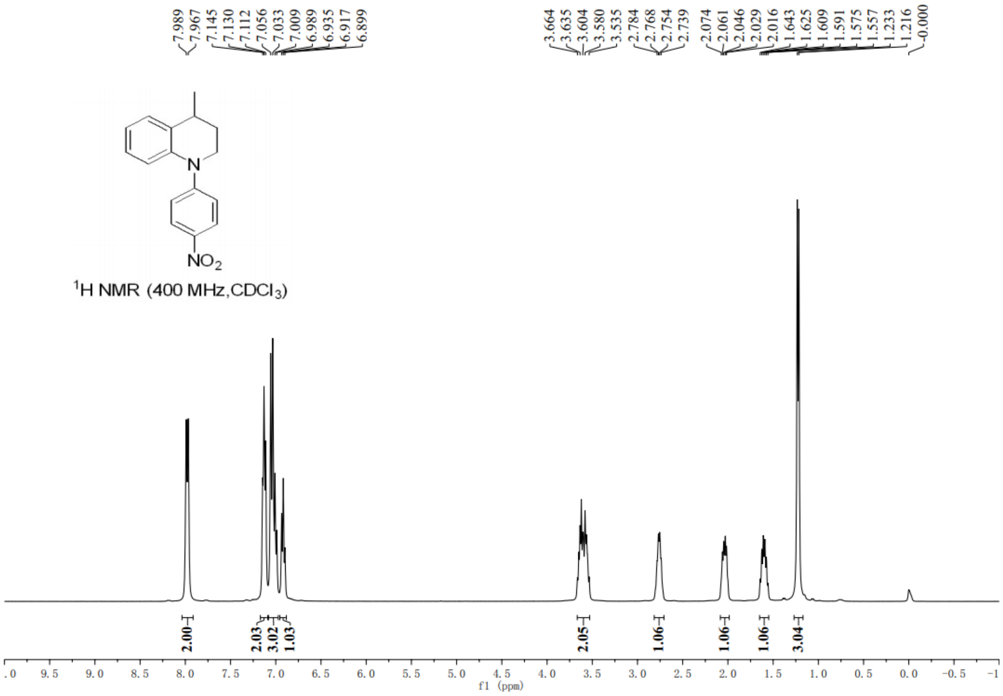

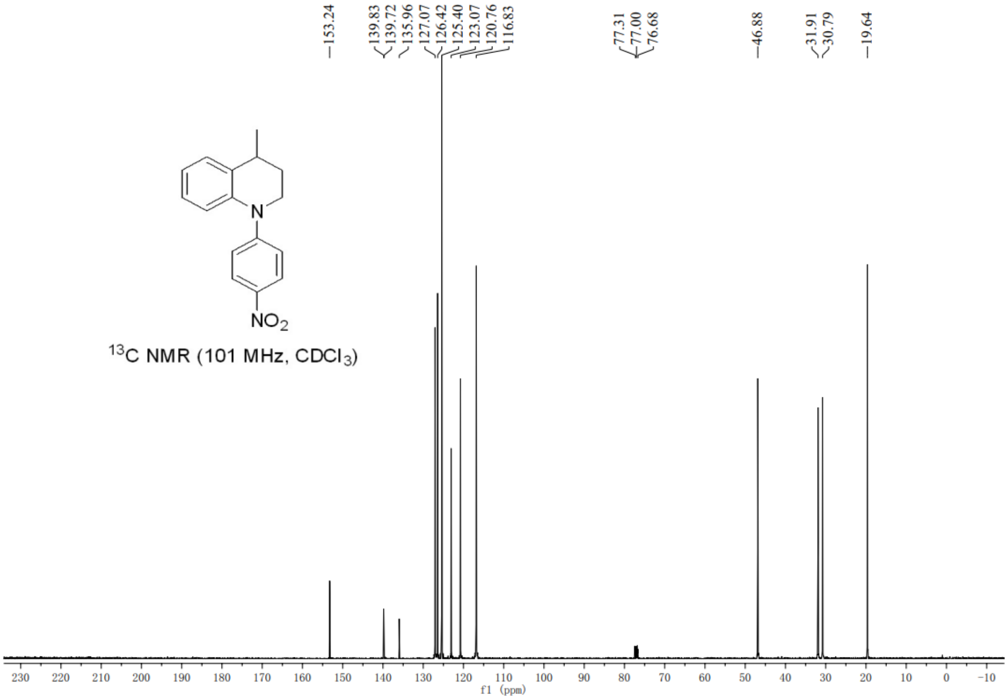

5-methyl-1-(4-nitrophenyl)-1,2,3,4-tetrahydroquinoline (3a-5)

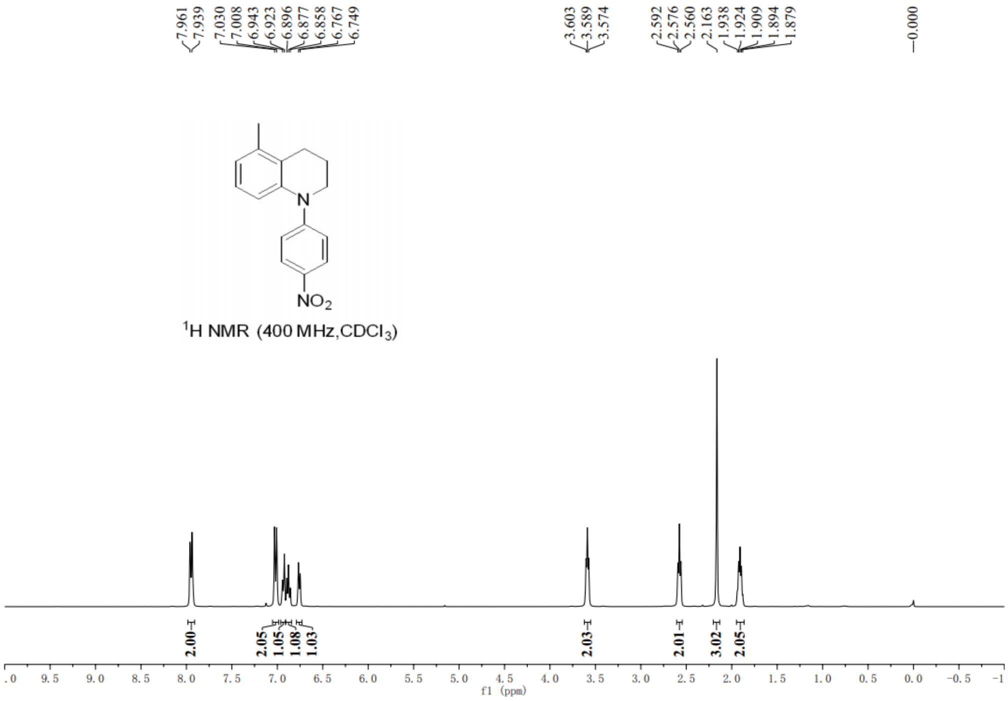

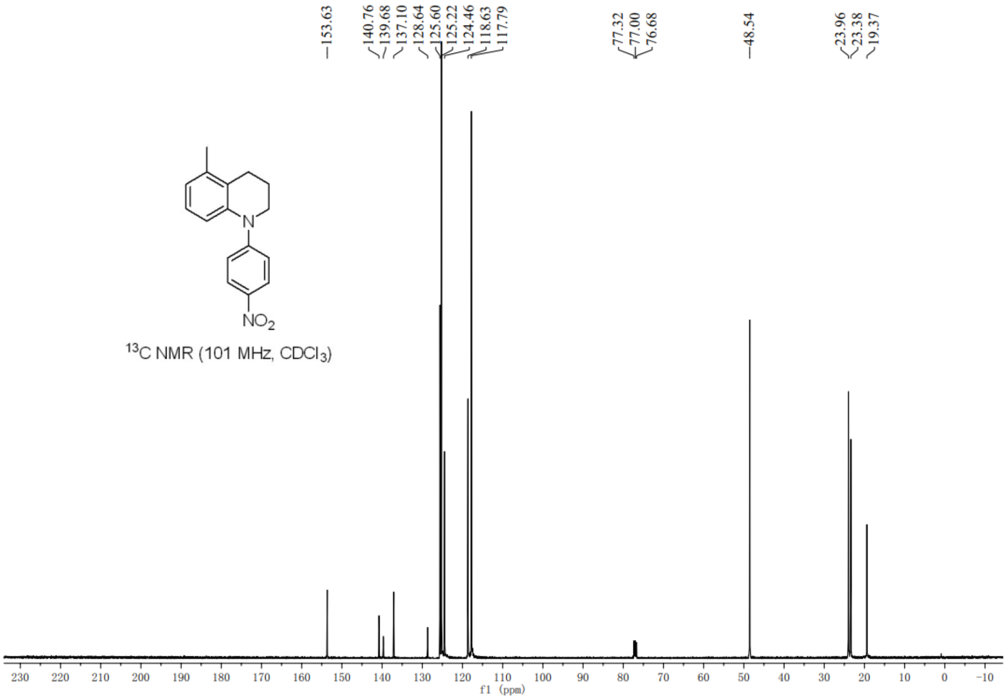

6-methyl-1-(4-nitrophenyl)-1,2,3,4-tetrahydroquinoline (3a-6)

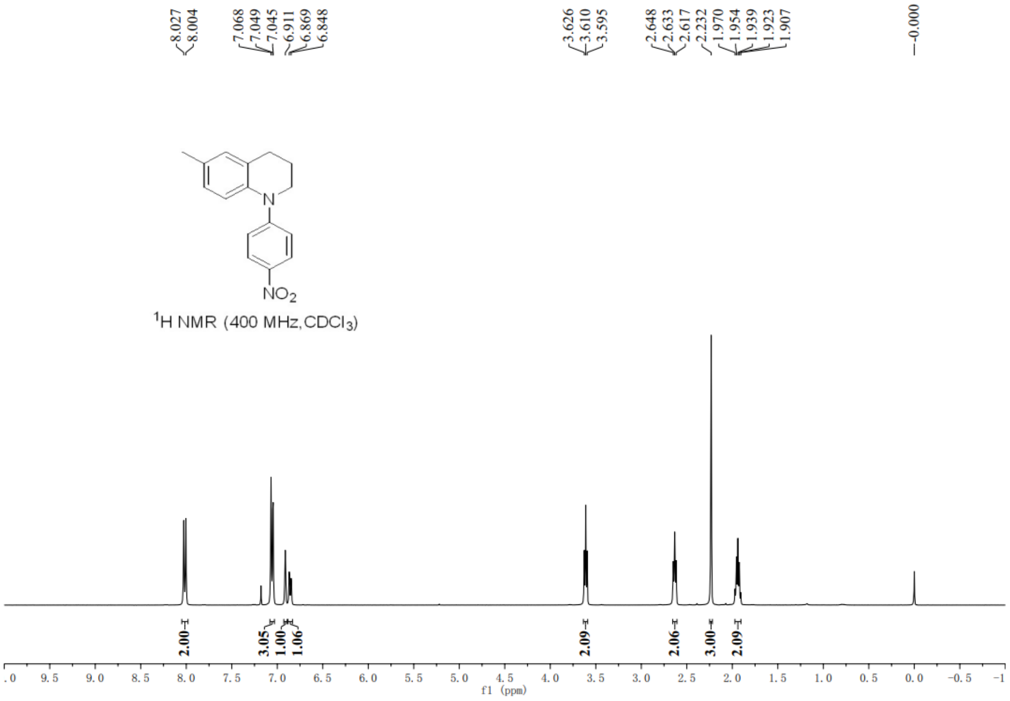

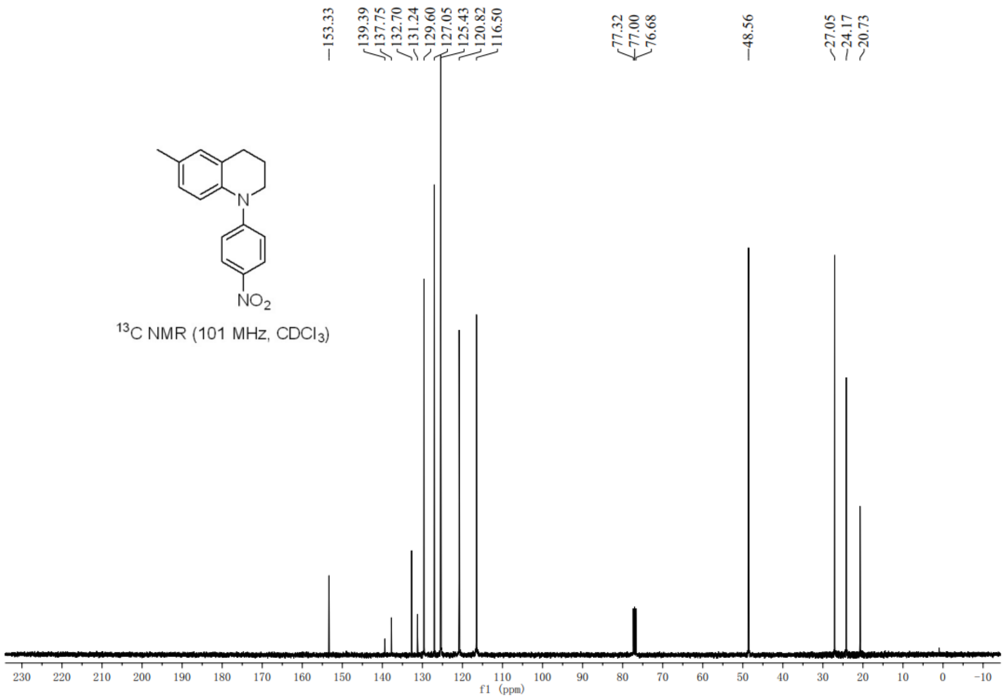

7-methyl-1-(4-nitrophenyl)-1,2,3,4-tetrahydroquinoline (3a-7)

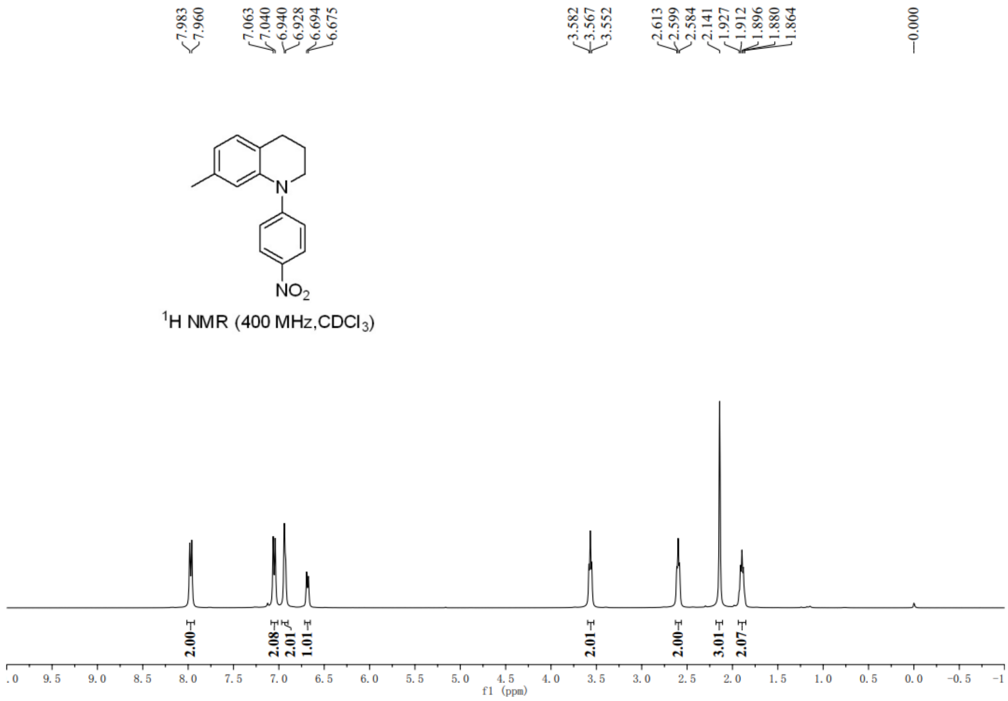

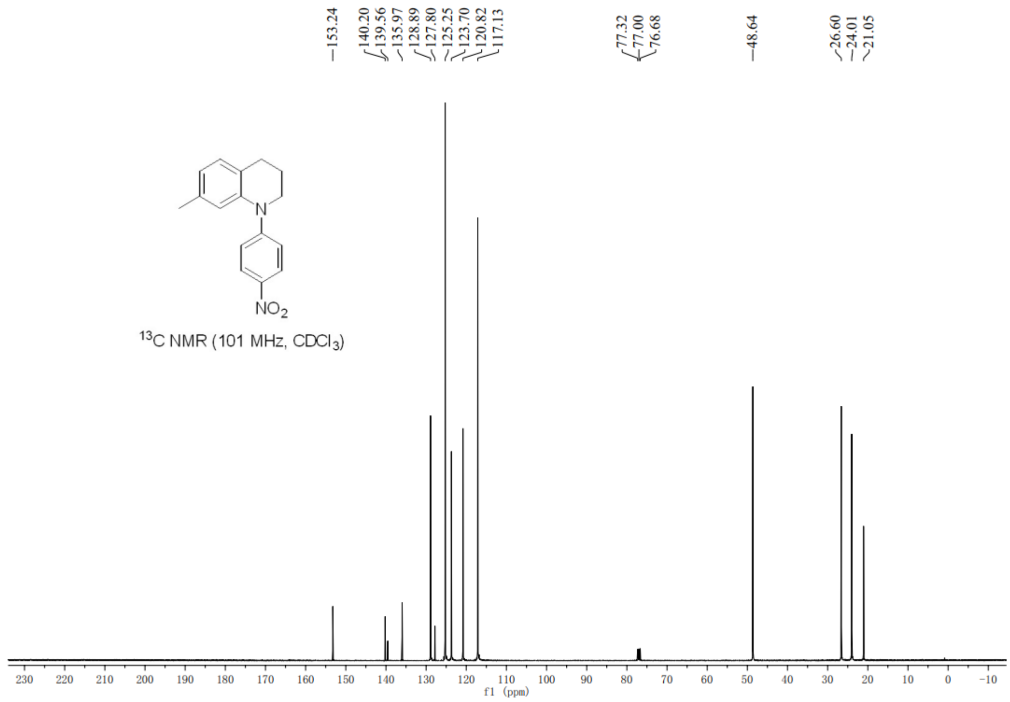

5-methoxy-1-(4-nitrophenyl)-1,2,3,4-tetrahydroquinoline (3a-8)

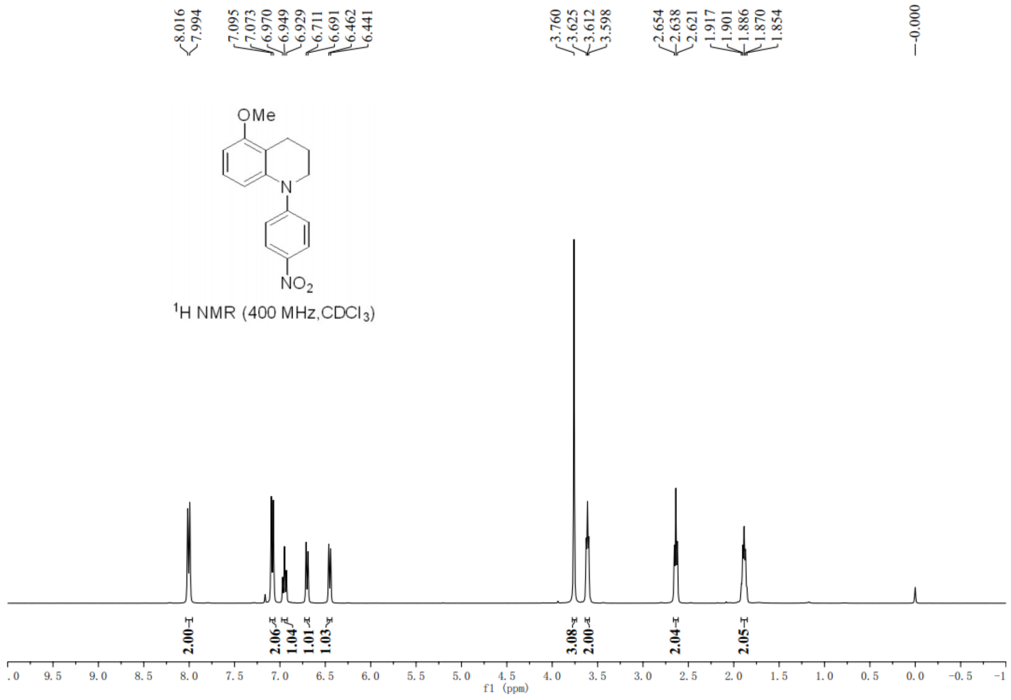

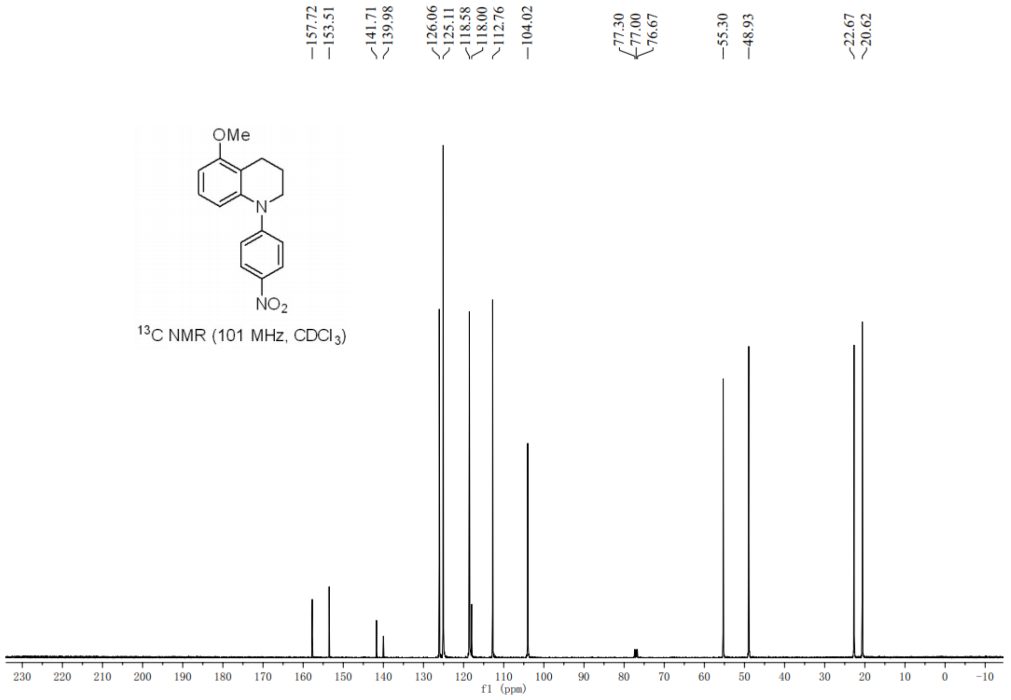

6-methoxy-1-(4-nitrophenyl)-1,2,3,4-tetrahydroquinoline (3a-9)

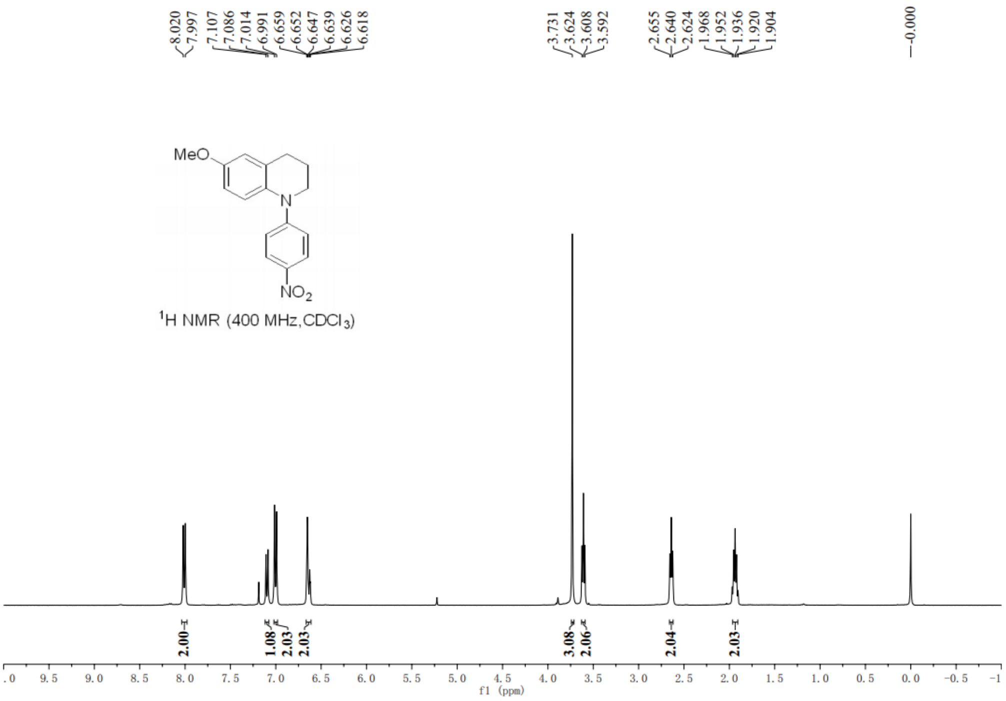

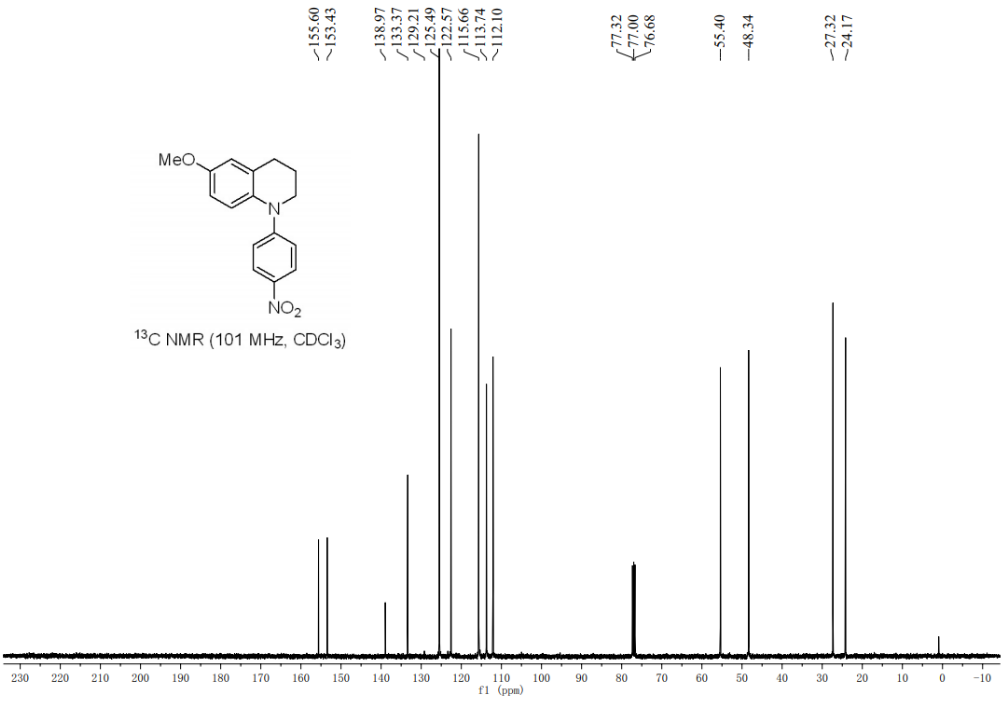

6-isopropoxy-1-(4-nitrophenyl)-1,2,3,4-tetrahydroquinoline (3a-10)

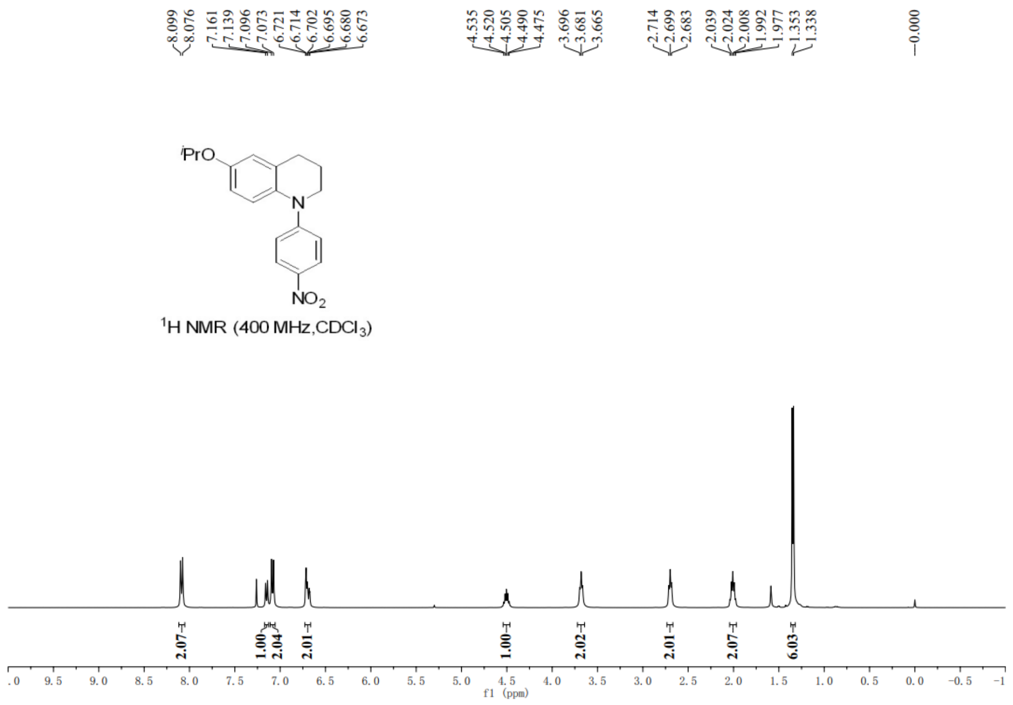

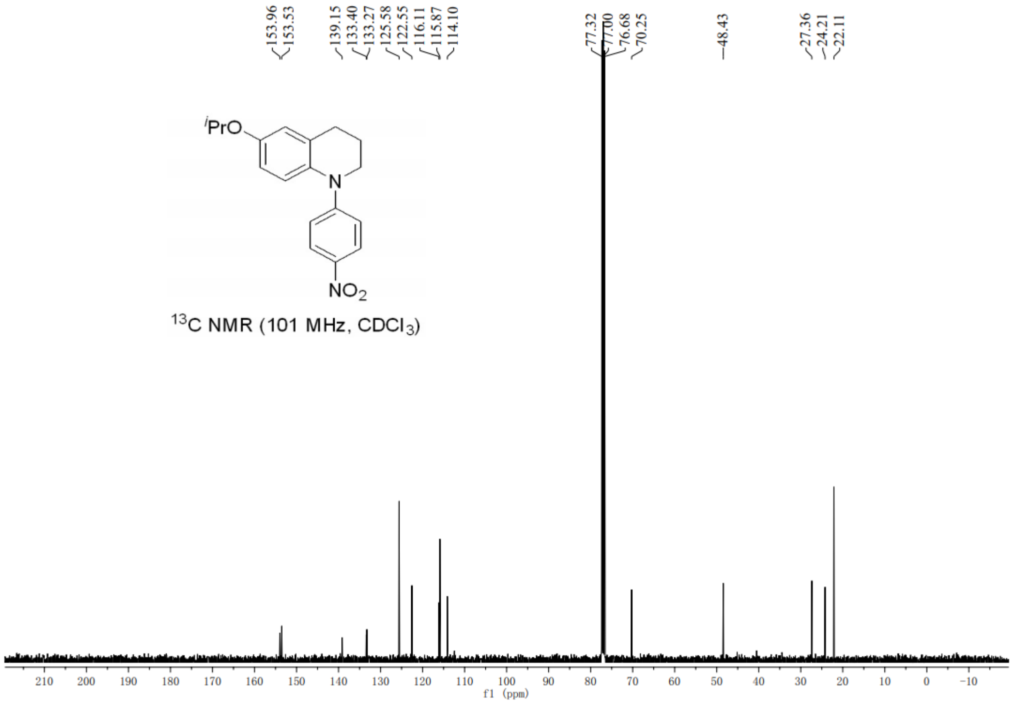

6-ethoxy-1-(4-nitrophenyl)-1,2,3,4-tetrahydroquinoline (3a-11)

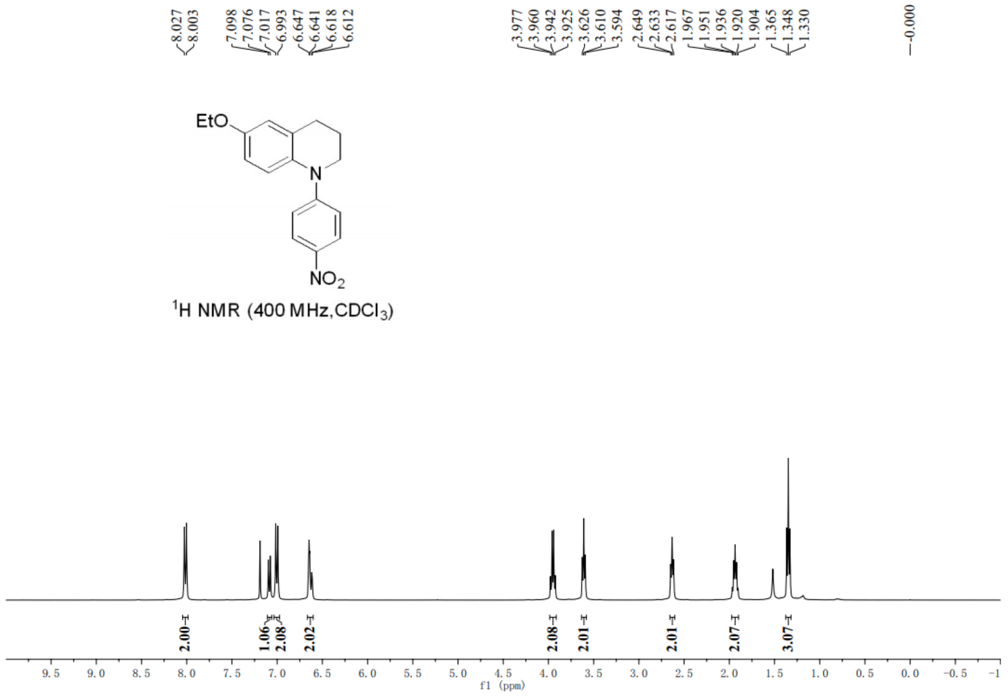

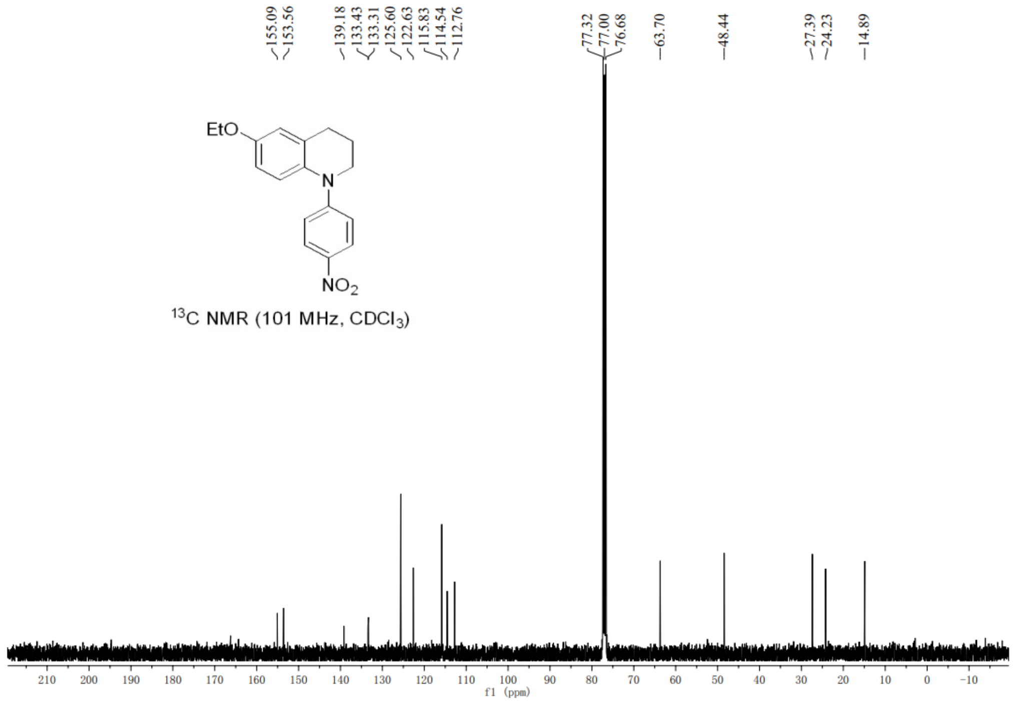

7-methoxy-1-(4-nitrophenyl)-1,2,3,4-tetrahydroquinoline (3a-12)

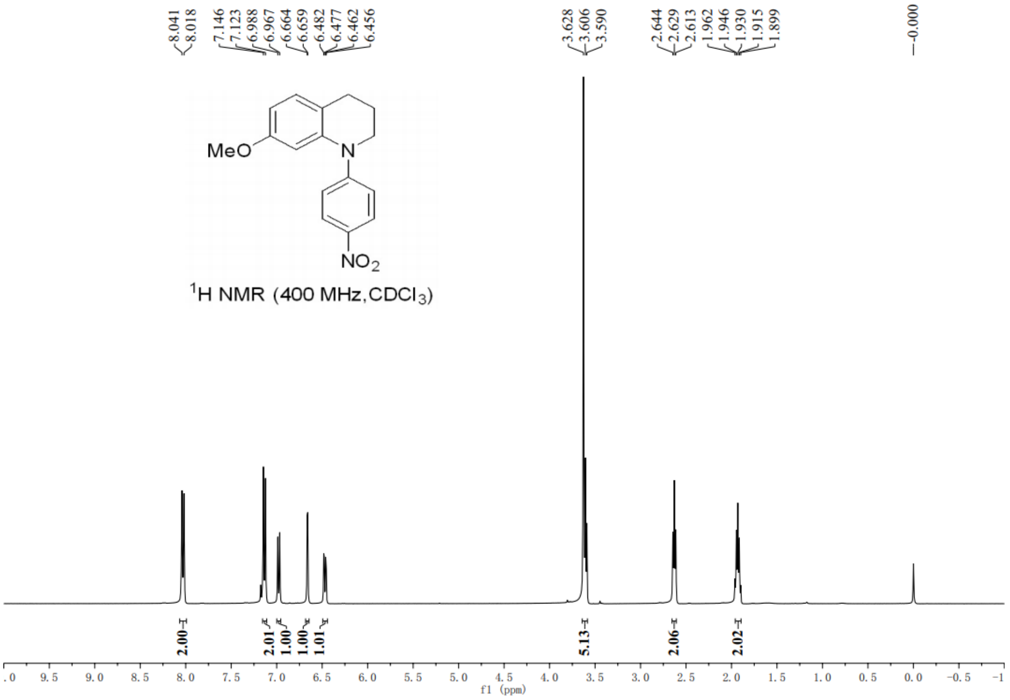

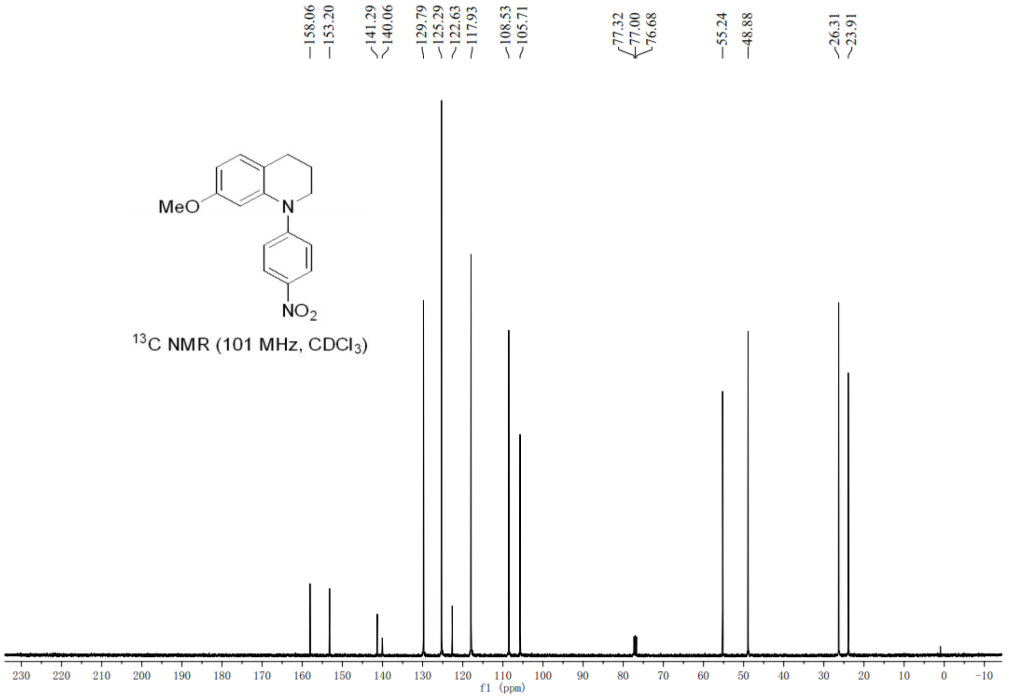

7-ethoxy-1-(4-nitrophenyl)-1,2,3,4-tetrahydroquinoline (3a-13)

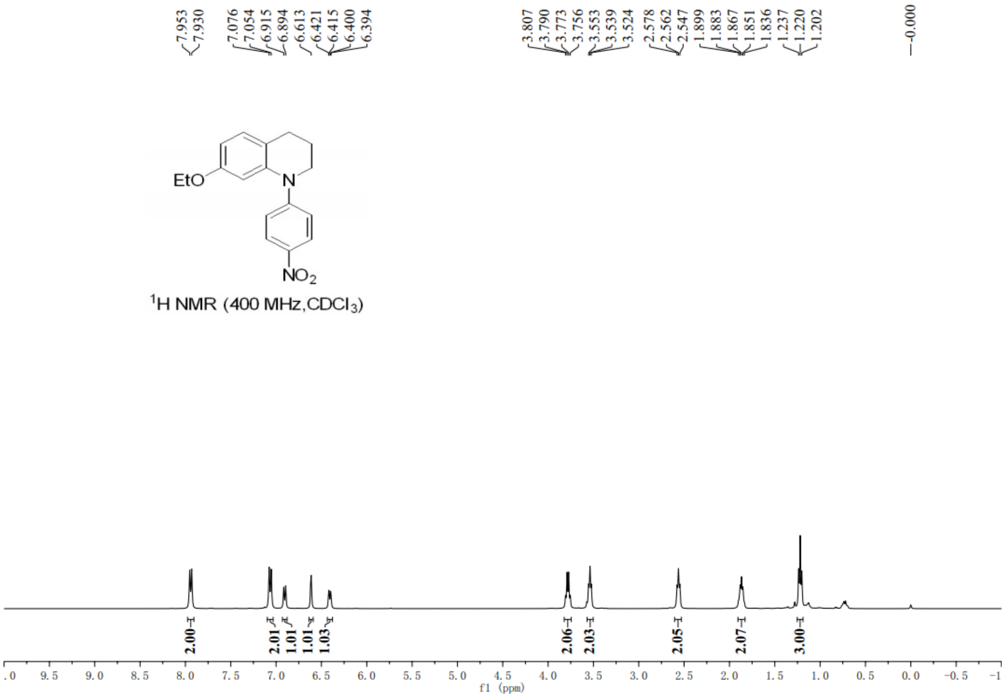

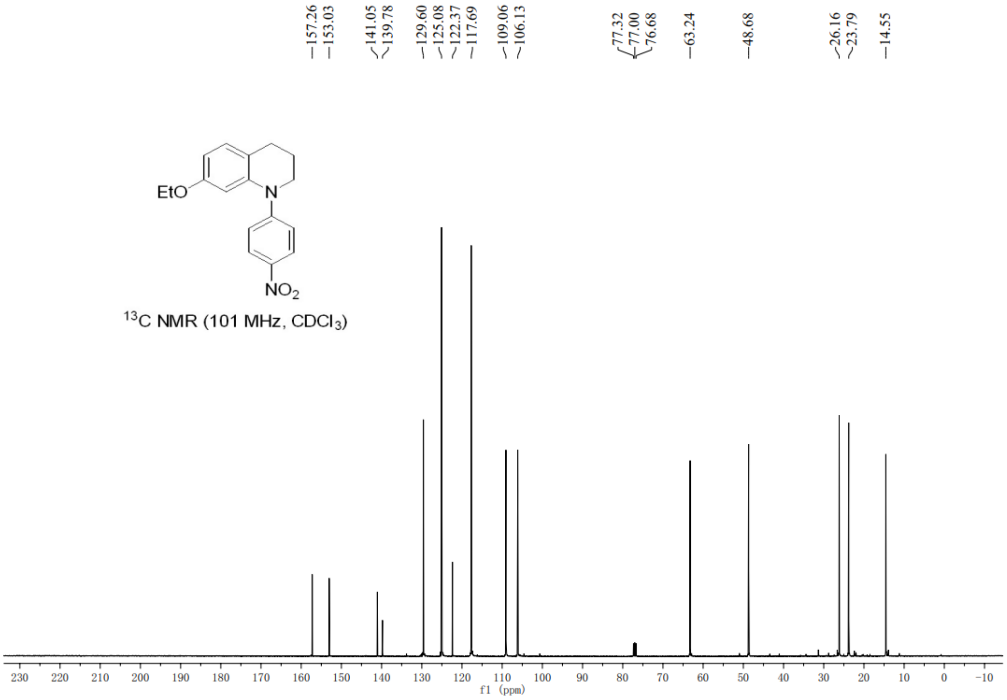

7-isopropoxy-1-(4-nitrophenyl)-1,2,3,4-tetrahydroquinoline (3a-14)

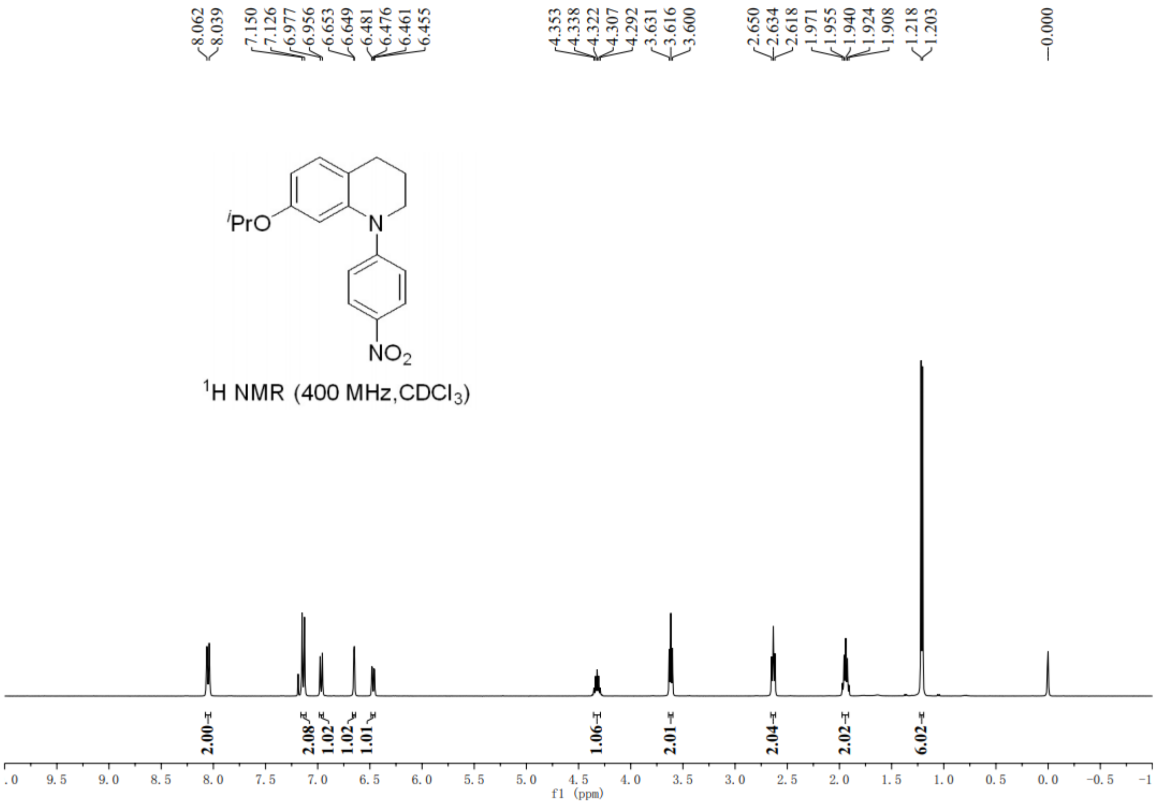

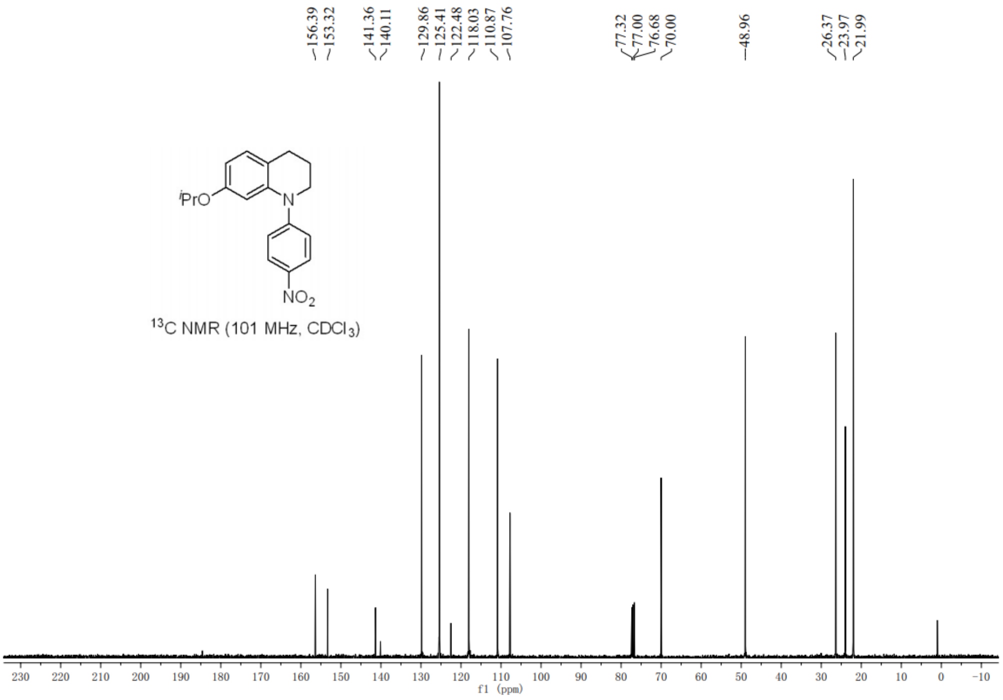

5-fluoro-1-(4-nitrophenyl)-1,2,3,4-tetrahydroquinoline (3a-15)

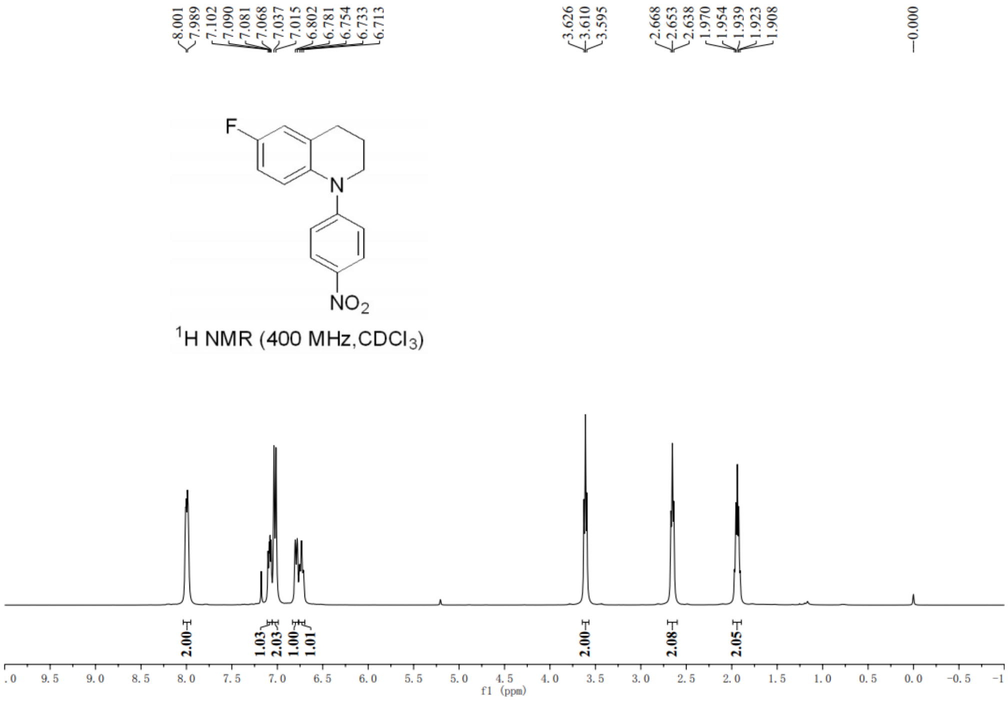

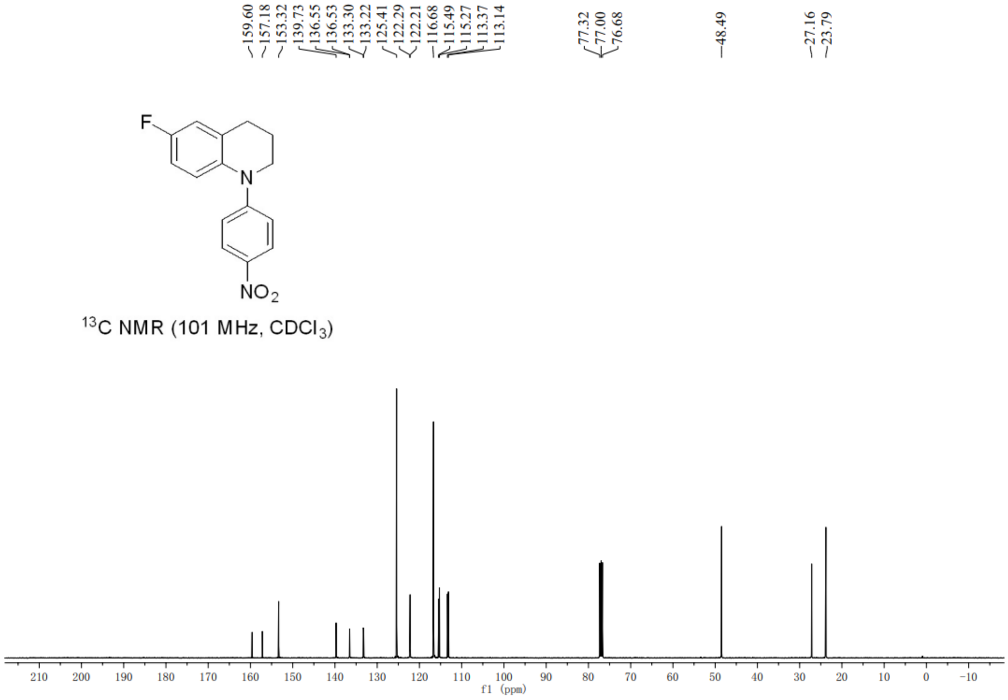

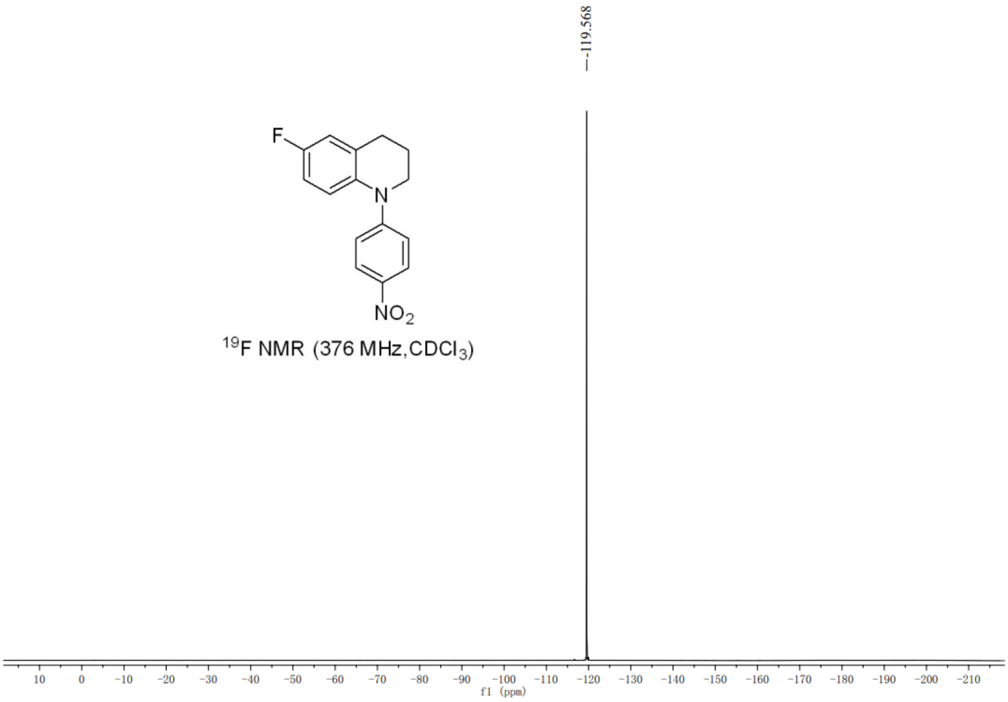
-119.568
F
N
NO2
19F NMR (376 MHz,CDCl3)
10 0 −10 −20 −30 −40 −50 −60 −70 −80 −90 −100 −110 −120 −130 −140 −150 −160 −170 −180 −190 −200 −210
f1 (ppm)

6-chloro-1-(4-nitrophenyl)-1,2,3,4-tetrahydroquinoline (3a-16)

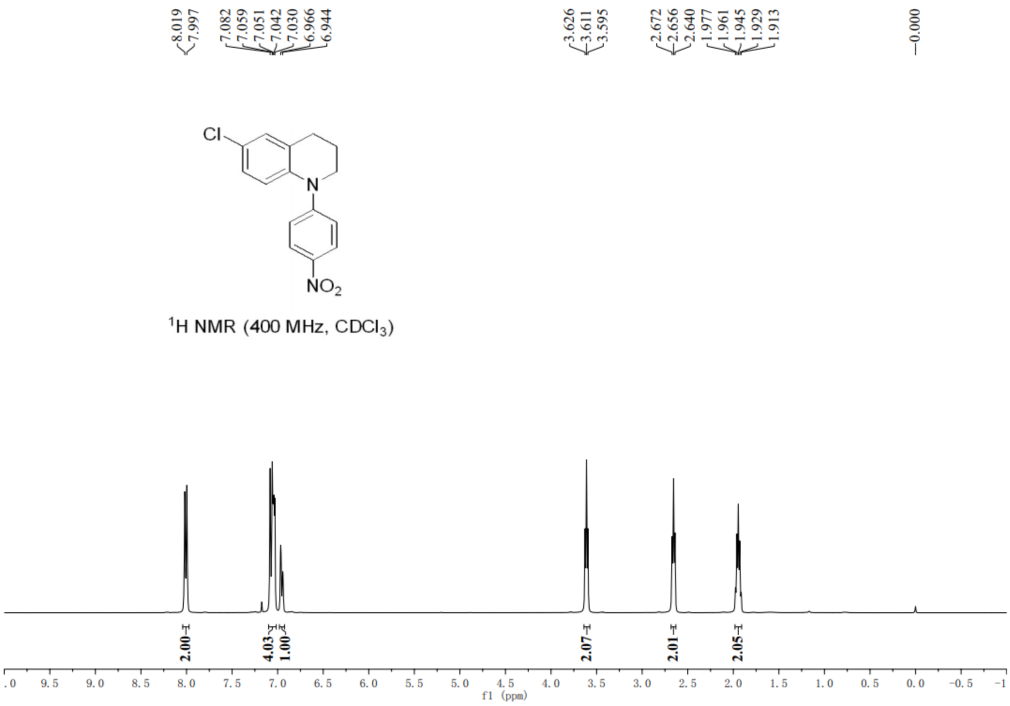

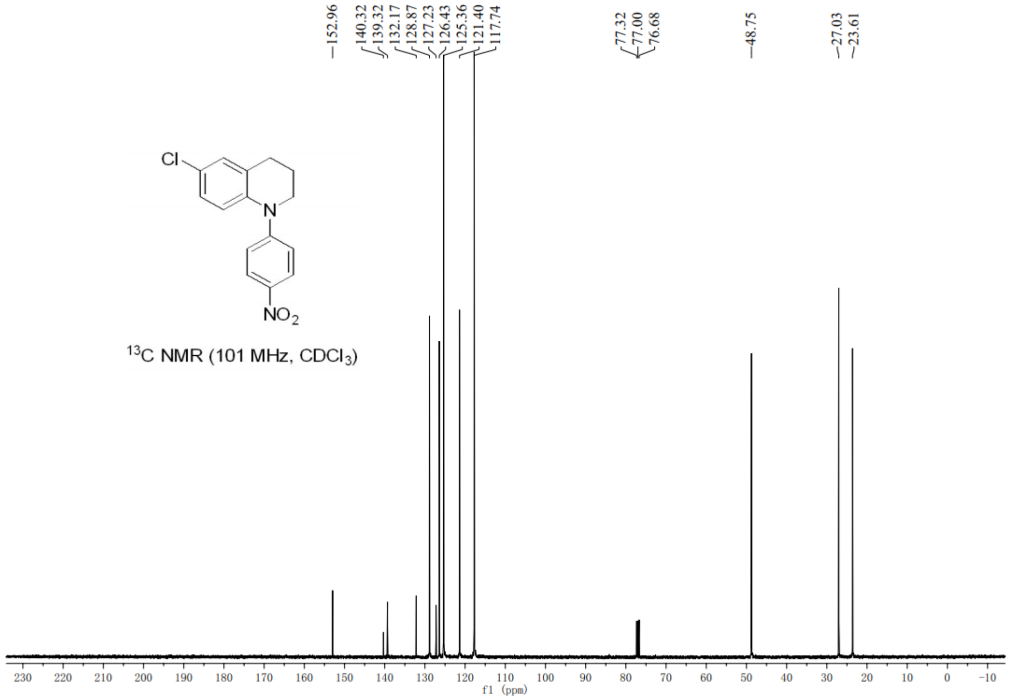

6-bromo-1-(4-nitrophenyl)-1,2,3,4-tetrahydroquinoline (3a-17)

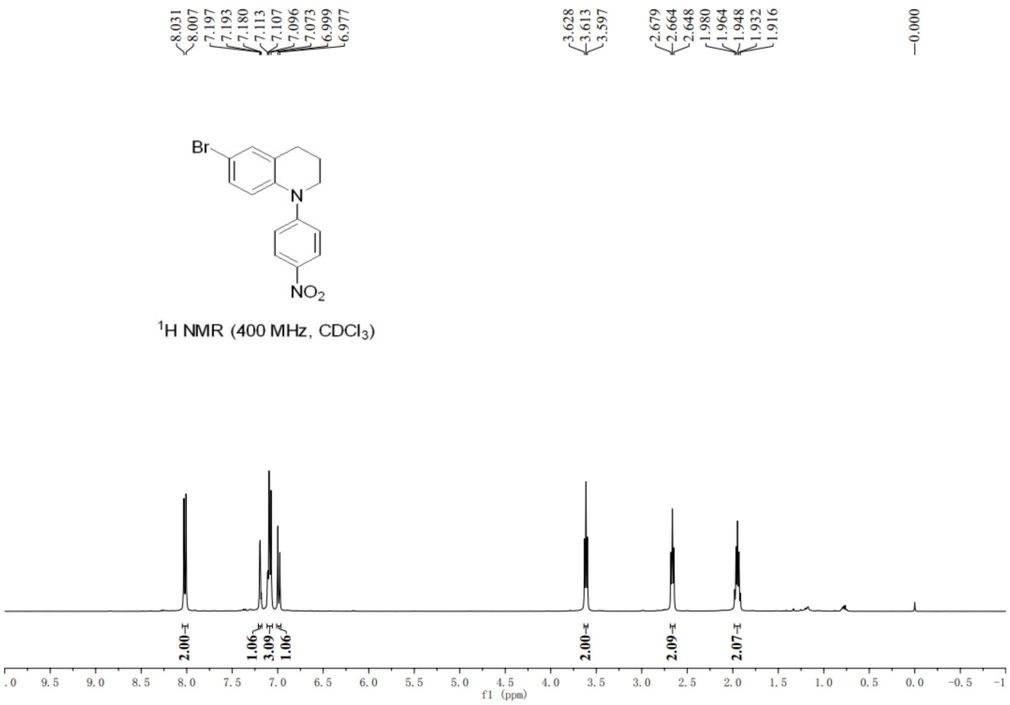

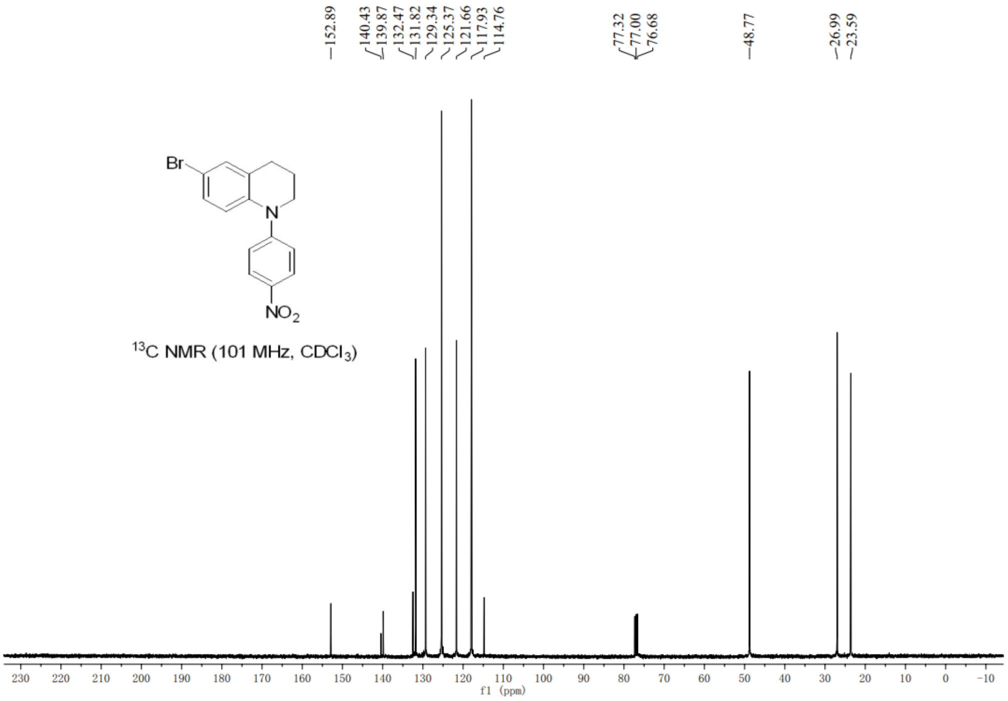

5-chloro-1-(4-nitrophenyl)-1,2,3,4-tetrahydroquinoline (3a-18)

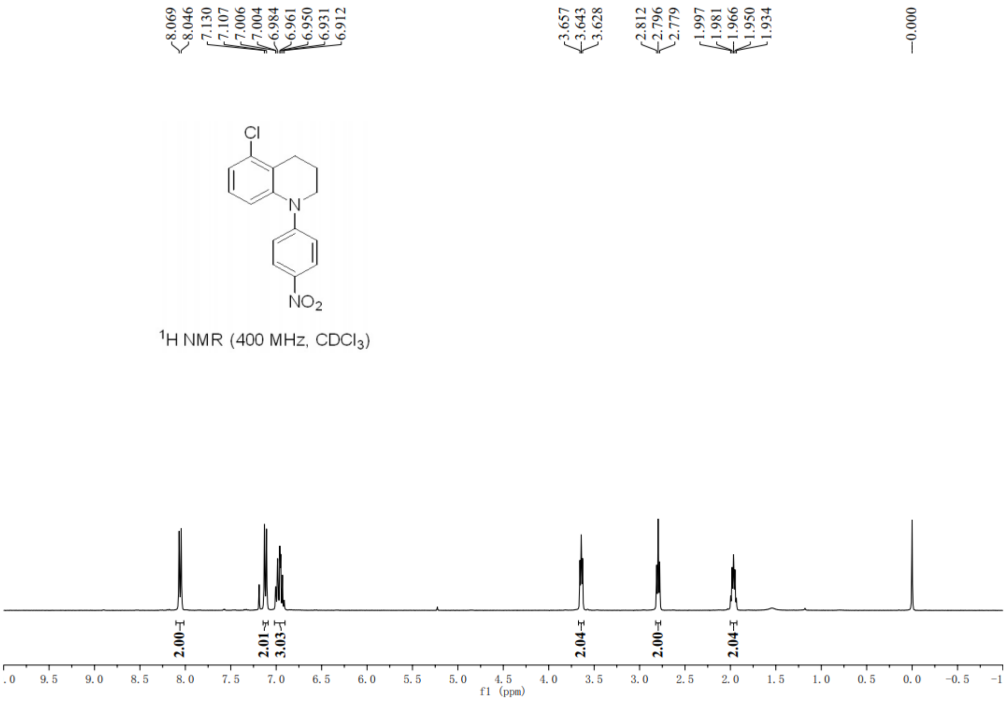

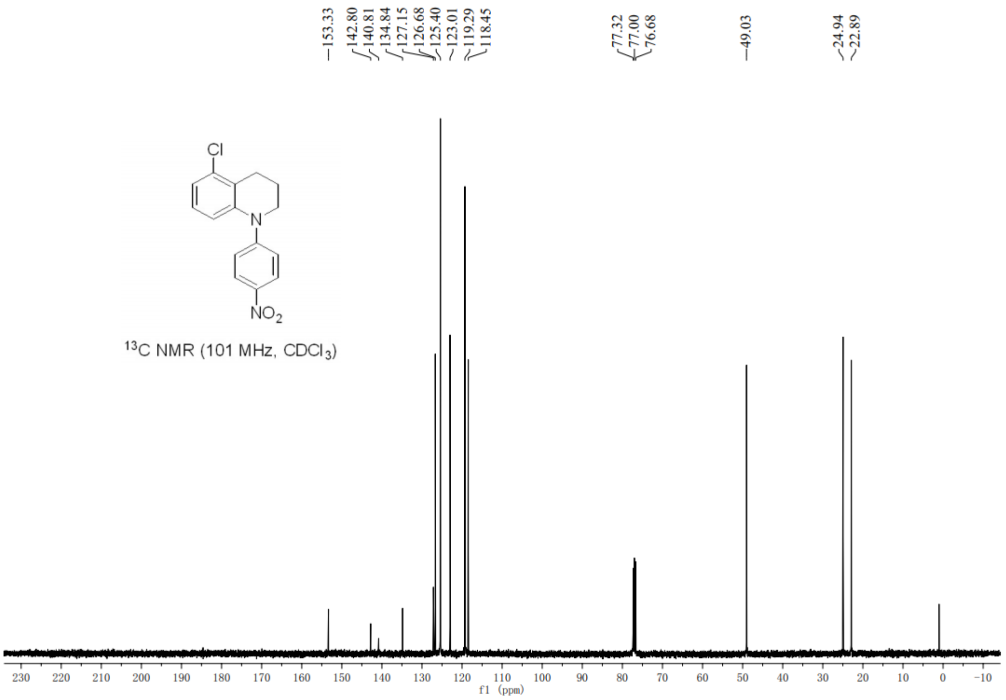

5-bromo-1-(4-nitrophenyl)-1,2,3,4-tetrahydroquinoline (3a-19)

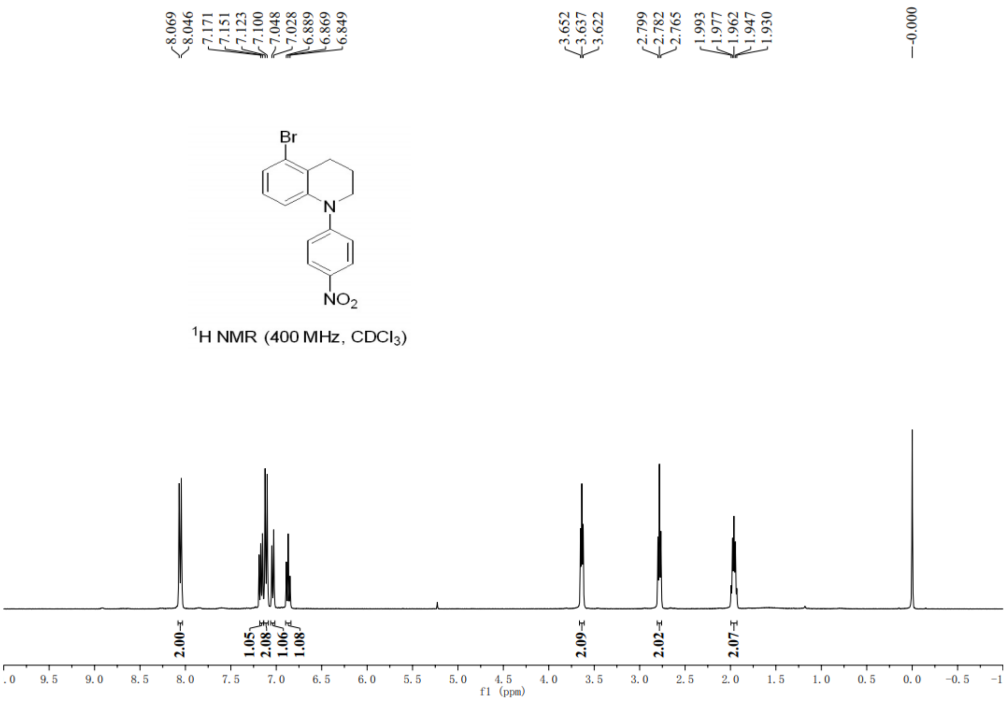

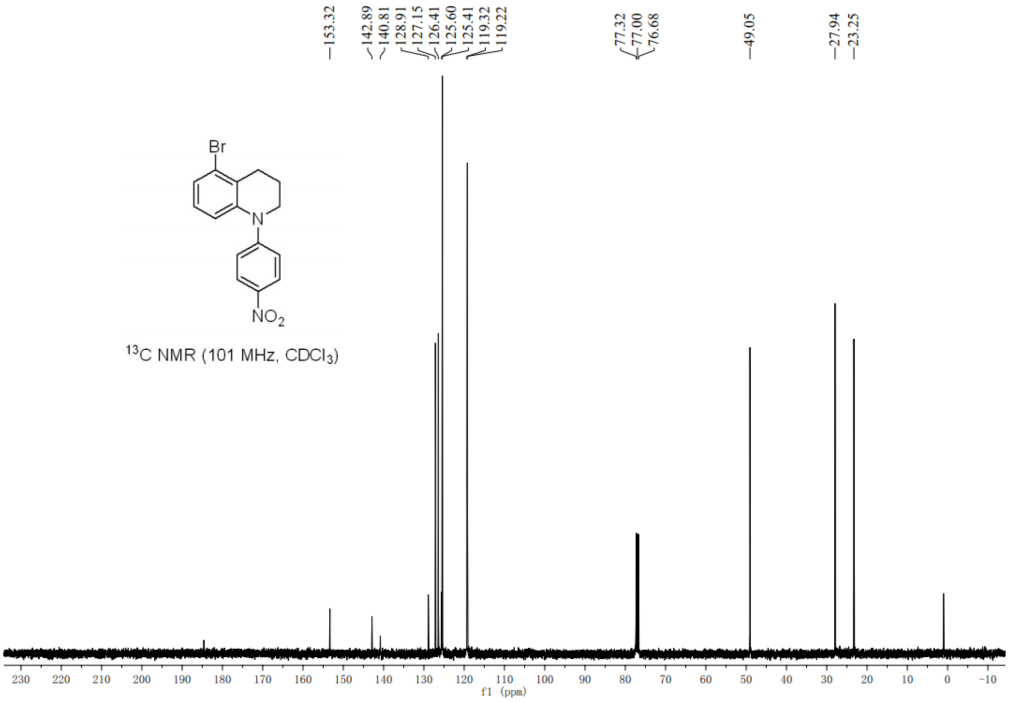

7-chloro-1-(4-nitrophenyl)-1,2,3,4-tetrahydroquinoline (3a-20)

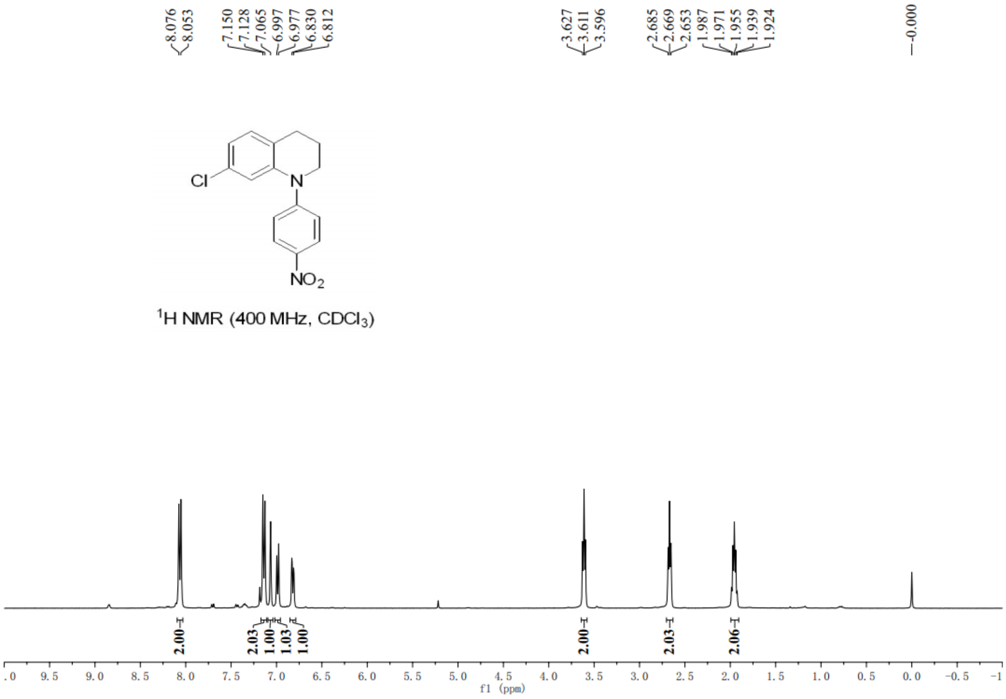

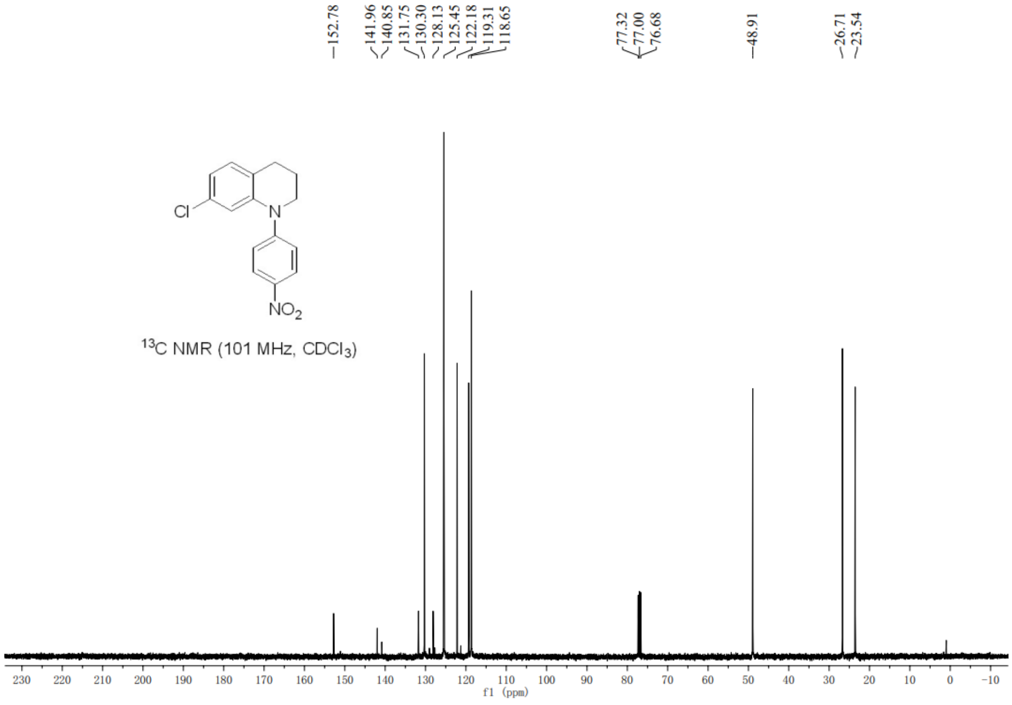

7-bromo-1-(4-nitrophenyl)-1,2,3,4-tetrahydroquinoline (3a-21)

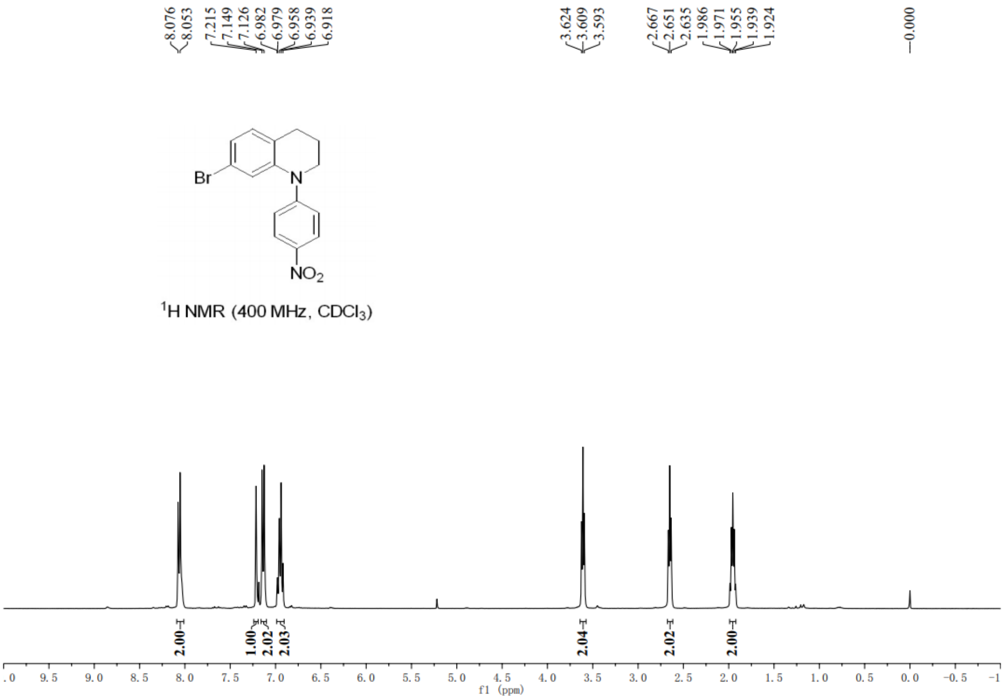

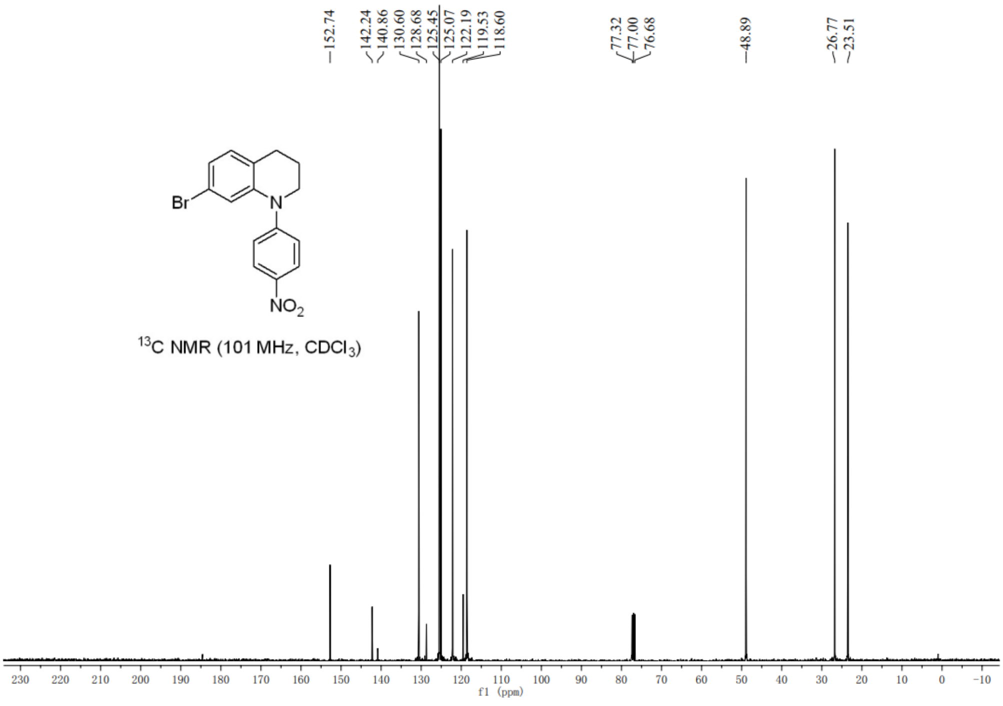

1-(4-nitrophenyl)-1,2,3,4-tetrahydroquinolin-7-ol (3a-22)

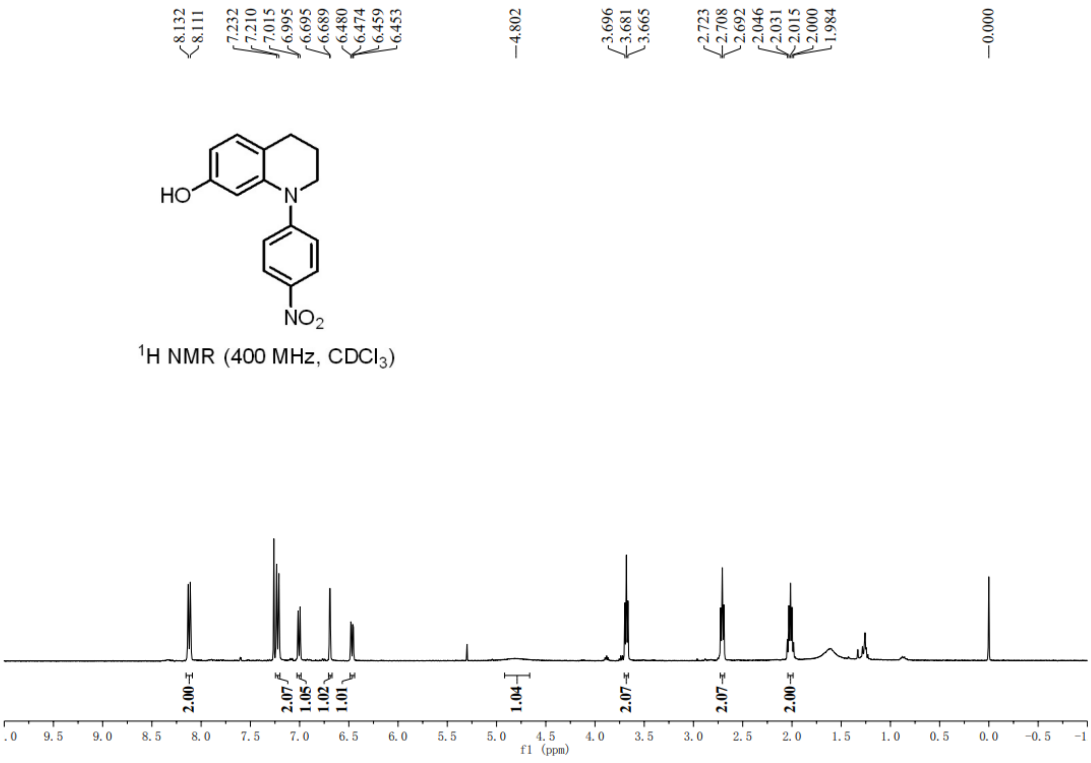

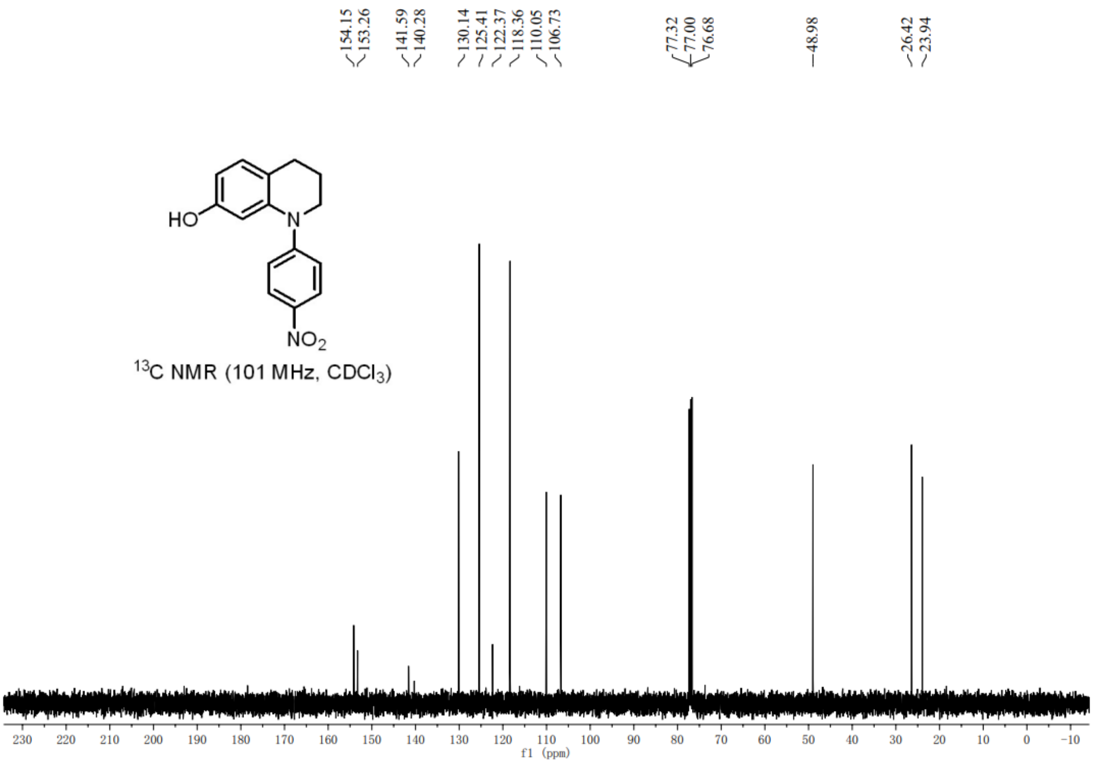

1-(4-nitrophenyl)-1,2,3,4-tetrahydroquinoline-6-carboxylic acid (3a-23)

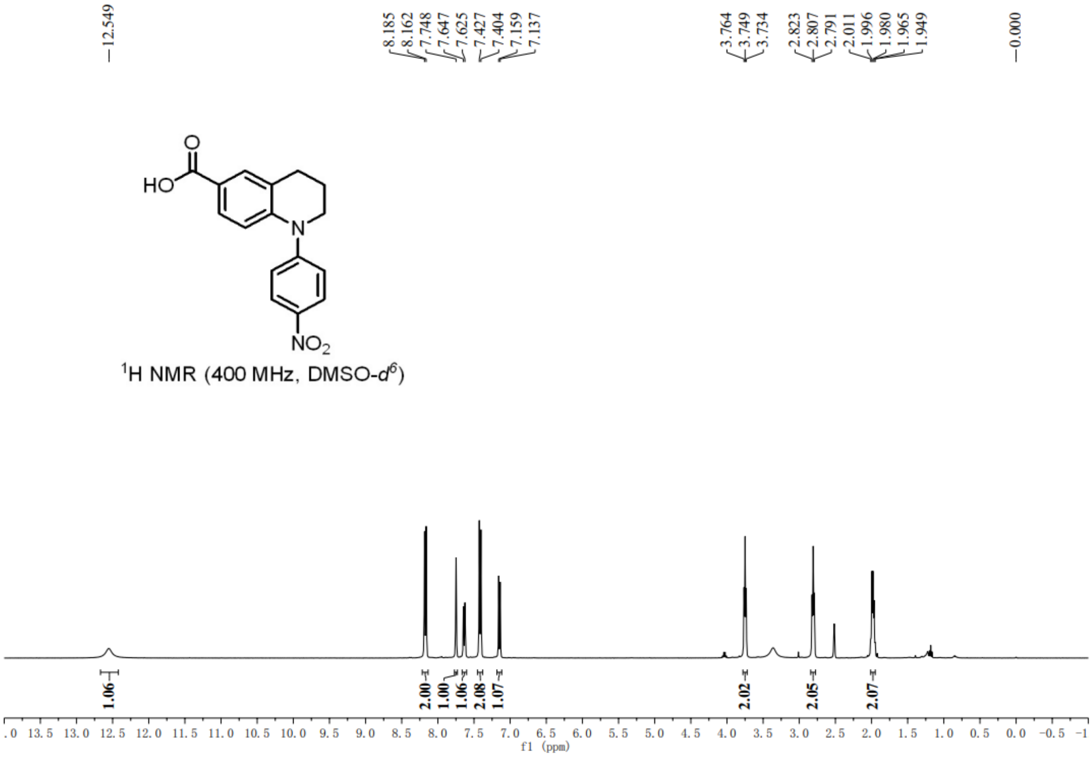

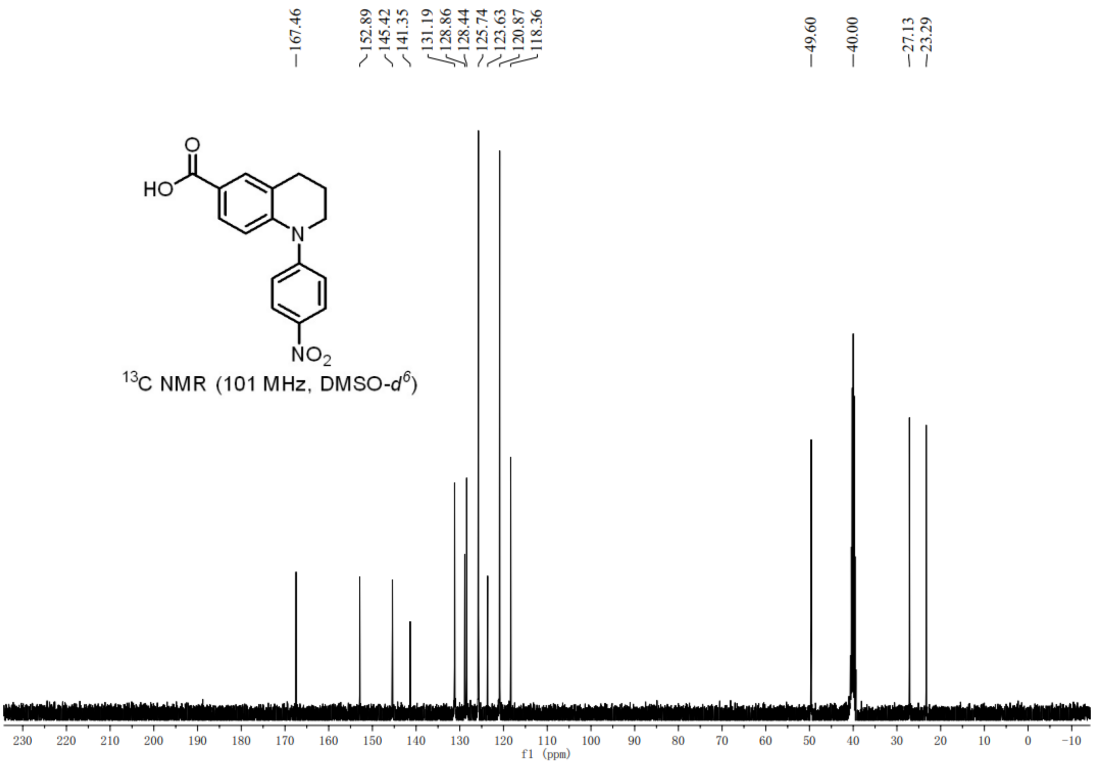

1-(4-nitrophenyl)-1,2,3,4-tetrahydroquinoline-3-carboxylic acid (3a-24)

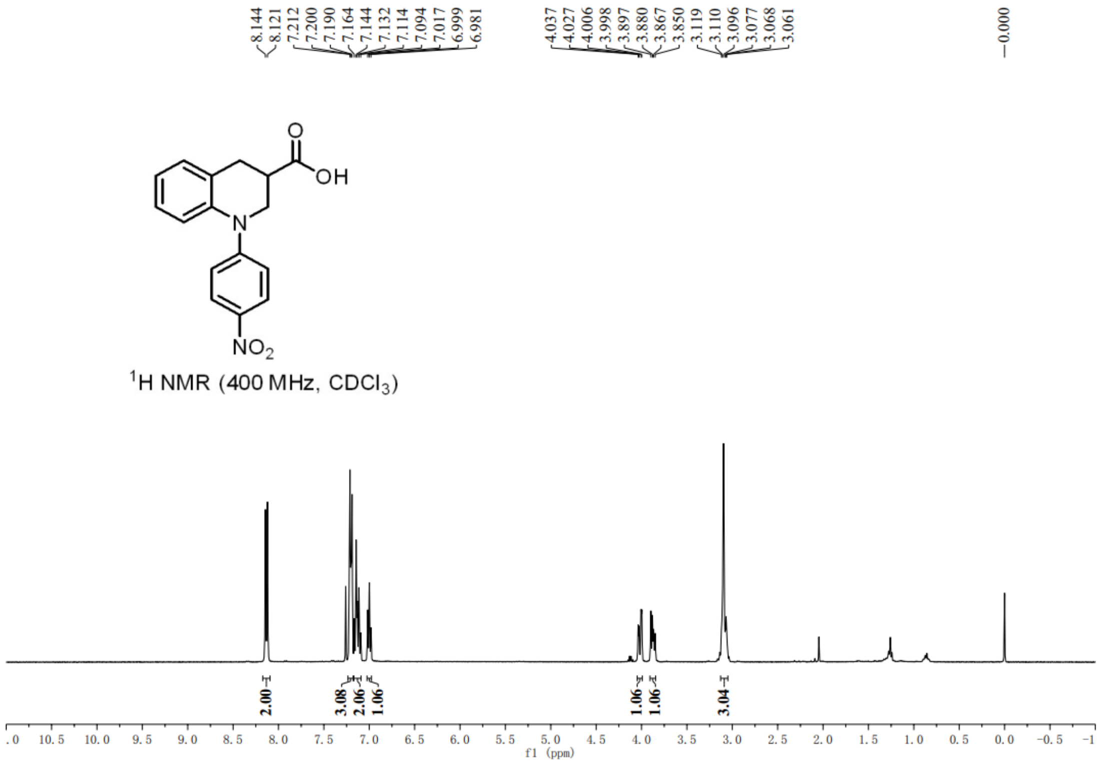

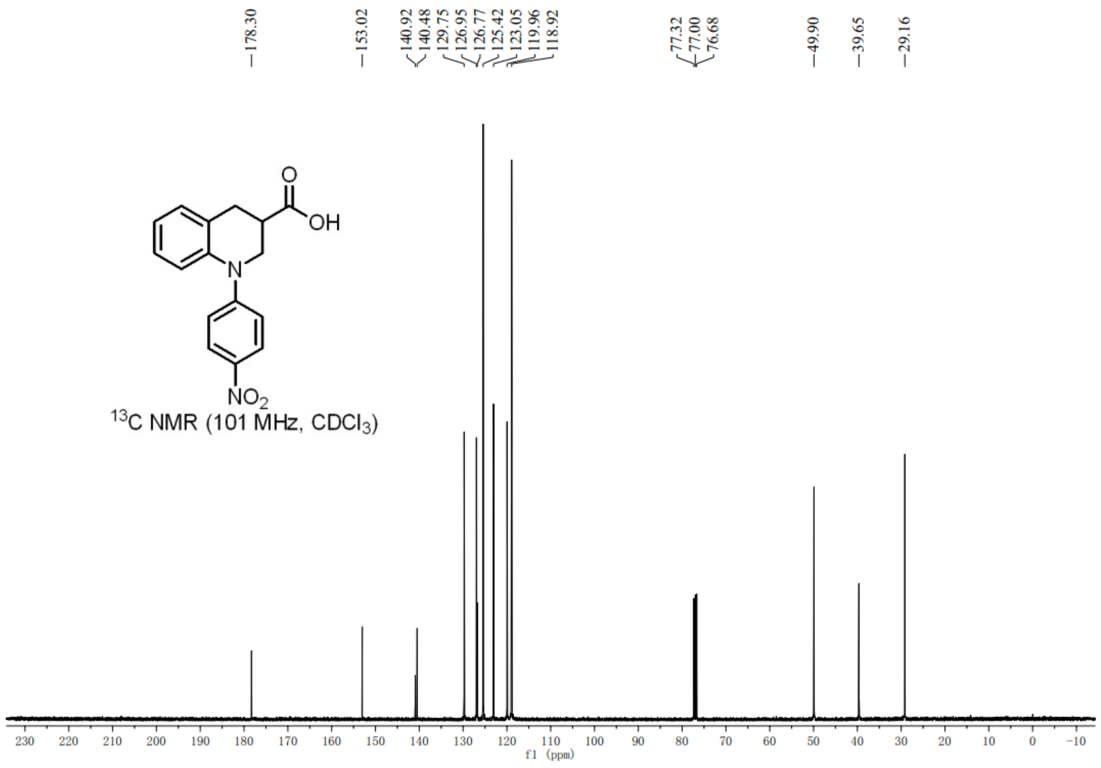

1-(4-nitrophenyl) indoline (3b-1)

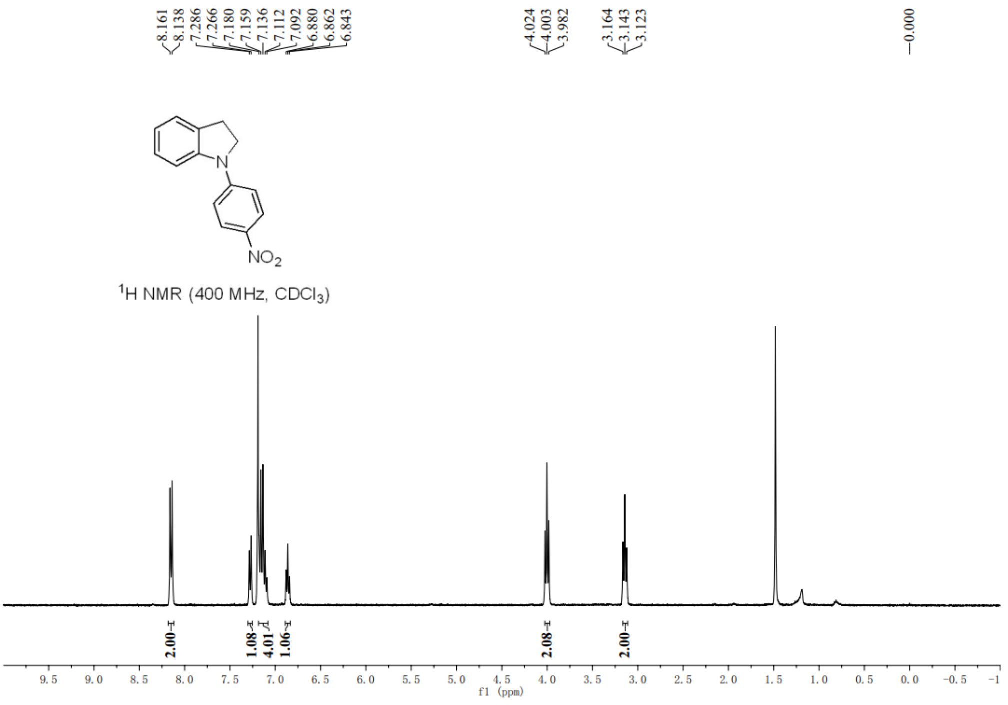

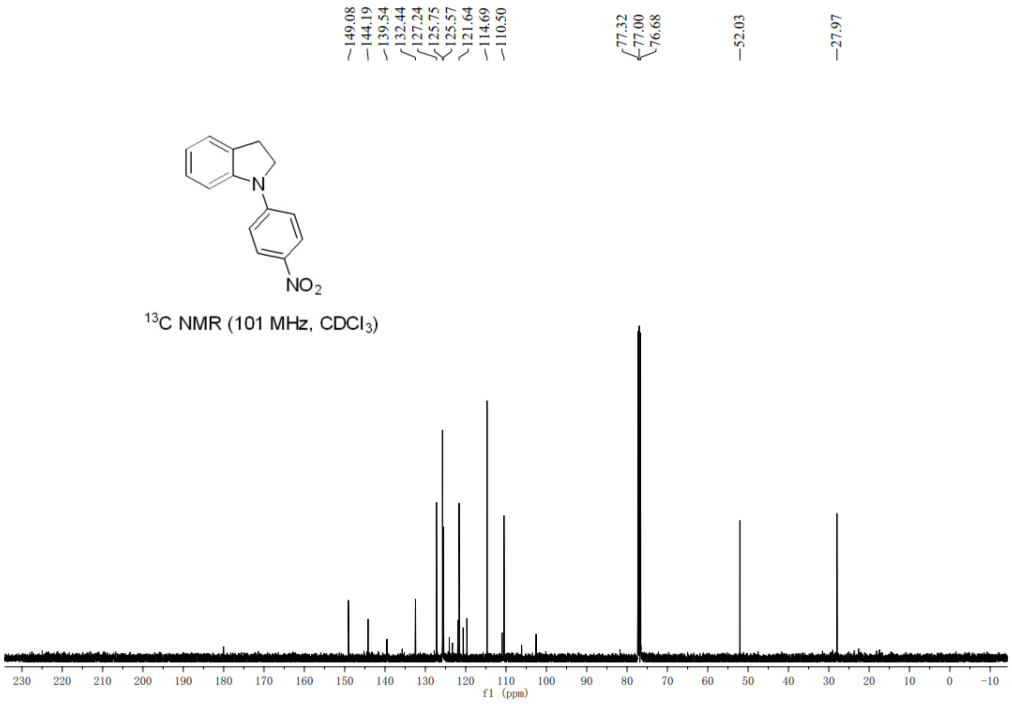

2-methyl-1-(4-nitrophenyl) indoline (3b-2)

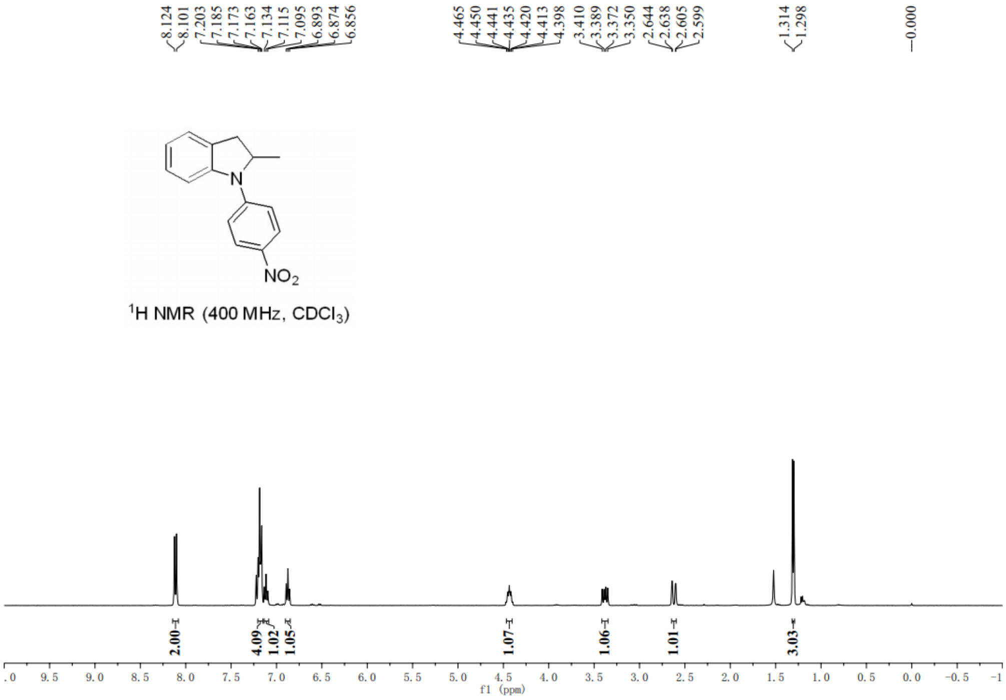

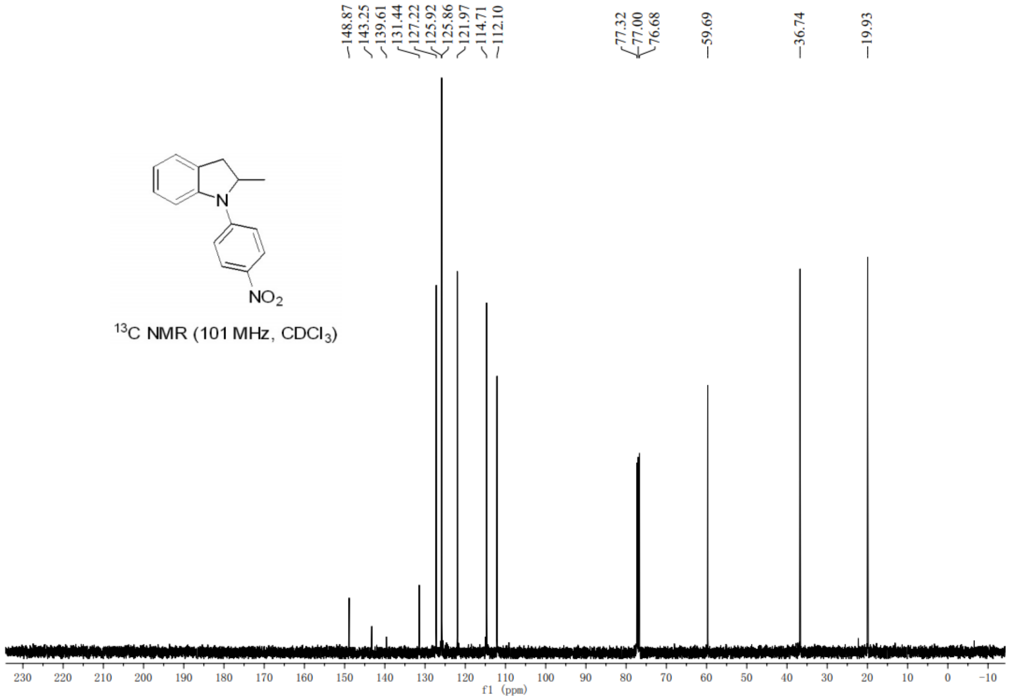

3-methyl-1-(4-nitrophenyl) indoline (3b-3)

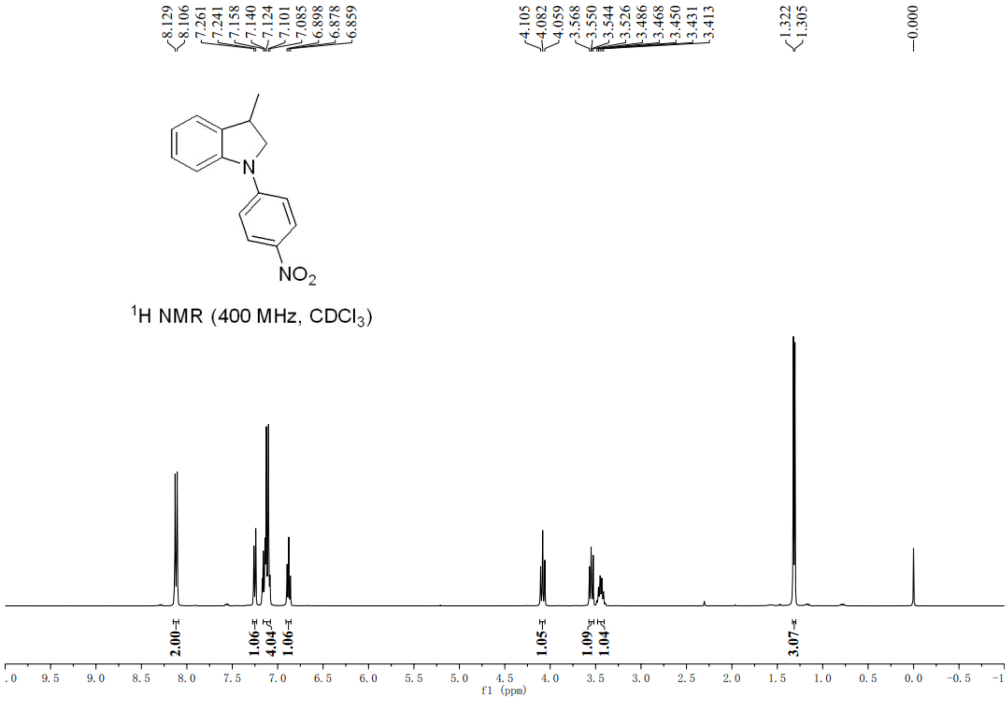

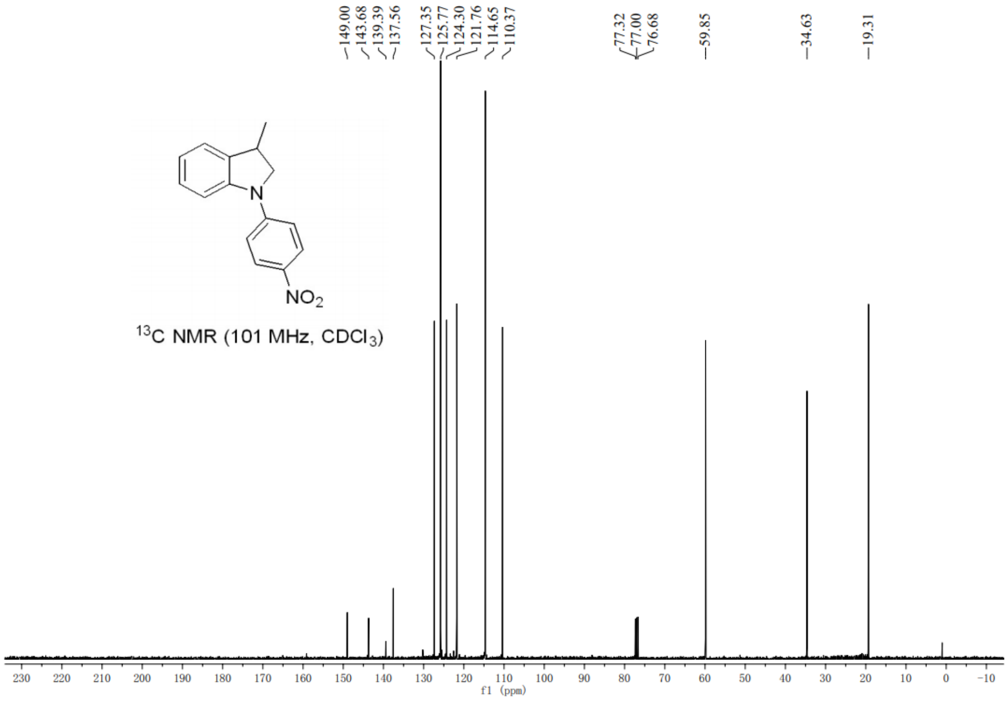

4-methyl-1-(4-nitrophenyl) indoline (3b-4)

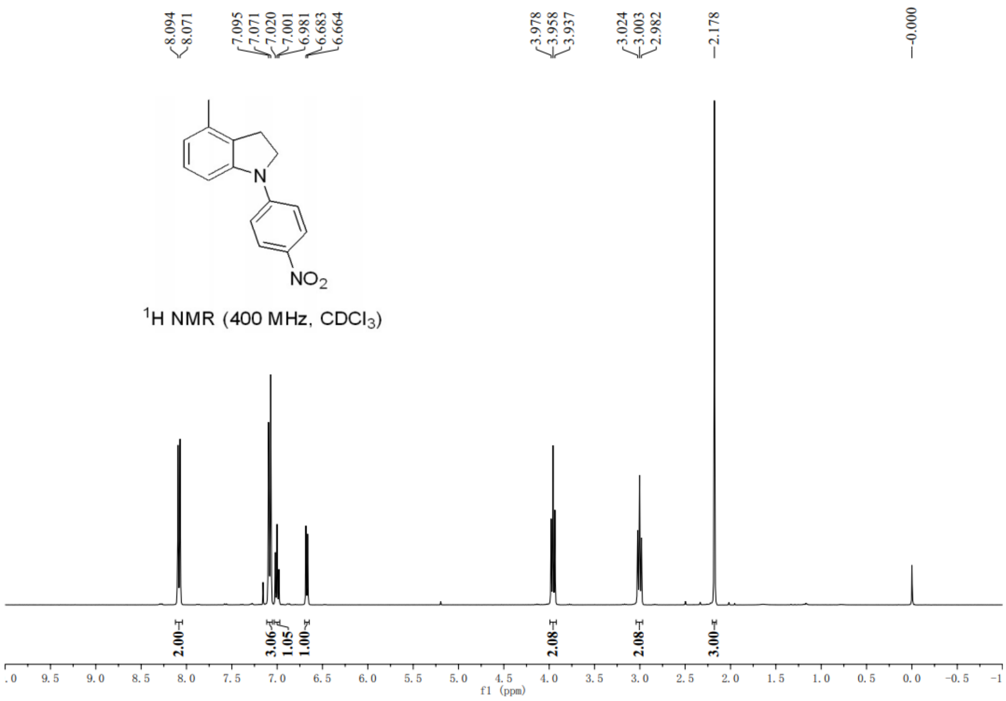

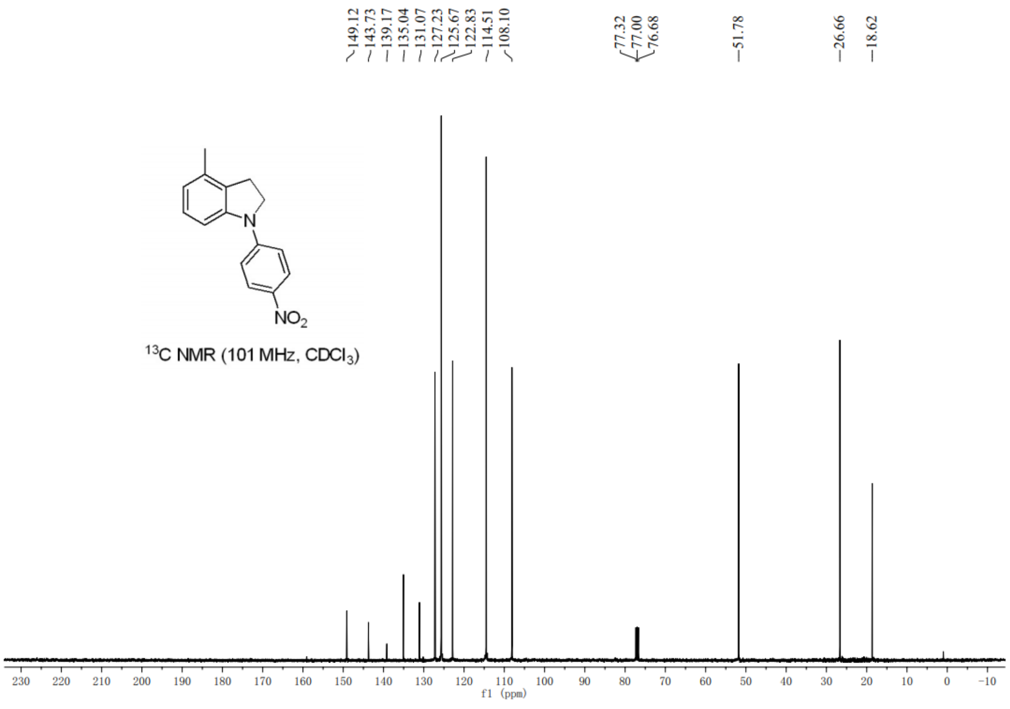

5-methyl-1-(4-nitrophenyl) indoline (3b-5)

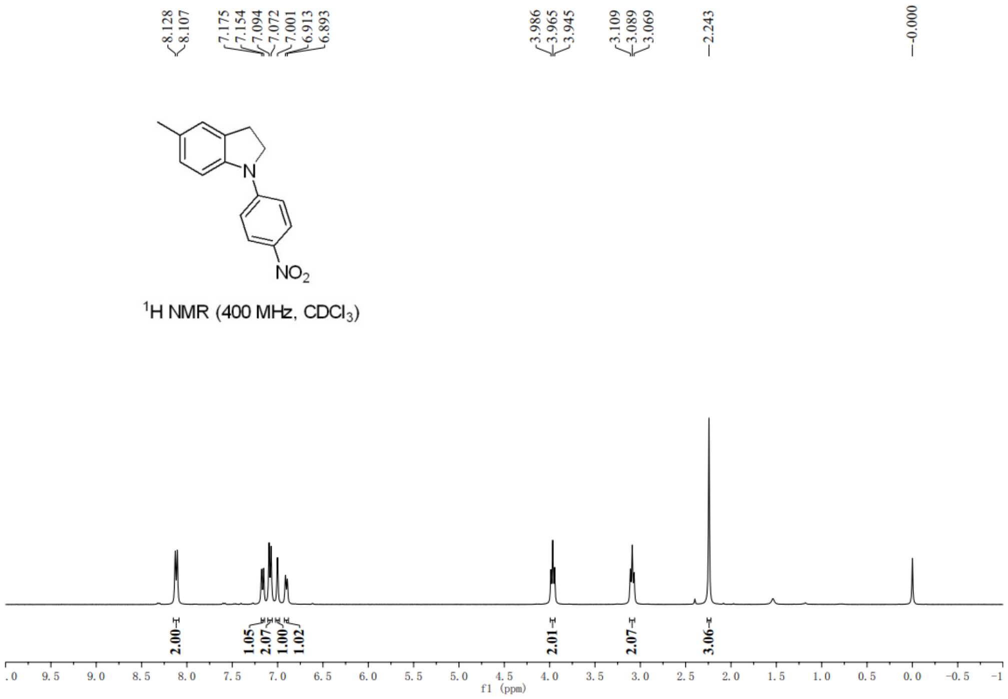

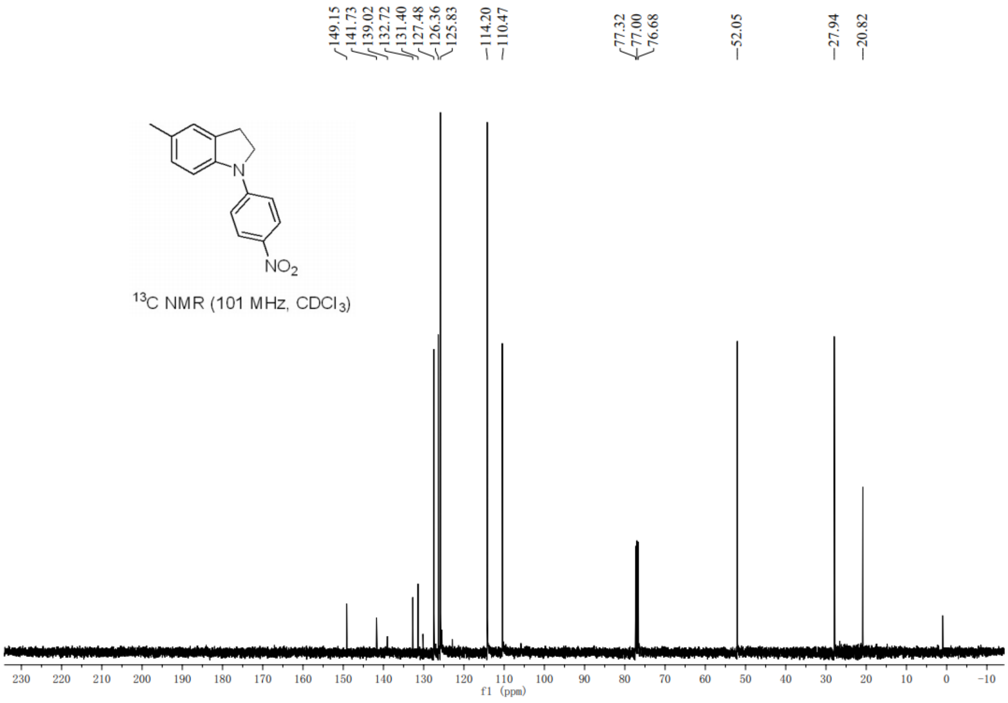

6-methyl-1-(4-nitrophenyl) indoline (3b-6)

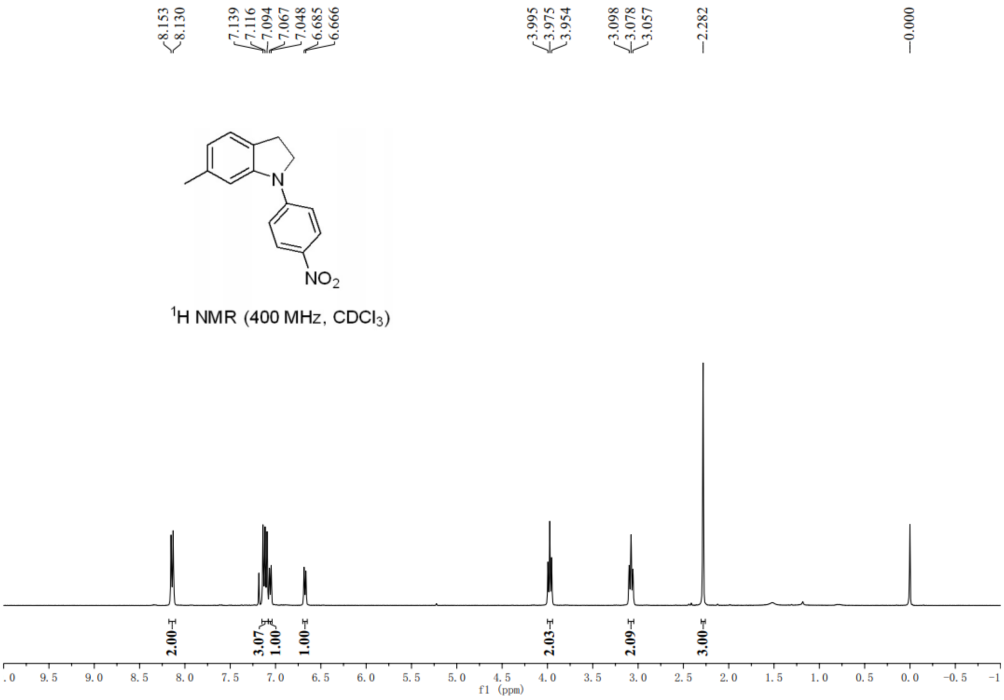

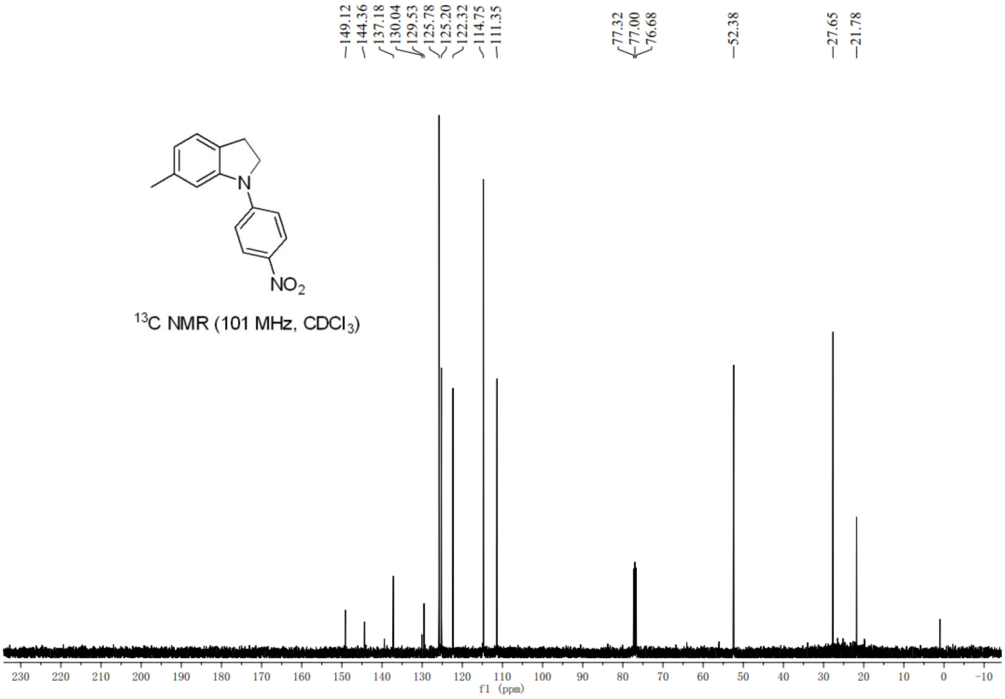

2,3-dimethyl-1-(4-nitrophenyl) indoline (3b-7)

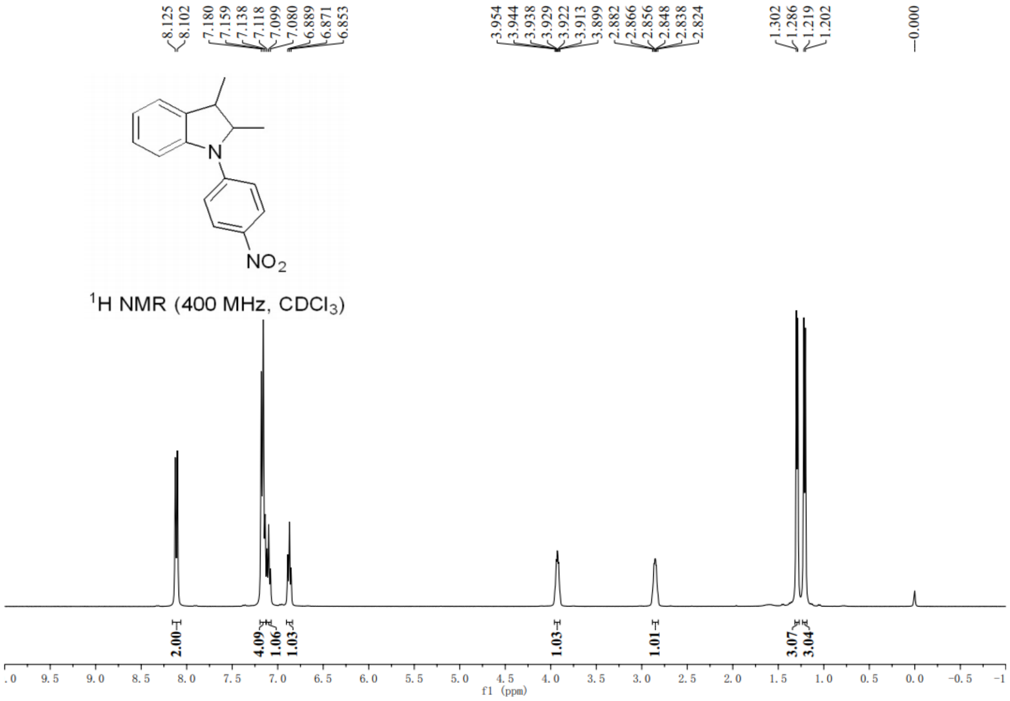

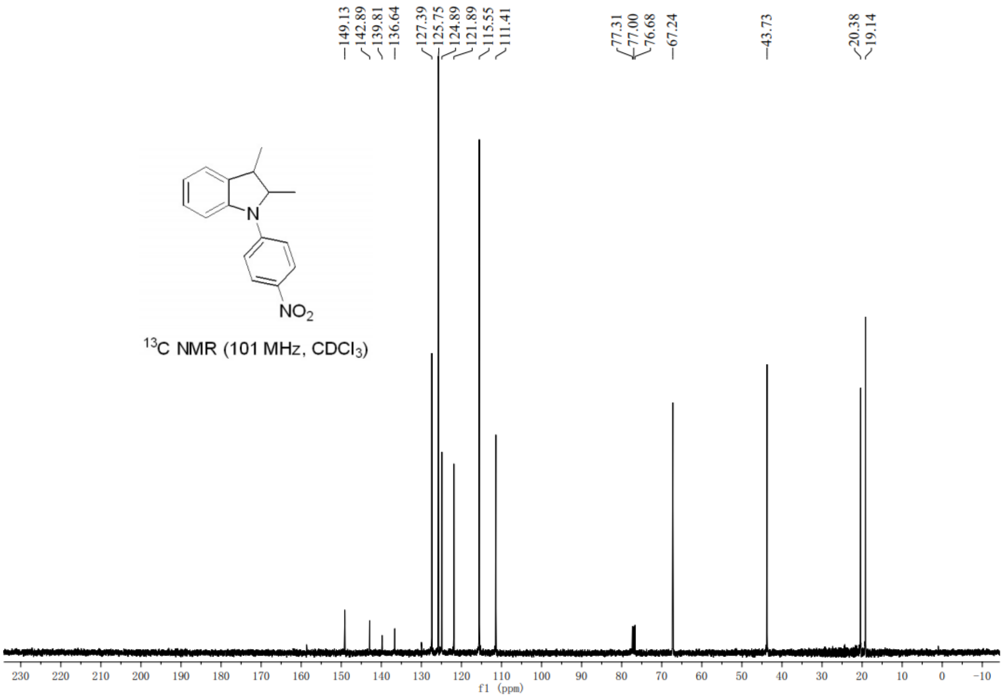

2,5-dimethyl-1-(4-nitrophenyl) indoline (3b-8)

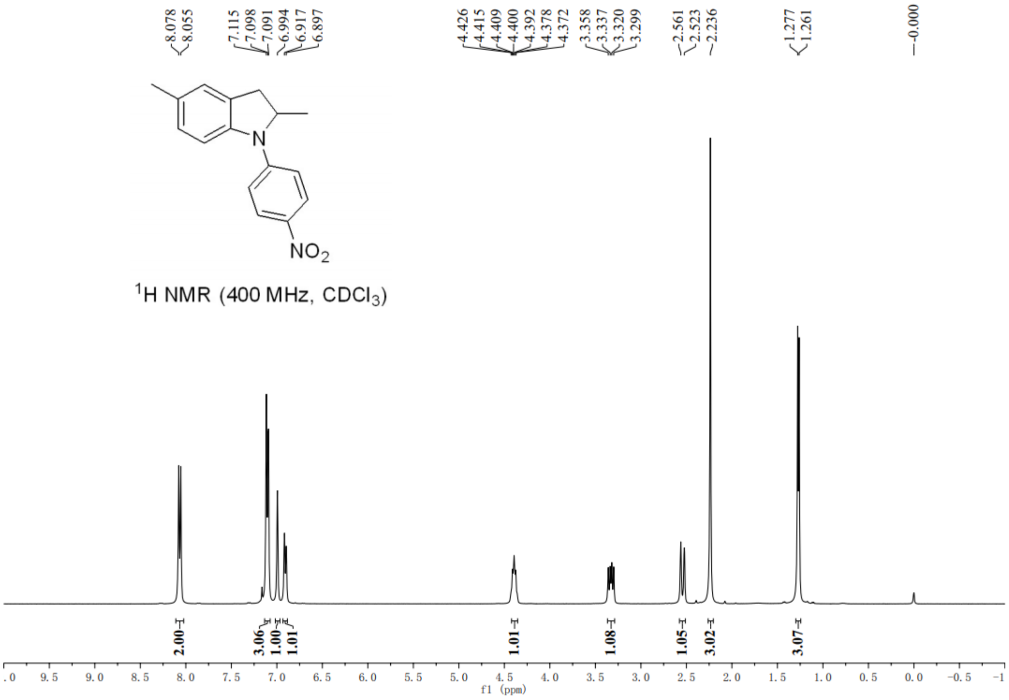

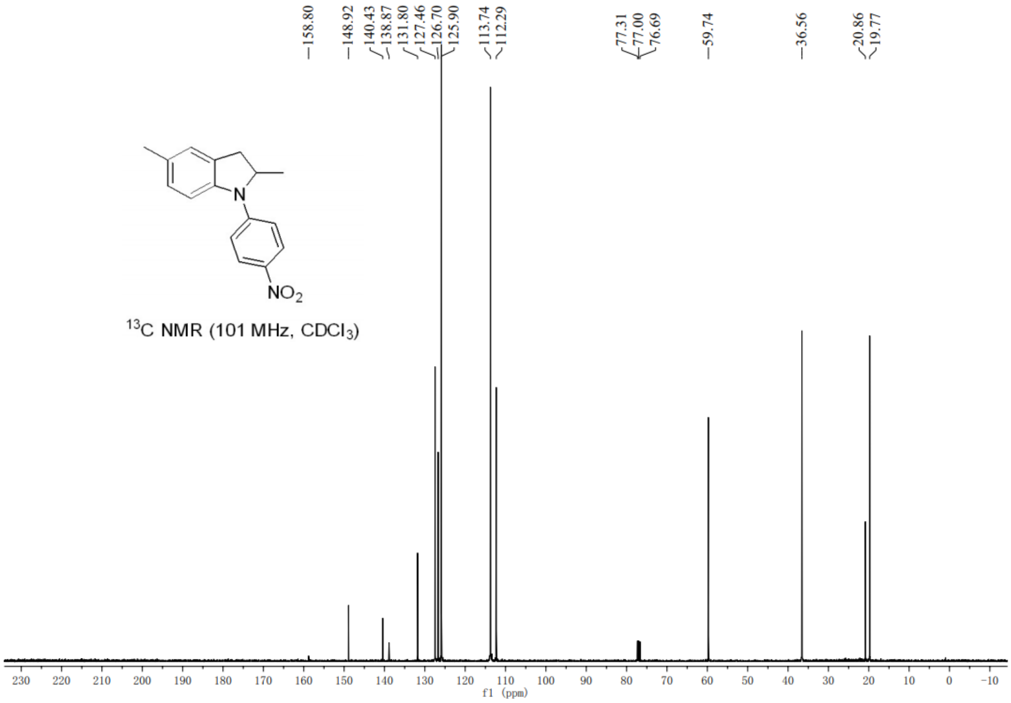

4-methoxy-1-(4-nitrophenyl) indoline (3b-9)

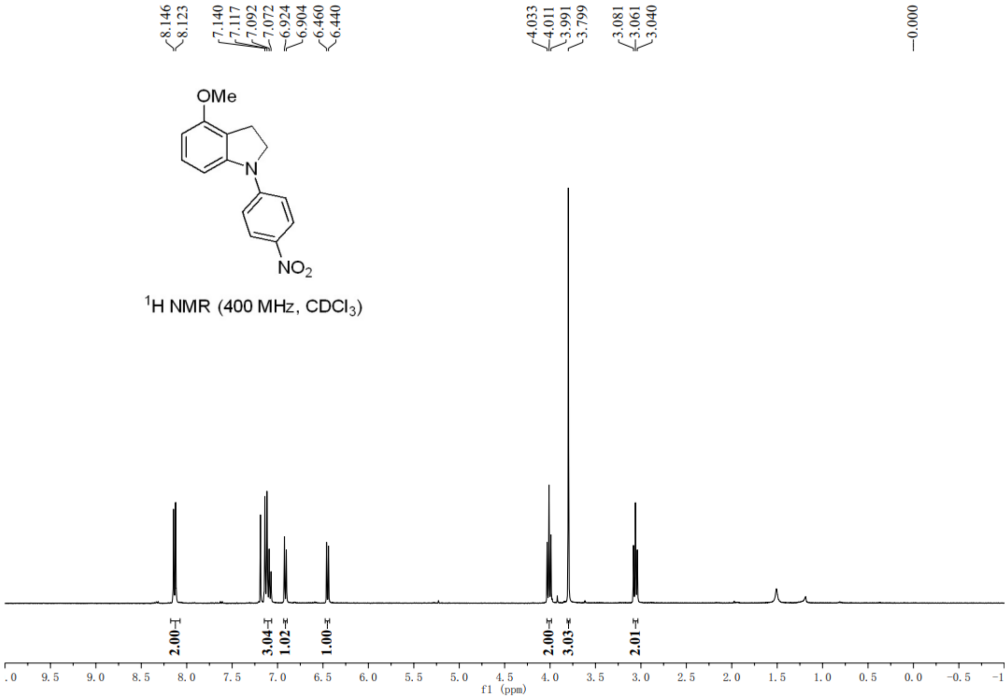

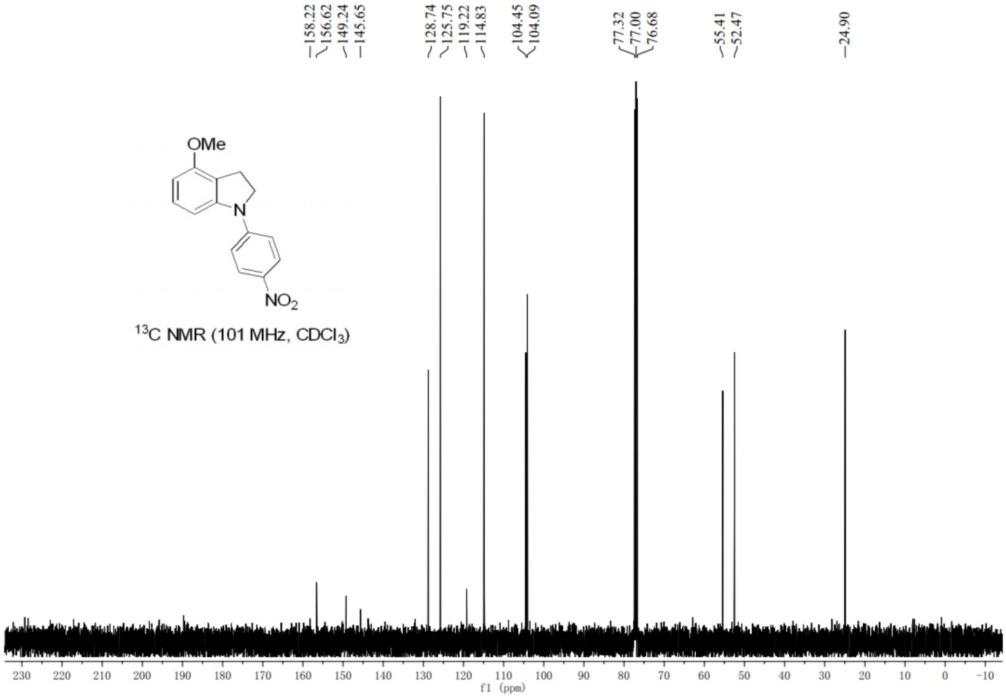

5-methoxy-1-(4-nitrophenyl) indoline (3b-10)

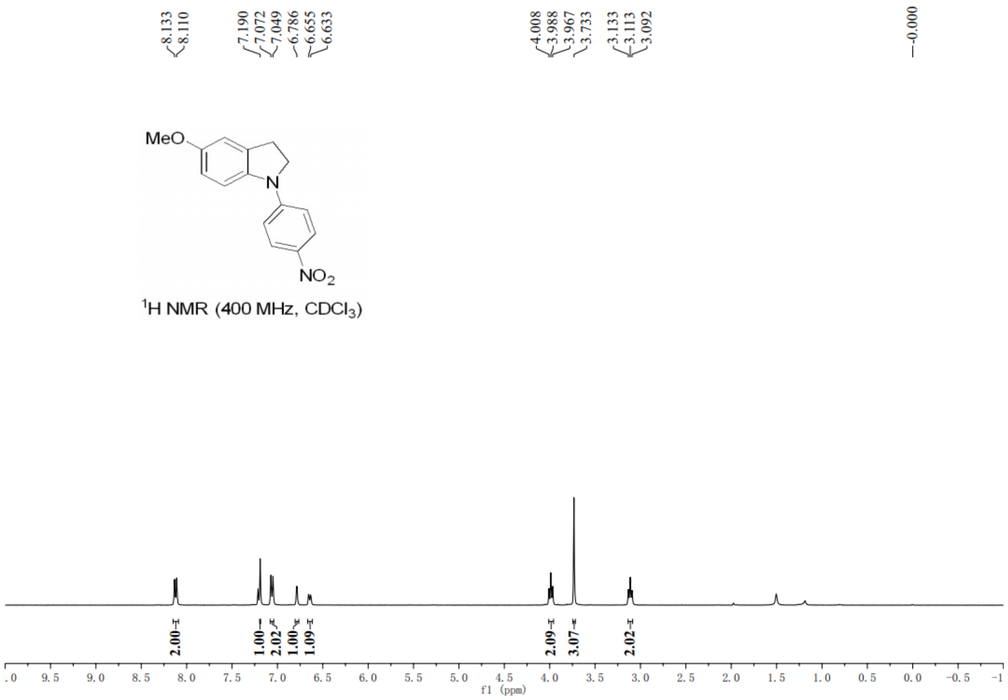

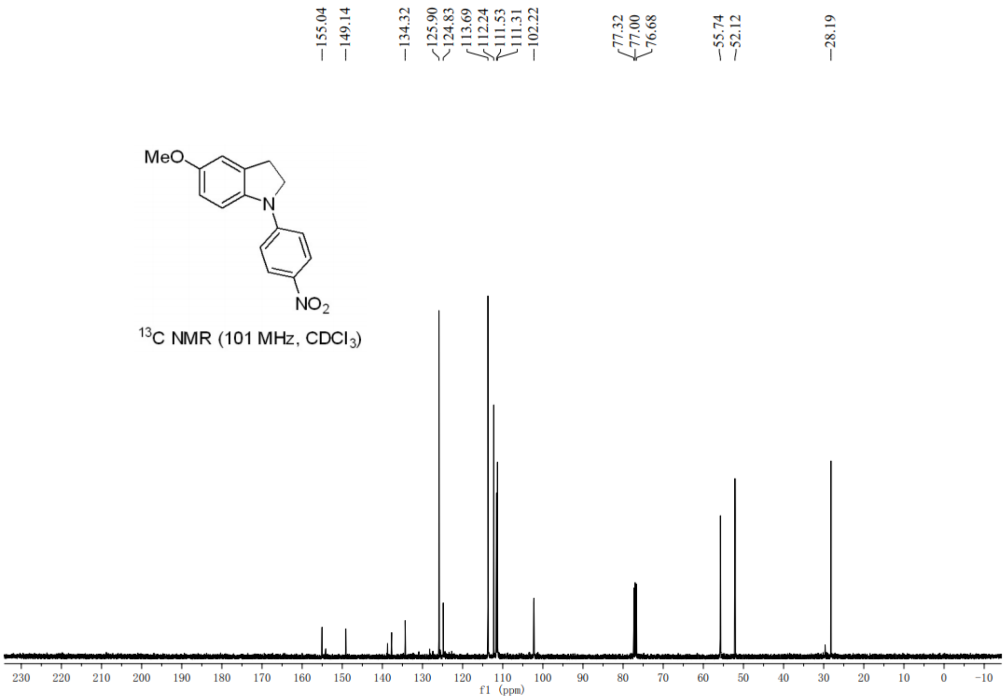

6-methoxy-1-(4-nitrophenyl) indoline (3b-11)

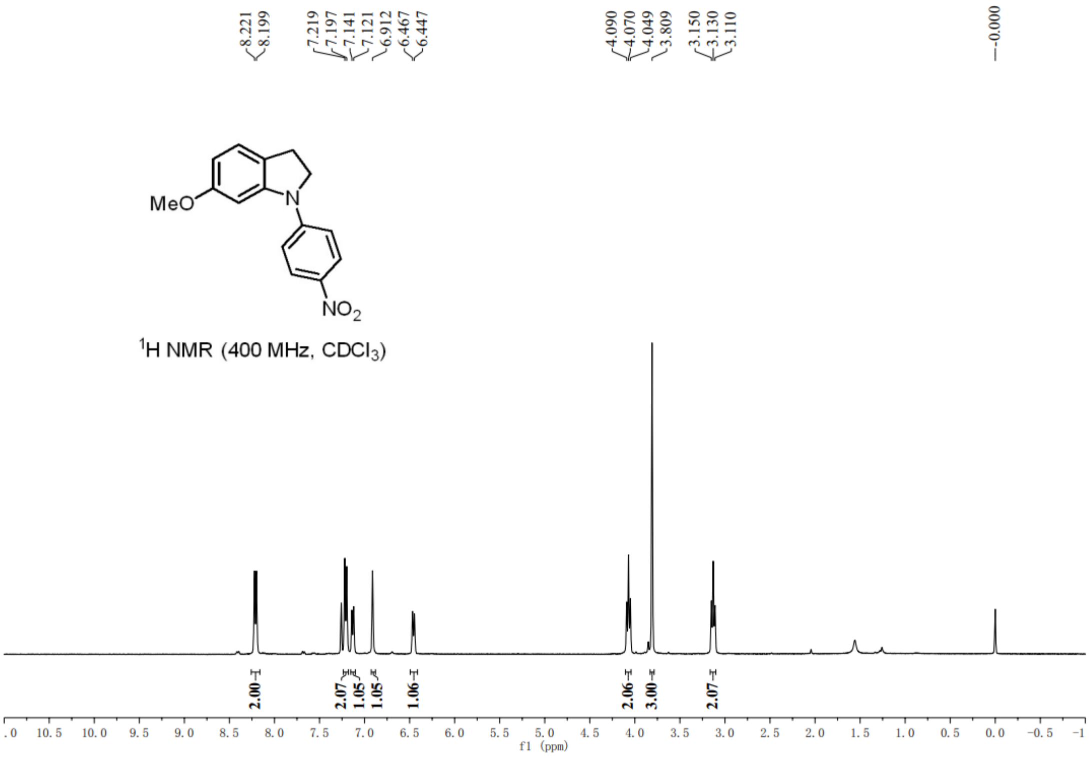

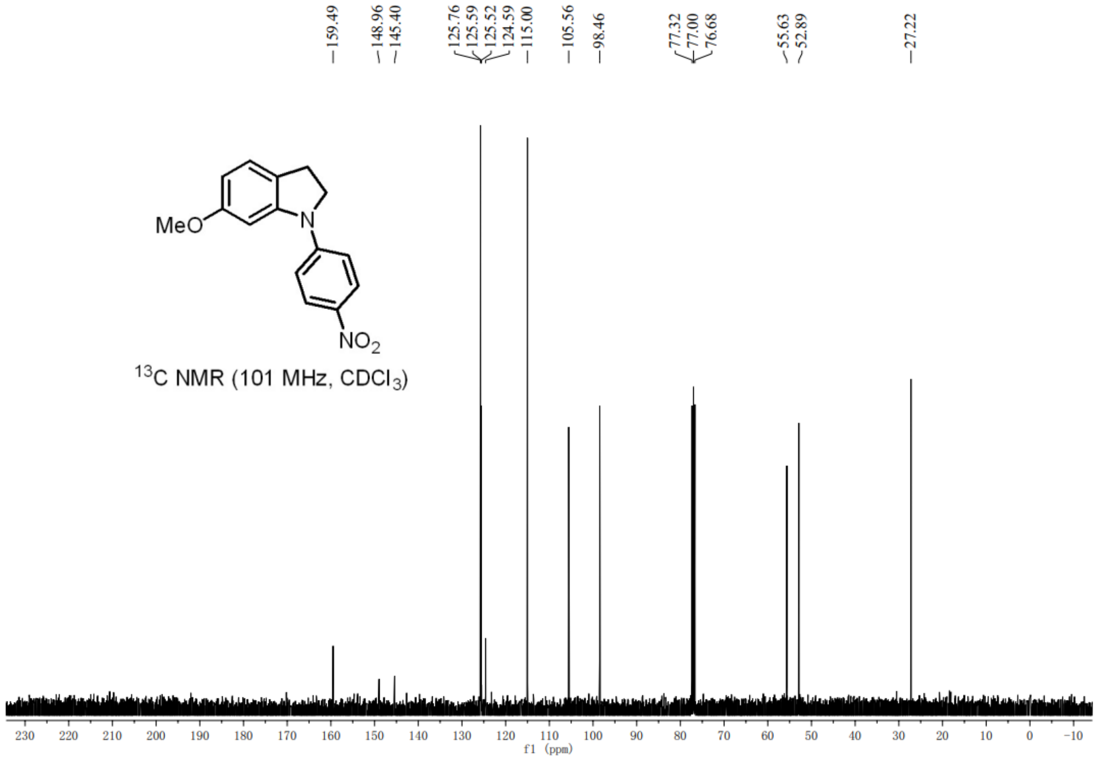

5-ethoxy-1-(4-nitrophenyl)I ndoline (3b-12)

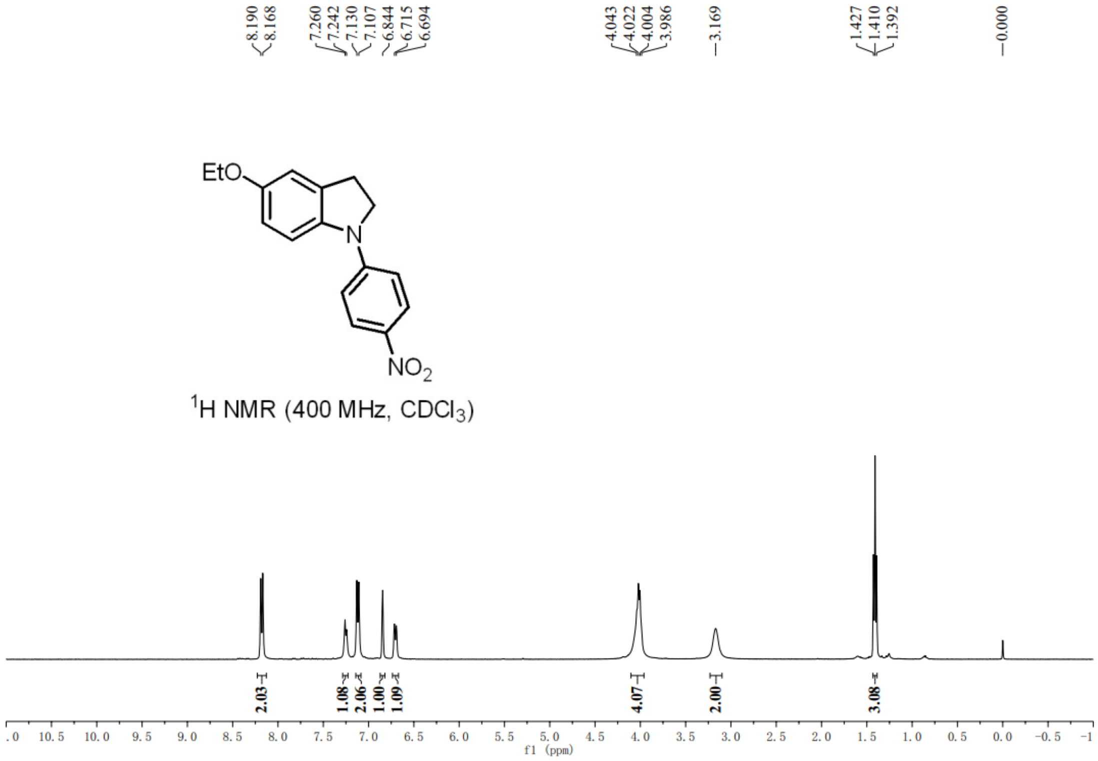

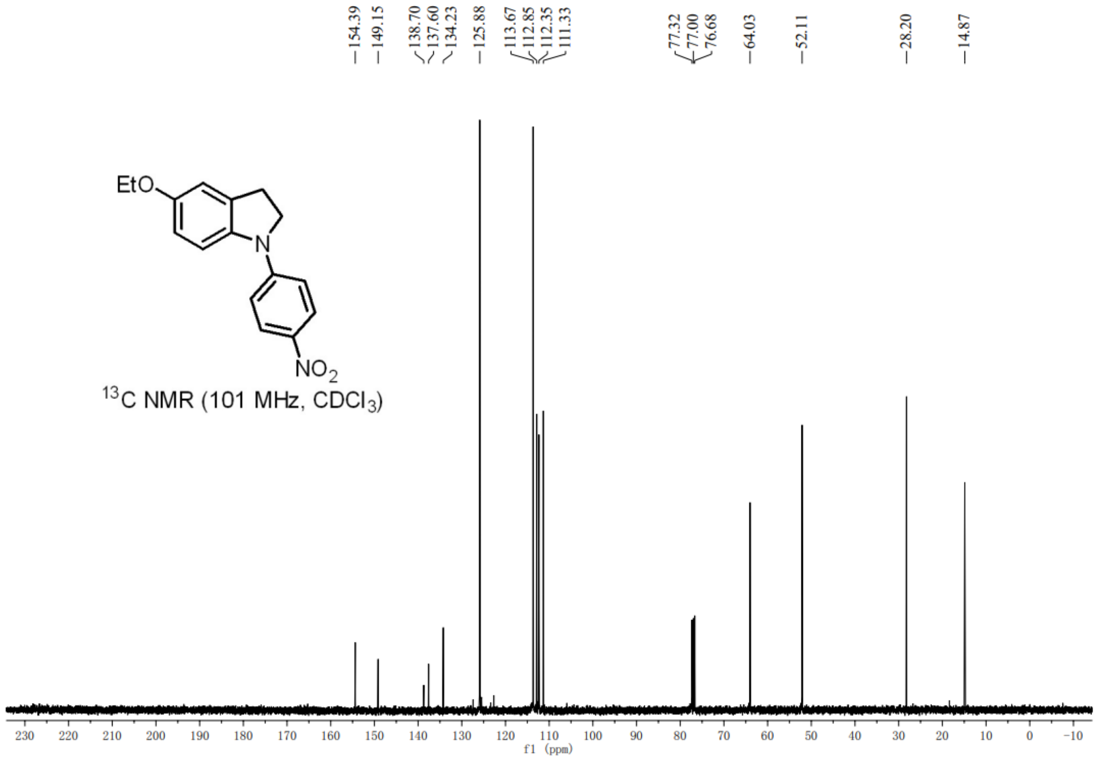

5-isopropoxy-1-(4-nitrophenyl) indoline (3b-13)

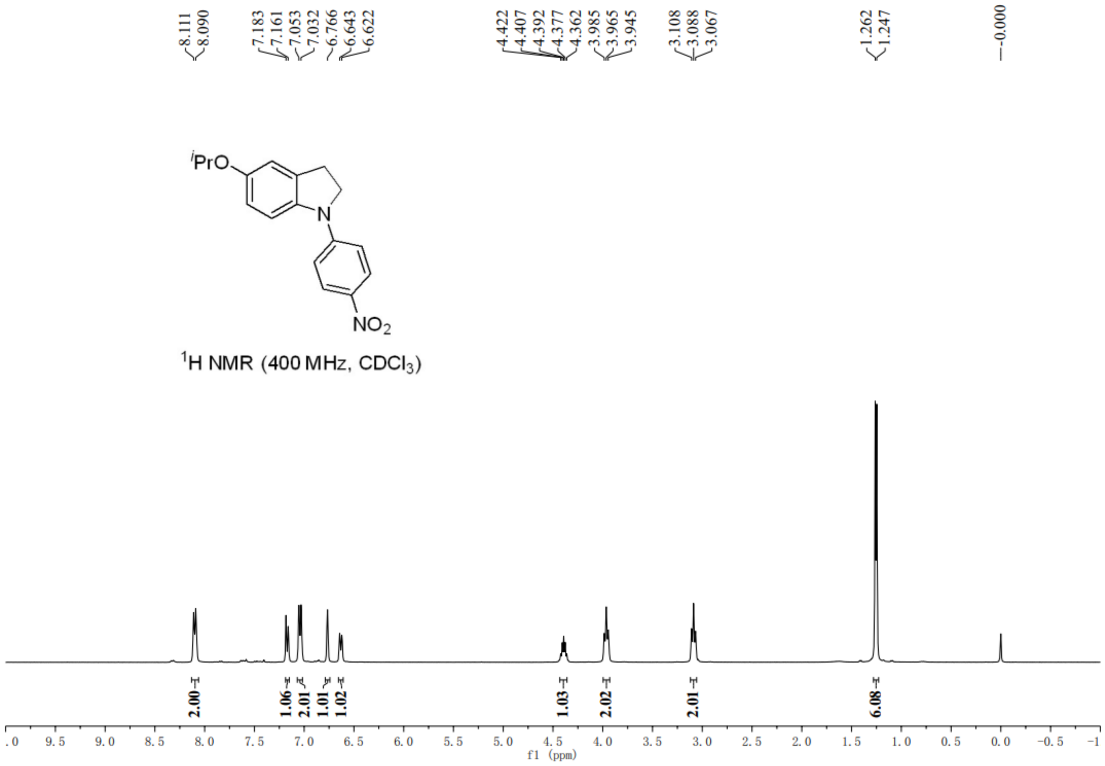

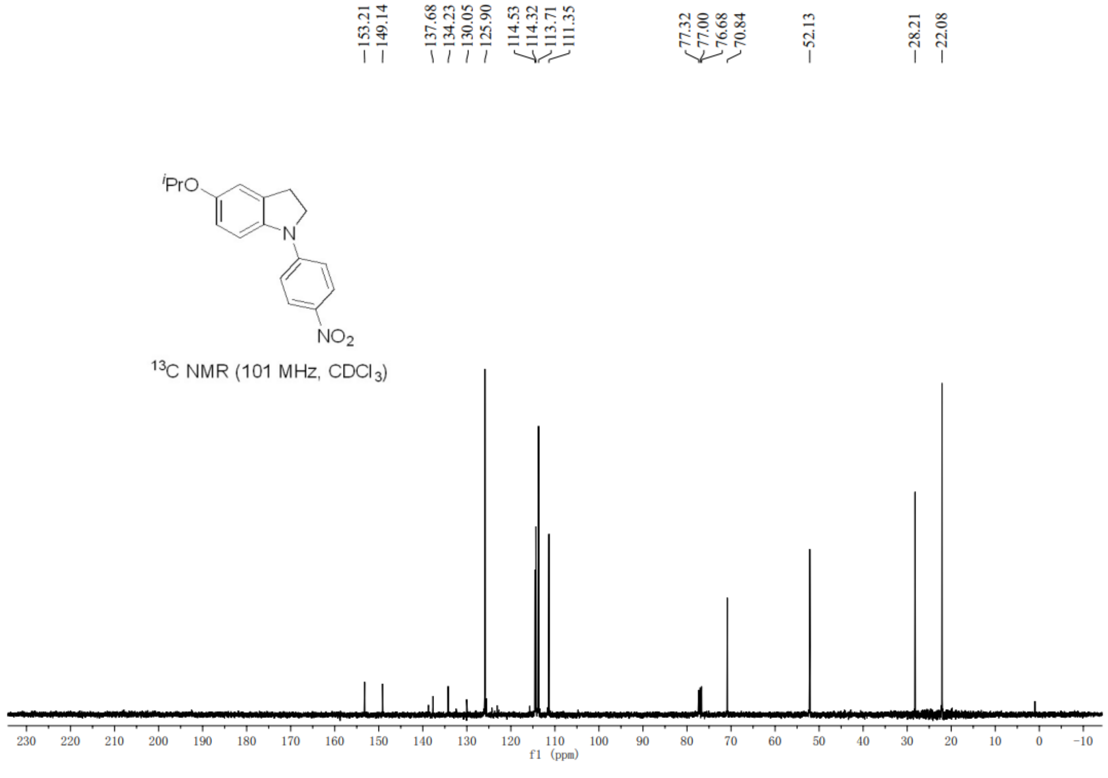

4-fluoro-1-(4-nitrophenyl) indoline (3b-14)

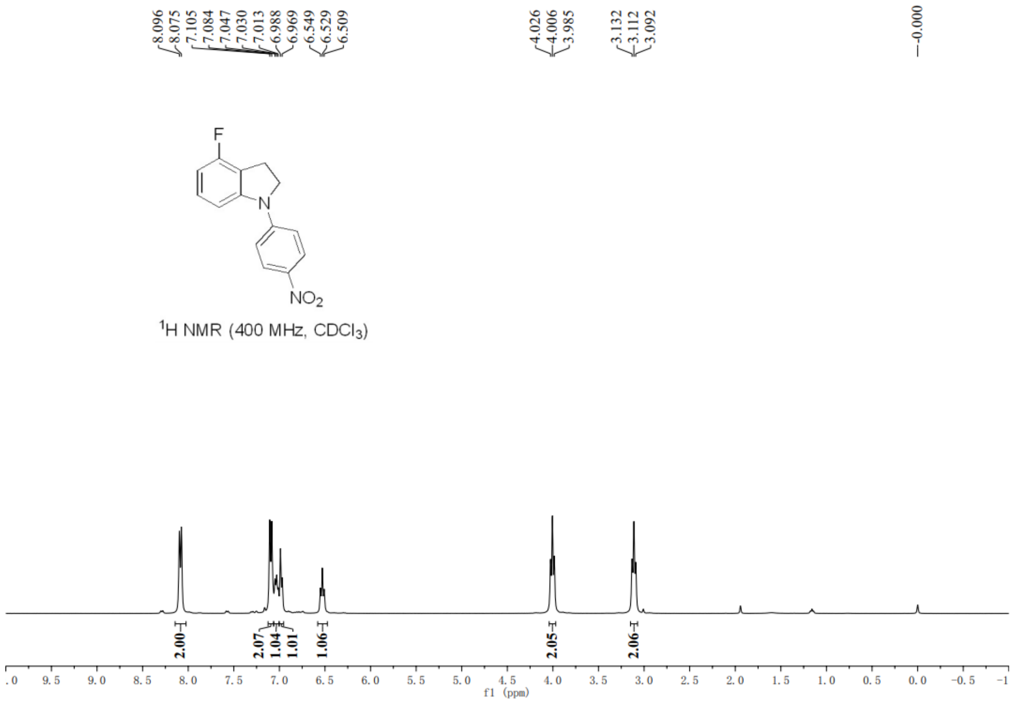

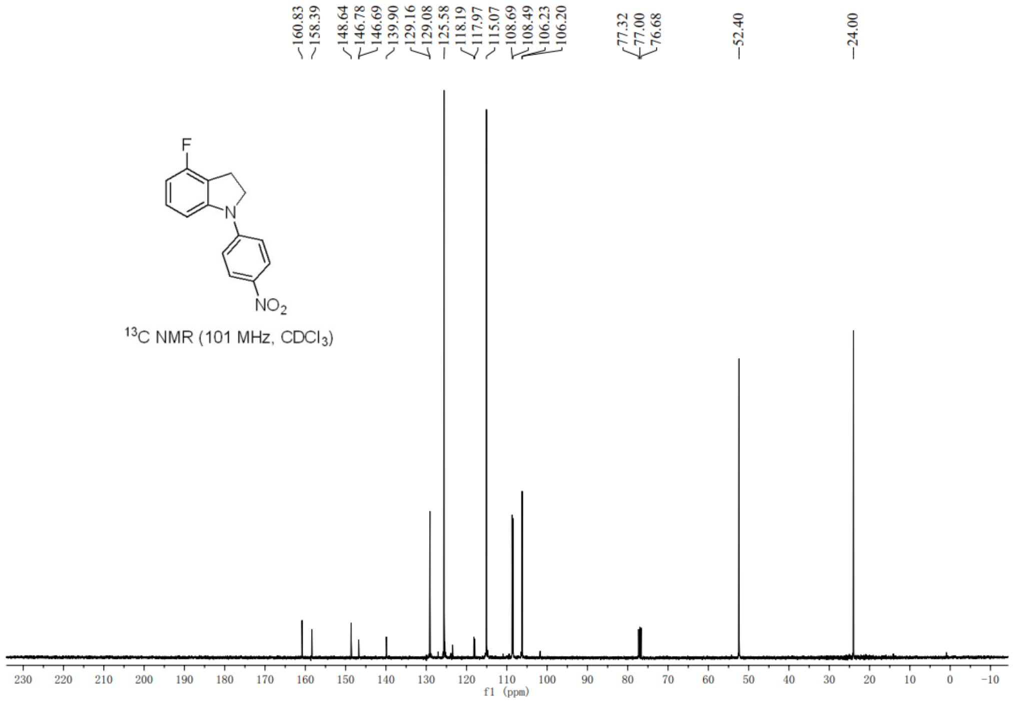

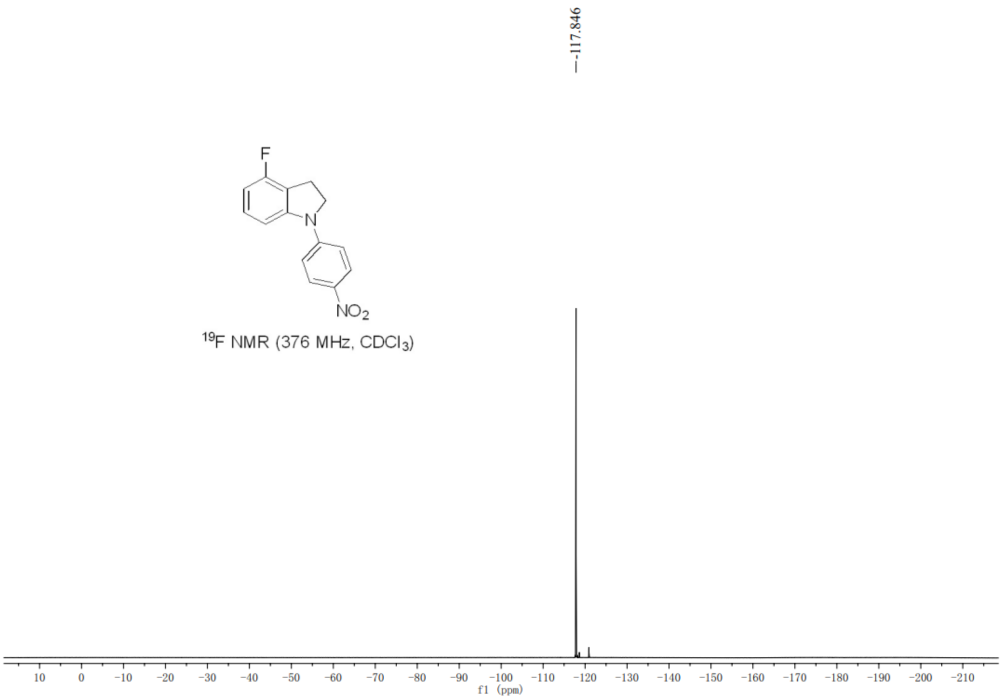

-117.846
F
N
NO2
19F NMR (376 MHz, CDCl3)
10 0 −10 −20 −30 −40 −50 −60 −70 −80 −90 −100 −110 −120 −130 −140 −150 −160 −170 −180 −190 −200 −210
f1 (ppm)

5-fluoro-1-(4-nitrophenyl) indoline (3b-15)

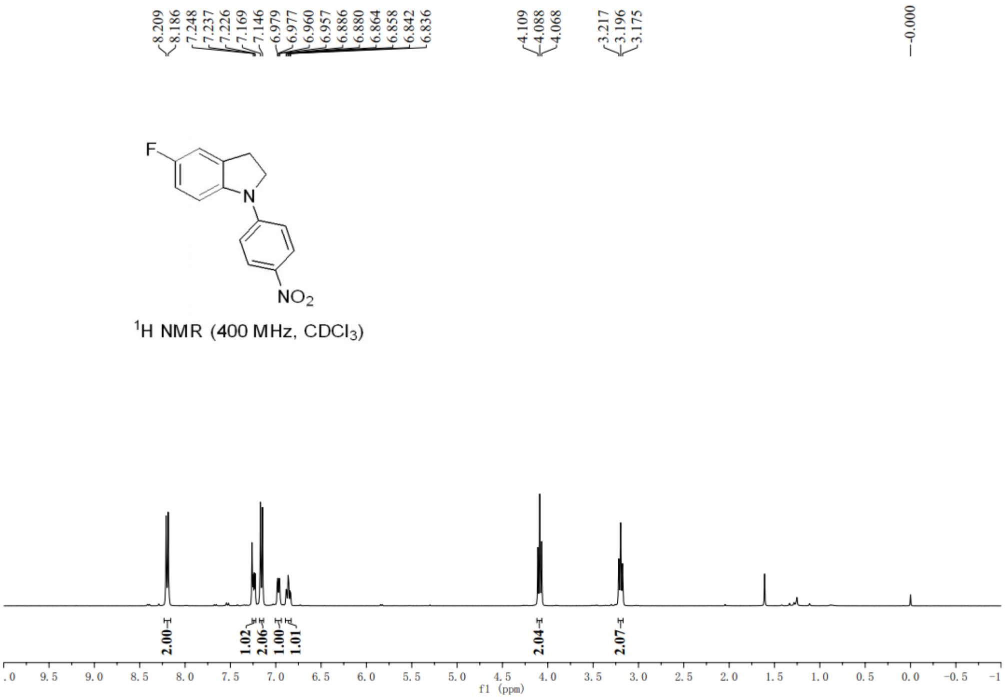

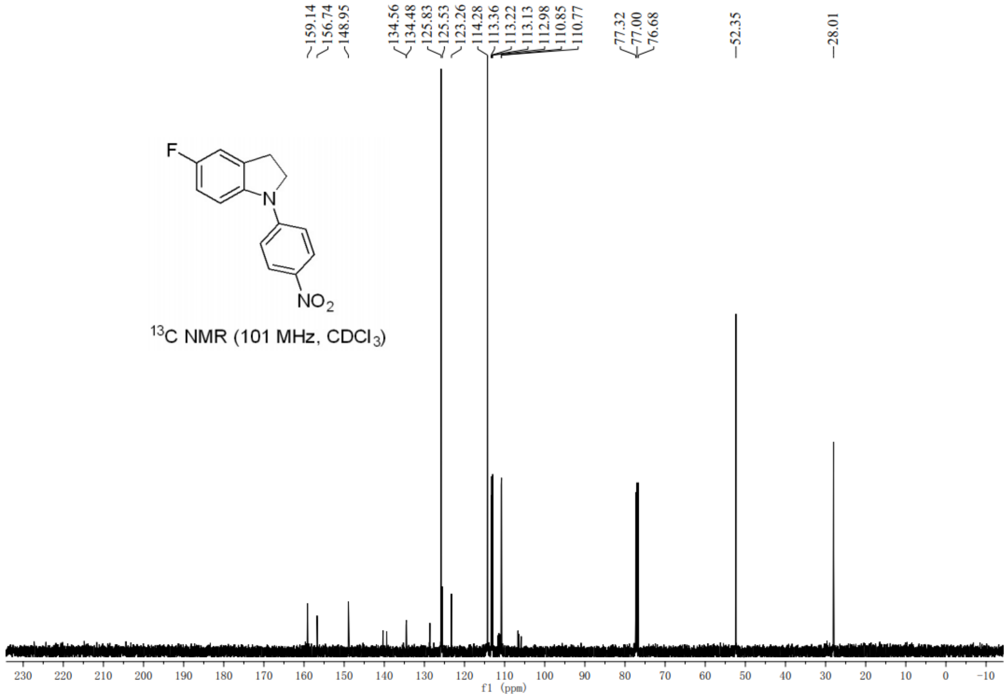

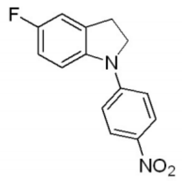

^{19}F NMR (376 MHz, $CDCl_3$)

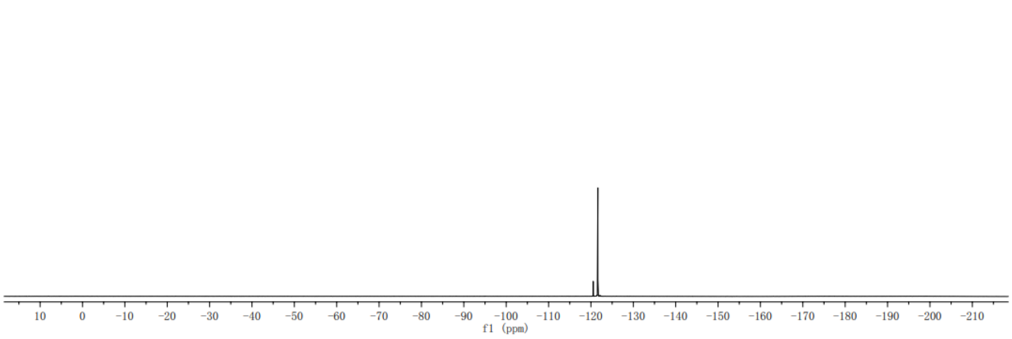

6-fluoro-1-(4-nitrophenyl) indoline (3b-16)

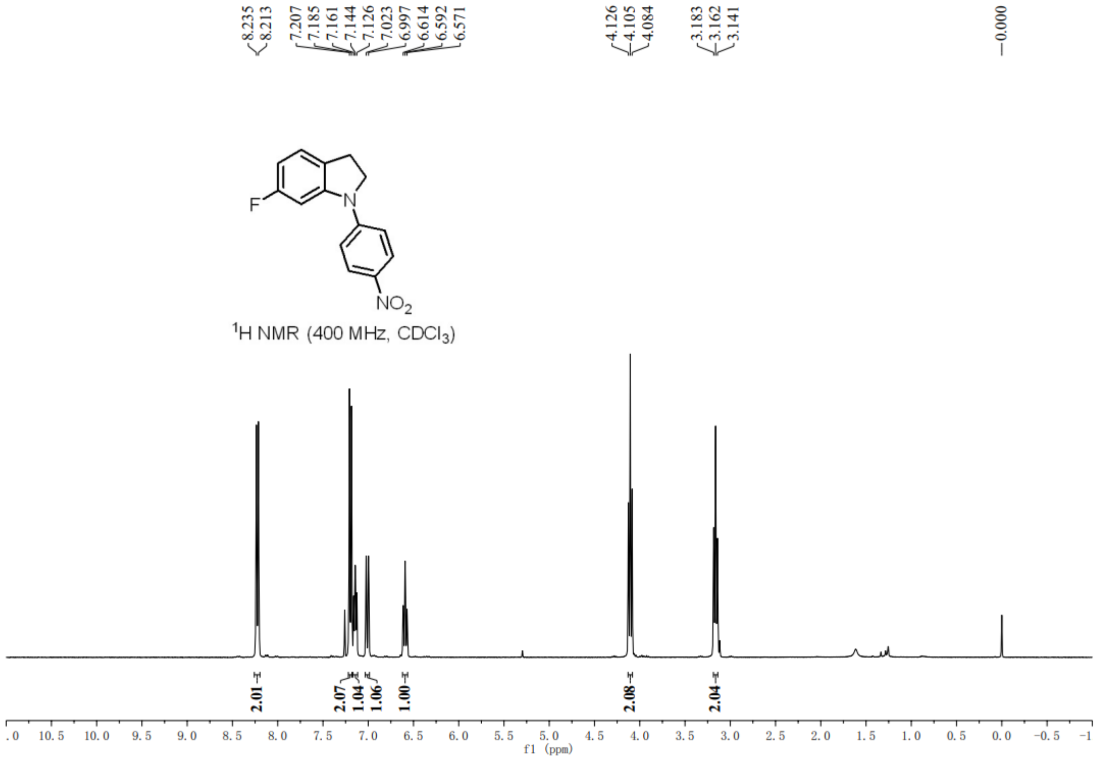

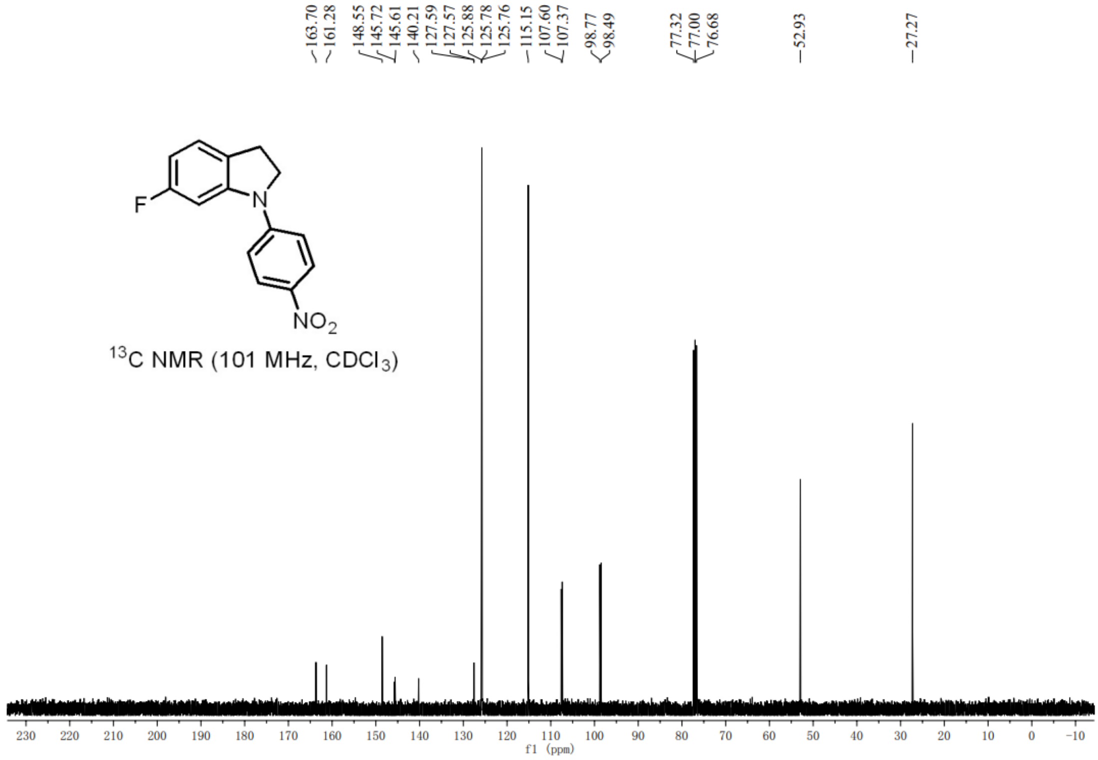

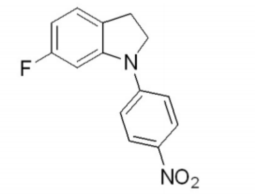

^{19}F NMR (376 MHz, $CDCl_3$)

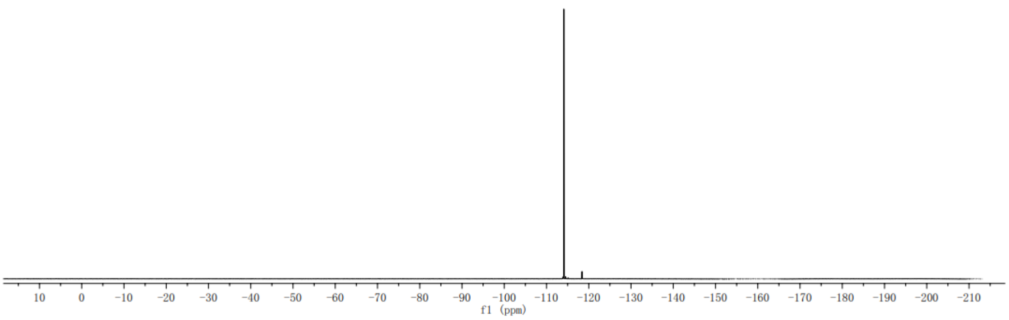

4-chloro-1-(4-nitrophenyl) indoline (3b-17)

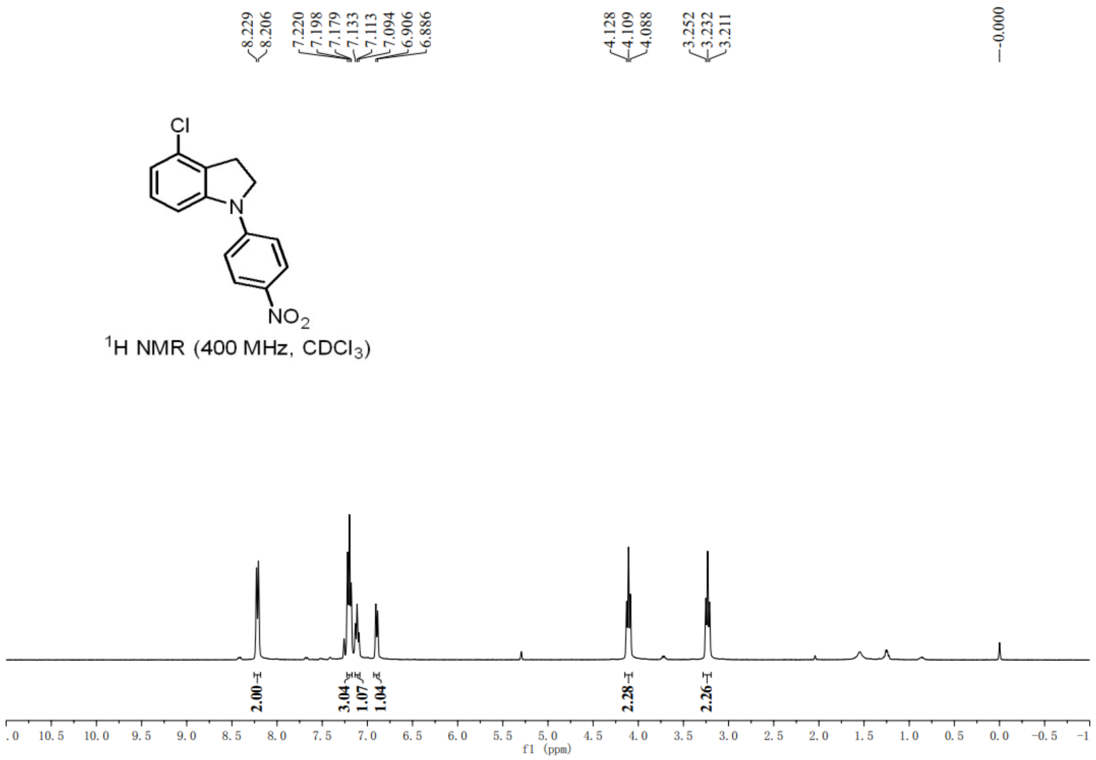

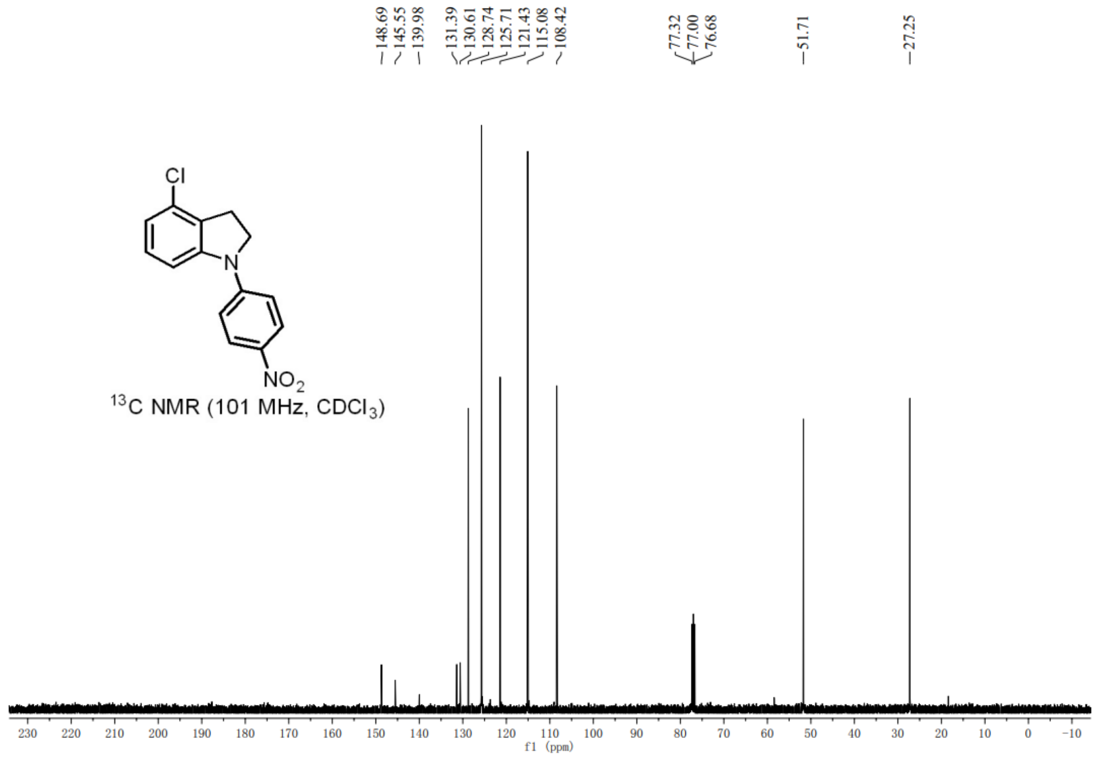

5-chloro-1-(4-nitrophenyl) indoline (3b-18)

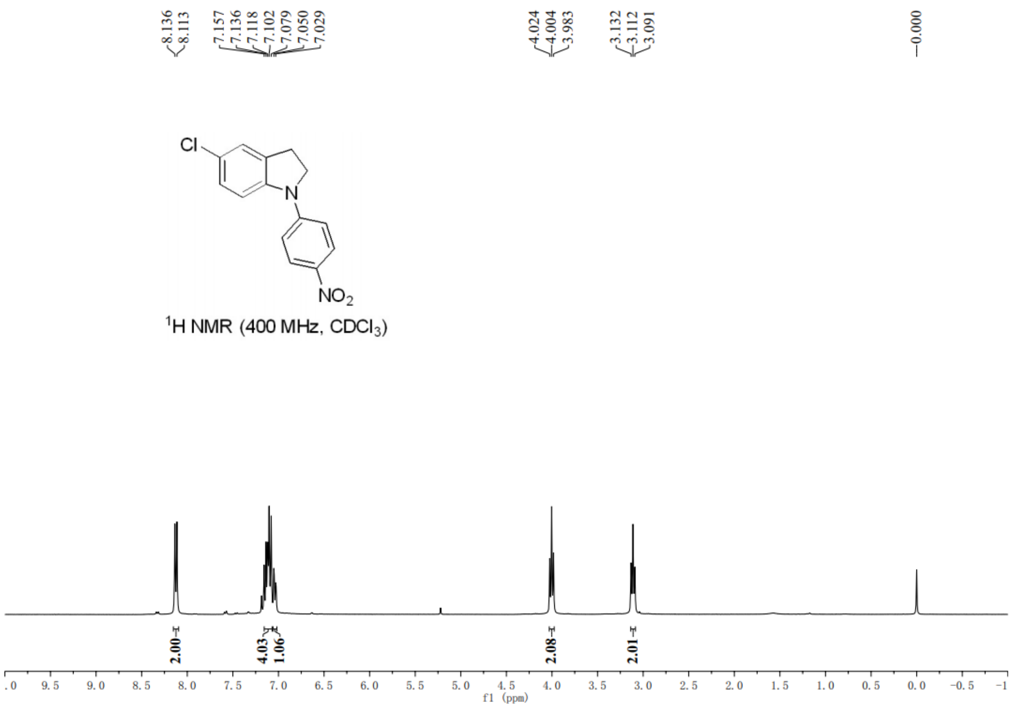

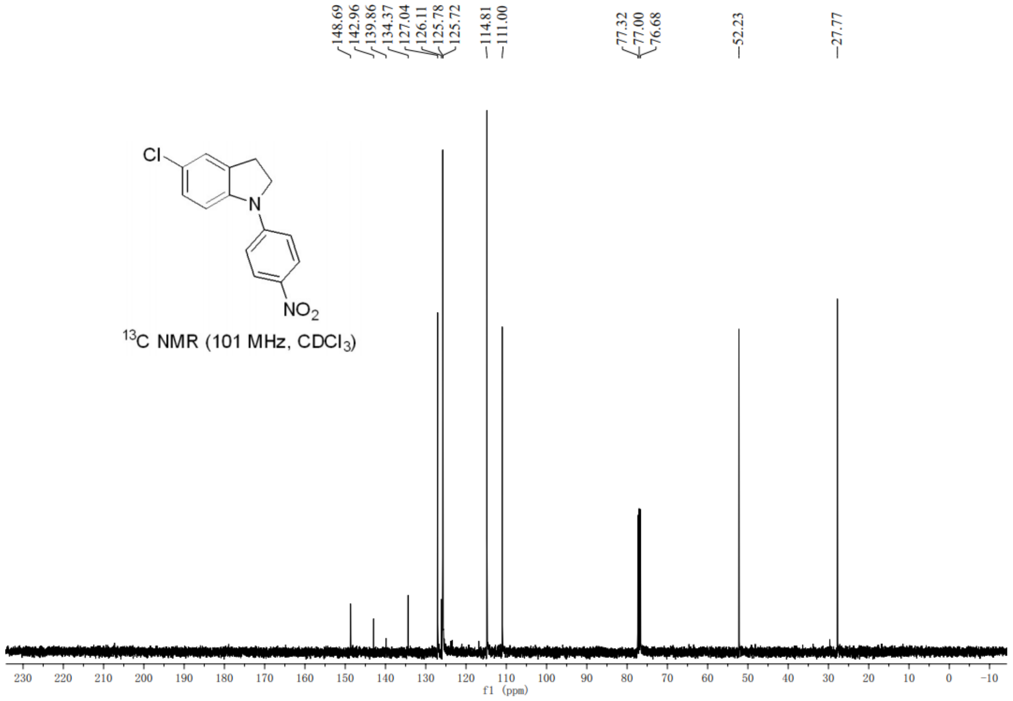

6-chloro-1-(4-nitrophenyl) indoline (3b-19)

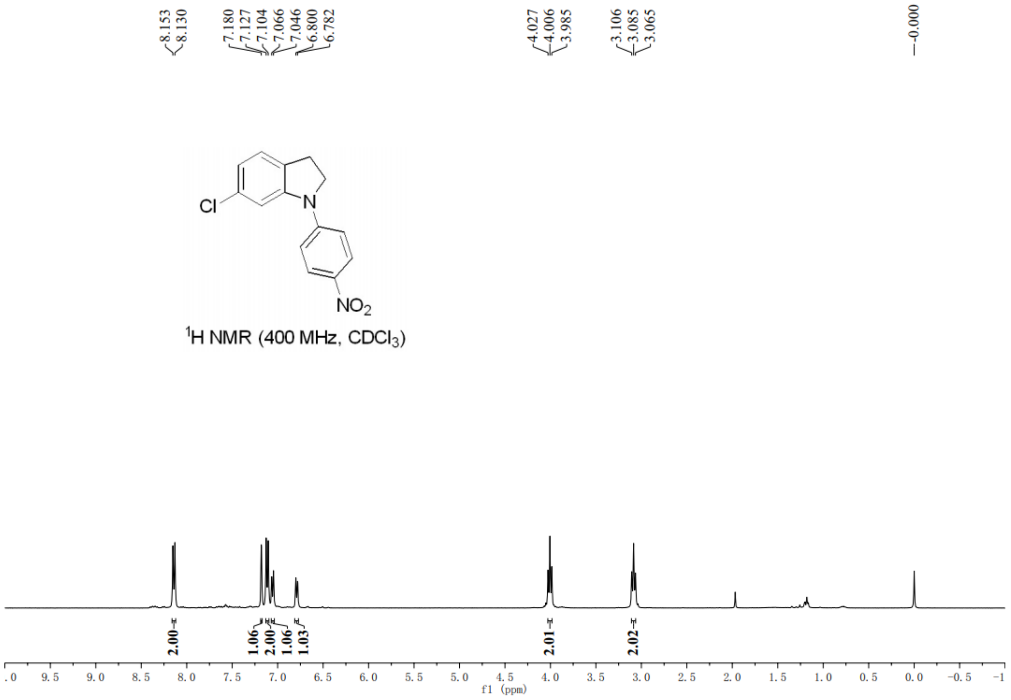

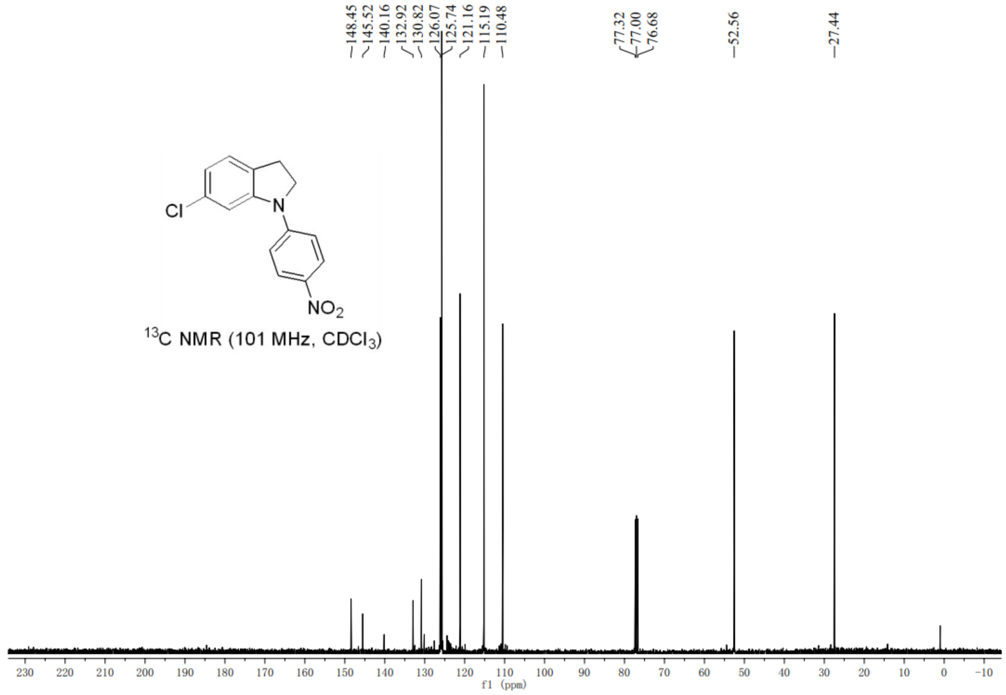

4-bromo-1-(4-nitrophenyl) indoline (3b-20)

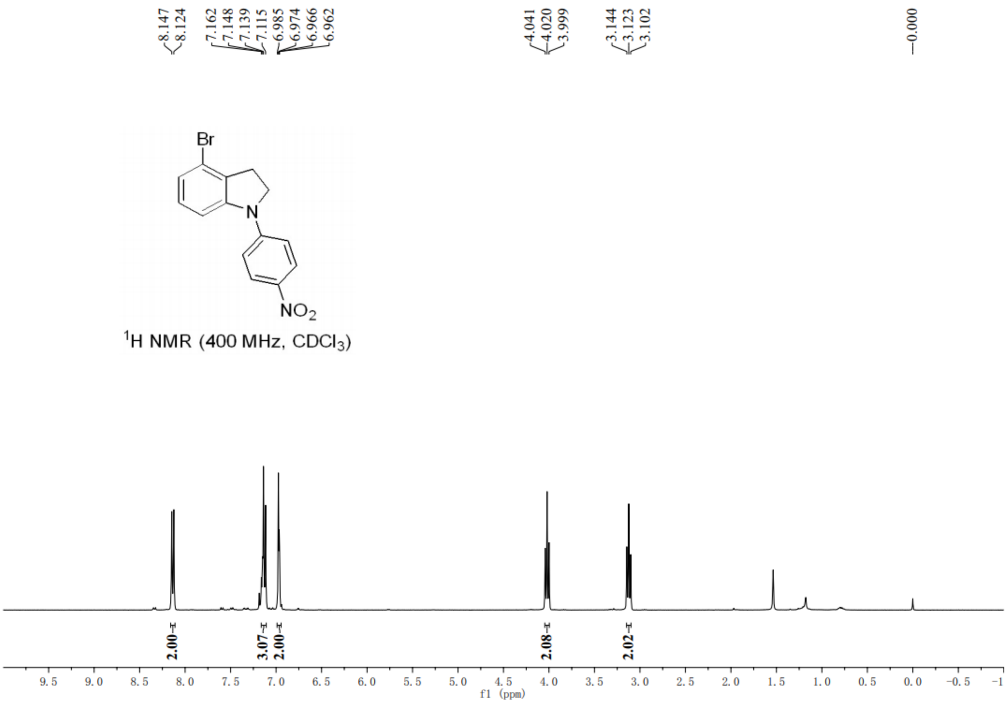

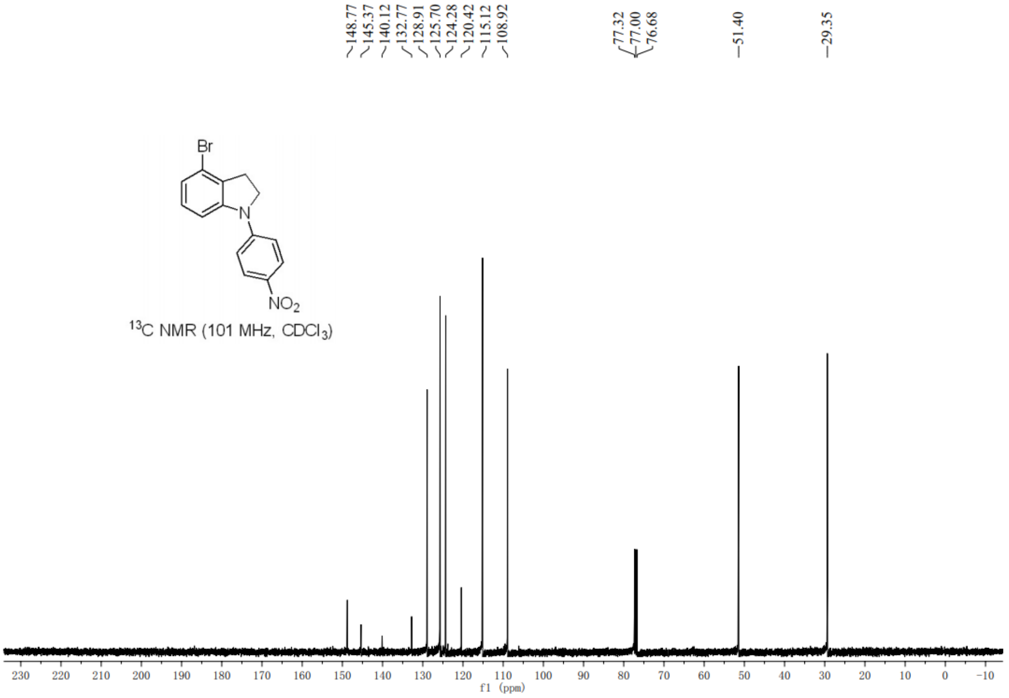

5-bromo-1-(4-nitrophenyl) indoline (3b-21)

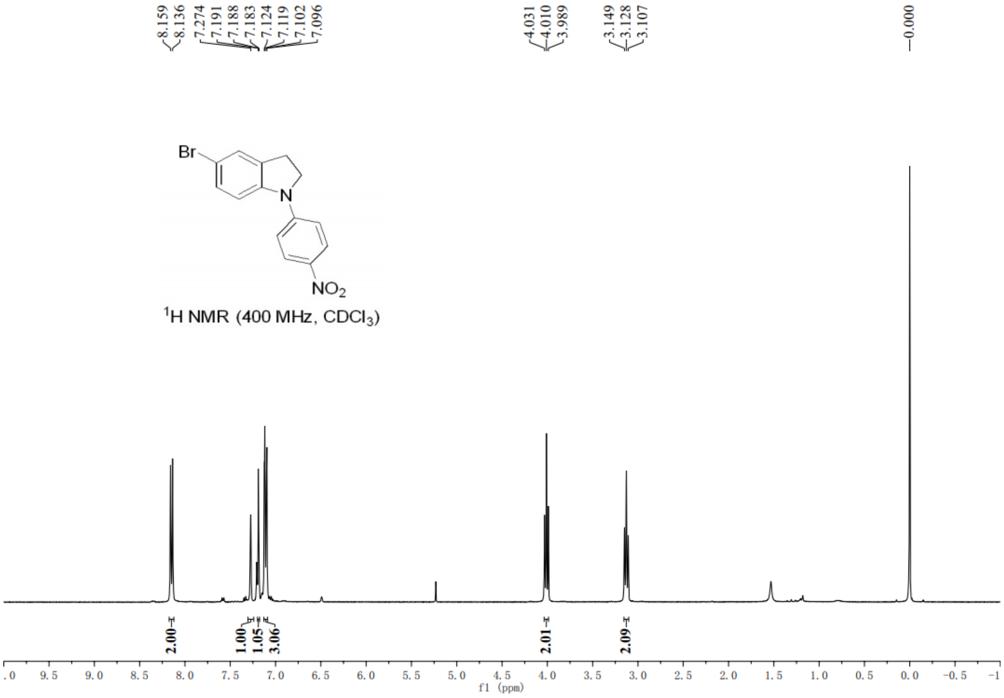

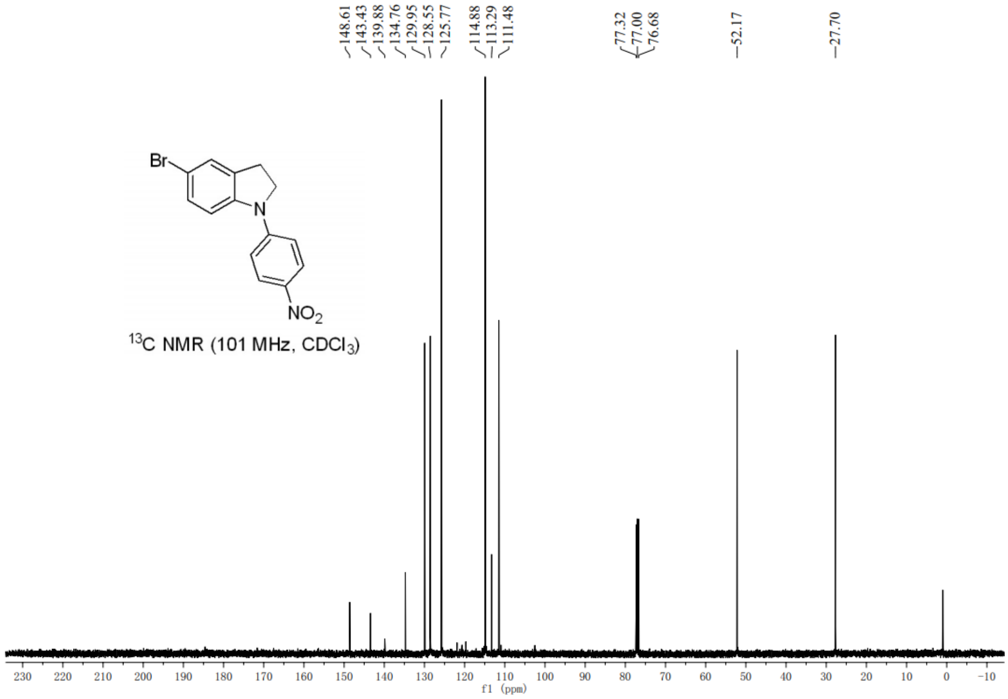

6-bromo-1-(4-nitrophenyl) indoline (3b-22)

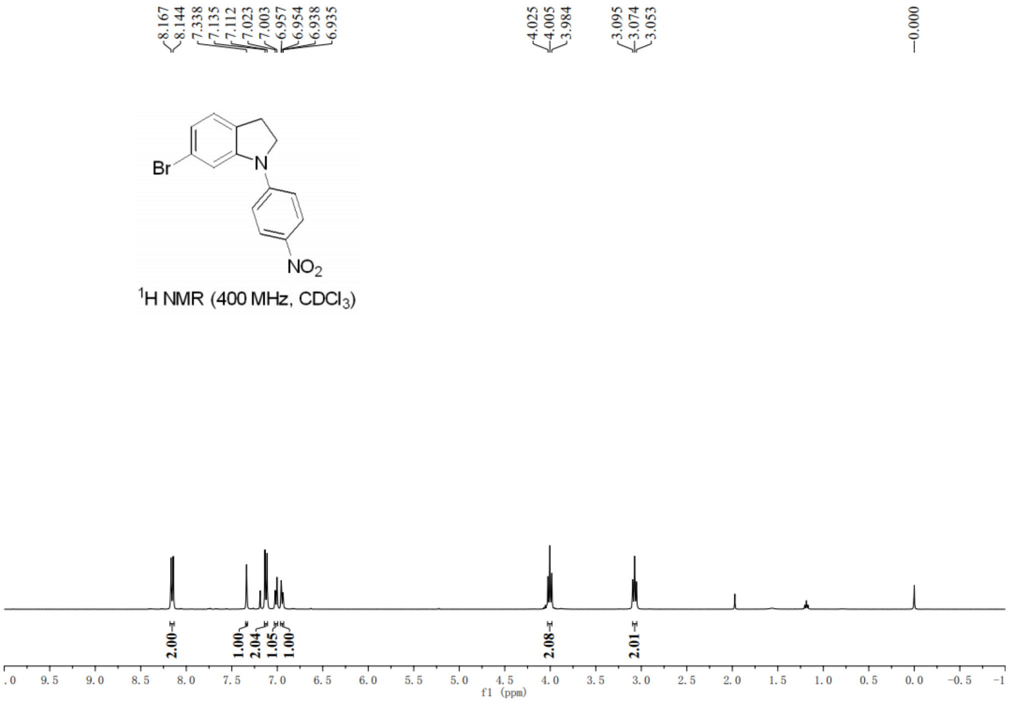

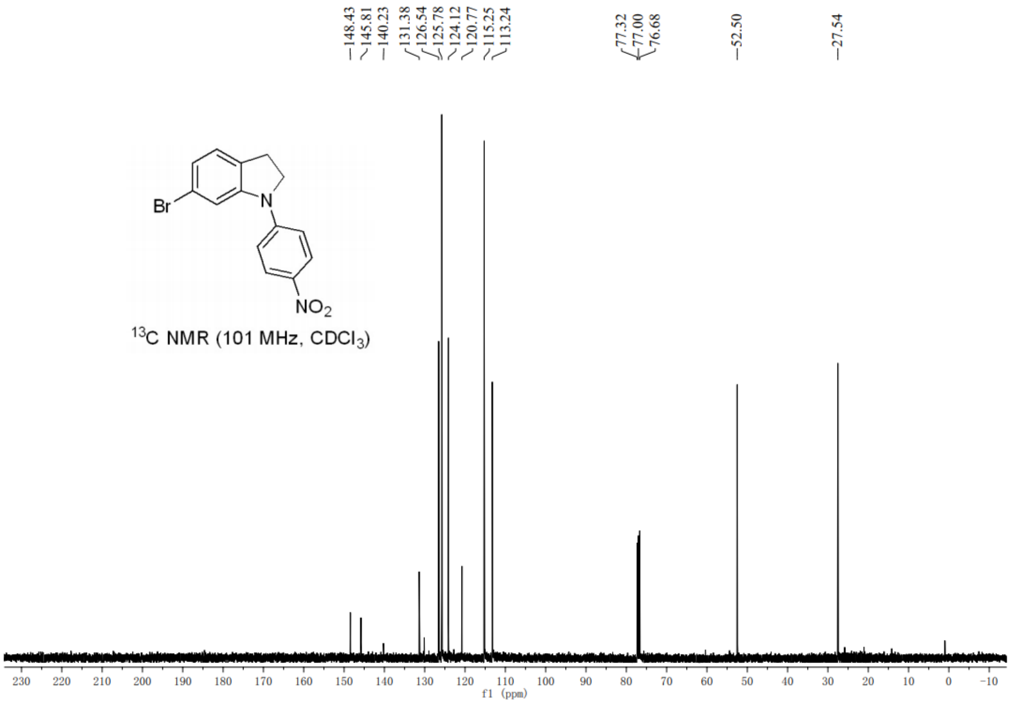

1-(4-nitrophenyl)-1*H*-indole (3b-23)

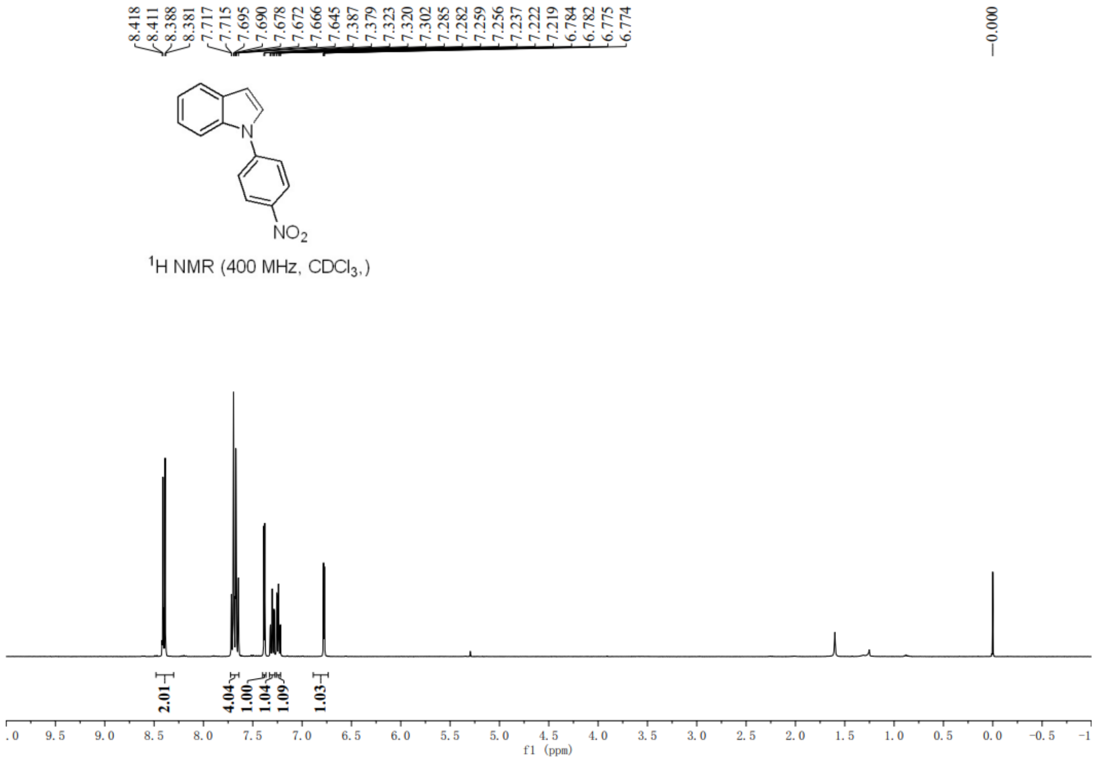

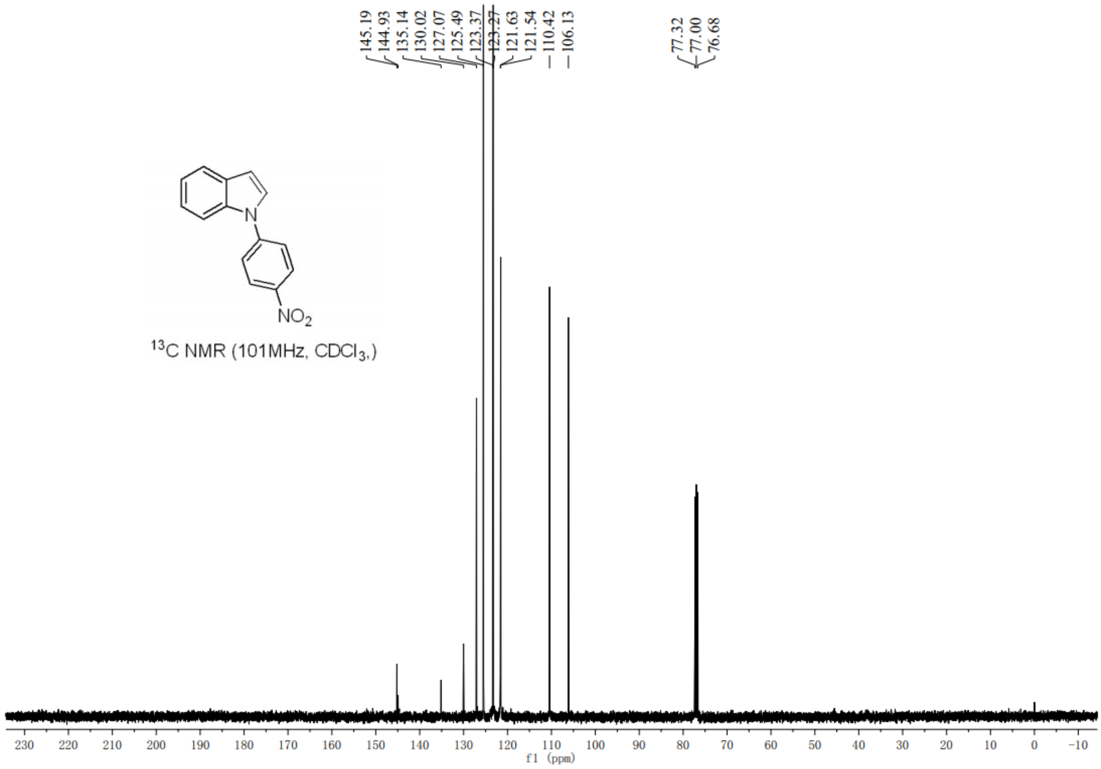

7-methyl-1-(4-nitrophenyl)indoline (3b-24)

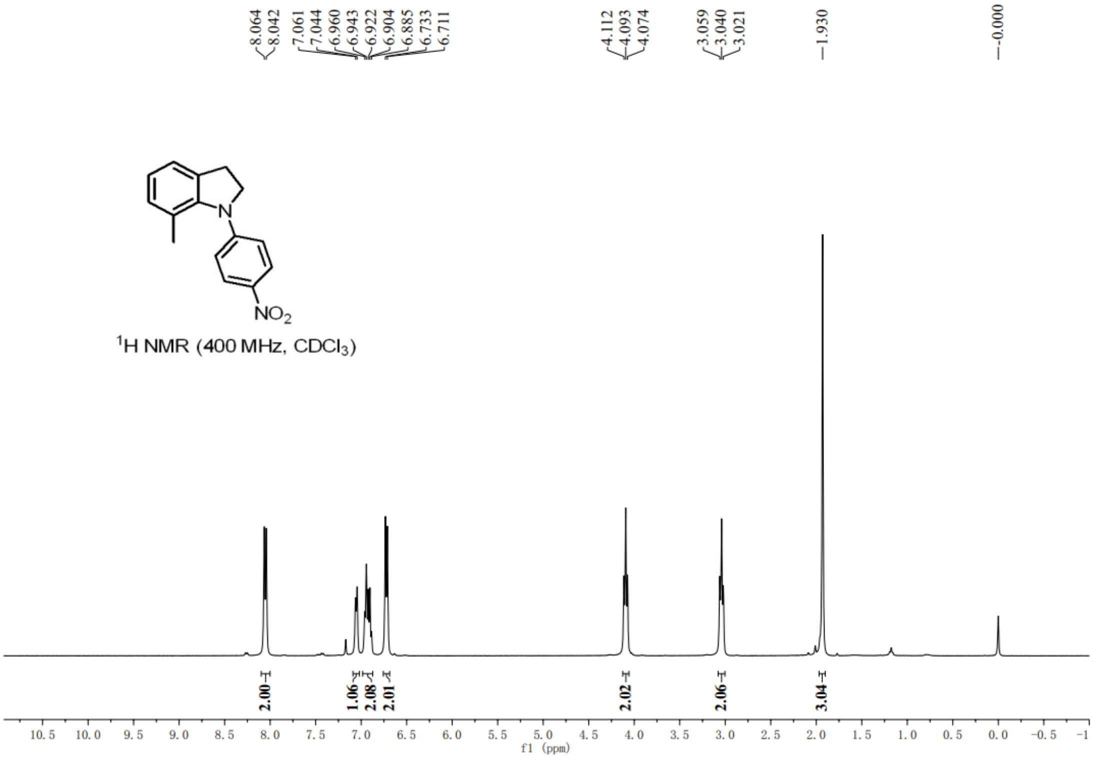

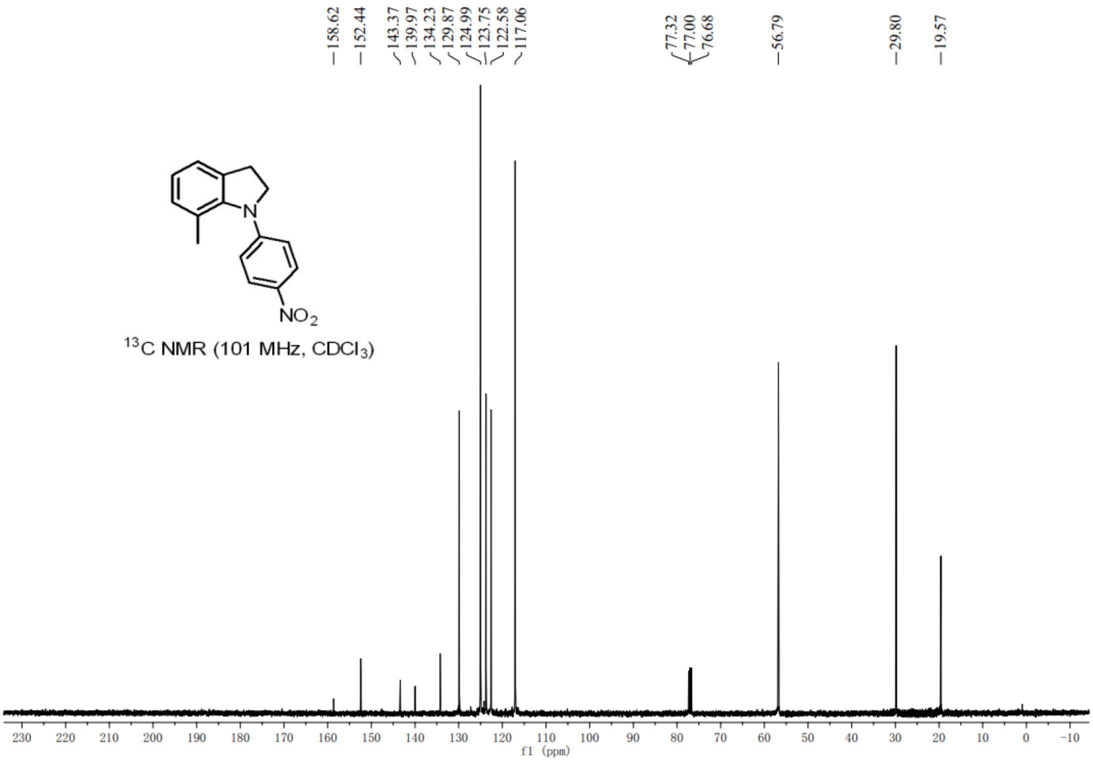

N-(4-nitrophenyl)naphthalen-1-amine (3c-1)

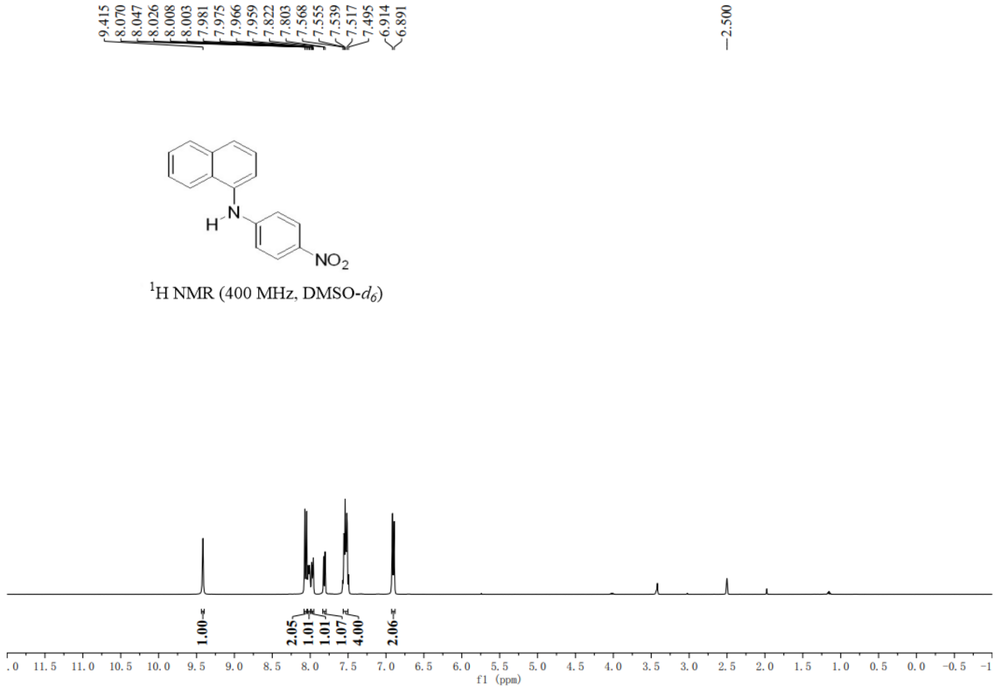

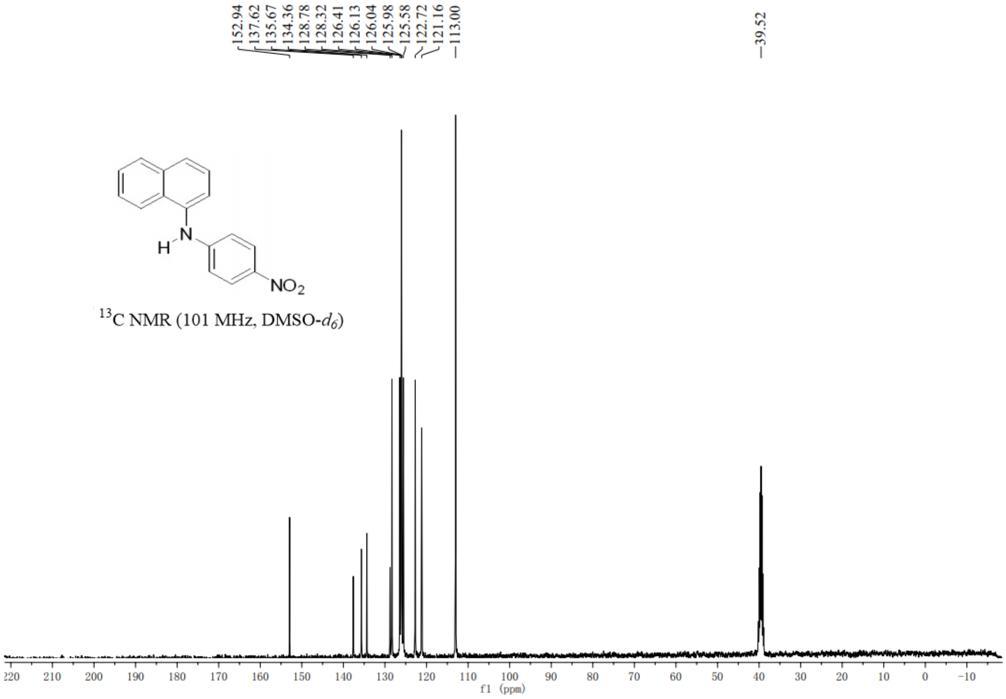

N-(4-nitrophenyl)naphthalen-2-amine (3c-2)

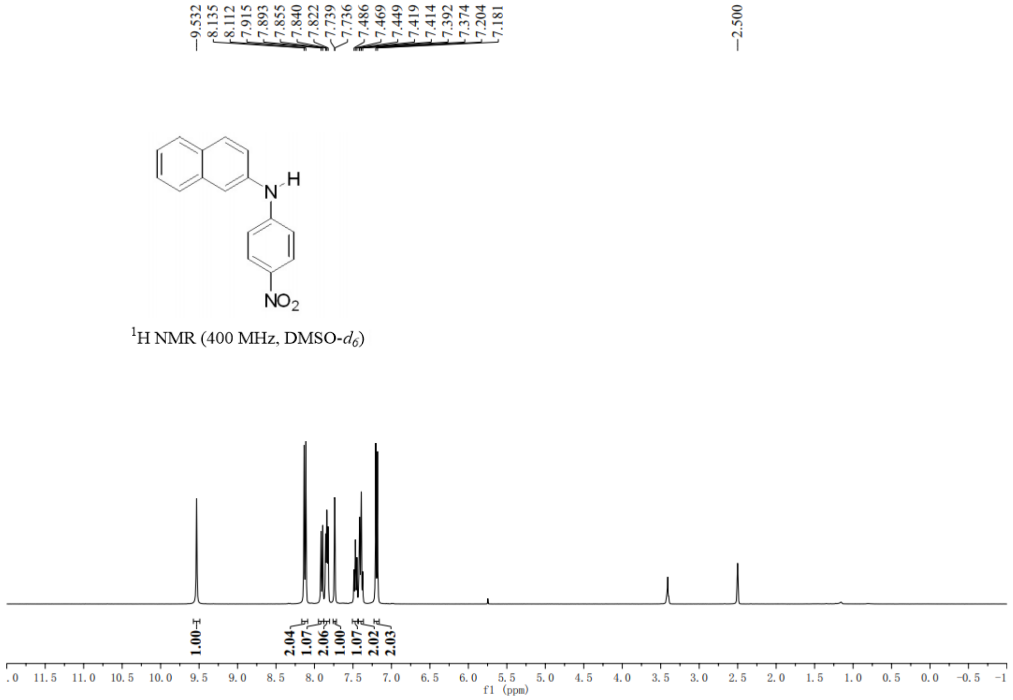

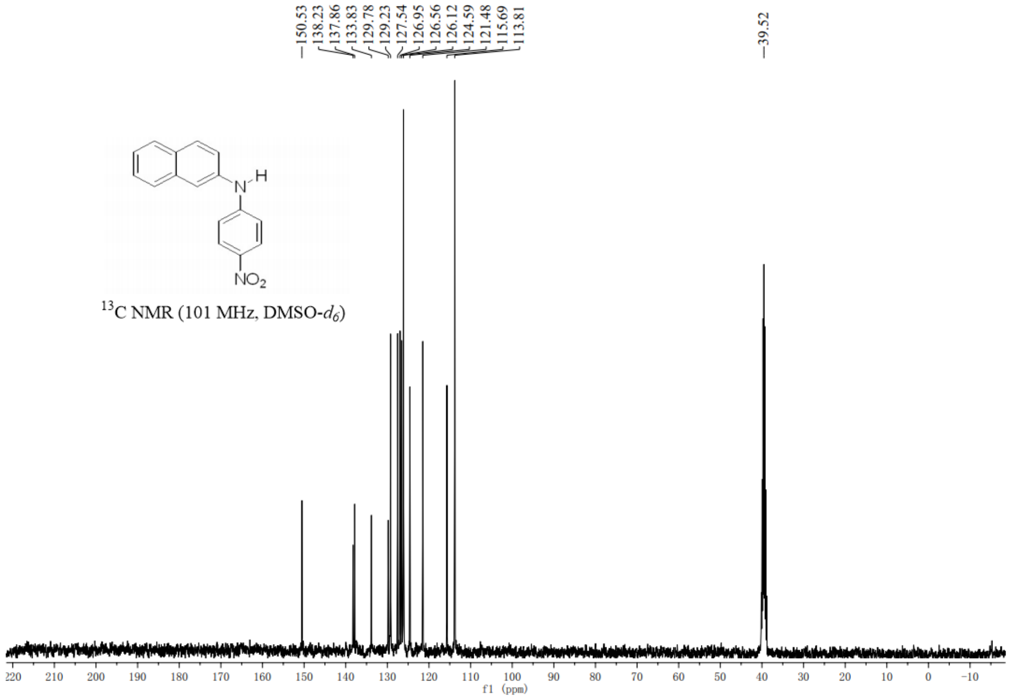

N-methyl-4-nitro-N-phenylaniline (3c-3)

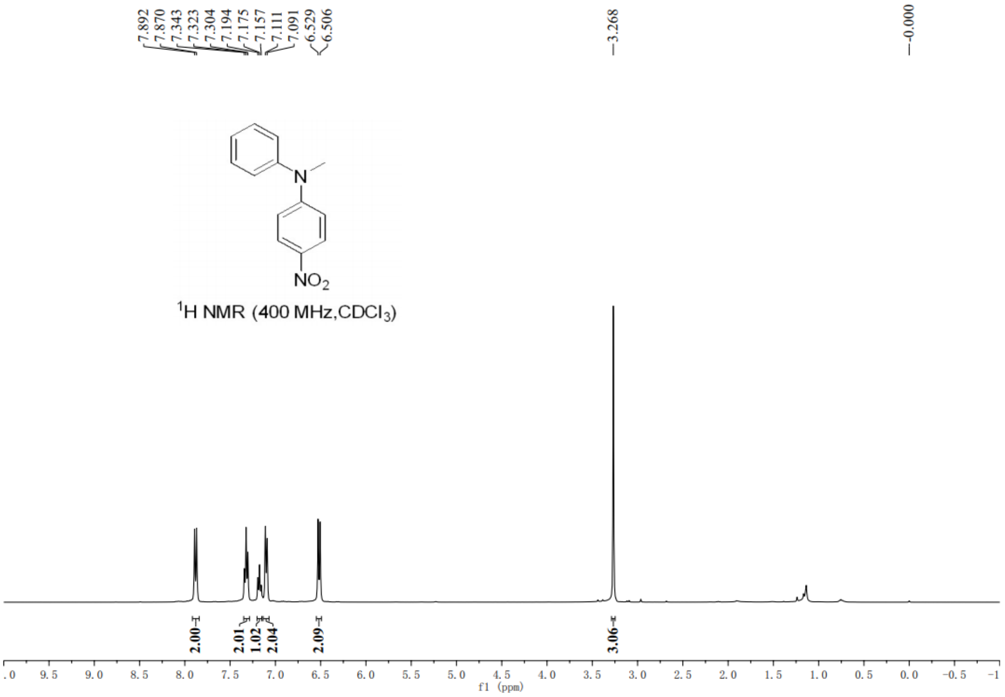

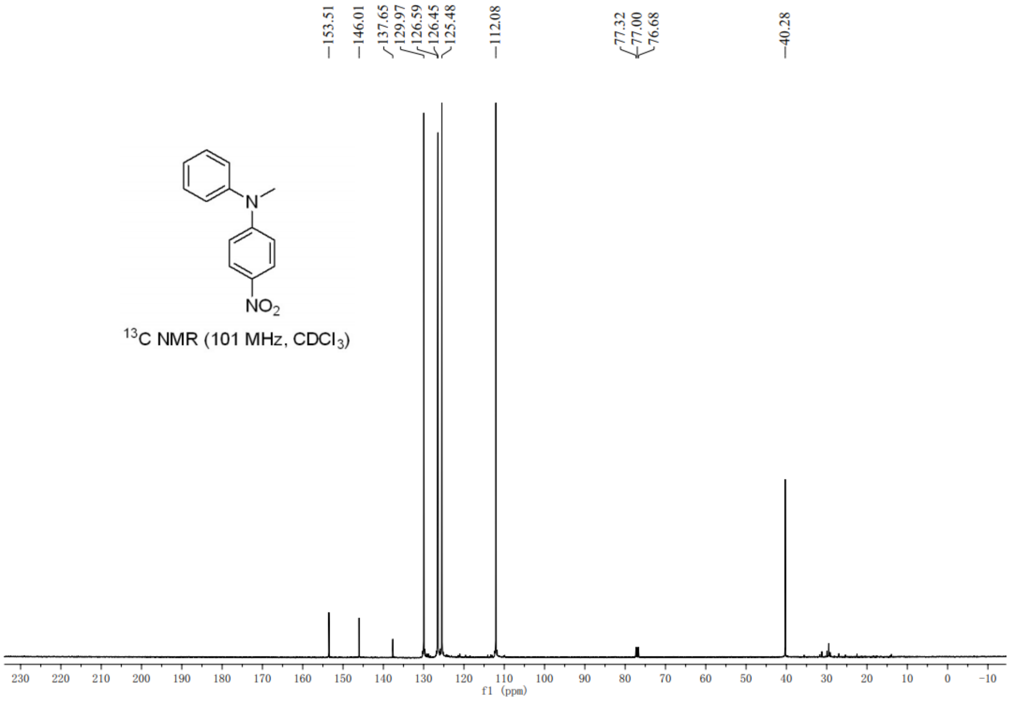

N,4-dimethyl-*N*-(4-nitrophenyl) aniline (3c-4)

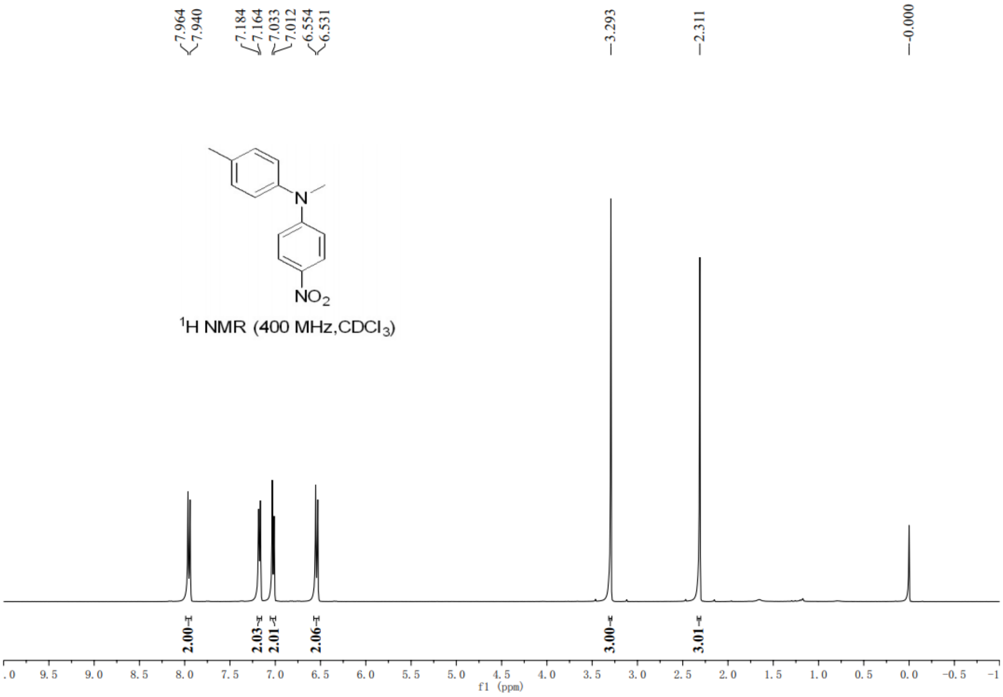

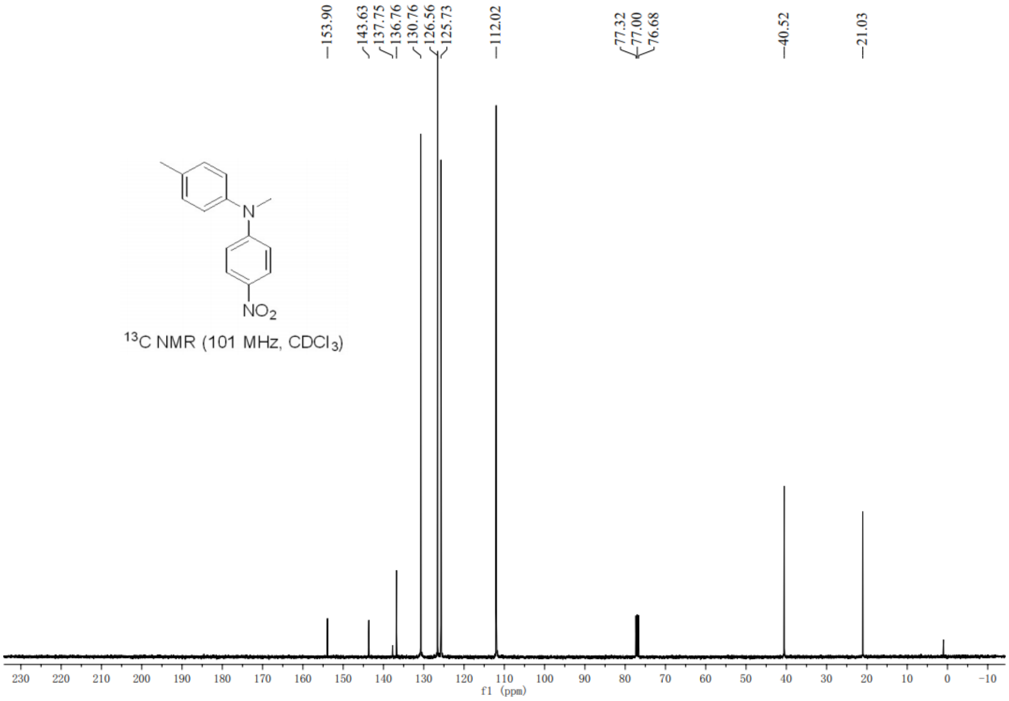

4-methoxy-*N*-methyl-*N*-(4-nitrophenyl) aniline (3c-5)

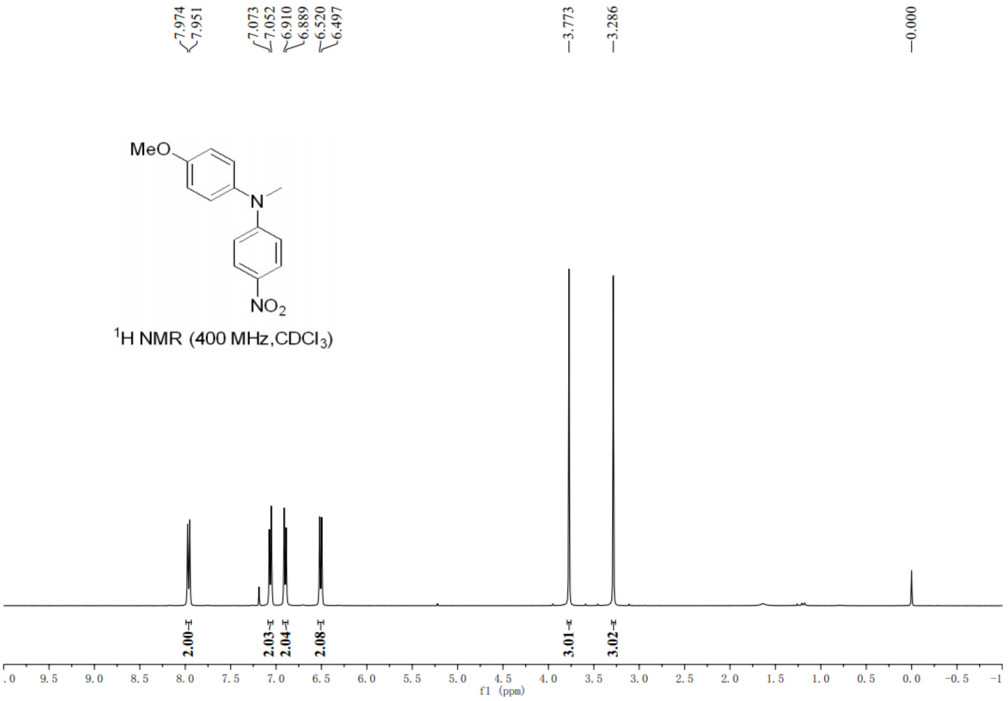

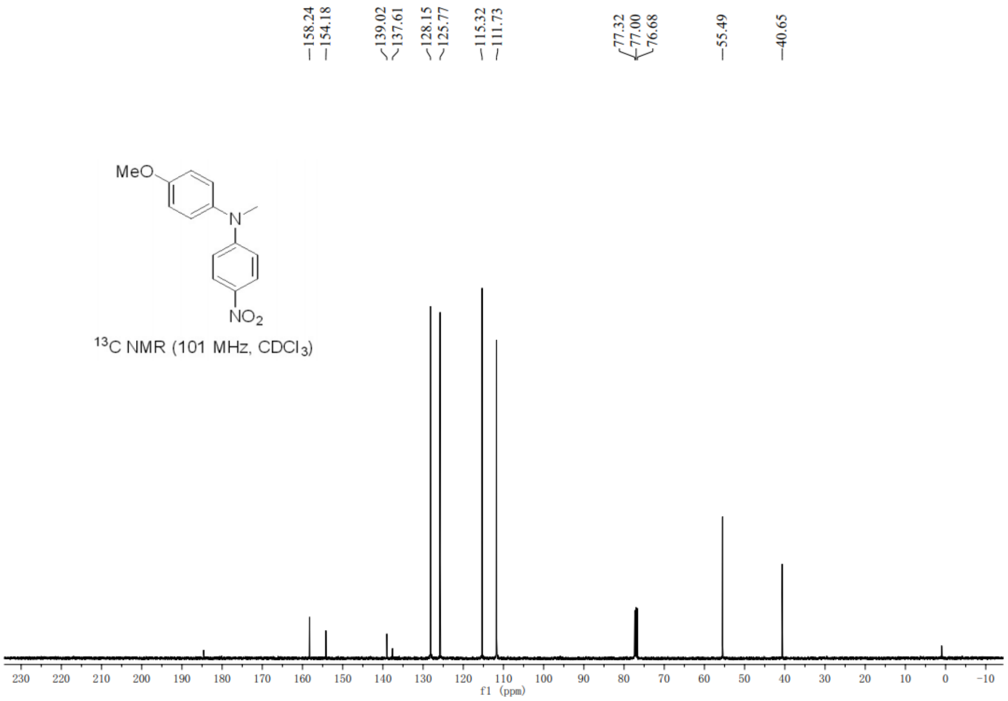

4-fluoro-*N*-methyl-*N*-(4-nitrophenyl) aniline (3c-6)

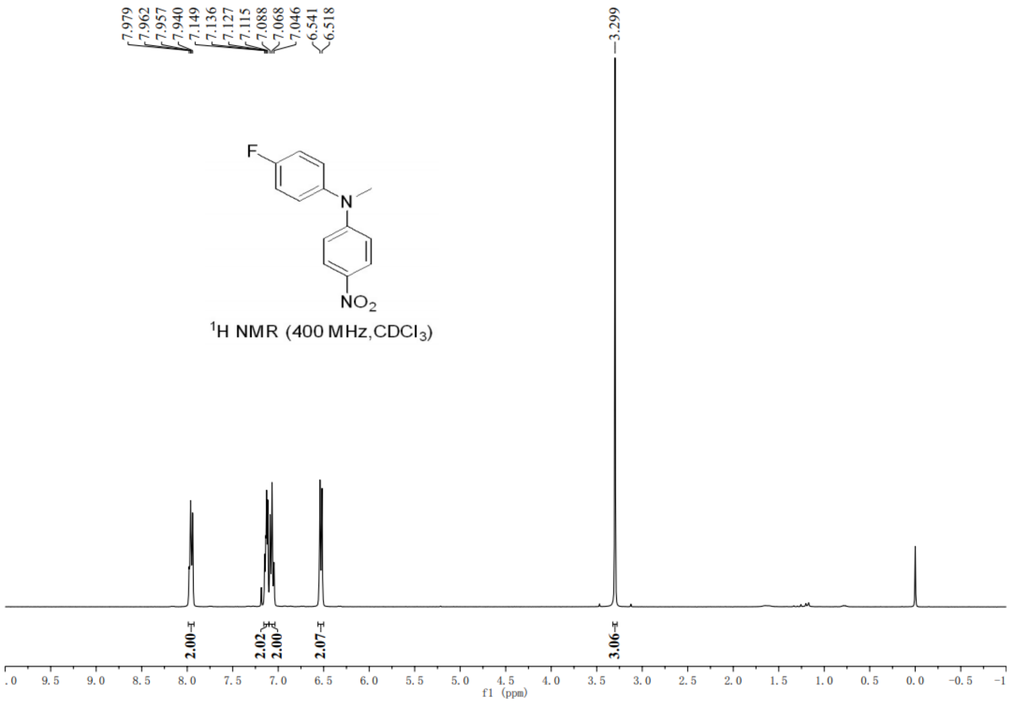

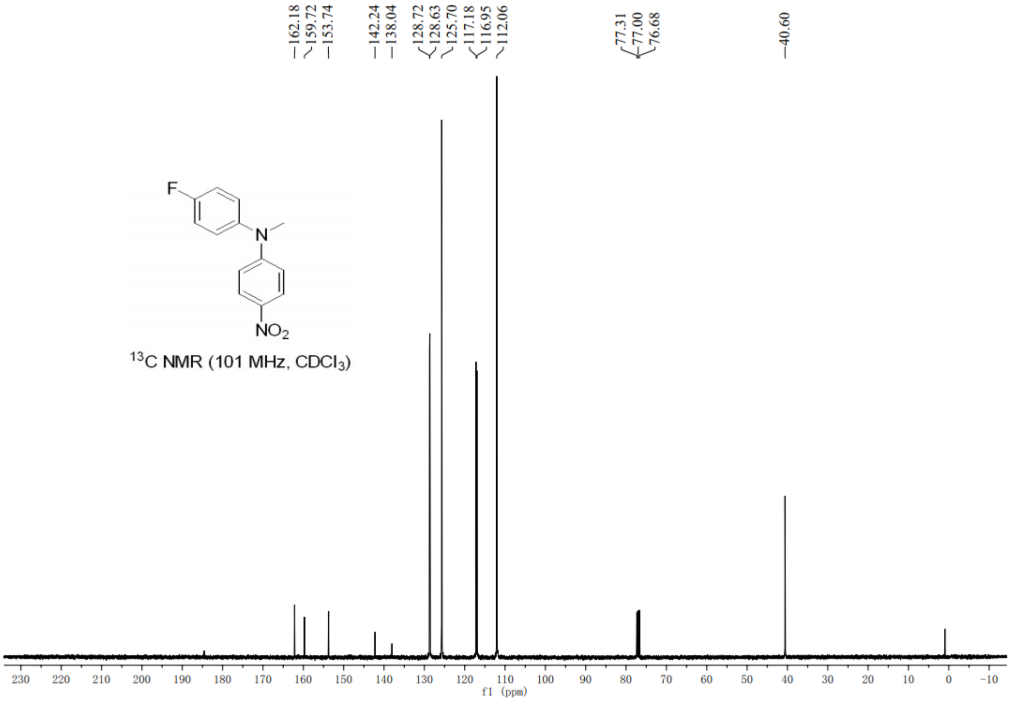

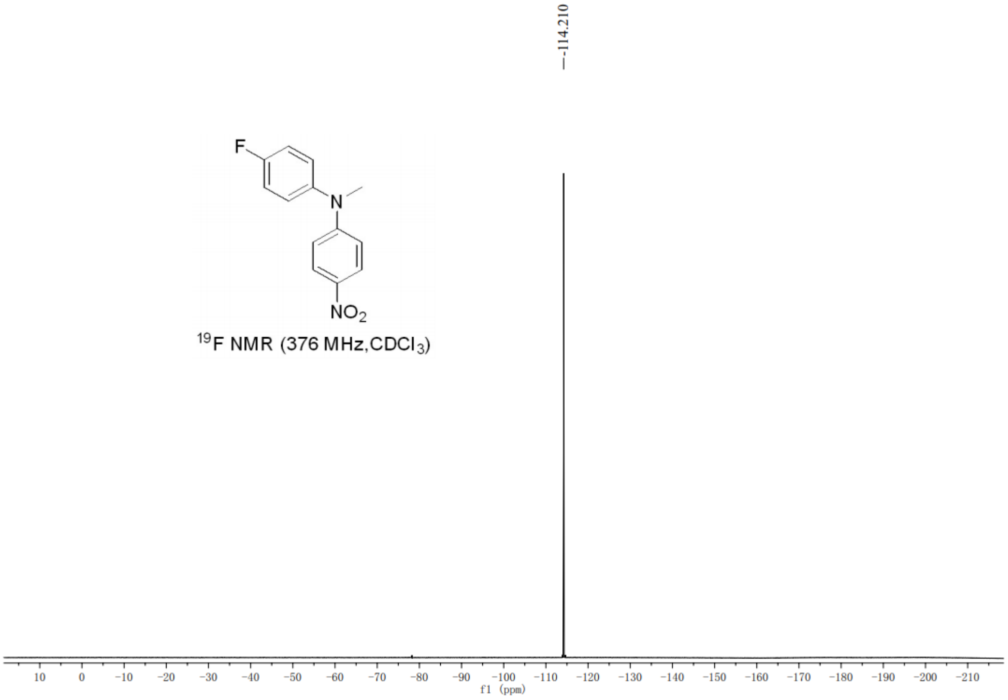
-114.210
F
N
NO2
19F NMR (376 MHz,CDCl3)
10 0 −10 −20 −30 −40 −50 −60 −70 −80 −90 −100 −110 −120 −130 −140 −150 −160 −170 −180 −190 −200 −210
f1 (ppm)

4-chloro-*N*-methyl-*N*-(4-nitrophenyl) aniline (3c-7)

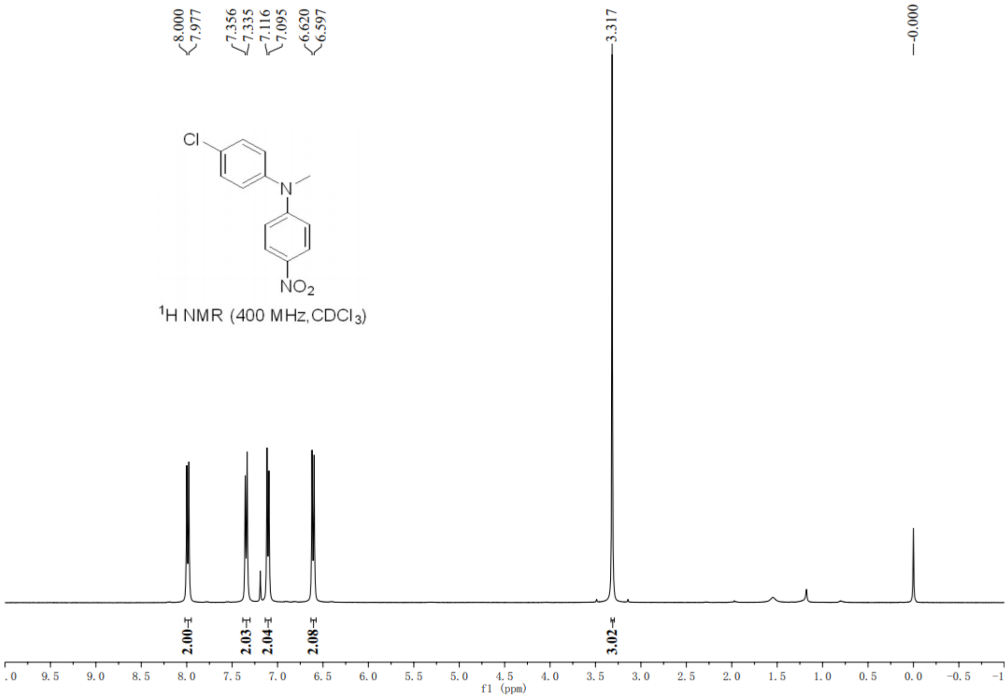

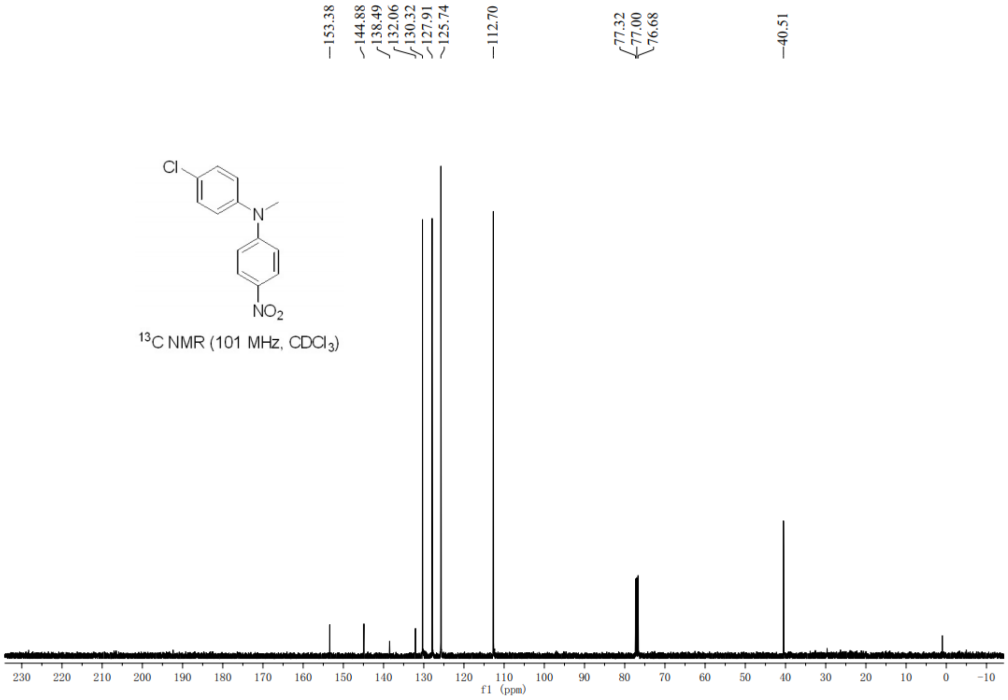

4-bromo-*N*-methyl-*N*-(4-nitrophenyl) aniline (3c-8)

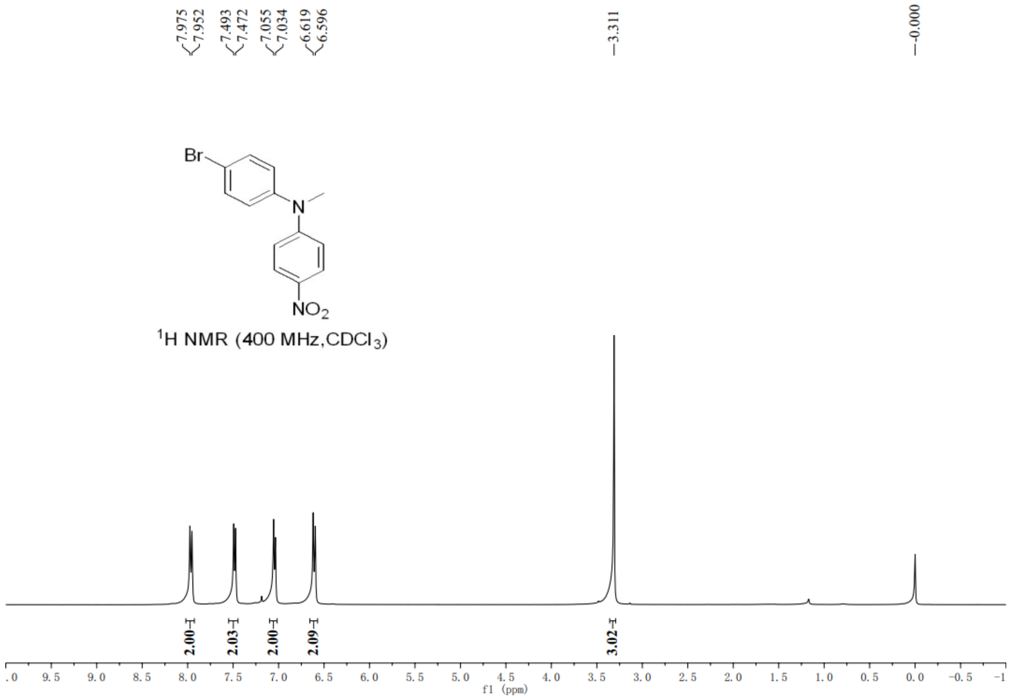

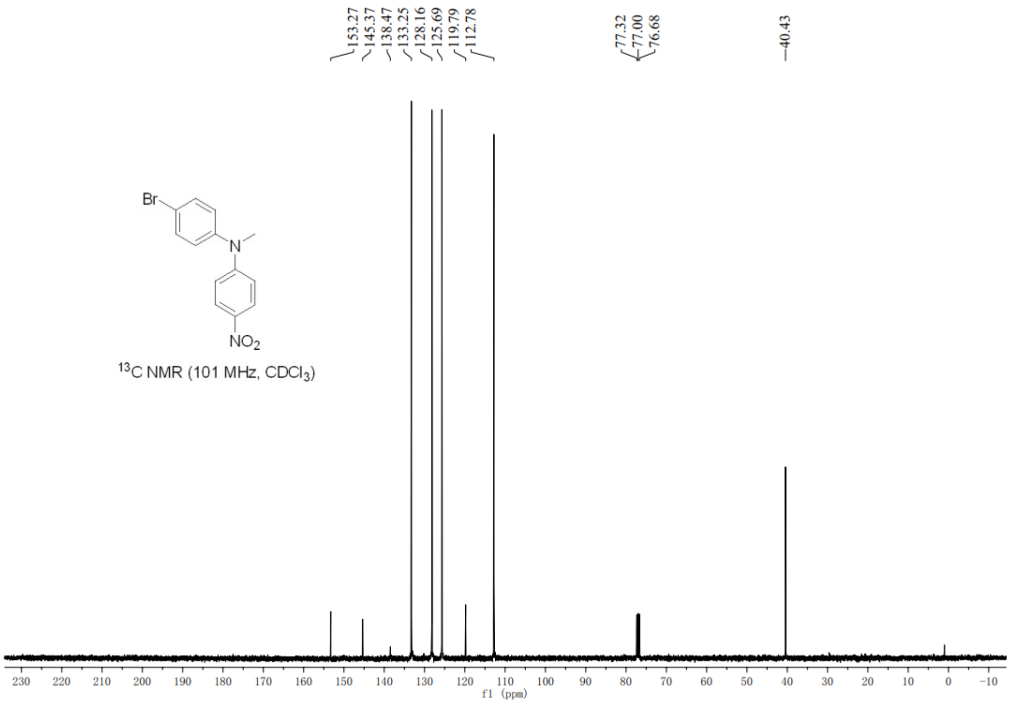

N-methyl-*N*-(4-nitrophenyl)pyridin-3-amine (3c-9)

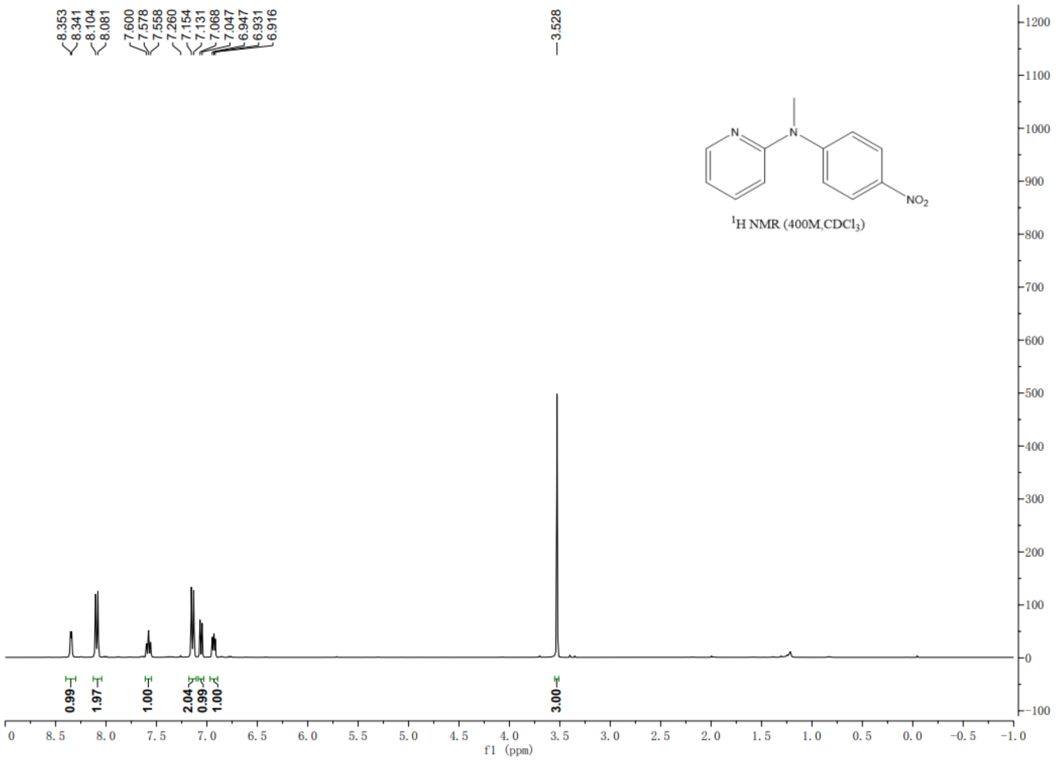

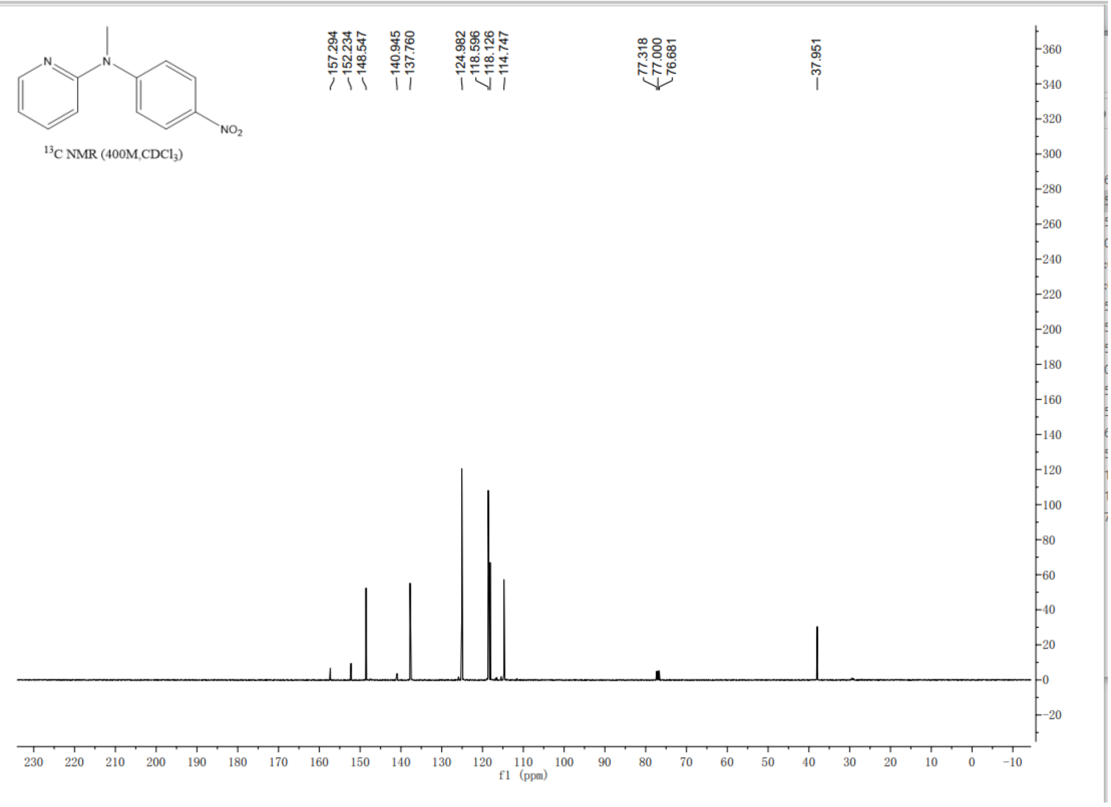

4-nitro-*N*,*N*-diphenylaniline (3c-10)

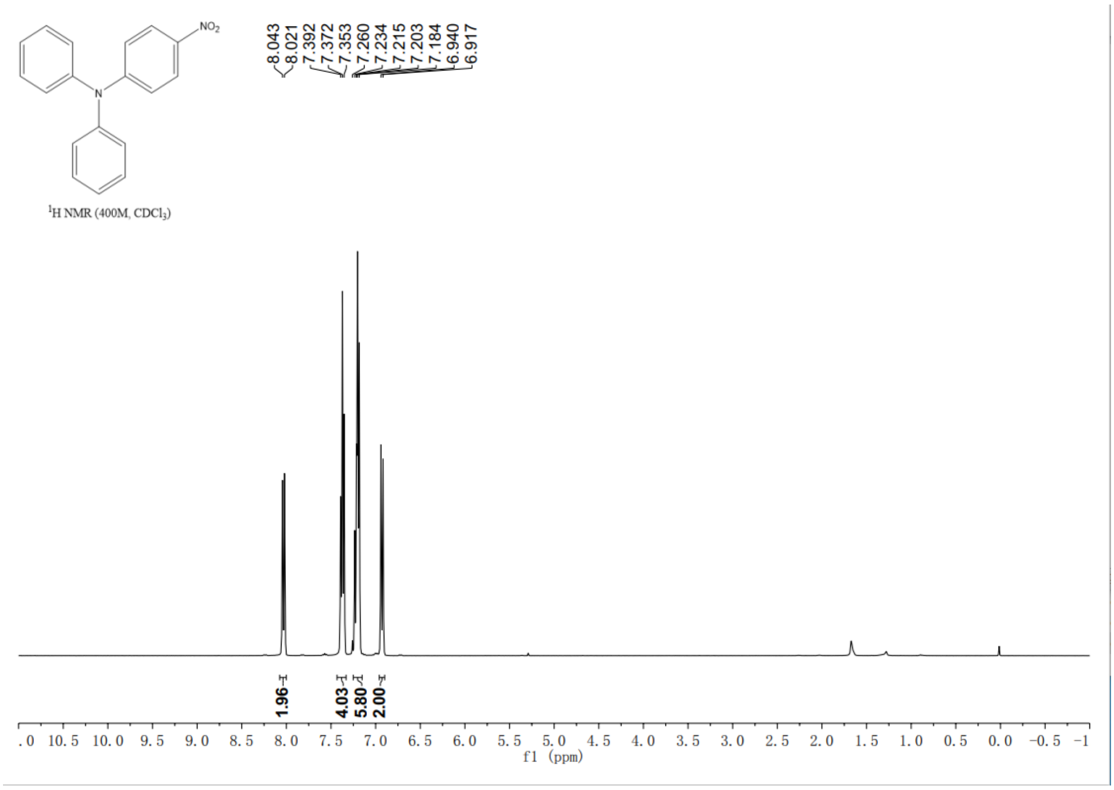

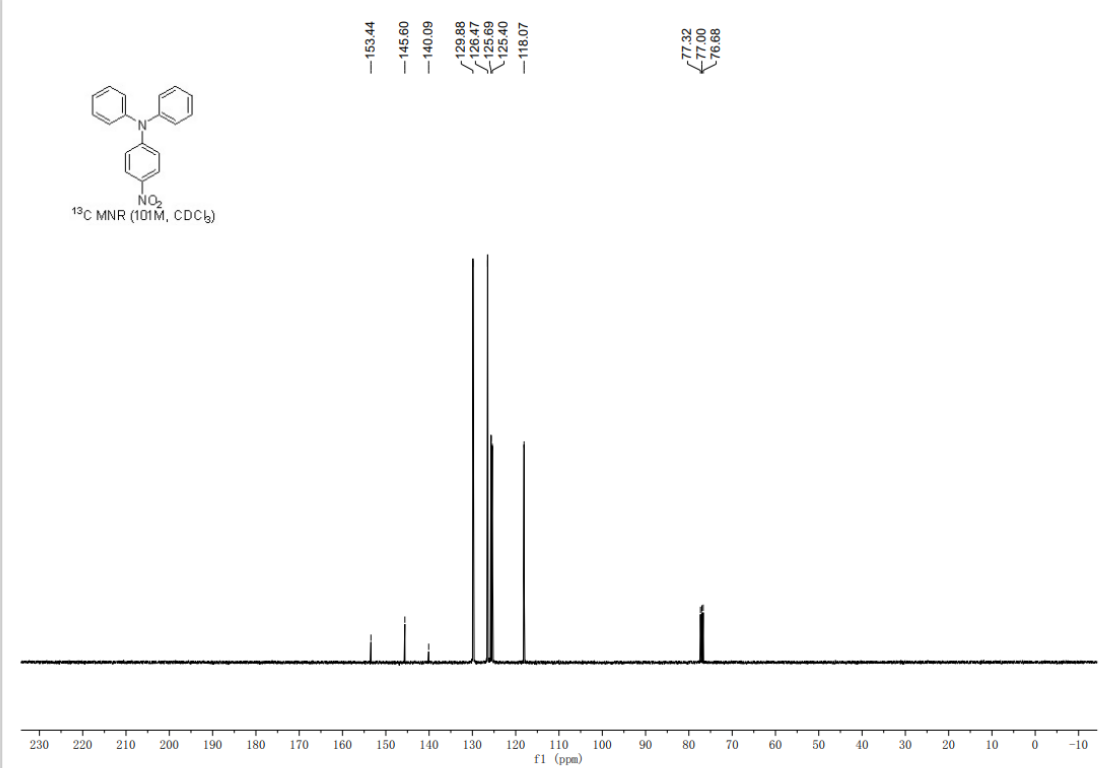

***N*-methyl-*N*-(4-nitrophenyl)pyridin-2-amine (3c-11)**

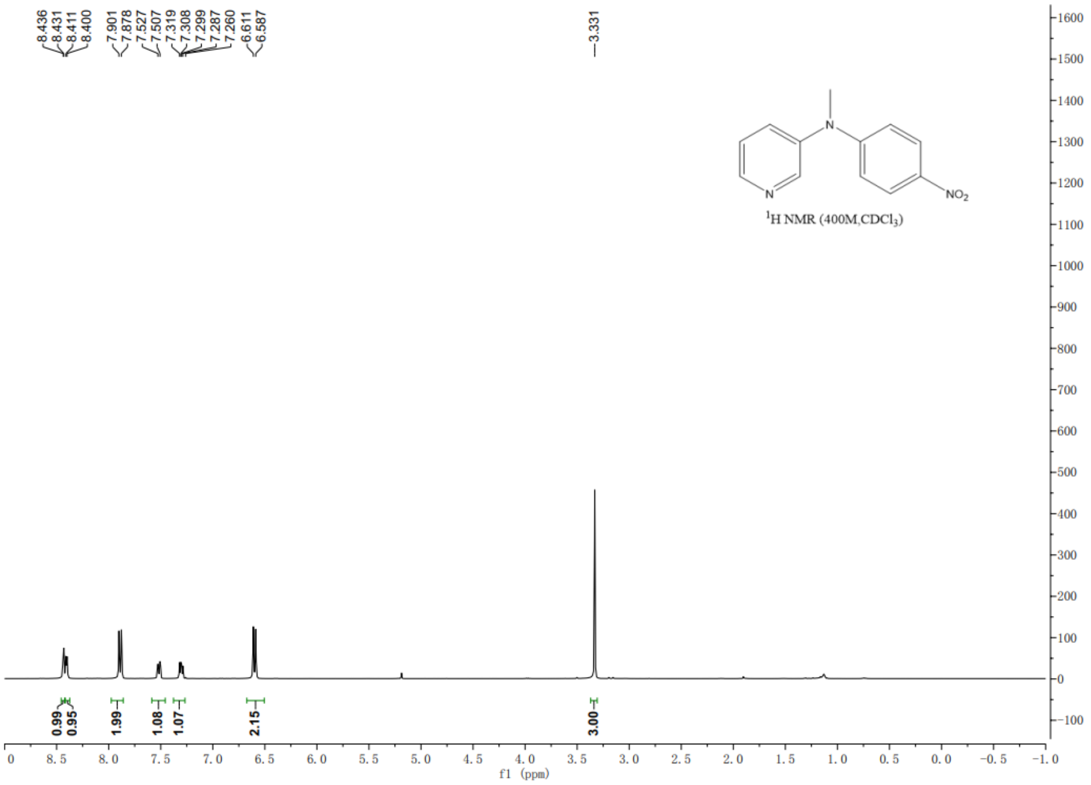

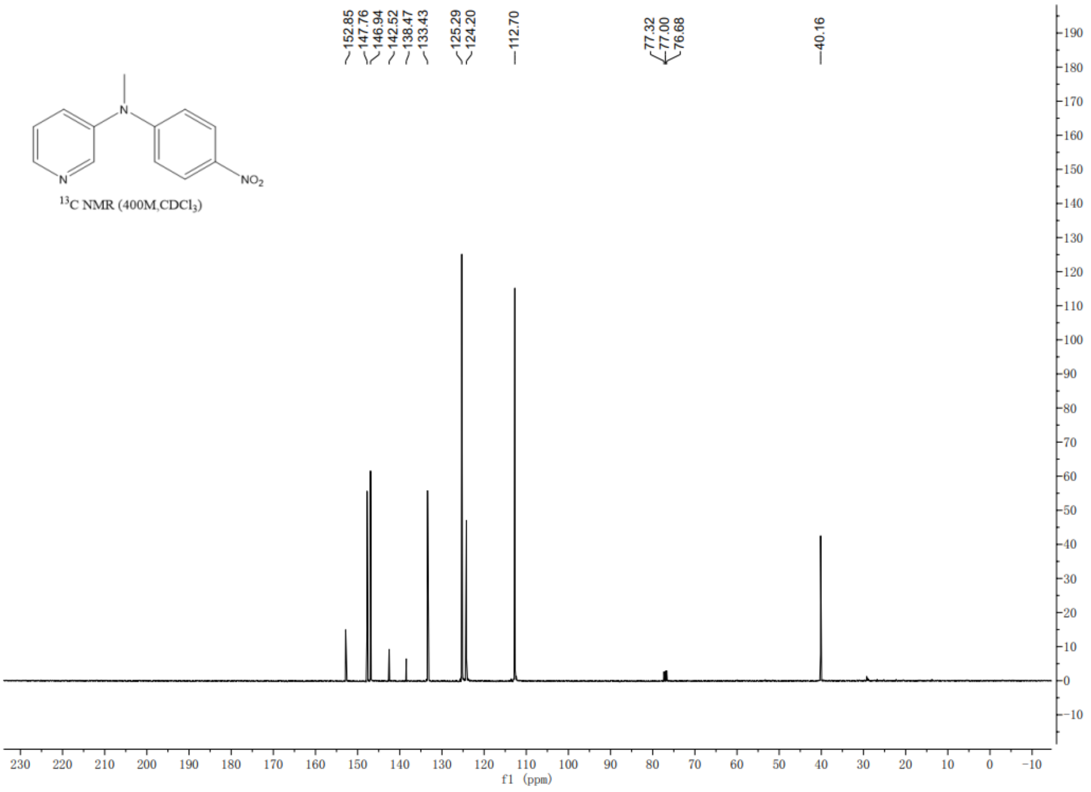

N-methyl-*N*-(4-nitrophenyl)pyridin-4-amine (3c-12)

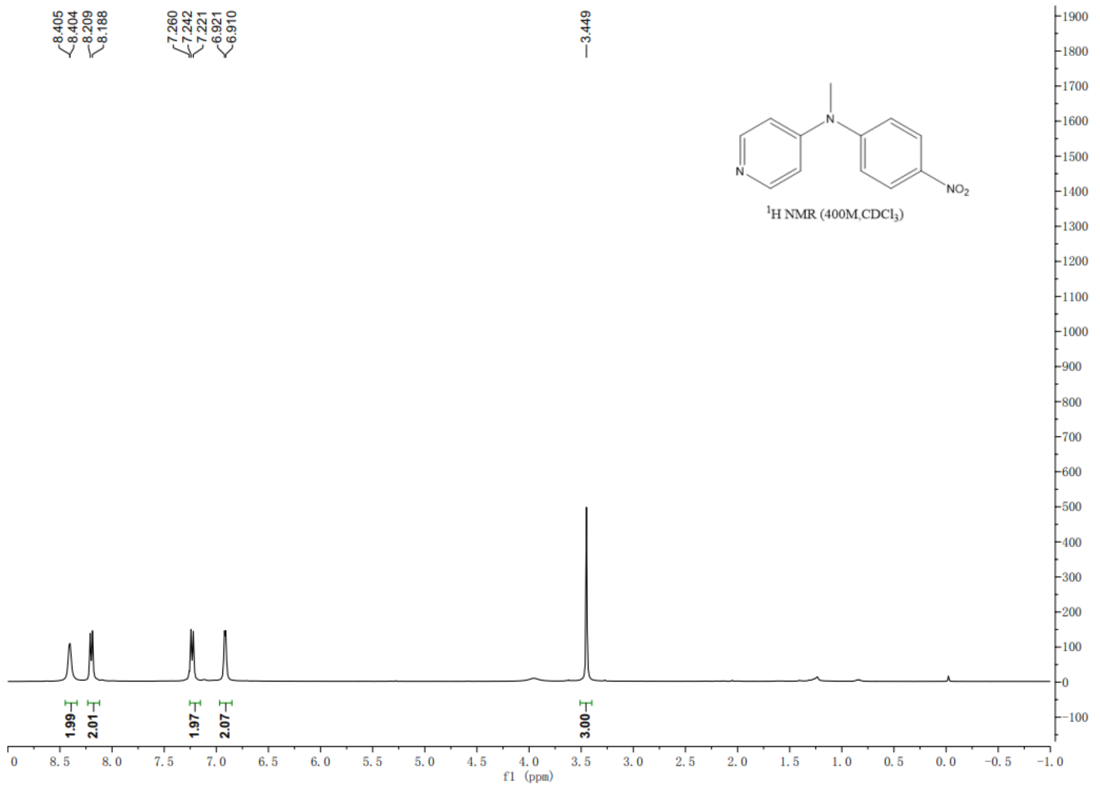

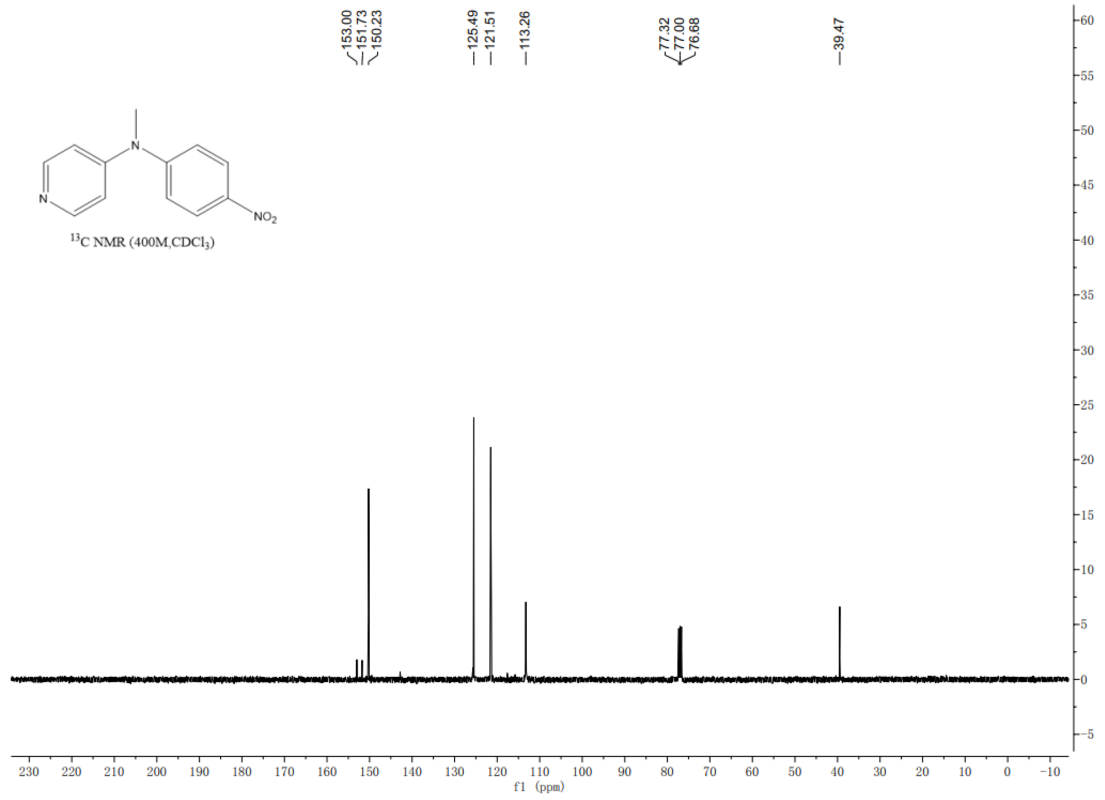

_N_-methyl-_N_-(4-nitrophenyl)pyrazin-2-amine (3c-13)

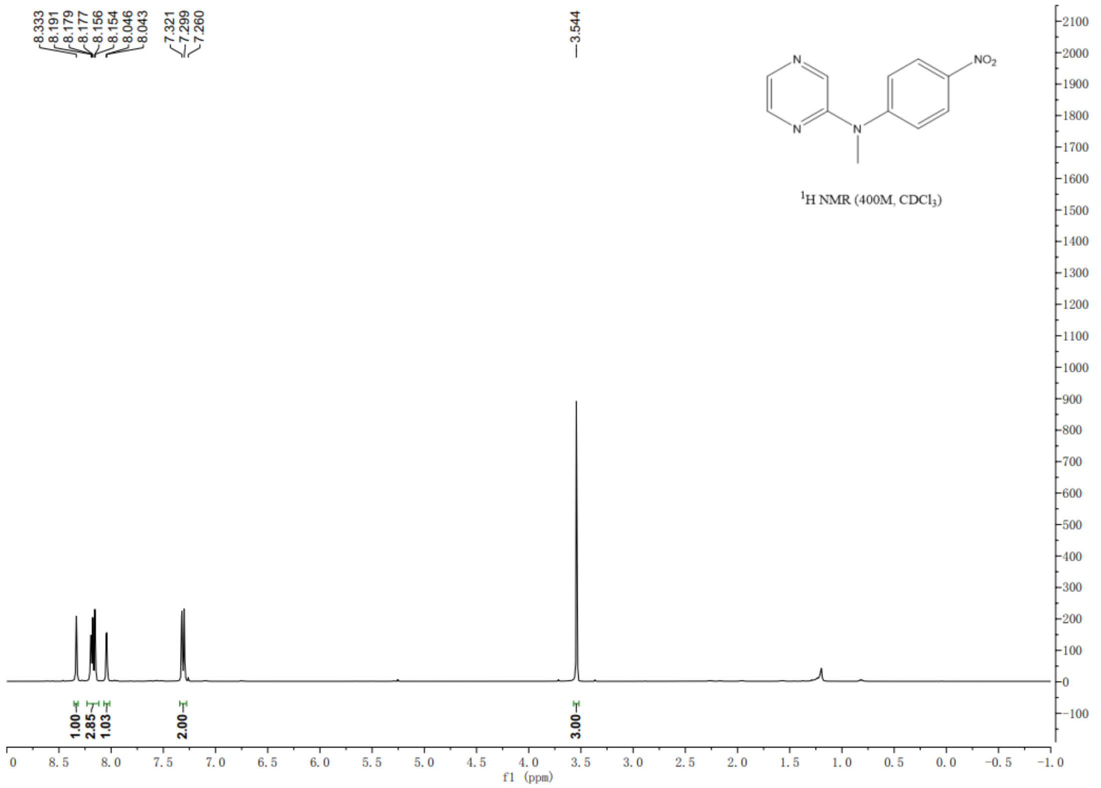

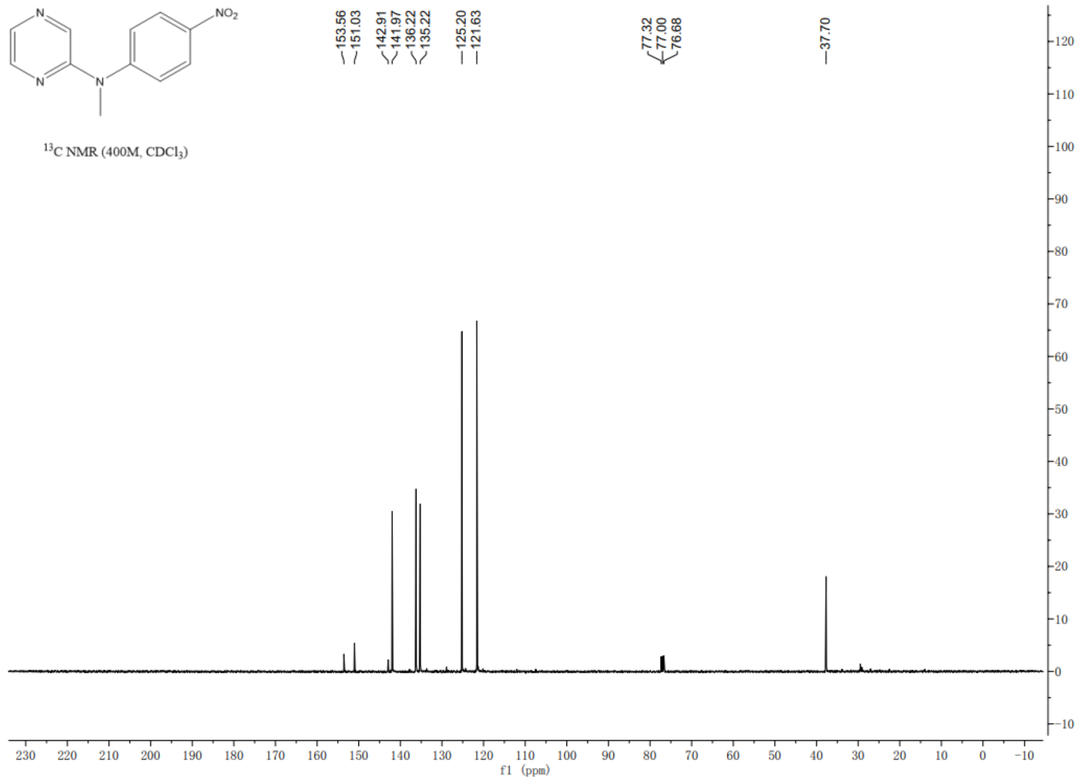

4-ethynyl-*N*-methyl-*N*-(4-nitrophenyl)aniline (3c-14)

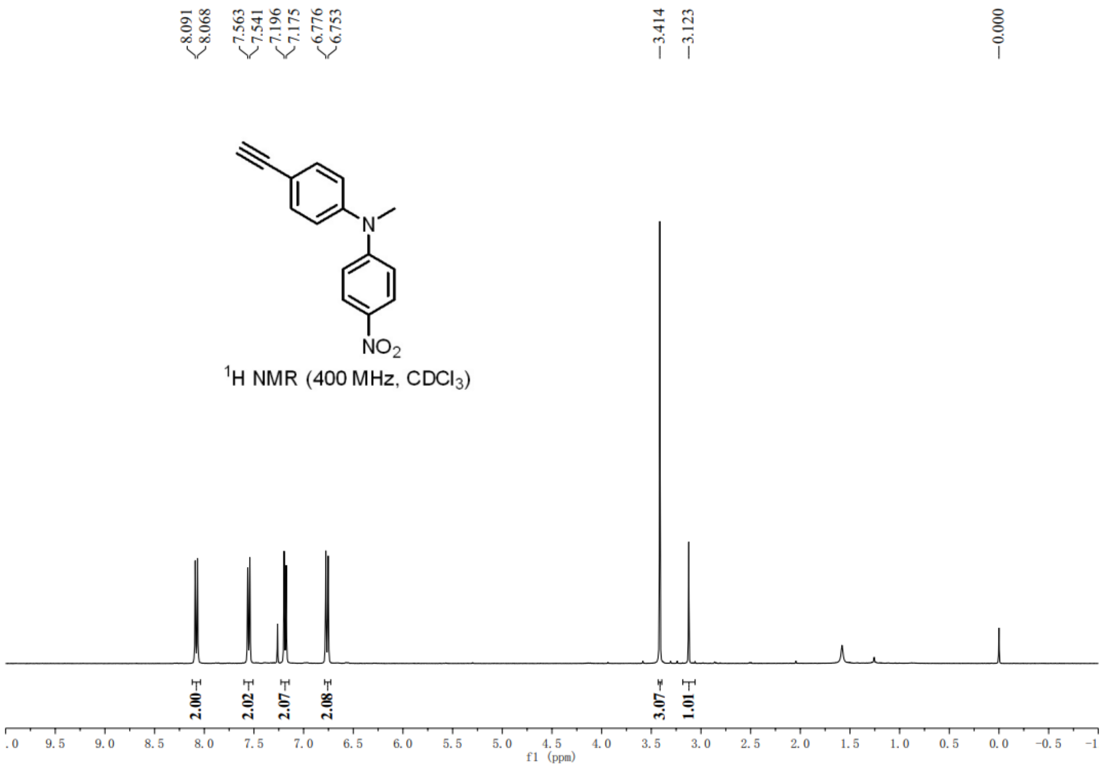

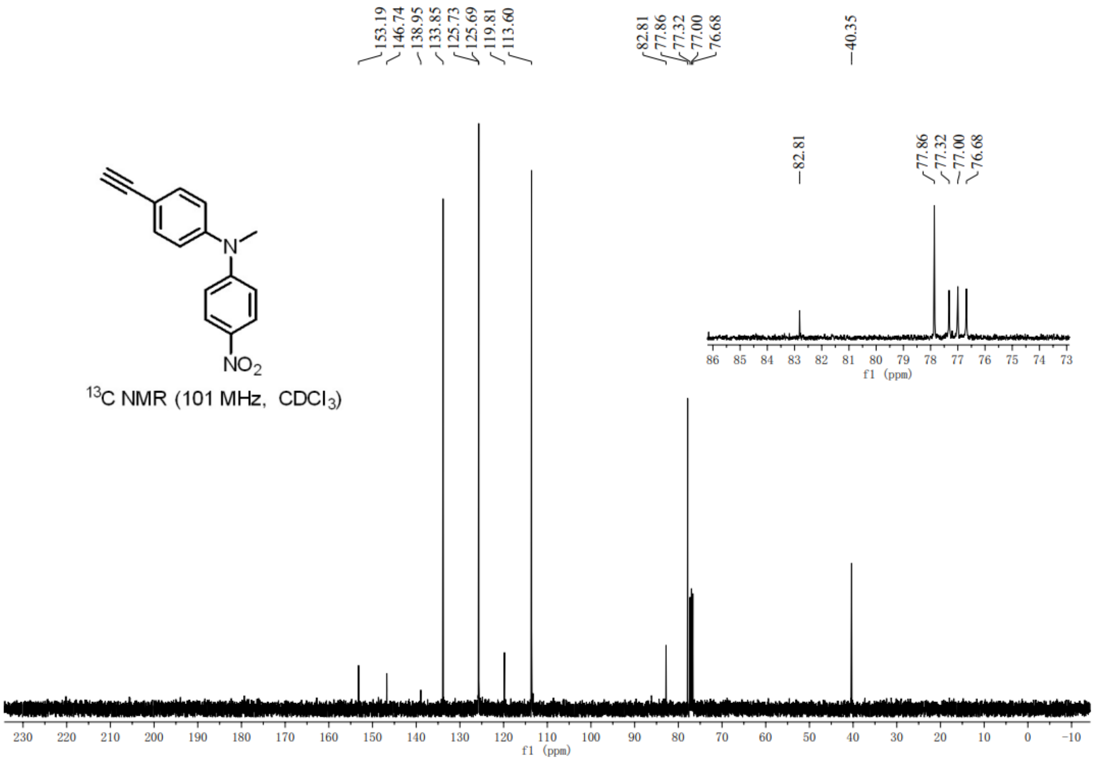

N-(4-(1H-pyrrol-1-yl)phenyl)-*N*-methyl-4-nitroaniline (3c-15)

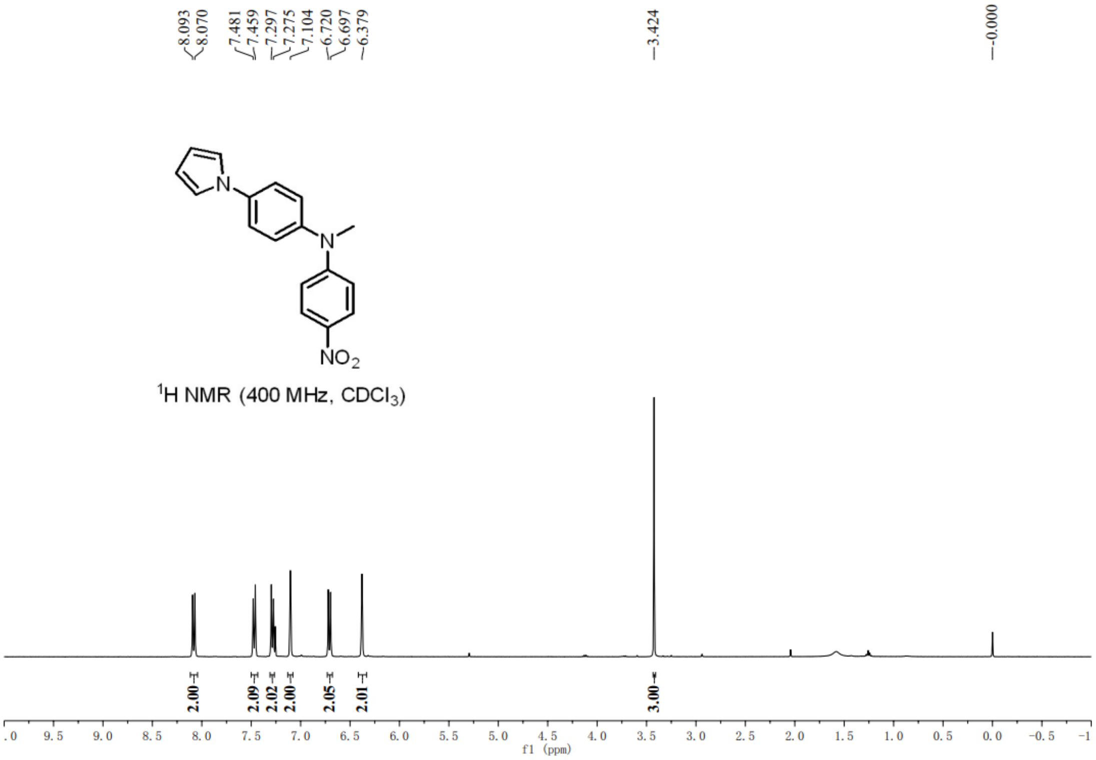

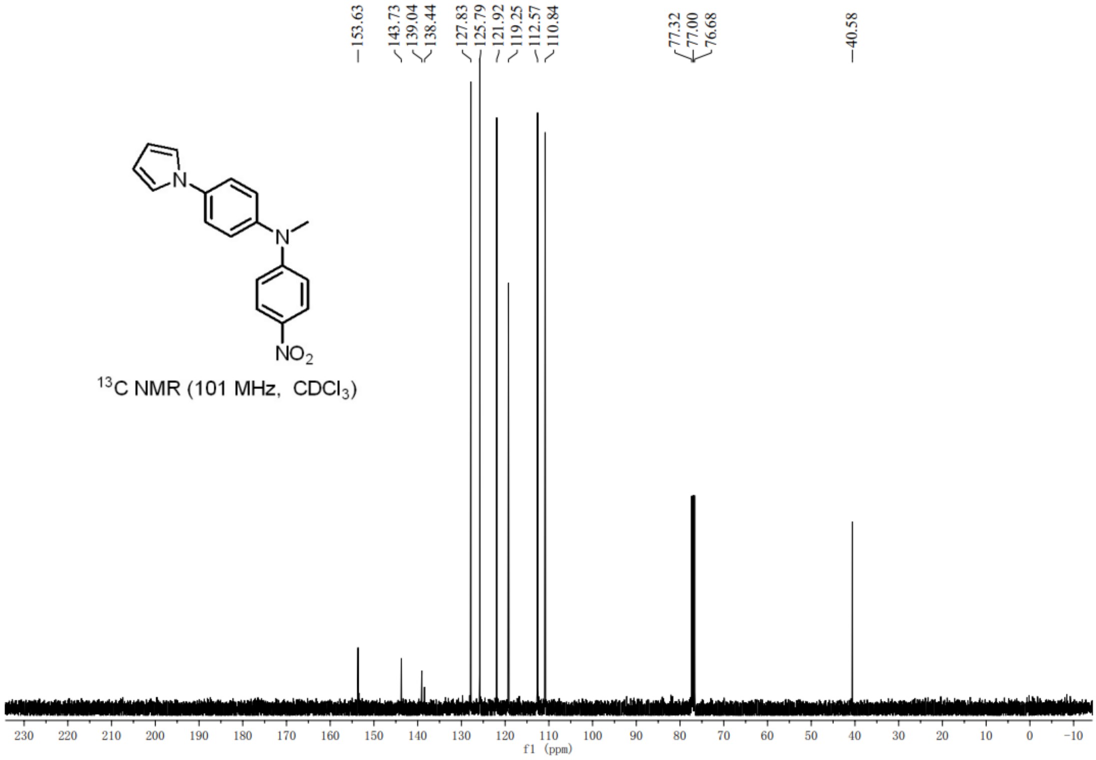

_N_-methyl-4-nitro-_N_-(4-(trifluoromethyl)phenyl)aniline (3c-16)

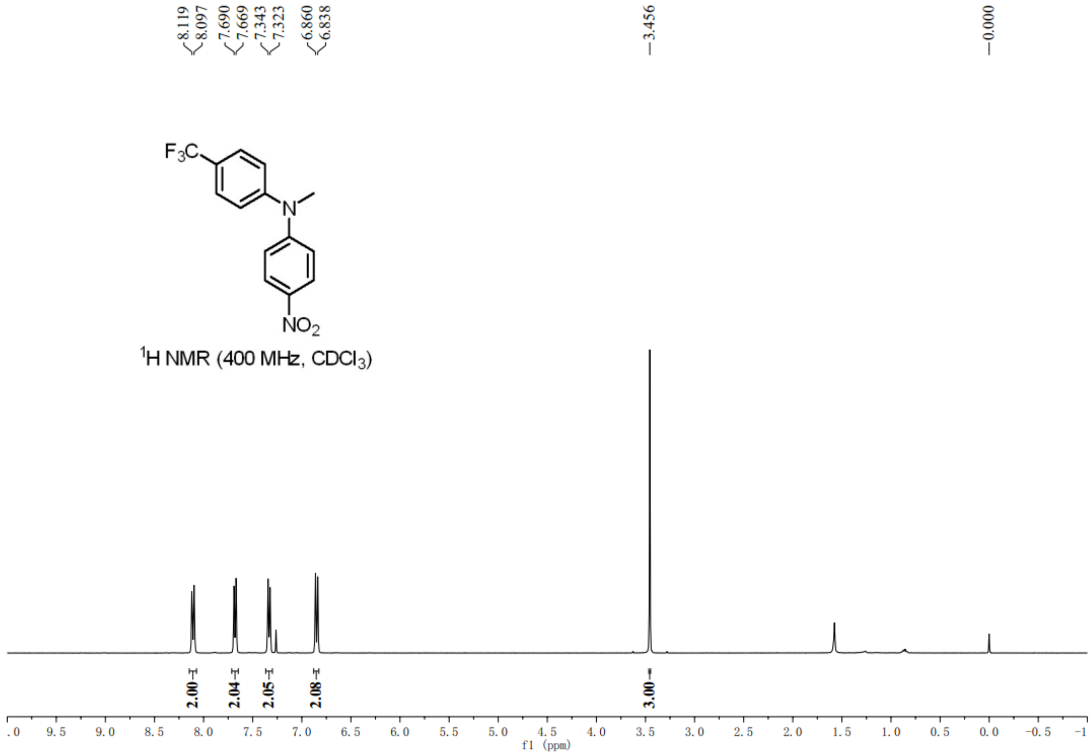

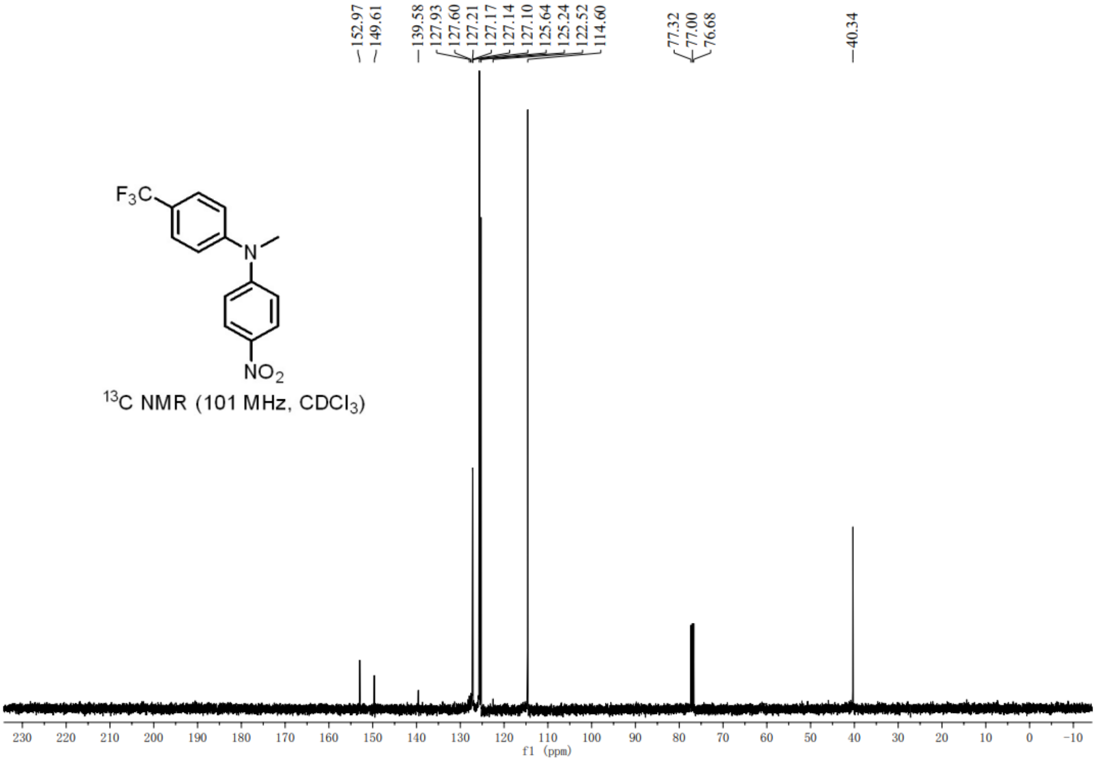

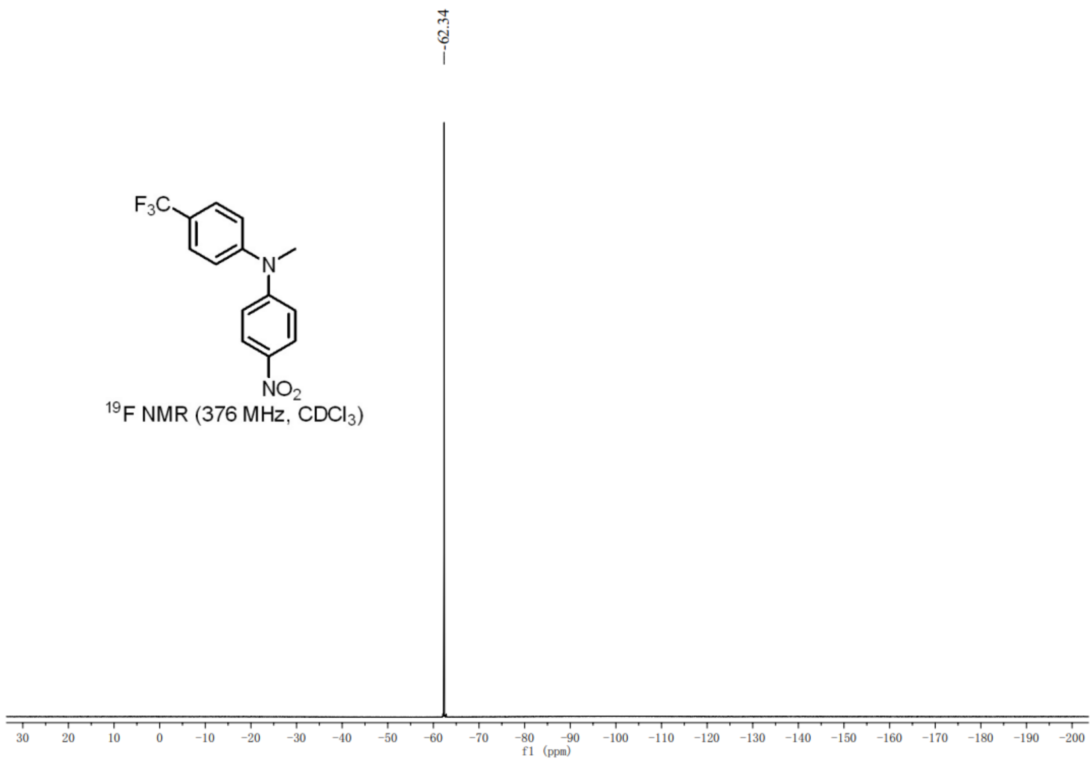
-62.34
F3C
N
NO2
19F NMR (376 MHz, CDCl3)
30 20 10 0 -10 -20 -30 -40 -50 -60 -70 -80 -90 -100 -110 -120 -130 -140 -150 -160 -170 -180 -190 -200
f1 (ppm)

N-methyl-4-nitro-N-(4-(trifluoromethoxy)phenyl)aniline (3c-17)

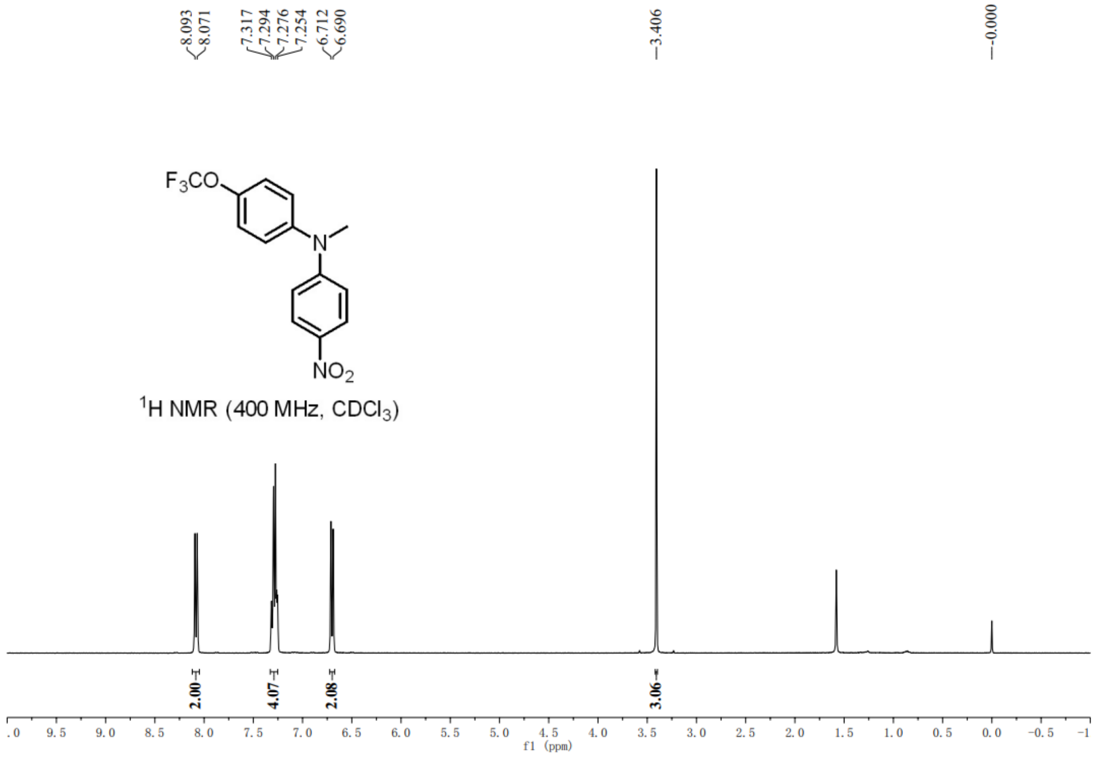

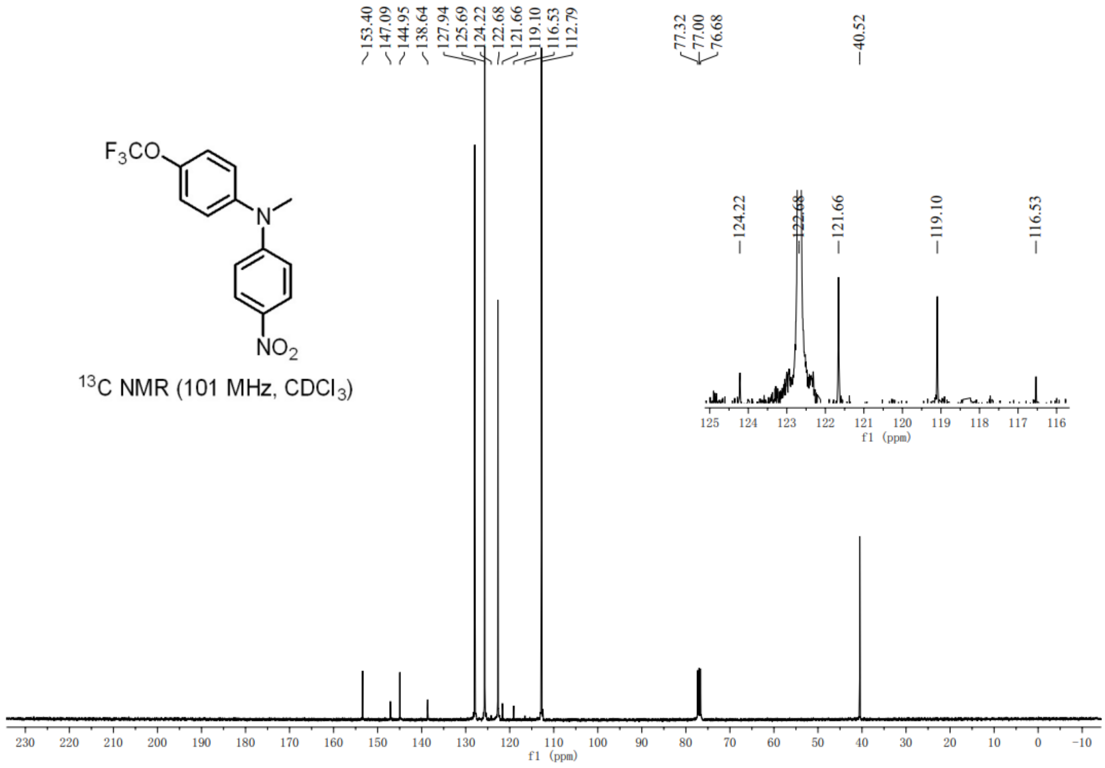

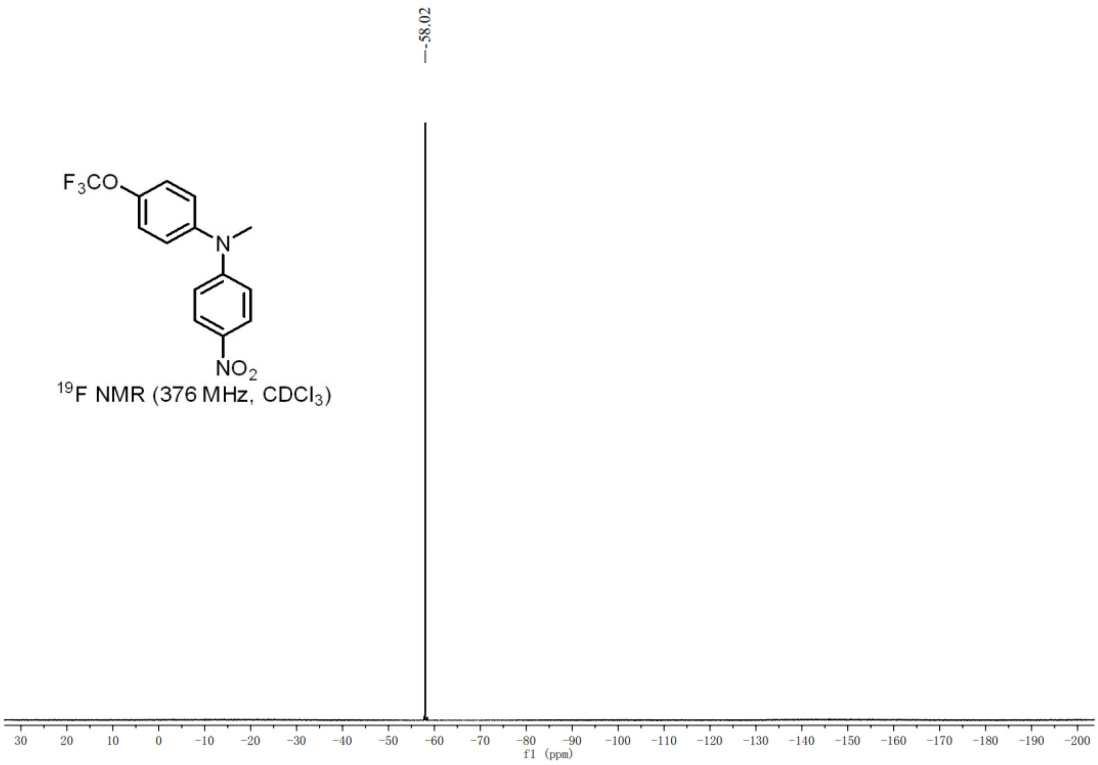
-58.02
F3CO
N
NO2
19F NMR (376 MHz, CDCl3)
30 20 10 0 −10 −20 −30 −40 −50 −60 −70 −80 −90 −100 −110 −120 −130 −140 −150 −160 −170 −180 −190 −200
f1 (ppm)

N-methyl-4-(methylthio)-N-(4-nitrophenyl)aniline (3c-18)

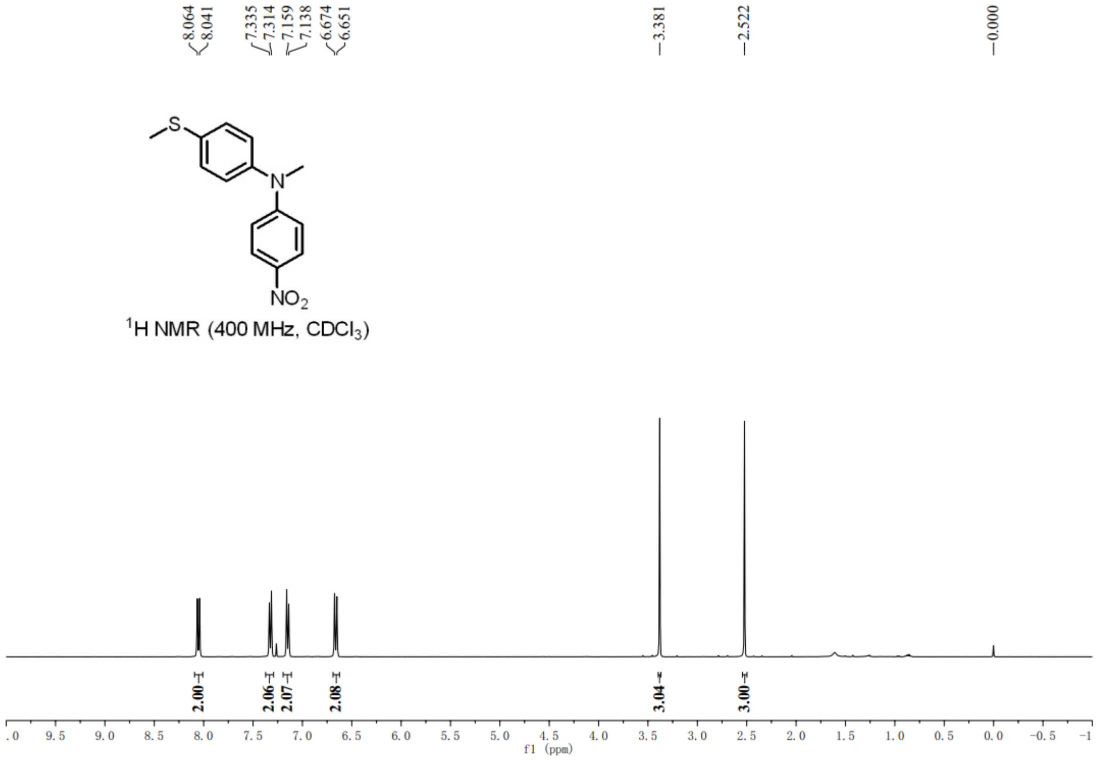

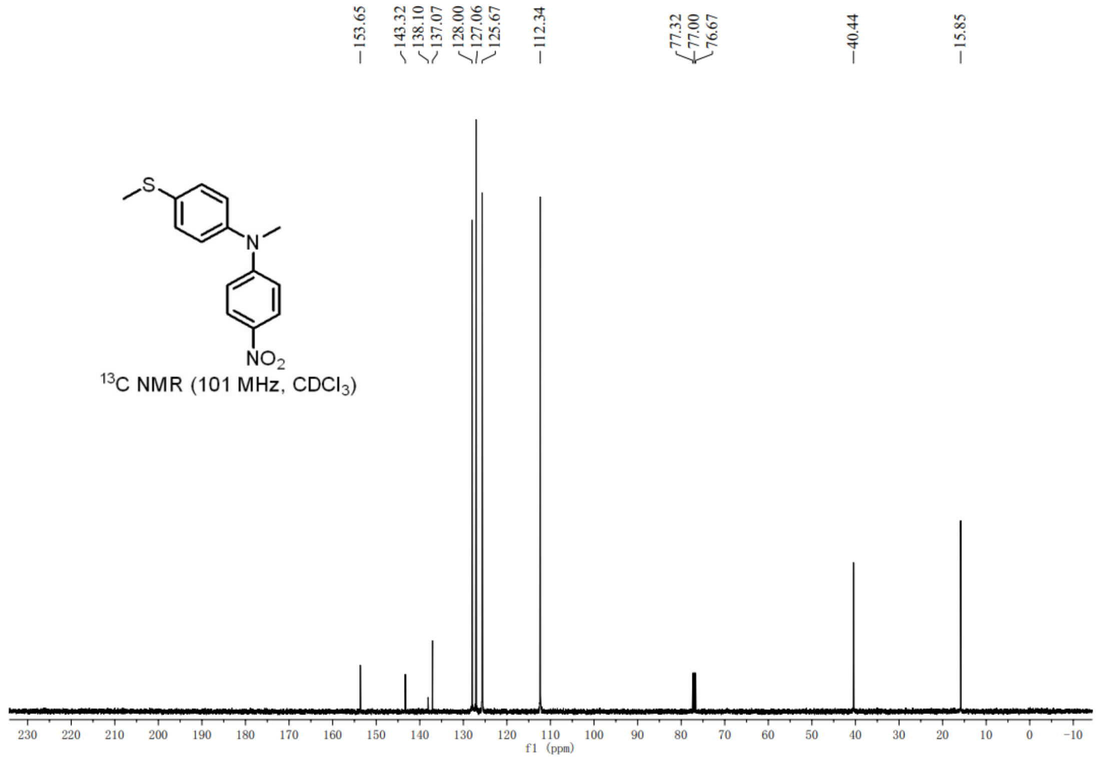

1-(4-(methyl(4-nitrophenyl)amino)phenyl)ethan-1-one (3c-19)

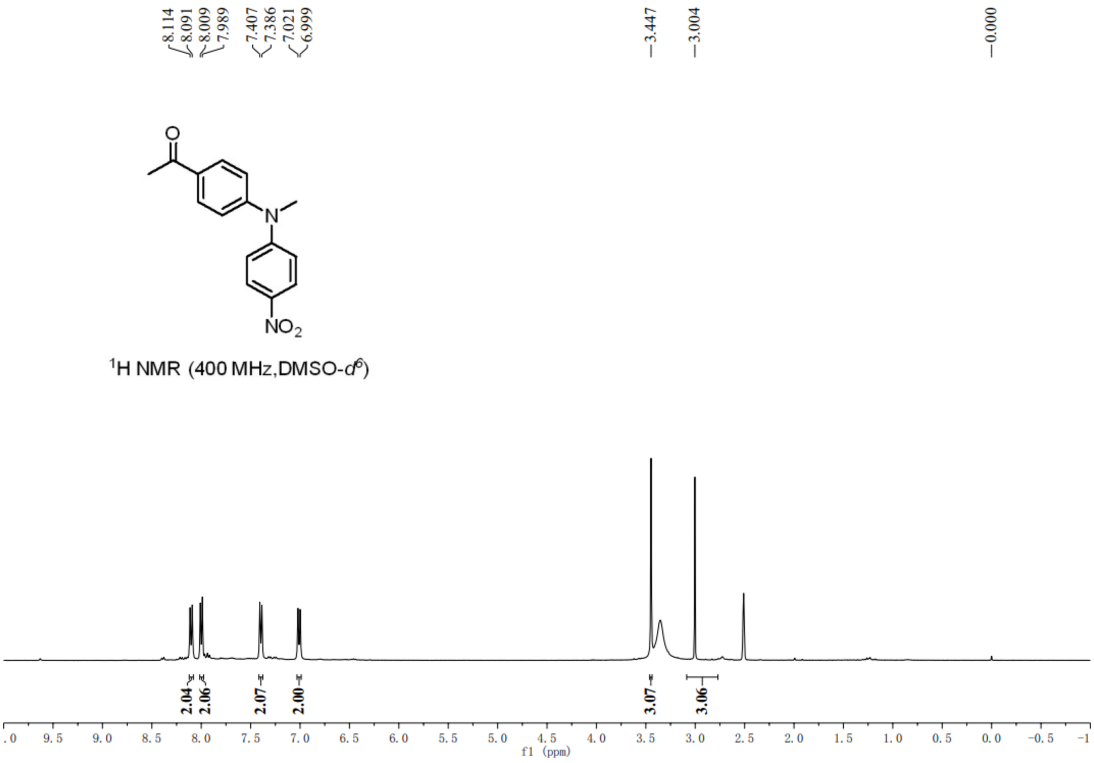

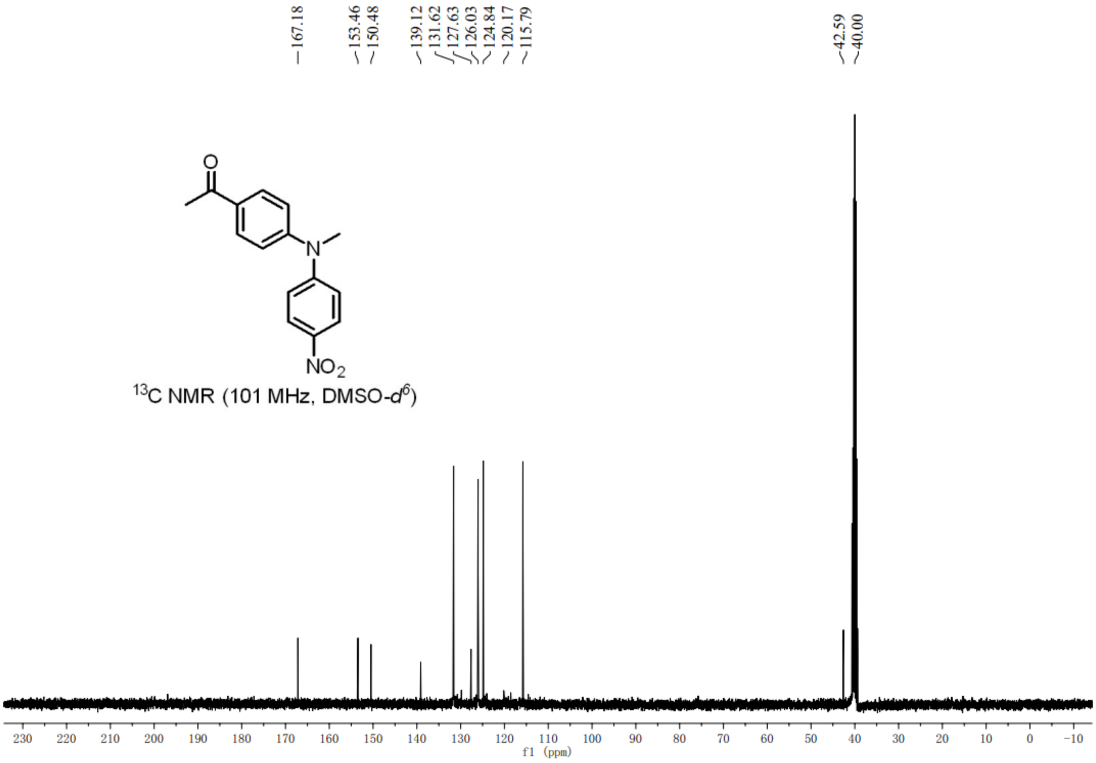

3-((4-nitrophenyl)(phenyl)amino)propan-1-ol (3c-20)

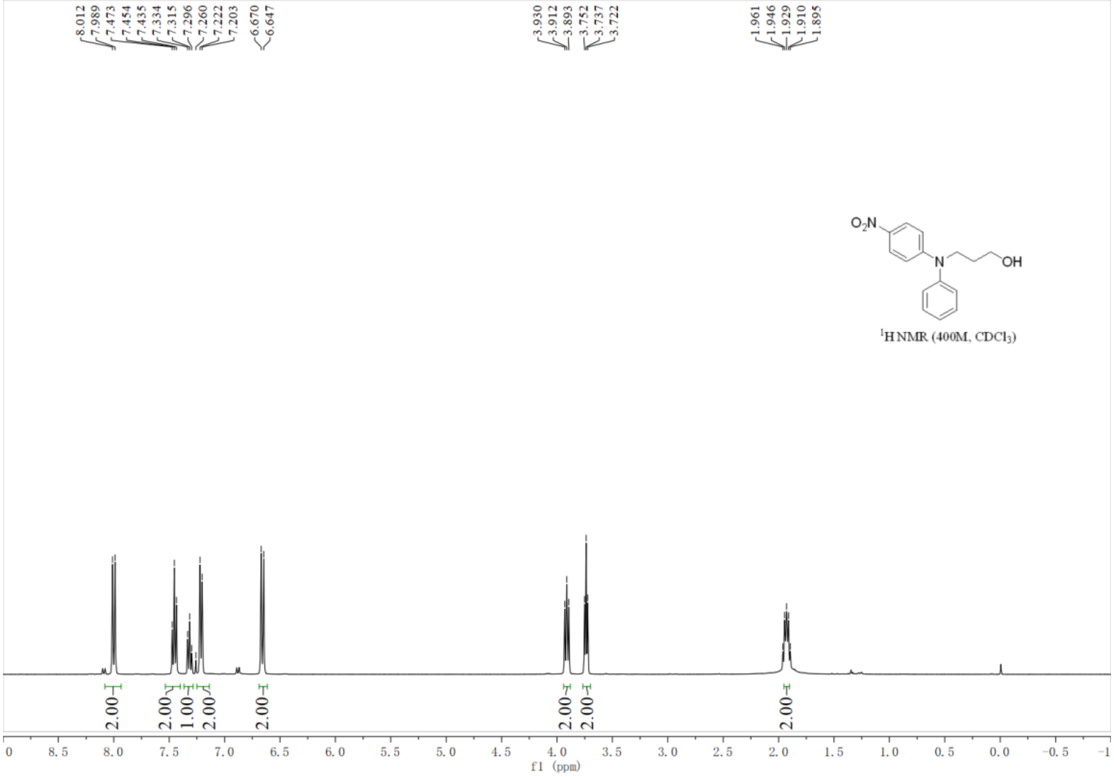

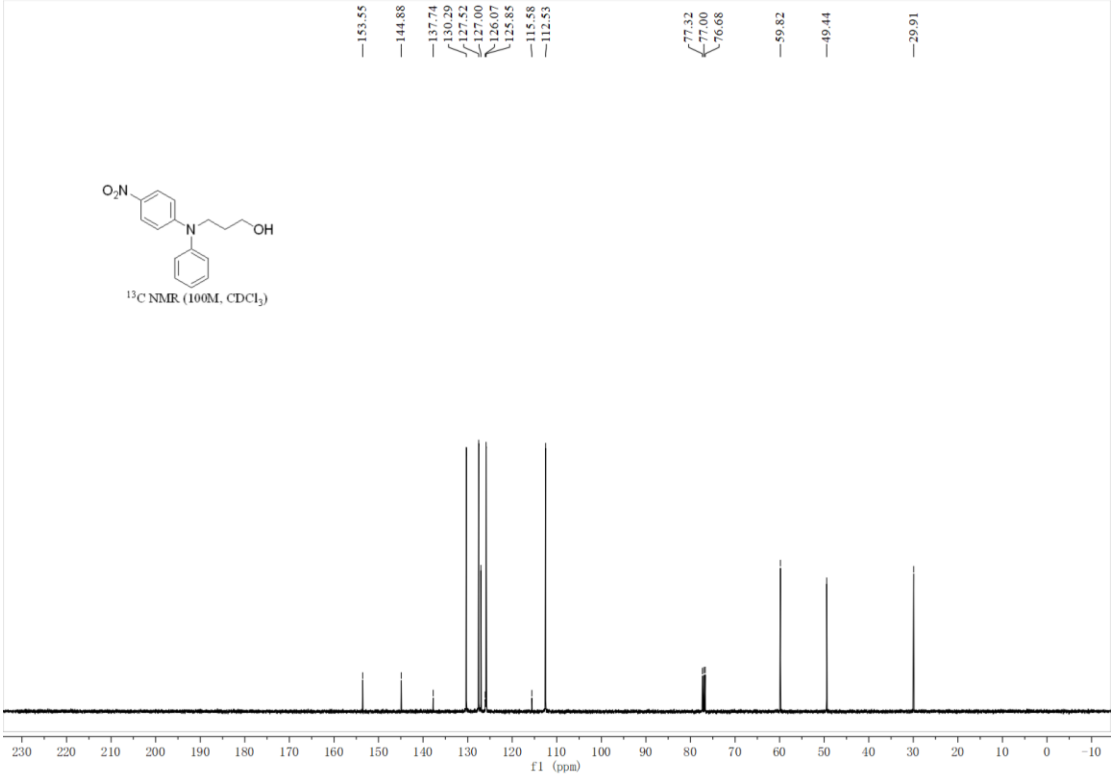

N-(2-methoxyethyl)-4-nitro-*N*-phenylaniline (3c-21)

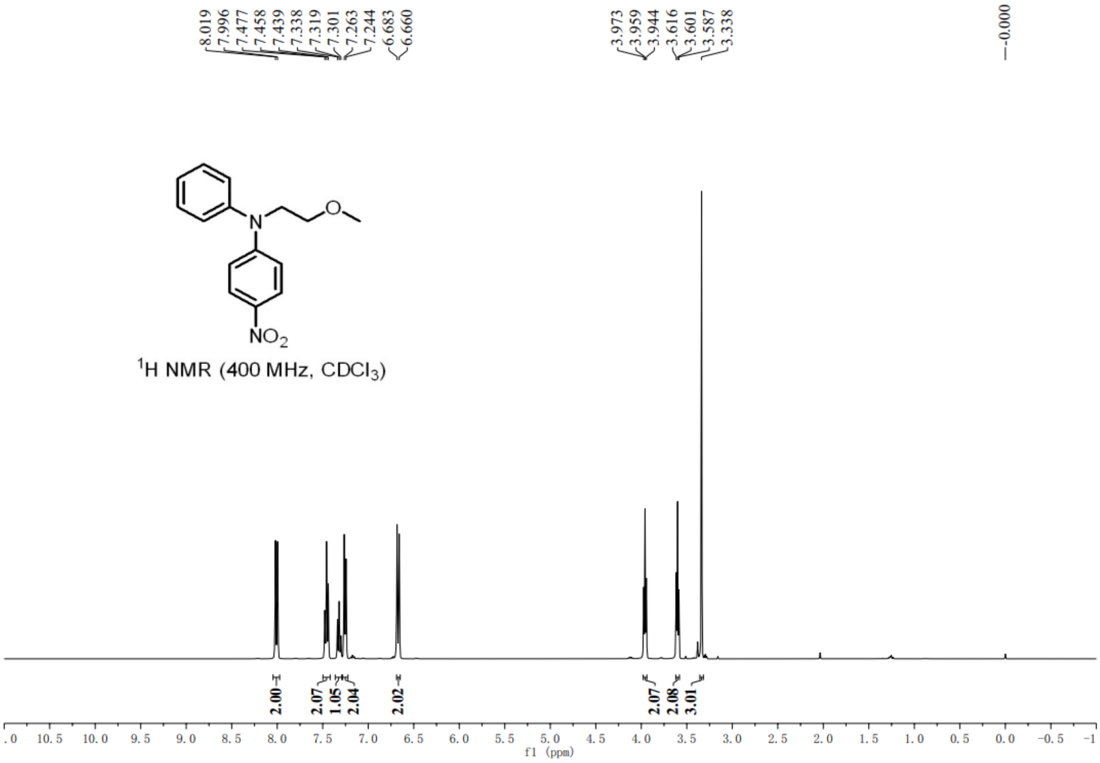

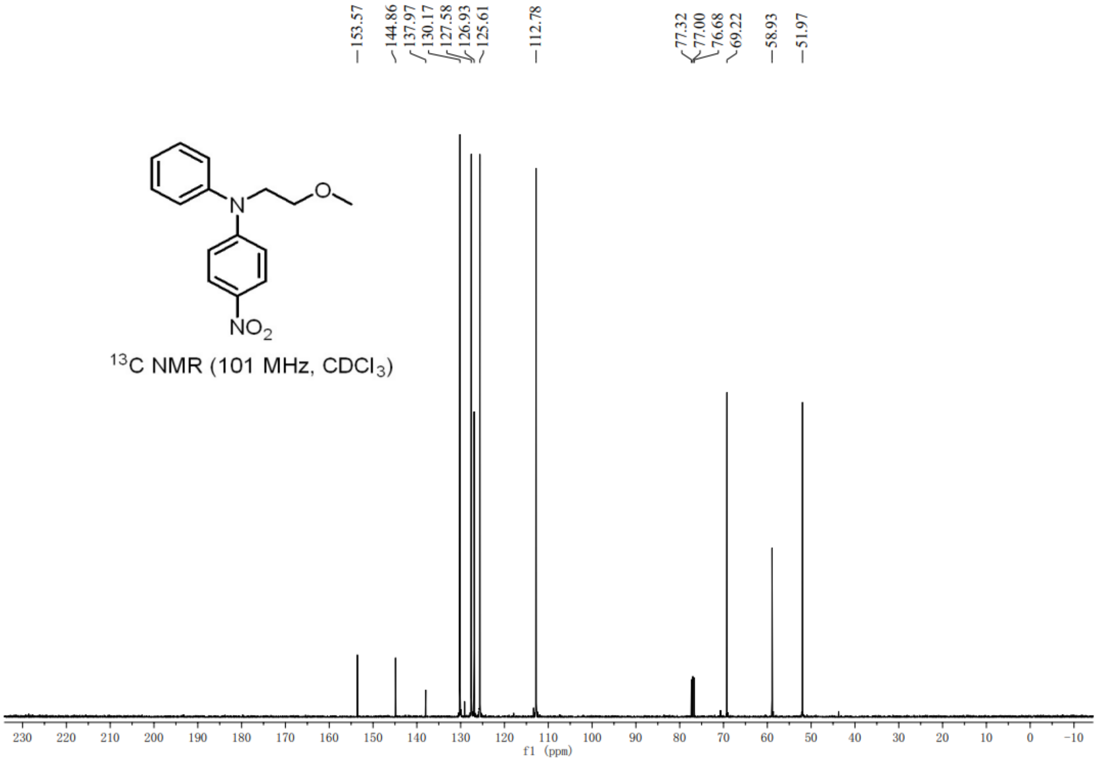

4-nitro-N-pentyl-N-phenylaniline (3c-22)

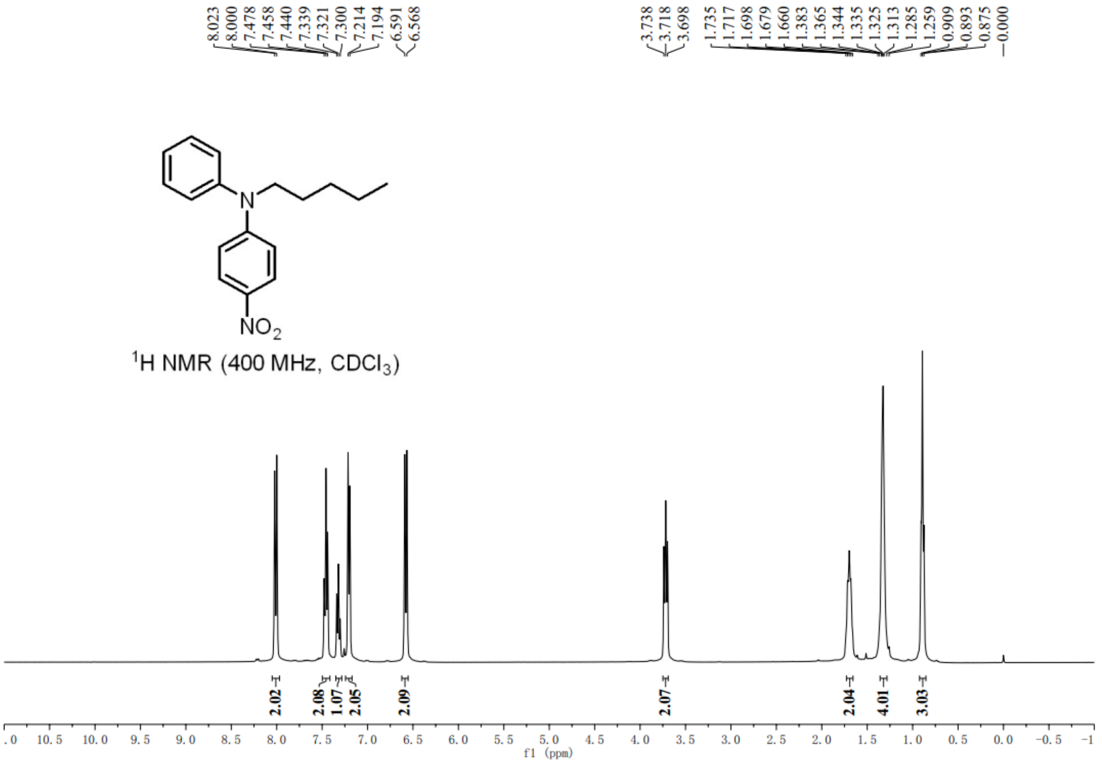

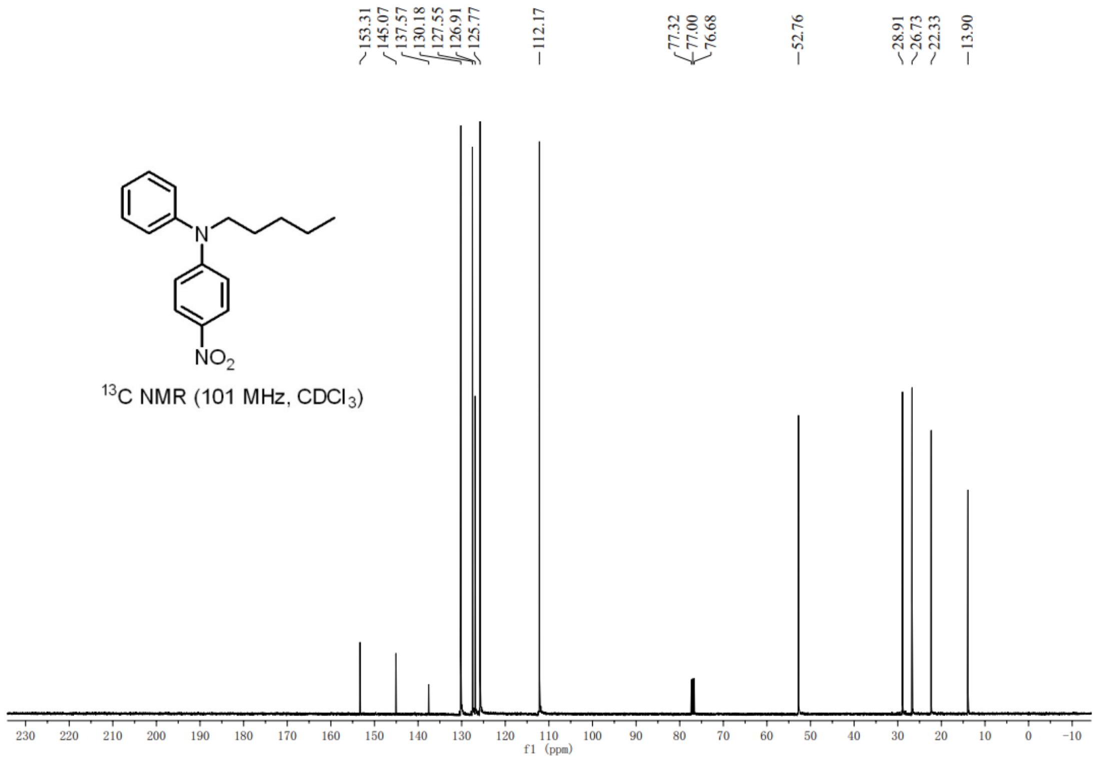

4-bromo-*N*-isobutyl-*N*-(4-nitrophenyl)aniline (3c-23)

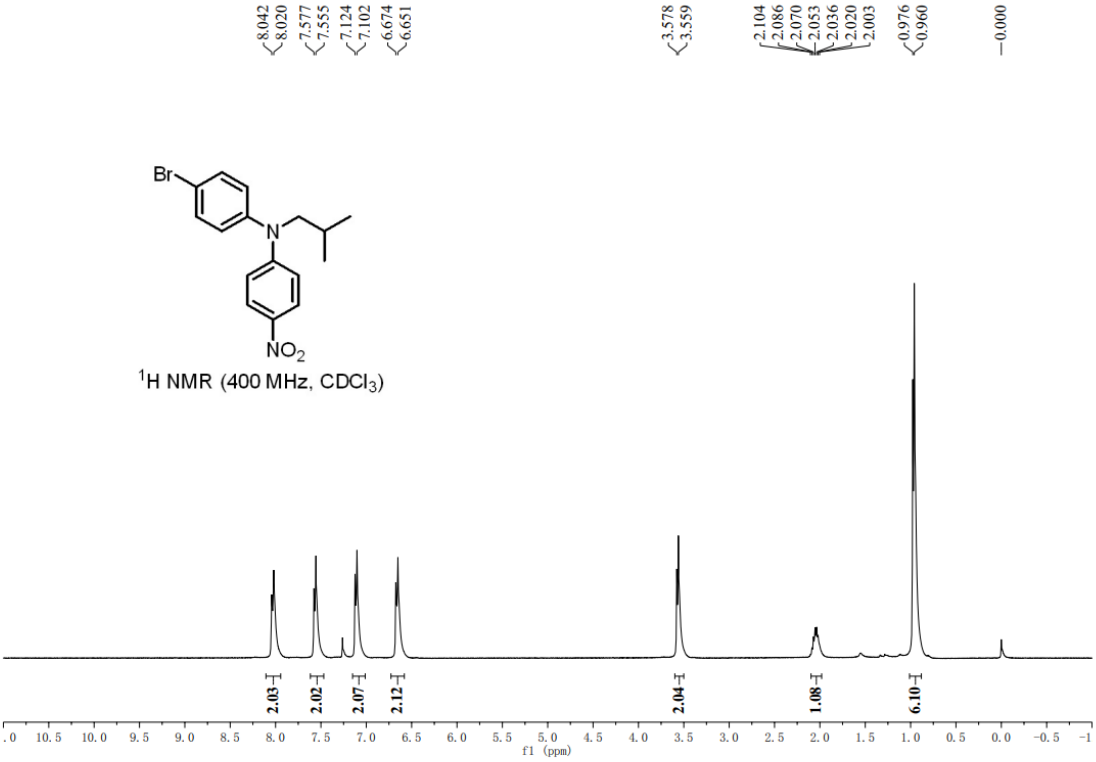

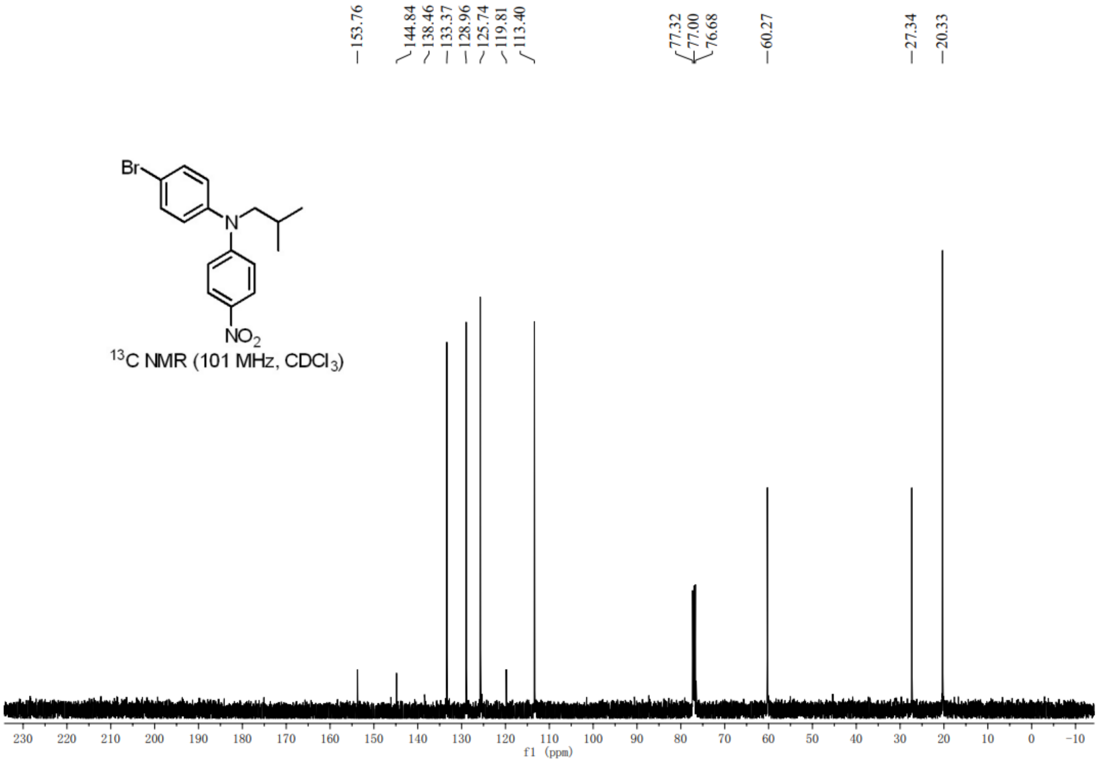

3-((4-nitrophenyl)(phenyl)amino)propanoic acid (3c-24)

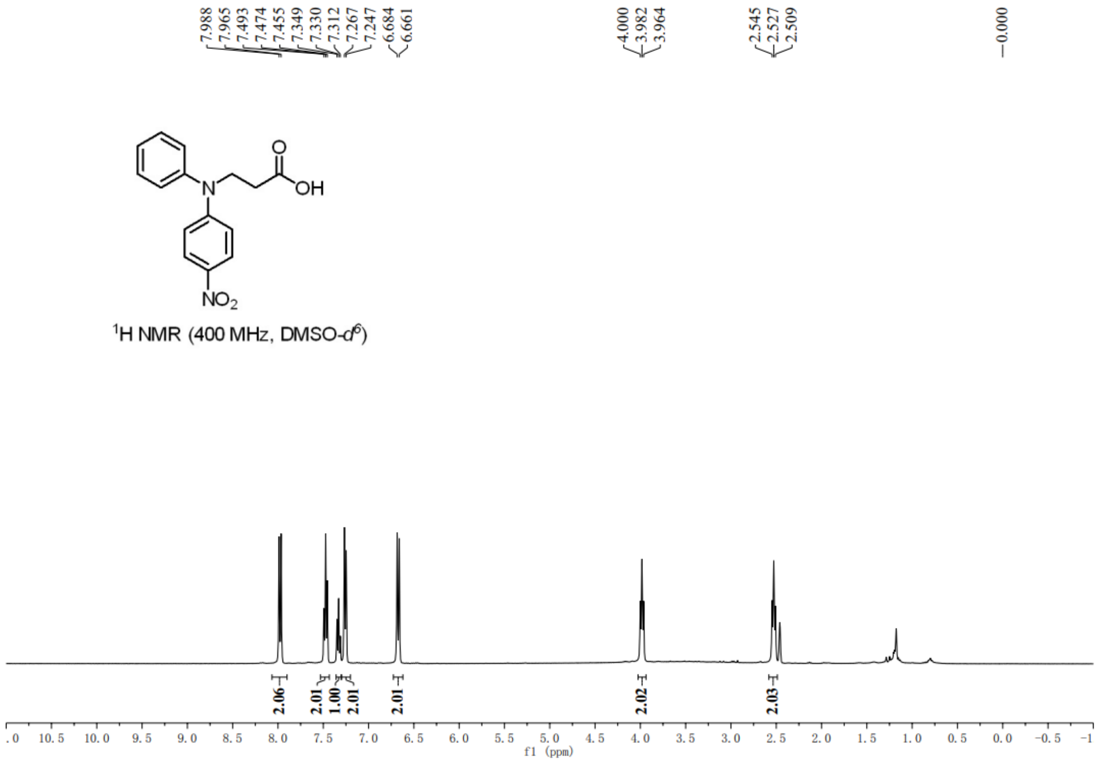

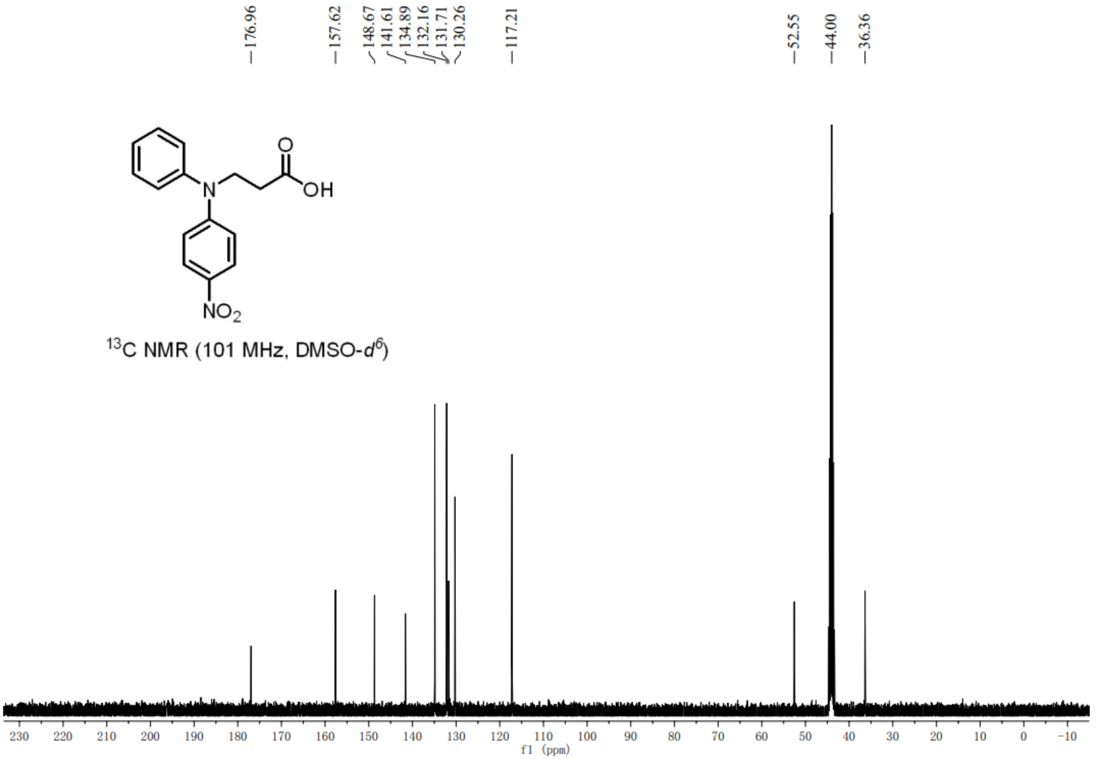

1-(4-nitrophenyl)pyrrolidine (3d-1)

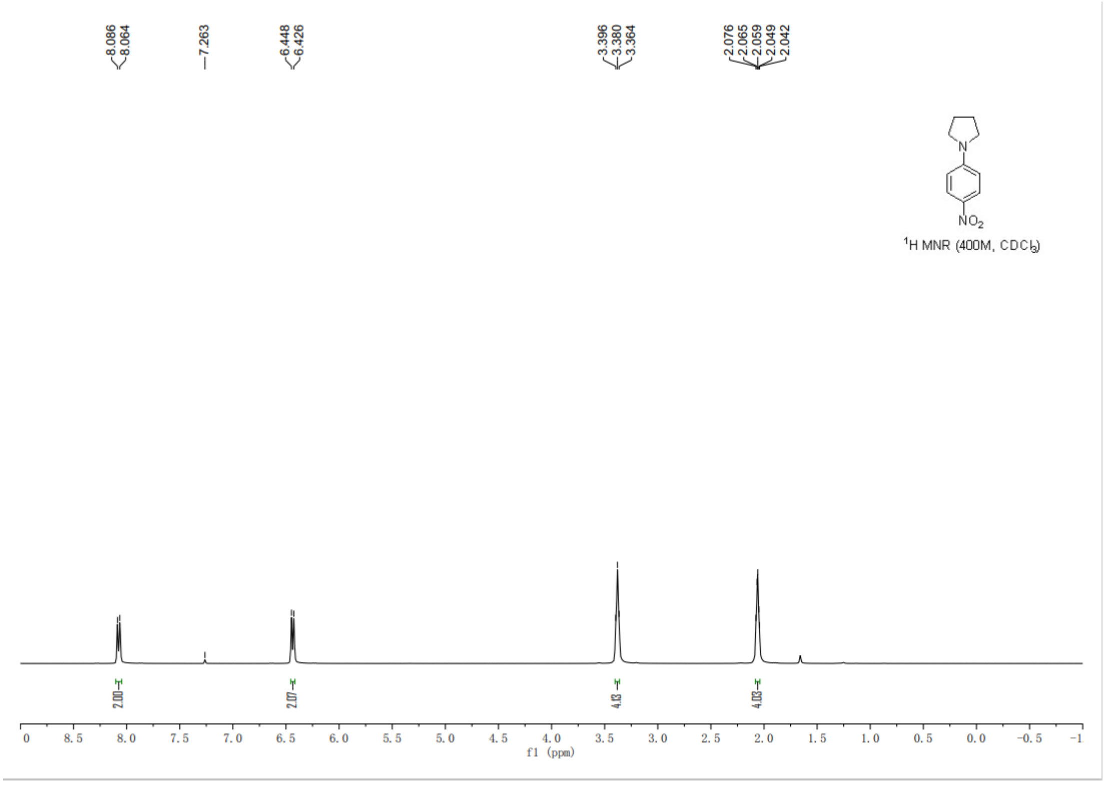

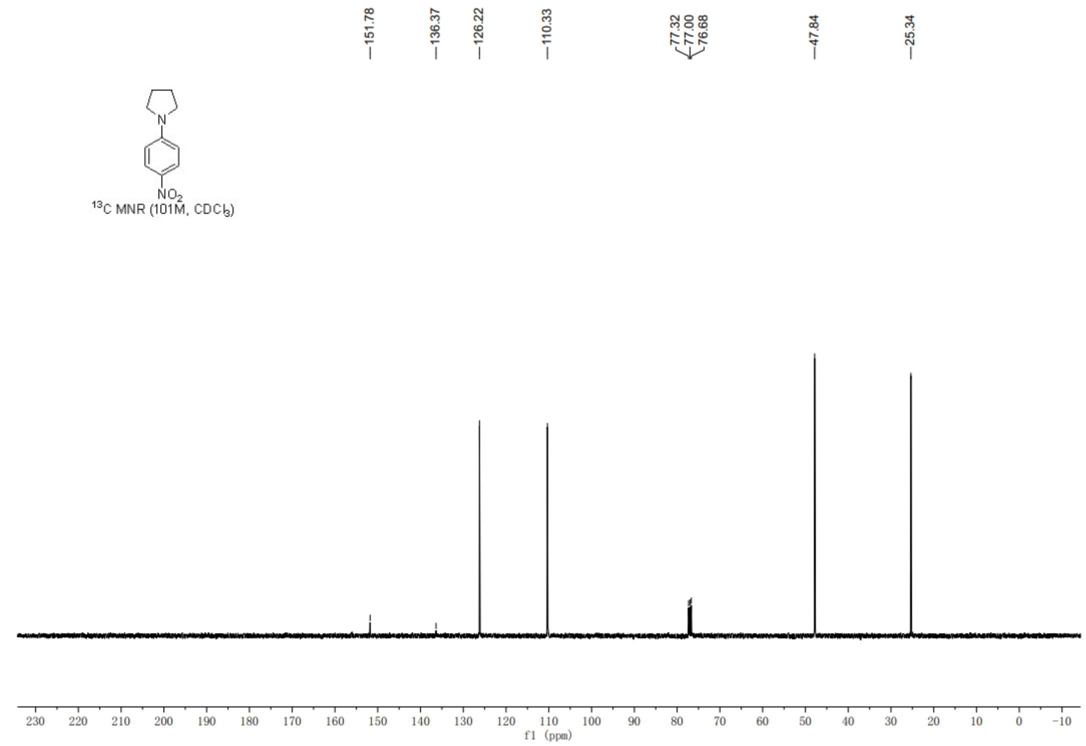

4-(4-nitrophenyl)morpholine (3d-2)

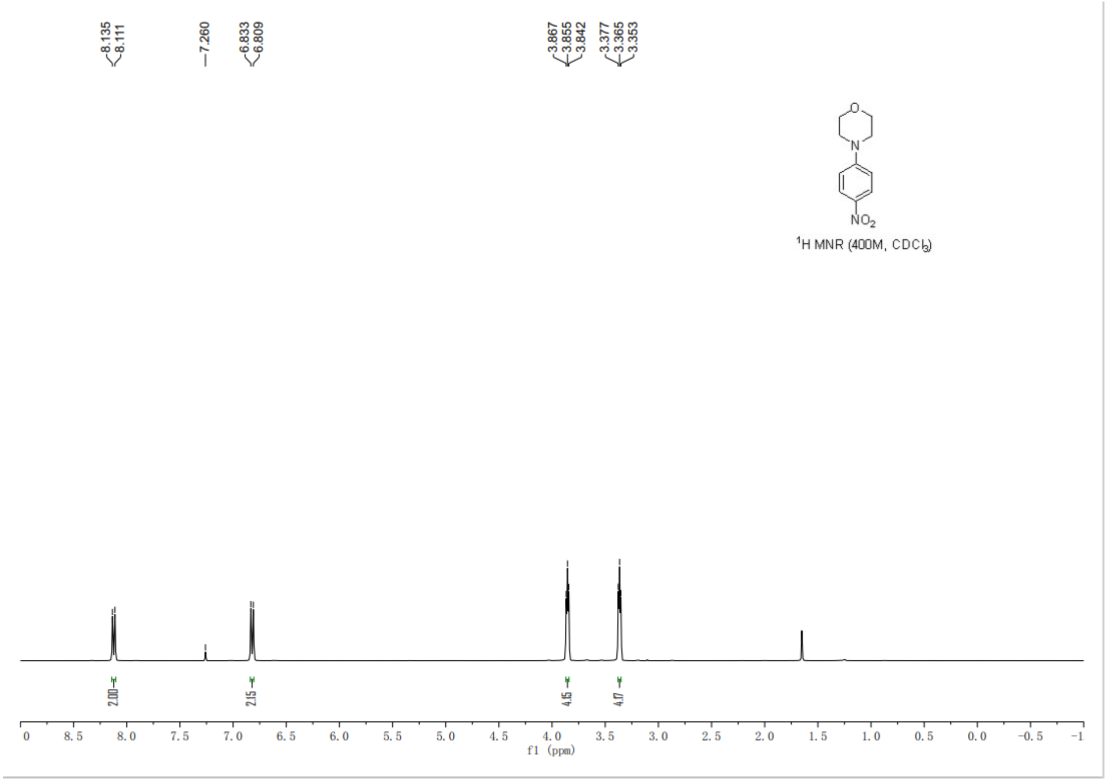

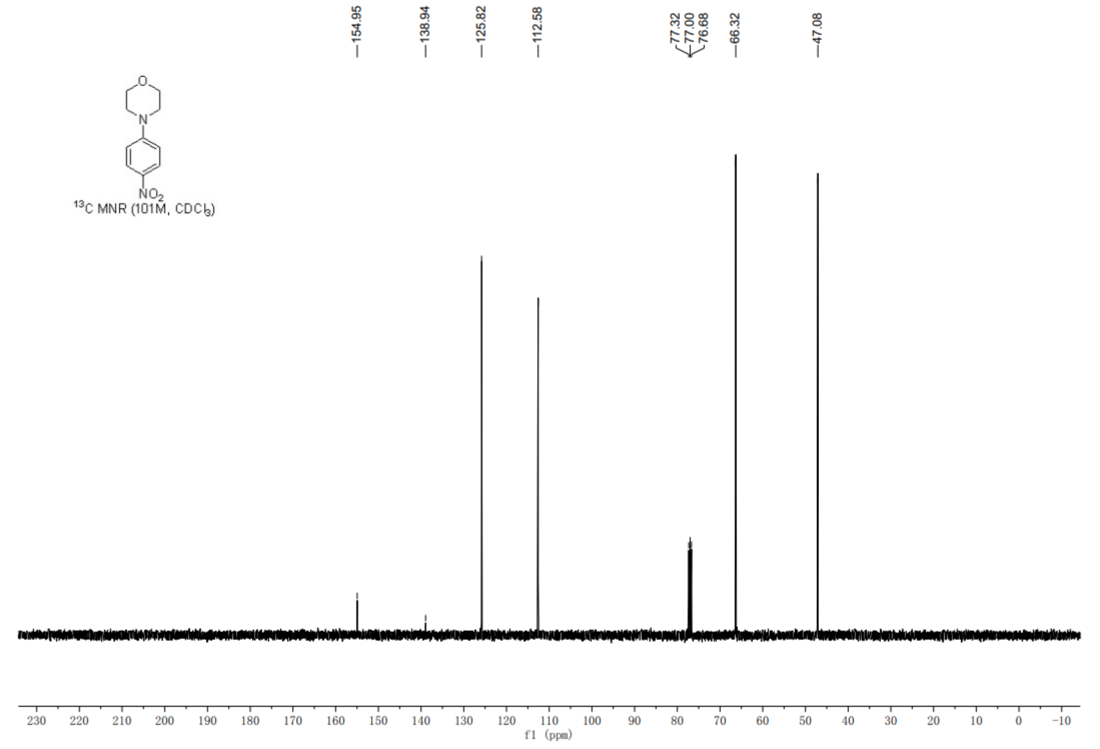

4-(4-nitrophenyl)thiomorpholine (3d-3)

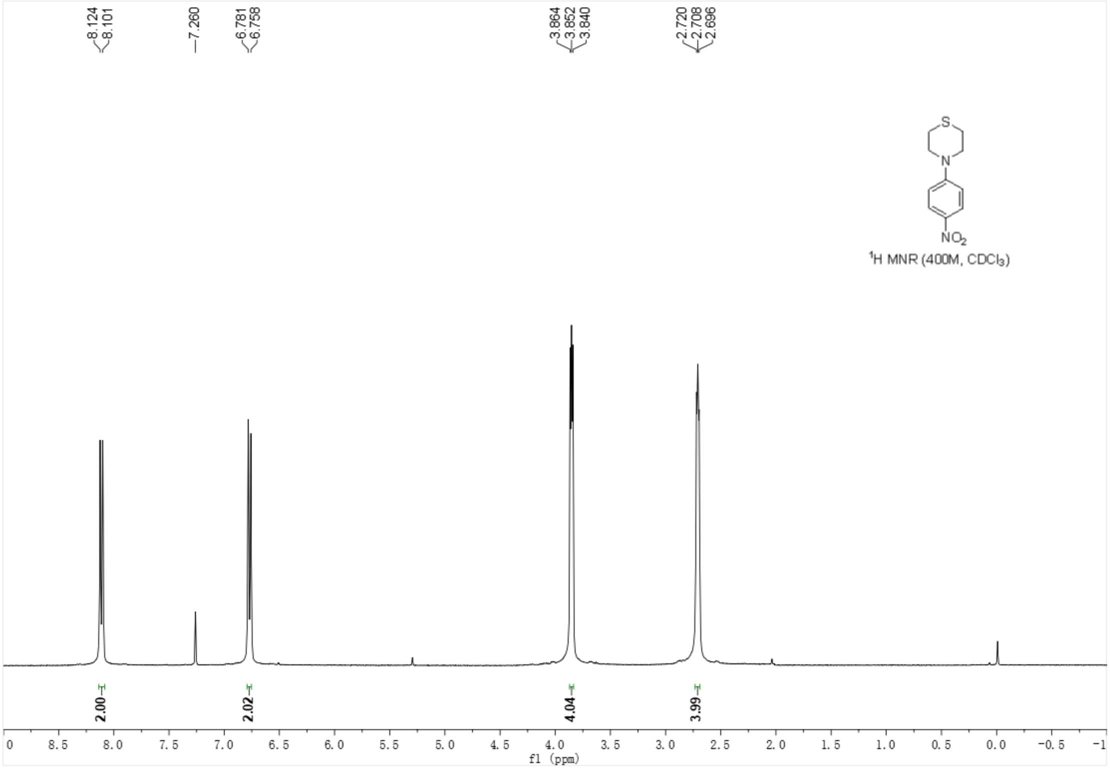

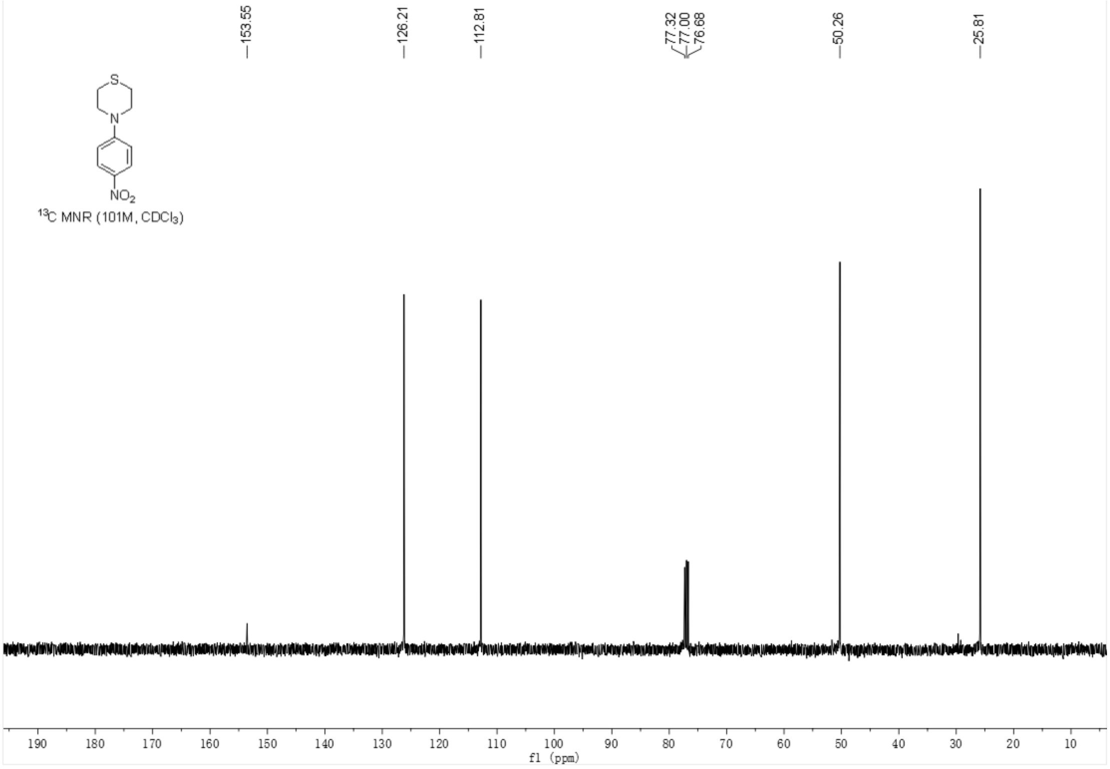

1-methyl-4-(4-nitrophenyl)piperazine (3d-4)

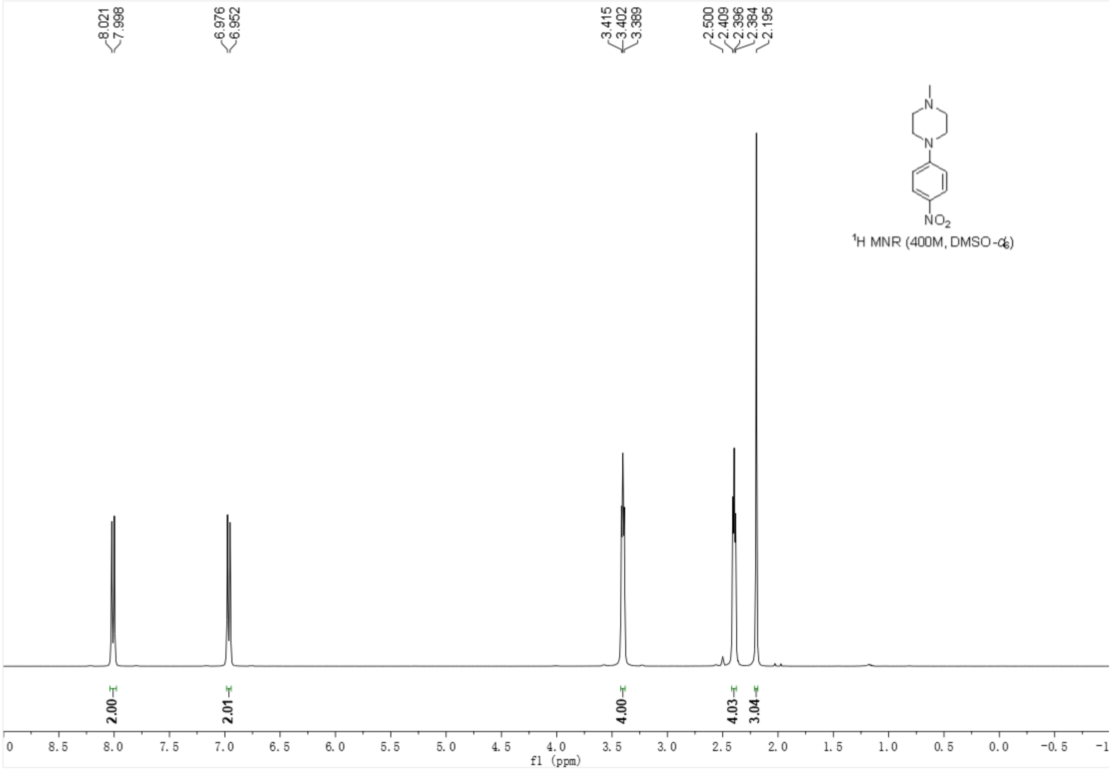

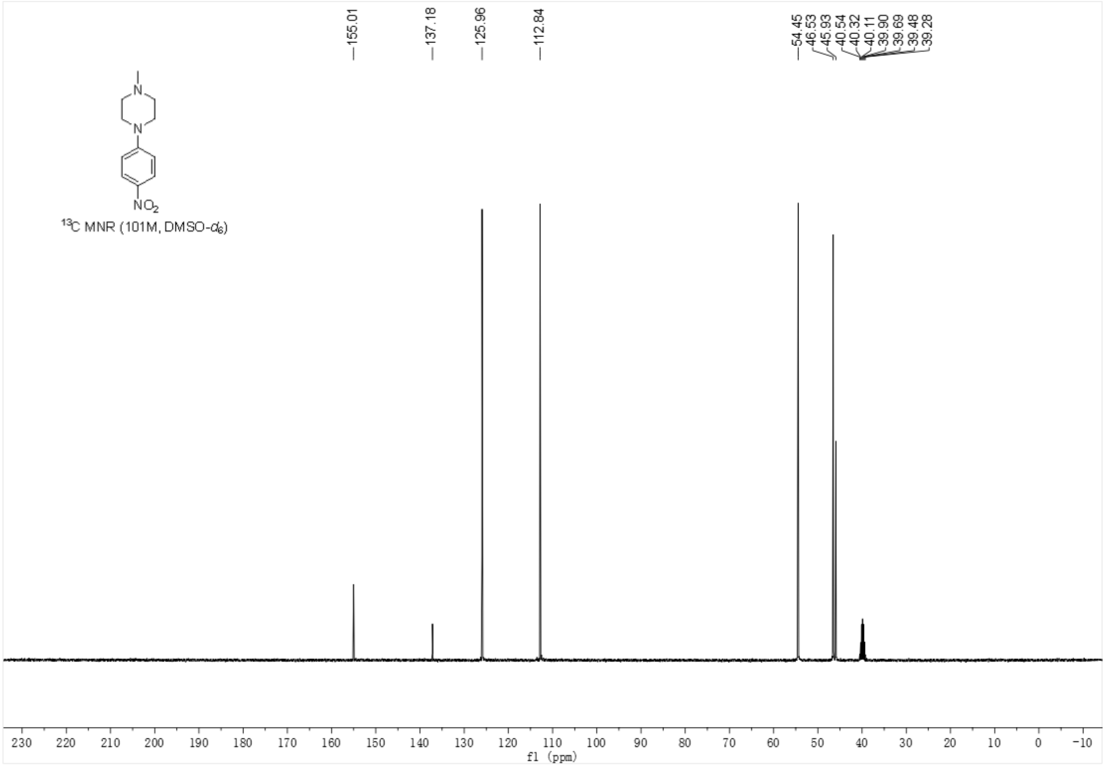

2-methyl-1-(4-nitrophenyl)pyrrolidine (3d-5)

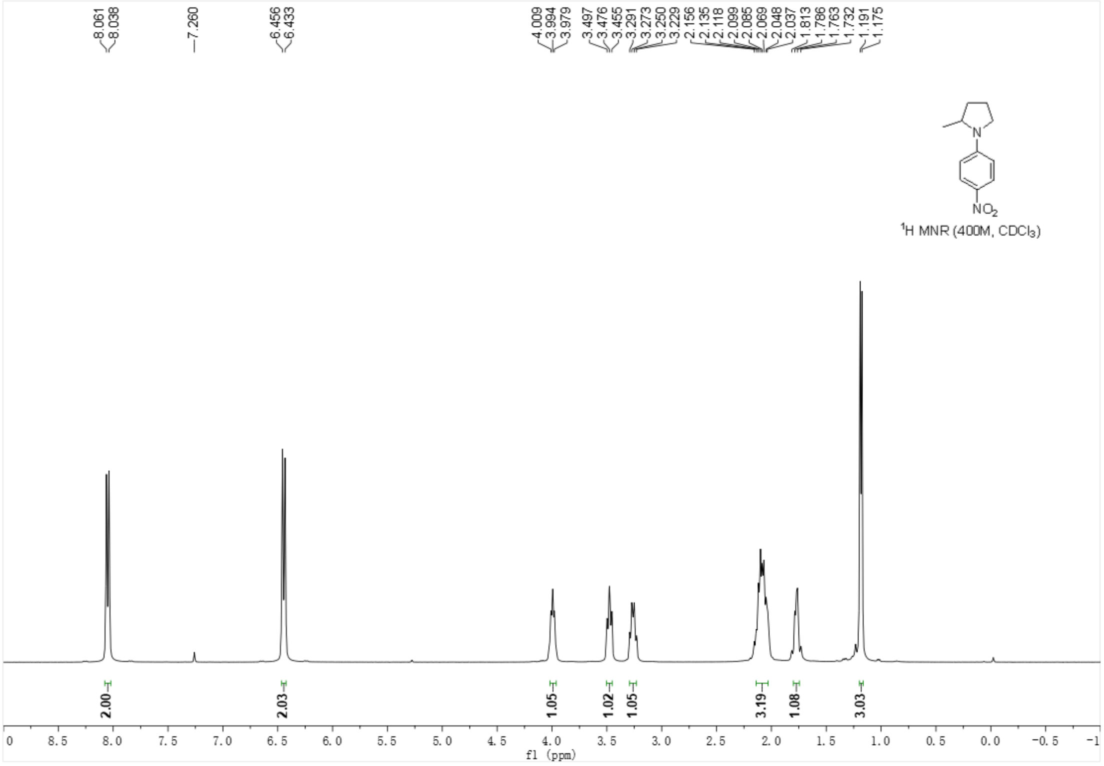

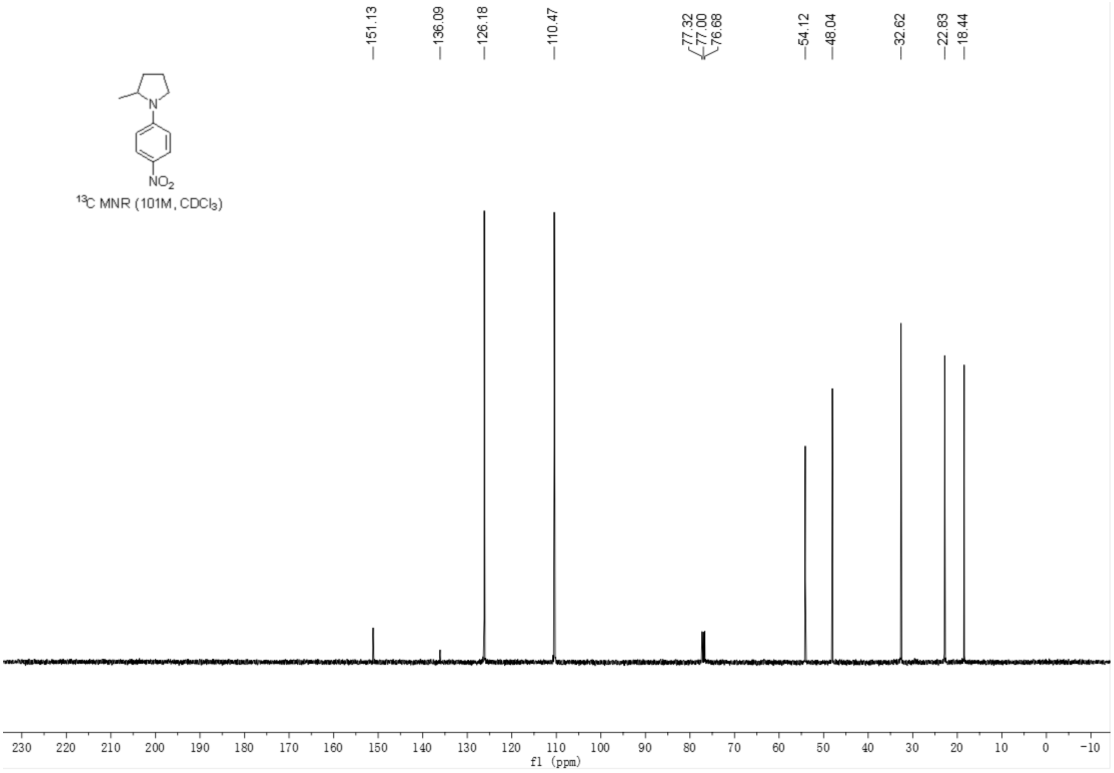

(4-nitrophenyl)proline (3d-6)

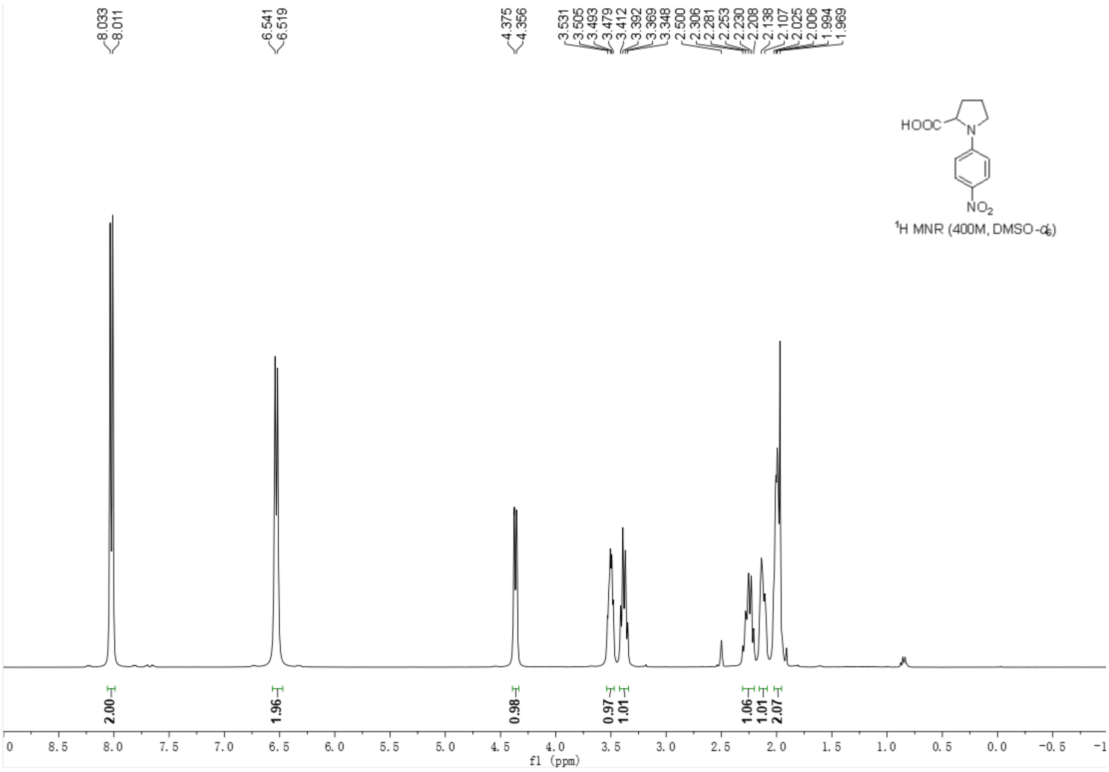

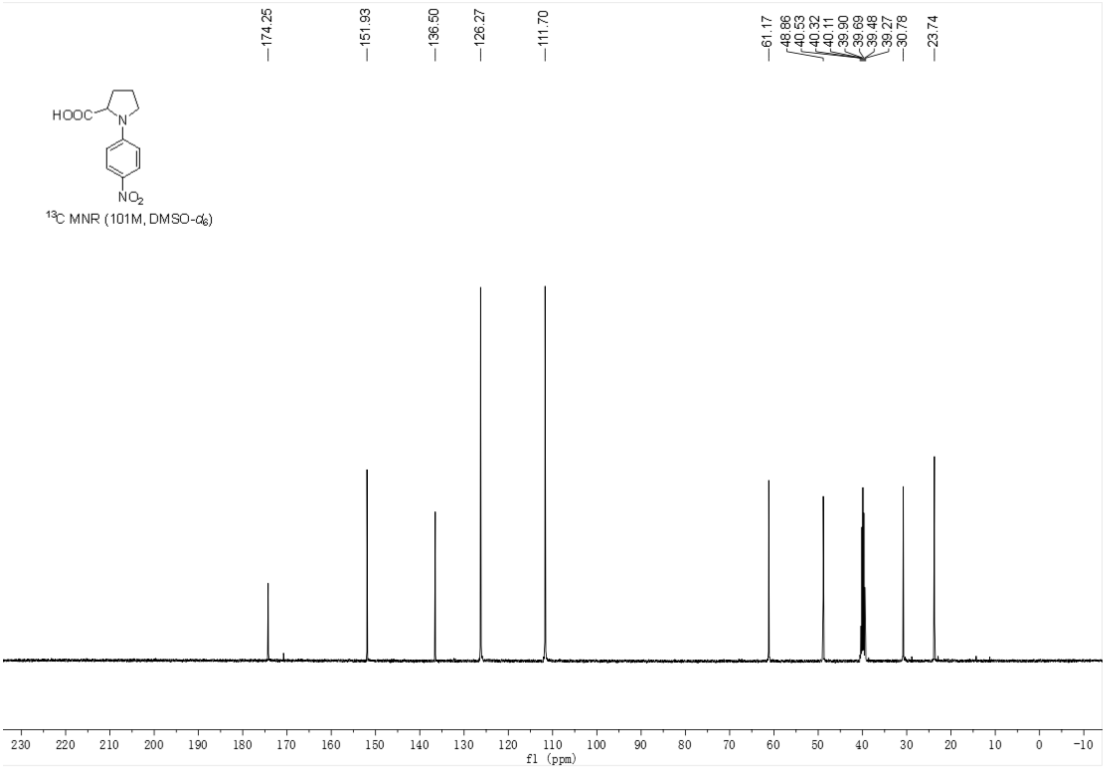

1-(4-nitrophenyl)pyrrolidine-2-carboxamide (3d-7)

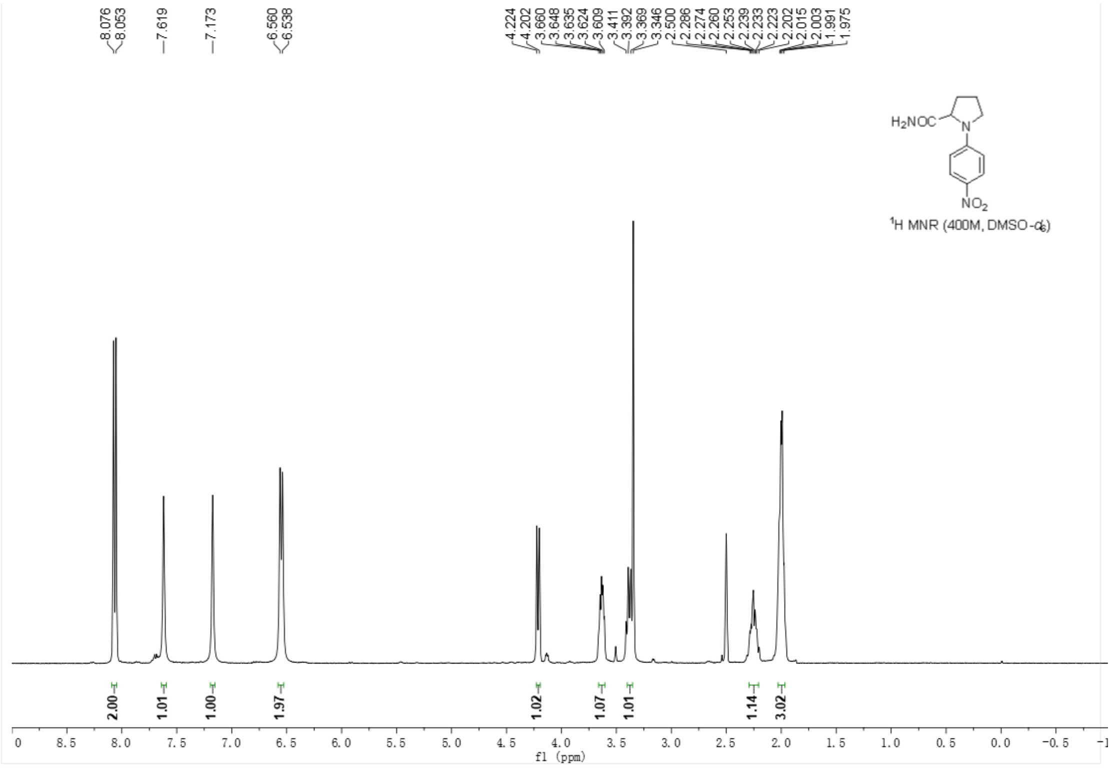

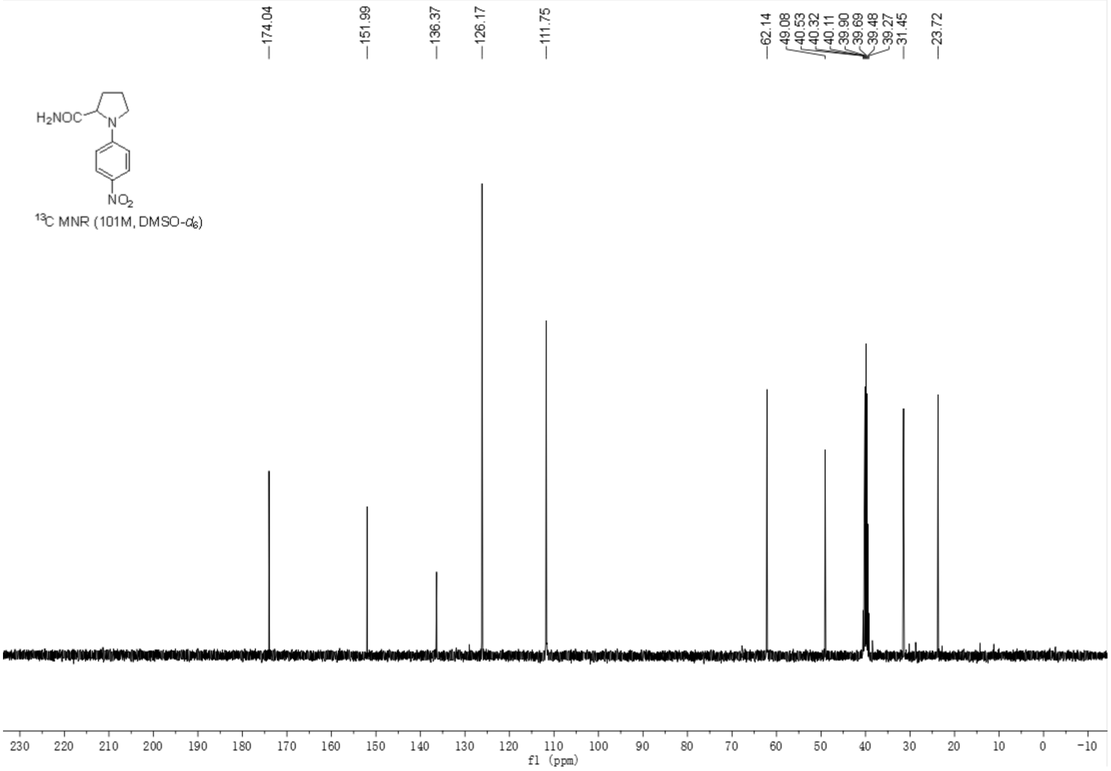

***N*,*N*-dimethyl-1-(4-nitrophenyl)pyrrolidin-3-amine (3d-8)**

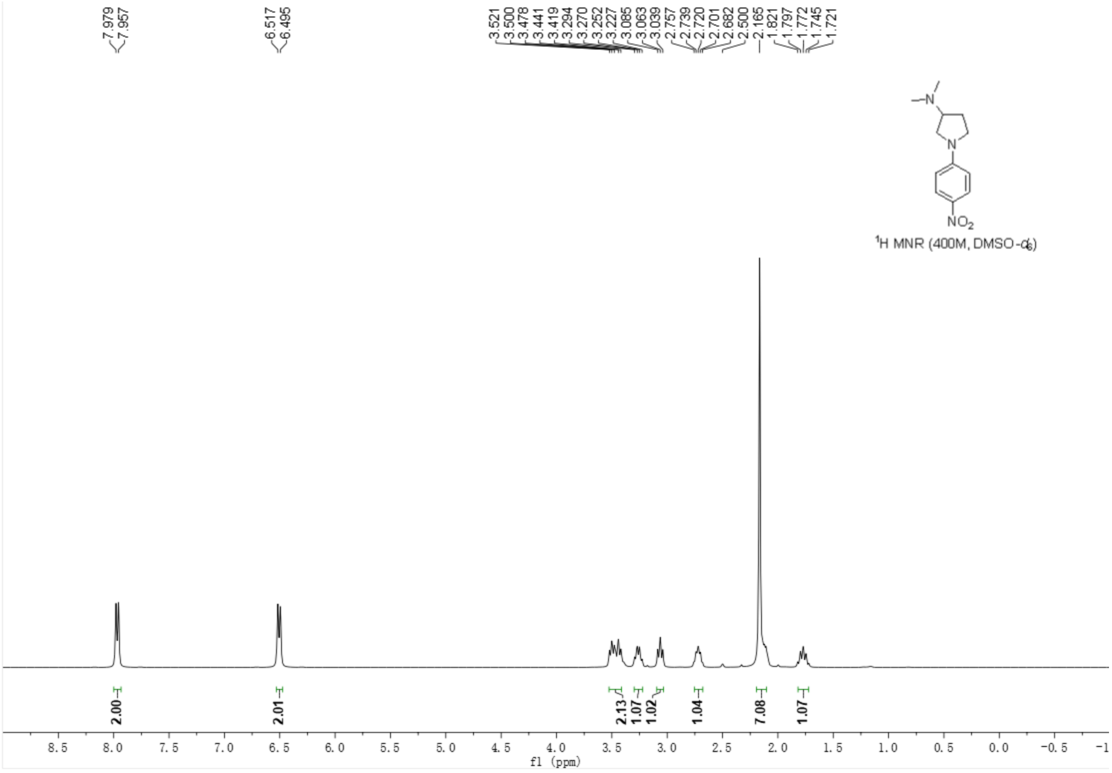

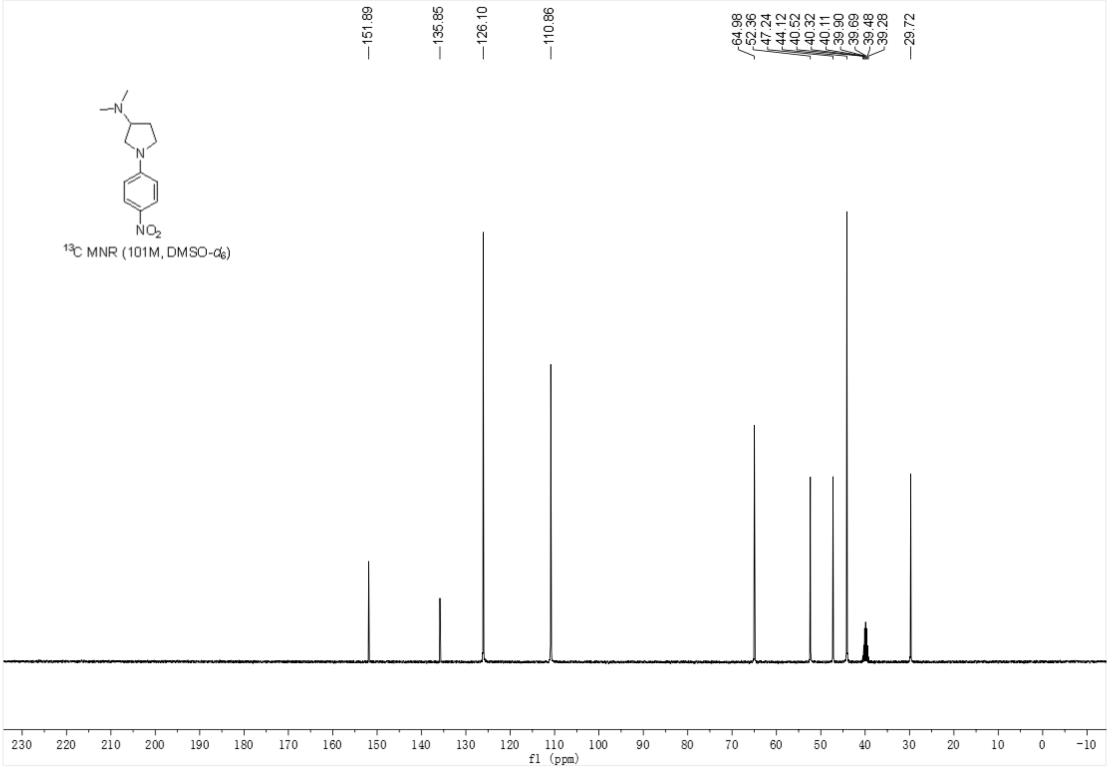

(1-(4-nitrophenyl)pyrrolidin-2-yl)methanol (3d-9)

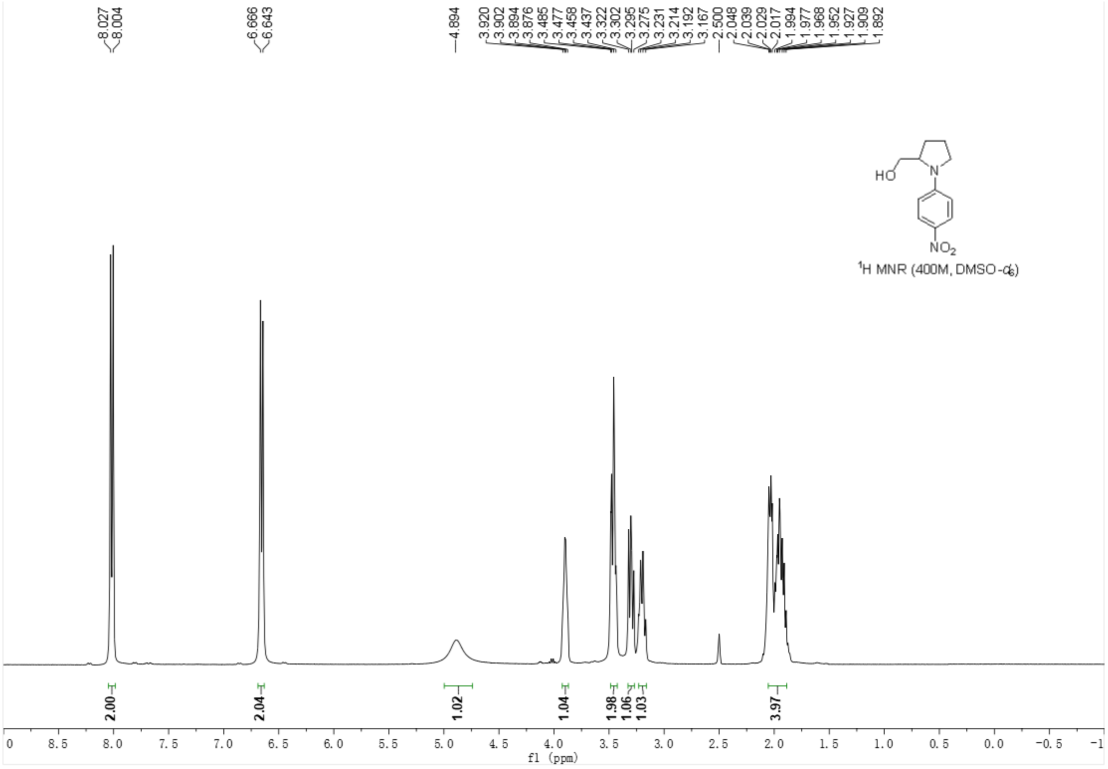

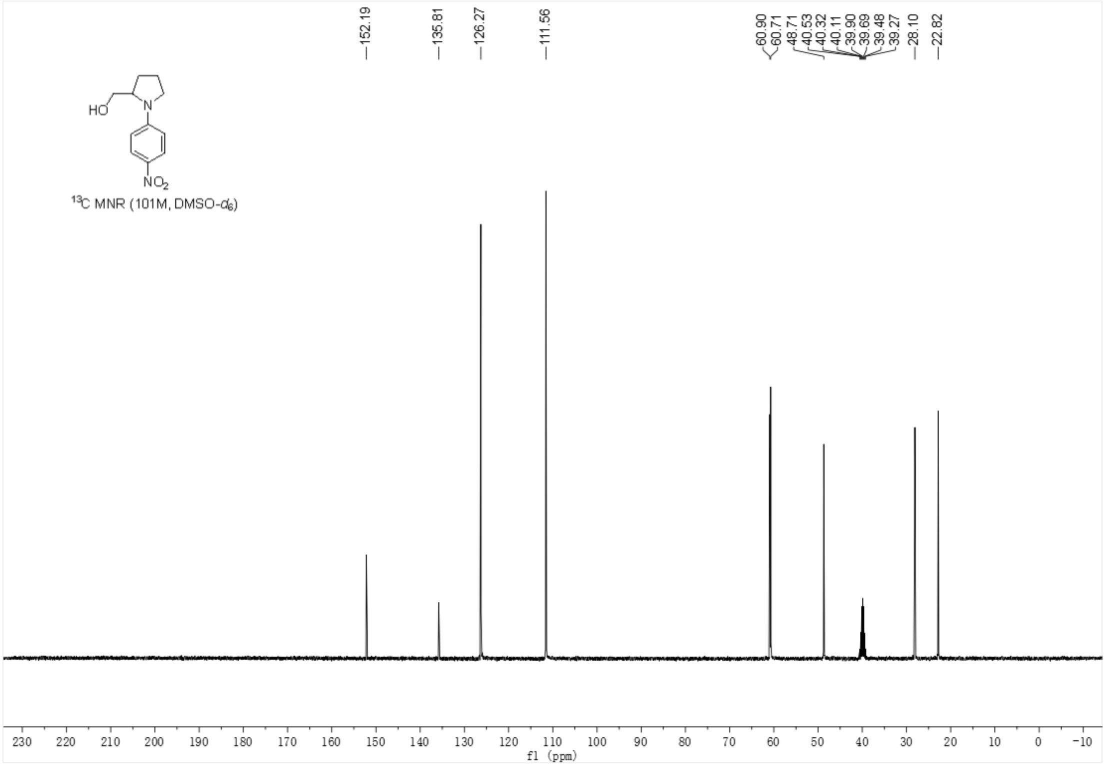

2-(methoxymethyl)-1-(4-nitrophenyl)pyrrolidine (3d-10)

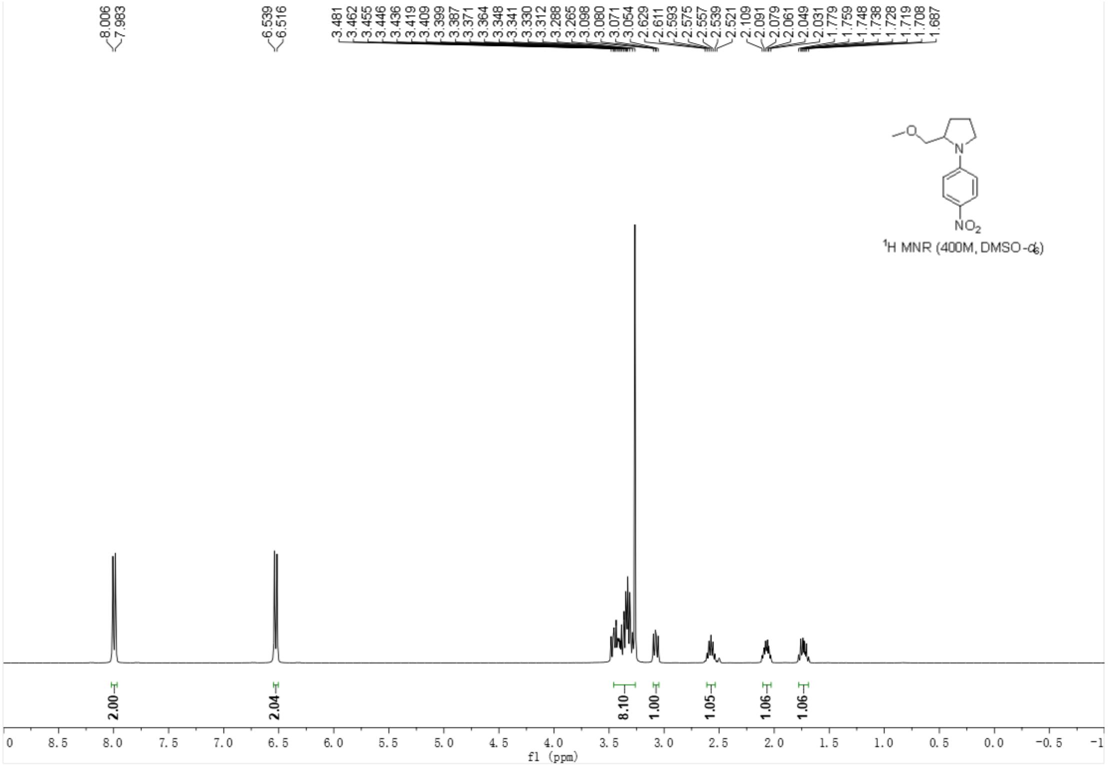

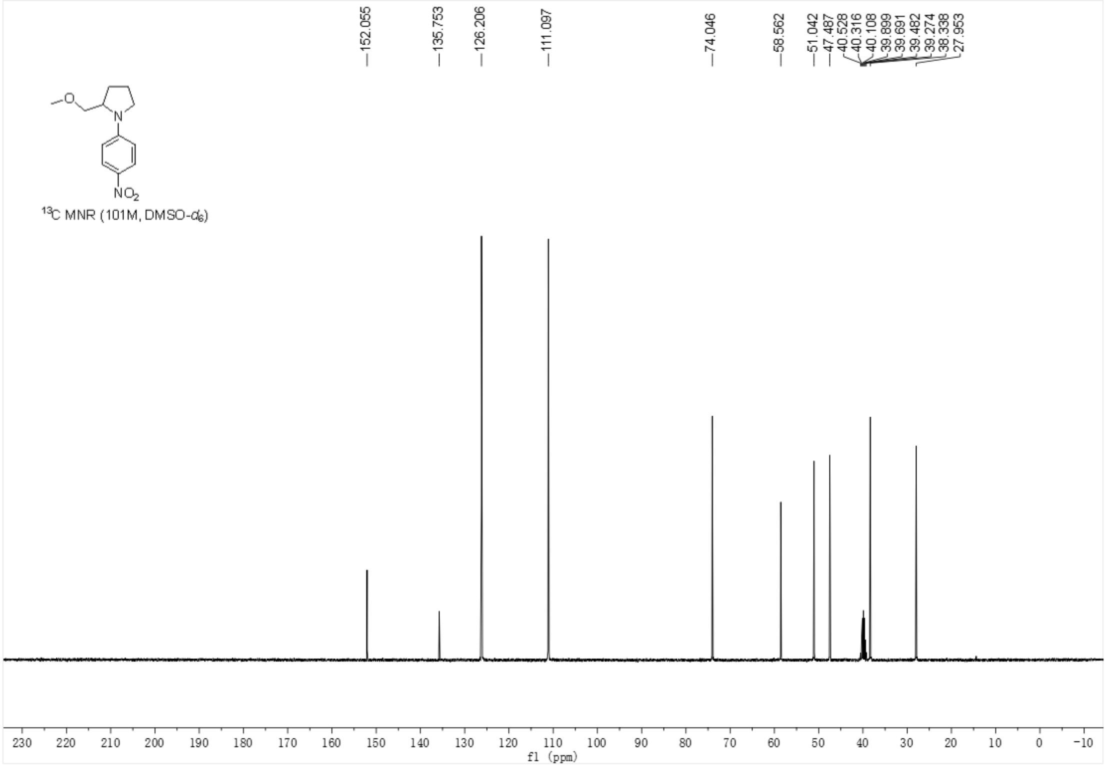

3-(1-(4-nitrophenyl)pyrrolidin-2-yl)pyridine (3d-11)

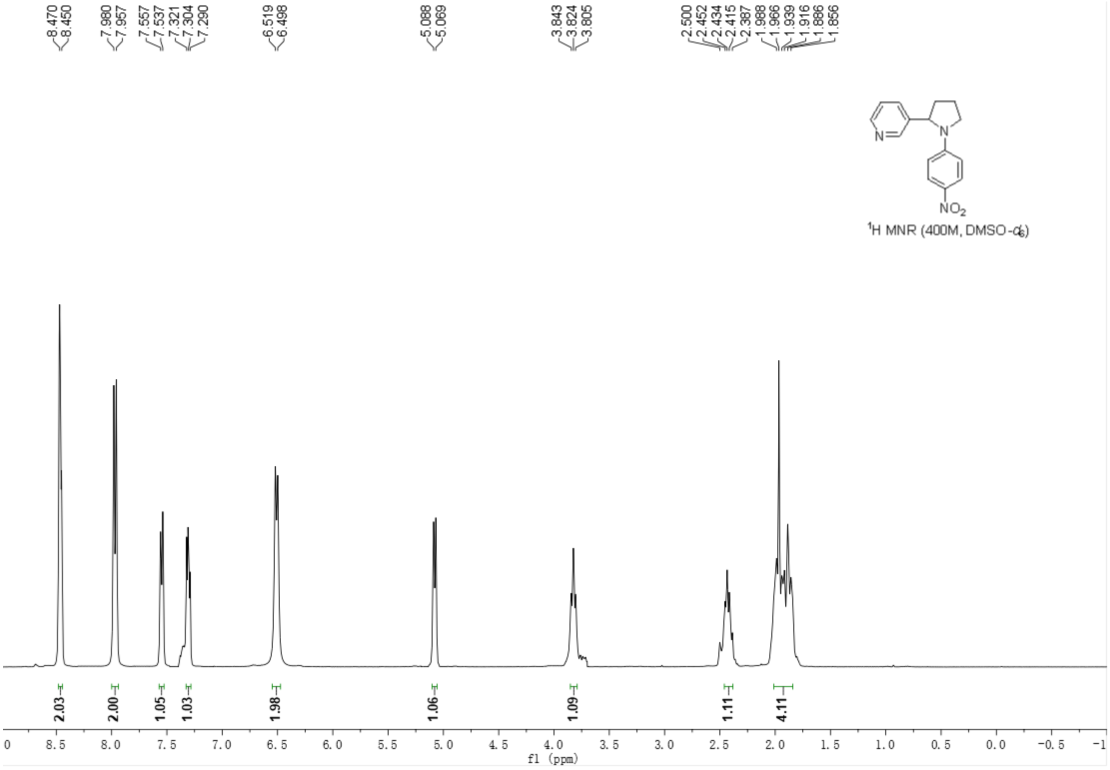

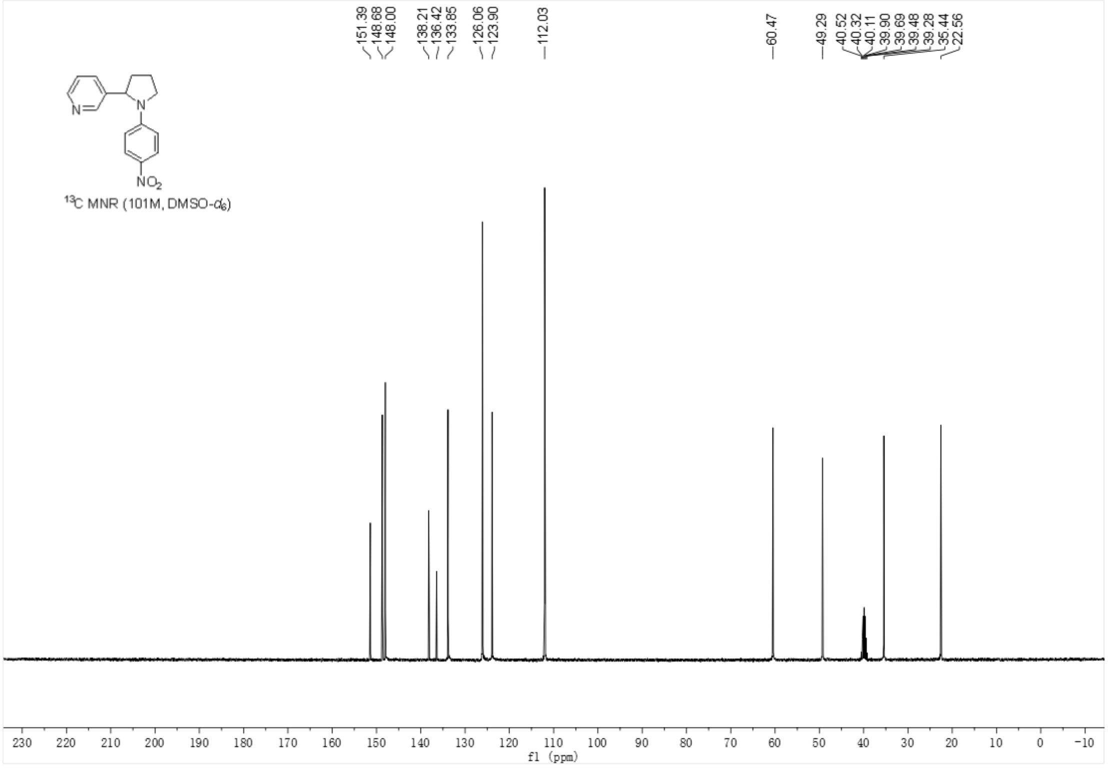

***N*-cyclohexyl-4-nitroaniline (3d-12)**

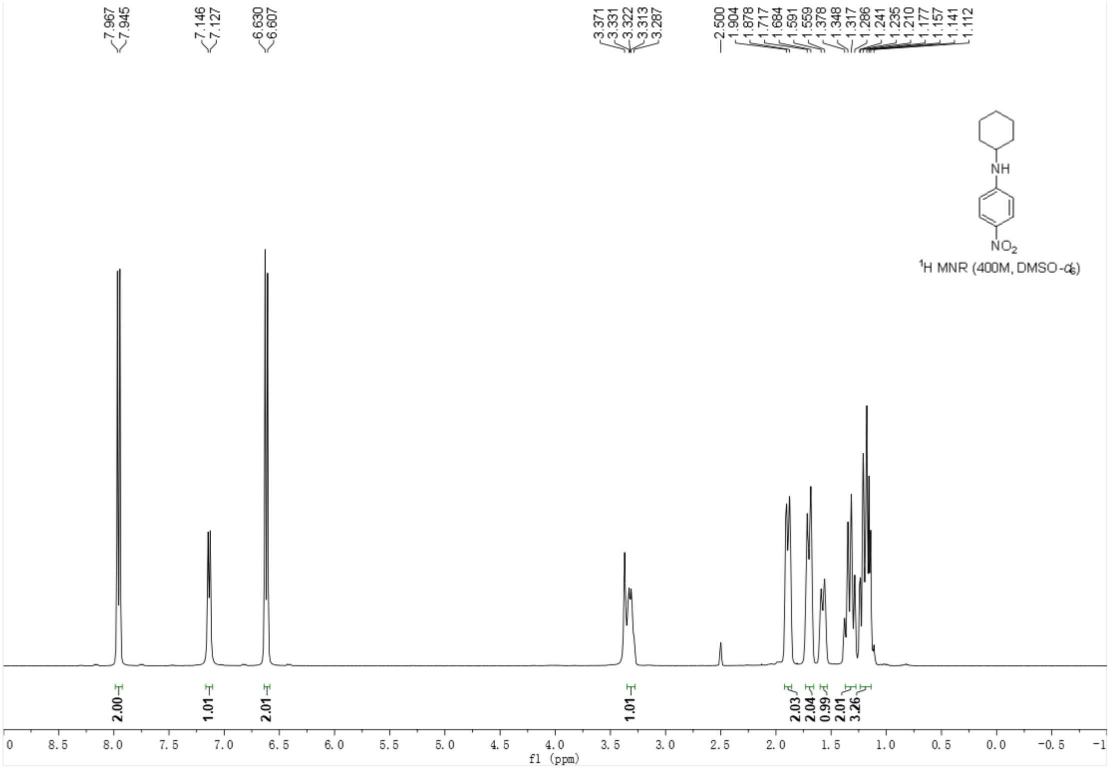

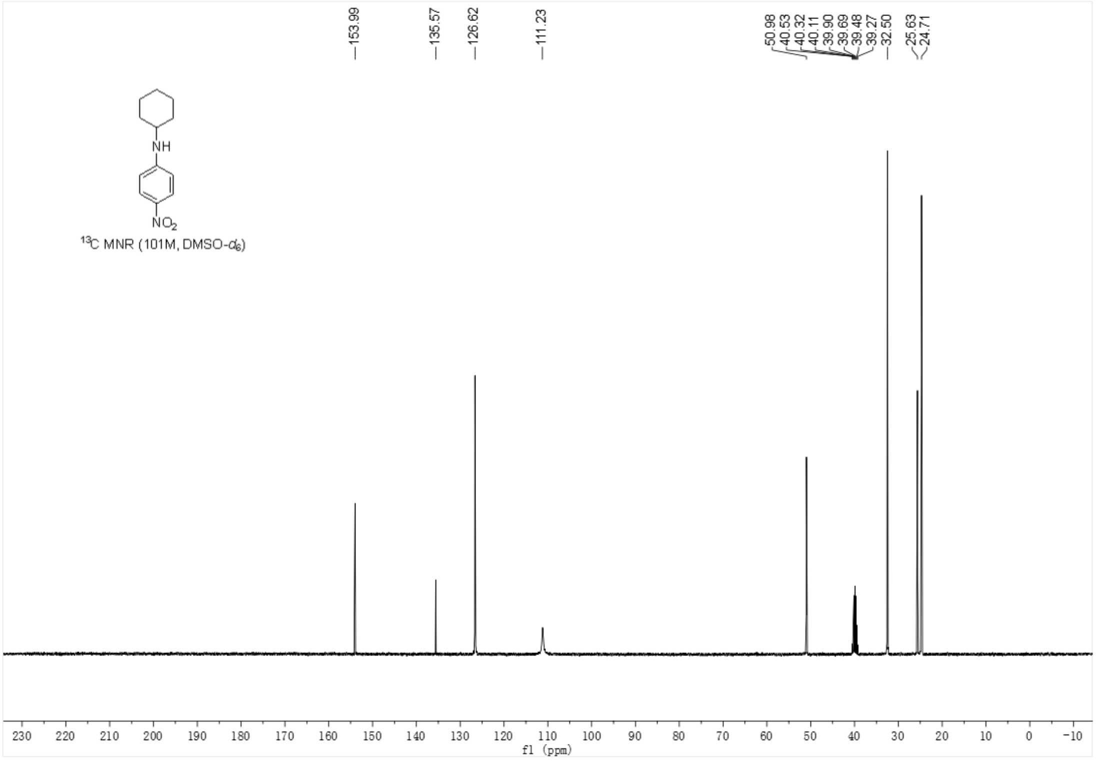

N-benzyl-N-methyl-4-nitroaniline (3d-13)

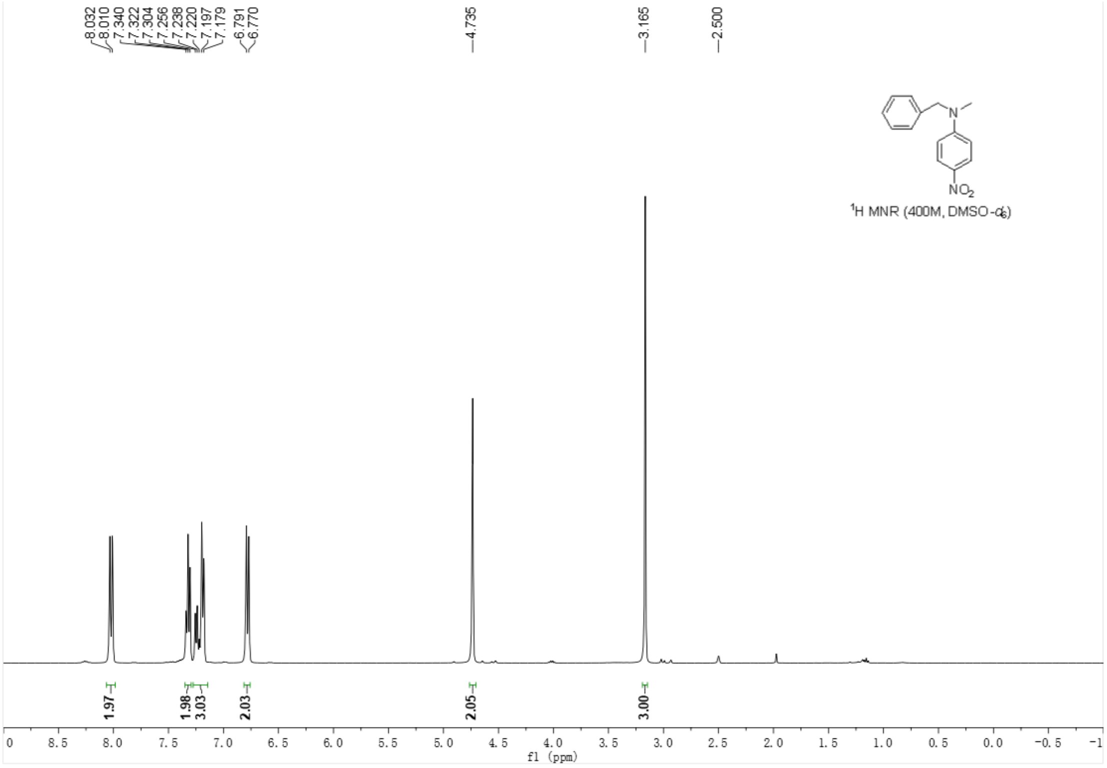

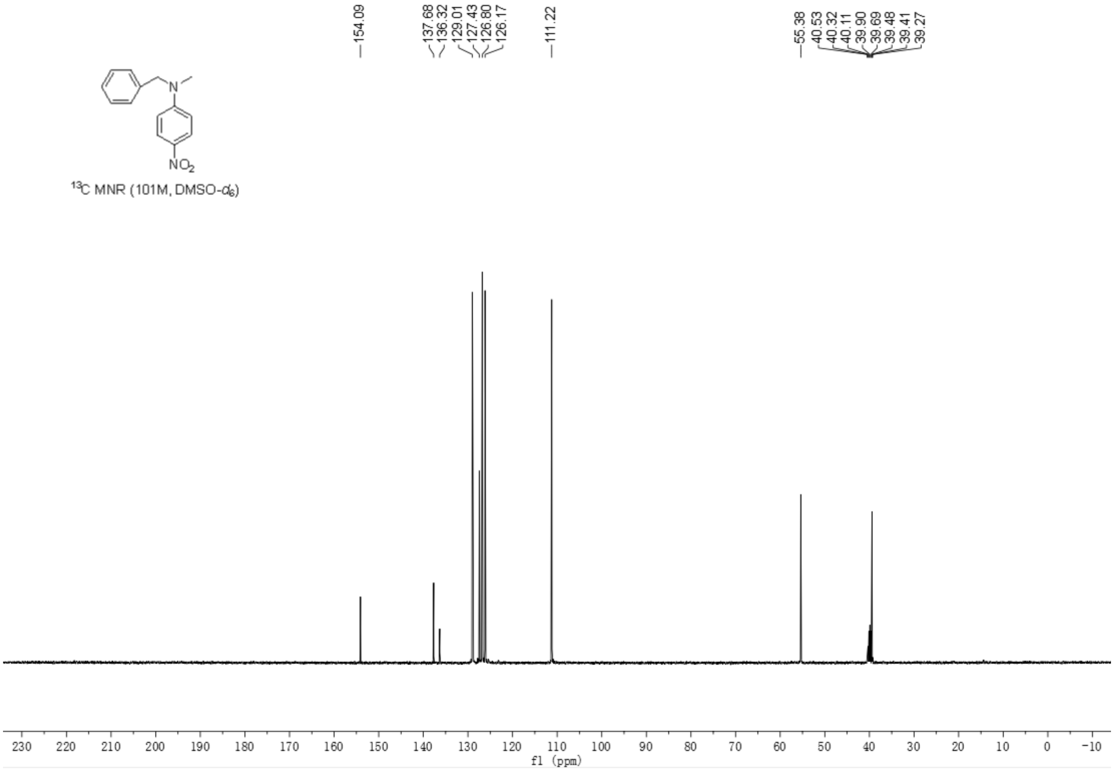

1-(3-chloro-4-nitrophenyl)-1,2,3,4-tetrahydroquinoline (4a-1)

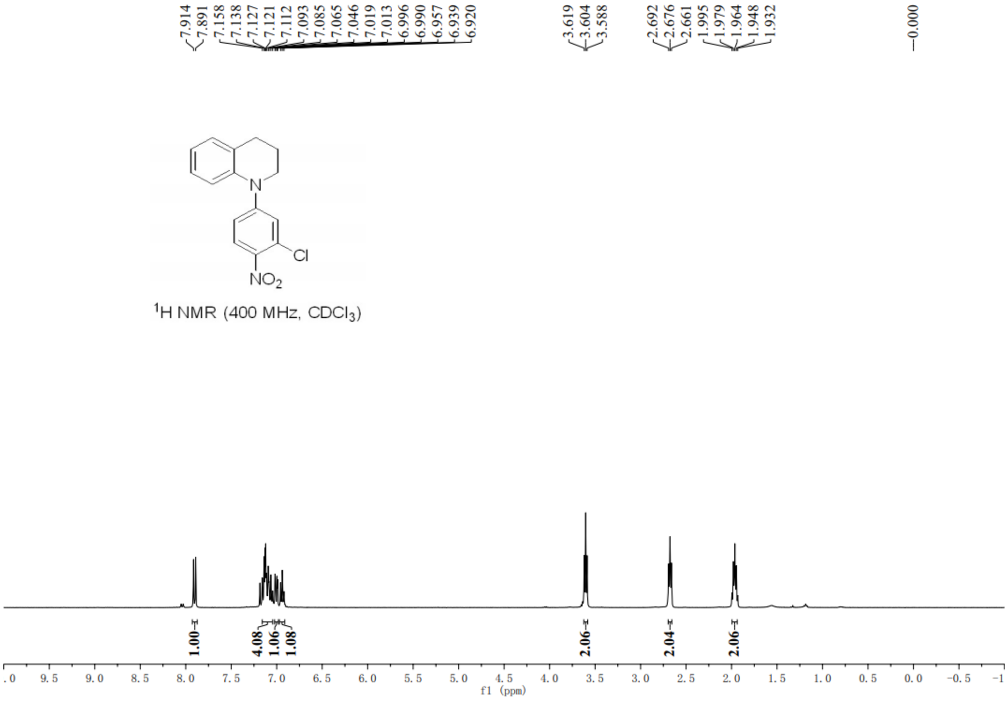

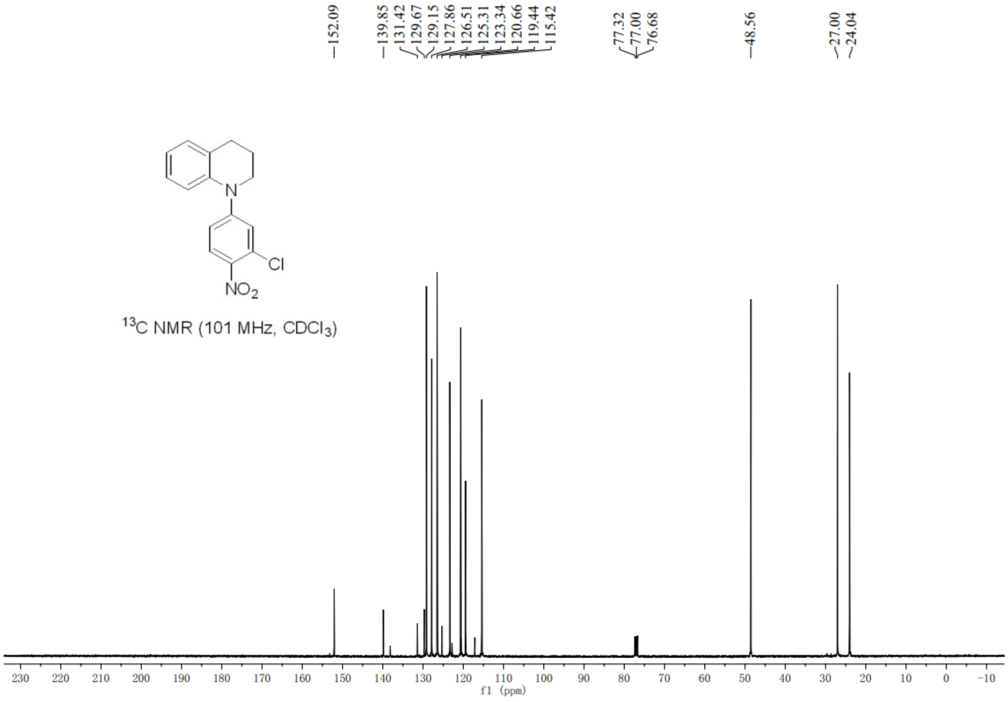

1-(3-chloro-4-nitrophenyl)indoline (4b-1)

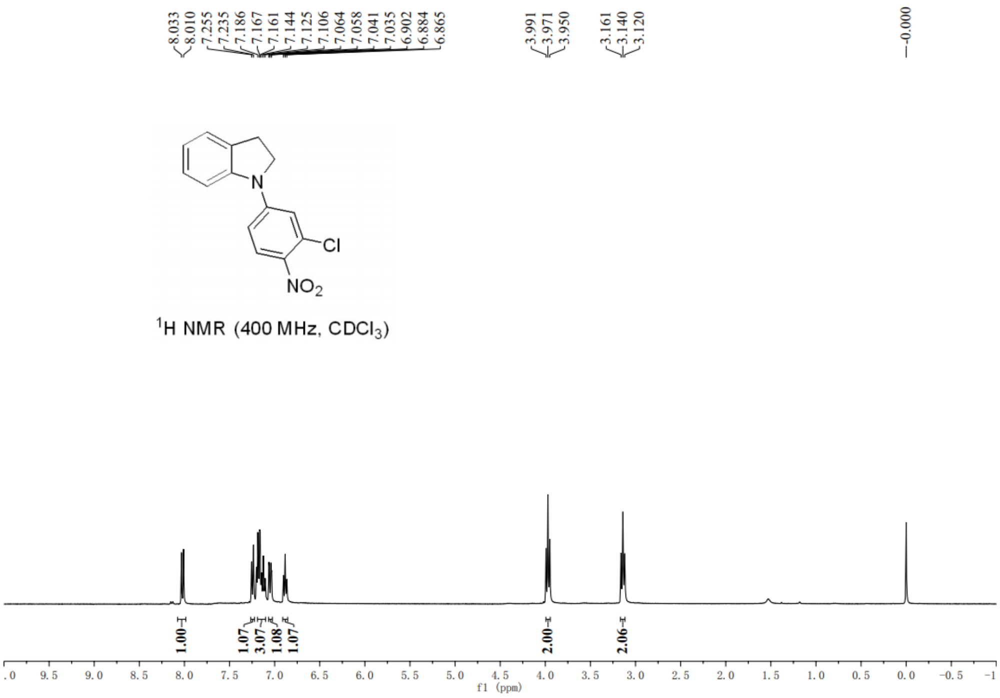

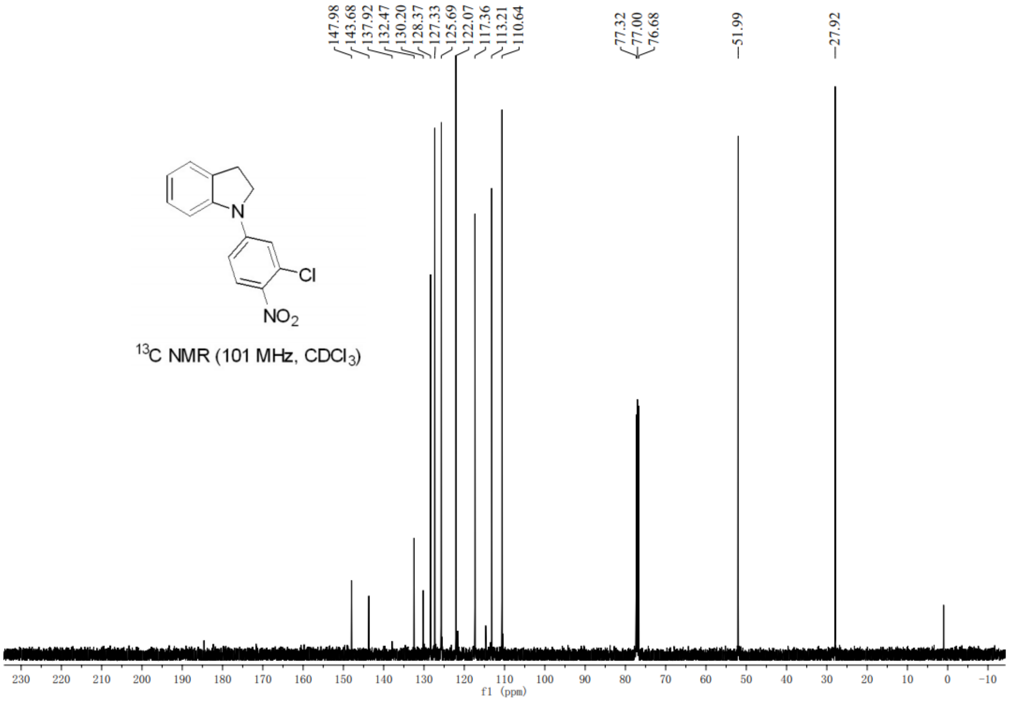

3-chloro-*N*-methyl-4-nitro-*N*-phenylaniline (4c-1)

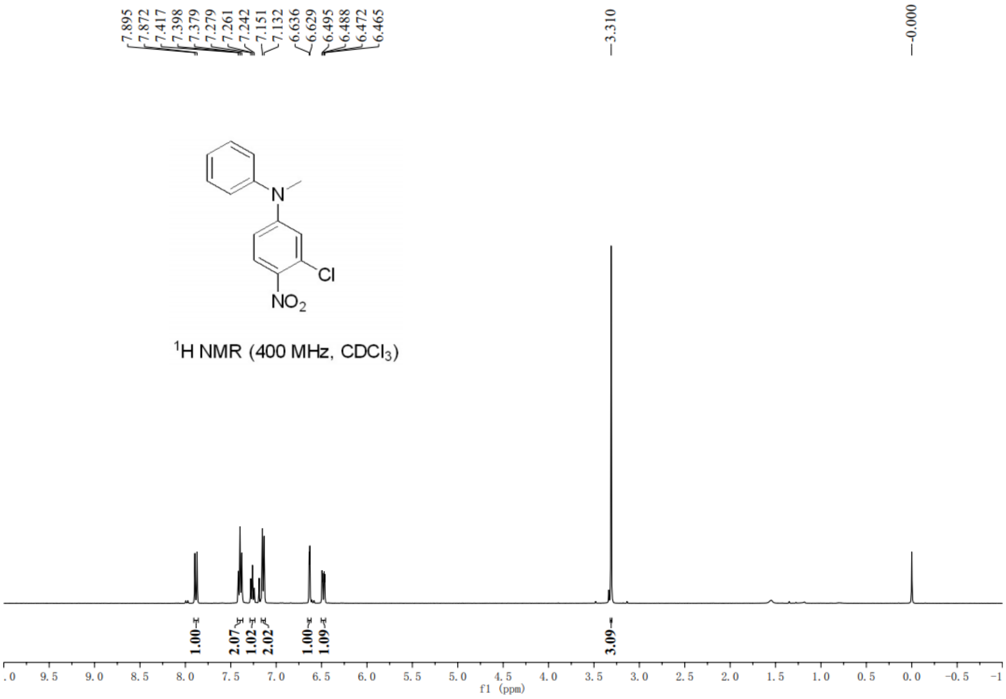

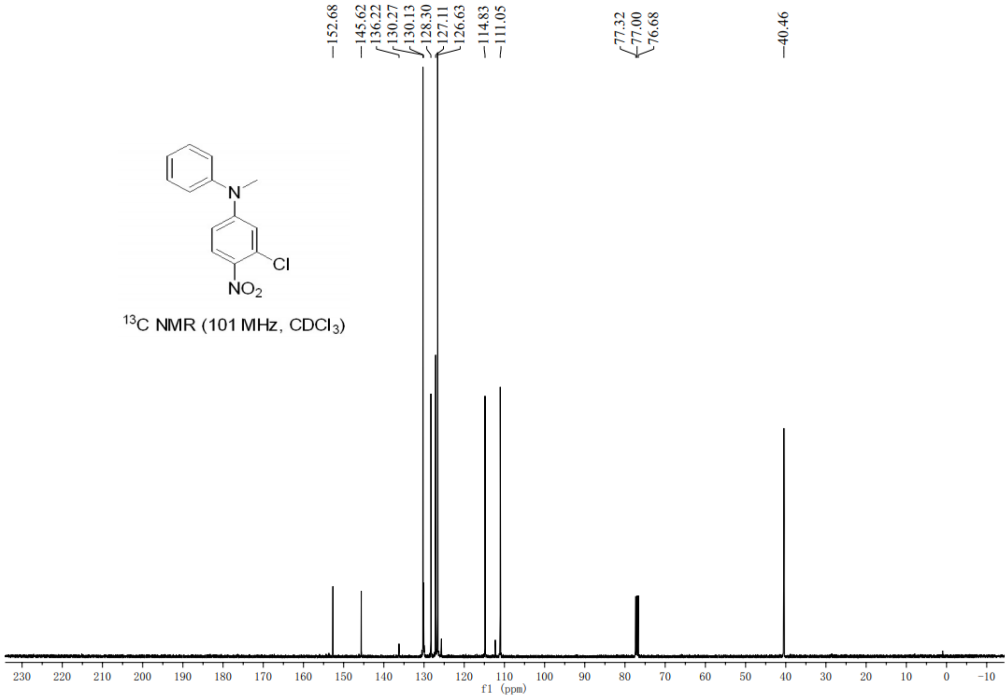

5-(3,4-dihydroquinolin-1(2*H*)-yl)-2-nitrobenzonitrile (4a-2)

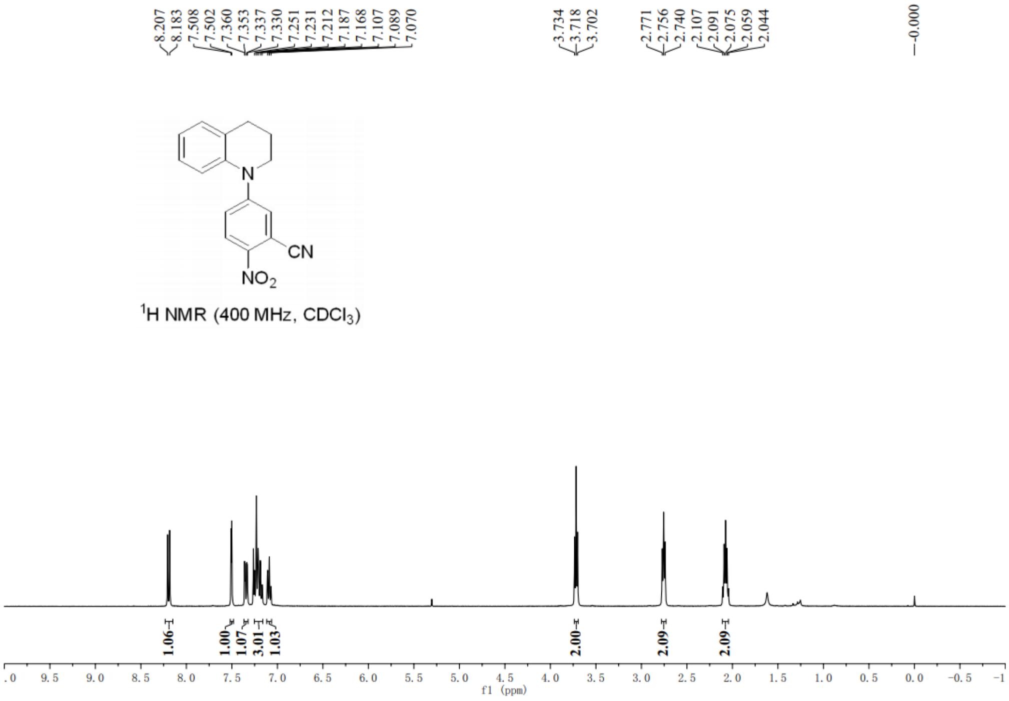

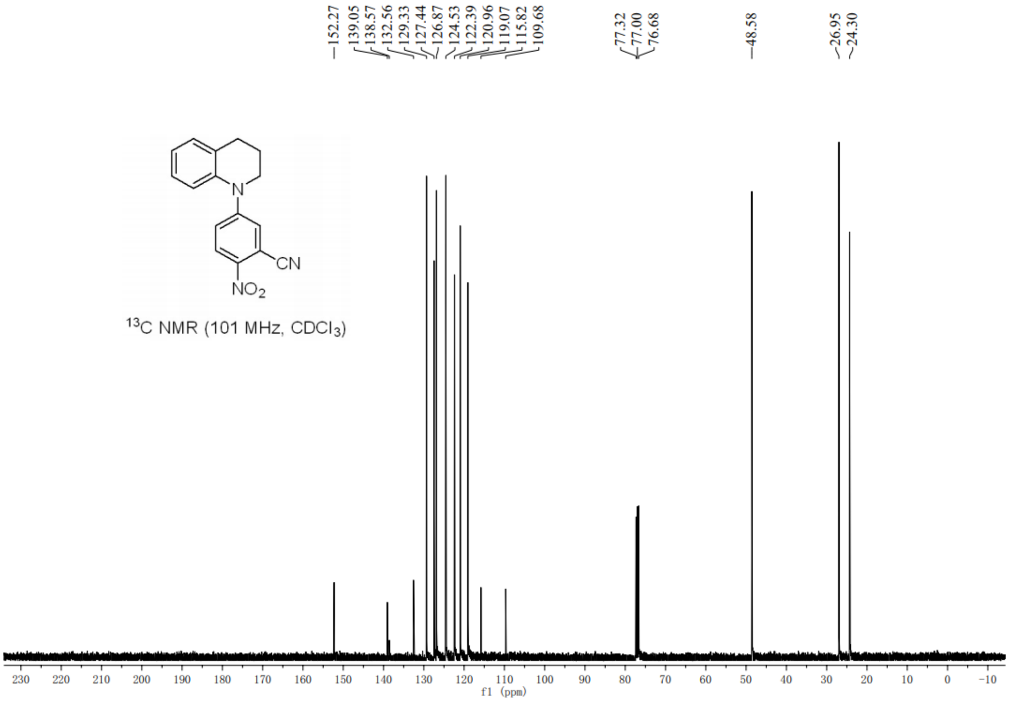

5-(indolin-1-yl)-2-nitrobenzonitrile (4b-2)

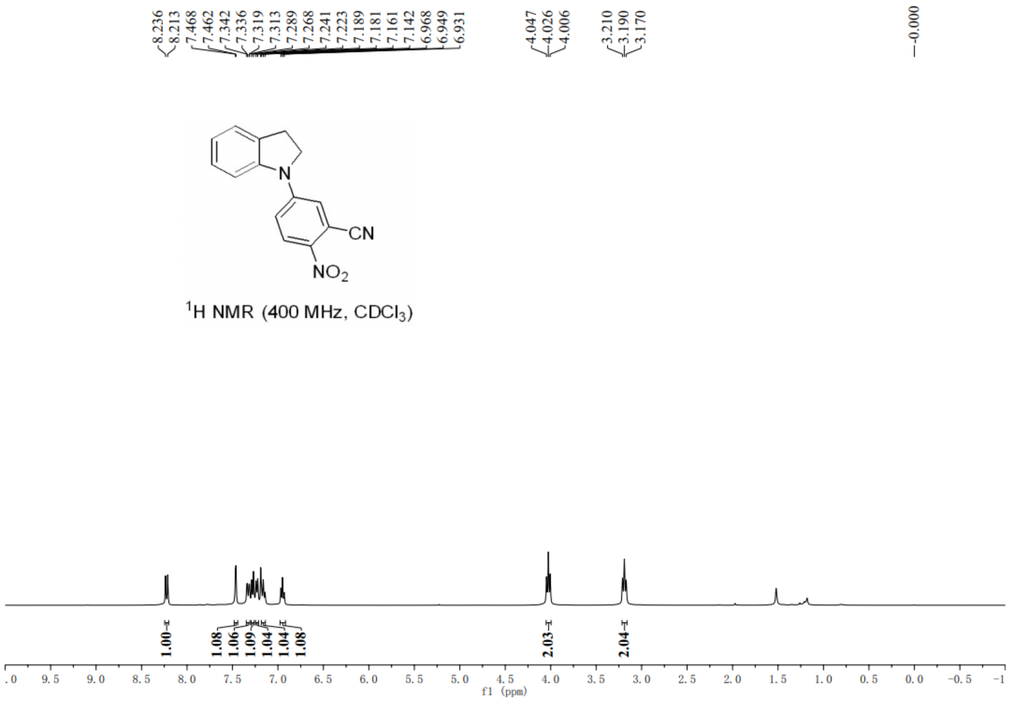

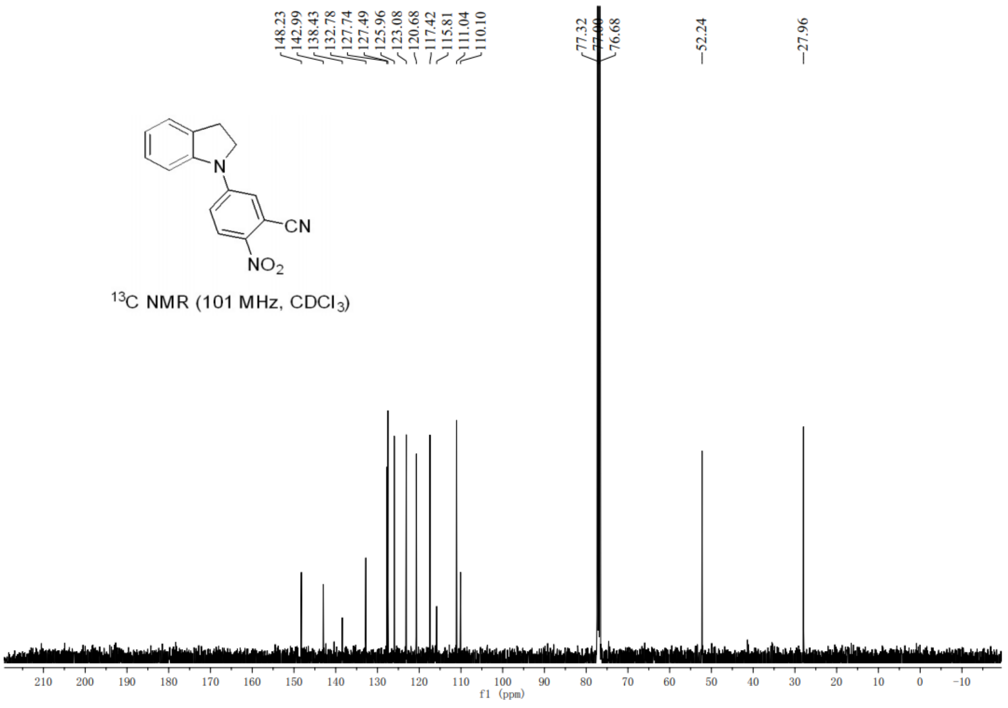

5-(methyl(phenyl)amino)-2-nitrobenzonitrile (4c-2)

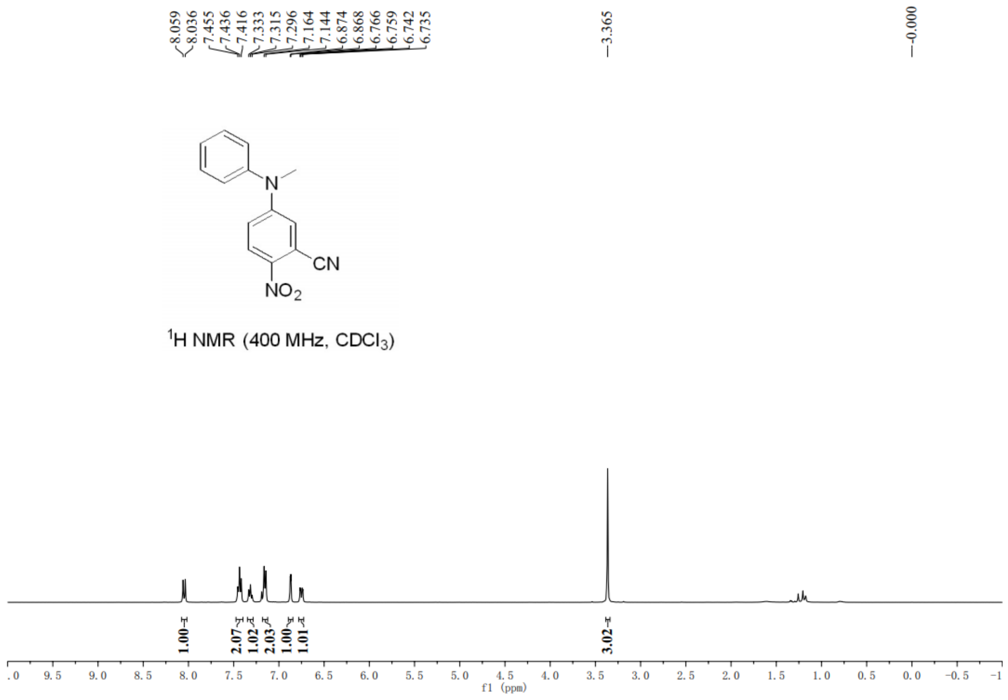

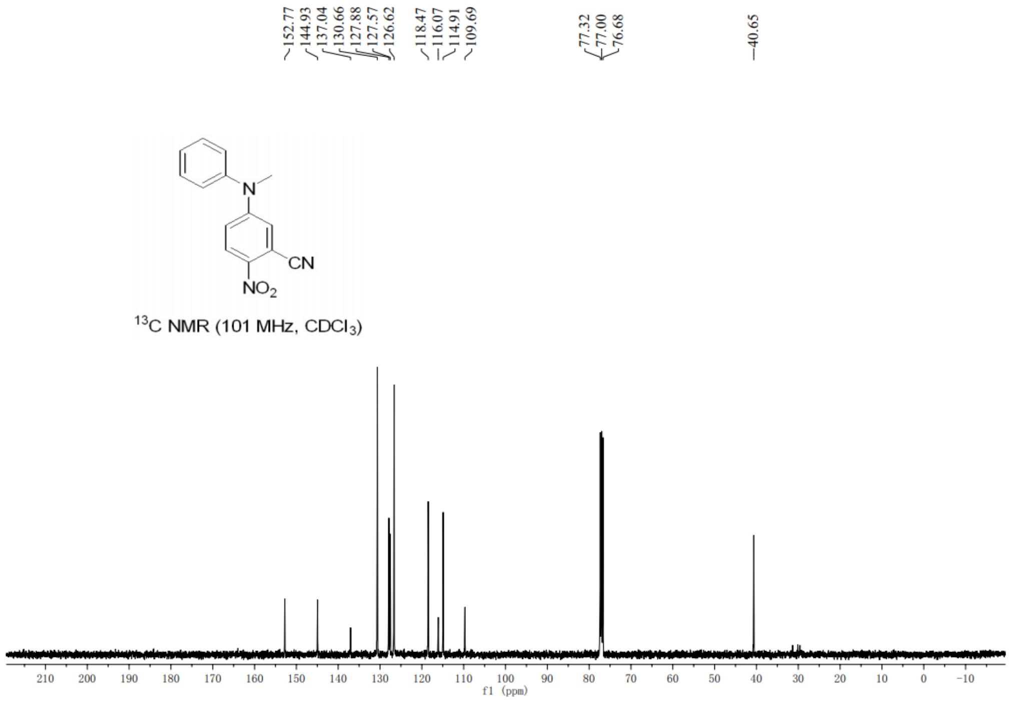

2-(3,4-dihydroquinolin-1(2*H*)-yl)-5-nitrobenzonitrile (4a-3)

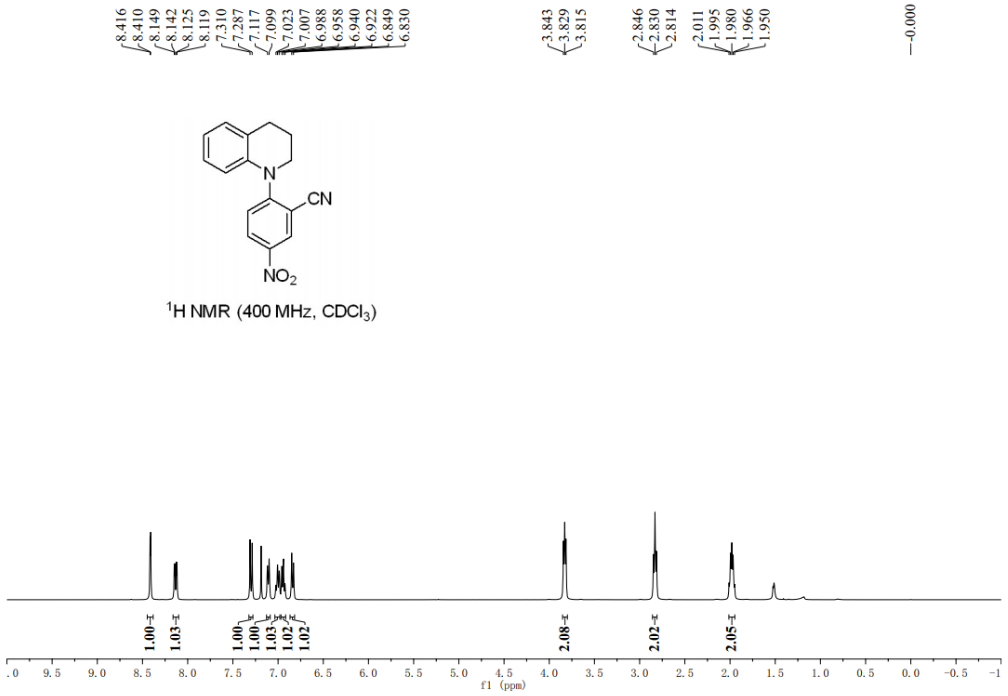

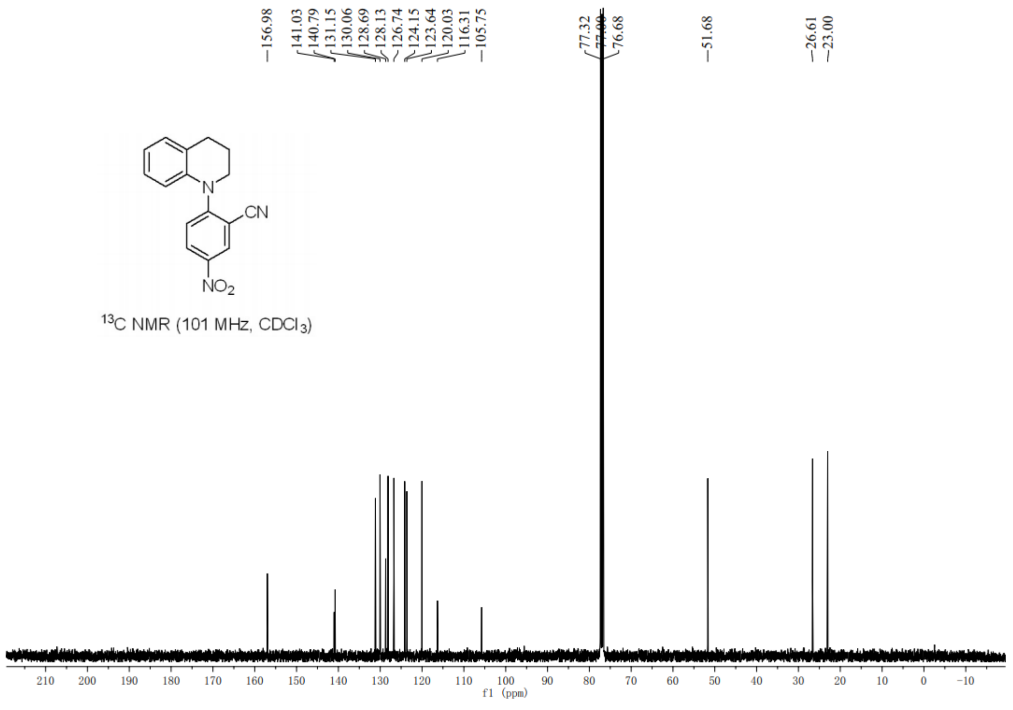

2-(indolin-1-yl)-5-nitrobenzonitrile (4b-3)

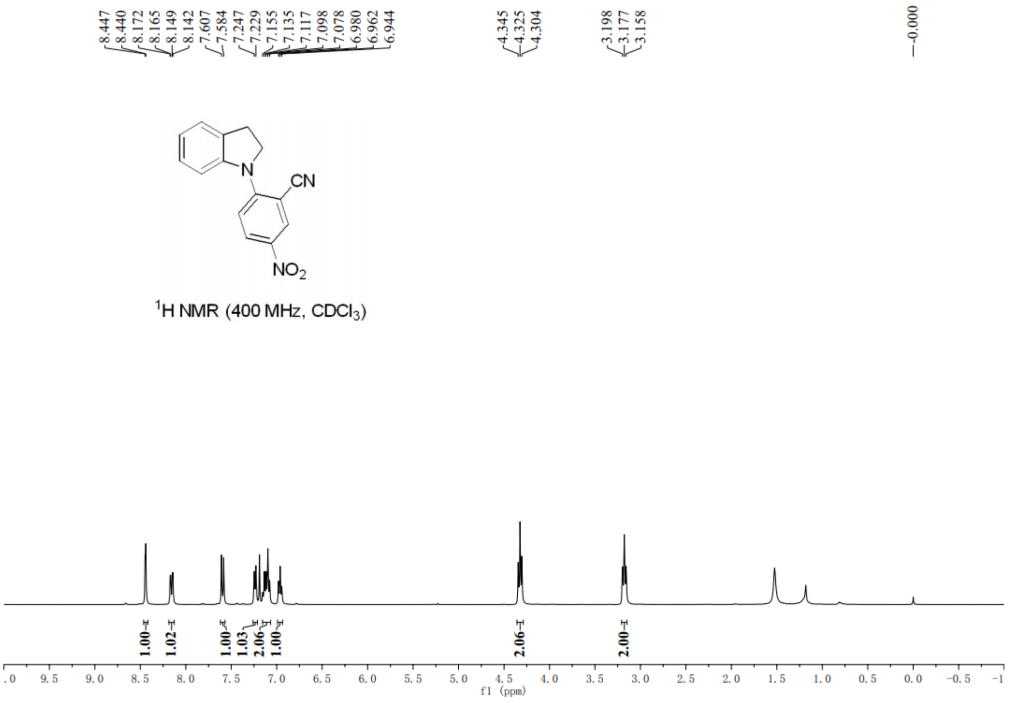

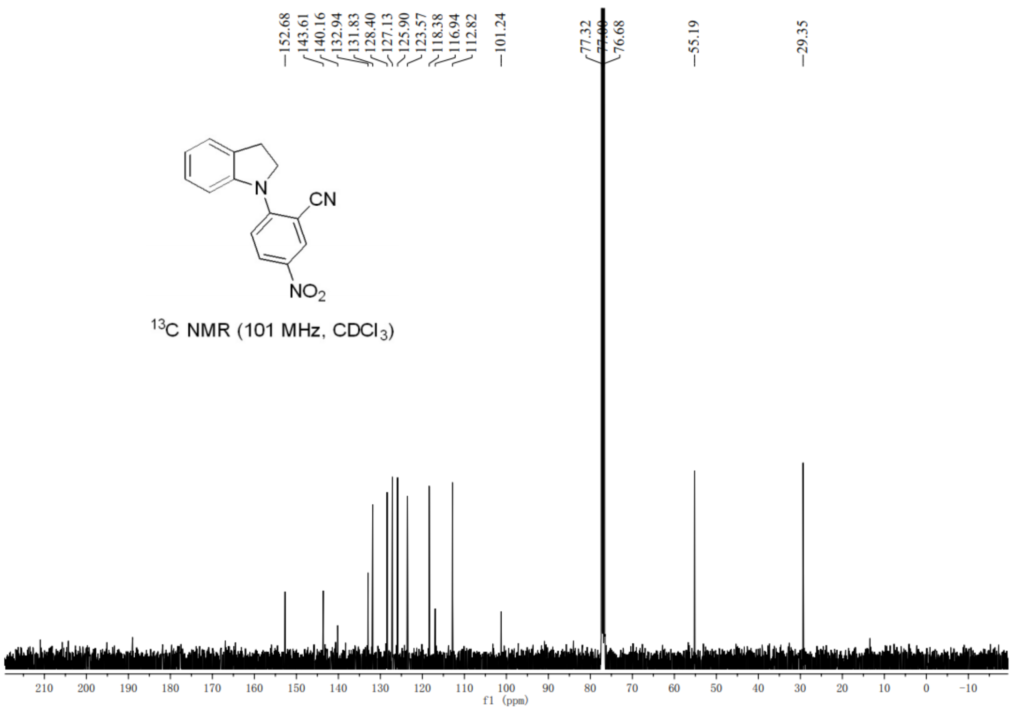

2-(methyl(phenyl)amino)-5-nitrobenzonitrile (4c-3)

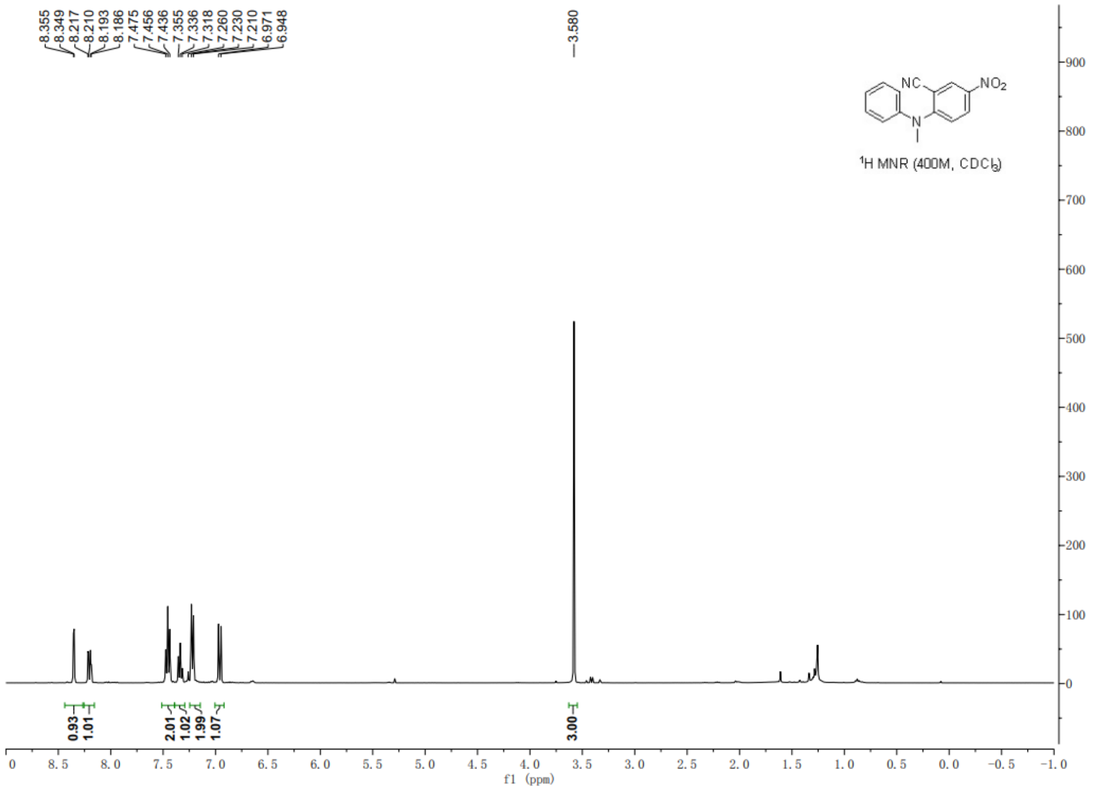

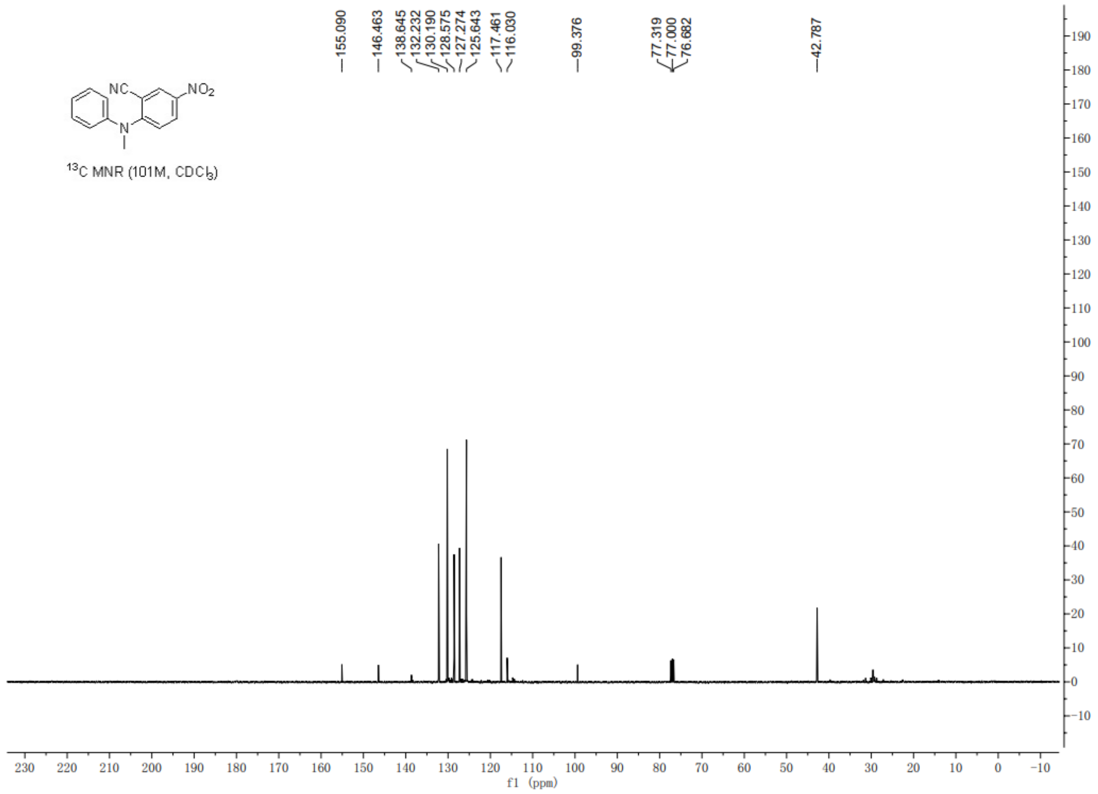

1-(4-nitronaphthalen-1-yl)-1,2,3,4-tetrahydroquinoline (4a-4)

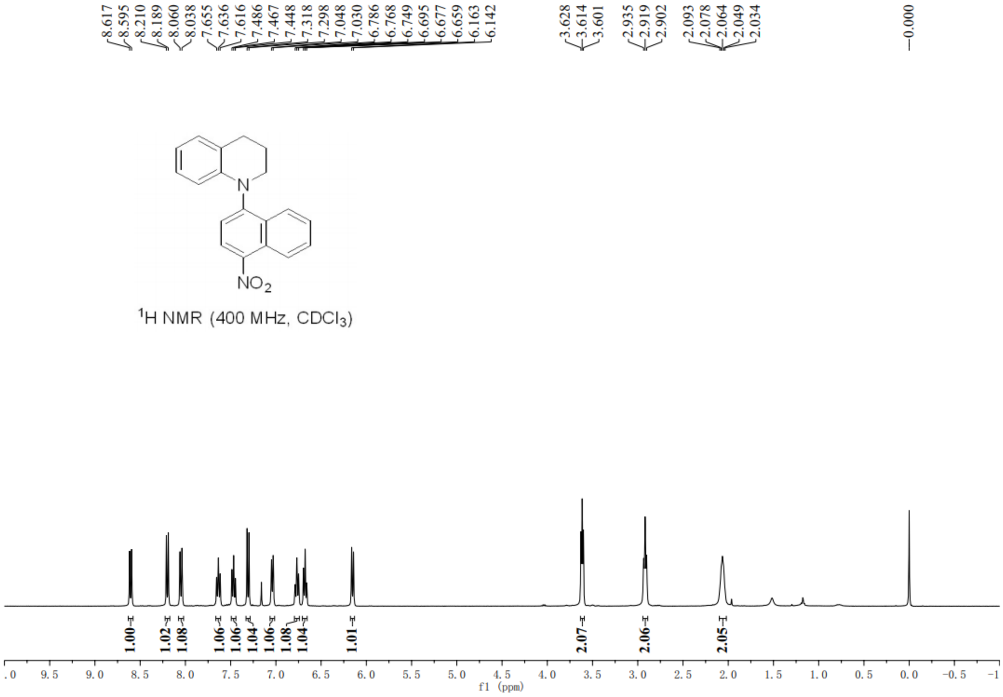

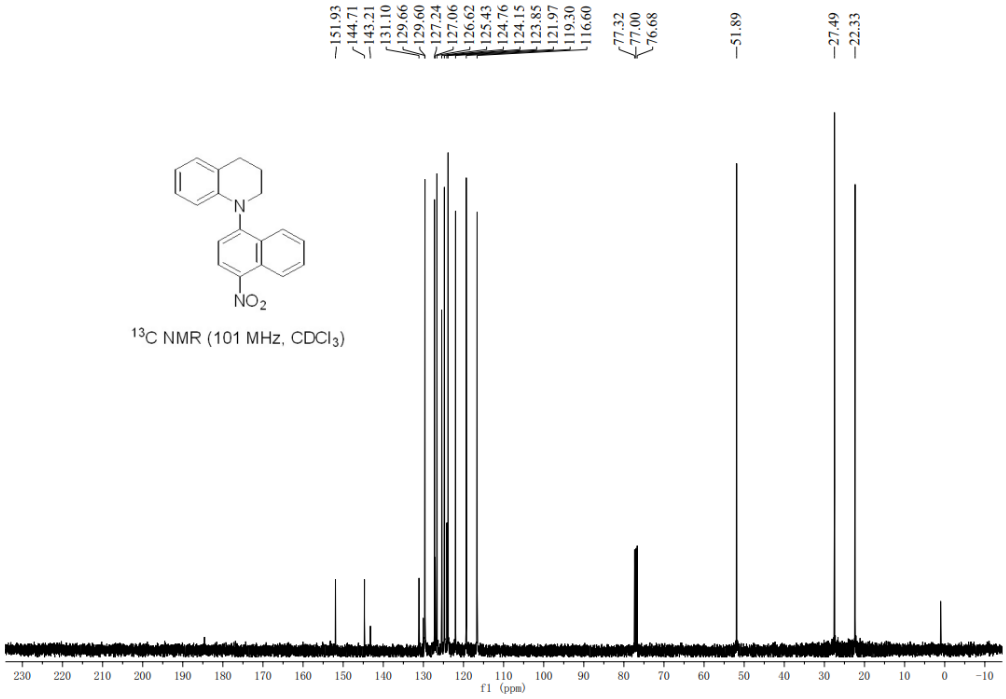

1-(4-nitronaphthalen-1-yl) indoline (4b-4)

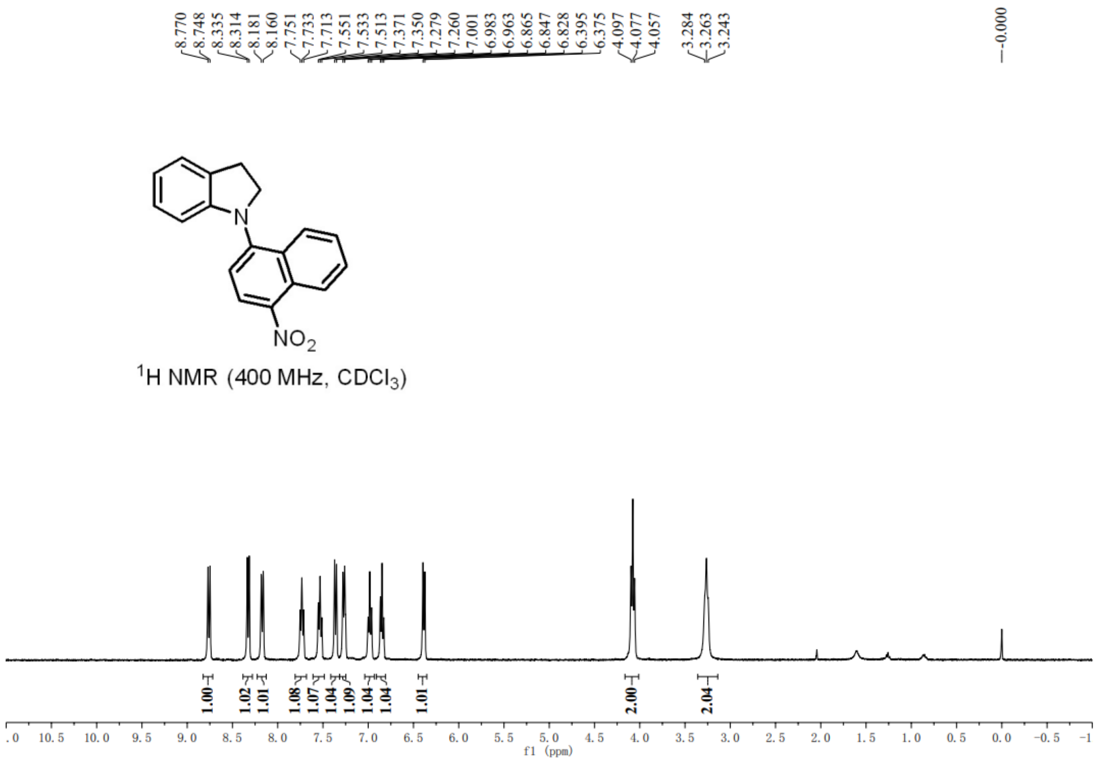

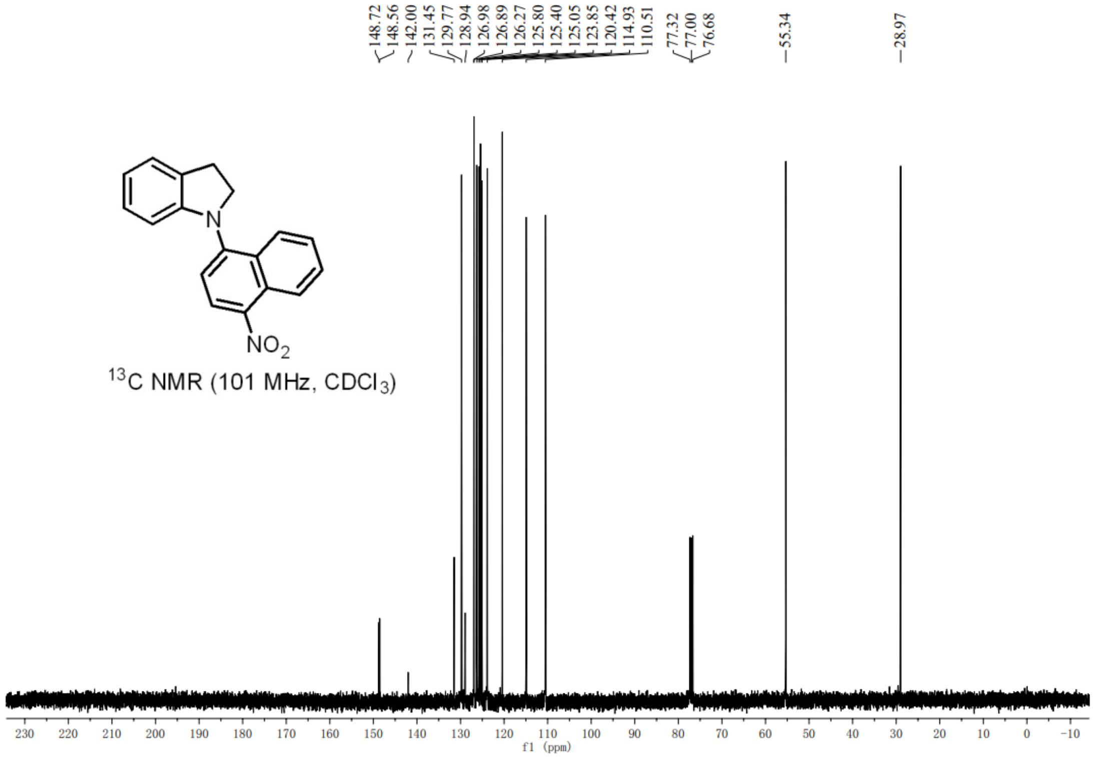

N-methyl-4-nitro-*N*-phenylnaphthalen-1-amine (4c-4)

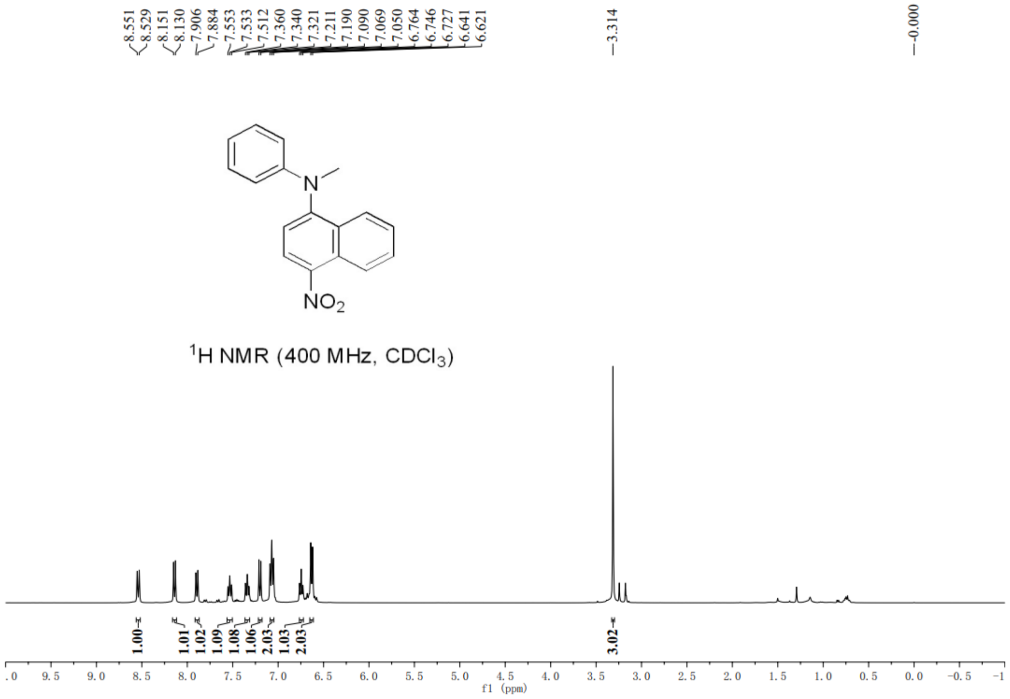

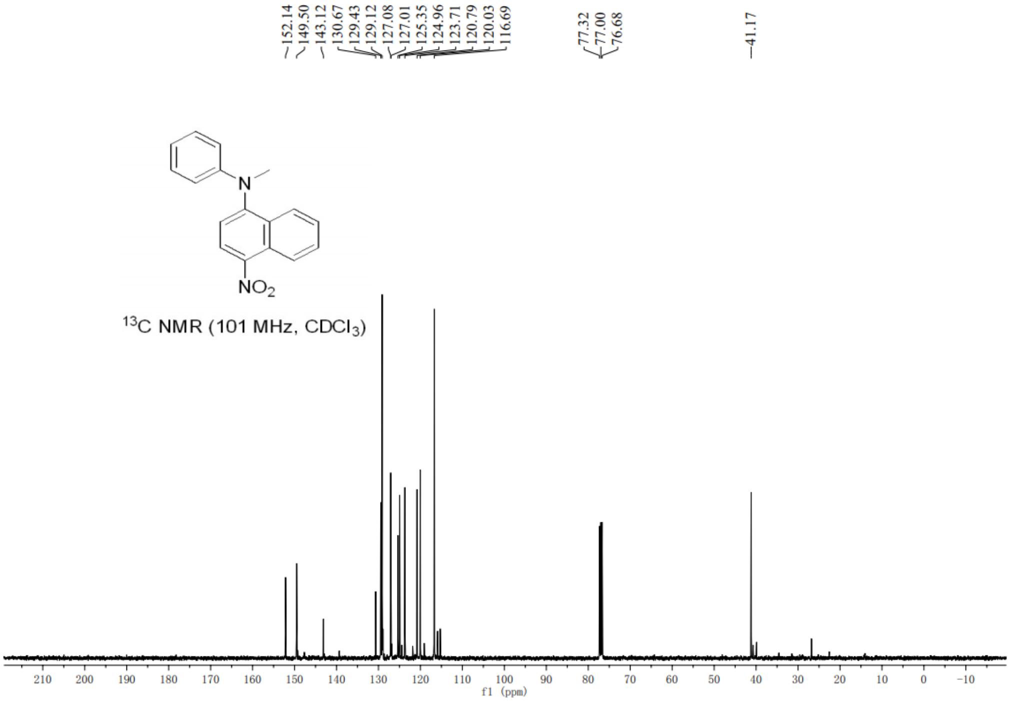

1-(3-methoxy-4-nitrophenyl)-1,2,3,4-tetrahydroquinoline (4a-5)

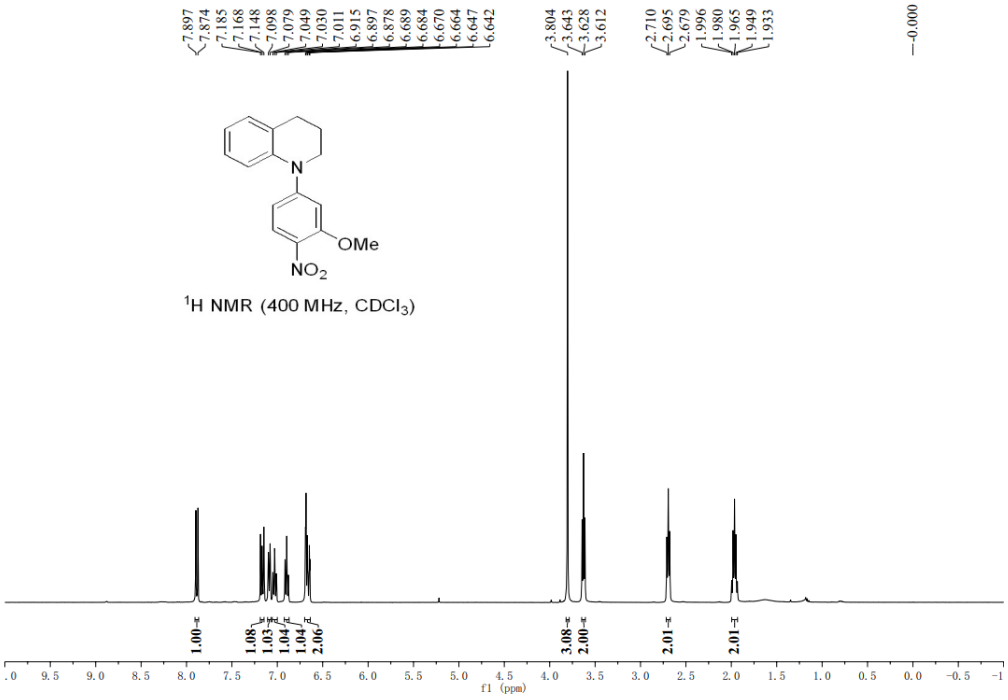

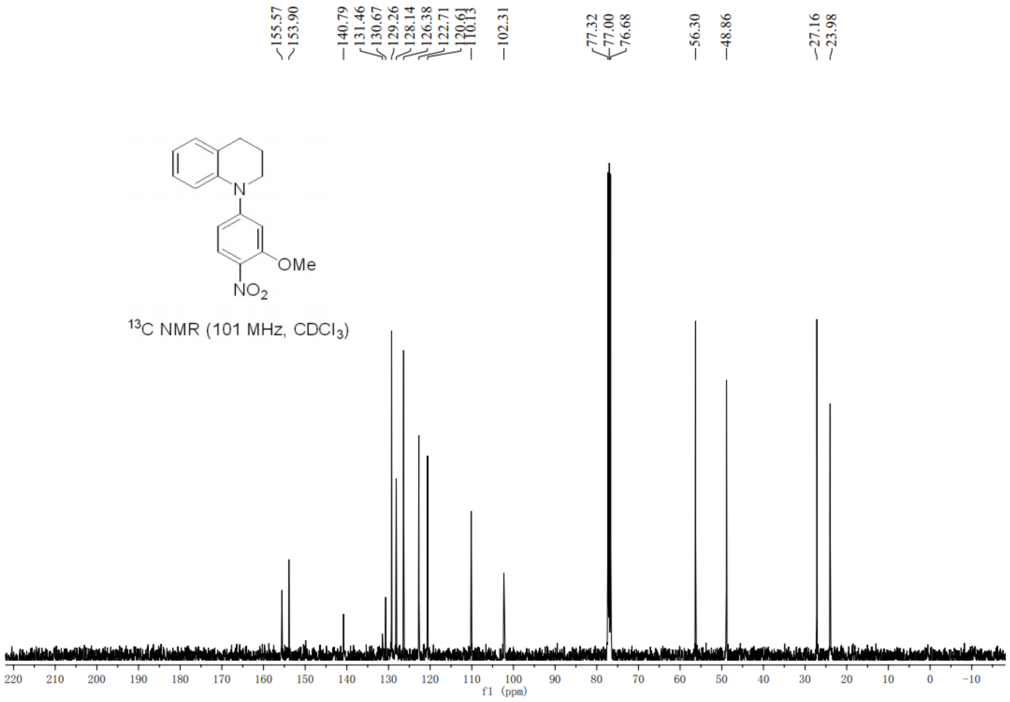

1-(3-methoxy-4-nitrophenyl) indoline (4b-5)

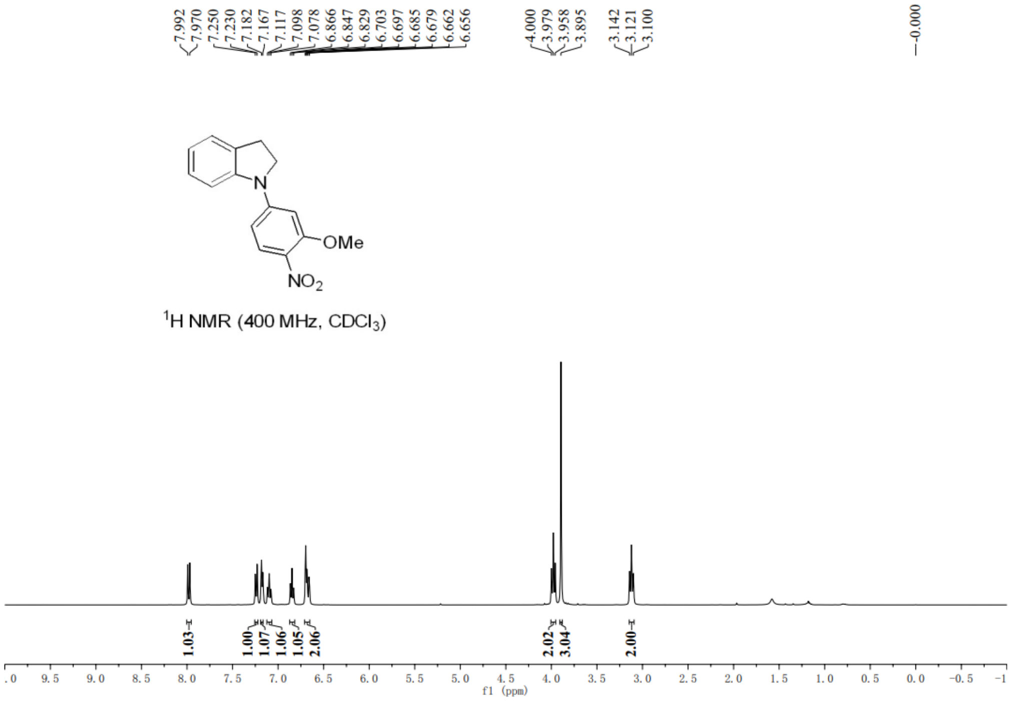

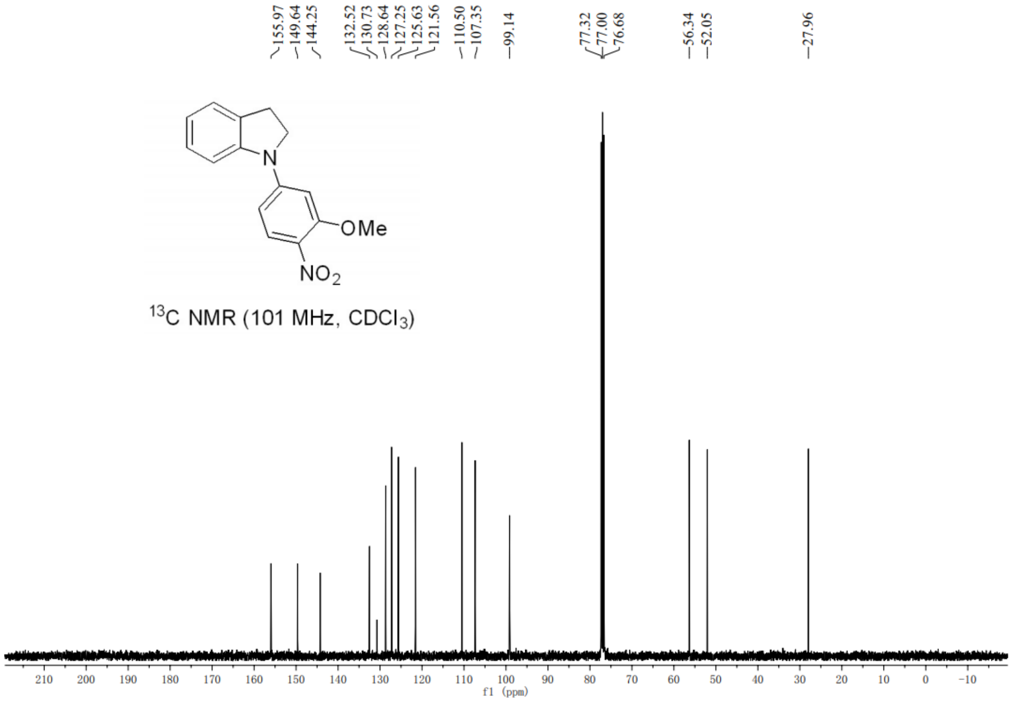

3-methoxy-*N*-methyl-4-nitro-*N*-phenylaniline (4c-5)

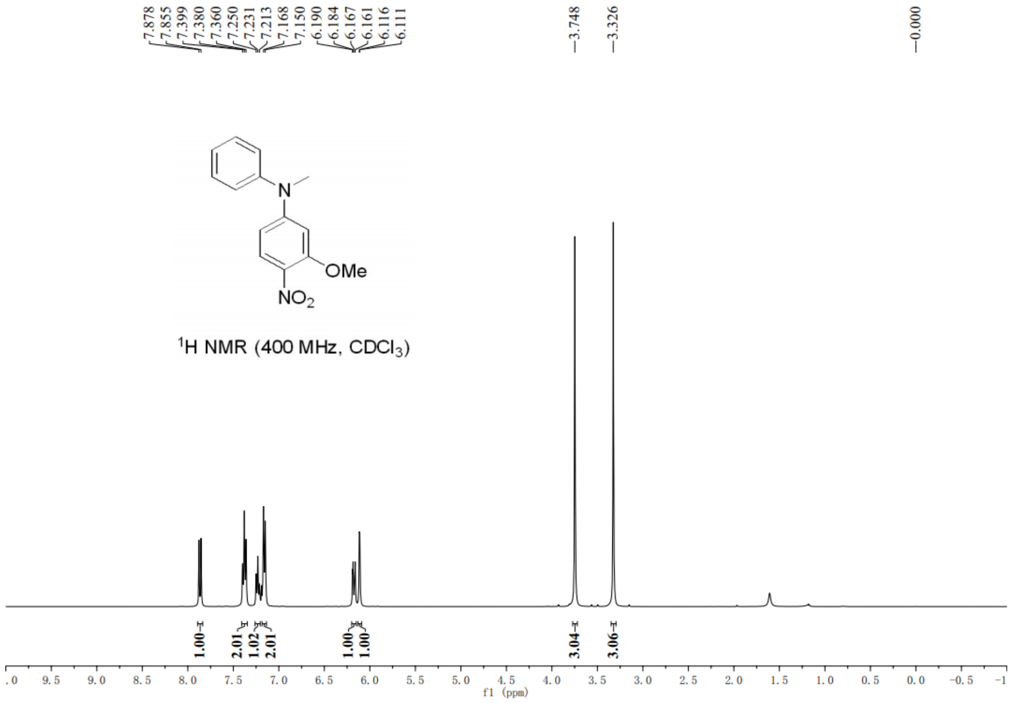

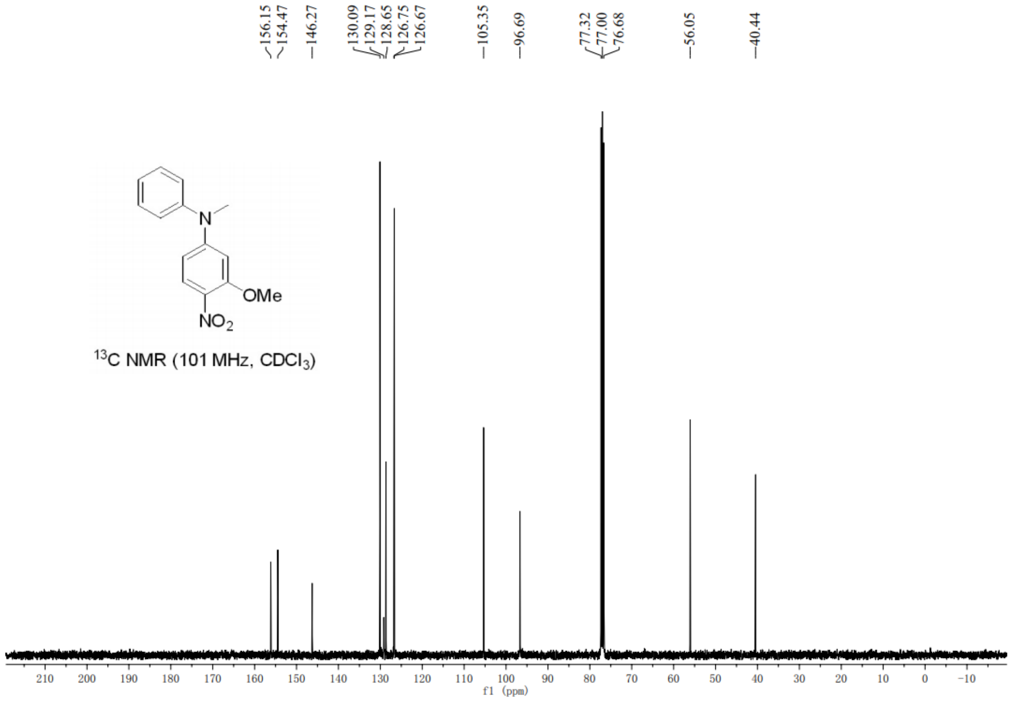

1-(2-methoxy-4-nitrophenyl)-1,2,3,4-tetrahydroquinoline (4a-6)

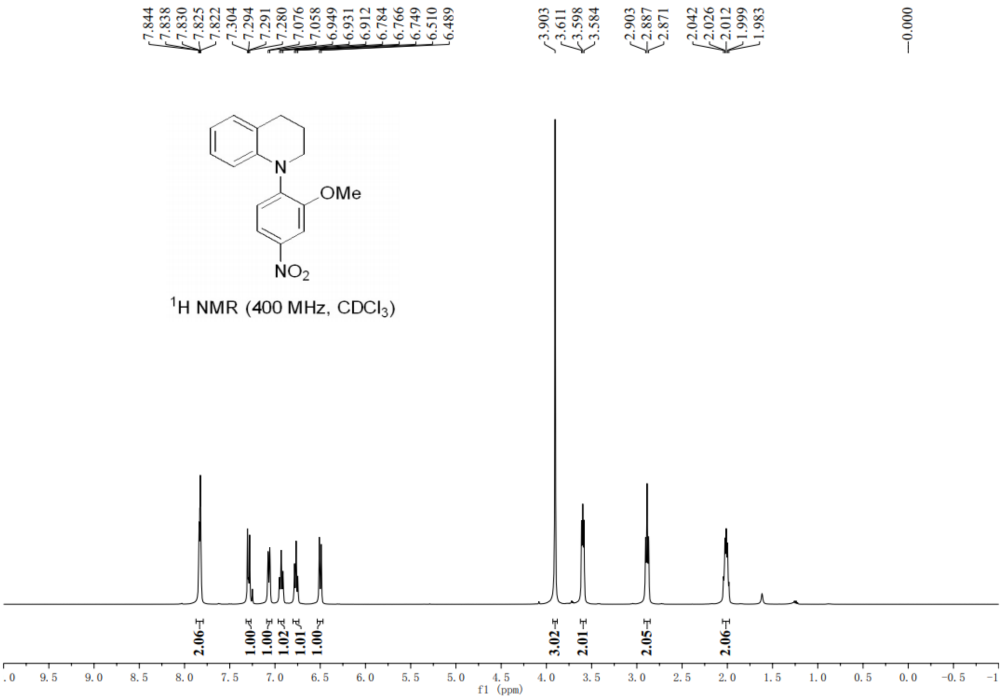

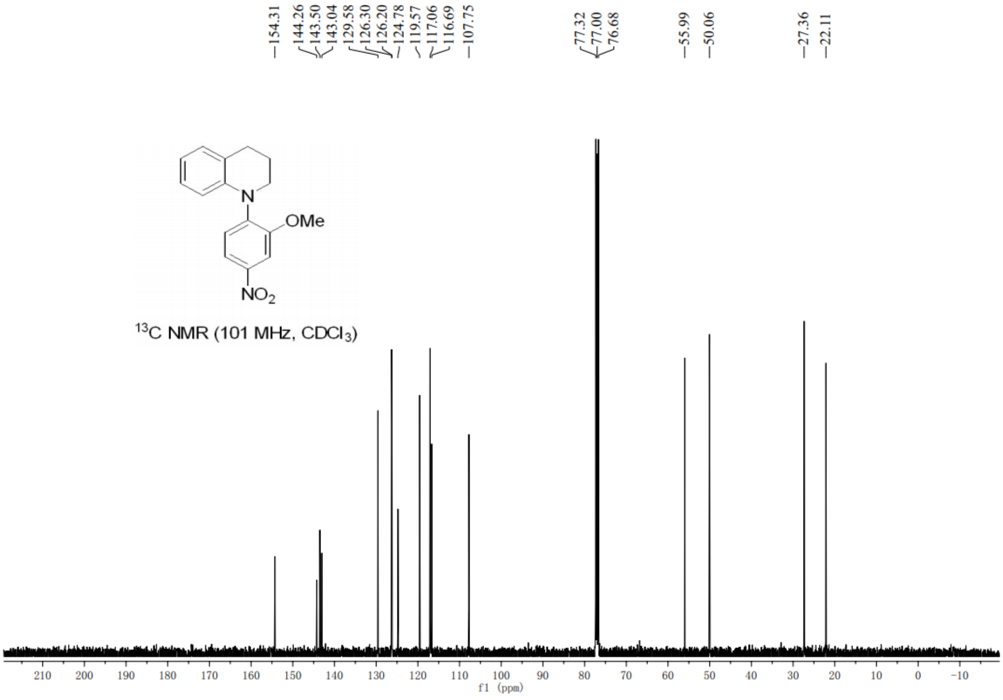

1-(2-methoxy-4-nitrophenyl) indoline (4b-6)

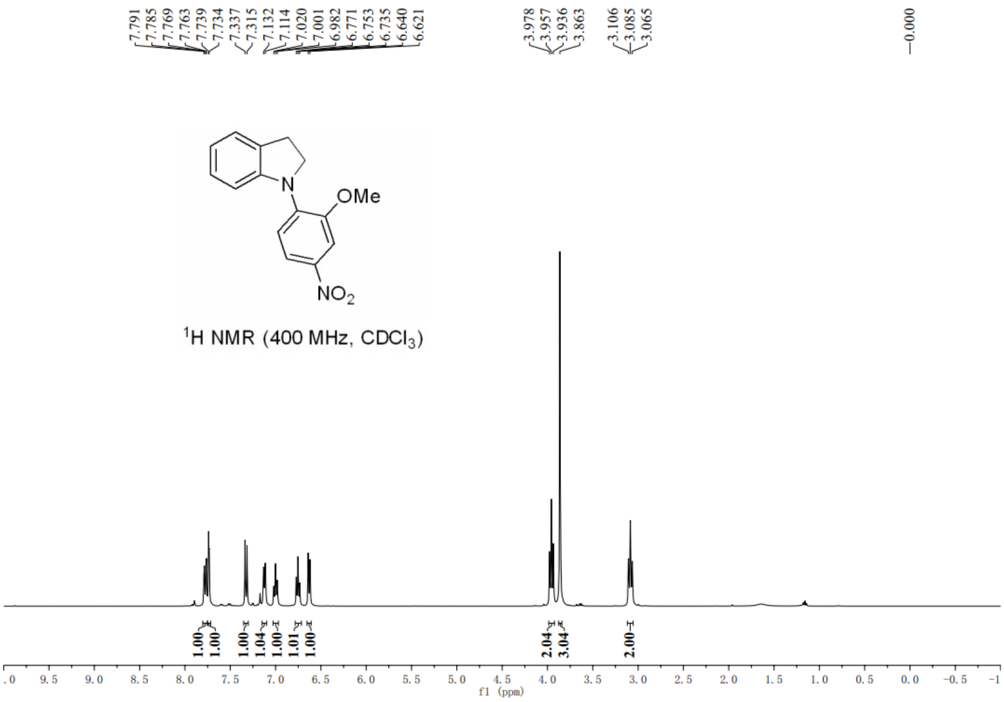

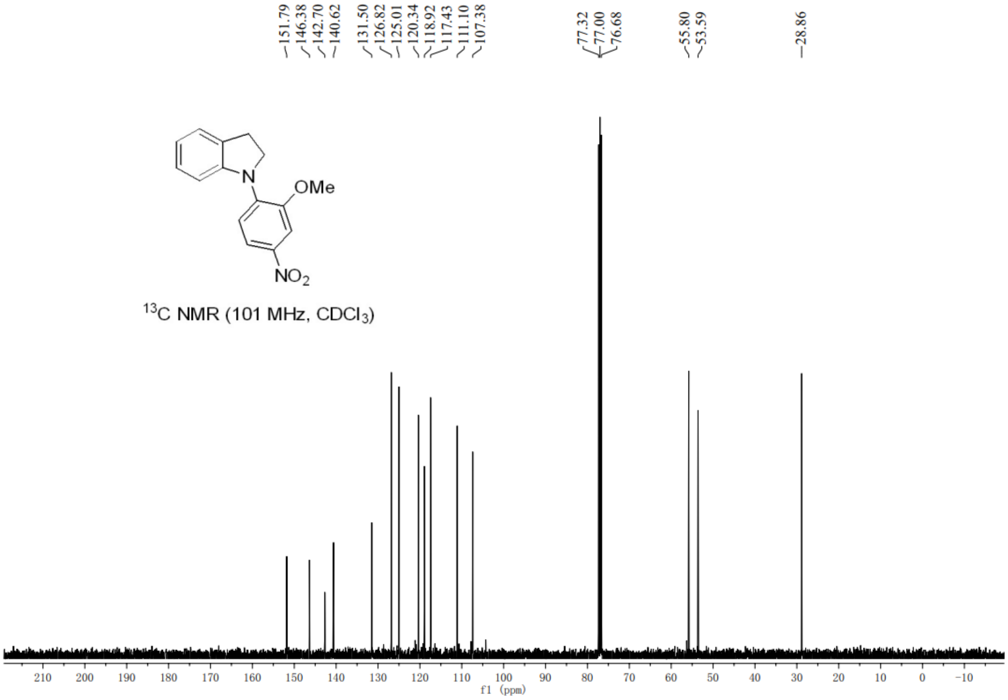

2-methoxy-*N*-methyl-4-nitro-*N*-phenylaniline (4c-6)

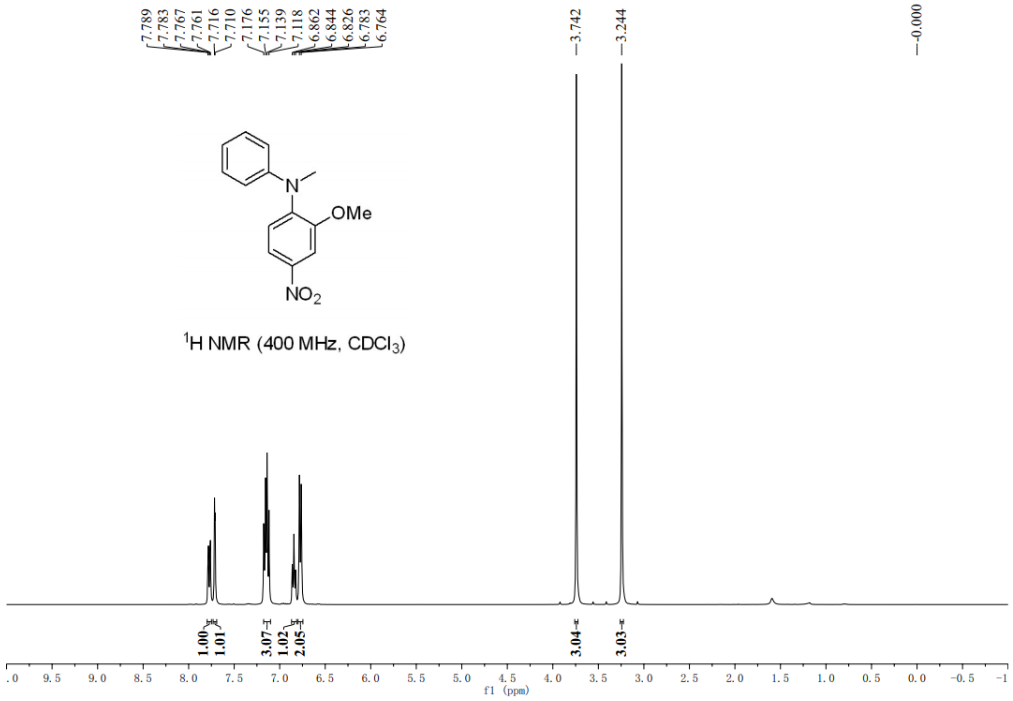

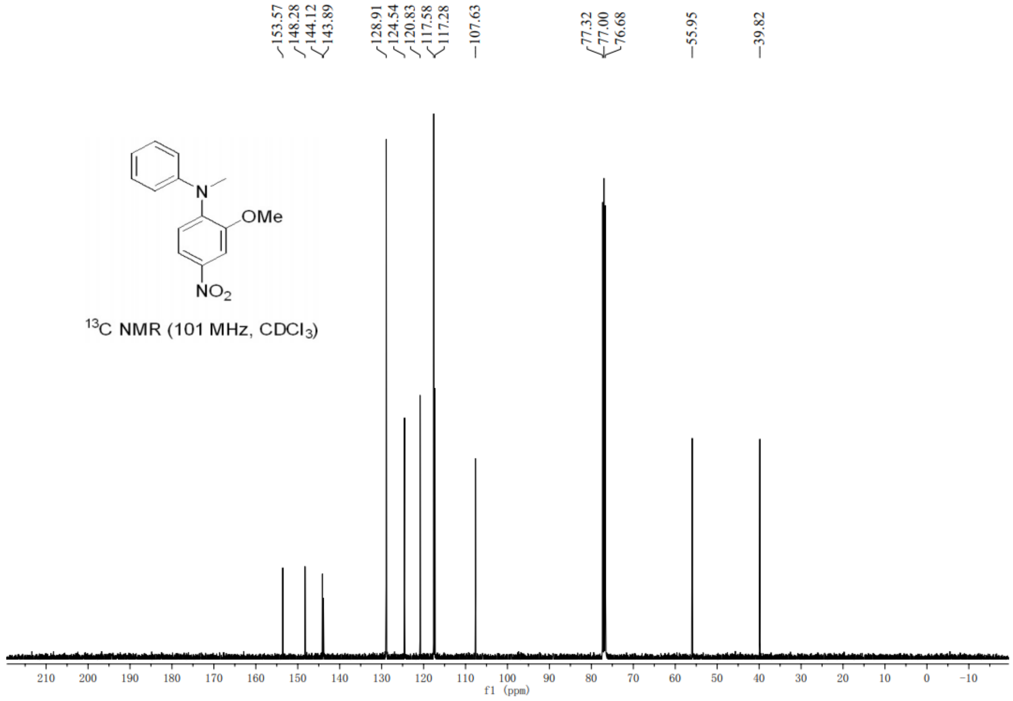

1-(2-fluoro-4-nitrophenyl)-1,2,3,4-tetrahydroquinoline (4a-7)

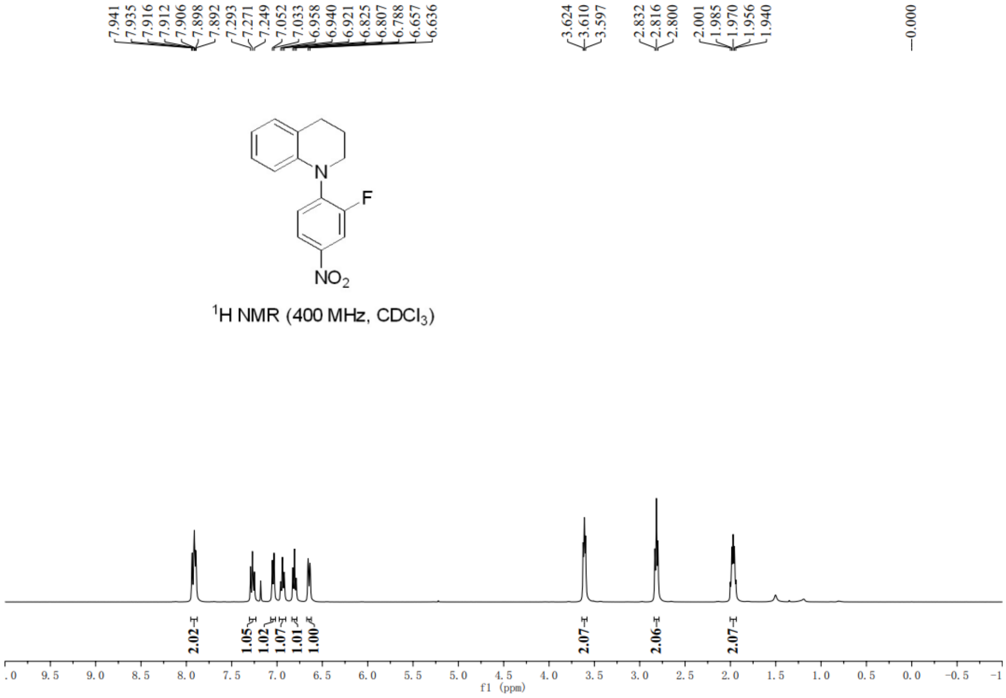

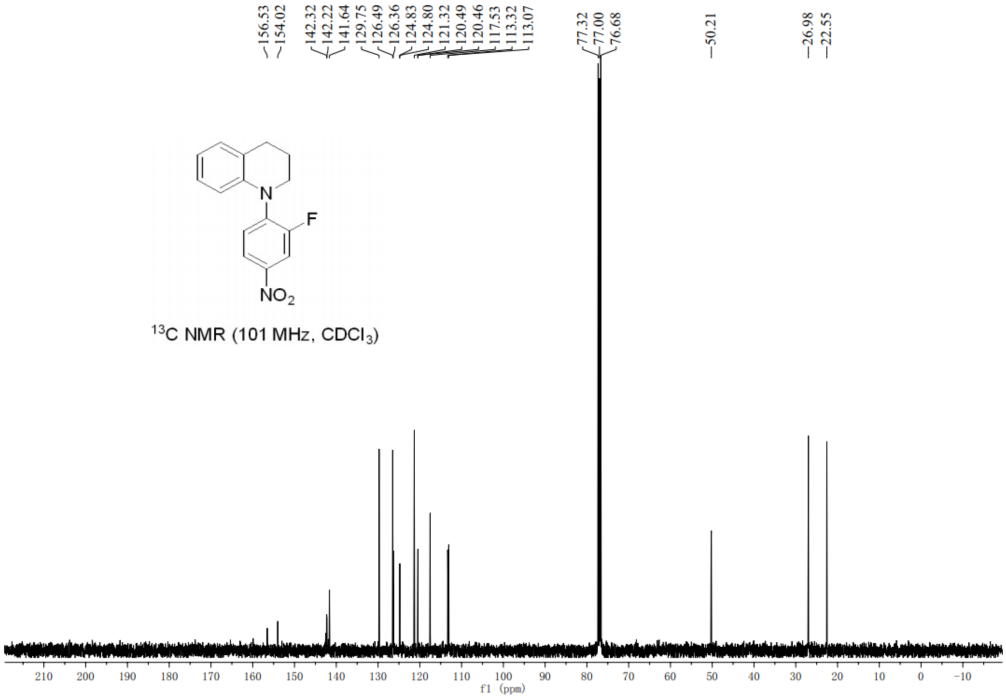

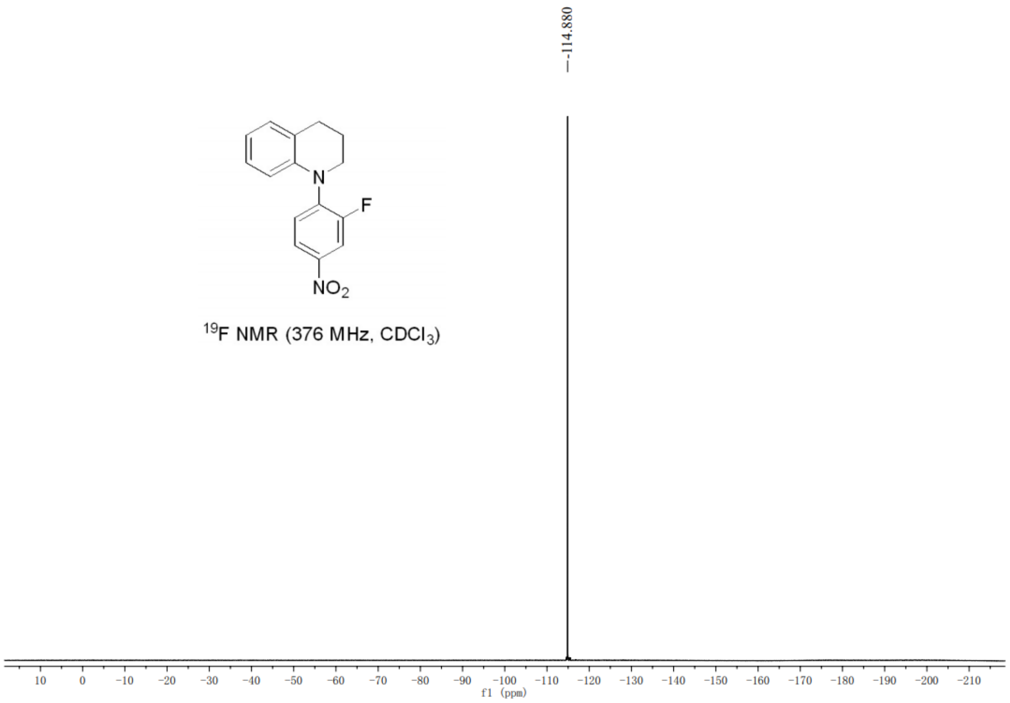

-114.880
N
F
NO2
19F NMR (376 MHz, CDCl3)
10 0 −10 −20 −30 −40 −50 −60 −70 −80 −90 −100 −110 −120 −130 −140 −150 −160 −170 −180 −190 −200 −210
f1 (ppm)

1-(2-chloro-4-nitrophenyl)-1,2,3,4-tetrahydroquinoline (4a-8)

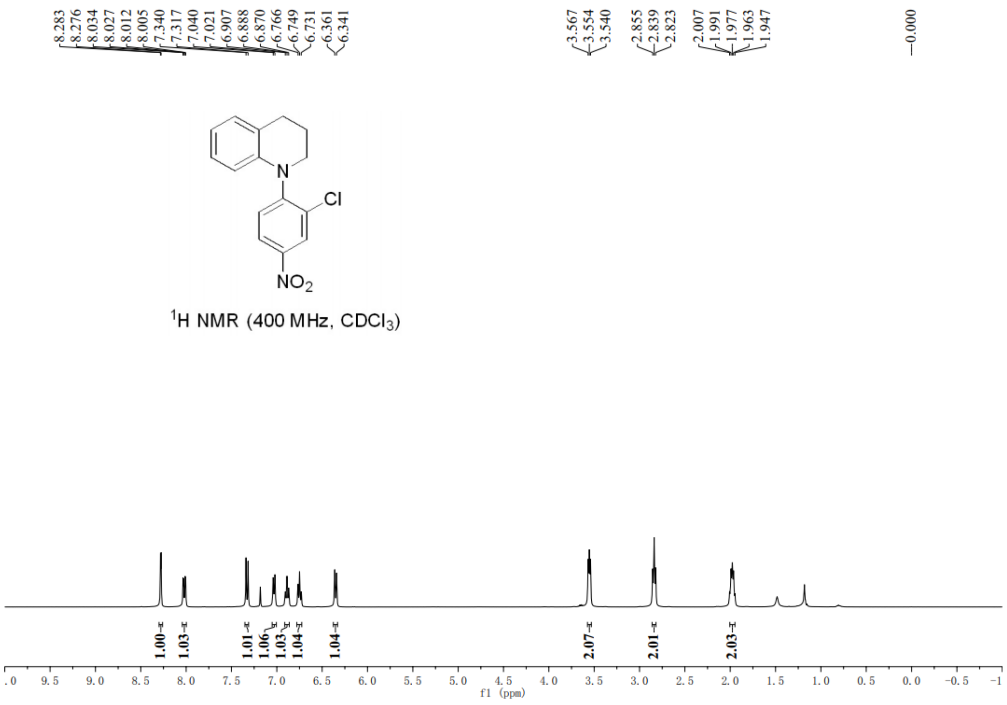

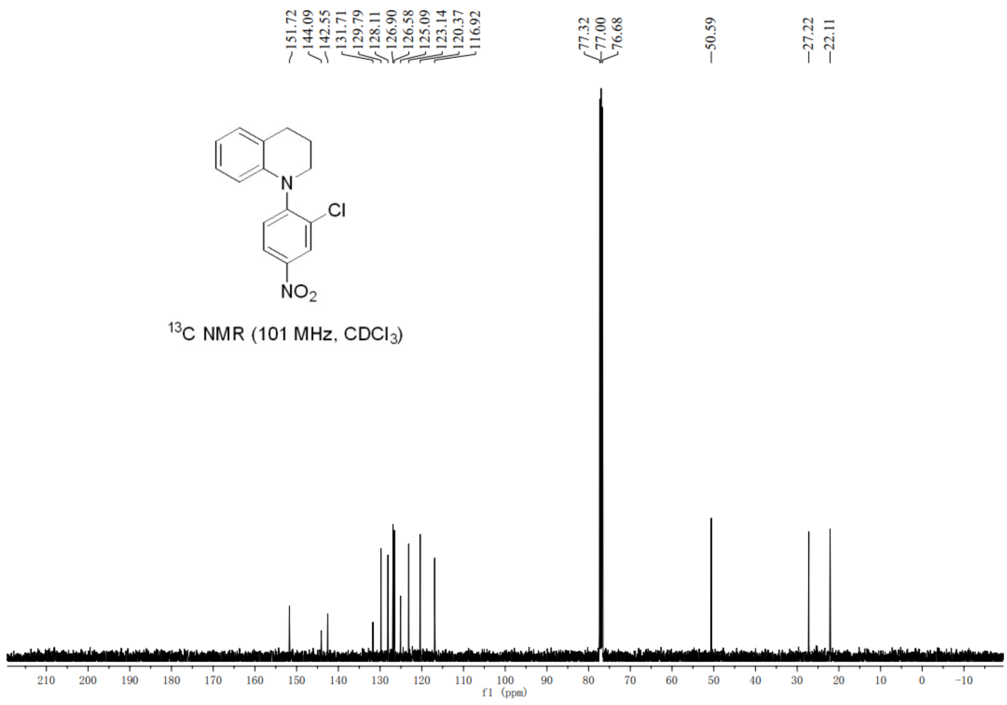

1-(2-bromo-4-nitrophenyl)-1,2,3,4-tetrahydroquinoline (4a-9)

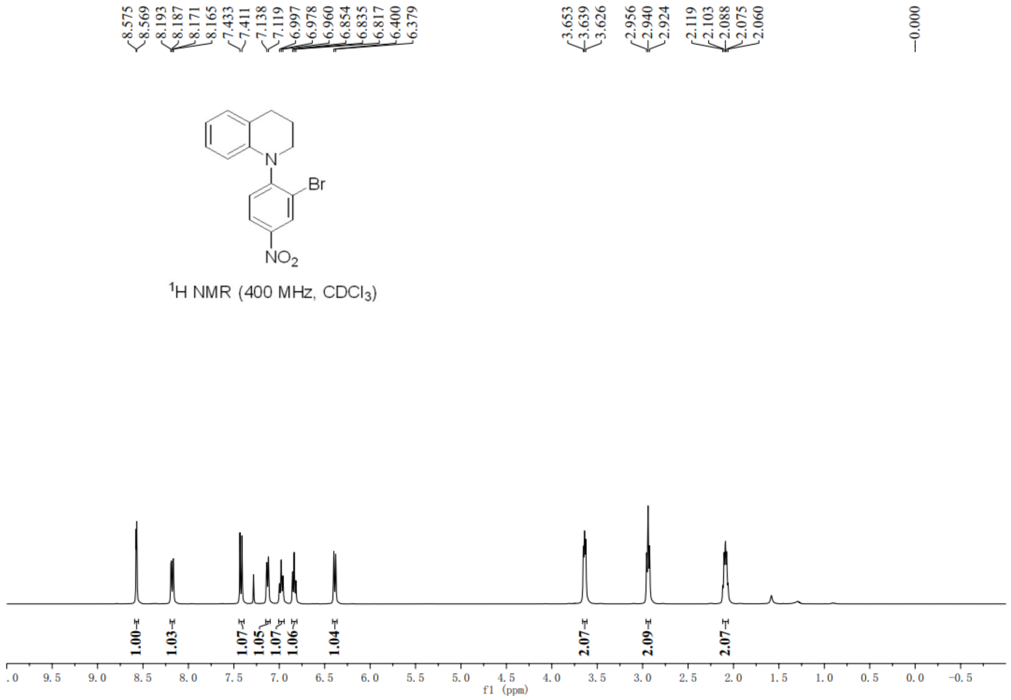

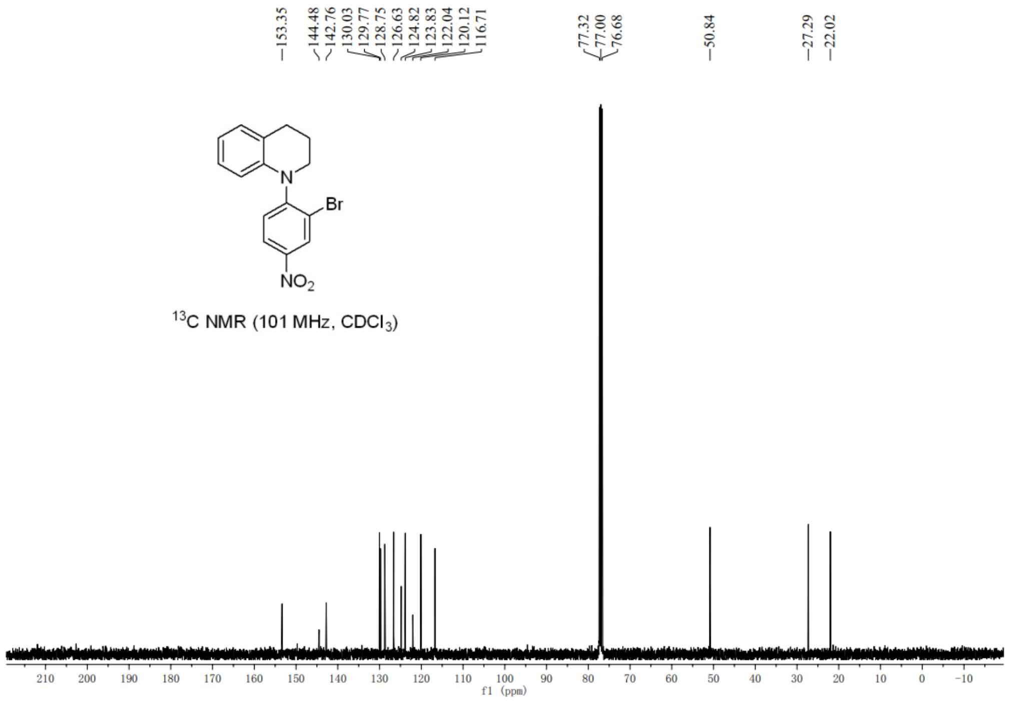

1-(2-fluoro-4-nitrophenyl) indoline (4b-7)

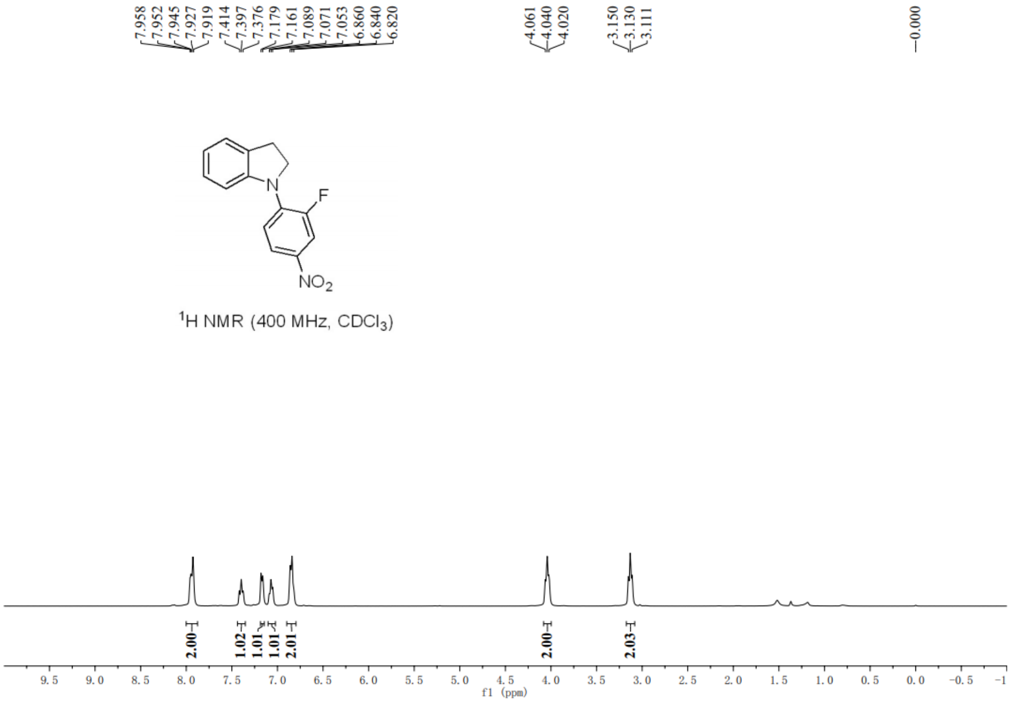

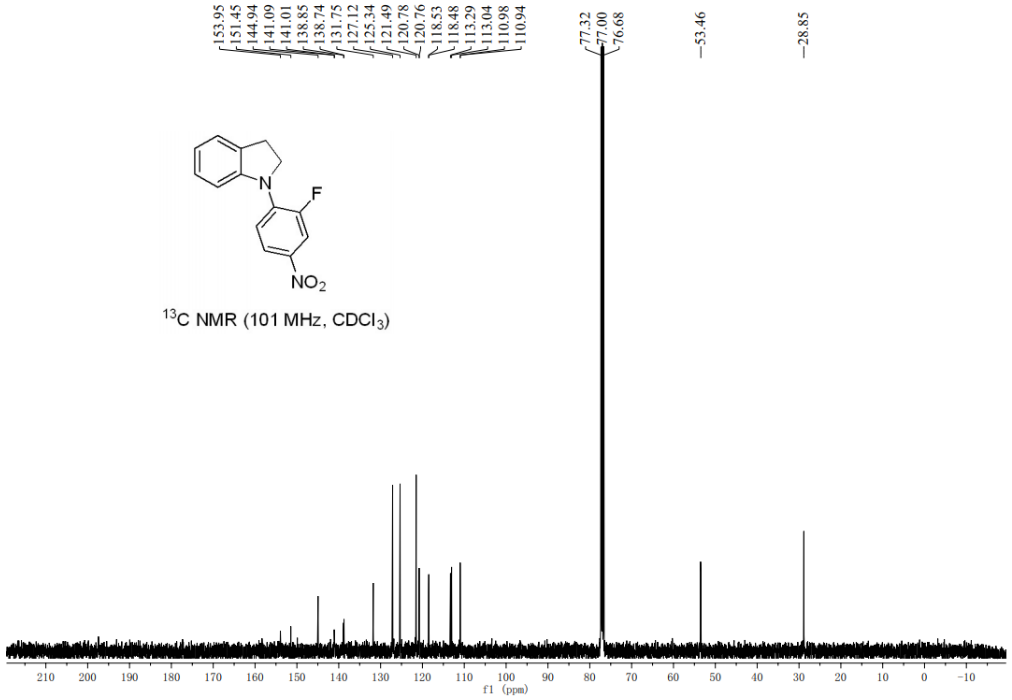

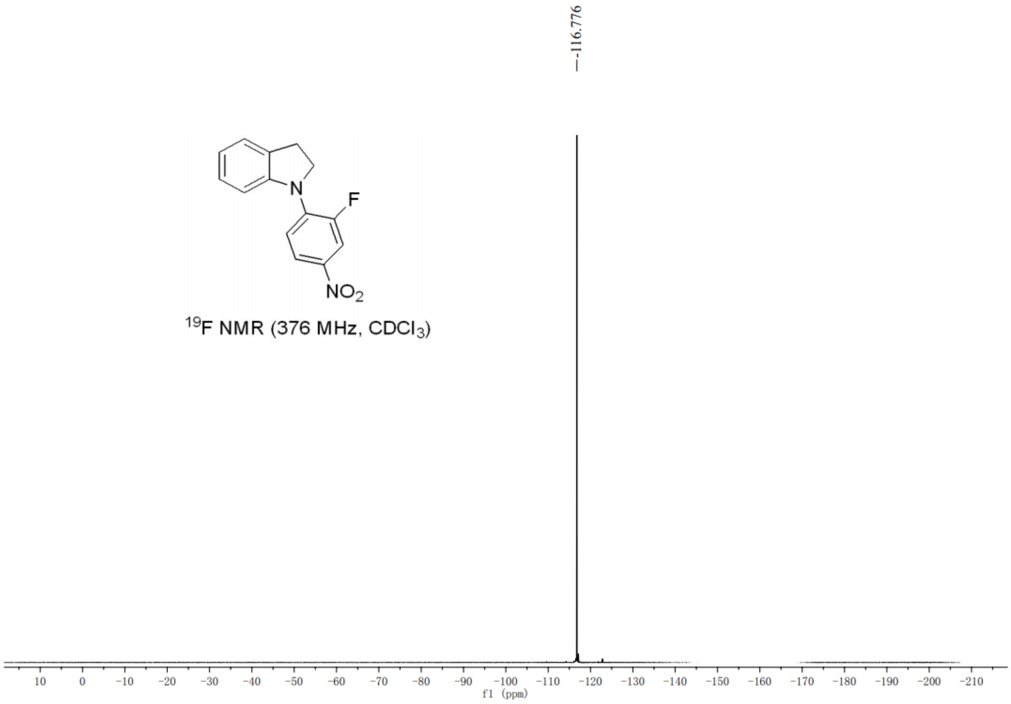

-116.776
F
N
NO2
19F NMR (376 MHz, CDCl3)
10 0 −10 −20 −30 −40 −50 −60 −70 −80 −90 −100 −110 −120 −130 −140 −150 −160 −170 −180 −190 −200 −210
f1 (ppm)

1-(2-chloro-4-nitrophenyl) indoline (4b-8)

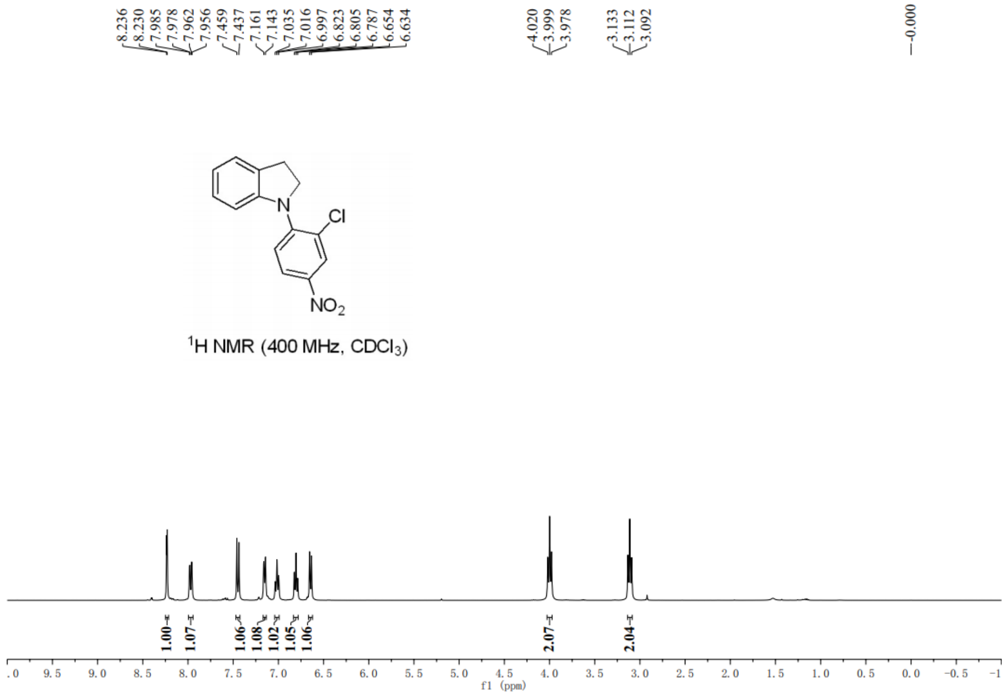

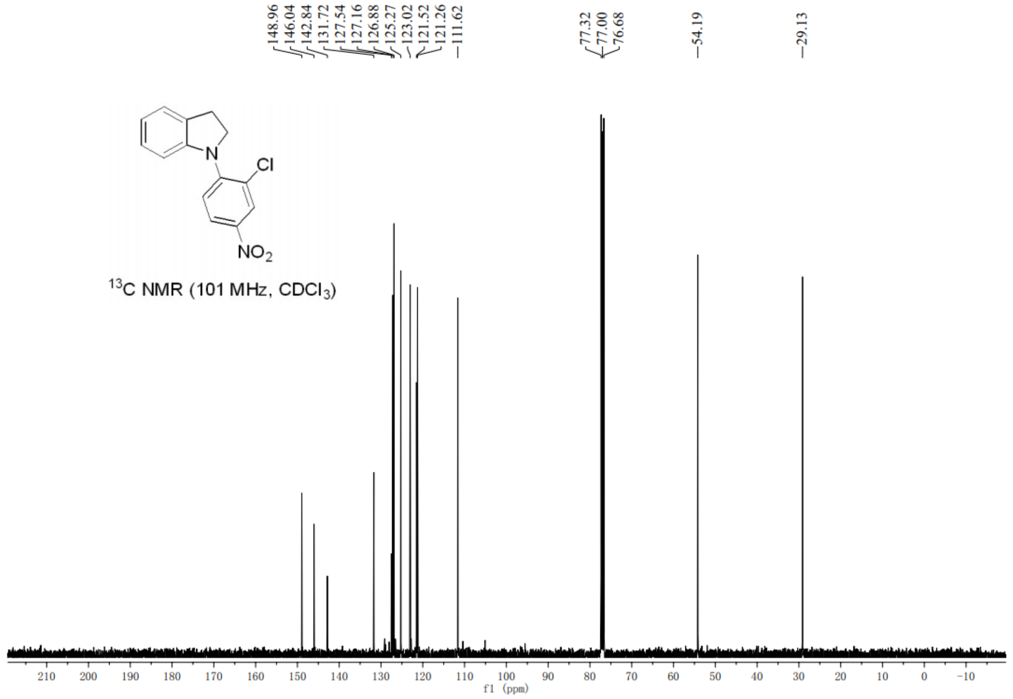

1-(2-bromo-4-nitrophenyl) indoline (4b-9)

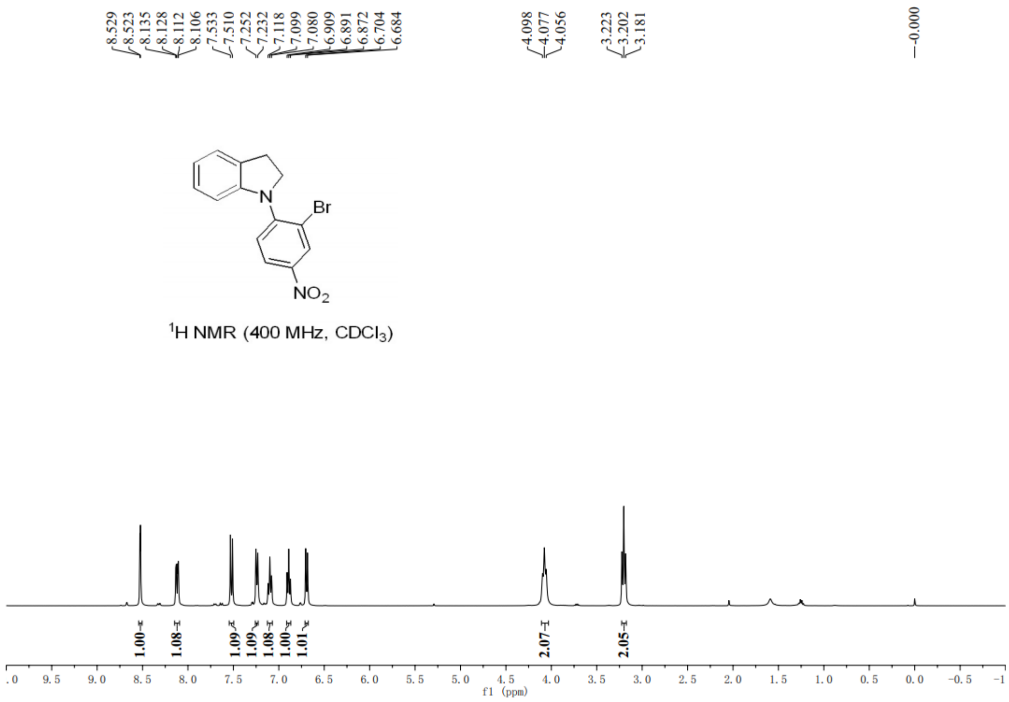

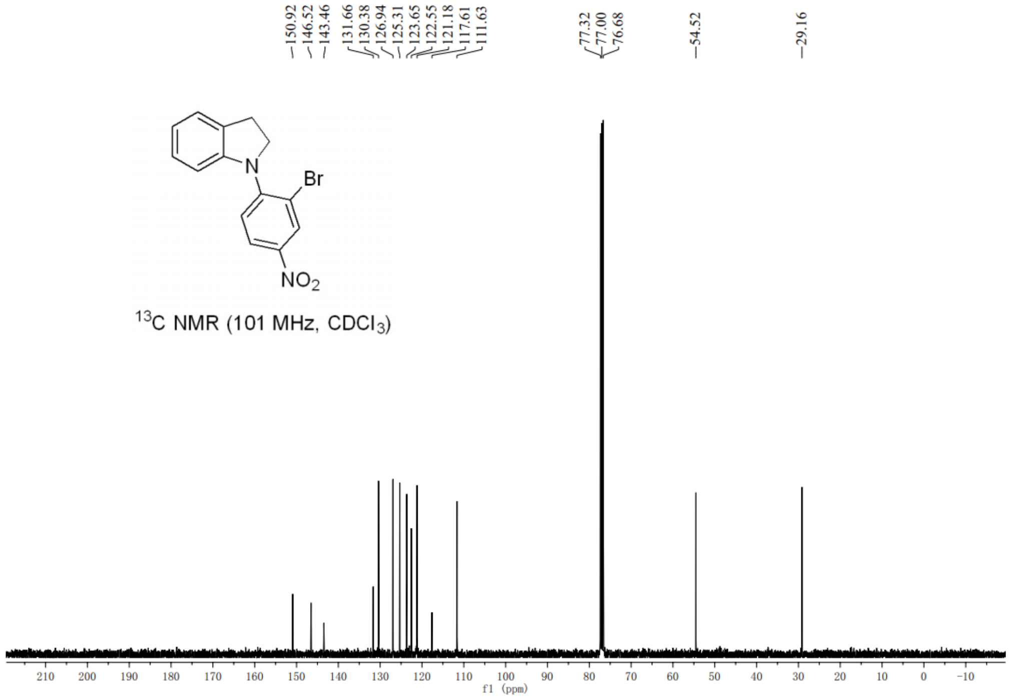

2-fluoro-*N*-methyl-4-nitro-*N*-phenylaniline (4c-7)

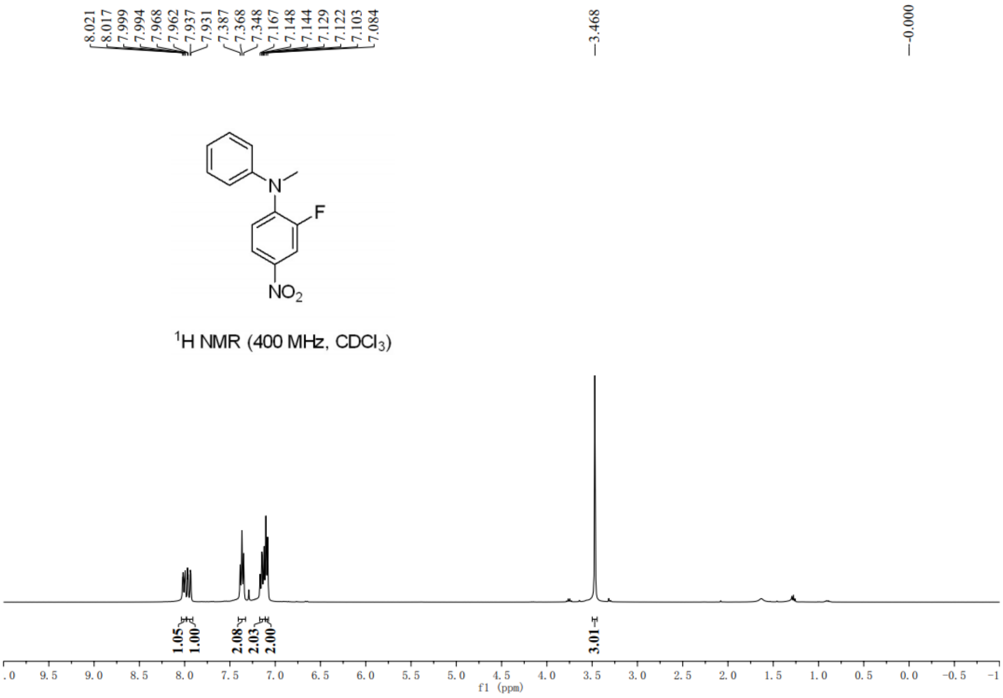

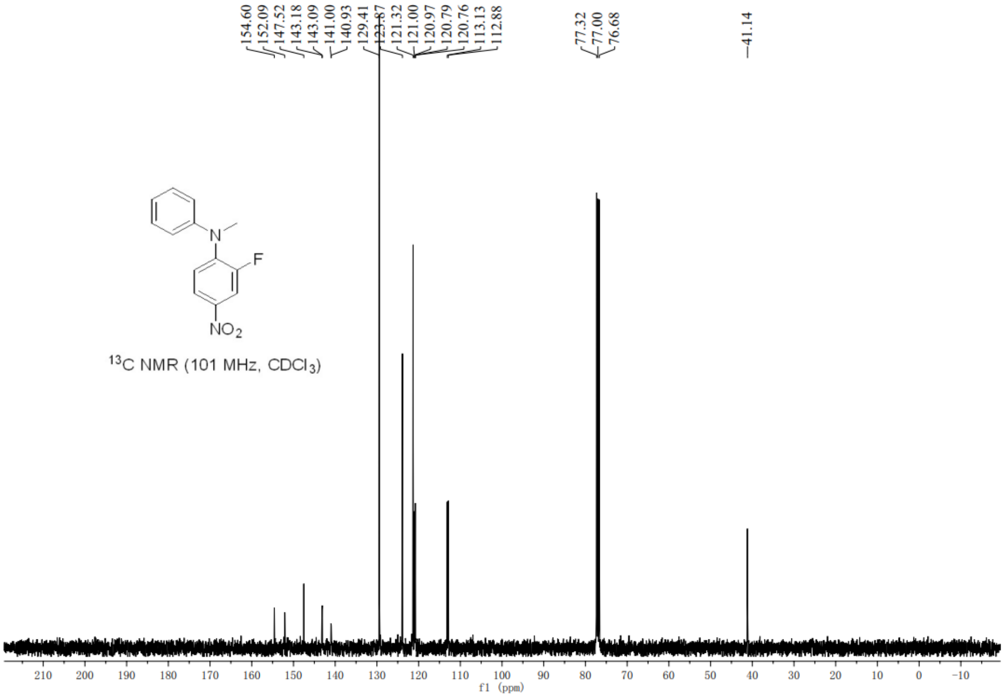

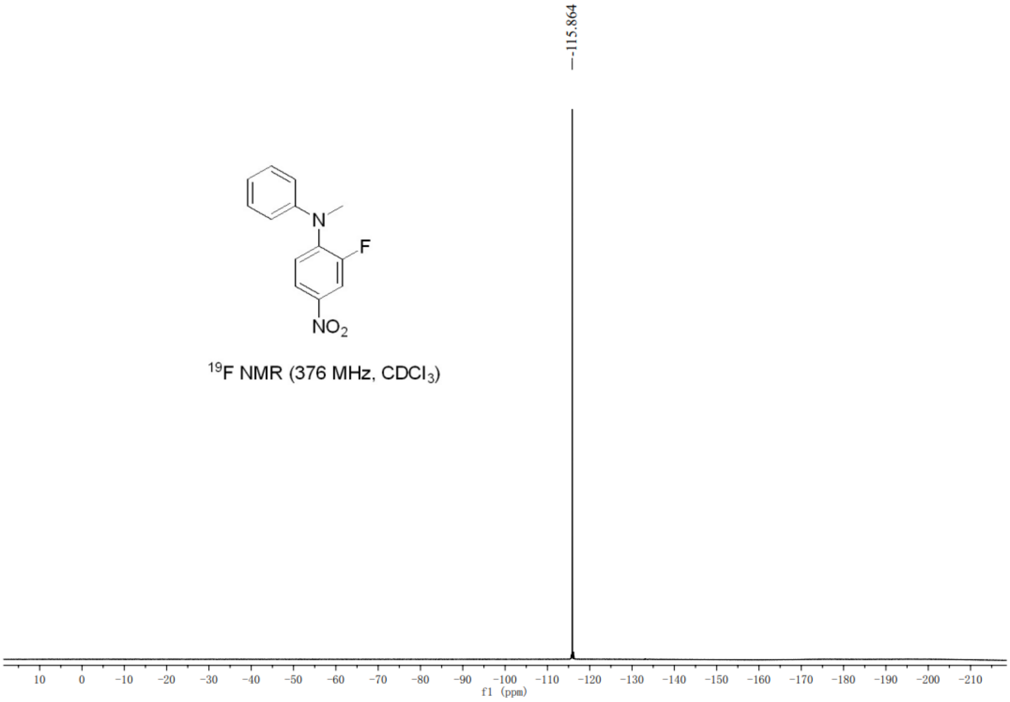
-115.864
F
N
NO2
19F NMR (376 MHz, CDCl3)
f1 (ppm)

2-chloro-*N*-methyl-4-nitro-*N*-phenylaniline (4c-8)

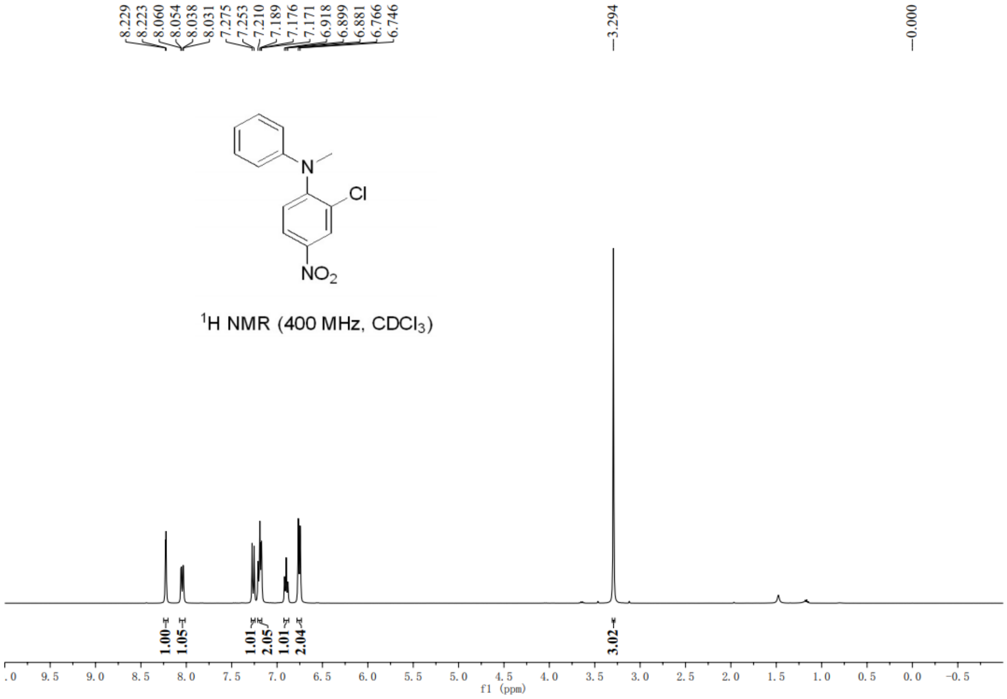

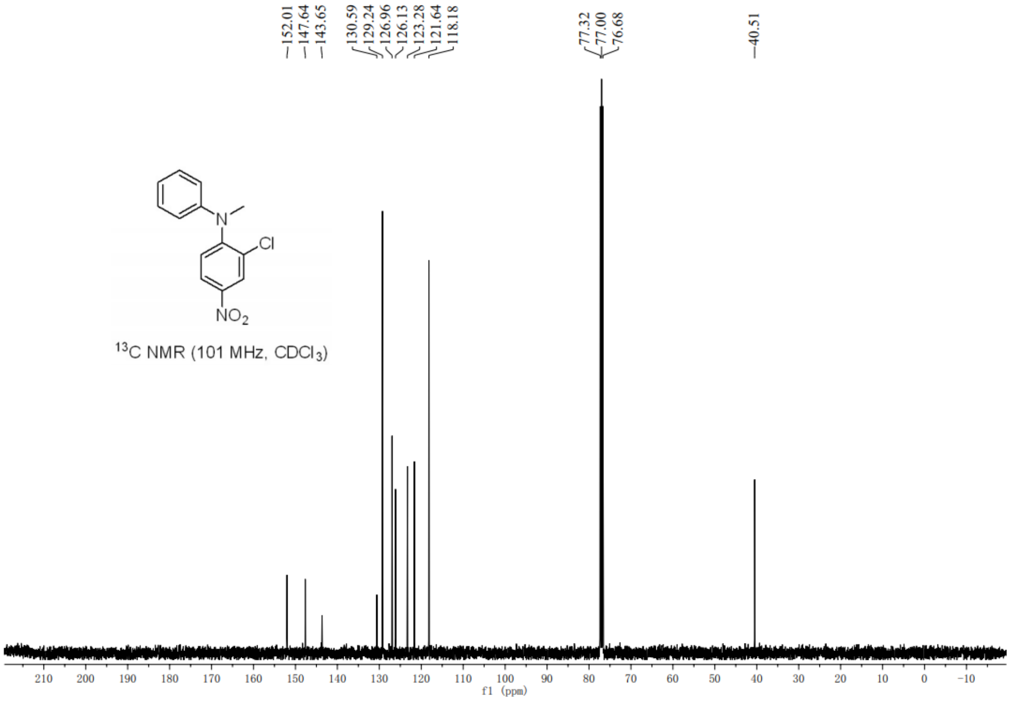

2-bromo-*N*-methyl-4-nitro-*N*-phenylaniline (4c-9)

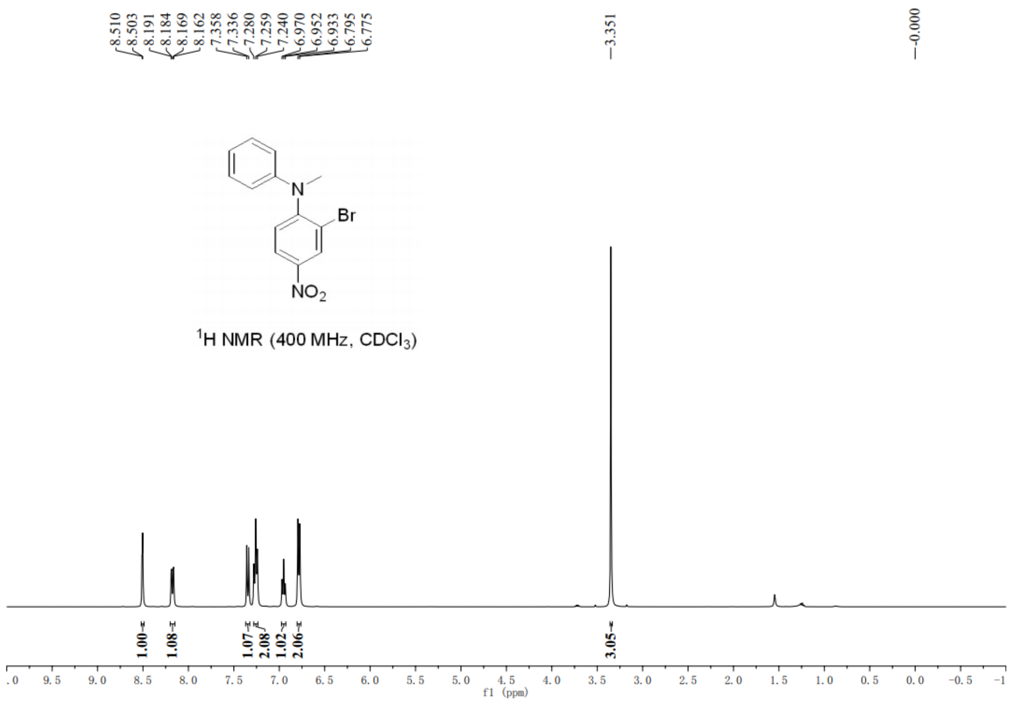

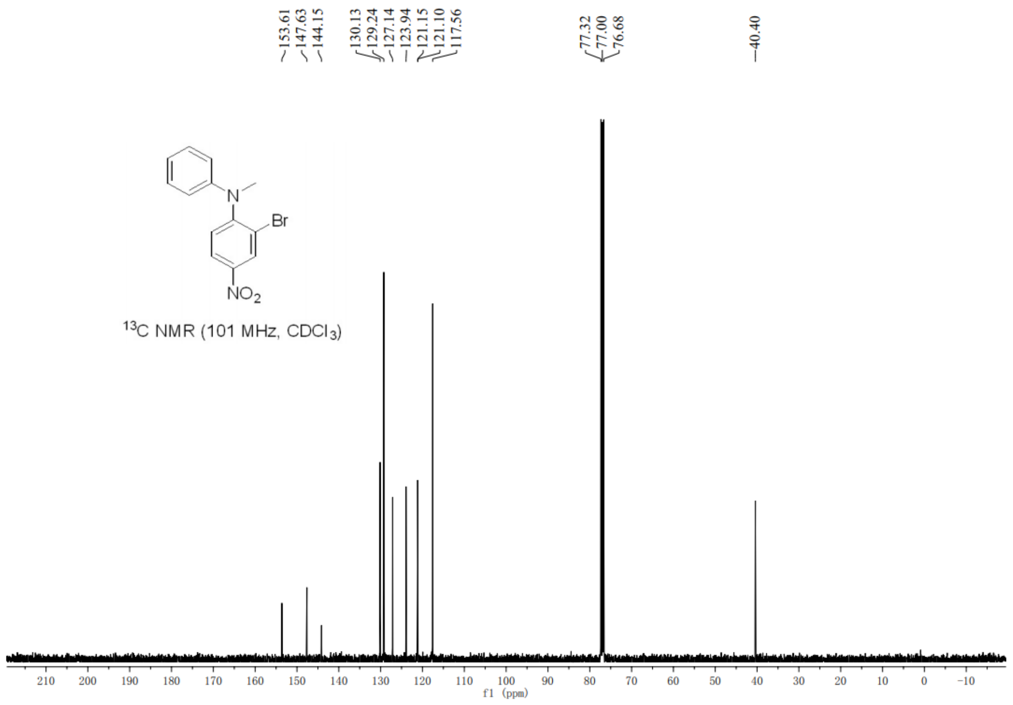

1-(3,4-dinitrophenyl)-1,2,3,4-tetrahydroquinoline (4a-10)

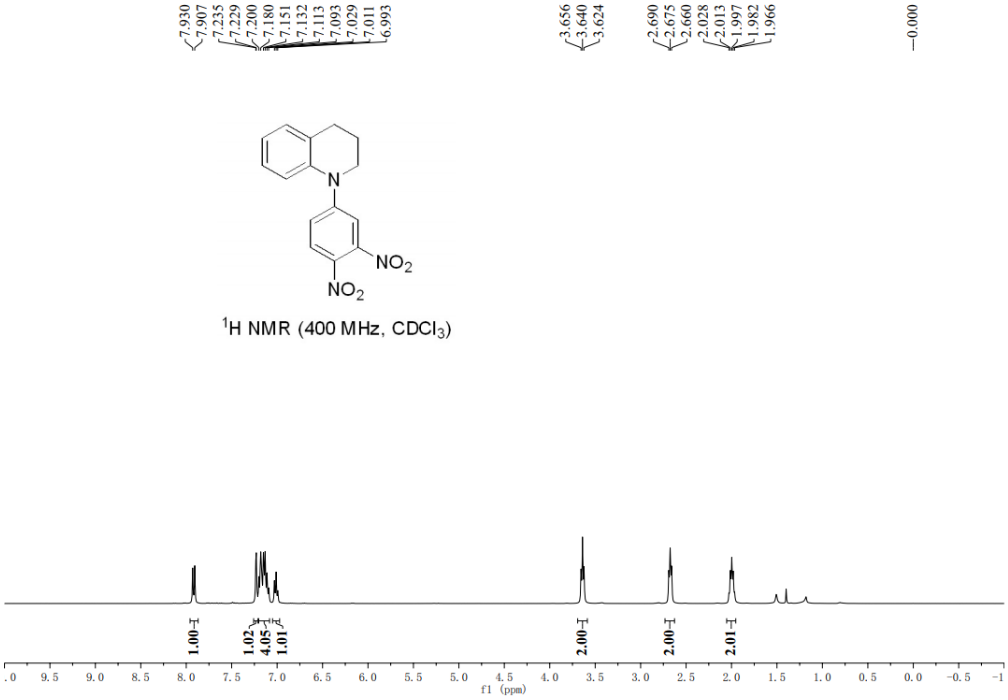

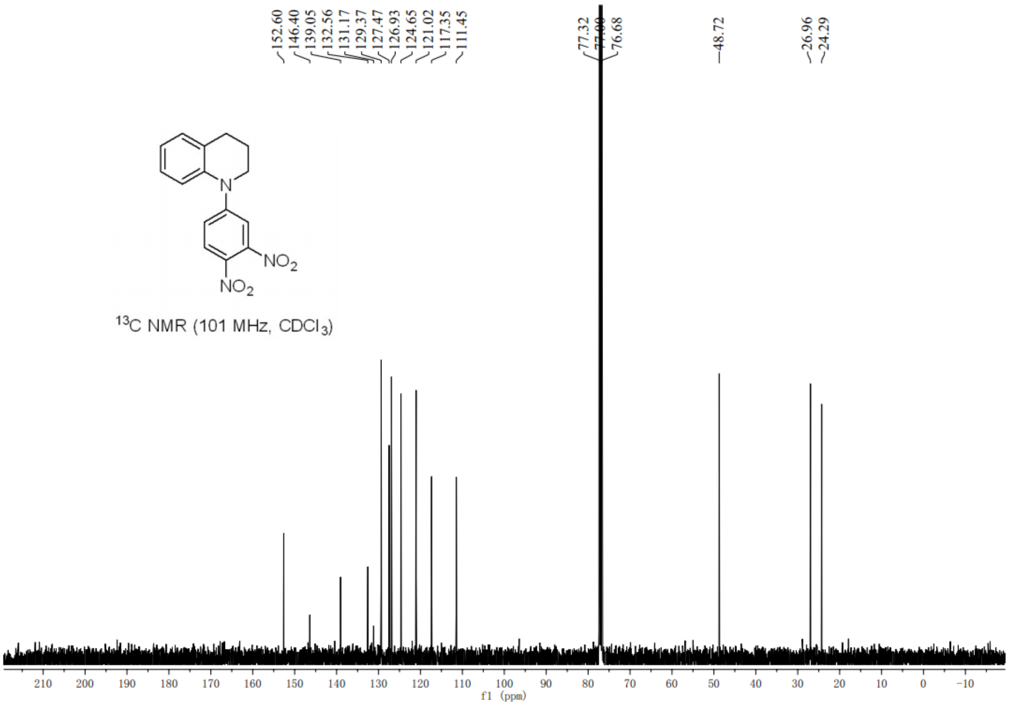

N-methyl-3,4-dinitro-N-phenylaniline (4c-10)

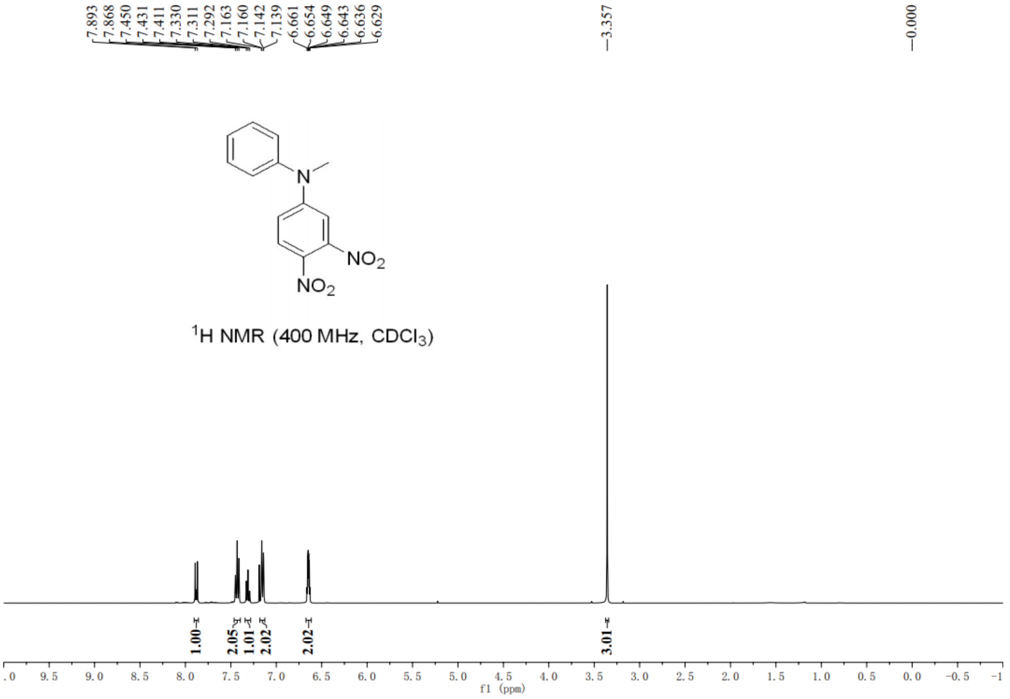

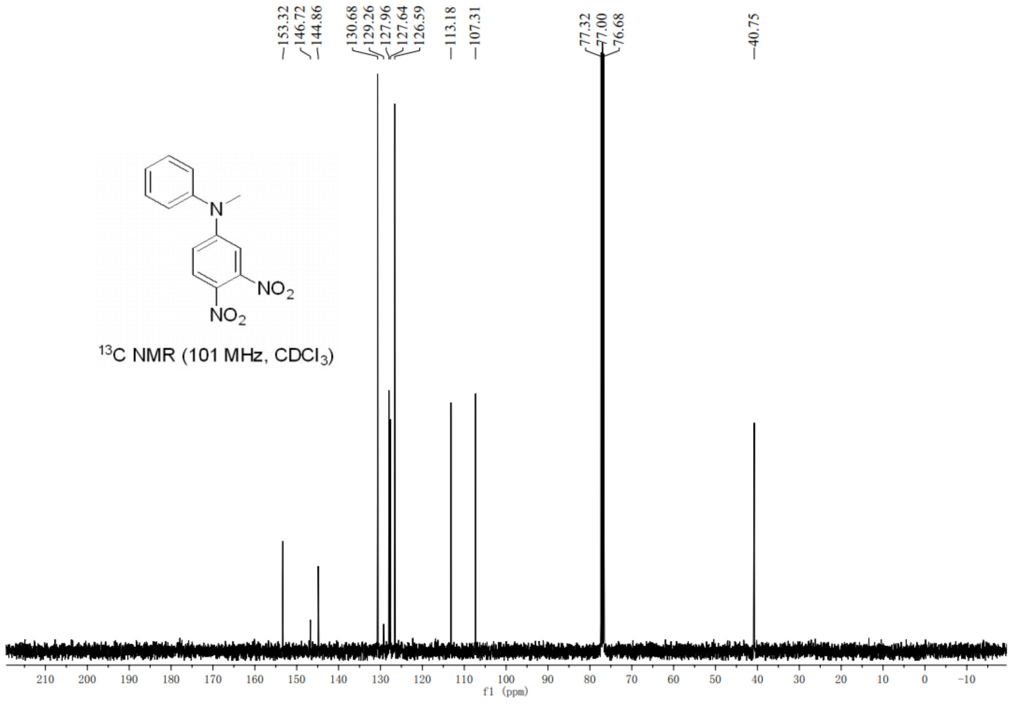

1-(2,6-difluoro-4-nitrophenyl)-1,2,3,4-tetrahydroquinoline (4a-11)

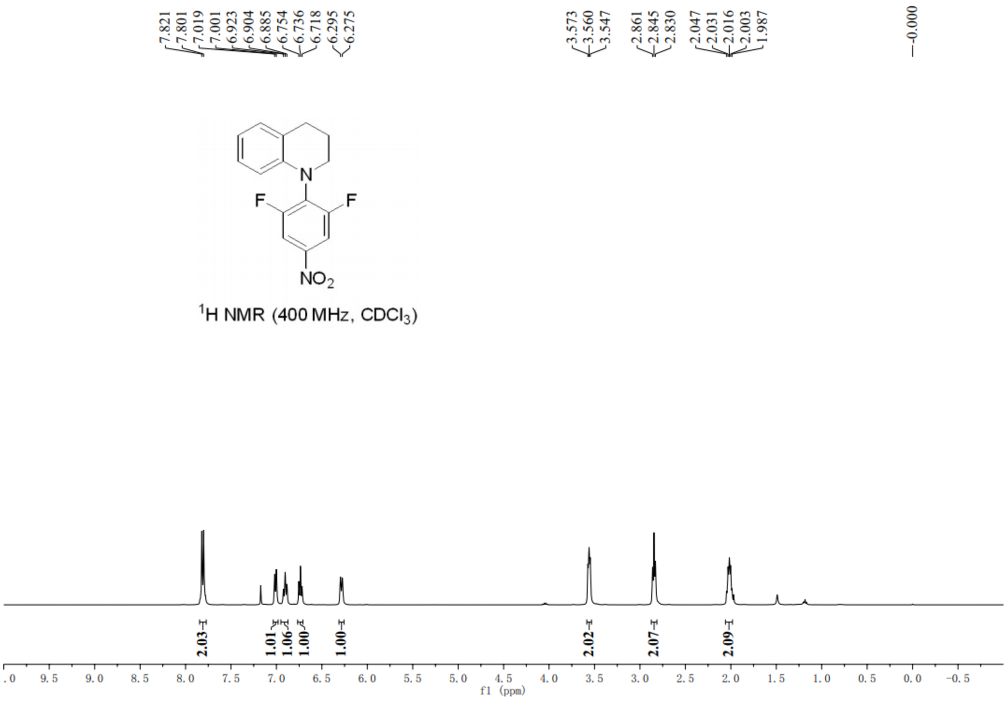

^{1}H NMR (400 MHz, $CDCl_3$)

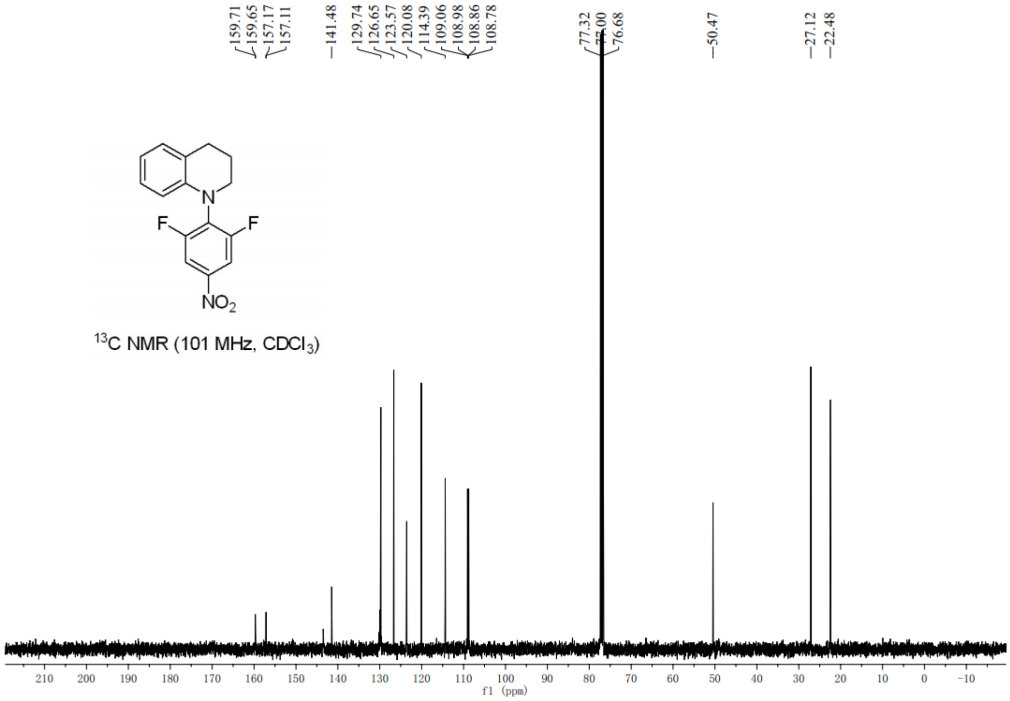

^{13}C NMR (101 MHz, $CDCl_3$)

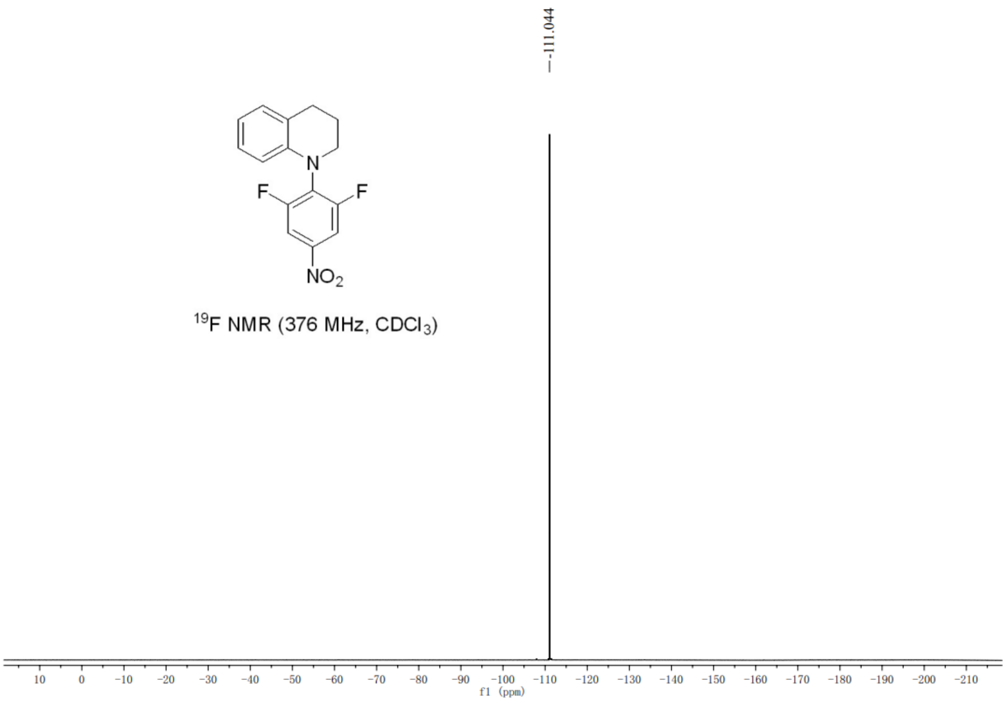

-111.044
F
F
N
NO_2
^{19}F NMR (376 MHz, $CDCl_3$)
10
0
-10
-20
-30
-40
-50
-60
-70
-80
-90
-100
-110
-120
-130
-140
-150
-160
-170
-180
-190
-200
-210
f1 (ppm)

1-(2,6-difluoro-4-nitrophenyl)indoline (4b-11)

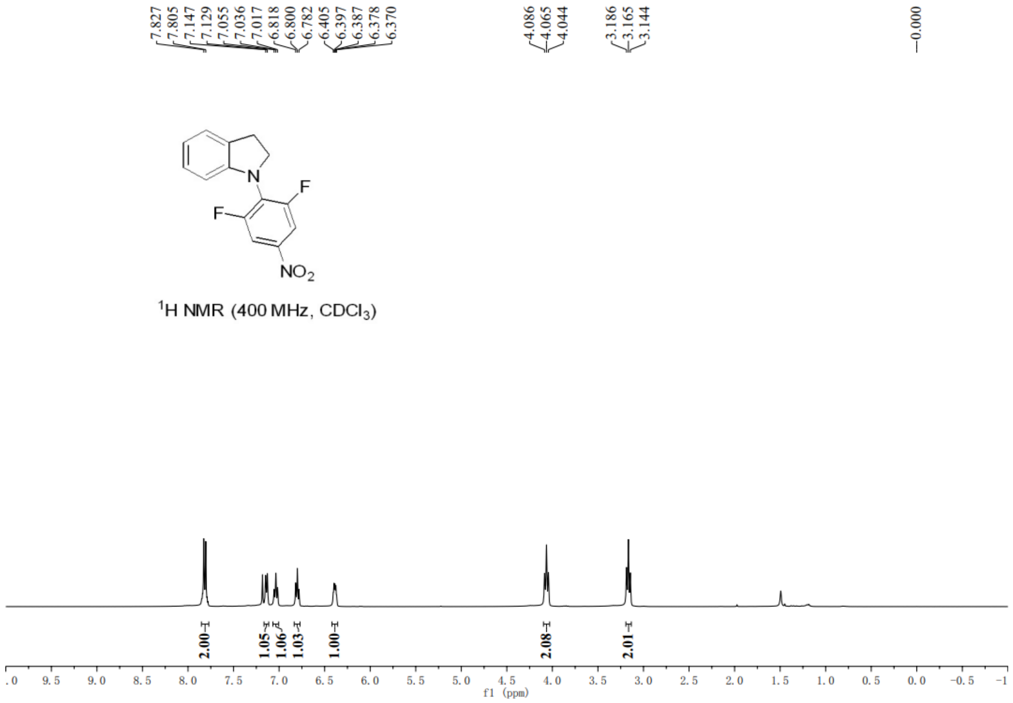

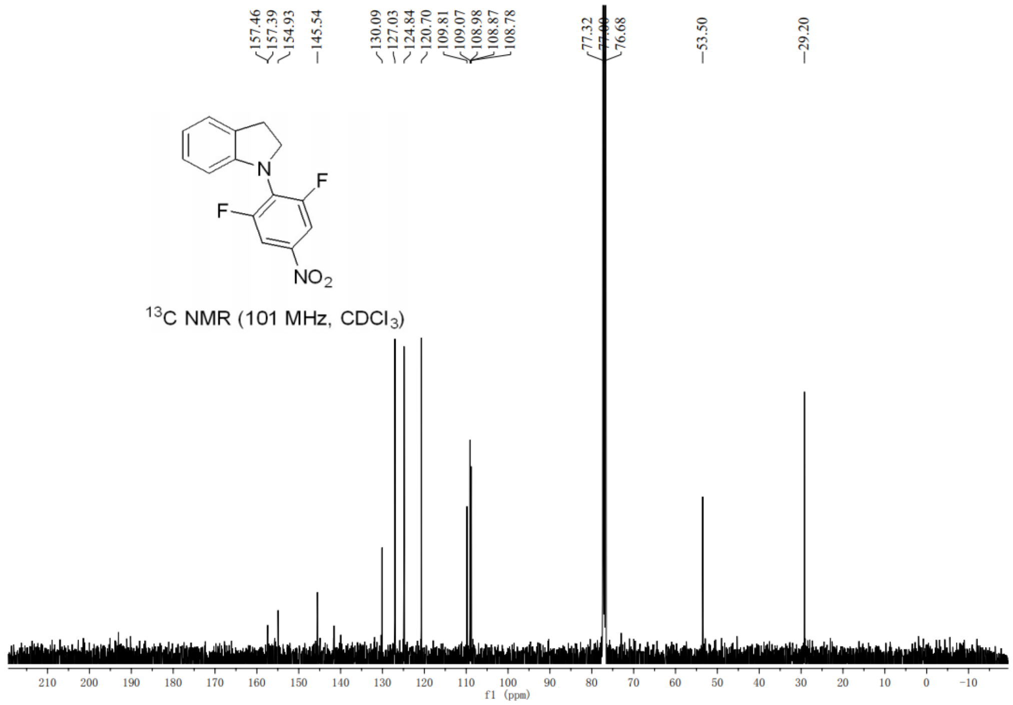

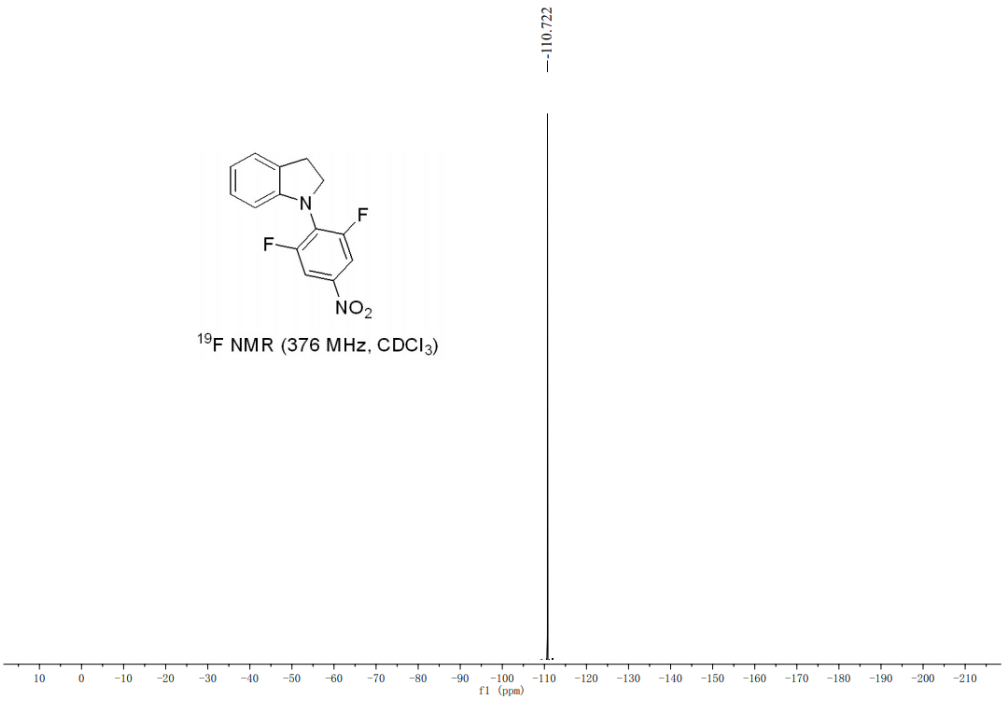
-110.722
F
F
N
NO2
19F NMR (376 MHz, CDCl3)
10 0 -10 -20 -30 -40 -50 -60 -70 -80 -90 -100 -110 -120 -130 -140 -150 -160 -170 -180 -190 -200 -210
f1 (ppm)

2,6-difluoro-*N*-methyl-4-nitro-*N*-phenylaniline (4c-11)

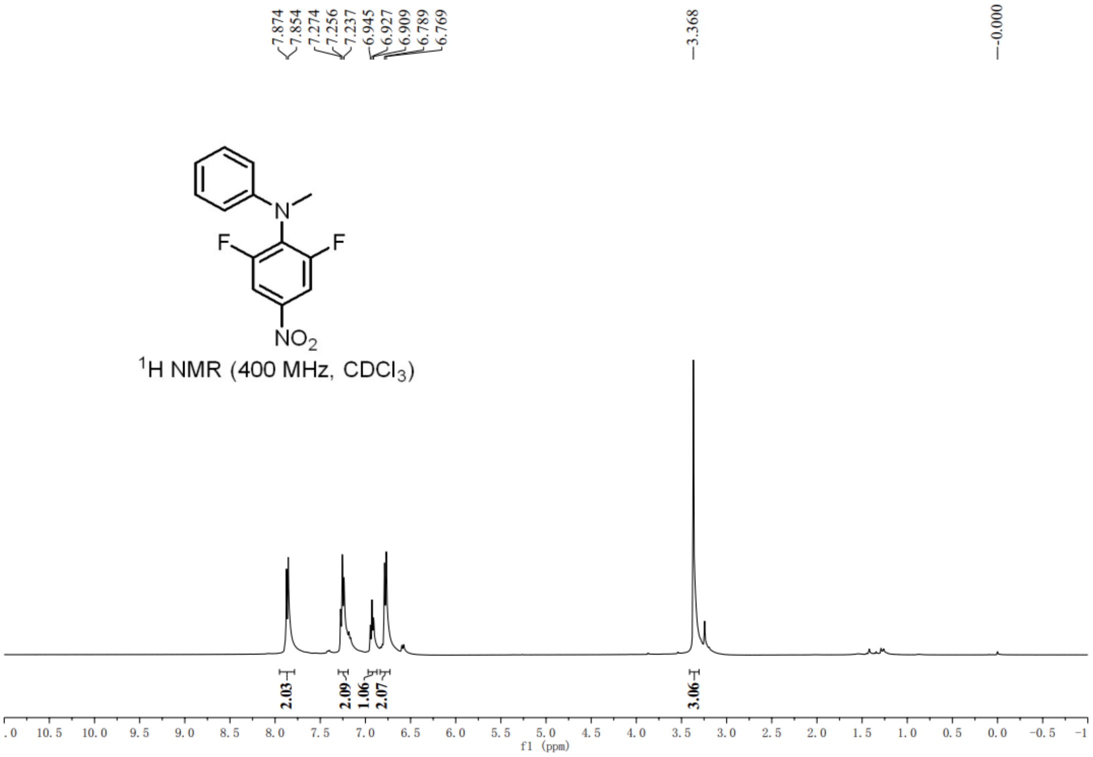

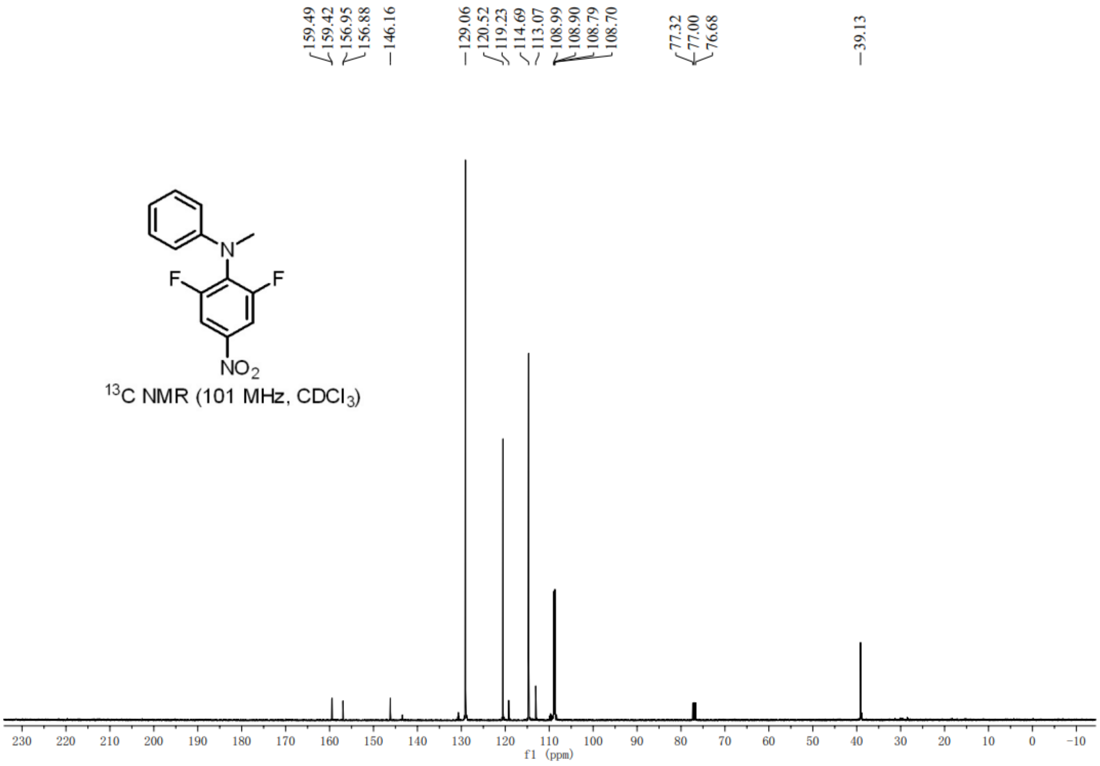

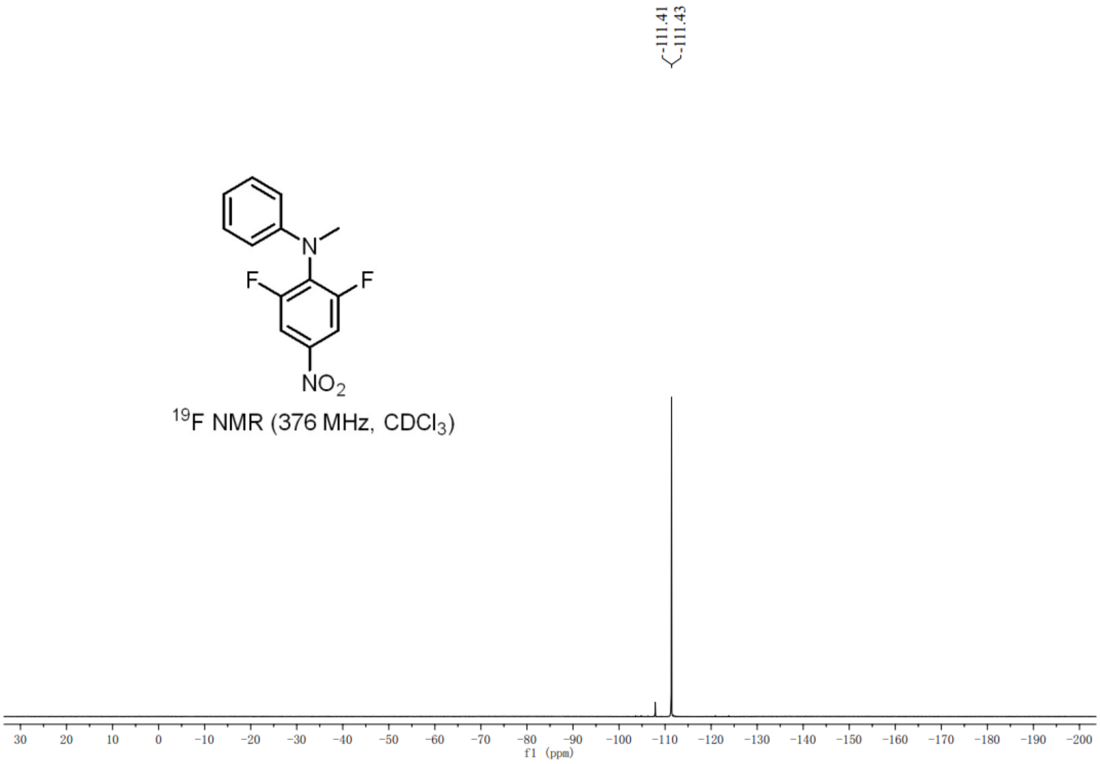
-111.41
-111.43
N
F
F
NO_2
^{19}F NMR (376 MHz, $CDCl_3$)
30 20 10 0 −10 −20 −30 −40 −50 −60 −70 −80 −90 −100 −110 −120 −130 −140 −150 −160 −170 −180 −190 −200
f1 (ppm)

1-(2,6-dichloro-4-nitrophenyl)-1,2,3,4-tetrahydroquinoline (4a-12)

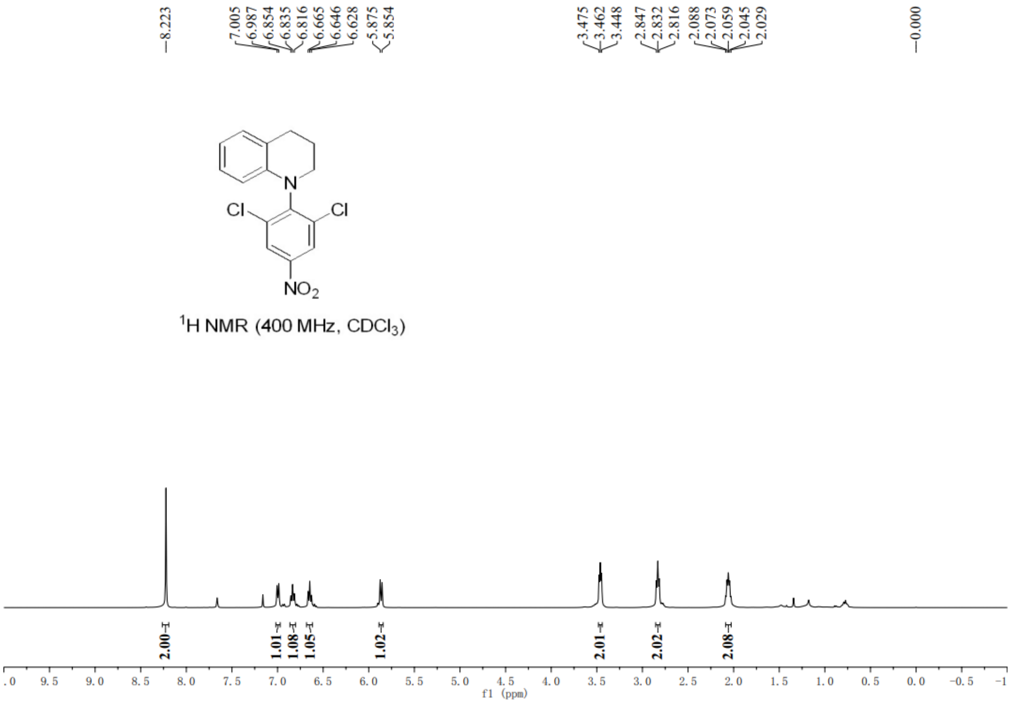

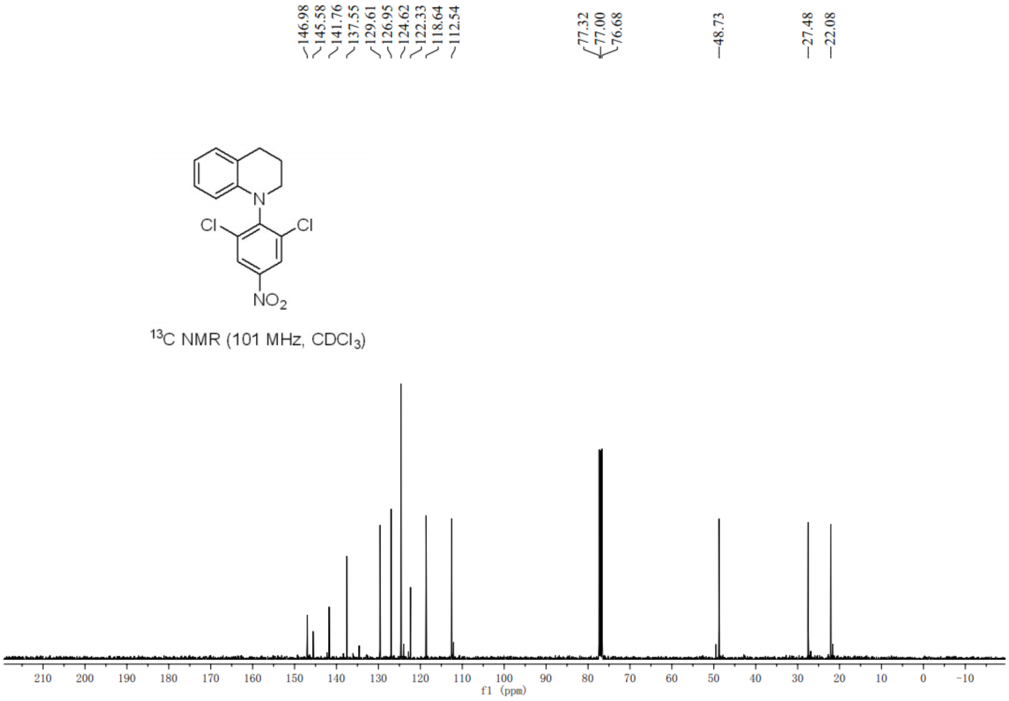

1-(2,6-dichloro-4-nitrophenyl)indoline (4b-12)

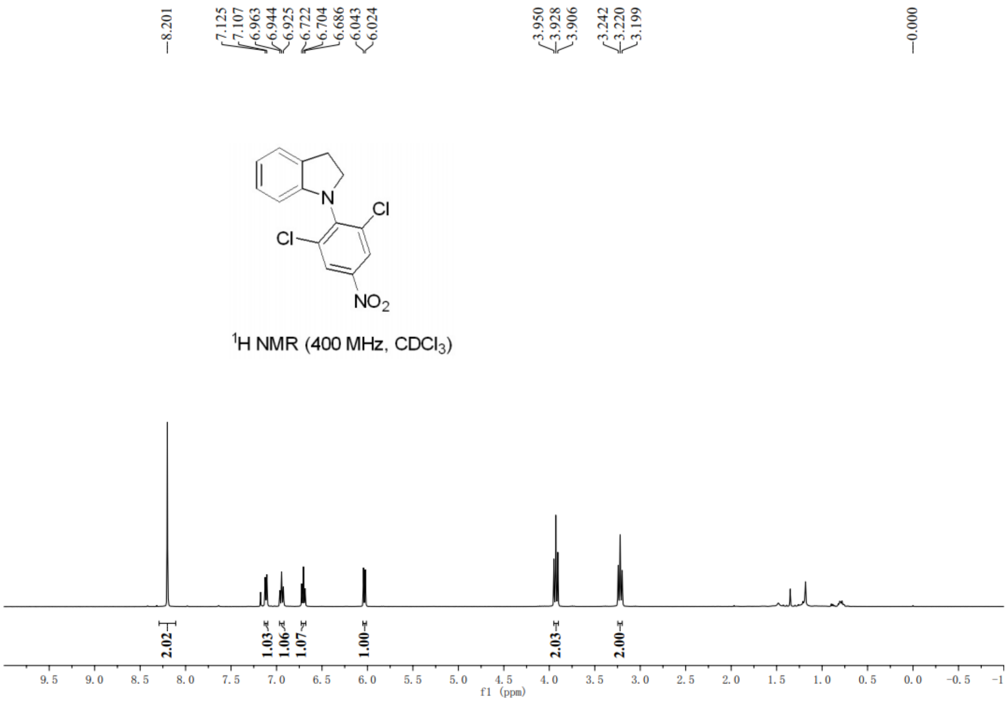

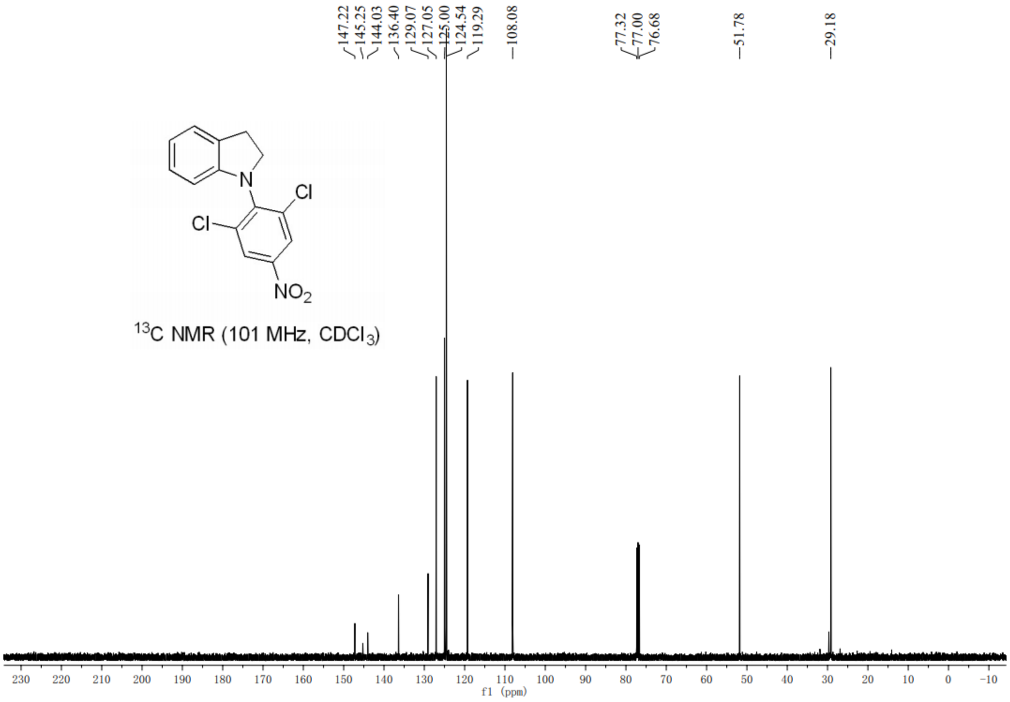

2,6-dichloro-*N*-methyl-4-nitro-*N*-phenylaniline (4c-12)

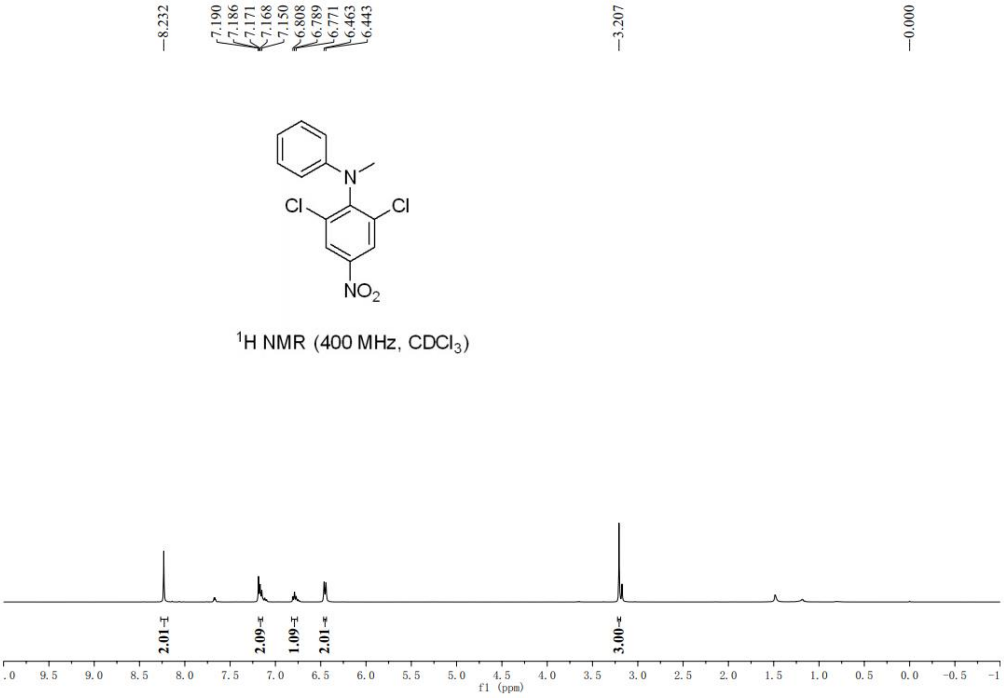

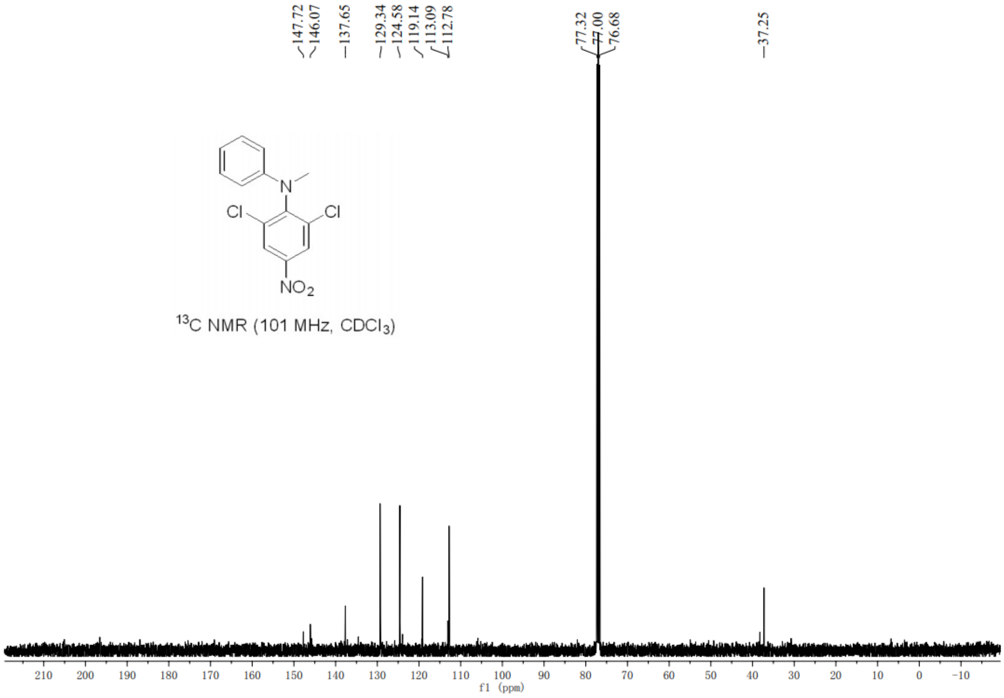

1-(2,6-dibromo-4-nitrophenyl)-1,2,3,4-tetrahydroquinoline (4a-13)

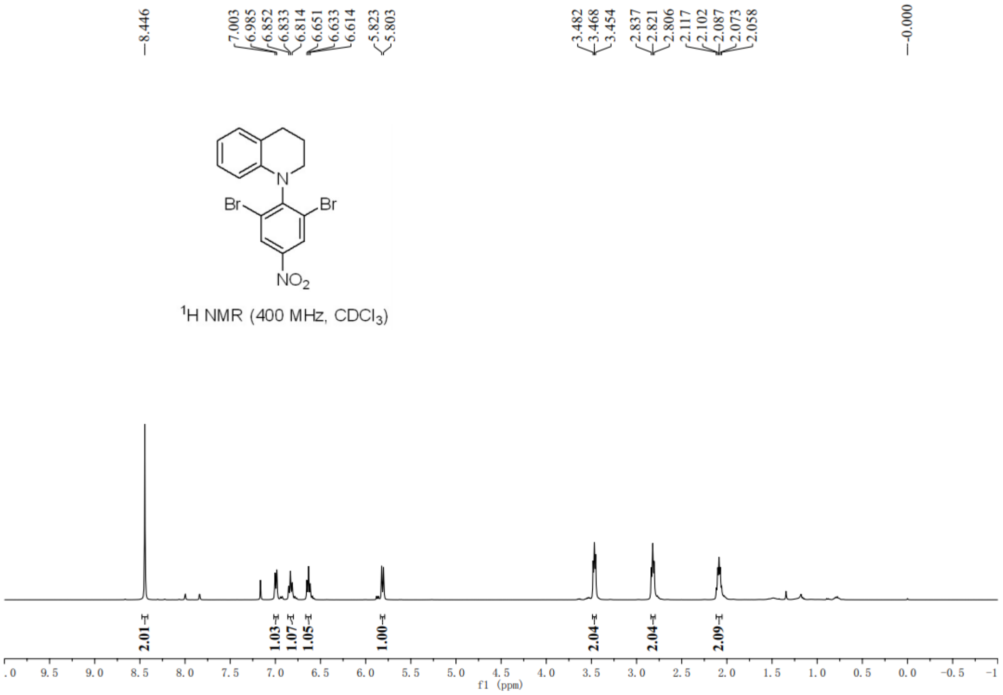

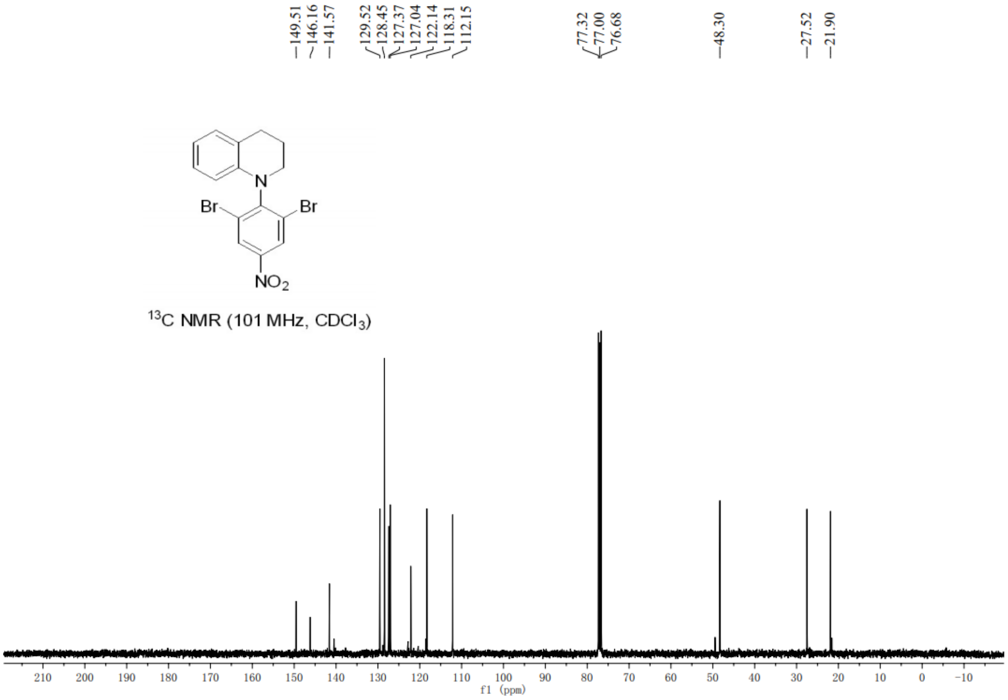

1-(2,6-dibromo-4-nitrophenyl) indoline (4b-13)

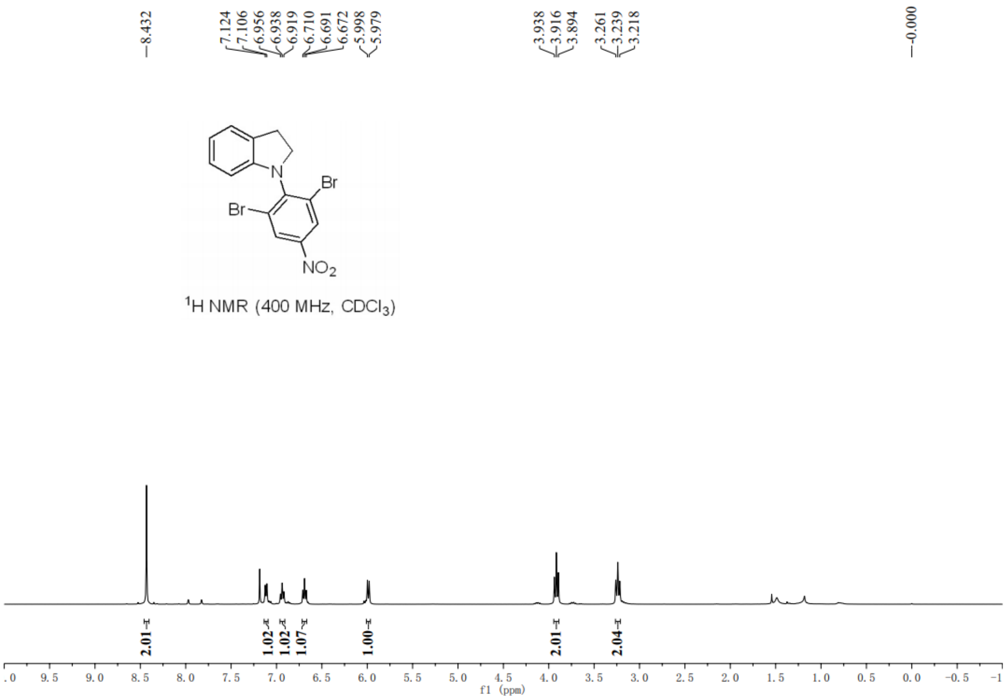

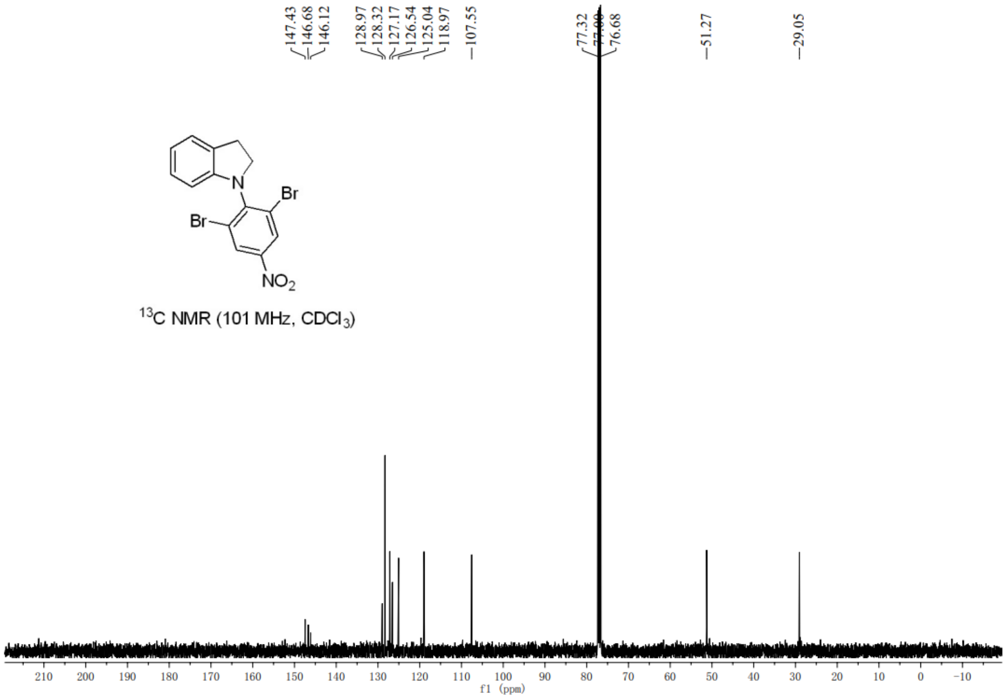

2,6-dibromo-N-methyl-4-nitro-N-phenylaniline (4c-13)

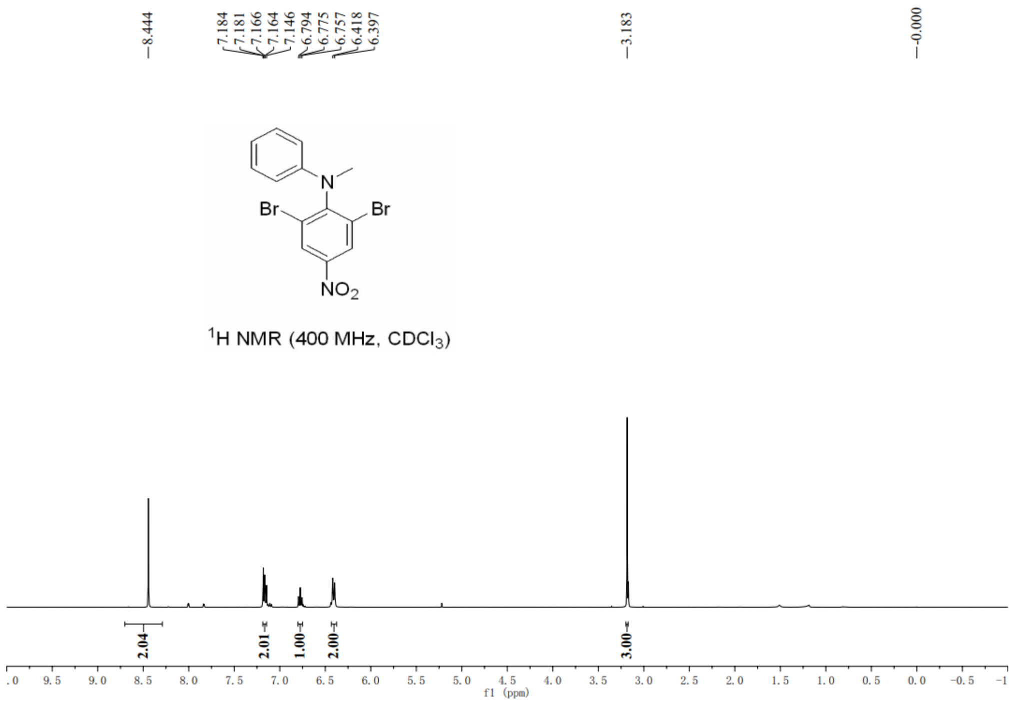

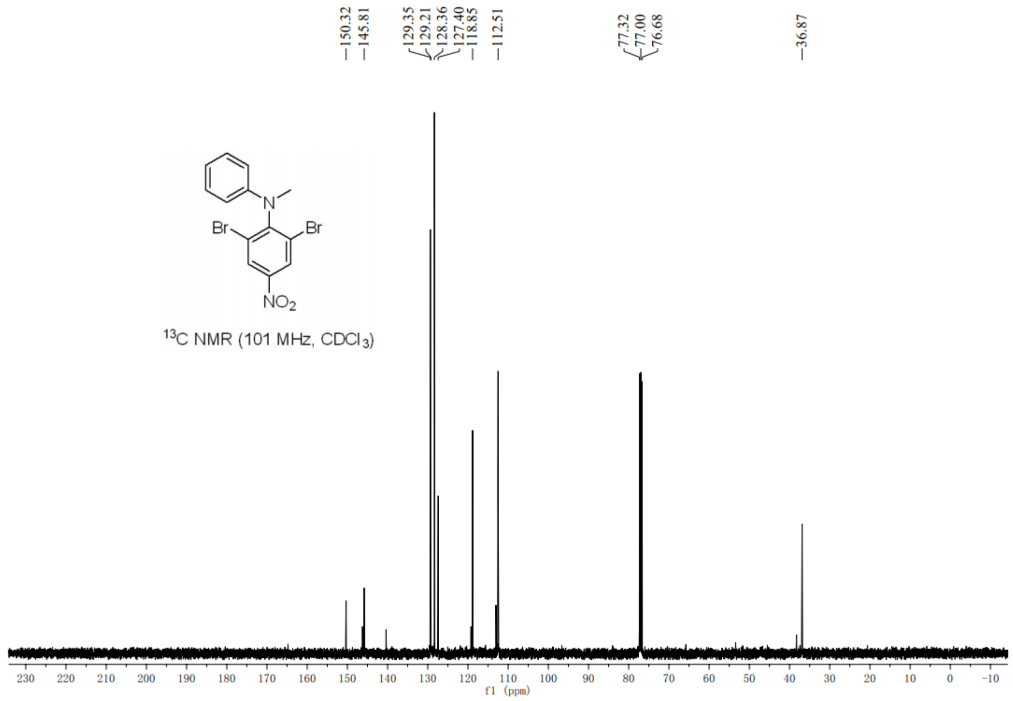

1-(2-bromo-6-chloro-4-nitrophenyl)-1,2,3,4-tetrahydroquinoline (4a-14)

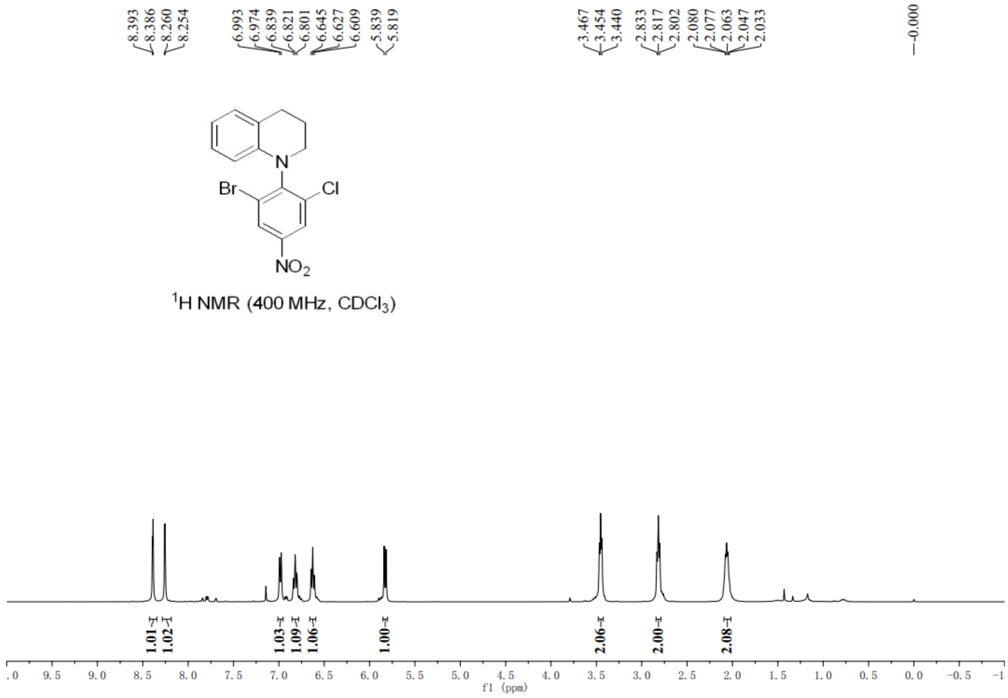

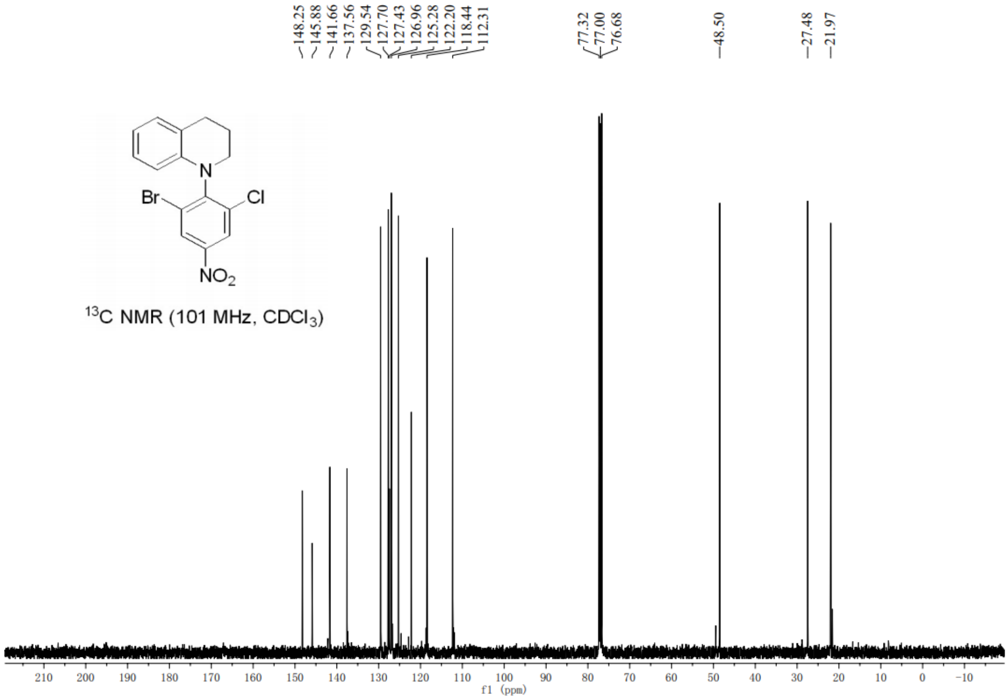

1-(2-bromo-6-chloro-4-nitrophenyl) indoline (4b-14)

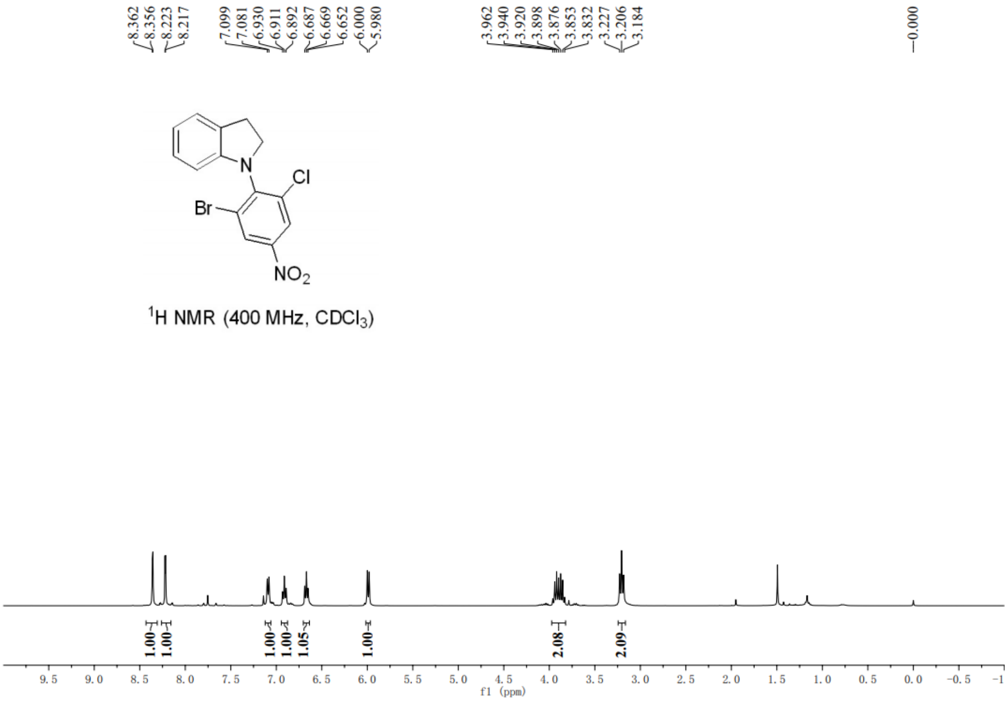

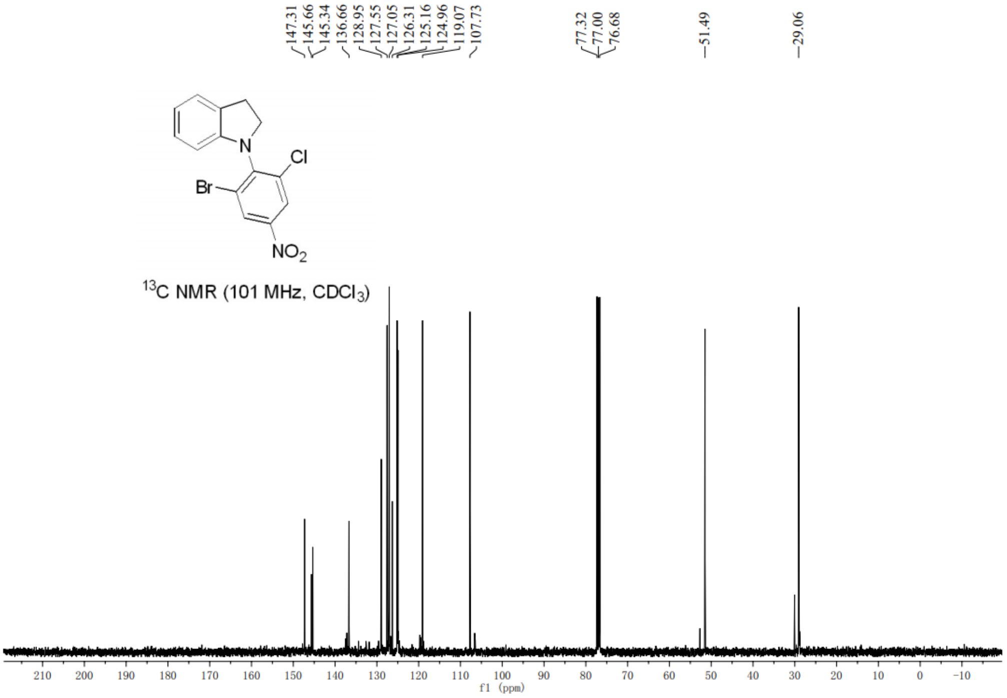

2-bromo-6-chloro-*N*-methyl-4-nitro-*N*-phenylaniline (4c-14)

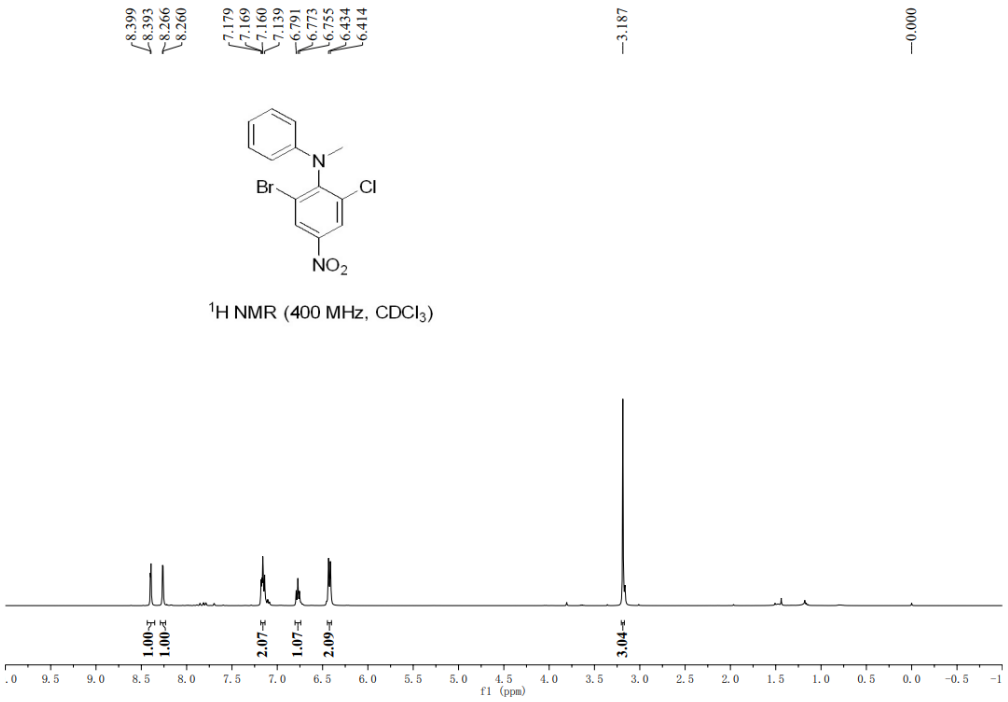

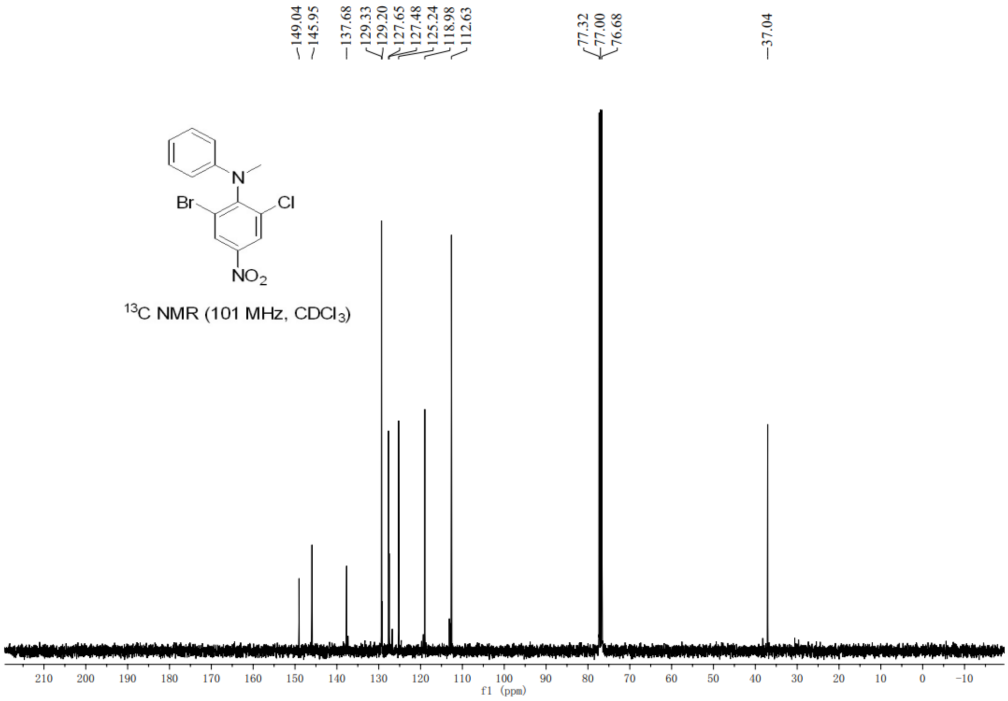

1-(4-nitro-3-(trifluoromethyl)phenyl)-1,2,3,4-tetrahydroquinoline (4a-15)

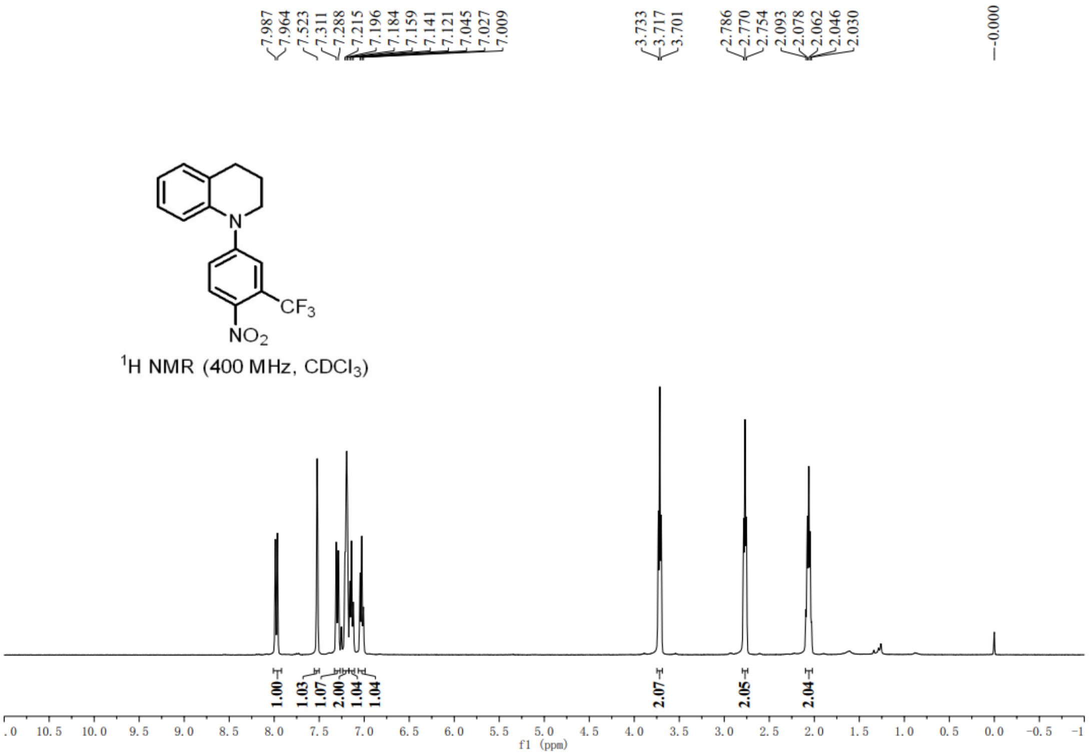

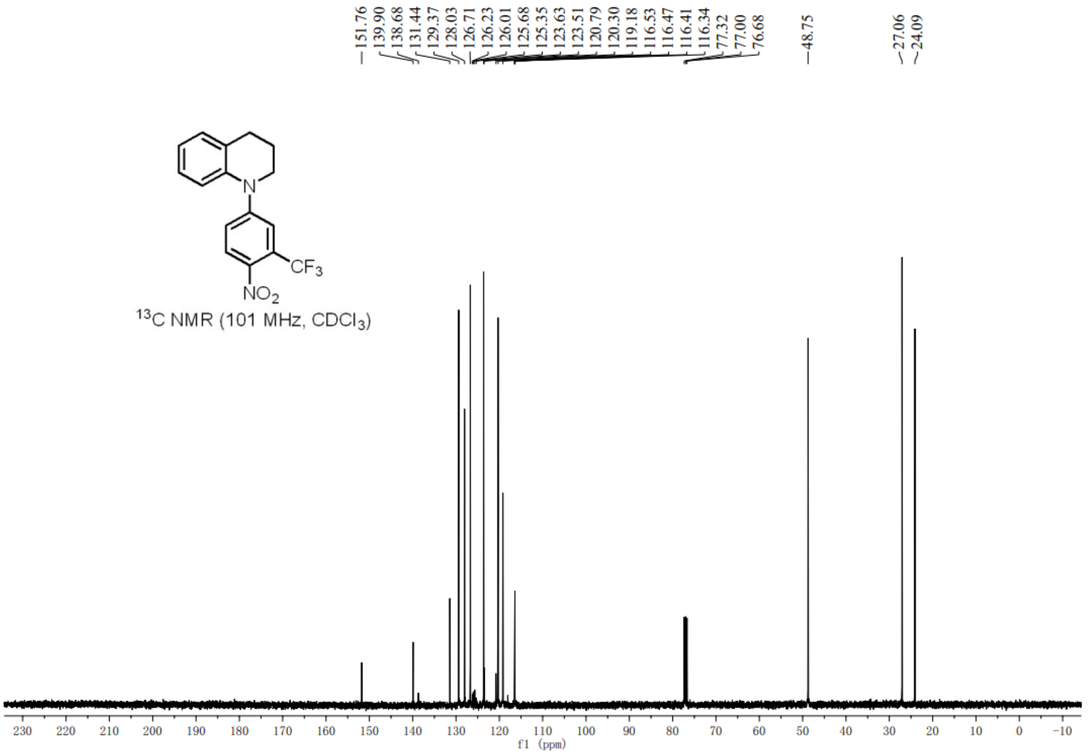

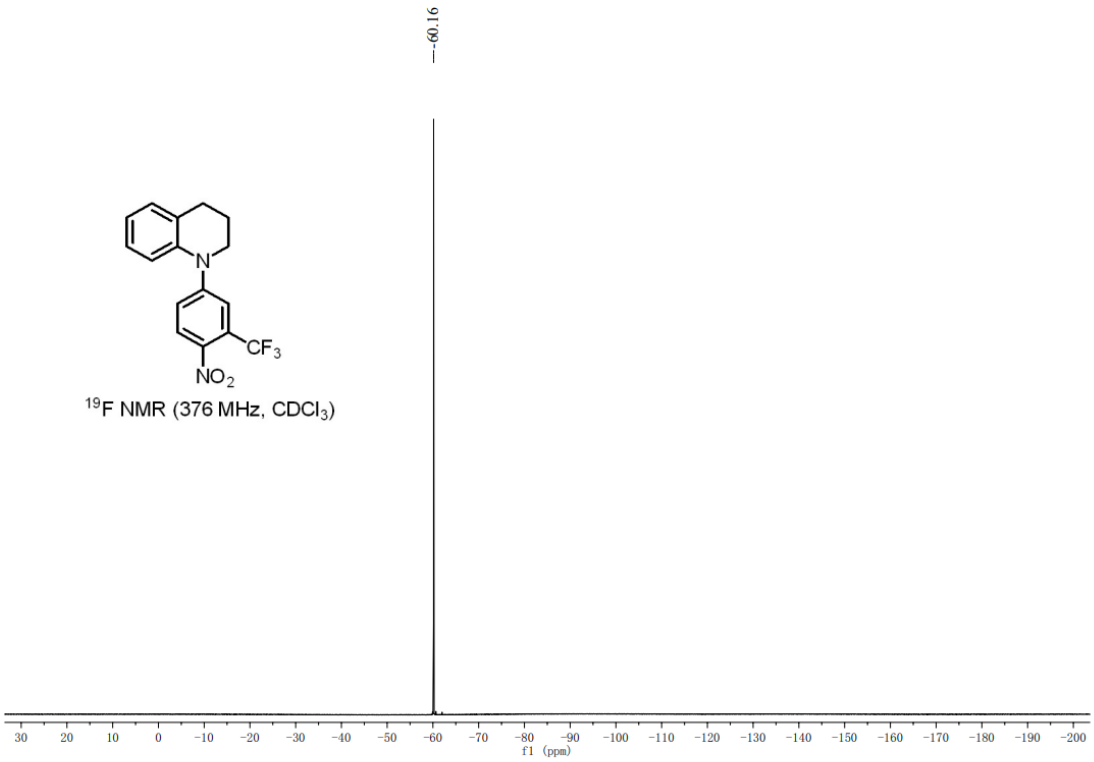

-60.16
CF3
NO2
N
19F NMR (376 MHz, CDCl3)
30 20 10 0 -10 -20 -30 -40 -50 -60 -70 -80 -90 -100 -110 -120 -130 -140 -150 -160 -170 -180 -190 -200
f1 (ppm)

1-(2-(methylthio)-4-nitrophenyl)-1,2,3,4-tetrahydroquinoline (4a-16)

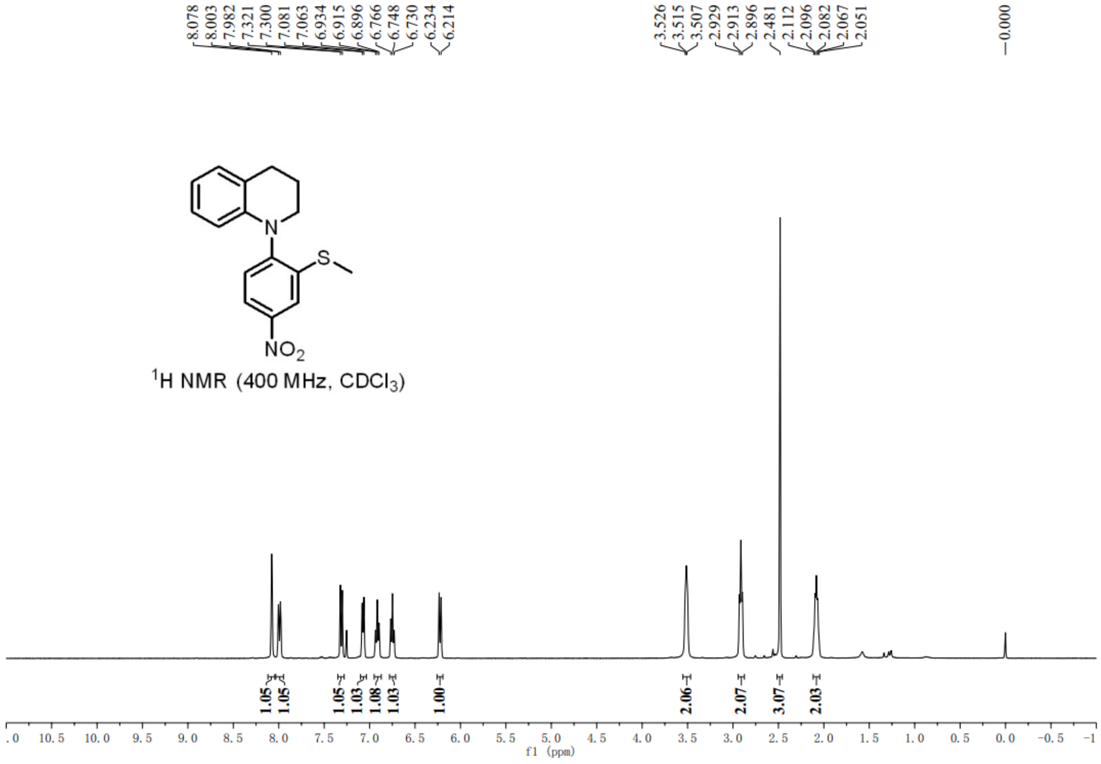

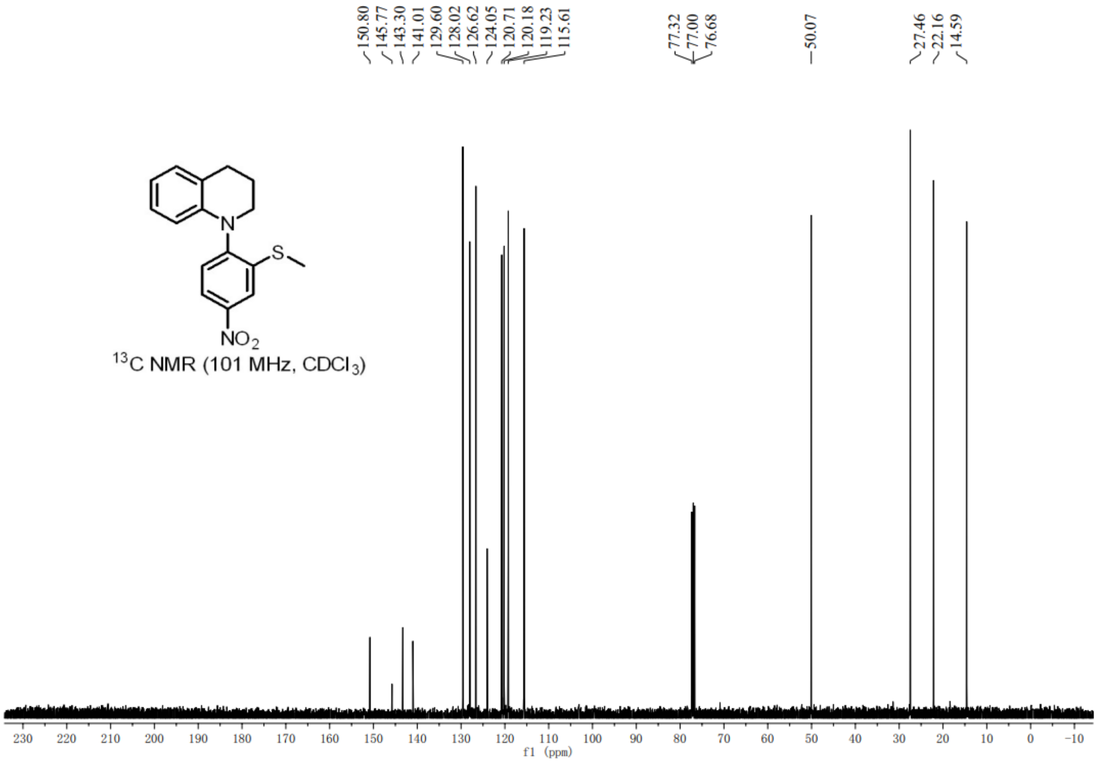

2-(3,4-dihydroquinolin-1(2H)-yl)-5-nitrobenzamide (4a-17)

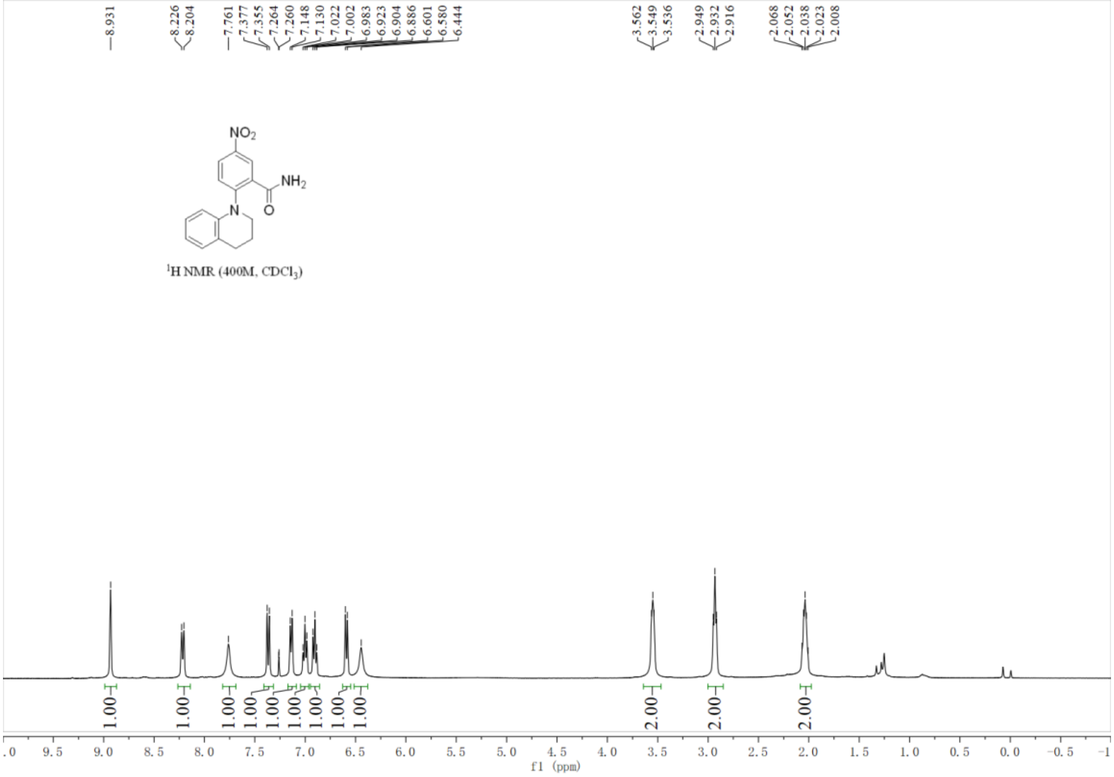

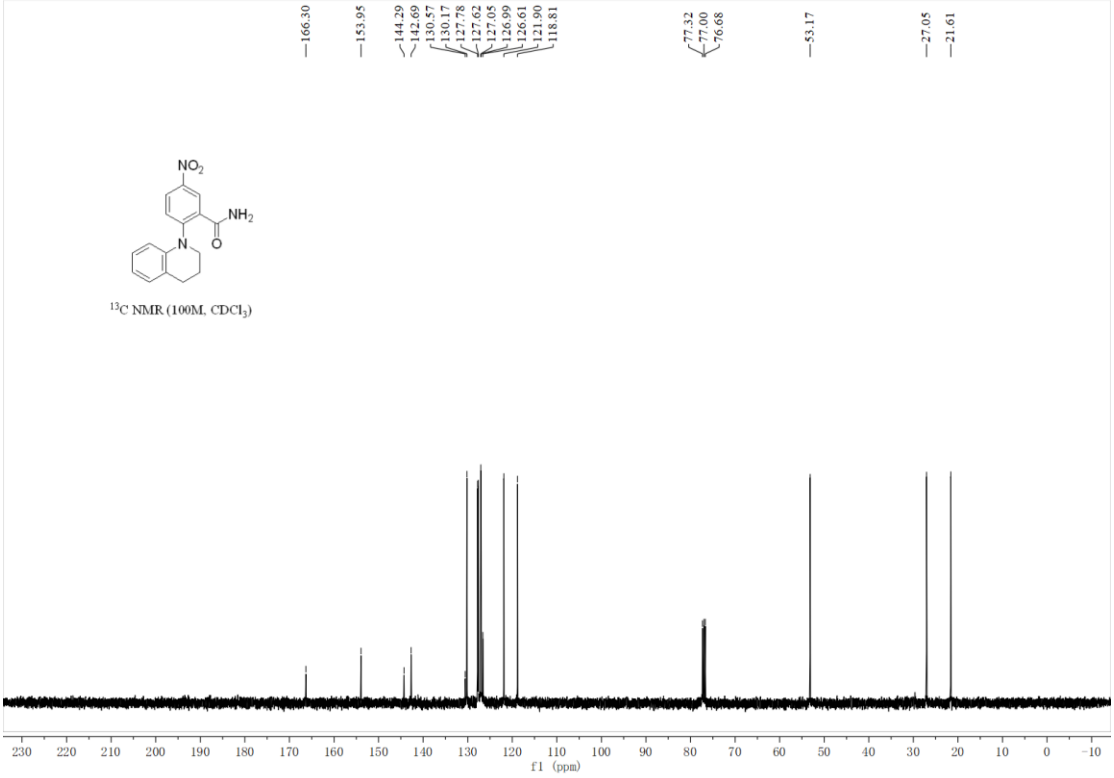

methyl 2-(3,4-dihydroquinolin-1(2*H*)-yl)-5-nitrobenzoate (4a-18)

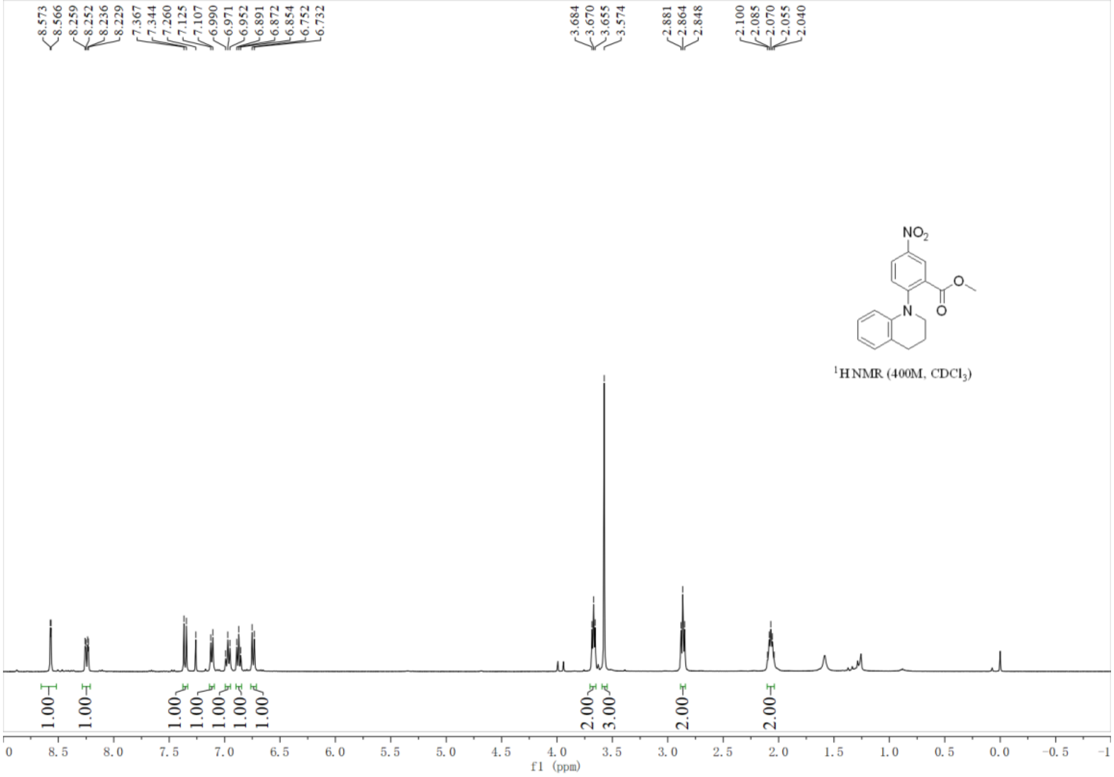

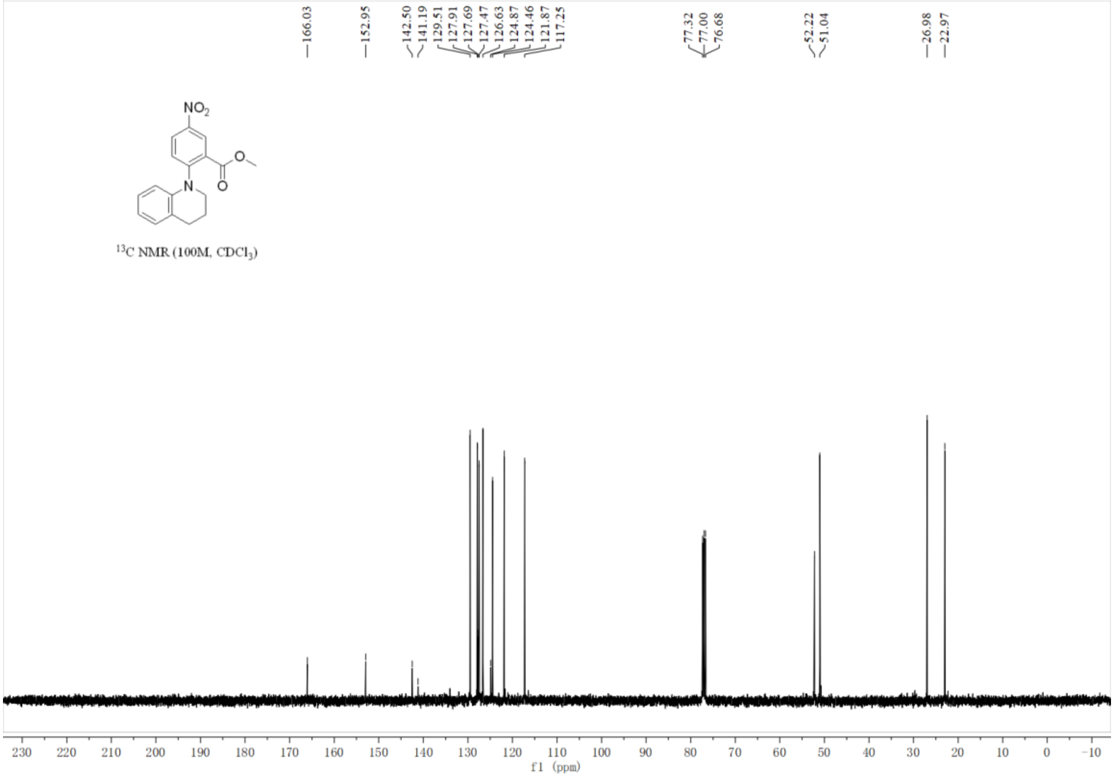

5-(3,4-dihydroquinolin-1(2H)-yl)-2-nitrophenol (4a-20)

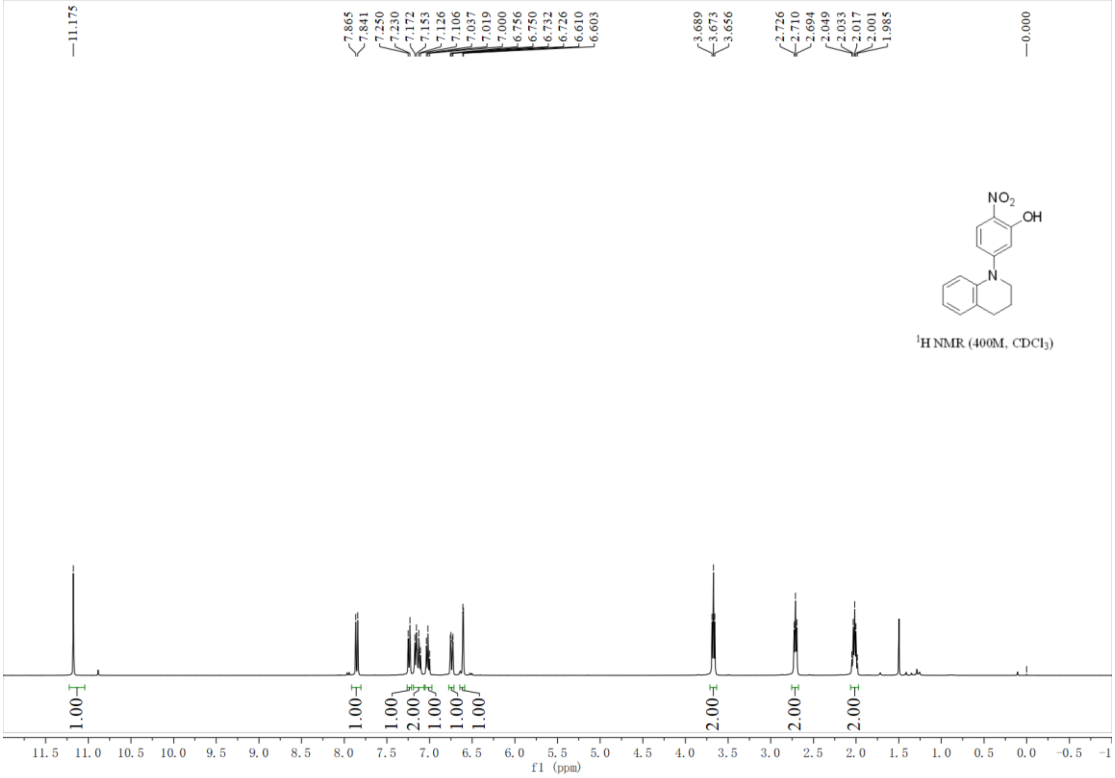

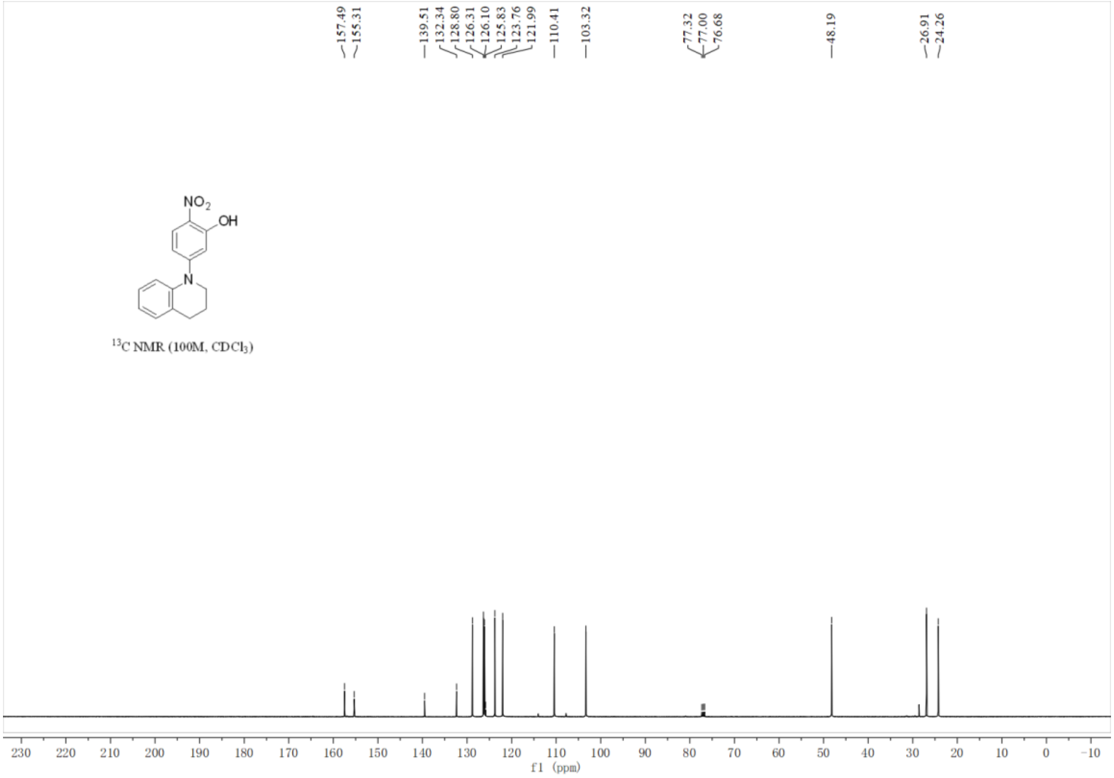

N,N-diphenyl-[1,1'-biphenyl]-4-amine (3c-10a)

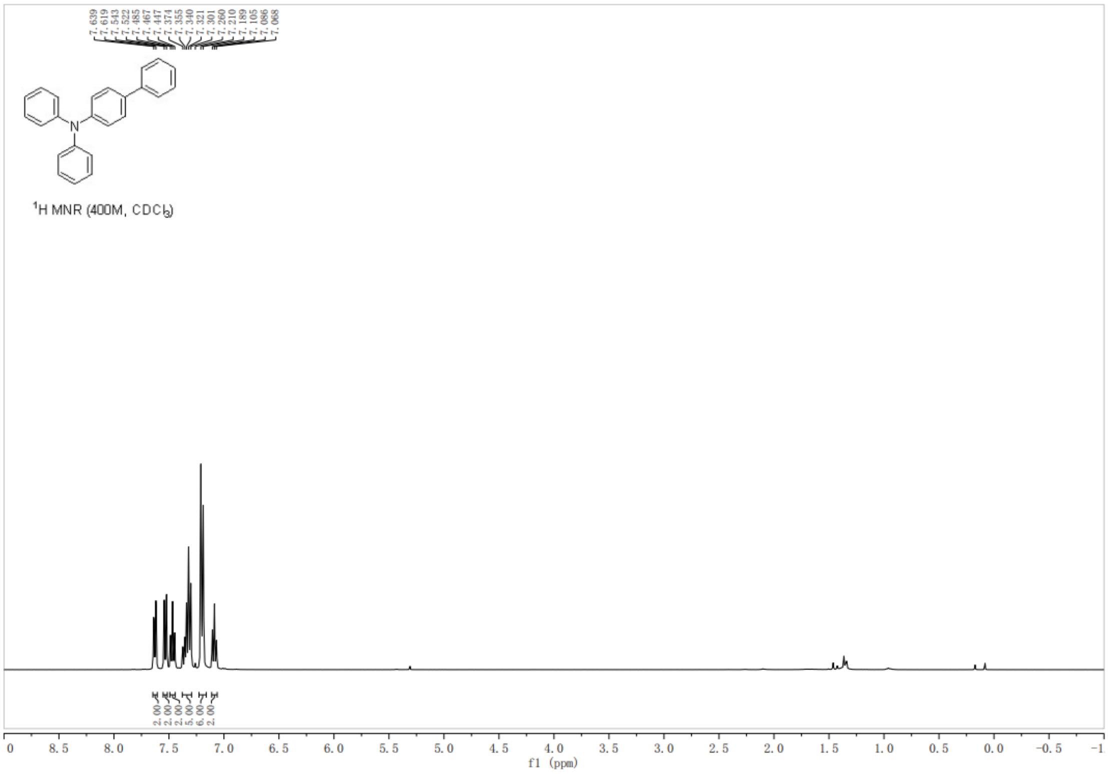

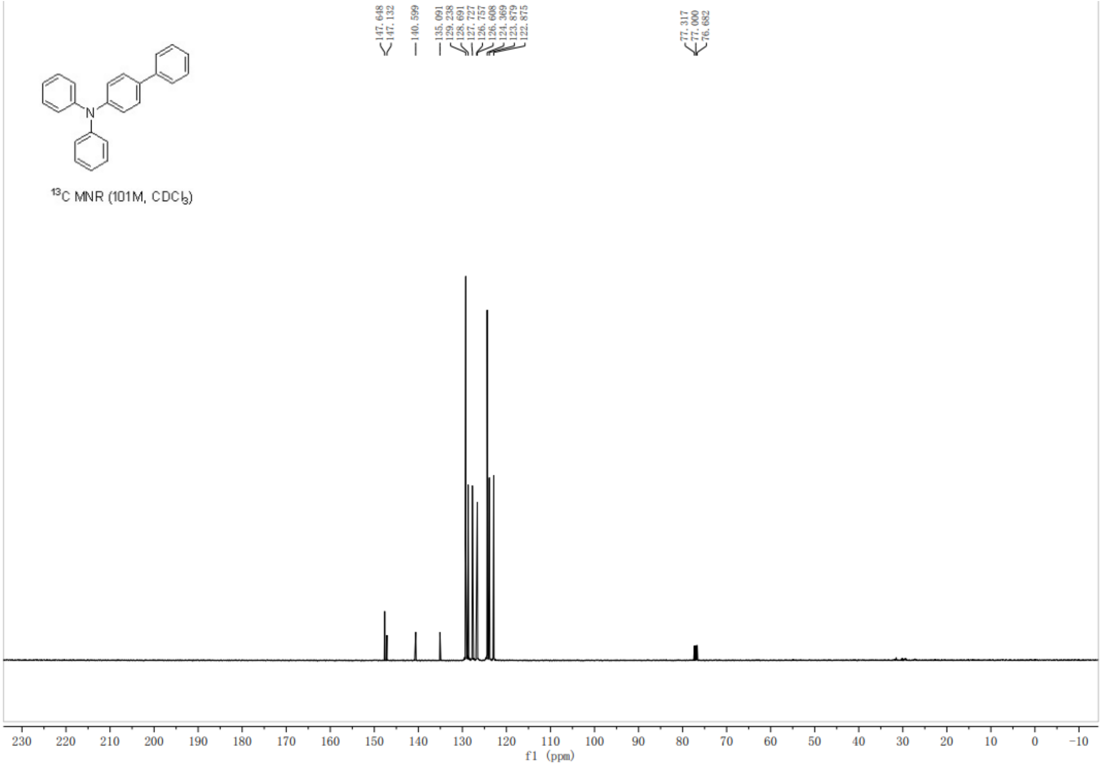

***N*[1],*N*[1]-diphenylbenzene-1,4-diamine (3c-10b)**

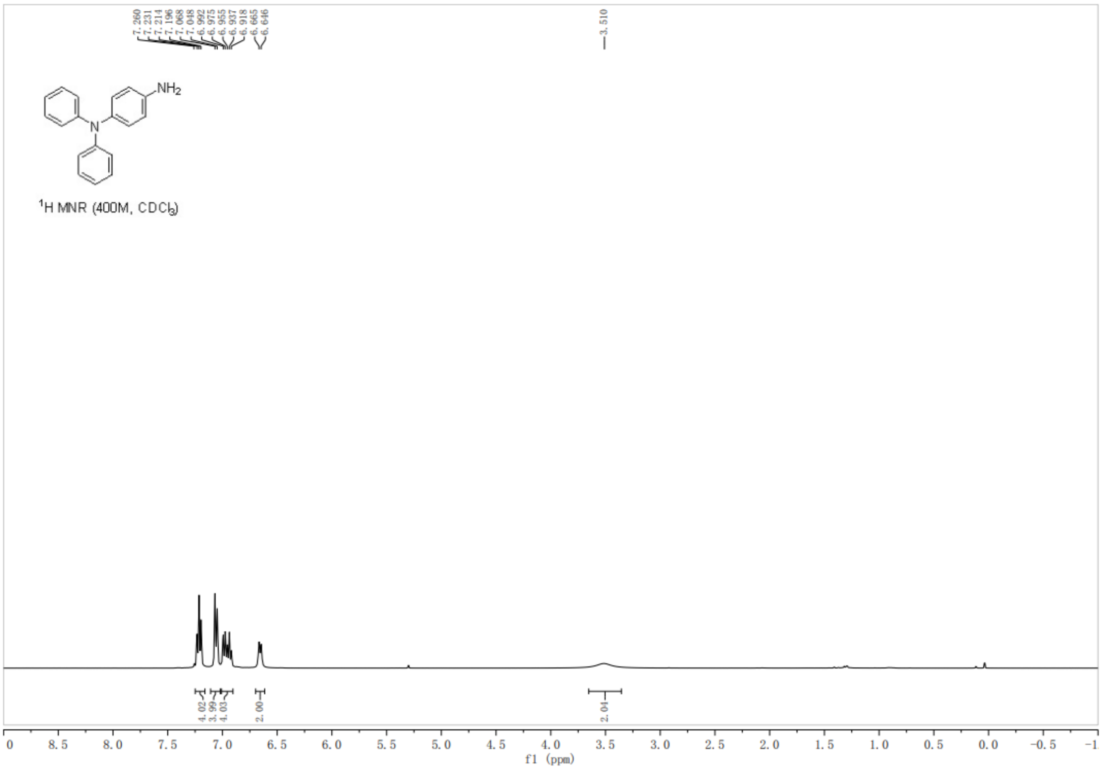

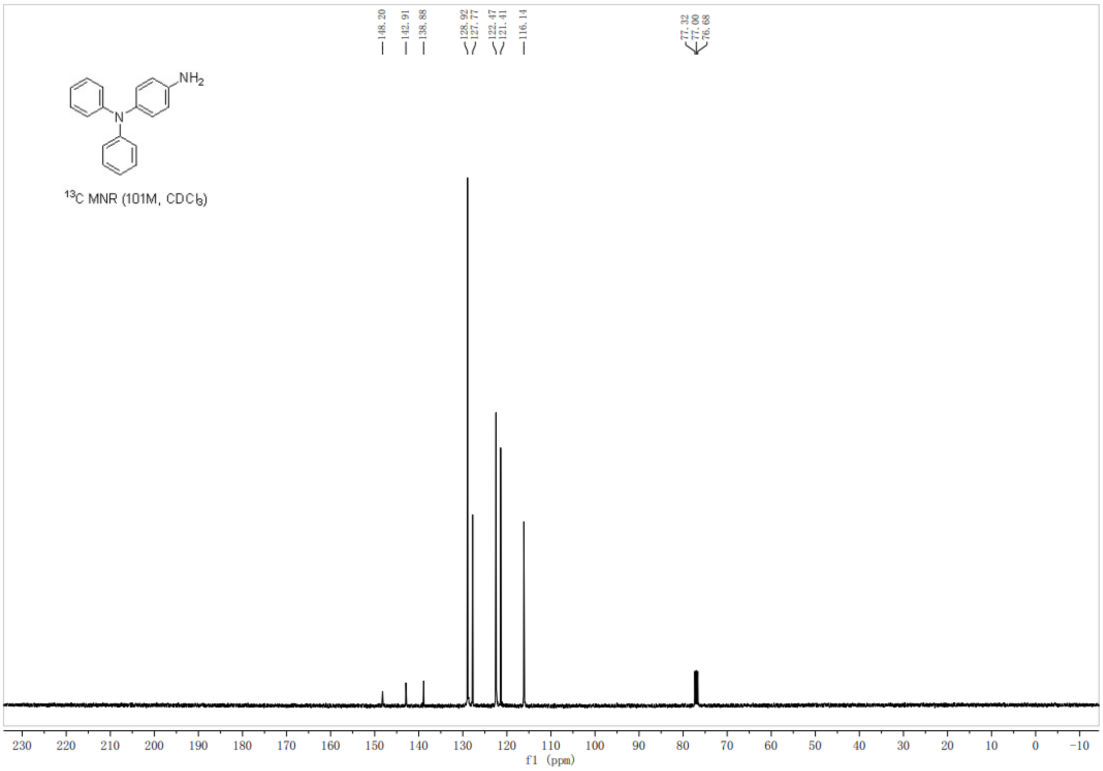

Triphenylamine (3c-10c)

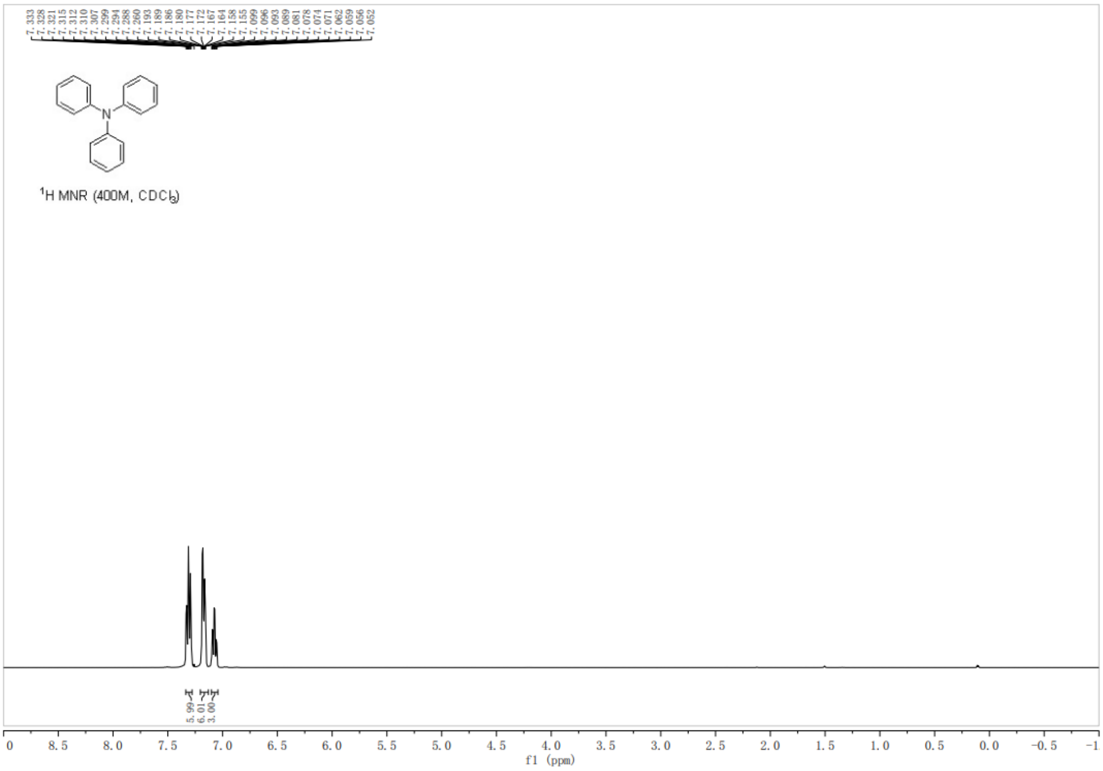

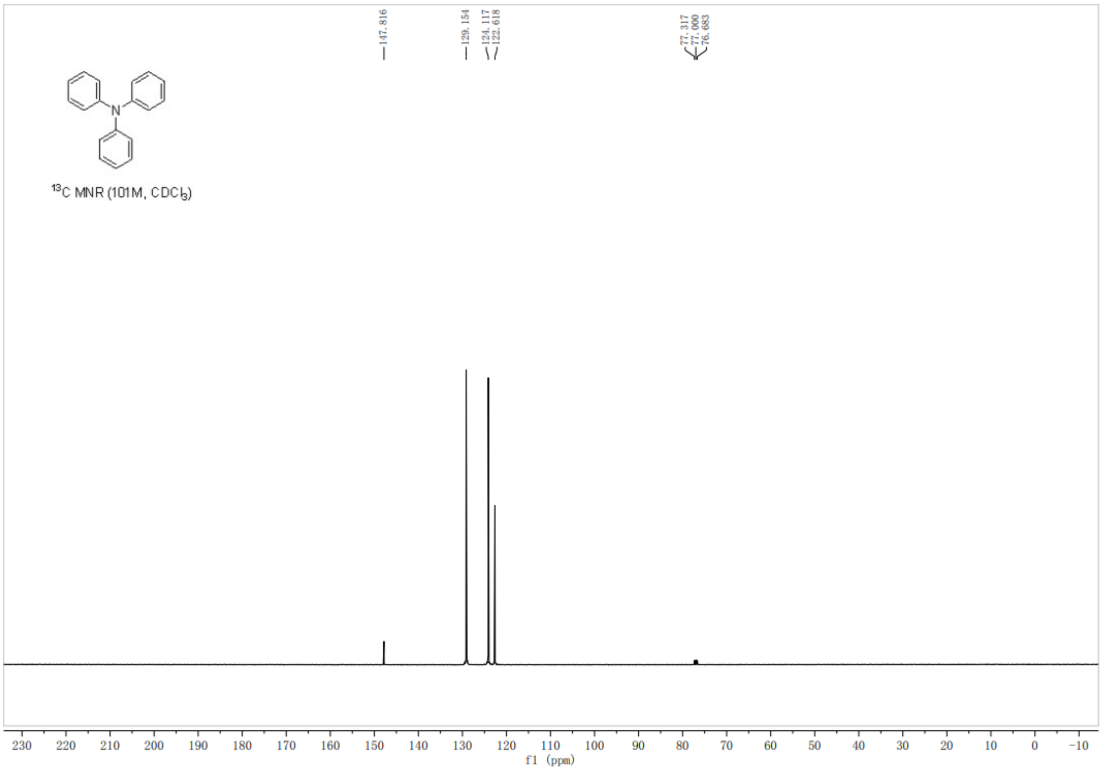

11. Supplementary References:

1. Mash, E. A.; Aavula, B. R., Synthesis of 7-Alkoxyquinolines, Coumarins, and Resorufins. *Synth. Commun*. **2000**, *30*, 367-375.

2. Mayer, R. T.; Netter, K. J.; Heubel, F.; Hahnemann, B.; Buchheister, A.; Mayer, G. K.; Burke, M. D., 7-alkoxyquinolines: New fluorescent substrates for cytochrome p450 monooxygenases. *Biochem. Pharmacol.* **1990**, *40*, 1645-1655.

3. Bhattacharyya, D.; Nandi, S.; Adhikari, P.; Sarmah, B. K.; Konwar, M.; Das, A., Boric acid catalyzed chemoselective reduction of quinolines. *Org. Biomol. Chem*. **2020**, *18*, 1214-1220.

4. Ji, Y.-G.; Wei, K.; Liu, T.; Wu, L.; Zhang, W.-H., "Naked" Iridium(IV) Oxide Nanoparticles as Expedient and Robust Catalysts for Hydrogenation of Nitrogen Heterocycles: Remarkable Vicinal Substitution Effect and Recyclability. *Adv. Synth. Catal*. **2017**, *359*, 933-940.

5. Iosub, A. V.; Stahl, S. S., Catalytic Aerobic Dehydrogenation of Nitrogen Heterocycles Using Heterogeneous Cobalt Oxide Supported on Nitrogen-Doped Carbon. *Org. Lett*. **2015**, *17*, 4404-4407.

6. Gong, Y.; He, J.; Wen, X.; Xi, H.; Wei, Z.; Liu, W., Transfer hydrogenation of N-heteroarenes with 2-propanol and ethanol enabled by manganese catalysis. *Org. Chem. Front.* **2021**, *8*, 6901-6908.

7. Kawauchi, D.; Noda, K.; Komatsu, Y.; Yoshida, K.; Ueda, H.; Tokuyama, H., Aerobic Dehydrogenation of *N*-Heterocycles with Grubbs Catalyst: Its Application to Assisted-Tandem Catalysis to Construct *N*-Containing Fused Heteroarenes. *Chem. Eur. J.* **2020**, *26*, 15793-15798.

8. Cui, X.; Huang, W.; Wu, L., Zirconium-hydride-catalyzed transfer hydrogenation of quinolines and indoles with ammonia borane. *Org. Chem. Front*. **2021**, *8*, 5002-5007.

9. Jung, D.; Kim, M. H.; Kim, J., Cu-Catalyzed Aerobic Oxidation of Di-*tert*-butyl Hydrazodicarboxylate to Di-*tert*-butyl Azodicarboxylate and Its Application on Dehydrogenation of 1,2,3,4-Tetrahydroquinolines under Mild Conditions. *Org. Lett*. **2016**, *18*, 6300-6303.

10. Prasada Rao Lingam, V. S.; Thomas, A.; Mukkanti, K.; Gopalan, B., Simple and Convenient Approach for Synthesis of Tetrahydroquinoline Derivatives and Studies on Aza-Cope Rearrangement. *Synth. Commun*. **2011**, *41*, 1809-1828.

11. Ohe, K.; Uemura, S.; Sugita, N.; Masuda, H.; Taga, T., Sodium arenetellurolate-catalyzed selective conversion of nitro aromatics to aromatic azoxy or azo compounds and its application for facile preparation of 3, 3′-and 4, 4’-bis [beta(aryltelluro) vinyl] azobenzenes from (3-and 4-nitrophenyl) acetylenes. *J. Org. Chem.* **1989**, *54*, 4169–4174.

12. Roscales, S.; Csákÿ A. G., Synthesis of Mono -*N*-Methyl Aromatic Amines from Nitroso Compounds and Methylboronic Acid. *ACS Omega* **2019**, *4*, 13943-13953.

13. (a) Song, Q.-W.; Yu, B.; Li, X.-D.; Ma, R.; Diao, Z.-F.; Li, R.-G.; Li, W.; He, L.-N., Efficient chemical fixation of CO_2 promoted by a bifunctional Ag_2WO_4/Ph_3P system. *Green Chem*. **2014**, *16*, 1633-1638. (b) Wu Z. J.; Xu, H.-C., Synthesis of C3-Fluorinated Oxindoles through Reagent-Free Cross-Dehydrogenative Coupling. *Angew. Chem. Int. Ed.* **2017**, *129*, 1-6.

14. Inoue, F.; Kashihara, M.; Yadav, M. R.; Nakao, Y., Buchwald-Hartwig Amination of Nitroarenes. *Angew. Chem. Int. Ed.* **2017**, *56*, 13307-13309.

15. Su, C.; Ye, Y.; Xu, L.; Zhang, C., Synthesis and charge–discharge properties of a ferrocene-containing polytriphenylamine derivative as the cathode of a lithiumion battery. *J. Mater. Chem.* **2012**, *22*, 22658-22662.

16. Kashihara, M.; Yadav, M. R.; Nakao, Y., Reductive Denitration of Nitroarenes. *Org. Lett*. **2018**, *20*, 1655-1658.

17. Perdew, J. P.; Burke, K.; Ernzerhof, M. Generalized Gradient Approximation Made Simple. *Phys. Rev. Lett.* **1996**, 77, 3865-3868.

18. Grimme, S.; Antony, J.; Ehrlich, S.; Krieg, H., A consistent and accurate ab initio parametrization of density functional dispersion correction (DFT-D) for the 94 elements H-Pu. *J. Chem. Phys.* **2010**, *132*, 154104.

19. Johnson, E. R.; Becke, A. D., A post-Hartree-Fock model of intermolecular interactions. *J. Chem. Phys.* **2005**, *123,* 024101.

20. Becke, A. D.; Johnson, E. R., A density-functional model of the dispersion interaction. *J. Chem. Phys.* **2005**, *123*, 154101.

21. Johnson, E. R.; Becke, A. D., A post-Hartree-Fock model of intermolecular interactions: Inclusion of higher-order corrections. *J. Chem. Phys.* **2006**, *124*, 174104.

22. Fukui K., The path of chemical reactions-the IRC approach. *Acc. Chem. Res.* **1981**, *14,* 363-368.

23. Gonzalez, C.; Schlegel, H. B., An improved algorithm for reaction path following. *J. Chem. Phys.* **1989**, *90,* 2154-2161.

24. Weigend, F.; Ahlrichs, R., Balanced basis sets of split valences, triple zeta valence and quadruple zeta valence quality for H to Rn: Design and assessment of accuracy. *Phys. Chem. Chem. Phys.* **2005**, *7,* 3297-3305.

25. Marenich, A. V.; Cramer, C. J.; Truhlar, D. G., Universal solvation model based on solute electron density and on a Continuum model of the solvent defined by the bulk dielectric constant and atomic surface tensions. *J. Phys. Chem. B,* **2009**, *113,* 6378-6396.

26. (a) Frisch, M. J.; Trucks, G. W.; Schlegel, H. B.; Scuseria, G. E.; Robb, M. A.; Cheeseman, J. R.; Scalmani, G.; Barone, V.; Petersson, G. A.; Nakatsuji, H.; Li, X.; Caricato, M.; Marenich, A. V.; Bloino, J.; Janesko, B. G.; Gomperts, R.; Mennucci, B.; Hratchian, H. P.; Ortiz, J. V.; Izmaylov, A. F.; Sonnenberg, J. L.; Williams-Young, D.; Ding, F.; Lipparini, F.; Egidi, F.; Goings, J.; Peng, B.; Petrone, A.; Henderson, T.; Ranasinghe, D.; Zakrzewski, V. G.; Gao, J.; Rega, N.; Zheng, G.; Liang, W.; Hada, M.; Ehara, M.; Toyota, K.; Fukuda,

R.; Hasegawa, J.; Ishida, M.; Nakajima, T.; Honda, Y.; Kitao, O.; Nakai, H.; Vreven, T.; Throssell, K.; Montgomery, J. A. Jr.; Peralta, J. E.; Ogliaro, F.; Bearpark, M. J.; Heyd, J. J.; Brothers, E. N.; Kudin, K. N.; Staroverov, V. N.; Keith, T. A.; Kobayashi, R.; Normand, J.; Raghavachari, K.; Rendell, A. P.; Burant, J. C.; Iyengar, S. S.; Tomasi, J.; Cossi, M.; Millam, J. M.; Klene, M.; Adamo, C.; Cammi, R.; Ochterski, J. W.; Martin, R. L.; Morokuma, K.; Farkas, O.; Foresman, J. B.; Fox, D. J. Gaussian 16, Revision C.01, Gaussian, Inc, Wallingford CT, **2019**. (b) Hirshfeld, F. L. Bonded-Atom Fragments for Describing Molecular Charge Densities. *Theoret. Chim. Acta (Berl.)* **1977**, *44*, 129-138. (c) Mayer, I. Bond Orders and Valences from Ab Initio Wave Functions. *Int. J. Quantum Chem.* **1986**, *29*, 477-483. (d) R. F. W. Bader, Atoms in Molecules, *Acc. Chem. Res.* **1985**, *18*, 9–15. (e) R. F. W. Bader, Atoms in Molecules: A Quantum Theory, Oxford University Press, Oxford, UK, **1990**. (f) R. F. W. Bader, A Quantum Theory of Molecular Structure and Its Applications, *Chem. Rev.* **1991**, *91*, 893–928.

27. Lu, T.; Chen, F., Multiwfn: A multifunctional wavefunction analyzer. *J. Comput. Chem.* **2012**, *33*, 580-592.

28. Legault, C.Y.; CYLview, version 1.0b, Université de Sherbrooke, **2009**.

29. Kumar, S.; Rathore, V.; Verma, A.; Prasad Ch, D.; Kumar, A.; Yadav, A.; Jana, S.; Sattar, M.; Meenakshi; Kumar, S., KO^{t}Bu-Mediated Aerobic Transition-Metal-Free Regioselective *β*-Arylation of Indoles: Synthesis of *β*-(2-/4-Nitroaryl)-indoles. *Org. Lett*. **2015**, *17*, 82-85.

30. (a) Prakash, S. G. K.; Fogassy, G.; Olah, G. A., Microwave-Assisted Nafion-H Catalyzed Friedel–Crafts Type Reaction of Aromatic Aldehydes with Arenes: Synthesis of Triarylmethanes. *Catal. Lett.* **2010**, *138*, 155-159. (b) Mutai K.; Kobayashi K. Photoinduced Intramolecular Aromatic Nucleophilic Substitution (the Photo-Smiles Rearrangement) in Amino Ethers. *Bull. Chem. Soc. Jpn.* **1981**, *54*, 462–465.

31. Baig, R. B. N.; Vaddula, B. R.; Nadagouda, M. N.; Varma, R. S., The copper–nicotinamide complex: sustainable applications in coupling and cycloaddition reactions. *Green Chem.* **2015**, *17*, 1243-1248.

32. Pilkington, L. I.; Sparrow, K.; Rees, S. W. P.; Paulin, E. K.; van Rensburg, M.; Xu, C. S.; Langley, R. J.; Leung, I. K. H.; Reynisson, J.; Leung, E.; Barker, D., Development, synthesis and biological investigation of a novel class of potent PC-PLC inhibitors. *Eur. J. Med. Chem.* **2020**, *191*, 112162.

33. Purkait, N.; Kervefors, G.; Linde, E.; Olofsson, B., Regiospecific *N*‐Arylation of Aliphatic Amines under Mild and Metal‐Free Reaction Conditions. *Angew. Chem., Int. Ed.* **2018**, *57*, 11427-11431.

34. Maclean, D.; Zhu, J.; Chen, M.; Hale, R.; Satymurthy, N.; Barrio, J. R., Safety-Catch Linker Strategies for the Production of Radiopharmaceuticals Labeled with Positron-Emitting Isotopes. *J. Am. Chem. Soc.* **2003,** *12*, 10168-10169.

35. Singer, R. A.; Tom, N. J.; Frost, H. N.; Simon, W. M., Discovery and synthesis of novel phosphine-based ligands for aryl aminations. *Tetrahedron Lett.* **2004**, *45*, 4715-4718.

36. Trump, R. P.; Blanc, J.-B. E.; Stewart, E. L.; Brown, P. J.; Caivano, M.; Gray, D. W.; Hoekstra, W. J.; Willson, T. M.; Han, B.; Turnbull, P., Design and Synthesis of an Array of Selective Androgen Receptor Modulators. *J. Comb. Chem.* **2006,** *9* , 107-114.

37. Huang, C. Q.; Baker, T.; Schwarz, D.; Fan, J.; Heise, C. E.; Zhang, M.; Goodfellow, V. S.; Markison, S.; Gogas, K. R.; Chen, T.; Wang, X.-C.; Zhu, Y.-F., 1-(4-Amino-phenyl)-pyrrolidin-3-yl-amine and 6-(3-amino-pyrrolidin-1-yl)-pyridin-3-yl-amine derivatives as melanin-concentrating hormone receptor-1 antagonists. Bioorg. *Med. Chem. Lett.* **2005**, *15*, 3701-3706.

38. Zhou, L.; Thakur, M., Molecular orientation in single crystal thin films of *N*-(4-nitrophenyl)-(*L*)-prolinol. *J. Mater. Res.* **1998**, *13*, 131-134.

39. Zhang, Y.; Zheng, L.; Yang, F.; Zhang, Z.; Dang, Q.; Bai, X., Substituent-directed reduction of cyclic aminals leading to two different heterocycles selectively: syntheses of functionalized nicotines and pyrido[2,3-b]azepines. *Tetrahedron* **2015**, *71*, 1930-1939.

40. Katritzky, A. R.; Laurenzo, K. S., Alkylaminonitrobenzenes by vicarious nucleophilic amination with 4-(alkylamino)-1,2,4-triazoles. *J. Org. Chem.* **1988,** *53*, 3978-3982.

41. Meenakshi, R.; Shakeela, K.; Kutti Rani, S.; Ranga Rao, G., Oxidation of Aniline to Nitrobenzene Catalysed by 1-Butyl-3-methyl imidazolium phosphotungstate Hybrid Material Using m-chloroperbenzoic Acid as an Oxidant. *Catal. Lett.* **2017**, *148*, 246-257.

42. Akram, M. O.; Das, A.; Chakrabarty,I.; Patil, N. T. Ligand-Enabled Gold-Catalyzed C(sp^2)−N Cross-Coupling Reactions of Aryl Iodides with Amines. *Org. Lett*. **2019**, 21, 8101−8105.
